# Supplementary material for: Mn(I)-catalyzed sigmatropic rearrangement of β, γ-unsaturated alcohols
Source: Nat Commun. 2023 Apr 3;14:1862. doi: 10.1038/s41467-023-37299-x (PMC10070501; doi:10.1038/s41467-023-37299-x)
Supplement: Supplementary file 1 — Supplementary Information [file 41467_2023_37299_MOESM1_ESM.pdf]

Supplementary Information

Mn(I)-Catalyzed Sigmatropic Rearrangment of β, γ-Unsaturated Alcohols

Can Yang[1], Xiaoyu Zhou[2], Lixing Shen[1], Zhuofeng Ke[2]*, Huanfeng Jiang[1], and Wei Zeng[1]*

[1] *Key Laboratory of Functional Molecular Engineering of Guangdong Province, School of Chemistry and Chemical Engineering, South China University of Technology, Guangzhou 510641, China*

[2] *School of Materials Science and Engineering, PFCM Lab, Sun Yat-sen University, Guangzhou 510275, China*

**Corresponding author. Email: kezhf3@mail.sysu.edu.cn (Z. K.); zengwei@scut.edu.cn (W. Z.)*

I. Supplementary Methods 2

1.1. General experimental information 2

1.2. Experimental procedures for the preparation of starting materials 3

1.3. Optimization of reaction conditions 20

1.4. Detail characterization for the sigmatropic rearrangement products **3** and **5** 21

1.5. Detail characterization for the sigmatropic rearrangement products **7** 29

1.6. Post-synthetic applications of the **3a**, **3q** and **7a** 35

1.7. Control experiments for the mechanism studies 38

1.8. Single crystal structure and data 41

1.9. Computational detail 44

II. ^{1}H NMR and ^{13}C NMR Spectrum of All Products. 50

III. HR-MS Spectrum of the New Products 189

IV. Supplementary References 288

I. Supplementary Methods

1.1. General experimental information

All reactions were carried out in flame-dried sealed tubes with magnetic stirring. Unless otherwise noted, all experiments were performed under argon atmosphere. Reagents were purchased from Accela, Acros, Aladdin, Adamas, Energy Chemical or TCI. All solvents were treated with 4 Å molecular sieves or sodium and distilled prior to use. Purifications of reaction products were carried out by flash chromatography using the silica gel (300-400 mesh) from Qingdao Haiyang Chemical Co. Ltd. Infrared spectra (IR) were recorded on a Brucker TENSOR 27 FTIR spectrophotometer and are reported as wavelength numbers (cm^{-1}). Infrared spectra were recorded by preparing a KBr pellet containing the title compounds. ^{1}H NMR and ^{13}C NMR spectra were recorded with tetramethylsilane (TMS) as internal standard at ambient temperature on a Bruker Avance III 400 MHz or 500 MHz for ^{1}H NMR and 100 MHz or 126 MHz for ^{13}C NMR. Chemical shifts are reported in parts per million (ppm) and coupling constants are reported as Hertz (Hz). Splitting patterns are designated as singlet (s), doublet (d), triplet (t), doublet of doublet (dd), quartet (q). Splitting patterns that could not be interpreted or easily visualized are designated as multiple (m). High resolution mass spectra (HRMS) were recorded on an IF-TOF spectrometer (Micromass).

1.2. Experimental procedures for the preparation of starting materials

General procedure for the synthesis of alcohols

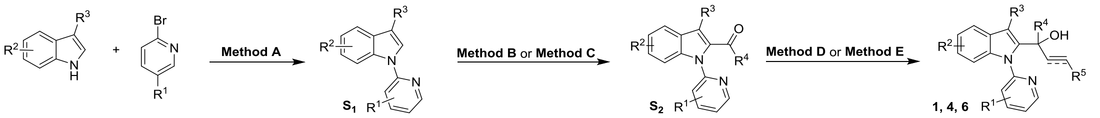

Method A:

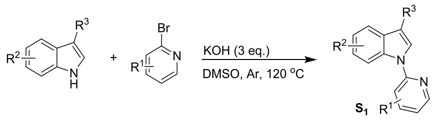

Synthetic procedure of substituted *N*-pyridyl indoles S1[1]: A pressure tube was charged with the corresponding indoles (10 mmol), KOH (3.0 eq.), 2-bromopyridines (2.0 eq.), and DMSO (10.0 mL) under Ar atmosphere. The resulting mixture was stirred in an oil bath at 120 ^{o}C until the end of the reaction. The mixture was quenched with a saturated solution of NH_4Cl and extracted with ethyl acetate. The organic phase was dried over $MgSO_4$, followed by evaporation under reduced pressure to remove the solvent. The product was purified by column chromatography on silica gel (petroleum ether /ethyl acetate = 100: 1 - 50: 1) to afford the corresponding indoles **S1**.

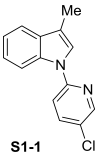
S1-1

1-(5-Chloropyridin-2-yl)-3-methyl-1H-indole (S1-1): white solid; m.p. 44.1 – 46.2 ^{o}C; 1.33 g, 55% yield; ^{1}H NMR (500 MHz, $CDCl_3$) δ 8.46 (d, *J* = 2.3 Hz, 1H), 8.18 (d, *J* = 8.3 Hz, 1H), 7.71 (dd, *J* = 8.7, 2.5 Hz, 1H), 7.58 (d, *J* = 7.8 Hz, 1H), 7.43 (d, *J* = 0.8 Hz, 1H), 7.35 (d, *J* = 8.7 Hz, 1H), 7.30 (t, *J* = 7.4 Hz, 1H), 7.23 (d, *J* = 8.0 Hz, 1H), 2.36 (d, *J* = 1.0 Hz, 3H); ^{13}C NMR (126 MHz, $CDCl_3$) δ 150.9, 147.4, 138.0, 135.3, 131.2, 126.7, 123.5, 122.9, 121.2, 119.2, 115.5, 114.5, 113.2, 9.7; HR-MS [ESI-MS(+)] calcd for $[M + H]^+$: $C_{14}H_{12}ClN_2$: 243.0684, found: 243.0681; IR (KBr): 3048, 1578, 1473, 1449, 1395, 1349, 1214, 1111, 918, 741 cm^{-1}.

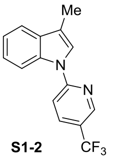
S1-2

3-Methyl-1-(5-(trifluoromethyl)pyridin-2-yl)-1H-indole (S1-2): light yellow solid; m.p. 58.3 – 59.6 ^{o}C; 2.15 g, 78% yield; ^{1}H NMR (400 MHz, $CDCl_3$) δ 8.81 (dd, *J* = 1.5, 0.8 Hz, 1H), 8.45 – 8.40 (m, 1H), 7.97 – 7.90 (m, 1H), 7.64 (d, *J* = 7.7 Hz, 1H), 7.50 (s, 1H), 7.41 (dd, *J* = 17.2, 8.8 Hz, 2H), 7.32 (t, *J* = 7.4 Hz, 1H), 2.41 (d, *J* = 1.2 Hz, 3H); ^{13}C NMR (101 MHz, CDCl3) δ 154.8, 146.1 (q, *J* = 4.6 Hz, $^{3}J_{CF}$), 135.5, 135.4 (q, *J* = 3.6 Hz, $^{3}J_{CF}$), 131.6, 123.9, 123.4 (d, *J* = 338.4 Hz, $^{1}J_{CF}$),122.4, 121.8, 121.3, 119.2, 116.7, 114.3, 112.2, 9.7; HR-MS [ESI-MS(+)] calcd for $[M + H]^+$: $C_{15}H_{12}F_3N_2$: 277.0947, found: 277.0941; IR (KBr): 2922, 1738, 1604, 1497, 1454, 1403, 1324, 1120, 1079, 1014, 742 cm^{-1}.

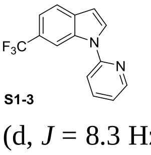
S1-3

1-(Pyridin-2-yl)-6-(trifluoromethyl)-1H-indole (S1-3): light yellow solid; m.p. 114.2 – 115.7 ^{o}C ; 1.97 g, 75% yield; ^{1}H NMR (400 MHz, $CDCl_3$) δ 8.63 (d, *J* = 4.9 Hz, 2H), 7.90 – 7.86 (m, 1H), 7.83 (d, *J* = 3.5 Hz, 1H), 7.76 (d, *J* = 8.3 Hz, 1H), 7.48 (d, *J* = 8.3 Hz, 2H), 7.25 (dd, *J* = 7.3, 4.9 Hz, 1H), 6.79 (d, *J* = 3.4 Hz, 1H); ^{13}C NMR (101 MHz, $CDCl_3$) δ 152.1, 149.1, 138.7, 134.2, 132.7, 128.3, 125.2 (d, *J* = 272.7 Hz, $^{1}J_{CF}$), 125.2 (d, *J* = 32.3 Hz, $^{2}J_{CF}$), 121.3, 120.7, 118.0 (q, *J* = 4.1 Hz, $^{3}J_{CF}$), 114.5, 111.1 (q, *J* = 4.6 Hz, $^{3}J_{CF}$), 105.5; HR-MS [ESI-MS(+)] calcd for $[M + H]^+$: $C_{14}H_{10}F_3N_2$: 263.0791, found: 263.0785; IR (KBr): 2955, 2916, 2849, 1590, 1477, 1452, 1352, 1329, 1213, 1145, 1103, 893, 818, 772, 732 cm^{-1}.

Method B:

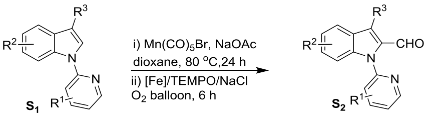

Synthetic procedure of substituted *N*-pyridyl 2-formylindoles S2：The following starting materials **S2** were prepared according to the literature procedure [2]. To a 100 mL dry Schlenk tube with a stirring bar, $Mn(CO)_5Br$ (5 mmol %), NaOAc (10 mmol %), $(CH_2O)_n$ (3.0 eq.), and *N*-pyridyl indoles **S1** (5 mmol) were added under air. After that, the reaction vessel was then evacuated and filled with argon for three times. Afterwards, dioxane (15 mL, anhydrous) was added sequentially under an argon atmosphere. The tube was sealed and the mixture was stirred at 80 ℃ for 15 h. After the reaction was cooled down to room temperature, $Fe(NO_3)_3 \cdot 9H_2O$ (10 mol %), TEMPO (10 mol %), and NaCl (10 mol %) were added into the reaction mixture, and the reaction vessel was then quickly evacuated and filled with oxygen using a balloon. The reaction was run for additional 6 h at room temperature. The reaction mixture was evaporated and purified *via* column chromatography on silica gel (eluent: petroleum ether/ethyl acetate = 10/1) afforded the pure desired products **S2**.

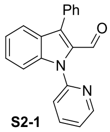

S2-1

3-Phenyl-1-(pyridin-2-yl)-1H-indole-2-carbaldehyde (S2-1): white solid; m.p. 147.3 - 149.5 °C; 983.0 mg, 66% yield; ^1H NMR (400 MHz, $CDCl_3$) δ 9.94 (s, 1H), 8.69 (dd, *J* = 4.9, 1.2 Hz, 1H), 7.97 – 7.92 (m, 1H), 7.82 (d, *J* = 8.1 Hz, 1H), 7.70 – 7.65 (m, 2H), 7.60 – 7.55 (m, 2H), 7.54 – 7.42 (m, 5H), 7.30 – 7.36 (m, 1H); ^{13}C NMR (101 MHz, $CDCl_3$) δ 182.4, 151.2, 149.2, 139.8, 138.2, 133.6, 131.6, 131.4, 130.9, 128.7, 128.3, 128.3, 126.7, 123.1, 122.3, 122.3, 122.3, 111.9; HR-MS [ESI-MS(+)] calcd for $[M + H]^+$: $C_{20}H_{15}N_2O$: 299.1179, found: 299.1188; IR (KBr): 3054, 2842, 1667, 1587, 1469, 1406, 1379, 1338, 1239, 1180, 1149, 1024, 926, 773, 749, 702 cm^{-1}.

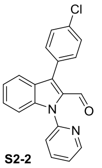

S2-2

3-(4-Chlorophenyl)-1-(pyridin-2-yl)-1H-indole-2-carbaldehyde (S2-2): light yellow oil; 650.0 mg, 39% yield; ^1H NMR (500 MHz, $CDCl_3$) δ 9.88 (s, 1H), 8.66 (d, *J* = 3.8 Hz, 1H), 7.95 – 7.91 (m, 1H), 7.74 (d, *J* = 8.2 Hz, 1H), 7.58 (d, *J* = 8.3 Hz, 2H), 7.52 (d, *J* = 8.3 Hz, 2H), 7.46 – 7.39 (m, 4H), 7.28 – 7.25 (m, 1H); ^{13}C NMR (126 MHz, $CDCl_3$) δ 181.9, 151.0, 149.3, 139.6, 138.3, 134.5, 132.1, 131.4, 130.1, 129.0, 128.4, 126.5, 123.2, 122.5, 122.2, 121.9, 111.9; HR-MS [ESI-MS(+)] calcd for $[M + H]^+$: $C_{20}H_{14}ClN_2O$: 333.0789, found: 333.0787; IR (KBr): 2924, 1734, 1662, 1585, 1467, 1442, 1379, 1236, 917, 743 cm^{-1}.

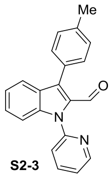

S2-3

1-(Pyridin-2-yl)-3-(*p*-tolyl)-1H-indole-2-carbaldehyde (S2-3): light yellow oil; 730.0 mg, 47% yield; ^1H NMR (400 MHz, $CDCl_3$) δ 9.94 (s, 1H), 8.70 – 8.68 (m, 1H), 7.96 – 7.92 (m, 1H), 7.82 (d, *J* = 8.1 Hz, 1H), 7.57 (d, *J* = 8.0 Hz, 2H), 7.48 – 7.37 (m, 6H), 7.29 – 7.25 (m, 1H), 2.50 (s, 3H); ^{13}C NMR (101 MHz, $CDCl_3$) δ 182.4, 151.3, 149.2, 139.8, 138.3, 138.1, 133.8, 131.4, 130.9, 130.8, 129.5, 128.8, 128.6, 128.2, 126.7, 123.0, 122.3, 122.3, 122.2, 111.9, 21.4.; HR-MS [ESI-MS(+)] calcd for $[M + H]^+$: $C_{21}H_{17}N_2O$: 313.1335, found: 313.1329; IR (KBr): 2931, 1734, 1675, 1663, 1677, 1577, 1466, 1438, 1379, 1350, 1187, 930, 747 cm^{-1}.

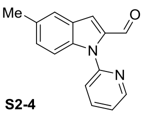

S2-4

5-Methyl-1-(pyridin-2-yl)-1H-indole-2-carbaldehyde (S2-4): yellow solid; m.p. 90.0 – 91.4 °C; 826.0 mg, 70% yield; ^1H NMR (400 MHz, $CDCl_3$) δ 9.95 (s, 1H), 8.69 – 8.64 (m, 1H), 7.94 – 7.90 (m, 1H), 7.58 (s, 1H), 7.46 – 7.37 (m, 4H), 7.25 (dd, *J* = 8.7, 1.3 Hz, 1H), 2.48 (s, 3H); ^{13}C NMR (101 MHz, $CDCl_3$) δ 181.6, 150.7, 149.2, 138.7, 138.2, 136.2, 131.7, 129.6, 127.2, 122.6, 121.5, 117.1, 111.7, 21.3; HR-MS [ESI-MS(+)] calcd for $[M + H]^+$: $C_{15}H_{13}N_2O$: 237.1022, found: 237.1010; IR (KBr): 2922, 1735, 1700, 1685, 1676, 1587, 1560, 1469, 1439, 1371, 1293, 1118, 989, 915, 774 cm^{-1}.

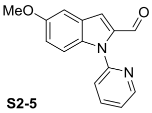
S2-5

5-Methoxy-1-(pyridin-2-yl)-1H-indole-2-carbaldehyde (S2-5): light yellow solid; m.p. 105.9 – 107.1 ^{o}C; 504.00 mg, 40% yield; ^{1}H NMR (400 MHz, $CDCl_3$) δ 9.93 (s, 1H), 8.65 (dd, *J* = 4.5, 1.3 Hz, 1H), 7.94 – 7.88 (m, 1H), 7.47 – 7.41 (m, 3H), 7.40 – 7.36 (m, 1H), 7.16 (d, *J* = 2.4 Hz, 1H), 7.08 (dd, *J* = 9.1, 2.5 Hz, 1H), 3.89 (s, 3H); ^{13}C NMR (101 MHz, $CDCl_3$) δ 181.6, 155.6, 150.6, 149.2, 138.3, 136.4, 135.6, 127.4, 122.7, 121.4, 119.2, 116.7, 113.1, 103.0, 55.7; HR-MS [ESI-MS(+)] calcd for $[M + H]^+$: $C_{15}H_{13}N_2O_2$: 253.0972, found: 253.0965; IR (KBr): 2920, 1672, 1587, 1524, 1468, 1437, 1399, 1335, 1207, 1173, 1125, 1027, 912, 772, 739 cm^{-1}.

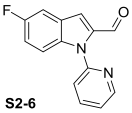
S2-6

5-Fluoro-1-(pyridin-2-yl)-1H-indole-2-carbaldehyde (S2-6): light yellow solid; m.p. 100.4 – 101.5 ^{o}C; 768.0 mg, 64% yield; ^{1}H NMR (400 MHz, $CDCl_3$) δ 9.95 (s, 1H), 8.66 (dd, *J* = 4.8, 1.1 Hz, 1H), 7.95 – 7.91 (m, 1H), 7. 50 – 7.40 (m, 5H), 7.19 – 7.14 (m, 1H); ^{13}C NMR (101 MHz, $CDCl_3$) δ 181.6, 158.8 (d, *J* = 240.4 Hz, $^{1}J_{CF}$), 150.4, 149.3, 138.4, 137.2, 136.7, 127.1 (d, *J* = 11.1 Hz, $^{2}J_{CF}$), 123.0, 121.6, 116.7 (d, *J* = 27.3 Hz, $^{2}J_{CF}$), 116.6 (d, *J* = 6.1 Hz, $^{3}J_{CF}$), 113.3 (d, *J* = 9.1 Hz, $^{3}J_{CF}$), 107.5 (d, *J* = 24.4 Hz, $^{2}J_{CF}$); HR-MS [ESI-MS(+)] calcd for $[M + H]^+$: $C_{14}H_{10}FN_2O$: 241.0772, found: 241.0763 ; IR (KBr): 3062, 2922, 1677, 1588, 1523, 1466, 1455, 1399, 1344, 1285, 1206, 1175, 1120, 1023, 911, 858, 766, 738 cm^{-1}.

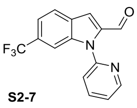
S2-7

1-(Pyridin-2-yl)-6-(trifluoromethyl)-1H-indole-2-carbaldehyde (S2-7): light yellow solid; m.p. 103.8 – 105.0 ^{o}C; 770.0 mg, 53% yield; ^{1}H NMR (400 MHz, $CDCl_3$) δ 10.00 (s, 1H), 8.70 (dd, *J* = 5.1, 1.5 Hz, 1H), 7.99 – 7.95 (m, 1H), 7.91 (d, *J* = 8.4 Hz, 1H), 7.80 (s, 1H), 7.53 (s, 1H), 7.51 – 7.44 (m, 3H); ^{13}C NMR (101 MHz, $CDCl_3$) δ 181.7, 149.9, 149.6, 138.8, 138.7, 137.9, 129.1(d, *J* = 32.3 Hz, $^{2}J_{CF}$), 128.9, 124.4 (d, *J* = 273.7 Hz, $^{1}J_{CF}$), 124.1, 123.4, 121.6, 118.7 (q, *J* = 3.0 Hz, $^{3}J_{CF}$), 116.1, 109.9 (q, *J* = 5.1 Hz, $^{3}J_{CF}$); HR-MS [ESI-MS(+)] calcd for $[M + H]^+$: $C_{15}H_{10}F_3N_2O$: 291.0740, found: 291.0732; IR (KBr): 2956, 2918, 2847, 1678, 1587, 1521, 1468, 1443, 1363, 1159, 1112, 1020, 968, 876, 853, 790, 741 cm^{-1}.

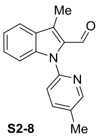
S2-8

3-Methyl-1-(5-methylpyridin-2-yl)-1H-indole-2-carbaldehyde (S2-8): light yellow solid; m.p. 100.0 – 103.2 ^{o}C; 837.0 mg, 67% yield; ^{1}H NMR (400 MHz, $CDCl_3$) δ 10.13 (s, 1H), 8.47 (s, 1H), 7.77 (d, *J* = 8.0 Hz, 1H), 7.71 (d, *J* = 7.9 Hz, 1H), 7.44 – 7.37 (m, 2H), 7.32 (d, *J* = 8.0 Hz, 1H), 7.25 (t, *J* = 7.1 Hz, 1H), 2.75 (s, 3H), 2.46 (s, 3H); ^{13}C NMR (101 MHz, $CDCl_3$) δ 181.7, 149.6, 148.6, 139.2, 138.8, 132.3, 131.9, 128.0, 127.7, 127.0, 121.4, 121.3, 120.9, 111.6, 18.1, 9.3; HR-MS [ESI-MS(+)] calcd for $[M + H]^+$: $C_{16}H_{15}N_2O$: 251.1179, found: 251.1171; IR (KBr): 2918, 1867, 1828, 1668, 1540, 1477, 1446, 1384, 1339, 1224, 1138, 1024, 932, 884, 824, 741 cm^{-1}.

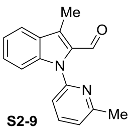
S2-9

3-Methyl-1-(6-methylpyridin-2-yl)-1H-indole-2-carbaldehyde (S2-9): light yellow solid; m.p. 66.6 – 68.1 ^{o}C; 688.0 mg, 55% yield; ^{1}H NMR (500 MHz, $CDCl_3$) δ 10.11 (s, 1H), 7.79 – 7.71 (m, 2H), 7.45 (d, *J* = 8.5 Hz, 1H), 7.40 – 7.35 (m, 1H), 7.24 – 7.17 (m, 3H), 2.72 (s, 3H), 2.60 (s, 3H); ^{13}C NMR (126 MHz, $CDCl_3$) δ 182.1, 158.8, 150.1, 138.8, 138.4, 131.9, 128.1, 127.7, 126.7, 122.0, 121.5, 121.4, 118.2, 111.6, 24.3, 9.4; HR-MS [ESI-MS(+)] calcd for $[M + H]^+$: $C_{16}H_{15}N_2O$: 251.1179, found: 251.1175; IR (KBr): 3058, 2920, 1664, 1593, 1569, 1536, 1455, 1337, 1220, 1143, 992, 933, 883, 785, 737 cm^{-1}.

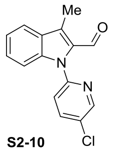
S2-10

1-(5-Chloropyridin-2-yl)-3-methyl-1H-indole-2-carbaldehyde (S2-10): light yellow solid; m.p. 131.2 – 132.1 ^{o}C; 1.22 g, 90% yield; ^{1}H NMR (500 MHz, $CDCl_3$) δ 10.12 (s, 1H), 8.56 (d, *J* = 2.3 Hz, 1H), 7.84 (dd, *J* = 8.5, 2.5 Hz, 1H), 7.76 (d, *J* = 7.7 Hz, 1H), 7.42 (t, *J* = 7.7 Hz, 2H), 7.33 (d, *J* = 8.5 Hz, 1H), 7.26 (d, *J* = 5.4 Hz, 1H), 2.72 (s,

3H); ^{13}C NMR (126 MHz, $CDCl_3$) δ 181.0, 149.3, 148.0, 139.2, 137.9, 131.6, 130.5, 128.6, 128.3, 128.1, 122.2, 121.9, 121.5, 111.6, 9.2; HR-MS [ESI-MS(+)] calcd for $[M + H]^+$: $C_{15}H_{12}ClN_2O$: 271.0633 , found: 271.0629; IR (KBr): 3052, 2924, 1700, 1541, 1466, 1411, 1382, 1338, 1112, 1012, 934, 888, 827, 742 cm^{-1}.

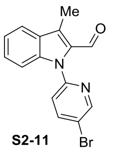
S2-11

1-(5-Bromopyridin-2-yl)-3-methyl-1H-indole-2-carbaldehyde (S2-11): yellow solid; m.p. 118.6 – 120.3 ^oC; 635.0 mg, 40% yield; ^{1}H NMR (400 MHz, $CDCl_3$) δ 10.14 (s, 1H), 8.68 (d, *J* = 2.4 Hz, 1H), 7.99 (dd, *J* = 8.4, 2.4 Hz, 1H), 7.77 (d, *J* = 8.0 Hz, 1H), 7.50 – 7.41 (m, 2H), 7.27 (dd, *J* = 14.5, 6.8 Hz, 2H), 2.74 (s, 3H); ^{13}C NMR (101 MHz, $CDCl_3$) δ 181.0, 150.2, 149.7, 140.7, 139.1, 131.6, 128.6, 128.3, 128.1, 122.7, 121.9, 121.5, 118.8, 111.6, 9.2; HR-MS [ESI-MS(+)] calcd for $[M + H]^+$: $C_{15}H_{12}BrN_2O$: 315.0128, found: 315.0128; IR (KBr): 2921, 2855, 1644, 1541, 1462, 1378, 1338, 1093, 742 cm^{-1}.

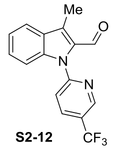
S2-12

3-Methyl-1-(5-(trifluoromethyl)pyridin-2-yl)-1H-indole-2-carbaldehyde (S2-12): white solid; m.p. 93.0 - 95.1 ^oC; 1.14 g, 75% yield; ^{1}H NMR (400 MHz, $CDCl_3$) δ 10.17 (s, 1H), 8.91 – 8.86 (m, 1H), 8.10 (dd, *J* = 8.5, 2.1 Hz, 1H), 7.79 (d, *J* = 8.0 Hz, 1H), 7.61 (d, *J* = 8.5 Hz, 1H), 7.50 – 7.44 (m, 2H), 7.30 (dd, *J* = 12.2, 5.1 Hz, 1H), 2.75 (s, 3H); ^{13}C NMR (101 MHz, $CDCl_3$) δ 180.8, 153.9, 146.2 (q, *J* = 4.0 Hz, $^3J_{CF}$), 139.0, 135.3 (q, *J* = 3.0 Hz, $^3J_{CF}$), 131.6, 129.8, 128.6, 128.4,124.8 (d, *J* = 33.3 Hz, $^2J_{CF}$), 123.4 (d, *J* = 273.7 Hz, $^1J_{CF}$), 122.3, 121.6, 121.0, 111.8, 9.2; HR-MS [ESI-MS(+)] calcd for $[M + H]^+$: $C_{16}H_{12}F_3N_2O$: 305.0896, found: 305.0883; IR (KBr): 2922, 2830, 1668, 1601, 1541, 1488, 1454, 1397, 1324, 1128, 1077, 1013, 743 cm^{-1}.

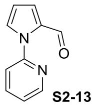
S2-13

1-(Pyridin-2-yl)-1H-pyrrole-2-carbaldehyde (S2-13): yellow oil; 200.0 mg, 23% yield; ^{1}H NMR (400 MHz, $CDCl_3$) δ 9.78 (s, 1H), 8.55 – 8.51 (m, 1H), 7.86 – 7.81 (m, 1H), 7.48 – 7.44 (m, 2H), 7.35 – 7.29 (m, 1H), 7.22 (dd, *J* = 3.8, 1.6 Hz, 1H), 6.44 (dd, *J* = 3.8, 2.7 Hz, 1H); ^{13}C NMR (101 MHz, $CDCl_3$) δ 179.5, 150.9, 148.7, 138.3, 132.2, 130.2, 124.5, 122.7, 119.1, 111.4; HR-MS [ESI-MS(+)] calcd for $[M + H]^+$: $C_{10}H_9N_2O$: 173.0709, found: 173.0712; IR (KBr): 3115, 2924, 1667, 1588, 1528, 1473, 1450, 1438, 1414, 1391, 1337, 1316, 1151, 1107, 1034, 989, 885, 787, 740 cm^{-1}.

Method C:

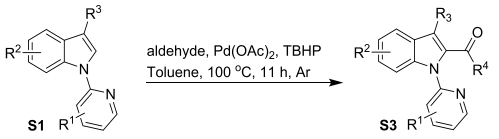

Synthetic procedure of substituted *N*-pyridyl 2-acylindoles S3: The following starting materials **S3** were prepared according to the literature procedures [1]. An oven-dried sealed tube charged with $Pd(OAc)_2$ (5.0 mol %), 70% TBHP aqueous solution (200 mol %), indole **S1** (3.0 mmol) and aldehydes (6.0 mmol), toluene (5.0 mL) were added under Ar atmosphere. The reaction mixture was then allowed to stir at 100 ℃ for 11 h. The corresponding reaction mixture was filtered through a pad of celite, washed with dichloromethane and concentrated under reduced pressure. The residue was purified by flash chromatography on silica gel using petroleum ether/ethyl acetate (20:1 ~ 10:1) as eluent to afford the desired products **S3**.

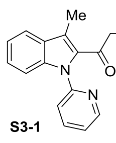
S3-1

1-(3-Methyl-1-(pyridin-2-yl)-1H-indol-2-yl)propan-1-one (S3-1): light yellow solid; m.p. 89.1 - 90.1 ^oC; 875.0 mg, 66% yield; ^{1}H NMR (400 MHz, $CDCl_3$) δ 8.60 – 8.59 (m, 1H), 7.92 – 7.87 (m, 1H), 7.75 – 7.71 (m, 1H), 7.47 – 7.39 (m, 2H), 7.37 – 7.33 (m, 1H), 7.32 – 7.29 (m, 1H), 7.27 – 7.23 (m, 1H), 2.69 – 2.64 (m, 2H), 2.64 (s, 3H),

1.13 (t, *J* = 7.3 Hz, 3H); ^{13}C NMR (101 MHz, $CDCl_3$) δ 196.2, 152.5, 149.4, 138.3, 135.1, 128.8, 126.3, 121.9, 121.4, 121.0, 120.9, 120.3, 111.2, 36.0, 10.6, 8.4; HR-MS [ESI-MS(+)] calcd for $[M + H]^+$: $C_{17}H_{17}N_2O$: 265.1335, found: 265.1333; IR (KBr): 2917, 1682, 1590, 1474, 1437, 1343, 1243, 1220, 1142, 993, 956, 784, 741 cm^{-1}.

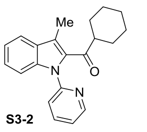

S3-2

Cyclohexyl(3-methyl-1-(pyridin-2-yl)-1H-indol-2-yl)methanone (S3-2): white solid; m.p. 98.2 - 100.7 ^oC; 1.2 g, 75% yield; ^{1}H NMR (500 MHz, $CDCl_3$) δ 8.56 (d, *J* = 4.4 Hz, 1H), 7.85 (t, *J* = 7.7 Hz, 1H), 7.69 (d, *J* = 7.9 Hz, 1H), 7.54 (d, *J* = 8.4 Hz, 1H), 7.39 – 7.30 (m, 2H), 7.23 (dd, *J* = 14.6, 7.0 Hz, 2H), 2.54 (s, 3H), 2.52 – 2.44 (m, 1H), 1.73 (t, *J* = 14.2 Hz, 4H), 1.62 – 1.56 (m, 1H), 1.45 – 1.37 (m, 2H), 1.18 – 1.04 (m, 3H); ^{13}C NMR (126 MHz, $CDCl_3$) δ 199.9, 152.3, 149.4, 138.3, 137.7, 134.8, 129.1, 126.0, 121.6, 121.4, 120.9, 120.4, 119.6, 111.2, 50.2, 28.9, 25.9, 25.8, 10.0; HR-MS [ESI-MS(+)] calcd for $[M + H]^+$: $C_{21}H_{23}N_2O$: 319.1805, found: 319.1804; IR (KBr): 2929, 1734, 1701, 1654, 1577, 1560, 1466, 1438, 1350, 1270, 1130, 744 cm^{-1}.

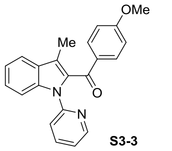

S3-3

(4-Methoxyphenyl)(3-methyl-1-(pyridin-2-yl)-1H-indol-2-yl)methanone (S3-3): light yellow solid; m.p. 134.9 - 136.1 ^oC; 942.0 mg, 55% yield; ^{1}H NMR (400 MHz, $CDCl_3$) δ 8.45 – 8.40 (m, 1H), 7.89 – 7.83 (m, 2H), 7.79 – 7.69 (m, 3H), 7.43 – 7.37 (m, 1H), 7.35 – 7.27 (m, 2H), 7.12 – 7.07 (m, 1H), 6.90 – 6.85 (m, 2H), 3.85 (s, 3H), 2.38 (s, 3H); ^{13}C NMR (101 MHz, $CDCl_3$) δ 188.4, 163.4, 151.5, 149.1, 137.9, 137.4, 134.0, 132.0, 131.8, 129.1, 125.8, 121.4, 121.1, 120.6, 120.4, 119.5, 113.7, 111.4, 55.5, 10.2; HR-MS [ESI-MS(+)] calcd for $[M + H]^+$: $C_{22}H_{19}N_2O_2$: 343.1441, found: 343.1439; IR (KBr): 2929, 1734, 1654, 1577, 1560, 1438, 1150, 1280, 979, 747 cm^{-1}.

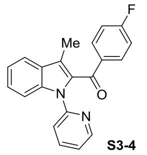

S3-4

(4-Fluorophenyl)(3-methyl-1-(pyridin-2-yl)-1H-indol-2-yl)methanone (S3-4): light yellow solid; m.p. 137.4 - 140.2 ^oC; 1.17 g, 71% yield; ^{1}H NMR (400 MHz, $CDCl_3$) δ 8.40 – 8.38 (m, 1H), 7.89 – 7.83 (m, 2H), 7.77 – 7.70 (m, 3H), 7.44 – 7.28 (m, 3H), 7.12 – 7.02 (m, 3H), 2.40 (s, 3H); ^{13}C NMR (101 MHz, $CDCl_3$) δ 188.1, 165.4 (d, *J* = 255.5 Hz, $^1J_{CF}$), 151.3, 149.2, 138.0, 137.4, 135.4 (d, *J* = 3.0 Hz, $^3J_{CF}$), 133.6, 132.1 (d, *J* = 9.1 Hz, $^3J_{CF}$), 129.0, 126.2, 121.6, 121.5, 121.2, 120.9, 119.4, 115.5 (d, *J* = 22.2 Hz, $^2J_{CF}$), 111.32, 10.19; HR-MS [ESI-MS(+)] calcd for $[M + H]^+$: $C_{21}H_{16}FN_2O$: 331.1241, found: 331.1238; IR (KBr): 2925, 1734, 1685, 1675, 1654, 1617, 1598, 1438, 1268, 1230, 1150, 742 cm^{-1}.

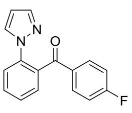

S3-5

(2-(1H-Pyrazol-1-yl)phenyl)(4-fluorophenyl)methanone (S3-5): light yellow oil; 0.60 g, 75% yield; ^{1}H NMR (400 MHz, $CDCl_3$) δ 7.69 – 7.63 (m, 4H), 7.62 – 7.57 (m, 2H), 7.53 – 7.48 (m, 1H), 7.42 (d, *J* = 1.5 Hz, 1H), 7.00 – 6.94 (m, 2H), 6.23 – 6.20 (m, 1H); ^{13}C NMR (101 MHz, $CDCl_3$) δ 194.3, 165.5 (d, *J* = 255.5 Hz, $^1J_{CF}$), 141.3, 138.5, 133.6, 133.3 (d, *J* = 3.0 Hz, $^3J_{CF}$), 131.7, 131.6, 131.4, 129.6 (d, *J* = 22.2 Hz, $^2J_{CF}$), 127.6, 123.2, 115.3 (d, *J* = 21.2 Hz, $^2J_{CF}$), 107.8.HR-MS [ESI-MS(+)] calcd for $[M + H]^+$: $C_{16}H_{12}FN_2O$: 267.0928, found: 267.0926; IR (KBr): 3117, 2924, 1669, 1598, 1519, 1451, 1409, 1306, 1274, 1236, 1149, 1046, 931, 758, 617 cm^{-1}.

Method D：

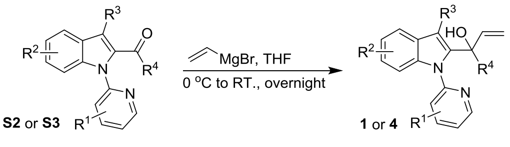

Synthetic procedure of α-(2-indolyl) allylic alcohols 1 and 4： 2-Acylindoles (which were

prepared according to the previous literatures) [1, 3] (1.0 mmol) was dissolved in anhydrous THF (10 mL) in an oven dried round bottom flask under Ar environment. The solution was then cooled to 0 ℃ and a solution of vinylmagnesium bromide in THF (2.0 mL, 2.0 mmol, 1.0 M) was added dropwise. The reaction was warmed to room temperature and stirred overnight. then was quenched by saturated NH_4Cl (aq), extracted by EtOAc for three times (3 × 5 mL), dried over anhydrous Na_2SO_4, and evaporated in vacuum to afford the crude product, which was further purified by flash chromatography on silica gel with petroleum ether/EtOAc (50: 1 ~ 10: 1) to give the corresponding alcohols **1** or **4**.

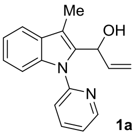

1a

1-(3-Methyl-1-(pyridin-2-yl)-1H-indol-2-yl)prop-2-en-1-ol (1a): light yellow oil; 238.0 mg, 90% yield; ^{1}H NMR (500 MHz, $CDCl_3$) δ 8.49 (dd, *J* = 5.0, 1.1 Hz, 1H), 7.90 – 7.86 (m, 1H), 7.61 (dd, *J* = 6.2, 2.7 Hz, 1H), 7.56 (d, *J* = 8.1 Hz, 1H), 7.45 (dd, *J* = 6.4, 2.3 Hz, 1H), 7.27 – 7.19 (m, 3H), 6.82 (d, *J* = 10.1 Hz, 1H), 5.80 – 5.73 (m, 1H), 5.57 (d, *J* = 9.5 Hz, 1H), 5.01 (d, *J* = 17.3 Hz, 1H), 4.77 (d, *J* = 10.6 Hz, 1H), 2.43 (s, 3H); ^{13}C NMR (126 MHz, $CDCl_3$) δ 152.0, 148.0, 139.1, 138.9, 136.9, 136.0, 129.7, 123.4, 121.5, 121.0, 119.9, 119.7, 113.9, 112.9, 110.0, 66.1, 9.1; HR-MS [ESI-MS(+)] calcd for $[M + H – H_2O]^+$: $C_{17}H_{15}N_2$: 247.1230, found: 247.1226; IR (KBr): 3723, 3634, 3056, 2921, 1591, 1475, 1456, 1439, 1363, 1319, 1223, 1150, 1111, 988, 779, 740 cm^{-1}.

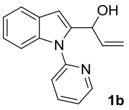

1b

1-(1-(Pyridin-2-yl)-1H-indol-2-yl)prop-2-en-1-ol (1b): light yellow solid; m.p. 60.6 - 61.6 ^{o}C; 223.0 mg, 89% yield; ^{1}H NMR (400 MHz, $CDCl_3$) δ 8.60 – 8.56 (m, 1H), 7.99 – 7.95 (m, 1H), 7.70 – 7.64 (m, 2H), 7.55 – 7.51 (m, 1H), 7.36 – 7.32 (m, 1H), 7.27 – 7.19 (m, 2H), 6.70 (s, 1H), 6.54 (d, *J* = 4.9 Hz, 1H), 6.19 – 6.11 (m, 1H), 5.46 – 5.41 (m, 1H), 5.30 (s, 1H), 5.22 – 5.19 (m, 1H); ^{13}C NMR (101 MHz, $CDCl_3$) δ 151.3, 148.5, 142.3, 139.2, 137.1, 136.7, 128.6, 123.1, 121.7, 121.5, 121.4, 119.9, 115.5, 110.3, 104.8, 67.2; HR-MS [ESI-MS(+)] calcd for $[M + H – H_2O]^+$: $C_{16}H_{13}N_2$: 233.1073, found: 233.1064; IR (KBr): 3671, 3646, 2921, 1866, 1681, 1647, 1541, 1454, 1434, 1373, 1338, 1206, 1145, 736 cm^{-1}.

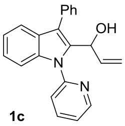

1c

1-(3-Phenyl-1-(pyridin-2-yl)-1H-indol-2-yl)prop-2-en-1-ol (1c): light yellow oil; 218.0 mg, 67 % yield; ^{1}H NMR (400 MHz, $CDCl_3$) δ 8.58 (dd, *J* = 5.0, 1.2 Hz, 1H), 7.99 – 7.95 (m, 1H), 7.75 (d, *J* = 7.7 Hz, 1H), 7.68 (d, *J* = 8.0 Hz, 3H), 7.55 (dd, *J* = 12.5, 5.1 Hz, 3H), 7.42 (t, *J* = 7.4 Hz, 1H), 7.36 – 7.23 (m, 3H), 6.76 (d, *J* = 9.5 Hz, 1H), 5.77 – 5.69 (m, 1H), 5.58 (s, 1H), 4.97 (d, *J* = 17.3 Hz, 1H), 4.76 – 4.69 (m, 1H); ^{13}C NMR (101 MHz, $CDCl_3$) δ 151.9, 148.2, 139.2, 138.9, 137.4, 136.2, 133.9, 130.3, 128.7, 128.3, 127.1, 123.7, 122.0, 121.7, 120.5, 120.2, 120.1, 113.8, 110.1, 66.6; HR-MS [ESI-MS(+)] calcd for $[M + H – H_2O]^+$: $C_{22}H_{17}N_2$: 309.1386, found: 309.1380; IR (KBr): 3729, 3629, 3055, 1591, 1472, 1438, 1369, 1149, 987, 921, 771, 742, 702 cm^{-1}.

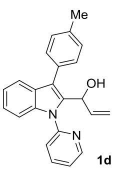

1d

1-(1-(Pyridin-2-yl)-3-(*p*-tolyl)-1H-indol-2-yl)prop-2-en-1-ol (1d): light yellow oil; 262.0 mg, 77% yield; ; ^{1}H NMR (400 MHz, $CDCl_3$) δ 8.58 – 8.56 (m, 1H), 7.98 – 7.93 (m, 1H), 7.76 (d, *J* = 7.8 Hz, 1H), 7.67 (d, *J* = 8.1 Hz, 1H), 7.62 – 7.54 (m, 3H), 7.38 (d, *J* = 8.0 Hz, 2H), 7.34 – 7.24 (m, 3H), 6.79 (dd, *J* = 10.9, 1.2 Hz, 1H), 5.82 – 5.69 (m, 1H), 5.62 (dd, *J* = 10.9, 2.0 Hz, 1H), 4.99 (d, *J* = 17.2 Hz, 1H), 4.73 (d, *J* = 10.6 Hz, 1H), 2.49 (s, 3H); ^{13}C NMR (101 MHz, $CDCl_3$) δ 151.9, 148.2, 139.2, 138.9, 137.3, 136.8, 136.2, 130.9, 130.2, 129.4, 128.4, 123.7, 121.9, 121.6, 120.6, 120.2, 120.1, 113.8, 110.1, 66.7, 21.4; HR-MS [ESI-MS(+)] calcd for $[M + H - H_2O]^+$: $C_{23}H_{19}N_2$: 323.1543, found: 323.1535; IR (KBr): 3049, 3022, 2920, 1591, 1473, 1455, 1439, 1319, 1229, 1108, 989, 831, 784, 743 cm^{-1}.

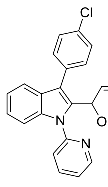
1e

1-(3-(4-Chlorophenyl)-1-(pyridin-2-yl)-1H-indol-2-yl)prop-2-en-1-ol (1e): light yellow solid; m.p. 120.3 - 123.4 ^{o}C; 220.0 mg, 61% yield ; ^{1}H NMR (400 MHz, $CDCl_3$) δ 8.57 (dd, *J* = 5.0, 1.2 Hz, 1H), 7.99 – 7.95 (m, 1H), 7.73 – 7.60 (m, 4H), 7.54 (dd, *J* = 15.1, 8.2 Hz, 3H), 7.38 – 7.24 (m, 3H), 6.72 (d, *J* = 10.9 Hz, 1H), 5.76 – 5.68 (m, 1H), 5.58 – 5.47 (m, 1H), 4.99 (d, *J* = 17.3 Hz, 1H), 4.78 – 4.71 (m, 1H); ^{13}C NMR (101 MHz, $CDCl_3$) δ 151.7, 148.3, 139.3, 138.6, 137.6, 136.2, 133.0, 132.5, 131.6, 128.9, 128.0, 124.0, 122.2, 121.9, 120.3, 120.2, 118.9, 114.1, 110.3, 66.6; HR-MS [ESI-MS(+)] calcd for $[M + H]^+$: $C_{22}H_{18}ClN_2O$: 361.1102, found: 361.1112; IR (KBr): 3661, 3621, 2926, 1654, 1467, 1438, 1382, 1368, 740 cm^{-1}.

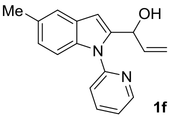
1f

1-(5-Methyl-1-(pyridin-2-yl)-1H-indol-2-yl)prop-2-en-1-ol (1f): light yellow solid; m.p. 90.5 - 91.5 ^{o}C; 262.0 mg, 77% yield; ^{1}H NMR (400 MHz, $CDCl_3$) δ 8.58 – 8.52 (m, 1H), 7.97 – 7.89 (m, 1H), 7.64 (d, *J* = 8.1 Hz, 1H), 7.47 – 7.40 (m, 2H), 7.30 (dd, *J* = 7.4, 5.0 Hz, 1H), 7.08 (dd, *J* = 8.5, 1.3 Hz, 1H), 6.66 – 6.61 (m, 2H), 6.21 – 6.12 (m, 1H), 5.48 – 5.42 (m, 1H), 5.34 – 5.27 (m, 1H), 5.23 – 5.20 (m, 1H), 2.49 (s, 3H); ^{13}C NMR (101 MHz, $CDCl_3$) δ 151.5, 148.3, 142.3, 139.2, 137.2, 135.0, 130.8, 128.9, 124.6, 121.5, 121.2, 119.7, 115.4, 110.1, 104.6, 67.2, 21.4; HR-MS [ESI-MS(+)] calcd for $[M + H – H_2O]^+$: $C_{17}H_{15}N_2$: 247.1230, found: 247.1223; IR (KBr): 3652, 3017, 2919, 1593, 1578, 1474, 1439, 1371, 1340, 1300, 1215, 1174, 1129, 1021, 989, 875, 779, 744 cm^{-1}.

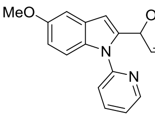
1g

1-(5-Methoxy-1-(pyridin-2-yl)-1H-indol-2-yl)prop-2-en-1-ol (1g): yellow oil; 196.0 mg, 70% yield; ^{1}H NMR (400 MHz, $CDCl_3$) δ 8.58 – 8.53 (m, 1H), 7.98 – 7.92 (m, 1H), 7.64 (d, *J* = 8.2 Hz, 1H), 7.42 (d, *J* = 9.0 Hz, 1H), 7.33 – 7.27 (m, 1H), 7.11 (d, *J* = 2.5 Hz, 1H), 6.88 (dd, *J* = 9.0, 2.5 Hz, 1H), 6.60 (d, *J* = 8.3 Hz, 2H), 6.18 – 6.10 (m, 1H), 5.45 – 5.40 (m, 1H), 5.27 (s, 1H), 5.22 – 5.18 (dt, *J* = 10.6, 1.7 Hz, 1H), 3.88 (s, 3H); ^{13}C NMR (101 MHz, $CDCl_3$) δ 155.2, 151.4, 148.4, 142.8, 139.2, 137.0, 131.7, 129.3, 121.5, 119.5, 115.4, 112.9, 111.2, 104.7, 103.2, 67.2, 55.8; HR-MS [ESI-MS(+)] calcd for $[M + H – H_2O]^+$: $C_{17}H_{15}N_2O$: 263.1179, found: 263.1172; IR (KBr): 3745, 3071, 2921, 2850, 1583, 1475, 1450, 1377, 1203, 1172, 1120, 1132, 781 cm^{-1}.

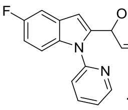
1h

1-(5-Fluoro-1-(pyridin-2-yl)-1H-indol-2-yl)prop-2-en-1-ol (1h): light yellow oil; 200.0 mg, 75% yield; ^{1}H NMR (400 MHz, $CDCl_3$) δ 8.59 – 8.57 (m, 1H), 8.01 – 7.94 (m, 1H), 7.63 (d, *J* = 8.1 Hz, 1H), 7.43 (dd, *J* = 9.0, 4.3 Hz, 1H), 7.37 – 7.34 (m, 1H), 7.29 (dd, *J* = 9.2, 2.4 Hz, 1H), 7.00 – 6.95 (m, 1H), 6.64 (s, 1H), 6.42 (d, *J* = 5.2 Hz, 1H), 6.17 – 6.09 (m, 1H), 5.46 – 5.41 (m, 1H), 5.26 (dd, *J* = 5.8, 4.0 Hz, 1H), 5.23 – 5.20 (m, 1H); ^{13}C NMR (101 MHz, $CDCl_3$) δ 158.6 (d, *J* = 237.4 Hz, $^{1}J_{CF}$), 151.1, 148.6, 143.7, 139.4, 136.7, 133.2, 129.2 (d, *J* = 10.1 Hz, $^{3}J_{CF}$), 121.9, 119.7, 115.7, 111.3 (d, *J* = 15.2 Hz, $^{2}J_{CF}$), 111.1, 106.4 (d, *J* = 23.2 Hz, $^{2}J_{CF}$), 104.6 (d, *J* = 4.0 Hz, $^{3}J_{CF}$), 67.1; HR-MS [ESI-MS(+)] calcd for $[M + Na]^+$: $C_{16}H_{13}FN_2ONa$: 291.0904, found: 291.0909; IR (KBr): 3732, 2922, 1582, 1472, 1488, 1373, 1173, 1106, 986, 927, 853, 771 cm^{-1}.

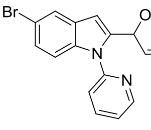
1i

1-(5-Bromo-1-(pyridin-2-yl)-1H-indol-2-yl)prop-2-en-1-ol (1i): yellow solid; m.p. 110.5 - 112.5 ^{o}C; 170.0 mg, 52% yield; ^{1}H NMR (400 MHz, $CDCl_3$) δ 8.58 (d, *J* = 4.3 Hz, 1H), 8.00 – 7.96 (m, 1H), 7.76 (d, *J* = 1.7 Hz, 1H), 7.60 (d, *J* = 8.1 Hz, 1H), 7.38 – 7.35 (m, 2H), 7.32 – 7.28 (m, 1H), 6.61 (s, 1H), 6.36 (d, *J* = 5.2 Hz, 1H), 6.16 – 6.08 (m, 1H), 5.46 – 5.41 (m, 1H), 5.26 (t, *J* = 4.9 Hz, 1H), 5.23 – 5.20 (m, 1H); ^{13}C NMR (101 MHz, $CDCl_3$) δ 150.9, 148.6, 143.5, 139.4, 136.7, 135.4, 130.3 125.9, 123.9, 122.2, 119.8, 115.8, 114.4, 111.8, 104.0, 67.0; HR-MS [ESI-MS(+)] calcd for $[M + H – H_2O]^+$:

$C_{16}H_{12}BrN_2$: 311.0178, found: 311.0168; IR (KBr): 3740, 3064, 1573, 1555, 1400, 1367, 1338, 1288, 1201, 1142, 984, 922, 865, 768 cm^{-1}.

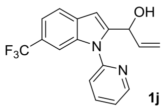
1j

1-(1-(Pyridin-2-yl)-6-(trifluoromethyl)-1H-indol-2-yl)prop-2-en-1-ol (1j): brown oil; 216.0 mg, 68% yield; ^{1}H NMR (400 MHz, $CDCl_3$) δ 8.64 – 8.59 (m, 1H), 8.05 – 8.02 (m, 1H), 7.74 (d, *J* = 11.1 Hz, 2H), 7.64 (d, *J* = 8.1 Hz, 1H), 7.47 – 7.39 (m, 2H), 6.74 (s, 1H), 6.29 (d, *J* = 5.3 Hz, 1H), 6.17 – 6.09 (m, 1H), 5.47 – 5.42 (m, 1H), 5.30 (t, *J* = 4.9 Hz, 1H), 5.26 – 5.19 (m, 1H); ^{13}C NMR (101 MHz, $CDCl_3$) δ 150.6, 148.8, 145.0, 139.7, 136.5, 135.8, 131.0, 125.1 (d, *J* = 32.3 Hz, $^2J_{CF}$), 125.0 (d, *J* = 272.7 Hz, $^1J_{CF}$), 122.5, 121.8, 120.0, 118.1 (q, *J* = 4.0 Hz, $^3J_{CF}$), 115.9, 107.8 (q, *J* = 4.0 Hz, $^3J_{CF}$), 104.5, 67.0; HR-MS [ESI-MS(+)] calcd for $[M + Na]^+$: $C_{17}H_{13}F_3N_2ONa$: 341.0872, found: 341.0875; IR (KBr): 3317, 3069, 2922, 2852, 1592, 1474, 1439, 1347, 1323, 1163, 1115, 1050, 992, 968, 870, 828, 786 cm^{-1}.

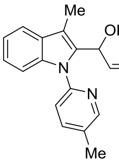
1k

1-(3-Methyl-1-(5-methylpyridin-2-yl)-1H-indol-2-yl)prop-2-en-1-ol (1k): light yellow solid; m.p. 77.4 - 78.6 °C; 222.0 mg, 80% yield; ^{1}H NMR (400 MHz, $CDCl_3$) δ 8.34 (s, 1H), 7.73 – 7.71 (m, 1H), 7.67 – 7.60 (m, 1H), 7.50 – 7.43 (m, 2H), 7.29 – 7.21 (m, 2H), 6.88 (d, *J* = 10.0 Hz, 1H), 5.84 – 5.76 (m, 1H), 5.58 (d, *J* = 7.4 Hz, 1H), 5.05 (d, *J* = 17.3 Hz, 1H), 4.82 (d, *J* = 10.5 Hz, 1H), 2.46 (s, 3H), 2.43 (s, 3H); ^{13}C NMR (101 MHz, $CDCl_3$) δ 149.7, 148.0, 139.7, 139.0, 136.9, 136.1, 131.3, 129.6, 123.2, 120.7, 119.6, 119.4, 113.9, 112.4, 110.0, 66.2, 18.0, 9.1; HR-MS [ESI-MS(+)] calcd for $[M + H – H_2O]^+$: $C_{18}H_{17}N_2$: 261.1386, found: 261.1383; IR (KBr): 3730, 3624, 2920, 1731, 1715, 1599, 1573, 1486, 1457, 1396, 1360, 1318, 1226, 1109, 1033, 987, 923, 829, 740 cm^{-1}.

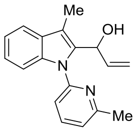
1l

1-(3-Methyl-1-(6-methylpyridin-2-yl)-1H-indol-2-yl)prop-2-en-1-ol (1l): light yellow solid; m.p. 90.4 - 91.8 °C; 214.0 mg, 77% yield; ^{1}H NMR (500 MHz, $CDCl_3$) δ 7.76 (t, *J* = 7.8 Hz, 1H), 7.61 – 7.60 (m, 1H), 7.48 – 7.43 (m, 1H), 7.35 (d, *J* = 8.0 Hz, 1H), 7.23 – 7.17 (m, 2H), 7.09 (d, *J* = 7.6 Hz, 1H), 7.03 (d, *J* = 9.9 Hz, 1H), 5.80 – 5.73 (m, 1H), 5.56 (d, *J* = 7.6 Hz, 1H), 5.01 (d, *J* = 17.3 Hz, 1H), 4.78 (d, *J* = 10.5 Hz, 1H), 2.59 (s, 3H), 2.43 (s, 3H); ^{13}C NMR (126 MHz, $CDCl_3$) δ 157.5, 151.3, 139.2, 139.2, 137.0, 136.1, 129.7, 123.2, 120.9, 120.8, 119.6, 116.7, 113.7, 112.7, 110.1, 66.1, 23.9, 9.1; HR-MS [ESI-MS(+)] calcd for $[M + H – H_2O]^+$: $C_{18}H_{17}N_2$: 261.1386, found: 261.1378; IR (KBr): 3647, 2918, 1573, 1541, 1455, 1359, 1223, 1152, 1109, 984, 919, 737 cm^{-1}.

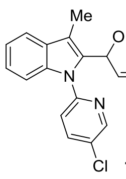
1m

1-(1-(5-Chloropyridin-2-yl)-3-methyl-1H-indol-2-yl)prop-2-en-1-ol (1m): light yellow solid; m.p. 133.1 - 134.3 °C; 238.0 mg, 80% yield; ^{1}H NMR (500 MHz, $CDCl_3$) δ 8.45 (d, *J* = 2.5 Hz, 1H), 7.86 - 7.84 (m, 1H), 7.62 – 7.61 (m, 1H), 7.52 (d, *J* = 8.6 Hz, 1H), 7.41 – 7.39 (m, 1H), 7.24 – 7.20 (m, 2H), 6.15 (d, *J* = 10.3 Hz, 1H), 5.82 – 5.76 (m, 1H), 5.58 – 5.54 (m, 1H), 5.07 – 4.99 (m, 1H), 4.87 – 4.79 (m, 1H), 2.42 (s, 3H); ^{13}C NMR (126 MHz, $CDCl_3$) δ 150.3, 146.8, 138.9, 138.9, 136.6, 136.0, 129.8, 129.2, 123.6, 121.2, 120.6, 119.8, 114.2, 113.5, 109.8, 66.0, 9.1; HR-MS [ESI-MS(+)] calcd for $[M + H – H_2O]^+$: $C_{17}H_{14}ClN_2$: 281.0840, found: 281.0835; IR (KBr): 3671, 3646, 3564, 3054, 2919, 1681, 1541, 1488, 1470, 1417, 1395, 1116, 986, 924, 743 cm^{-1}.

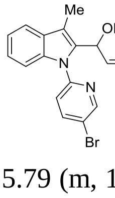
1n

1-(1-(5-Bromopyridin-2-yl)-3-methyl-1H-indol-2-yl)prop-2-en-1-ol (1n): white solid; m.p. 107.4 - 112.7 °C; 140.0 mg, 41% yield; ^{1}H NMR (400 MHz, $CDCl_3$) δ 8.57 (d, *J* = 2.1 Hz, 1H), 8.02 – 7.99 (m, 1H), 7.66 – 7.64 (m, 1H), 7.49 (d, *J* = 8.6 Hz, 1H), 7.44 – 7.43 (m, 1H), 7.29 – 7.24 (m, 2H), 6.19 (d, *J* = 10.1 Hz, 1H), 5.87 – 5.79 (m, 1H), 5.60 (d, *J* = 7.3 Hz, 1H), 5.07 (d, *J* = 17.3 Hz, 1H), 4.88 (d, *J* = 10.5 Hz, 1H), 2.46 (s, 3H); ^{13}C NMR (101 MHz, $CDCl_3$) δ 150.7, 149.1, 141.7, 138.8, 136.6, 135.9, 129.8, 123.7,

121.3, 121.1, 119.8, 117.1, 114.3, 113.6, 109.8, 66.1, 9.2; HR-MS [ESI-MS(+)] calcd for $[M + H – H_2O]^+$: $C_{17}H_{14}BrN_2$: 325.0335, found: 325.0327; IR (KBr): 3445, 2920, 1648, 1547, 1467, 1386, 1316, 1220, 1099, 1011, 924, 740 cm^{-1}.

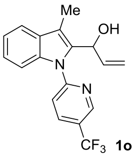
1o

1-(3-Methyl-1-(5-(trifluoromethyl)pyridin-2-yl)-1H-indol-2-yl)prop-2-en-1-ol (1o): light yellow solid; m.p. 137.0 – 139.0 °C; 133.0 mg, 40% yield; ^{1}H NMR (400 MHz, $CDCl_3$) δ 8.79 – 8.78 (m, 1H), 8.15 – 8.12 (m, 1H), 7.73 (d, *J* = 8.5 Hz, 1H), 7.68 – 7.65 (m, 1H), 7.54 – 7.48 (m, 1H), 7.32 – 7.27 (m, 2H), 6.30 (d, *J* = 10.3 Hz, 1H), 5.88 – 5.80 (m, 1H), 5.66 – 5.61 m, 1H), 5.09 – 5.03 (m, 1H), 4.88 – 4.85 (m, 1H), 2.47 (s, 3H); ^{13}C NMR (101 MHz, $CDCl_3$) δ 154.6, 145.4 (q, *J* = 4.0 Hz, $^3J_{CF}$), 138.9, 136.7, 136.4 (q, *J* = 3.0 Hz, $^3J_{CF}$), 135.8, 130.2, 124.0 (d, *J* = 34.3 Hz, $^2J_{CF}$), 123.9, 123.2 (d, *J* = 272.7 Hz, $^1J_{CF}$), 121.8, 119.9, 119.4, 114.7, 114.3, 109.9, 66.0, 9.2; HR-MS [ESI-MS(+)] calcd for $[M + H – H_2O]^+$: $C_{18}H_{14}F_3N_2$: 315.1098 , found: 315.1093; IR (KBr): 3646, 3615, 2923, 1647, 1541, 1488, 1455, 1327, 1164, 1128, 747 cm^{-1}.

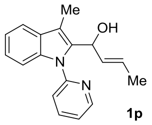
1p

(*E*)-1-(3-Methyl-1-(pyridin-2-yl)-1H-indol-2-yl)but-2-en-1-ol (1p): light yellow oil; 150.0 mg, 54% yield; ^{1}H NMR (500 MHz, $CDCl_3$) δ 8.56 – 8.53 (m, 1H), 7.95 – 7.92 (m, 1H), 7.63 – 7.59 (m, 2H), 7.47 – 7.45 (m, 1H), 7.31 – 7.28 (m, 1H), 7.23 – 7.18 (m, 2H), 6.76 (d, *J* = 10.3 Hz, 1H), 5.80 – 5.77 (m, 1H), 5.27 – 5.17 (m, 2H), 2.47 (s, 3H), 1.50 (d, *J* = 6.2 Hz, 3H); ^{13}C NMR (126 MHz, $CDCl_3$) δ 152.2, 148.3, 139.2, 138.0, 135.9, 131.9, 129.8, 125.3, 123.2, 121.5, 120.9, 119.9, 119.6, 112.1, 109.9, 62.4, 13.0, 9.1; HR-MS [ESI-MS(+)] calcd for $[M + H – H_2O]^+$: $C_{18}H_{17}N_2$: 261.1386, found: 261.1383; IR (KBr): 3699, 3631, 2923, 1852, 1585, 1438, 1352, 1216, 970, 740 cm^{-1}.

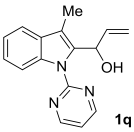
1q

1-(3-Methyl-1-(pyrimidin-2-yl)-1H-indol-2-yl)prop-2-en-1-ol (1q): light yellow solid; m.p. 94.4 - 96.7 °C; 123.0 mg, 46% yield; ^{1}H NMR (500 MHz, $CDCl_3$) δ 8.73 (d, *J* = 4.8 Hz, 2H), 8.38 (d, *J* = 8.3 Hz, 1H), 7.58 (d, *J* = 7.7 Hz, 1H), 7.33 – 7.23 (m, 2H), 7.11 (t, *J* = 4.8 Hz, 1H), 6.95 (d, *J* = 10.8 Hz, 1H), 5.97 – 5.91 (m, 1H), 5.68 – 5.65 (m, 1H), 5.03 (d, *J* = 17.4 Hz, 1H), 4.87 (d, *J* = 10.6 Hz, 1H), 2.42 (s, 3H); ^{13}C NMR (126 MHz, $CDCl_3$) δ 157.8, 157.7, 139.9, 136.5, 135.7, 130.4, 124.4, 122.2, 119.2, 116.7, 116.6, 114.4, 114.2, 66.1, 9.4; HR-MS [ESI-MS(+)] calcd for $[M + H – H_2O]^+$: $C_{16}H_{14}N_3$: 248.1182, found: 248.1171; IR (KBr): 3671, 3647, 2921, 1563, 1541, 1488, 1455, 1429, 1339, 1010, 744 cm^{-1}.

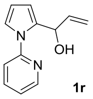
1r

1-(1-(Pyridin-2-yl)-1H-pyrrol-2-yl)prop-2-en-1-ol (1r): brown oil; 55.0 mg, 28% yield; ^{1}H NMR (500 MHz, $CDCl_3$) δ 8.43 (d, *J* = 4.7 Hz, 1H), 7.89 – 7.78 (m, 1H), 7.37 (d, *J* = 8.2 Hz, 1H), 7.24 – 7.22 (m, 1H), 7.05 (s, 1H), 6.67 (d, *J* = 5.1 Hz, 1H), 6.29 – 6.27 (m, 2H), 6.15 – 6.09 (m, 1H), 5.39 (d, *J* = 17.2 Hz, 1H), 5.23 (s, 1H), 5.16 (d, *J* = 10.5 Hz, 1H). ^{13}C NMR (126 MHz, $CDCl_3$) δ 152.4, 147.5, 139.6, 137.7, 135.9, 121.4, 121.1, 116.3, 115.0, 111.5, 110.3, 66.7; HR-MS [ESI-MS(+)] calcd for $[M + H – H_2O]^+$: $C_{12}H_{11}N_2$: 183.0917, found: 183.0916; IR (KBr): 3647, 2924, 2855, 1867, 1714, 1681, 1647, 1472, 1440, 1417, 1338, 1154, 993 cm^{-1}.

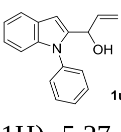
1u

1-(1-Phenyl-1H-indol-2-yl)prop-2-en-1-ol (1u): yellow soild; m.p. 95.7 – 99.3 °C; 154.4 mg, 62% yield; ^{1}H NMR (400 MHz, $CDCl_3$) δ 7.68 – 7.66 (m, 1H), 7.59 – 7.48 (m, 5H), 7.20 – 7.13 (m, 3H), 6.68 (s, 1H), 6.23 – 6.14 (m, 1H), 5.35 – 5.31 (m, 1H), 5.27 – 5.24 (m, 1H), 5.24 – 5.20 (m, 1H), 1.85 (d, *J* = 6.0 Hz, 1H). ^{13}C NMR (101 MHz, $CDCl_3$) δ 141.5, 138.7, 138.1, 137.5, 129.5, 128.5, 128.2, 127.4, 122.5, 120.8, 120.4, 115.7, 110.6,

101.3, 67.5; HR-MS [ESI-MS(+)] calcd for $[M + H – H_2O]^+$: $C_{17}H_{14}N_2$: 232.1121, found: 232.1118; IR (KBr): 3057, 2921, 1595, 1498, 1454, 1338, 1217, 1017, 988, 929, 750, 698 cm^{-1}.

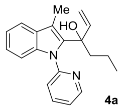
4a

3-(3-Methyl-1-(pyridin-2-yl)-1H-indol-2-yl)hex-1-en-3-ol (4a): yellow solid; m.p. 58.7 - 60.3 °C; 202.0 mg, 66% yield; ^1H NMR (400 MHz, $CDCl_3$) δ 8.54 – 8.52 (m, 1H), 7.94 – 7.89 (m, 1H), 7.67 – 7.63 (m, 1H), 7.50 (d, *J* = 8.0 Hz, 1H), 7.35 – 7.31 (m, 1H), 7.23 – 7.17 (m, 3H), 6.66 (s, 1H), 6.02 – 5.95 (m, 1H), 5.09 – 5.04 (m, 1H), 4.82 – 4.79 (m, 1H), 2.56 (s, 3H), 2.11 – 2.02 (m, 1H), 1.93 – 1.86 (m, 1H), 1.50 – 1.40 (m, 1H), 1.36 – 1.25 (m, 1H), 0.89 (t, *J* = 7.4 Hz, 3H); ^{13}C NMR (101 MHz, $CDCl_3$) δ 153.5, 148.1, 142.9, 139.1, 138.9, 136.9, 130.2, 123.1, 122.3, 122.3, 120.6, 119.2, 112.2, 111.3 109.6, 75.2, 43.5, 17.3, 14.5, 11.3; HR-MS [ESI-MS(+)] calcd for $[M + H – H_2O]^+$: $C_{20}H_{21}N_2$: 289.1699, found: 289.1684; IR (KBr): 3341, 2959, 2928, 2870, 1591, 1473, 1458, 1437, 1357, 1311, 1205, 1150, 1002, 921, 777, 741 cm^{-1}.

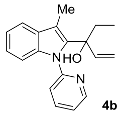
4b

3-(3-Methyl-1-(pyridin-2-yl)-1H-indol-2-yl)pent-1-en-3-ol (4b): yellow solid; m.p. 64.0 - 65.2 °C; 102.0 mg, 35% yield; ^1H NMR (500 MHz, $CDCl_3$) δ 8.49 (d, *J* = 4.4 Hz, 1H), 7.87 (t, *J* = 7.7 Hz, 1H), 7.64 – 7.59 (m, 1H), 7.47 (d, *J* = 8.0 Hz, 1H), 7.32 – 7.27 (m, 1H), 7.21 – 7.14 (m, 3H), 6.68 (s, 1H), 5.92 – 5.87 (m, 1H), 5.01 (d, *J* = 17.3 Hz, 1H), 4.78 (d, *J* = 10.7 Hz, 1H), 2.54 (s, 3H), 2.18 – 2.11 (m, 1H), 1.99 – 1.92 (m 1H), 0.89 (t, *J* = 7.3 Hz, 3H); ^{13}C NMR (126 MHz, $CDCl_3$) δ 153.5, 148.0, 142.4, 139.1, 139.0, 136.9, 130.3, 123.1, 122.3, 122.3, 120.6, 119.2, 112.6, 111.2, 109.6, 75.5, 33.5, 11.3, 8.3; HR-MS [ESI-MS(+)] calcd for $[M + H – H_2O]^+$: $C_{19}H_{19}N_2$: 275.1543, found: 275.1536; IR (KBr): 3671, 3646, 3055, 2921, 2855, 1589, 1556, 1472, 1455, 1434, 1351, 1143, 963, 919, 738 cm^{-1}.

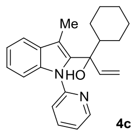
4c

1-Cyclohexyl-1-(3-methyl-1-(pyridin-2-yl)-1H-indol-2-yl)prop-2-en-1-ol (4c): yellow oil; 243.0 mg, 70% yield; ^1H NMR (500 MHz, $CDCl_3$) δ 8.55 – 8.50 (m, 1H), 7.94 – 7.91 (m, 1H), 7.61 – 7.56 (m, 1H), 7.52 (d, *J* = 8.0 Hz, 1H), 7.34 – 7.32 (m, 1H), 7.18 – 7.12 (m, 3H), 6.19 – 6.13 (m, 1H), 5.92 (s, 1H), 5.38 – 5.35 (m, 1H), 5.12 – 5.09 (m, 1H), 2.49 (s, 3H), 1.76 (d, *J* = 10.6 Hz, 1H), 1.69 – 1.65 (m, 1H), 1.59 (s, 1H), 1.52 (d, *J* = 11.5 Hz, 2H), 1.41 (d, *J* = 11.0 Hz, 1H), 1.02 – 0.87 (m, 5H); ^{13}C NMR (126 MHz, $CDCl_3$) δ 154.0, 148.5, 139.5, 139.0, 138.8, 137.1, 130.2, 122.9, 122.4, 122.3, 120.5, 119.0, 113.6, 111.9, 109.6, 77.7, 46.3, 28.7, 26.9, 26.7, 26.6, 26.6, 11.5; HR-MS [ESI-MS(+)] calcd for $[M + H – H_2O]^+$: $C_{23}H_{25}N_2$: 329.2012, found: 329.2005; IR (KBr): 3699, 3632, 2926, 2853, 1654, 1588, 1467, 1437, 1351, 1301, 1210, 930, 741 cm^{-1}.

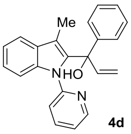
4d

1-(3-Methyl-1-(pyridin-2-yl)-1H-indol-2-yl)-1-phenylprop-2-en-1-ol (4d): white solid; m.p. 138.2 - 139.5 °C; 100.0 mg, 30% yield; ^1H NMR (500 MHz, $CDCl_3$) δ 8.34 (d, *J* = 4.5 Hz, 1H), 7.77 (s, 1H), 7.65 (d, *J* = 7.2 Hz, 1H), 7.52 (t, *J* = 7.6 Hz, 1H), 7.21 – 7.13 (m, 3H), 7.10 (d, *J* = 7.5 Hz, 2H), 7.05 – 7.01 (m, 1H), 7.00 – 6.89 (m, 4H), 6.43 – 6.38 (m, 1H), 5.44 (d, *J* = 17.0 Hz, 1H), 5.09 (d, *J* = 10.5 Hz, 1H), 2.47 (s, 3H); ^{13}C NMR (126 MHz, $CDCl_3$) δ 152.5, 147.7, 146.3, 143.4, 138.8, 138.6, 136.5, 130.0, 127.5, 126.1, 125.6, 123.4, 122.1, 121.6, 120.7, 119.5, 113.5, 111.6, 109.5, 76.4, 11.1; HR-MS [ESI-MS(+)] calcd for $[M + H – H_2O]^+$: $C_{23}H_{19}N_2$: 323.1543, found: 323.1535; IR (KBr): 3632, 3597, 2928, 1654, 1617, 1577, 1560, 1438, 1122, 744 cm^{-1}.

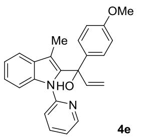
4e

1-(4-Methoxyphenyl)-1-(3-methyl-1-(pyridin-2-yl)-1H-indol-2-yl)prop-2-en-1-ol (4e): yellow oil; 110.0 mg, 30% yield; ^1H NMR (400 MHz, $CDCl_3$) δ 8.39 – 8.38 (m, 1H), 7.78 (d, *J* = 1.0 Hz, 1H), 7.69 – 7.59 (m, 2H), 7.23 – 7.19 (m, 3H), 7.11 – 7.03 (m, 4H), 6.56 (d, *J* = 8.8 Hz, 2H), 6.44 – 6.41 (m, 1H), 5.42 – 5.37 (m, 1H), 5.10 –

5.07 (m, 1H), 3.70 (s, 3H), 2.46 (s, 3H); ^{13}C NMR (101 MHz, $CDCl_3$) δ 157.9, 152.6, 147.6, 143.6, 139.0, 138.6, 138.4, 136.6, 130.0, 126.9, 123.4, 122.0, 121.6, 120.6, 119.4, 113.3, 113.0, 111.3, 109.5, 76.0, 55.2, 11.0; HR-MS [ESI-MS(+)] calcd for $[M + H - H_2O]^+$: $C_{24}H_{21}N_2O$: 353.1648, found: 353.1646; IR (KBr): 3239, 3056, 2925, 2834, 1591, 1506, 1473, 1457, 1438, 1359, 1300, 1246, 1169, 1034, 921, 833, 774, 741 cm^{-1}.

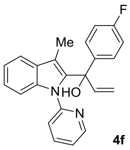

4f

1-(4-Fluorophenyl)-1-(3-methyl-1-(pyridin-2-yl)-1H-indol-2-yl)prop-2-en-1-ol (4f): yellow solid; m.p. 123.7 - 125.8 °C; 133.0 mg, 37% yield; ^{1}H NMR (400 MHz, $CDCl_3$) δ 8.40 – 8.36 (m, 1H), 7.86 (d, J = 1.2 Hz, 1H), 7.71 – 7.60 (m, 2H), 7.26 – 7.20 (m, 3H), 7.14 – 7.04 (m, 4H), 6.74 – 6.66 (m, 2H), 6.45 – 6.38 (m, 1H), 5.46 – 5.41 (m, 1H), 5.15 – 5.12 (m, 1H), 2.50 (s, 3H); ^{13}C NMR (101 MHz, $CDCl_3$) δ 161.2 (d, J = 246.4 Hz, $^1J_{CF}$), 152.4, 147.7, 143.3, 142.1 (d, J = 3.0 Hz, $^3J_{CF}$), 138.7, 138.5, 136.5, 130.0, 127.4 (d, J = 8.1 Hz, $^3J_{CF}$), 123.6, 121.9, 121.7, 120.8, 119.5, 114.3 (d, J = 21.2 Hz, $^2J_{CF}$), 113.6, 111.9, 109.6, 76.0, 11.1; HR-MS [ESI-MS(+)] calcd for $[M + H - H_2O]^+$: $C_{23}H_{18}FN_2$: 341.1449, found: 341.1444; IR (KBr): 3661, 3632, 2925, 1734, 1685, 1654, 1629, 1437, 1351, 1217, 1148, 905, 840, 741 cm^{-1}.

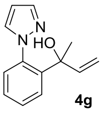

4g

2-(2-(1H-Pyrazol-1-yl)phenyl)but-3-en-2-ol (4g): yellow oil; 164 mg, 77% yield; ^{1}H NMR (400 MHz, $CDCl_3$) δ 7.75 – 7.66 (m, 2H), 7.59 (d, J = 2.0 Hz, 1H), 7.47 – 7.37 (m, 2H), 7.28 – 7.25 (m, 1H), 6.81 (s, 1H), 6.43 (t, J = 2.1 Hz, 1H), 5.75 (dd, J = 17.3, 10.7 Hz, 1H), 4.88 (dd, J = 17.3, 1.0 Hz, 1H), 4.61 (dd, J = 10.7, 1.1 Hz, 1H), 1.59 (s, 3H); ^{13}C NMR (101 MHz, $CDCl_3$) δ 143.7, 141.5, 139.7, 139.40, 131.3, 128.7, 128.4, 127.8, 127.2, 110.1, 107.3, 73.2, 28.3; HR-MS [ESI-MS(+)] calcd for $[M + H - H_2O]^+$: $C_{13}H_{13}N_2$: 197.1073, found: 197.1075; IR (KBr): 2982, 2929, 1518, 1493, 1451, 1396, 1325, 1195, 1107, 1053, 925, 761, cm^{-1}.

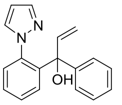

4h

1-(2-(1H-Pyrazol-1-yl)phenyl)-1-phenylprop-2-en-1-ol (4h): yellow oil; 180.0 mg, 65% yield; ^{1}H NMR (500 MHz, $CDCl_3$) δ 7.71 – 7.67 (m, 1H), 7.48 (d, J = 1.5 Hz, 1H), 7.44 – 7.37 (m, 2H), 7.33 (s, 1H), 7.21 – 7.17 (m, 1H), 7.08 – 6.98 (m, 6H), 6.20 (dd, J = 17.0, 10.6 Hz, 1H), 6.02 (t, J = 1.9 Hz, 1H), 5.36 (d, J = 17.0 Hz, 1H), 5.13 (d, J = 10.6 Hz, 1H); ^{13}C NMR (126 MHz, $CDCl_3$) δ 145.6, 142.8, 142.6, 139.6, 139.4, 130.9, 130.2, 128.6, 128.5, 127.5, 127.4, 126.1, 125.0, 112.7, 107.0, 78.0; HR-MS [ESI-MS(+)] calcd for $[M + H - H_2O]^+$: $C_{18}H_{15}N_2$: 259.1230, found: 259.1234; IR (KBr): 3061, 3022, 1518, 1490, 1451, 1396, 1326, 1194, 1051, 944, 922, 763, 703 cm^{-1}.

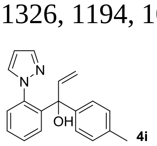

4i

1-(2-(1H-Pyrazol-1-yl)phenyl)-1-(p-tolyl)prop-2-en-1-ol (4i): white solid; m.p. 103.0 – 106.2 °C; 200 mg, 69% yield; ^{1}H NMR (500 MHz, $CDCl_3$) δ 7.64 – 7.61 (m, 1H), 7.47 (s, 1H), 7.40 – 7.34 (m, 2H), 7.29 (s, 1H), 7.18 (d, J = 7.8 Hz, 1H), 7.10 (d, J = 2.2 Hz, 1H), 6.97 (d, J = 8.0 Hz, 2H), 6.87 (d, J = 8.0 Hz, 2H), 6.17 (dd, J = 17.0, 10.6 Hz, 1H), 6.04 (s, 1H), 5.31 (d, J = 17.0 Hz, 1H), 5.07 (d, J = 10.6 Hz, 1H), 2.20 (s, 3H); ^{13}C NMR (126 MHz, $CDCl_3$) δ 143.0, 142.7, 142.6, 139.6, 139.4, 135.6, 131.0, 130.2, 128.6, 128.5, 128.2, 127.4, 125.1, 112.3, 107.0, 77.9, 21.0; HR-MS [ESI-MS(+)] calcd for $[M + H - H_2O]^+$: $C_{19}H_{17}N_2$: 273.1386, found: 273.1399; IR (KBr): 3253, 3104, 2920, 1508, 1427, 1397, 1326, 1185, 1104, 1052, 994, 922, 818, 761 cm^{-1}.

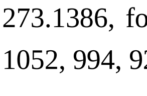

4j

1-(2-(1H-Pyrazol-1-yl)phenyl)-1-(4-methoxyphenyl)prop-2-en-1-ol (4j): white solid; m.p. 147.8 – 149.6 °C; 202 mg, 66% yield; ^{1}H NMR (400 MHz, $CDCl_3$) δ 7.68 – 7.64 (m, 1H), 7.54 (d, J = 1.6 Hz, 1H), 7.46 – 7.40 (m, 2H), 7.36 (d, J = 1.1 Hz, 1H), 7.25 – 7.21 (m, 1H), 7.16 (d, J = 2.2 Hz, 1H), 7.07 – 7.03 (m, 2H), 6.68 – 6.63 (m, 2H), 6.25 – 6.17 (m, 1H), 6.11 (t, J = 2.2 Hz, 1H), 5.31 (dd, J = 17.1, 1.5 Hz, 1H), 5.11 (dd, J = 10.5,

1.5 Hz, 1H), 3.74 (s, 3H); ^{13}C NMR (101 MHz, $CDCl_3$) δ 157.9, 143.0, 142.7, 139.6, 139.4, 137.7, 131.0, 130.3, 128.6, 128.5, 127.4, 126.3, 113.0, 112.3, 107.1, 77.7, 55.2; HR-MS [ESI-MS(+)] calcd for $[M + H – H_2O]^+$: $C_{19}H_{17}N_2O$: 289.1335, found: 289.1340; IR (KBr): 3242, 3114, 2842, 1607, 1508, 1453, 1395, 1295, 1249, 1172, 1108, 1052, 1028, 997, 944, 922, 832, 769, 673 cm^{-1}.

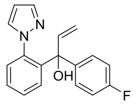
4k

1-(2-(1H-Pyrazol-1-yl)phenyl)-1-(4-fluorophenyl)prop-2-en-1-ol (4k): white solid; m.p. 134.3 – 139.0 °C; 220.0 mg, 75% yield; ^{1}H NMR (500 MHz, $CDCl_3$) δ 7.70 – 7.65 (m, 1H), 7.49 (s, 1H), 7.44 – 7.38 (m, 2H), 7.21 (d, J = 7.2 Hz, 1H), 7.10 (d, J = 2.1 Hz, 1H), 7.06 – 7.03 (m, 2H), 6.74 (t, J = 8.6 Hz, 2H), 6.20 – 6.07 (m, 1H), 6.07 (s, 1H), 5.33 (d, J = 17.0 Hz, 1H), 5.14 (d, J = 10.6 Hz, 1H); ^{13}C NMR (126 MHz, $CDCl_3$) δ 161.3 (d, J = 244.4 Hz, $^1J_{CF}$), 142.7, 142.3, 141.5 (d, J = 3.8 Hz, $^3J_{CF}$), 139.6, 139.3, 130.9, 130.1, 128.7 (d, J =23.9 Hz, $^2J_{CF}$), 127.4, 126.7, 126.6, 114.2 (d, J = 21.4 Hz, $^2J_{CF}$), 112.9, 107.2, 77.7; HR-MS [ESI-MS(+)] calcd for $[M + H – H_2O]^+$: $C_{18}H_{14}FN_2$: 277.1136, found: 277.1138; IR (KBr): 3194, 3105, 1597, 1500, 1451, 1399, 1329, 1296, 1208, 1156, 1097, 1055, 994, 925, 837, 764, 714 cm^{-1}.

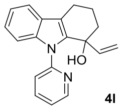
4l

9-(Pyridin-2-yl)-1-vinyl-2,3,4,9-tetrahydro-1H-carbazol-1-ol (4l): light yellow solid; m.p. 96.9 - 99.4 °C; 183.0 mg, 63% yield; ^{1}H NMR (400 MHz, $CDCl_3$) δ 8.47 – 8.47 (m, 1H), 7.92 – 7.88 (m, 1H), 7.64 – 7.57 (m, 2H), 7.51 – 7.49 (m, 1H), 7.29 – 7.21 (m, 3H), 7.09 (d, J = 1.3 Hz, 1H), 5.84 – 5.77 (m, 1H), 4.93 – 4.89 (m, 1H), 4.65 – 4.62 (m, 1H), 2.97 – 2.91 (m, 1H), 2.79 – 2.70 (m, 1H), 2.24 – 2.10 (m, 2H), 1.99 – 1.83 (m, 2H); ^{13}C NMR (101 MHz, $CDCl_3$) δ 152.0, 147.9, 144.1, 139.0, 138.3, 136.2, 128.2, 123.3, 121.3, 121.0, 119.7, 119.4, 116.2, 112.3, 110.0, 70.1, 39.6, 21.9, 19.6; HR-MS [ESI-MS(+)] calcd for $[M + H – H_2O]^+$: $C_{19}H_{17}N_2$: 273.1386, found: 273.1383; IR (KBr): 3282, 2925, 2850, 1592, 1477, 1444, 1374, 1341, 1307, 1117, 991, 968, 927, 775, 740 cm^{-1}.

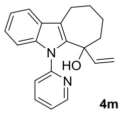
4m

5-(Pyridin-2-yl)-6-vinyl-5,6,7,8,9,10-hexahydrocyclohepta[b]indol-6-ol (4m): white solid; m.p. 78.2 - 82.5 °C; 216.0 mg, 71% yield; ^{1}H NMR (400 MHz, $CDCl_3$) δ 8.52 – 8.48 (m, 1H), 7.90 – 7.86 (m, 1H), 7.69 – 7.64 (m, 1H), 7.50 (d, J = 8.1 Hz, 1H), 7.42 – 7.33 (m, 2H), 7.30 – 7.20 (m, 3H), 5.86 – 5.79 (m, 1H), 4.75 (d, J = 17.4 Hz, 1H), 4.55 (d, J = 10.7 Hz, 1H), 3.11 – 3.05 (m, 1H), 2.96 – 2.89 (m, 1H), 2.20 – 2.13 (m, 3H), 2.10 – 2.05 (m, 1H), 2.00 – 1.91 (m, 1H), 1.81 – 1.76 (m, 1H); ^{13}C NMR (101 MHz, $CDCl_3$) δ 152.9, 147.8, 144.9, 139.8, 139.0, 136.4, 129.0, 123.0, 121.8, 121.3, 120.8, 119.2, 119.1, 111.5, 109.9, 74.6, 37.7, 25.8, 20.8, 20.5; HR-MS [ESI-MS(+)] calcd for $[M + H – H_2O]^+$: $C_{20}H_{19}N_2$: 287.1543, found: 287.1539; IR (KBr): 3725, 3630, 3052, 2929, 2857, 1590, 1569, 1473, 1456, 1439, 1355, 1310, 1222, 1150, 1127, 989, 915, 776, 740 cm^{-1}.

Method E:

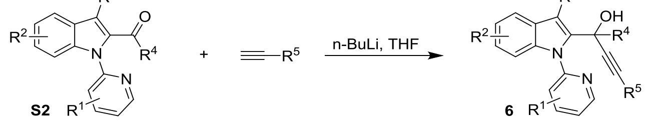

Synthetic procedure of substituted α-(2-indolyl)propargyl alcohols 6 [4]: To a solution of alkynes (2.0 mmol, 2.0 eq.) in anhydrous THF (10 mL) at –78 °C under Ar atmosphere was added *n*-BuLi (1.6 M, 1.3 mL, 2.0 eq.) dropwise. Then the reaction was stirred at room temperature for 1 h. After cooling to –78 °C, aldehydes (1.0 mmol, 1.0 eq.) in THF (5 mL) was added to the mixture dropwise. The reaction mixture was warmed up to room temperature gradually, and was stirred overnight. The reaction was quenched with aqueous NH_4Cl. The mixture was extracted with EtOAc (10 mL × 3). The combined organic phases were washed with brine and dried over

anhydrous Na_2SO_4, then concentrated under reduced pressure. The residue was purified by column chromatography to give the desired products **6**.

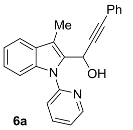
6a

1-(3-Methyl-1-(pyridin-2-yl)-1H-indol-2-yl)-3-phenylprop-2-yn-1-ol (6a): yellow oil; 253.0 mg, 75% yield; ^{1}H NMR (400 MHz, $CDCl_3$) δ 8.64 – 8.62 (m, 1H), 7.99 – 7.95 (m, 1H), 7.75 – 7.66 (m, 2H), 7.58 (d, *J* = 7.8 Hz, 1H), 7.50 (d, *J* = 11.0 Hz, 1H), 7.35 – 7.16 (m, 6H), 6.99 (d, *J* = 7.1 Hz, 2H), 6.02 (d, *J* = 11.0 Hz, 1H), 2.53 (s, 3H); ^{13}C NMR (101 MHz, $CDCl_3$) δ 152.2, 148.4, 139.2, 135.9, 135.5, 131.5, 129.5, 128.2, 128.0, 123.9, 122.6, 121.5, 121.2, 120.0, 119.7, 112.6, 110.1, 88.3, 85.1, 56.0, 9.1; HR-MS [ESI-MS(+)] calcd for $[M + H]^+$: $C_{23}H_{19}N_2O$: 339.1492, found: 339.1489; IR (KBr): 3286, 3080, 2916, 2200, 1591, 1473, 1440, 1362, 1219, 1150, 1092, 1010, 963, 775, 739 cm^{-1}.

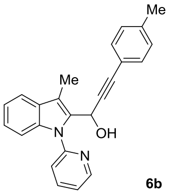
6b

1-(3-Methyl-1-(pyridin-2-yl)-1H-indol-2-yl)-3-(*p*-tolyl)prop-2-yn-1-ol (6b): yellow solid; m.p. 118.1 - 119.7 °C; 244.0 mg, 69% yield; ^{1}H NMR (400 MHz, $CDCl_3$) δ 8.64 – 8.62 (m, 1H), 7.98 – 7.94 (m, 1H), 7.76 – 7.65 (m, 2H), 7.58 (d, *J* = 7.8 Hz, 1H), 7.48 (d, *J* = 11.0 Hz, 1H), 7.34 – 7.25 (m, 3H), 7.00 (d, *J* = 8.2 Hz, 2H), 6.89 (d, *J* = 8.0 Hz, 2H), 6.02 (d, *J* = 11.0 Hz, 1H), 2.53 (s, 3H), 2.31 (s, 3H); ^{13}C NMR (101 MHz, $CDCl_3$) δ 152.2, 148.4, 139.2, 138.3, 135.9, 135.6, 131.4, 129.5, 128.8, 123.8, 121.5, 121.2, 120.0, 119.7, 119.5, 112.5, 110.1, 87.6, 85.2, 56.1, 21.4, 9.0; HR-MS [ESI-MS(+)] calcd for $[M + H – H_2O]^+$: $C_{24}H_{19}N_2$: 335.1543, found: 335.1531; IR (KBr): 3247, 3078, 2911, 2210, 1588, 1474, 1438, 1362, 1091, 1006, 962, 817, 780, 740 cm^{-1}.

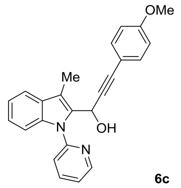
6c

3-(4-Methoxyphenyl)-1-(3-methyl-1-(pyridin-2-yl)-1H-indol-2-yl)prop-2-yn-1-ol (6c): yellow oil; 121.0 mg, 33% yield; ^{1}H NMR (400 MHz, $CDCl_3$) δ 8.63 (d, *J* = 3.6 Hz, 1H), 7.99 – 7.95 (m, 1H), 7.72 (d, *J* = 8.1 Hz, 1H), 7.66 (d, *J* = 7.4 Hz, 1H), 7.56 (d, *J* = 8.0 Hz, 1H), 7.42 (d, *J* = 11.0 Hz, 1H), 7.34 – 7.24 (m, 3H), 6.89 (d, *J* = 8.7 Hz, 2H), 6.70 (d, *J* = 8.7 Hz, 2H), 5.96 (d, *J* = 10.9 Hz, 1H), 3.77 (s, 3H), 2.50 (s, 3H); ^{13}C NMR (101 MHz, $CDCl_3$) δ 159.4, 152.2, 148.4, 139.1, 135.9, 135.6, 132.9, 129.5, 123.7, 121.4, 121.1, 119.9, 119.6, 114.6, 113.6, 112.5, 110.0, 86.8, 84.9, 56.0, 55.2, 9.0; HR-MS [ESI-MS(+)] calcd for $[M + H – H_2O]^+$: $C_{24}H_{19}N_2O$: 351.1492, found: 351.1486; IR (KBr): 3242, 3054, 2921, 2836, 2201, 1591, 1508, 1474, 1455, 1439, 1364, 1287, 1247, 1172, 1031, 966, 832, 779, 741 cm^{-1}.

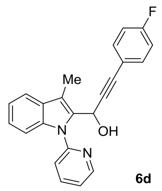
6d

3-(4-Fluorophenyl)-1-(3-methyl-1-(pyridin-2-yl)-1H-indol-2-yl)prop-2-yn-1-ol (6d): brown solid; m.p. 113.9 - 114.9 °C; 192.0 mg, 54% yield; ^{1}H NMR (500 MHz, $CDCl_3$) δ 8.59 – 8.54 (m, 1H), 7.92 – 7.89 (m, 1H), 7.66 (d, *J* = 8.1 Hz, 1H), 7.62 (d, *J* = 7.5 Hz, 1H), 7.51 (d, *J* = 8.2 Hz, 1H), 7.42 (d, *J* = 11.0 Hz, 1H), 7.27 – 7.20 (m, 3H), 6.93 – 6.86 (m, 2H), 6.82 (t, *J* = 8.7 Hz, 2H), 5.93 (d, *J* = 11.0 Hz, 1H), 2.45 (s, 3H); ^{13}C NMR (126 MHz, $CDCl_3$) δ 162.3 (d, *J* = 250.7, $^1J_{CF}$), 152.2, 148.4, 139.2, 135.9, 135.3, 133.3 (d, *J* = 7.6 Hz, $^3J_{CF}$), 129.5, 123.9, 121.4, 121.3, 120.0, 119.6, 118.6 (d, *J* = 2.5 Hz, $^3J_{CF}$), 115.3 (d, *J* = 22.7 Hz, $^2J_{CF}$), 112.7, 110.1, 88.0, 84.0, 55.9, 9.0; HR-MS [ESI-MS(+)] calcd for $[M + H]^+$: $C_{23}H_{18}FN_2O$: 357.1398, found: 357.1397; IR (KBr): 3056, 2953, 2923, 2854, 2220, 1591, 1506, 1474, 1455, 1439, 1364, 1232, 1154, 1093, 1014, 967, 835, 779, 741 cm^{-1}.

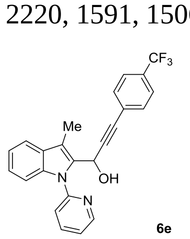
6e

1-(3-Methyl-1-(pyridin-2-yl)-1H-indol-2-yl)-3-(4-(trifluoromethyl)phenyl)prop-2-yn-1-ol (6e): yellow oil; 210.0 mg, 52% yield; ^{1}H NMR (500 MHz, $CDCl_3$) δ 8.65 – 8.57 (m, 1H), 7.96 – 7.93 (m, 1H), 7.69 (d, *J* = 8.1 Hz, 1H), 7.64 (d, *J* = 7.7 Hz, 1H), 7.54 (d, *J* = 8.2 Hz, 1H), 7.47 (d, *J* = 11.0 Hz, 1H), 7.40 (d, *J* = 8.2 Hz, 2H), 7.32 – 7.22 (m, 3H), 7.02 (d, *J* = 8.1 Hz, 2H), 5.95 (d, *J* = 10.9 Hz, 1H),

2.47 (s, 3H); ^{13}C NMR (126 MHz, $CDCl_3$) δ 152.1, 148.4, 139.2, 135.9, 134.9, 131.6, 129.9 (d, *J* = 7.6 Hz, $^3J_{CF}$),129.4, 126.3, 125.0 (q, *J* = 3.8 Hz, $^3J_{CF}$), 122.7, 124.0, 121.4, 121.3, 120.03, 119.5, 113.0, 110.1, 90.8, 83.6, 55.9, 9.0; HR-MS [ESI-MS(+)] calcd for $[M + H]^+$: $C_{24}H_{18}F_3N_2O$: 407.1366, found: 407.1370; IR (KBr): 3715, 3060, 2924, 1507, 1473, 1455, 1324, 1166, 1125, 1067, 1017, 842, 742 cm^{-1}.

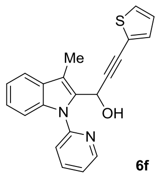
6f

1-(3-Methyl-1-(pyridin-2-yl)-1H-indol-2-yl)-3-(thiophen-2-yl)prop-2-yn-1-ol (6f): brown solid; m.p. 65.7 - 67.2 °C; 130.0 mg, 38% yield; ^{1}H NMR (400 MHz, $CDCl_3$) δ 8.62 – 8.61 (m, 1H), 8.02 – 7.98 (m, 1H), 7.72 (d, *J* = 8.1 Hz, 1H), 7.66 (d, *J* = 7.2 Hz, 1H), 7.56 (d, *J* = 7.9 Hz, 1H), 7.45 (d, *J* = 11.1 Hz, 1H), 7.37 – 7.31 (m, 1H), 7.30 – 7.24 (m, 2H), 7.13 – 7.12 (m, 1H), 6.92 – 6.75 (m, 2H), 5.97 (d, *J* = 11.0 Hz, 1H), 2.49 (s, 3H); ^{13}C NMR (101 MHz, $CDCl_3$) δ 152.1, 148.3, 139.3, 135.9, 135.2, 131.9, 129.4, 127.0, 126.7, 123.9, 122.4, 121.6, 121.2, 120.0, 119.7, 112.6, 110.0, 92.1, 78.3, 56.1, 9.0; HR-MS [ESI-MS(+)] calcd for $[M + H]^+$: $C_{21}H_{17}N_2OS$: 345.1056, found: 345.1055; IR (KBr): 3725, 3071, 2922, 1654, 1589, 1504, 1474, 1438, 1364, 1323, 1265, 1153, 1011, 850, 742, 705 cm^{-1}.

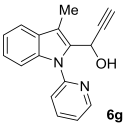
6g

1-(3-Methyl-1-(pyridin-2-yl)-1H-indol-2-yl)prop-2-yn-1-ol (6g): brown solid; m.p. 145.0 - 146.2 °C; 126.0 mg, 48% yield; ^{1}H NMR (500 MHz, $CDCl_3$) δ 8.54 (d, *J* = 4.7 Hz, 1H), 7.94 (t, *J* = 7.7 Hz, 1H), 7.65 – 7.61 (m, 2H), 7.52 (d, *J* = 8.2 Hz, 1H), 7.43 (d, *J* = 11.0 Hz, 1H), 7.31 – 7.29 (m, 1H), 7.27 – 7.19 (m, 2H), 5.76 – 5.73 (m, 1H), 2.43 (s, 3H), 2.04 (d, *J* = 2.2 Hz, 1H); ^{13}C NMR (126 MHz, $CDCl_3$) δ 151.9, 148.1, 139.2, 135.8, 134.8, 129.4, 123.9, 121.5, 121.2, 120.0, 119.6, 112.8, 110.1, 82.6, 72.7, 55.3, 9.0; HR-MS [ESI-MS(+)] calcd for $[M + H]^+$: $C_{17}H_{15}N_2O$: 263.1179, found: 263.1170; IR (KBr): 3555, 3288, 2922, 1591, 1474, 1455, 1439, 1363, 1319, 1283, 1093, 1017, 778, 740 cm^{-1}.

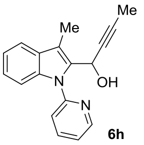
6h

1-(3-Methyl-1-(pyridin-2-yl)-1H-indol-2-yl)but-2-yn-1-ol (6h): yellow solid; m.p. 135.3 – 138.0 °C; 192.3 mg, 70% yield; ^{1}H NMR (400 MHz, $CDCl_3$) δ 8.59 (dd, *J* = 5.0, 1.2 Hz, 1H), 7.99 – 7.94 (m, 1H), 7.69 – 7.61 (m, 2H), 7.53 (d, *J* = 7.6 Hz, 1H), 7.33 – 7.21 (m, 4H), 5.72 (dd, *J* = 10.8, 2.2 Hz, 1H), 2.46 (s, 3H), 1.45 (d, *J* = 2.3 Hz, 3H); ^{13}C NMR (101 MHz, $CDCl_3$) δ 152.2, 148.4, 139.0, 135.9, 135.8, 129.4, 123.7, 121.4, 121.1, 119.9, 119.7, 112.1, 110.0, 81.4, 78.3, 55.7, 8.9, 3.3; HR-MS [ESI-MS(+)] calcd for $[M + H– H_2O]^+$: $C_{18}H_{15}N_2$: 259.1230, found: 259.1231; IR (KBr): 3251, 3057, 2919, 1592, 1474, 1441, 1364, 1231, 1146, 1091, 997, 916, 742 cm^{-1}.

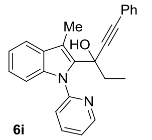
6i

3-(3-Methyl-1-(pyridin-2-yl)-1H-indol-2-yl)-1-phenylpent-1-yn-3-ol (6i): brown oil; 120.0 mg, 33% yield; ^{1}H NMR (400 MHz, $CDCl_3$) δ 8.63 – 8.62 (m, 1H), 7.92 – 7.87 (m, 1H), 7.75 – 7.68 (m, 2H), 7.64 (d, *J* = 8.1 Hz, 1H), 7.37 – 7.30 (m, 2H), 7.27 – 7.17 (m, 5H), 7.05 – 7.03 (m, 2H), 2.66 (s, 3H), 2.58 – 2.46 (m, 2H), 1.33 (d, *J* = 7.2 Hz, 3H); ^{13}C NMR (101 MHz, $CDCl_3$) δ 153.4, 148.4, 139.1, 137.4, 136.6, 131.4, 130.2, 128.0, 127.9, 123.5, 122.8, 122.0, 121.9, 120.8, 119.5, 110.8, 109.8, 91.5, 85.7, 69.9, 34.2, 11.0, 9.7; HR-MS [ESI-MS(+)] calcd for $[M + H]^+$: $C_{25}H_{23}N_2O$: 367.1805, found: 367.1801; IR (KBr): 3280, 3055, 2970, 2928, 1664, 1584, 1534, 1457, 1381, 1276, 1145, 1008, 924, 740, 700 cm^{-1}.

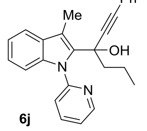
6j

3-(3-Methyl-1-(pyridin-2-yl)-1H-indol-2-yl)-1-phenylhex-1-yn-3-ol (6j): brown solid; m.p. 130.3 - 132.6 °C; 155.0 mg, 41% yield; ^{1}H NMR (500 MHz, $CDCl_3$) δ 8.57 (d, *J* = 4.7 Hz, 1H), 7.87 – 7.80 (m, 1H), 7.65 – 7.63 (m, 1H), 7.58 (d, *J* = 8.2 Hz, 2H), 7.30 – 7.25 (m, 2H), 7.22 – 7.14 (m, 5H), 7.00 (d, *J* = 7.2 Hz, 2H), 2.61 (s, 3H), 2.45 – 2.31 (m, 2H), 1.88 – 1.81 (m, 1H), 1.72 – 1.65 (m, 1H), 1.05 (t, *J* = 7.4 Hz, 3H); ^{13}C

NMR (126 MHz, $CDCl_3$) δ 153.4, 148.4, 139.1, 137.5, 136.6, 131.4, 130.2, 127.9, 127.9, 123.5, 122.8, 122.0, 121.9, 120.8, 119.5, 110.8, 109.7, 91.9, 85.6, 69.4, 43.5, 18.5, 14.3, 11.0; HR-MS [ESI-MS(+)] calcd for $[M + H]^+$: $C_{26}H_{25}N_2O$: 381.1961, found: 381.1963; IR (KBr): 3782, 3057, 2958, 2924, 2869, 1654, 1591, 1472, 1458, 1438, 1357, 1313, 1282, 1209, 1147, 967, 756, 741 cm^{-1}.

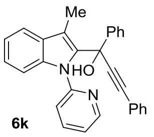

6k

1-(3-Methyl-1-(pyridin-2-yl)-1H-indol-2-yl)-1,3-diphenylprop-2-yn-1-ol (6k): yellow oil; 283.0 mg, 68% yield; ^1H NMR (400 MHz, $CDCl_3$) δ 8.60 – 8.58 (m, 1H), 8.38 (s, 1H), 7.84 – 7.75 (m, 3H), 7.62 (d, *J* = 7.3 Hz, 1H), 7.51 (d, *J* = 8.0 Hz, 1H), 7.38 – 7.30 (m, 4H), 7.27 – 7.19 (m, 6H), 7.11 (d, *J* = 7.0 Hz, 2H), 1.85 (s, 3H); ^{13}C NMR (101 MHz, $CDCl_3$) δ 153.0, 148.1, 143.5, 139.1, 137.7, 136.4, 131.4, 130.0, 128.1, 128.0, 128.0, 127.6, 126.9, 123.8, 122.7, 121.9, 121.7, 120.9, 119.7, 113.3, 109.7, 92.5, 86.3, 69.9, 9.5; HR-MS [ESI-MS(+)] calcd for $[M + H]^+$: $C_{29}H_{23}N_2O$: 415.1805, found: 415.1808; IR (KBr): 3240, 3065, 2922, 2856, 1654, 1572, 1534, 1458, 1277, 1147, 1011, 924, 741, 700 cm^{-1}.

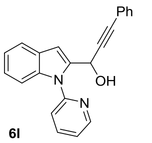

6l

3-Mhenyl-1-(1-(pyridin-2-yl)-1H-indol-2-yl)prop-2-yn-1-ol (6l): light yellow oil; 227.0 mg, 70% yield; ^1H NMR (400 MHz, $CDCl_3$) δ 8.64 – 8.63 (m, 1H), 8.02 – 7.97 (m, 1H), 7.76 – 7.70 (m, 2H), 7.59 (d, *J* = 8.3 Hz, 1H), 7.38 – 7.32 (m, 2H), 7.30 – 7.28 (m, 2H), 7.27 – 7.24 (m, 2H), 7.23 (t, *J* = 1.8 Hz, 1H), 7.16 – 7.14 (m, 2H), 6.91 (s, 1H), 5.82 (d, *J* = 8.6 Hz, 1H); ^{13}C NMR (101 MHz, $CDCl_3$) δ 151.6, 148.5, 140.0, 139.3, 136.8, 131.6, 128.4, 128.3, 128.1, 128.1, 123.6, 121.8, 121.7, 121.7, 119.6, 110.4, 105.7, 87.5, 85.3, 58.7; HR-MS [ESI-MS(+)] calcd for $[M + H – H_2O]^+$: $C_{22}H_{15}N_2$: 307.1230, found: 307.1234; IR (KBr): 3745, 3240, 3057, 2921, 2200, 1593, 1489, 1454, 1439, 1347, 1283, 1213, 1151, 1028, 958, 756, 691 cm^{-1}.

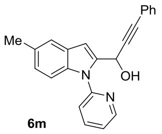

6m

1-(5-Methyl-1-(pyridin-2-yl)-1H-indol-2-yl)-3-phenylprop-2-yn-1-ol (6m): yellow oil; 220.0 mg, 65% yield; ^1H NMR (400 MHz, $CDCl_3$) δ 8.62 – 8.60 (m, 1H), 7.99 – 7.95 (m, 1H), 7.72 (d, *J* = 8.2 Hz, 1H), 7.48 (d, *J* = 8.0 Hz, 2H), 7.35 – 7.30 (m, 2H), 7.26 – 7.23 (m, 3H), 7.17 – 7.09 (m, 3H), 6.83 (s, 1H), 5.80 (d, *J* = 8.5 Hz, 1H), 2.49 (s, 3H); ^{13}C NMR (101 MHz, $CDCl_3$) δ 151.7, 148.4, 140.0, 139.3, 135.1, 131.8, 131.6, 131.1, 128.7, 128.3, 128.1, 125.1, 122.6, 121.6, 121.4, 119.4, 110.1, 105.4, 87.6, 85.2, 58.7, 21.4; HR-MS [ESI-MS(+)] calcd for $[M + H – H_2O]^+$: $C_{23}H_{17}N_2$: 321.1386, found: 321.1378; IR (KBr): 3745, 3252, 3058, 2917, 2202, 1595, 1474, 1440, 1375, 1340, 1305, 1174, 1029, 959, 874, 783, 775, 691 cm^{-1}.

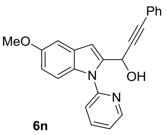

6n

1-(5-Methoxy-1-(pyridin-2-yl)-1H-indol-2-yl)-3-phenylprop-2-yn-1-ol (6n): yellow oil; 242.0 mg, 68% yield; ^1H NMR (500 MHz, $CDCl_3$) δ 8.59 (d, *J* = 4.7 Hz, 1H), 7.95 (t, *J* = 7.7 Hz, 1H), 7.68 (d, *J* = 8.1 Hz, 1H), 7.45 (d, *J* = 9.0 Hz, 1H), 7.34 – 7.29 (m, 1H), 7.25 – 7.16 (m, 4H), 7.14 – 7.05 (m, 3H), 6.91 – 6.89 (m, 1H), 6.80 (s, 1H), 5.75 (d, *J* = 8.2 Hz, 1H), 3.87 (s, 3H); ^{13}C NMR (126 MHz, $CDCl_3$) δ 155.3, 151.6, 148.4, 140.5, 139.3, 131.8, 131.6, 129.1, 128.3, 128.1, 122.5, 121.6, 119.2, 113.4, 111.3, 105.6, 103.3, 87.5, 85.3, 58.7, 55.8; HR-MS [ESI-MS(+)] calcd for $[M + H]^+$: $C_{23}H_{19}N_2O_2$: 355.1441, found: 355.1442; IR (KBr): 3249, 3062, 2927, 2831, 1618, 1596, 1583, 1475, 1449, 1380, 1304, 1280, 1204, 1172, 1031, 758, 692 cm^{-1}.

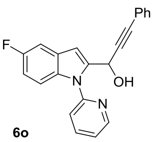

6o

1-(5-Fluoro-1-(pyridin-2-yl)-1H-indol-2-yl)-3-phenylprop-2-yn-1-ol (6o): brown oil; 150.0 mg, 44% yield; ^1H NMR (500 MHz, $CDCl_3$) δ 8.60 (d, *J* = 4.8 Hz, 1H), 7.96 (t, *J* = 7.8 Hz, 1H), 7.65 (d, *J* = 8.1 Hz, 1H), 7.46 – 7.44 (m, 1H), 7.36 – 7.28 (m, 2H), 7.25 – 7.18 (m, 3H), 7.11 (d, *J* = 7.3 Hz, 2H), 7.03 (d, *J* = 8.6

Hz, 1H), 7.00 – 6.96 (m, 1H), 6.83 (s, 1H), 5.75 (d, J = 8.6 Hz, 1H); ^{13}C NMR (126 MHz, $CDCl_3$) δ 158.7 (d, J = 236.9, $^1J_{CF}$), 151.3, 148.6, 141.5, 139.5, 133.3, 131.8, 131.6, 128.4, 128.1, 122.4, 122.0, 119.5, 111.7 (d, J = 25.2, $^2J_{CF}$), 111.3 (d, J = 10.1, $^3J_{CF}$), 106.6 (d, J = 23.9, $^2J_{CF}$), 105.4 (d, J = 5.0, $^3J_{CF}$), 87.2, 85.5, 58.6; HR-MS [ESI-MS(+)] calcd for $[M + H – H_2O]^+$: $C_{22}H_{14}FN_2$: 325.1136, found: 325.1128; IR (KBr): 3745, 3274, 2923, 2200, 1587, 1475, 1450, 1381, 1301, 1174, 1029, 954, 858, 786, 757, 691 cm^{-1}.

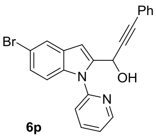

6p

1-(5-Bromo-1-(pyridin-2-yl)-1H-indol-2-yl)-3-phenylprop-2-yn-1-ol (6p): yellow solid; m.p. 155.9 - 157.4 ^{o}C; 150.0 mg, 37% yield; ^{1}H NMR (400 MHz, $CDCl_3$) δ 8.66 – 8.63 (m, 1H), 8.03 – 7.99 (m, 1H), 7.81 (d, J = 1.8 Hz, 1H), 7.67 (d, J = 8.1 Hz, 1H), 7.43 – 7.34 (m, 3H), 7.28 – 7.21 (m, 3H), 7.15 – 7.13 (m, 2H), 7.03 (d, J = 8.7 Hz, 1H), 6.83 (s, 1H), 5.79 (d, J = 8.6 Hz, 1H); ^{13}C NMR (101 MHz, $CDCl_3$) δ 151.1, 148.7, 141.1, 139.5, 135.5, 131.6, 130.1, 128.4, 128.1, 126.4, 124.2, 122.3, 122.2, 119.6, 114.6, 111.9, 104.9, 87.0, 85.6, 58.6; HR-MS [ESI-MS(+)] calcd for $[M + H – H_2O]^+$: $C_{22}H_{14}BrN_2$: 385.0335, found: 385.0329; IR (KBr): 3743, 3264, 3064, 2921, 2854, 2210, 1588, 1473, 1444, 1376, 1339, 1299, 1208, 1143, 1024, 873, 758, 690 cm^{-1}.

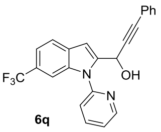

6q

3-Phenyl-1-(1-(pyridin-2-yl)-6-(trifluoromethyl)-1H-indol-2-yl)prop-2-yn-1-ol (6q): light pink solid; m.p. 117.1 - 119.3 ^{o}C; 221.0 mg, 56% yield; ^{1}H NMR (400 MHz, $CDCl_3$) δ 8.68 – 8.67 (m, 1H), 8.08 – 8.04 (m, 1H), 7.82 (s, 1H), 7.79 (d, J = 8.3 Hz, 1H), 7.71 (d, J = 8.1 Hz, 1H), 7.49 (d, J = 8.3 Hz, 1H), 7.46 – 7.42 (m, 1H), 7.30 – 7.21 (m, 3H), 7.17 – 7.15 (m, 2H), 6.97 (d, J = 8.8 Hz, 2H), 5.84 (d, J = 8.7 Hz, 1H); ^{13}C NMR (101 MHz, $CDCl_3$) δ 150.9, 148.9, 142.7, 139.8, 135.9, 131.6, 130.8, 128.5, 128.2, 124.9 (d, J = 272.7, $^1J_{CF}$), 125.6 (d, J = 32.3, $^2J_{CF}$), 122.6, 122.2, 122.1, 119.7, 118.3 (q, J = 3.0, $^3J_{CF}$),107.9 (q, J = 6.0, $^3J_{CF}$), 105.4, 86.9, 85.7, 58.6; HR-MS [ESI-MS(+)] calcd for $[M + H – H_2O]^+$: $C_{23}H_{14}F_3N_2$: 375.1104, found: 375.1098; IR (KBr): 3745, 3056, 2923, 1592, 1474, 1440, 1344, 1324, 1304, 1164, 1116, 1050, 1024, 971, 829, 757, 691 cm^{-1}.

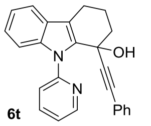

6t

1-(Phenylethynyl)-9-(pyridin-2-yl)-2,3,4,9-tetrahydro-1H-carbazol-1-ol (6t): light yellow solid; m.p. 96.9 - 99.4 ^{o}C; 211.0 mg, 56% yield; ^{1}H NMR (500 MHz, $CDCl_3$) δ 8.58 – 8.57 (m, 1H), 7.92 – 7.89 (m, 1H), 7.68 – 7.64 (m, 2H), 7.60 (d, J = 7.7 Hz, 1H), 7.54 (d, J = 8.1 Hz, 1H), 7.28 – 7.20 (m, 3H), 7.18 – 7.10 (m, 3H), 6.92 – 6.85 (m, 2H), 2.93 (d, J = 16.1 Hz, 1H), 2.79 – 2.70 (m, 1H), 2.56 (d, J = 13.1 Hz, 1H), 2.31 – 2.13 (m, 2H), 2.05 – 1.97 (m, 1H); ^{13}C NMR (126 MHz, $CDCl_3$) δ 152.1, 148.4, 139.1, 137.1, 136.0, 131.4, 128.1, 127.9, 123.7, 122.7, 121.3, 121.2, 119.7, 119.3, 115.3, 110.1, 92.3, 83.2, 62.8, 39.8, 21.7, 19.4; HR-MS [ESI-MS(+)] calcd for $[M + H – H_2O]^+$: $C_{25}H_{19}N_2$: 347.1543, found: 347.1555; IR (KBr): 3281, 3056, 2928, 2840, 1592, 1474, 1443, 1371, 1346, 1263, 1137, 973, 917, 831, 788, 755, 740, 691 cm^{-1}.

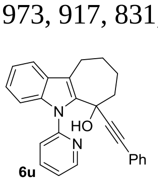

6u

6-(Phenylethynyl)-5-(pyridin-2-yl)-5,6,7,8,9,10-hexahydrocyclohepta[b]indol-6-ol (6u): light yellow oil; 243.0 mg, 64% yield; ^{1}H NMR (400 MHz, $CDCl_3$) δ 8.63 – 8.62 (m, 1H), 8.09 (d, J = 1.2 Hz, 1H), 7.93 – 7.89 (m, 1H), 7.70 – 7.67 (m, 2H), 7.49 – 7.47 (m, 1H), 7.34 – 7.24 (m, 3H), 7.24 – 7.14 (m, 3H), 6.96 – 6.93 (m, 2H), 3.15 – 2.93 (m, 2H), 2.71 – 2.49 (m, 2H), 2.31 – 2.17 (m, 1H), 2.09 – 2.01 (m, 2H), 1.98 – 1.88 (m, 1H); ^{13}C NMR (101 MHz, $CDCl_3$) δ 152.9, 148.3, 139.2, 138.5, 136.2, 131.4, 128.8, 127.9, 123.5, 122.7, 121.7, 121.0, 120.9, 119.4, 117.8, 110.0, 92.8, 84.1, 68.0, 37.6, 25.4, 20.6, 20.5; HR-MS [ESI-MS(+)] calcd for $[M + H]^+$: $C_{26}H_{21}N_2$: 361.1699, found: 361.1694; IR (KBr): 3260, 3056, 2931, 2860, 1591, 1570, 1474, 1456, 1440, 1365, 1313, 1021, 1005, 915, 777, 757, 740, 691 cm^{-1}.

Method F:

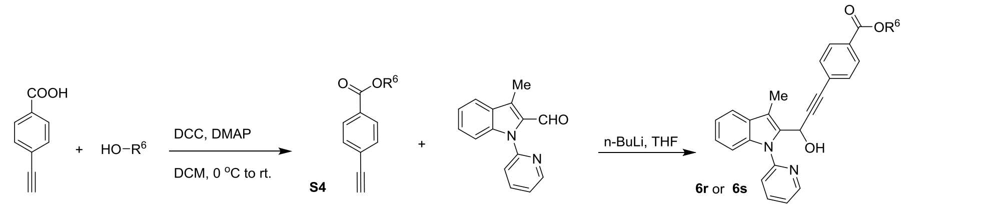

Synthetic procedure of α-(2-indolyl)propargyl alcohols 6r and 6s: The following starting materials **S4** were prepared according to the literature procedures [23]. To an oven-dried 50 mL flask, 4-ethynyl-benzoic acid (6.0 mmol), alcohol (5.5 mmol), DMAP (1.1 mmol) and DCM (30 mL) were added sequentially under Ar at 0 °C. Then DCC (6 mmol) was added and stirred for 10 min at this temperature and stirred at room temperature overnight. The reaction mixture filtered through a short pad of silica gel and washed by DCM. The filtrate was concentrated in vacuo before it was purified by flash chromatography on silica gel to afford the desired compound **S4**. After obtaining **S4**, we also used the **method E** to synthesize **6r** and **6s**.

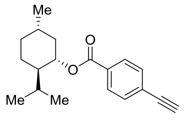

S4-1

(1S,2R,5S)-2-isopropyl-5-methylcyclohexyl 4-ethynylbenzoate (S4-1) [5]: ^{1}H NMR (400 MHz, $CDCl_3$) δ 8.02 (d, *J* = 8.4 Hz, 2H), 7.57 (d, *J* = 8.3 Hz, 2H), 4.99 – 4.92 (m, 1H), 3.24 (s, 1H), 2.16 – 2.13 (m, 1H), 2.00 – 1.92 (m, 1H), 1.80 – 1.69 (m, 2H), 1.64 – 1.49 (m, 2H), 1.20 – 1.07 (m, 2H), 0.96 – 0.93 (m, 7H), 0.81 (d, *J* = 7.0 Hz, 3H); ^{13}C NMR (101 MHz, $CDCl_3$) δ 165.4, 132.0, 130.9, 129.4, 126.5, 82.9, 79.9, 75.2, 47.3, 41.0, 34.3, 31.5, 26.5, 23.6, 22.1, 20.8, 16.5;

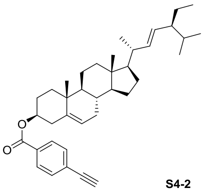

S4-2

(3S,8S,9S,10R,13R,14S,17R)-17-((2R,5S,E)-5-ethyl-6-methylhept-3-en-2-yl)-10,13-dimethyl-2,3,4,7,8,9,10,11,12,13,14,15,16,17-tetradecahydro-1H-cyclopenta[a]phenanthren-3-yl 4-ethynylbenzoate (S4-2): white solid; m.p. 158.4 – 161.8 °C; 2.50 g, 84% yield; ^{1}H NMR (400 MHz, $CDCl_3$) δ 8.01 (d, *J* = 8.4 Hz, 2H), 7.56 (d, *J* = 8.4 Hz, 2H), 5.44 (d, *J* = 4.2 Hz, 1H), 5.22 – 5.16 (m, 1H), 5.08 – 5.02 (m, 1H), 4.93 – 4.81 (m, 1H), 3.24 (s, 1H), 2.48 (d, *J* = 7.7 Hz, 2H), 2.10 – 1.92 (m, 5H), 1.80 – 1.70 (m, 2H), 1.63 – 1.42 (m, 8H), 1.37 – 1.12 (m, 6H), 1.09 (s, 3H), 1.05 (d, *J* = 6.6 Hz, 5H), 0.88 (d, *J* = 6.3 Hz, 3H), 0.85 (s, 1H), 0.83 (d, *J* = 6.9 Hz, 5H), 0.73 (s, 3H); ^{13}C NMR (101 MHz, $CDCl_3$) δ 165.3, 139.5, 138.3, 132.0, 130.9, 129.4, 129.3, 126.5, 122.9, 82.9, 79.9, 74.9, 56.8, 56.0, 51.3, 50.1, 42.2, 40.5, 39.7, 38.2, 37.0, 36.7, 31.9, 31.9, 28.9, 27.9, 25.4, 24.4, 21.3, 21.1, 21.0, 19.4, 19.0, 12.3, 12.1; HR-MS [ESI-MS(+)] calcd for $[M + Na]^+$: $C_{38}H_{52}NaO_2$: 563.3860, found: 563.3866; IR (KBr): 3250, 2949, 2864, 2104, 1705, 1605, 1460, 1307, 1269, 1173, 1120, 1106, 1019, 974, 859, 769 cm^{-1}.

(1R,2S,5R)-2-isopropyl-5-methylcyclohexyl 4-(3-hydroxy-3-(3-methyl-1-(pyridin-2-yl)-1H-indol-2-yl)prop-1-yn-1-yl)benzoate (6r): brown solid; m.p. 110.7 – 114.6 °C; 140.0 mg, 27% yield; ^{1}H NMR (400 MHz, $CDCl_3$) δ 8.63 (d, *J* = 4.8 Hz, 1H), 7.96 (t, *J* = 7.8 Hz, 1H), 7.86 (d, *J* = 7.5 Hz, 2H), 7.72 (d, *J* = 8.1 Hz, 1H), 7.67 (d, *J* = 7.2 Hz, 1H), 7.57 (d, *J* = 8.0 Hz, 1H), 7.50 (d, *J* = 10.8 Hz, 1H), 7.35 – 7.24 (m, 3H), 7.02 (d, *J* = 8.0 Hz, 2H), 6.00 (d, *J* = 9.8 Hz, 1H), 4.96 – 4.90 (m, 1H), 2.51 (s, 3H), 2.11 (d, *J* = 12.1 Hz, 1H), 1.96 – 1.91 (m, 1H), 1.75 (d, *J* = 11.5 Hz, 2H), 1.55 (t, *J* = 11.7 Hz, 2H), 1.18 – 1.04 (m, 2H), 0.95 – 0.92 (m, 7H), 0.80 (d, *J* = 6.9 Hz, 3H); ^{13}C NMR (101 MHz, $CDCl_3$) δ 165.5, 152.1, 148.4, 139.2, 135.8, 135.1, 131.3, 130.2, 129.4, 129.1, 127.0, 124.0, 121.5, 121.3, 120.0, 119.6, 112.9, 110.1, 91.2, 84.3, 75.1, 56.0, 47.3, 41.0, 34.3, 31.4, 26.5, 23.6, 22.0, 20.8, 16.5, 9.0; HR-MS [ESI-MS(+)] calcd for $[M + H]^+$:

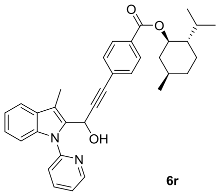

6r

$C_{34}H_{37}N_2O_3$: 521.2799, found: 521.2796; IR (KBr): 2955, 2922, 2857, 1712, 1597, 1505, 1457, 1377, 1273, 1175, 1106, 1018, 965, 745 cm^{-1}.

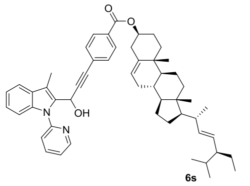

6s

(3S,8S,9S,10R,13R,14S,17R)-17-((2R,5S,E)-5-ethyl-6-methylhept-3-en-2-yl)-10,13-dimethyl-2,3,4,7,8,9,10,11,12,13,14,15,16,17-tetradecahydro-1H-cyclopenta[a]phenanthren-3-yl 4-(3-hydroxy-3-(3-methyl-1-(pyridin-2-yl)-1H-indol-2-yl)prop-1-yn-1-yl)benzoate (6s): light yellow solid; m.p. 152.7 – 153.5 °C; 220.0 mg, 28% yield; ^{1}H NMR (400 MHz, $CDCl_3$) δ 8.63 (dd, *J* = 5.0, 1.3 Hz, 1H), 7.98 – 7.94 (m, 1H), 7.85 (d, *J* = 8.4 Hz, 2H), 7.71 (d, *J* = 8.1 Hz, 1H), 7.69 – 7.65 (m, 1H), 7.57 (d, *J* = 7.9 Hz, 1H), 7.49 (d, *J* = 11.0 Hz, 1H), 7.34 – 7.24 (m, 3H), 7.00 (d, *J* = 8.4 Hz, 2H), 5.99 (d, *J* = 11.0 Hz, 1H), 5.43 (d, *J* = 4.0 Hz, 1H), 5.22 – 5.16 (m, 1H), 5.08 – 5.02 (m, 1H), 4.90 – 4.78 (m, 1H), 2.50 (s, 3H), 2.45 (d, *J* = 7.7 Hz, 2H), 2.09 – 1.90 (m, 5H), 1.75 – 1.70 (m, 2H), 1.61 – 1.44 (m, 8H), 1.27 – 1.18 (m, 6H), 1.10 – 1.03 (m, 8H), 0.88 (d, *J* = 6.2 Hz, 3H), 0.86 (s, 1H), 0.83 (d, *J* = 7.0 Hz, 5H), 0.73 (s, 3H); ^{13}C NMR (101 MHz, $CDCl_3$) δ 165.3, 152.1, 148.4, 139.5, 139.2, 138.3, 135.9, 135.0, 131.2, 130.2, 129.4, 129.3, 129.1, 126.9, 123.9, 122.9, 121.5, 121.3, 120.0, 119.6, 112.9, 110.1, 100.0, 91.2, 84.3, 77.3, 74.8, 56.8, 55.9, 51.3, 50.1, 42.2, 40.5, 39.6, 38.2, 37.0, 36.7, 31.9, 29.7, 28.9, 27.9, 25.4, 24.4, 21.3, 21.1, 21.0, 19.4, 19.0, 12.3, 12.1, 9.0; HR-MS [ESI-MS(+)] calcd for $[M + H]^+$: $C_{53}H_{65}N_2O_3$: 777.4990, found: 777.4984; IR (KBr): 2952, 2864, 1716, 1591, 1475, 1439, 1365, 1271, 1174, 1112, 1017, 969, 856, 772, 740 cm^{-1}.

1.3. Optimization of reaction conditions

Supplementary Table 1 | Mn-catalysts screening for the reaction [a]

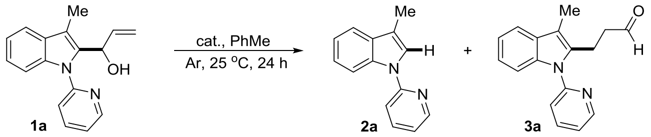

entry	catalysts	yield **2a**/**3a** (%) [b]
1	$MnCl_2$	0/0
2	$Mn(OTf)_2$	0/0
3	$Mn(OAc)_3{\cdot}H_2O$	0/0
4	$Mn_2(CO)_{10}$	0/23
5	$Mn(CO)_5Br$	3/27

[a] All the reactions were carried out employing allylic alcohol **1a** (0.20 mmol), different Mn catalysts (0.01 mmol, 5 mol %) and PhMe (2.0 mL) under Ar atmosphere. The reaction mixture was then allowed to stir at 25 °C for 24 h, followed by flash chromatography on SiO_2. [b] Isolated yield.

Supplementary Table 2 | The effect of the temperature for the reaction [a]

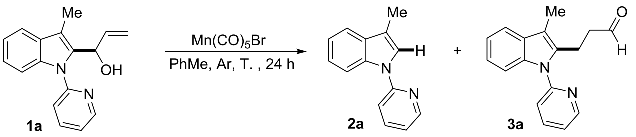

entry	T(°C)	yield **2a**/**3a** (%) [b]
1	25	3/27
2	45	4/56

3	75	7/61
4	85	5/54

[a] All the reactions were carried out employing allylic alcohol **1a** (0.20 mmol), $Mn(CO)_5Br$ (0.01 mmol, 5 mol %) and PhMe (2.0 mL) under Ar atmosphere. The reaction mixture was then allowed to stir at different temperature for 24 h, followed by flash chromatography on SiO_2. [b] Isolated yield.

Supplementary Table 3 | Various solvents screening for the reaction [a]

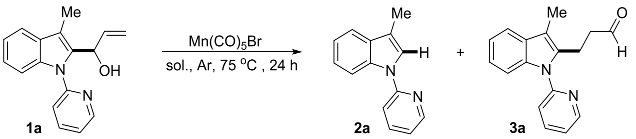

entry	solvent	yield **2a**/**3a** (%) [b]
1	Toluene	7/61
2	Benzotrifluoride	0/43
3	THF	0/17
4	DMF	0/38
5	CH_3CN	0/0
6	DCE	0/80

[a] All the reactions were carried out employing allylic alcohol **1a** (0.20 mmol), $Mn(CO)_5Br$ (0.01 mmol, 5 mol %) and solvent (2.0 mL) under Ar atmosphere. The reaction mixture was then allowed to stir at 75 ^oC for 24 h, followed by flash chromatography on SiO_2. [b] Isolated yield.

Supplementary Table 4 | Other transition-metal catalysts screening for the reaction [a]

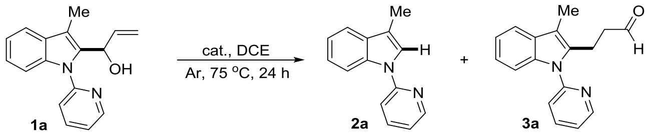

entry	catalysts	yield **2a**/**3a** (%) [b]
1	$Cp^*Co(CO)I_2$	0/5
2	$Cp^*Rh(CH_3CN)_3(SbF_6)_2$	47/25

[a]All the reactions were carried out employing allylic alcohol **1a** (0.20 mmol), catalyst (0.01 mmol, 5 mol %) and DCE (2.0 mL) under Ar atmosphere. The reaction mixture was then allowed to stir at 75 ^oC for 24 h, followed by flash chromatography on SiO_2. [b]Isolated yield.

1.4. Detail characterization for the sigmatropic rearrangement products 3 and 5

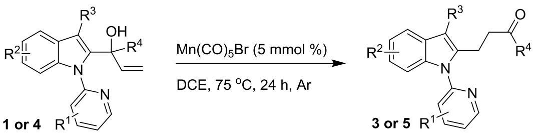

An oven-dried sealed tube charged allylic alcohols **1** or **4** (0.20 mmol), $Mn(CO)_5Br$ (0.01 mmol, 5 mol %) and DCE (2.0 mL) under Ar atmosphere. The reaction mixture was then allowed to stir at 75 ℃ for 24 h. After the reaction mixture was cooled down, the corresponding reaction mixture

was purified by flash chromatography on silica gel using ethyl acetate/petroleum ether (1: 10) as eluent to afford the desired products **2a**, **3** or **5**.

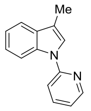
2a

3-Methyl-1-(pyridin-2-yl)-1H-indole (2a) [6]**:** ^{1}H NMR (400 MHz, $CDCl_3$) δ 8.59 – 8.56 (m, 1H), 8.28 (d, *J* = 8.3 Hz, 1H), 7.83 – 7.77 (m, 1H), 7.65 (d, *J* = 7.6 Hz, 1H), 7.56 (s, 1H), 7.47 (d, *J* = 8.3 Hz, 1H), 7.37 – 7.34 (m, 1H), 7.29 – 7.26 (m, 1H), 7.15 – 7.12 (m, 1H), 2.42 (s, 3H); ^{13}C NMR (101 MHz, $CDCl_3$) δ 152.6, 148.9, 138.3, 135.4, 131.1, 123.3, 123.2, 120.9, 119.5, 119.1, 114.8, 114.0, 113.1, 9.7;

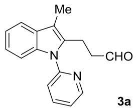
3a

3-(3-Methyl-1-(pyridin-2-yl)-1H-indol-2-yl)propanal (3a): The title compound was prepared from allylic alcohol **1a** (0.20 mmol, 52.8 mg) and was purified by column chromatography (10:1 = petroleum ether: ethyl acetate) to give a light yellow solid; m.p. 68.3 - 71.1 ^{o}C; 42.0 mg, 80% yield; ^{1}H NMR (500 MHz, $CDCl_3$) δ 9.70 (s, 1H), 8.60 (dd, *J* = 4.7, 1.4 Hz, 1H), 7.89 – 7.85 (m, 1H), 7.55 – 7.53 (m, 1H), 7.45 (d, *J* = 8.0 Hz, 1H), 7.33 – 7.31 (m, 1H), 7.29 – 7.27 (m, 1H), 7.17 – 7.13 (m, 2H), 3.21 – 3.16 (m, 2H), 2.71 – 2.66 (m, 2H), 2.32 (s, 3H); ^{13}C NMR (126 MHz, $CDCl_3$) δ 201.5, 151.6, 149.6, 138.4, 136.4, 135.0, 129.4, 122.2, 121.8, 120.7, 120.5, 118.4, 110.5, 109.9, 43.9, 17.9, 8.8; HR-MS [ESI-MS(+)] calcd for [M + H]$^+$: $C_{17}H_{17}N_2O$: 265.1335, found: 265.1339; IR (KBr): 3050, 2921, 1747, 1722, 1715, 1471, 1434, 1361, 1338, 991, 740 cm^{-1}.

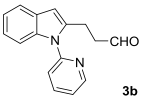
3b

3-(1-(Pyridin-2-yl)-1H-indol-2-yl)propanal (3b) [7]**:** The title compound was prepared from allylic alcohol **1b** (0.20 mmol, 50.0 mg) and was purified by column chromatography (10:1 = petroleum ether: ethyl acetate) to give a light yellow oil; 33.0 mg, 66% yield; ^{1}H NMR (400 MHz, $CDCl_3$) δ 9.81 (t, *J* = 1.3 Hz, 1H), 8.69 – 8.65 (m, 1H), 7.95 – 7.90 (m, 1H), 7.62 – 7.58 (m, 1H), 7.50 (d, *J* = 8.0 Hz, 1H), 7.38 – 7.33 (m, 2H), 7.19 – 7.15 (m, 2H), 6.47 (d, *J* = 0.7 Hz, 1H), 3.21 (t, *J* = 7.5 Hz, 2H), 2.88 – 2.84 (m, 2H); ^{13}C NMR (101 MHz, $CDCl_3$) δ 201.4, 151.2, 149.8, 139.4, 138.5, 137.3, 128.5, 122.2, 122.1, 121.0, 120.9, 120.2, 110.1, 102.6, 42.9, 20.4.

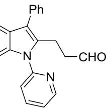
3c

3-(3-Phenyl-1-(pyridin-2-yl)-1H-indol-2-yl)propanal (3c): The title compound was prepared from allylic alcohol **1c** (0.20 mmol, 62.5 mg) and was purified by column chromatography (10:1 = petroleum ether: ethyl acetate) to give a light yellow oil; 46.3 mg, 71% yield; ^{1}H NMR (500 MHz, $CDCl_3$) δ 9.52 (s, 1H), 8.65 (dd, *J* = 4.8, 1.3 Hz, 1H), 7.94 – 7.91 (m, 1H), 7.59 (d, *J* = 7.3 Hz, 1H), 7.54 (d, *J* = 8.0 Hz, 1H), 7.52 – 7.46 (m, 4H), 7.37 – 7.34 (m, 3H), 7.21 – 7.14 (m, 2H), 3.32 – 3.26 (m, 2H), 2.56 (t, *J* = 7.7 Hz, 2H); ^{13}C NMR (126 MHz, $CDCl_3$) δ 201.1, 151.3, 149.9, 138.6, 136.7, 135.4, 134.6, 129.9, 128.7, 128.3, 126.7, 122.7, 122.4, 121.2, 121.1, 119.3, 118.0, 110.1, 43.9, 18.2; HR-MS [ESI-MS(+)] calcd for [M + H]$^+$: $C_{22}H_{19}N_2O$: 327.1492, found: 327.1488 ; IR (KBr): 3053, 2900, 1715, 1558, 1470, 1456, 1435, 1371, 1338, 1189, 743 cm^{-1}.

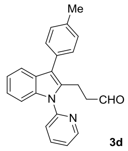
3d

3-(1-(Pyridin-2-yl)-3-(p-tolyl)-1H-indol-2-yl)propanal (3d): The title compound was prepared from allylic alcohol **1d** (0.20 mmol, 68.0 mg) and was purified by column chromatography (10:1 = petroleum ether: ethyl acetate) to give a brown solid; m.p. 62.4 - 65.1 ^{o}C; 51.0 mg, 75% yield; ^{1}H NMR (400 MHz, $CDCl_3$) δ 9.57 (s, 1H), 8.69 (dd, *J* = 4.8, 1.3 Hz, 1H), 7.98 – 7.94 (m, 1H), 7.64 – 7.62 (m, 1H), 7.58 (d, *J* = 8.0 Hz, 1H), 7.45 (d, *J* = 8.0 Hz, 2H), 7.41 – 7.33 (m, 4H), 7.25 – 7.18 (m, 2H), 3.36 – 3.29 (m, 2H), 2.62 – 2.58 (m, 2H), 2.48 (s, 3H); ^{13}C NMR (101 MHz, $CDCl_3$) δ 201.2, 151.3, 149.8, 138.6, 136.6, 136.4, 135.2, 131.6, 129.8, 129.5, 128.4, 122.7, 122.4, 121.2, 121.1, 119.4, 117.9, 110.1, 43.9, 21.3, 18.3; HR-MS [ESI-MS(+)] calcd for [M + H]$^+$: $C_{23}H_{21}N_2O$: 341.1648, found: 341.1643;

IR (KBr): 3051, 2923, 2854, 1721, 1586, 1567, 1470, 1457, 1437, 1370, 1317, 1190, 1148, 821, 786, 743 cm^{-1}.

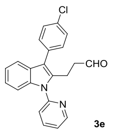

3e

1-(3-(4-Chlorophenyl)-1-(pyridin-2-yl)-1H-indol-2-yl)prop-2-en-1-ol (3e): The title compound was prepared from allylic alcohol **1e** (0.20 mmol, 72.0 mg) and was purified by column chromatography (10:1 = petroleum ether: ethyl acetate) to give a light yellow oil; 50.4 mg, 70% yield; ^{1}H NMR (400 MHz, $CDCl_3$) δ 9.57 (s, 1H), 8.72 – 8.67 (m, 1H), 7.99 – 7.95 (m, 1H), 7.60 – 7.55 (m, 2H), 7.52 – 7.45 (m, 4H), 7.42 – 7.37 (m, 2H), 7.26 – 7.18 (m, 2H), 3.35 – 3.27 (m, 2H), 2.61 – 2.57 (m, 2H); ^{13}C NMR (101 MHz, $CDCl_3$) δ 200.8, 151.1, 149.9, 138.7, 136.7, 135.6, 133.2, 132.6, 131.2, 129.0, 128.0, 122.89, 122.6, 121.4, 121.2, 119.0, 116.7, 110.2, 43.8, 18.1; HR-MS [ESI-MS(+)] calcd for $[M + H]^+$: $C_{22}H_{18}ClN_2O$: 361.1102, found: 361.1097; IR (KBr): 2923, 2852, 1734, 1654, 1617, 1577, 1571, 1467, 1437, 1420, 1382, 745 cm^{-1}.

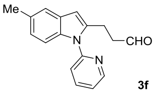

3f

3-(5-Methyl-1-(pyridin-2-yl)-1H-indol-2-yl)propanal (3f): The title compound was prepared from allylic alcohol **1f** (0.20 mmol, 52.8 mg) and was purified by column chromatography (10:1 = petroleum ether: ethyl acetate) to give a brown solid; m.p. 86.7 – 88.7 °C; 38.5 mg, 73% yield; ^{1}H NMR (400 MHz, $CDCl_3$) δ 9.80 (t, J = 1.3 Hz, 1H), 8.67 – 8.64 (m, 1H), 7.93 – 7.89 (m, 1H), 7.49 (d, J = 8.0 Hz, 1H), 7.39 (s, 1H), 7.35 – 7.32 (m, 1H), 7.26 (d, J = 8.4 Hz, 1H), 7.01 – 6.99 (m, 1H), 6.38 (s, 1H), 3.21 (t, J = 7.5 Hz, 2H), 2.87 – 2.83 (m, 2H), 2.47 (s, 3H); ^{13}C NMR (101 MHz, $CDCl_3$) δ 201.5, 151.4, 149.7, 139.5, 138.4, 135.6, 130.2, 128.8, 123.6, 122.0, 120.7, 120.0, 109.8, 102.3, 42.9, 21.4, 20.5; HR-MS [ESI-MS(+)] calcd for $[M + H]^+$: $C_{17}H_{17}N_2O$: 265.1335, found: 265.1335; IR (KBr): 2920, 2855, 1719, 1587, 1470, 1437, 1378, 1298, 786 cm^{-1}.

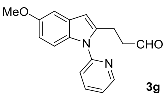

3g

3-(5-Methoxy-1-(pyridin-2-yl)-1H-indol-2-yl)propanal (3g): The title compound was prepared from allylic alcohol **1g** (0.20 mmol, 56.0 mg) and was purified by column chromatography (8:1 = petroleum ether: ethyl acetate) to give a light yellow oil; 39.8 mg, 71% yield; ^{1}H NMR (400 MHz, $CDCl_3$) δ 9.79 (s, 1H), 8.65 (dd, J = 4.9, 1.2 Hz, 1H), 7.92 – 7.87 (m, 1H), 7.46 (d, J = 8.0 Hz, 1H), 7.34 – 7.31 (m, 1H), 7.27 (d, J = 8.9 Hz, 1H), 7.07 (d, J = 2.4 Hz, 1H), 6.82 (dd, J = 8.9, 2.5 Hz, 1H), 6.39 (s, 1H), 3.87 (s, 3H), 3.20 (t, J = 7.5 Hz, 2H), 2.86 – 2.82 (m, 2H); ^{13}C NMR (101 MHz, $CDCl_3$) δ 201.4, 154.9, 151.3, 149.7, 140.0, 138.5, 132.3, 129.1, 122.0, 120.6, 111.7, 111.0, 102.6, 102.3, 55.8, 42.9, 20.5; HR-MS [ESI-MS(+)] calcd for $[M + H]^+$: $C_{17}H_{17}N_2O_2$: 281.1285, found: 281.1276; IR (KBr): 2929, 2831, 1720, 1616, 1583, 1474, 1450, 1438, 1389, 1205, 1173, 1033, 843, 786, 746 cm^{-1}.

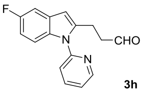

3h

3-(5-Fluoro-1-(pyridin-2-yl)-1H-indol-2-yl)propanal (3h): The title compound was prepared from allylic alcohol **1h** (0.20 mmol, 53.6 mg) and was purified by column chromatography (10:1 = petroleum ether: ethyl acetate) to give a light yellow oil; 38.7 mg, 72% yield; ^{1}H NMR (400 MHz, $CDCl_3$) δ 9.80 (s, 1H), 8.68 – 8.66 (m, 1H), 7.95 – 7.91 (m, 1H), 7.46 (d, J = 8.0 Hz, 1H), 7.39 – 7.35 (m, 1H), 7.29 – 7.21 (m, 2H), 6.92 – 6.87 (m, 1H), 6.41 (s, 1H), 3.18 (t, J = 7.4 Hz, 2H), 2.88 – 2.84 (m, 2H); ^{13}C NMR (101 MHz, $CDCl_3$) δ 201.1, 158.5 (d, J = 236.3, $^{1}J_{CF}$), 151.0, 149.8, 141.1, 138.6, 133.8, 128.9 (d, J = 10.1, $^{3}J_{CF}$), 122.5, 120.9, 110.9 (d, J = 9.1, $^{3}J_{CF}$), 110.1 (d, J = 26.3, $^{2}J_{CF}$), 105.2 (d, J = 23.2, $^{2}J_{CF}$), 102.5 (d, J = 4.0, $^{3}J_{CF}$), 42.8, 20.3; HR-MS [ESI-MS(+)] calcd for $[M + H]^+$: $C_{16}H_{14}FN_2O$: 269.1085, found: 269.1078; IR (KBr): 3065, 2924, 1724, 1584, 1471, 1449, 1437, 1387, 1177, 1108, 955, 855, 781 cm^{-1}.

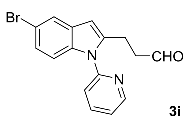
3i

3-(5-Bromo-1-(pyridin-2-yl)-1H-indol-2-yl)propanal (3i): The title compound was prepared from allylic alcohol **1i** (0.20 mmol, 65.6 mg) and was purified by column chromatography (10:1 = petroleum ether: ethyl acetate) to give a yellow oil; 42.8 mg, 65% yield; ^{1}H NMR (400 MHz, $CDCl_3$) δ 9.80 (s, 1H), 8.67 (dd, *J* = 4.8, 1.0 Hz, 1H), 7.95 – 7.91 (m, 1H), 7.70 (d, *J* = 1.5 Hz, 1H), 7.44 (d, *J* = 7.6 Hz, 1H), 7.40 – 7.35 (m, 1H), 7.25 – 7.18 (m, 2H), 6.38 (s, 1H), 3.17 (t, *J* = 7.4 Hz, 2H), 2.85 (t, *J* = 7.5 Hz, 2H); ^{13}C NMR (101 MHz, $CDCl_3$) δ 201.0, 150.8, 149.9, 140.8, 138.7, 135.9, 130.1, 124.8, 122.6, 120.9, 114.0, 111.6, 101.9, 42.7, 20.2; HR-MS [ESI-MS(+)] calcd for $[M + H]^+$: $C_{16}H_{14}BrN_2O$: 329.0284, found: 329.0279; IR (KBr): 2921, 1719, 1584, 1468, 1438, 1382, 1338, 1204, 1144, 1049, 864, 782, 740 cm^{-1}.

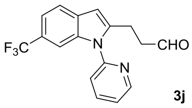
3j

3-(1-(Pyridin-2-yl)-6-(trifluoromethyl)-1H-indol-2-yl)propanal (3j): The title compound was prepared from allylic alcohol **1j** (0.20 mmol, 63.6 mg) and was purified by column chromatography (10:1 = petroleum ether: ethyl acetate) to give a brown oil; 46.00 mg, 70% yield; ^{1}H NMR (400 MHz, $CDCl_3$) δ 9.81 (s, 1H), 8.71 (dd, *J* = 4.9, 1.2 Hz, 1H), 8.00 – 7.96 (m, 1H), 7.67 (d, *J* = 8.3 Hz, 1H), 7.58 (s, 1H), 7.48 (d, *J* = 8.0 Hz, 1H), 7.44 – 7.39 (m, 2H), 6.52 (s, 1H), 3.19 (t, *J* = 7.4 Hz, 2H), 2.88 (t, *J* = 7.4 Hz, 2H); ^{13}C NMR (101 MHz, $CDCl_3$) δ 200.8, 150.4, 150.1, 142.4, 138.9, 136.3, 130.8, 125.2 (d, *J* = 272.7, $^1J_{CF}$), 124.1 (d, *J* = 31.3, $^2J_{CF}$), 123.0, 121.1, 120.4, 117.6 (q, *J* = 4.0, $^3J_{CF}$), 107.7 (q, *J* = 4.0, $^3J_{CF}$), 102.5, 42.7, 20.2; HR-MS [ESI-MS(+)] calcd for $[M + H]^+$: $C_{17}H_{14}F_3N_2O$: 319.1058, found: 319.0146; IR (KBr): 2927, 2854, 1724, 1588, 1547, 1472, 1450, 1349, 1323, 1162, 1114, 1059, 956, 824, 787, 745, 659 cm^{-1}.

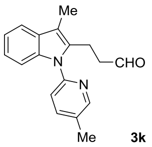
3k

3-(3-Methyl-1-(5-methylpyridin-2-yl)-1H-indol-2-yl)propanal (3k): The title compound was prepared from allylic alcohol **1k** (0.20 mmol, 55.6 mg) and was purified by column chromatography (10:1 = petroleum ether: ethyl acetate) to give a yellow solid; m.p. 61.7 - 62.7 °C; 37.0 mg, 67% yield; ^{1}H NMR (500 MHz, $CDCl_3$) δ 9.68 (s, 1H), 8.41 (s, 1H), 7.67 (dd, *J* = 8.0, 1.9 Hz, 1H), 7.54 – 7.52 (m, 1H), 7.32 (d, *J* = 8.0 Hz, 1H), 7.27 – 7.24 (m, 1H), 7.15 – 7.12 (m, 2H), 3.16 – 3.12 (m, 2H), 2.66 (t, *J* = 7.6 Hz, 2H), 2.41 (s, 3H), 2.31 (s, 3H); ^{13}C NMR (126 MHz, $CDCl_3$) δ 201.7, 149.8, 149.1, 139.0, 136.6, 134.9, 131.7, 129.2, 122.0, 120.3, 120.2, 118.3, 110.1, 109.9, 43.9, 18.1, 17.9, 8.8; HR-MS [ESI-MS(+)] calcd for $[M + H]^+$: $C_{18}H_{19}N_2O$: 279.1492, found: 279.1491; IR (KBr): 3045, 2922, 1867, 1715, 1484, 1457, 1387, 1361, 1316, 1221, 1135, 1025, 830, 740 cm^{-1}.

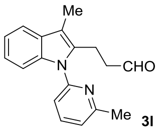
3l

3-(3-Methyl-1-(6-methylpyridin-2-yl)-1H-indol-2-yl)propanal (3l): The title compound was prepared from allylic alcohol **1l** (0.20 mmol, 55.6 mg) and was purified by column chromatography (10:1 = petroleum ether: ethyl acetate) to give a light yellow oil; 36.7 mg, 66% yield; ^{1}H NMR (400 MHz, $CDCl_3$) δ 9.77 (s, 1H), 7.79 (t, *J* = 7.7 Hz, 1H), 7.59 – 7.56 (m, 1H), 7.38 – 7.34 (m, 1H), 7.29 (d, *J* = 7.8 Hz, 1H), 7.20 – 7.16 (m, 3H), 3.23 – 3.18 (m, 2H), 2.79 (t, *J* = 7.6 Hz, 2H), 2.60 (s, 3H), 2.35 (s, 3H); ^{13}C NMR (101 MHz, $CDCl_3$) δ 201.7, 158.9, 150.7, 138.6, 136.36, 134.9, 129.3, 122.1, 121.3, 120.3, 118.4, 117.5, 110.3, 110.0, 44.1, 24.3, 18.0, 8.8; HR-MS [ESI-MS(+)] calcd for $[M + H]^+$: $C_{18}H_{19}N_2O$: 279.1492, found: 279.1491; IR (KBr): 2922, 1867, 1715, 1541, 1488, 1456, 1360, 1338, 1148, 989, 740 cm^{-1}.

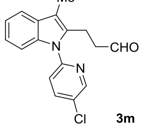
3m

3-(1-(5-Chloropyridin-2-yl)-3-methyl-1H-indol-2-yl)propanal (3m): The title compound was prepared from allylic alcohol **1m** (0.20 mmol, 59.6 mg) and was purified by column chromatography (10:1 = petroleum ether: ethyl acetate) to give

a yellow oil; 42.0 mg, 70% yield; ^{1}H NMR (400 MHz, $CDCl_3$) δ 9.76 (s, 1H), 8.57 (d, *J* = 2.6 Hz, 1H), 7.89 – 7.89 (m, 1H), 7.59 – 7.56 (m, 1H), 7.46 (d, *J* = 8.5 Hz, 1H), 7.35 – 7.32 (m, 1H), 7.22 – 7.18 (m, 2H), 3.21 – 3.16 (m, 2H), 2.75 (t, *J* = 7.6 Hz, 2H), 2.34 (s, 3H)； ^{13}C NMR (101 MHz, $CDCl_3$) δ 201.4, 149.7, 148.4, 138.2, 136.2, 134.9, 129.7, 129.5, 122.5, 121.2, 120.8, 118.6, 111.1, 109.7, 43.9, 17.9, 8.8; HR-MS [ESI-MS(+)] calcd for $[M + H]^+$: $C_{17}H_{16}ClN_2O$: 299.0946, found: 299.0944; IR (KBr): 3052, 2923, 2315, 1714, 1681, 1648, 1470, 1434, 1417, 1114, 1011, 742, 680 cm^{-1}.

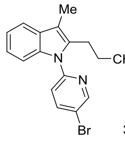
3n

3-(1-(5-Bromopyridin-2-yl)-3-methyl-1H-indol-2-yl)propanal (3n): The title compound was prepared from allylic alcohol **1n** (0.20 mmol, 68.4 mg) and was purified by column chromatography (10:1 = petroleum ether: ethyl acetate) to give a yellow oil; 45.0 mg, 66% yield; ^{1}H NMR (500 MHz, $CDCl_3$) δ 9.72 (s, 1H), 8.63 (d, *J* = 2.2 Hz, 1H), 7.97 – 7.95 (m, 1H), 7.55 – 7.52 (m, 1H), 7.36 (d, *J* = 8.5 Hz, 1H), 7.32 – 7.29 (m, 1H), 7.18 – 7.15 (m, 2H), 3.15 (t, *J* = 7.6 Hz, 2H), 2.71 (t, *J* = 7.6 Hz, 2H), 2.30 (s, 3H)； ^{13}C NMR (126 MHz, $CDCl_3$) δ 201.3, 150.6, 150.1, 141.0, 136.2, 134.9, 129.6, 122.5, 121.6, 120.8, 118.6, 118.0, 111.2, 109.8, 43.9, 17.9, 8.8; HR-MS [ESI-MS(+)] calcd for $[M + H]^+$: $C_{17}H_{16}BrN_2O$: 343.0441, found: 343.0438; IR (KBr): 2920, 1747, 1730, 1714, 1681, 1556, 1506, 1488, 1417, 1455, 742 cm^{-1}.

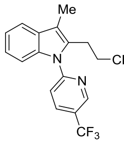
3o

3-(3-Methyl-1-(5-(trifluoromethyl)pyridin-2-yl)-1H-indol-2-yl)propanal (3o): The title compound was prepared from allylic alcohol **1o** (0.20 mmol, 66.4 mg) and was purified by column chromatography (20:1 = petroleum ether: ethyl acetate) to give a yellow solid; m.p. 64.9 - 69.2 ^{o}C; 45.3 mg, 68% yield; ^{1}H NMR (400 MHz, $CDCl_3$) δ 9.75 (s, 1H), 8.84 (s, 1H), 8.10 – 8.07 (m, 1H), 7.61 (d, *J* = 8.4 Hz, 1H), 7.57 – 7.53 (m, 1H), 7.43 – 7.38 (m, 1H), 7.22 – 7.17 (m, 2H), 3.23 – 3.18 (m, 2H), 2.80 – 2.74 (m, 2H), 2.32 (s, 3H); ^{13}C NMR (101 MHz, $CDCl_3$) δ 201.2, 154.2, 146.7 (q, *J* = 4.0, $^{3}J_{CF}$), 135.9, 135.7 (q, *J* = 4.0, $^{3}J_{CF}$), 135.0, 129.9, 124.0 (d, *J* = 34.3, $^{2}J_{CF}$), 123.5 (d, *J* = 272.7, $^{1}J_{CF}$), 122.8, 121.3, 119.5, 118.8, 112.2, 109.9, 44.0, 18.1, 8.8; HR-MS [ESI-MS(+)] calcd for $[M + H]^+$: $C_{18}H_{16}F_3N_2O$: 333.1209, found: 333.1203; IR (KBr): 2924, 1715, 1556, 1540, 1506, 1488, 1455, 1325, 1120, 1110, 740 cm^{-1}.

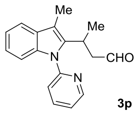
3p

3-(3-Methyl-1-(pyridin-2-yl)-1H-indol-2-yl)butanal (3p): The title compound was prepared from allylic alcohol **1p** (0.20 mmol, 55.6 mg) and was purified by column chromatography (10:1 = petroleum ether: ethyl acetate) to give a yellow oil; 34.5 mg, 62% yield; ^{1}H NMR (400 MHz, $CDCl_3$) δ 9.74 – 9.67 (m, 1H), 8.67 (dd, *J* = 4.9, 1.2 Hz, 1H), 7.96 – 7.91 (m, 1H), 7.60 – 7.56 (m, 1H), 7.48 (d, *J* = 7.9 Hz, 1H), 7.39 – 7.36 (m, 1H), 7.22 – 7.15 (m, 3H), 3.80 – 3.66 (m, 1H), 3.11 – 3.04 (m, 1H), 2.85 – 2.79 (m, 1H), 2.45 (s, 3H), 1.39 (d, *J* = 7.2 Hz, 3H)； ^{13}C NMR (101 MHz, $CDCl_3$) δ 201.8, 151.8, 149.7, 138.9, 138.4, 136.5, 129.7, 122.4, 122.2, 122.2, 120.3, 118.1, 109.9, 109.3, 49.9, 26.2, 19.9, 9.6; HR-MS [ESI-MS(+)] calcd for $[M + H]^+$: $C_{18}H_{19}N_2O$: 279.1492, found: 279.1489; IR (KBr): 3052, 2967, 2925, 2722, 1721, 1587, 1568, 1470, 1458, 1437, 1361, 1310, 1225, 1198, 1148, 1012, 779, 742 cm^{-1}.

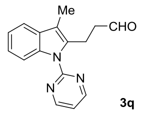
3q

3-(3-Methyl-1-(pyrimidin-2-yl)-1H-indol-2-yl)propanal (3q) [7]**:** The title compound was prepared from allylic alcohol **1q** (0.20 mmol, 53.0 mg) and was purified by column chromatography (10:1 = petroleum ether: ethyl acetate) to give a light yellow solid; 40.3 mg, 76% yield; ^{1}H NMR (500 MHz, $CDCl_3$) δ 9.81 (s, 1H), 8.71 (d, *J* = 4.8 Hz, 2H), 8.34 (d, *J* = 7.9 Hz, 1H), 7.50 (d, *J* = 7.9 Hz, 1H), 7.27 – 7.20 (m, 2H), 7.07 (t, *J* = 4.8 Hz, 1H), 3.45 – 3.39 (m, 2H), 2.84 (t, *J* = 7.5 Hz, 2H), 2.29 (s, 3H); ^{13}C NMR (126 MHz,

$CDCl_3$) δ 202.0, 158.1, 136.1, 135.0, 130.4, 123.2, 121.8, 118.1, 116.7, 114.3, 113.7, 44.3, 19.5, 8.8.

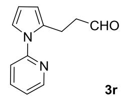

3r

3-(1-(Pyridin-2-yl)-1H-pyrrol-2-yl)propanal (3r): The title compound was prepared from allylic alcohol **1r** (0.20 mmol, 40.0 mg) and was purified by column chromatography (10:1 = petroleum ether: ethyl acetate) to give a brown oil; 14.0 mg, 35% yield; ^{1}H NMR (500 MHz, $CDCl_3$) δ 9.79 (s, 1H), 8.49 (d, *J* = 3.8 Hz, 1H), 7.81 – 7.78 (m, 1H), 7.31 (d, *J* = 8.1 Hz, 1H), 7.22 – 7.20 (m, 1H), 7.02 – 6.99 (m, 1H), 6.24 (t, *J* = 3.2 Hz, 1H), 6.08 (s, 1H), 3.21 (t, *J* = 7.5 Hz, 2H), 2.80 (t, *J* = 7.5 Hz, 2H); ^{13}C NMR (126 MHz, $CDCl_3$) δ 202.2, 152.8, 148.7, 138.5, 132.2, 121.2, 120.8, 117.1, 109.5, 109.4, 43.6, 20.7; HR-MS [ESI-MS(+)] calcd for $[M + H]^+$: $C_{12}H_{13}N_2O$: 201.1022, found: 201.1028; IR (KBr): 2925, 1747, 1731, 1715, 1697, 1541, 1506, 1488, 1455, 742 cm^{-1}.

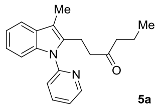

5a

1-(3-Methyl-1-(pyridin-2-yl)-1H-indol-2-yl)hexan-3-one (5a): The title compound was prepared from tertiary allylic alcohol **4a** (0.20 mmol, 61.2 mg) and was purified by column chromatography (20:1 = petroleum ether: ethyl acetate) to give a yellow oil; 52.0 mg, 85% yield; ^{1}H NMR (500 MHz, $CDCl_3$) δ 8.59 (d, *J* = 4.6 Hz, 1H), 7.87 – 7.84 (m, 1H), 7.53 (d, *J* = 6.8 Hz, 1H), 7.42 (d, *J* = 8.0 Hz, 1H), 7.31 (d, *J* = 8.2 Hz, 1H), 7.28 – 7.26 (m, 1H), 7.17 – 7.11 (m, 2H), 3.13 – 3.07 (m, 2H), 2.64 – 2.60 (m, 2H), 2.30 (s, 3H), 2.26 (t, *J* = 7.3 Hz, 2H), 1.55 – 1.50 (m, 2H), 0.84 (t, *J* = 7.4 Hz, 3H); ^{13}C NMR (126 MHz, $CDCl_3$) δ 210.3, 151.7, 149.6, 138.4, 136.5, 135.7, 129.4, 122.0, 121.8, 120.8, 120.3, 118.4, 110.3, 109.9, 44.7, 42.5, 19.5, 17.3, 13.7, 8.7; HR-MS [ESI-MS(+)] calcd for $[M + H]^+$: $C_{20}H_{23}N_2O$: 307.1805, found: 307.1789; IR (KBr): 2960, 2928, 1709, 1585, 1469, 1458, 1437, 1359, 1220, 1150, 1000, 783, 740 cm^{-1}.

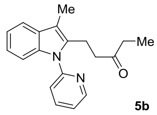

5b

1-(3-Methyl-1-(pyridin-2-yl)-1H-indol-2-yl)pentan-3-one (5b): The title compound was prepared from tertiary allylic alcohol **4b** (0.20 mmol, 58.4 mg) and was purified by column chromatography (20:1 = petroleum ether: ethyl acetate) to give a brown solid; m.p. 62.9 – 65.0 ^{o}C; 46.6 mg, 77% yield; ^{1}H NMR (400 MHz, $CDCl_3$) δ 8.65 – 8.63 (m, 1H), 7.92 – 7.88 (m, 1H), 7.59 – 7.57 (m, 1H), 7.47 (d, *J* = 8.0 Hz, 1H), 7.37 – 7.30 (m, 2H), 7.21 – 7.16 (m, 2H), 3.18 – 3.11 (m, 2H), 2.69 – 2.64 (m, 2H), 2.37 – 2.31 (m, 5H), 1.03 (t, *J* = 7.3 Hz, 3H); ^{13}C NMR (101 MHz, $CDCl_3$) δ 210.7, 151.7, 149.6, 138.4, 136.5, 135.7, 129.5, 122.0, 121.8, 120.8, 120.3, 118.4, 110.3, 109.9, 42.0, 35.9, 19.6, 8.7, 7.8; HR-MS [ESI-MS(+)] calcd for $[M + H]^+$: $C_{19}H_{21}N_2O$: 293.1648, found: 293.1649; IR (KBr): 2925, 1713, 1584, 1556, 1470, 1456, 1435, 1360, 1110, 780, 740 cm^{-1}.

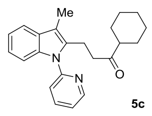

5c

1-Cyclohexyl-3-(3-methyl-1-(pyridin-2-yl)-1H-indol-2-yl)propan-1-one (5c): The title compound was prepared from tertiary allylic alcohol **4c** (0.20 mmol, 69.2 mg) and was purified by column chromatography (20:1 = petroleum ether: ethyl acetate) to give a yellow oil; 44.7 mg, 65% yield; ^{1}H NMR (500 MHz, $CDCl_3$) δ 8.59 (dd, *J* = 4.8, 1.2 Hz, 1H), 7.88 – 7.84 (m, 1H), 7.54 – 7.52 (m, 1H), 7.43 (d, *J* = 8.0 Hz, 1H), 7.32 – 7.26 (m, 2H), 7.16 – 7.11 (m, 2H), 3.09 – 3.05 (m, 2H), 2.69 – 2.65 (m, 2H), 2.30 (s, 3H), 2.21 (t, *J* = 10.4 Hz, 1H), 1.72 (d, *J* = 9.9 Hz, 4H), 1.64 (s, 1H), 1.28 – 1.16 (m, 5H); ^{13}C NMR (126 MHz, $CDCl_3$) δ 213.25, 151.7, 149.6, 138.3, 136.5, 135.9, 129.5, 121.9, 121.7, 120.8, 120.3, 118.3, 110.1, 109.9, 50.8, 40.4, 28.4, 25.9, 25.6, 19.5, 8.7; HR-MS [ESI-MS(+)] calcd for $[M + H]^+$: $C_{23}H_{27}N_2O$: 347.2118, found: 347.2112; IR (KBr): 2928, 2853, 1705, 1585, 1472, 1459, 1437, 1363, 1205, 1146, 1010, 779, 740 cm^{-1}.

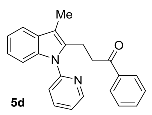

5d

3-(3-Methyl-1-(pyridin-2-yl)-1H-indol-2-yl)-1-phenylpropan-1-one (5d): The title compound was prepared from tertiary allylic alcohol **4d** (0.20 mmol, 68.0 mg) and was purified by column chromatography (20:1 = petroleum ether: ethyl acetate) to give a brown oil; 55.6 mg, 82% yield; ^{1}H NMR (400 MHz, $CDCl_3$) δ 8.64 – 8.62 (m, 1H), 7.94 – 7.88 (m, 3H), 7.62 – 7.54 (m, 2H), 7.53 – 7.43 (m, 3H), 7.40 – 7.36 (m, 1H), 7.32 – 7.29 (m, 1H), 7.24 – 7.18 (m, 2H), 3.32 (s, 4H), 2.39 (s, 3H); ^{13}C NMR (101 MHz, $CDCl_3$) δ 199.4, 151.7, 149.7, 138.4, 136.7, 136.5, 135.8, 133.1, 129.5, 128.6, 128.1, 122.1, 121.9, 120.9, 120.4, 118.4, 110.4, 109.9, 38.9, 20.2, 8.8; HR-MS [ESI-MS(+)] calcd for $[M + H]^+$: $C_{23}H_{21}N_2O$: 341.1648, found: 341.1644; IR (KBr): 3055, 2921, 1682, 1585, 1471, 1437, 1362, 1223, 1205, 1148, 972, 780, 739, 690 cm^{-1}.

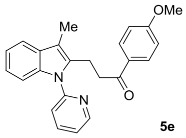

5e

1-(4-Methoxyphenyl)-3-(3-methyl-1-(pyridin-2-yl)-1H-indol-2-yl)propan-1-one (5e): The title compound was prepared from tertiary allylic alcohol **4e** (0.20 mmol, 74.0 mg) and was purified by column chromatography (20:1 = petroleum ether: ethyl acetate) to give a light yellow oil; 54.8 mg, 74% yield; ^{1}H NMR (400 MHz, $CDCl_3$) δ 8.62 (dd, *J* = 4.9, 1.8 Hz, 1H), 7.92 – 7.86 (m, 3H), 7.60 – 7.56 (m, 1H), 7.49 (d, *J* = 8.0 Hz, 1H), 7.38 – 7.36 (m, 1H), 7.32 – 7.28 (m, 1H), 7.22 – 7.16 (m, 2H), 6.93 – 6.88 (m, 2H), 3.87 (s, 3H), 3.31 – 3.26 (m, 2H), 3.25 – 3.20 (m, 2H), 2.37 (s, 3H); ^{13}C NMR (101 MHz, $CDCl_3$) δ 198.0, 163.4, 151.7, 149.7, 138.4, 136.5, 135.9, 130.3, 129.9, 129.5, 122.0, 121.8, 120.9, 120.4, 118.4, 113.7, 110.4, 109.9, 55.5, 38.5, 20.3, 8.8; HR-MS [ESI-MS(+)] calcd for $[M + H]^+$: $C_{24}H_{23}N_2O_2$: 371.1754, found: 371.1749; IR (KBr): 2925, 1710, 1654, 1468, 1437, 1357, 1257, 1167, 739 cm^{-1}.

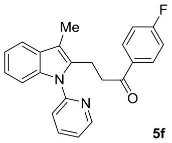

5f

1-(4-Fluorophenyl)-3-(3-methyl-1-(pyridin-2-yl)-1H-indol-2-yl)propan-1-one (5f): The title compound was prepared from tertiary allylic alcohol **4f** (0.20 mmol, 71.6 mg) and was purified by column chromatography (20:1 = petroleum ether: ethyl acetate) to give a brown solid; m.p. 78.2 – 79.8 ^{o}C; 59.1 mg, 83% yield; ^{1}H NMR (400 MHz, $CDCl_3$) δ 8.61 (dd, *J* = 4.8, 1.3 Hz, 1H), 7.97 – 7.88 (m, 3H), 7.60 – 7.58 (m, 1H), 7.50 (d, *J* = 8.0 Hz, 1H), 7.40 – 7.34 (m, 1H), 7.32 – 7.28 (m, 1H), 7.23 – 7.17 (m, 2H), 7.14 – 7.08 (m, 2H), 3.29 (s, 4H), 2.37 (s, 3H); ^{13}C NMR (101 MHz, $CDCl_3$) δ 197.8, 165.7 (d, *J* = 255.5, $^{1}J_{CF}$), 151.7, 149.7, 138.4, 136.5, 135.6, 133.2 (d, *J* = 3.0, $^{3}J_{CF}$), 130.7 (d, *J* = 10.1, $^{3}J_{CF}$), 129.5, 122.1, 121.9, 120.9, 120.4, 118.4, 115.6 (d, *J* = 21.2, $^{2}J_{CF}$), 110.5, 109.9, 38.8, 20.2, 8.8; HR-MS [ESI-MS(+)] calcd for $[M + H]^+$: $C_{23}H_{20}FN_2O$: 359.1554, found: 359.1551; IR (KBr): 3056, 2922, 1685, 1595, 1472, 1458, 1437, 1363, 1226, 1204, 1156, 977, 845, 779, 741 cm^{-1}.

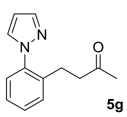

5g

4-(2-(1H-Pyrazol-1-yl)phenyl)butan-2-one (5g) [25]: The title compound was prepared from tertiary allylic alcohol **4g** (0.20 mmol, 42.8 mg), The reaction mixture was allowed to stir at 100 ℃ for 24 h and was purified by column chromatography (10:1 = petroleum ether: ethyl acetate) to give a light yellow oil; 36.0 mg, 84% yield; ^{1}H NMR (400 MHz, $CDCl_3$) δ 7.72 (d, *J* = 1.4 Hz, 1H), 7.63 (d, *J* = 2.2 Hz, 1H), 7.38 – 7.34 (m, 2H), 7.32 – 7.28 (m, 2H), 6.46 (t, *J* = 2.1 Hz, 1H), 2.84 (t, *J* = 7.7 Hz, 2H), 2.61 (t, *J* = 7.7 Hz, 2H), 2.07 (s, 3H); ^{13}C NMR (101 MHz, $CDCl_3$) δ 207.8, 140.4, 139.7, 137.3, 130.7, 130.6, 128.8, 127.1, 126.6, 106.5, 44.4, 29.8, 25.9;

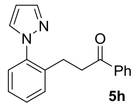

5h

3-(2-(1H-Pyrazol-1-yl)phenyl)-1-phenylpropan-1-one (5h) [26]: The title compound was prepared from tertiary allylic alcohol **4h** (0.20 mmol, 55.2 mg), The reaction mixture was allowed to stir at 100 ℃ for 24 h and was purified by column chromatography (10:1 = petroleum ether: ethyl acetate) to give a light yellow oil;

39.2 mg, 71% yield; ^{1}H NMR (400 MHz, $CDCl_3$) δ 7.93 – 7.89 (m, 2H), 7.73 (d, *J* = 1.6 Hz, 1H), 7.66 (d, *J* = 2.3 Hz, 1H), 7.55 (t, *J* = 7.4 Hz, 1H), 7.47 – 7.37 (m, 4H), 7.35 – 7.31 (m, 2H), 6.47 (t, *J* = 2.1 Hz, 1H), 3.20 – 3.15 (m, 2H), 3.04 – 2.99 (m, 2H); ^{13}C NMR (101 MHz, $CDCl_3$) δ 199.3, 140.5, 139.9, 137.5, 136.7, 133.0, 130.8, 130.7, 128.9, 128.5, 128.1, 127.1, 126.6, 106.6, 39.8, 26.6;

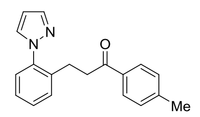

5i

3-(2-(1H-Pyrazol-1-yl)phenyl)-1-(p-tolyl)propan-1-one (5i): The title compound was prepared from tertiary allylic alcohol **4i** (0.20 mmol, 58.0 mg), The reaction mixture was allowed to stir at 100 ℃ for 24 h and was purified by column chromatography (10:1 = petroleum ether: ethyl acetate) to give a light yellow oil; 44.0 mg, 76% yield; ^{1}H NMR (500 MHz, $CDCl_3$) δ 7.78 (d, *J* = 7.9 Hz, 2H), 7.70 (s, 1H), 7.62 (s, 1H), 7.39 (d, *J* = 7.5 Hz, 1H), 7.37 – 7.33 (m, 1H), 7.30 (d, *J* = 4.2 Hz, 2H), 7.20 (d, *J* = 7.9 Hz, 2H), 6.43 (s, 1H), 3.13 – 3.09 (m, 2H), 3.00 – 2.95 (m, 2H), 2.38 (s, 3H); ^{13}C NMR (126 MHz, $CDCl_3$) δ 198.9, 143.8, 140.5, 139.9, 137.6, 134.2, 130.8, 130.7, 129.2, 128.8, 128.3, 127.1, 126.6, 106.5, 39.7, 26.7, 21.6; HR-MS [ESI-MS(+)] calcd for $[M + H]^+$: $C_{19}H_{19}N_2O$: 291.1492, found: 291.1492; IR (KBr): 3037, 2925, 1678, 1606, 1513, 1452, 1394, 1289, 1182, 1046, 976, 939, 821, 761 cm^{-1}.

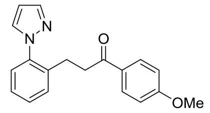

5j

3-(2-(1H-Pyrazol-1-yl)phenyl)-1-(4-methoxyphenyl)propan-1-one (5j): The title compound was prepared from tertiary allylic alcohol **4j** (0.20 mmol, 61.2 mg), The reaction mixture was allowed to stir at 100 ℃ for 24 h and was purified by column chromatography (5:1 = petroleum ether: ethyl acetate) to give a light yellow oil; 43.5 mg, 71% yield; ^{1}H NMR (500 MHz, $CDCl_3$) δ 7.86 (d, *J* = 8.7 Hz, 2H), 7.70 (s, 1H), 7.63 (d, *J* = 2.0 Hz, 1H), 7.40 (d, *J* = 7.6 Hz, 1H), 7.37 – 7.33 (m, 1H), 7.30 (d, *J* = 4.1 Hz, 2H), 6.88 (d, *J* = 8.7 Hz, 2H), 6.43 (s, 1H), 3.83 (s, 3H), 3.10 – 3.06 (m, 2H), 2.99 – 2.95 (m, 2H); ^{13}C NMR (126 MHz, $CDCl_3$) δ 197.8, 163.4, 140.4, 139.8, 137.7, 130.8, 130.8, 130.4, 129.8, 128.9, 127.0, 126.6, 113.7, 106.5, 55.5, 39.4, 26.8; HR-MS [ESI-MS(+)] calcd for $[M + H]^+$: $C_{19}H_{19}N_2O_2$: 307.1441, found: 307.1444; IR (KBr): 3124, 2939, 2845, 1672, 1595, 1508, 1445, 1312, 1260, 1211, 1161, 1022, 977, 938, 836, 768 cm^{-1}.

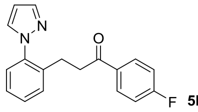

5k

3-(2-(1H-Pyrazol-1-yl)phenyl)-1-(4-fluorophenyl)propan-1-one (5k): The title compound was prepared from tertiary allylic alcohol **4k** (0.20 mmol, 58.8 mg), The reaction mixture was allowed to stir at 100 ℃ for 24 h and was purified by column chromatography (10:1 = petroleum ether: ethyl acetate) to give a light yellow oil; 50.5 mg, 86% yield; ^{1}H NMR (400 MHz, $CDCl_3$) δ 7.95 – 7.90 (m, 2H), 7.73 (d, *J* = 1.7 Hz, 1H), 7.66 (d, *J* = 2.3 Hz, 1H), 7.44 – 7.36 (m, 2H), 7.35 – 7.30 (m, 2H), 7.10 (t, *J* = 8.6 Hz, 2H), 6.47 (t, *J* = 2.1 Hz, 1H), 3.17 – 3.12 (m, 2H), 3.02 – 2.97 (m, 2H); ^{13}C NMR (101 MHz, $CDCl_3$) δ 197.7, 165.7 (d, *J* = 255.5, $^{1}J_{CF}$), 140.5, 139.8, 137.4, 133.1 (d, *J* = 3.0, $^{3}J_{CF}$), 130.9 (d, *J* = 6.1, $^{3}J_{CF}$), 130.8 (d, *J* = 2.0, $^{3}J_{CF}$), 128.9, 127.2, 126.6, 115.7, 115.5, 106.6, 39.8, 26.7; HR-MS [ESI-MS(+)] calcd for $[M + H]^+$: $C_{18}H_{16}FN_2O$: 295.1241, found: 295.1248; IR (KBr): 3123, 3064, 2915, 1683, 1594, 1506, 1399, 1296, 1205, 1155, 980, 939, 842, 767 cm^{-1}.

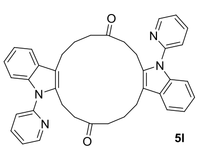

5l

5,16-Di(pyridin-2-yl)-5,6,7,9,10,11,16,17,18,20,21,22-dodecahydrocyclohexadeca[1,2-b:9,10-b']diindole-8,19-dione (5l): The title compound was prepared from tertiary allylic alcohol **4l** (0.20 mmol, 58.0 mg) and was purified by column chromatography (5:1 = petroleum ether: ethyl acetate) to give a white solid; m.p. 197.1 – 198.7 °C; 23.2 mg, 40% yield; ^{1}H NMR (500 MHz, $CDCl_3$) δ 8.64 – 8.58 (m, 2H), 7.89 – 7.86 (m, 2H), 7.53 – 7.51 (m, 2H), 7.41 (d, *J* = 7.9 Hz, 2H), 7.32 – 7.30 (m,

2H), 7.29 – 7.26 (m, 2H), 7.14 – 7.09 (m, 4H), 3.14 – 3.07 (m, 4H), 2.82 (t, *J* = 7.4 Hz, 4H), 2.60 – 2.54 (m, 4H), 2.41 (t, *J* = 6.4 Hz, 4H), 2.01 – 1.95 (m, 4H); ^{13}C NMR (126 MHz, $CDCl_3$) δ 209.5, 151.5, 149.8, 138.5, 136.9, 136.2, 128.5, 122.1, 121.0, 120.3, 119.5, 118.6, 114.0, 110.0, 42.8, 41.5, 24.5, 23.6, 19.2; HR-MS [ESI-MS(+)] calcd for $[M + H]^+$: $C_{38}H_{37}N_4O_2$: 581.2911, found: 581.2907; IR (KBr): 2924, 2854, 1709, 1585, 1472, 1459, 1437, 1369, 1321, 1225, 1147, 993, 782, 741 cm^{-1}.

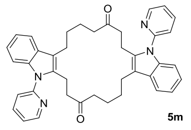

5,17-Di(pyridin-2-yl)-6,7,10,11,12,17,18,19,21,22,23,24-dodecahydrocyclooctadeca[1,2-b:10,11-b']diindole-8,20(5H,9H)-dione (5m): The title compound was prepared from tertiary allylic alcohol **4m** (0.20 mmol, 60.8 mg) and was purified by column chromatography (5:1 = petroleum ether: ethyl acetate) to give a white solid; m.p. 234.8 – 236.0 °C; 37.7 mg, 62% yield; ^{1}H NMR (400 MHz, $CDCl_3$) δ 8.62 (d, *J* = 4.5 Hz, 2H), 7.90 (t, *J* = 7.6 Hz, 2H), 7.59 – 7.51 (m, 2H), 7.46 (d, *J* = 8.0 Hz, 2H), 7.35 – 7.29 (m, 4H), 7.15 – 7.13 (m, 4H), 3.16 – 3.07 (m, 4H), 2.79 (t, *J* = 6.9 Hz, 4H), 2.58 – 2.49 (m, 4H), 2.34 (t, *J* = 7.0 Hz, 4H), 1.71 – 1.64 (m, 4H), 1.63 (d, *J* = 11.3 Hz, 4H); ^{13}C NMR (101 MHz, $CDCl_3$) δ 209.7, 151.6, 149.7, 138.4, 136.8, 136.1, 128.6, 122.0, 121.9, 120.9, 120.2, 118.6, 114.3, 109.9, 42.7, 42.4, 29.5, 24.0, 23.9, 18.8; HR-MS [ESI-MS(+)] calcd for $[M + H]^+$: $C_{40}H_{41}N_4O_2$: 609.3224, found: 609.3218; IR (KBr): 3050, 2920, 1867, 1790, 1747, 1714, 1681, 1555, 1540, 1471, 1455, 1434, 1361, 1338, 1147, 992, 777, 741 cm^{-1}.

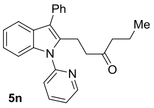

1-(3-Phenyl-1-(pyridin-2-yl)-1H-indol-2-yl)hexan-3-one (5n): brown oil; 11.5 mg, 31% yield; ^{1}H NMR (500 MHz, $CDCl_3$) δ 8.65 (dd, *J* = 4.7, 1.5 Hz, 1H), 7.95 – 7.91 (m, 1H), 7.62 – 7.56 (m, 1H), 7.55 – 7.50 (m, 3H), 7.47 (t, *J* = 7.6 Hz, 2H), 7.38 – 7.32 (m, 3H), 7.21 – 7.13 (m, 2H), 3.23 – 3.17 (m, 2H), 2.54 – 2.48 (m, 2H), 2.14 (t, *J* = 7.4 Hz, 2H), 1.47 – 1.39 (m, 2H), 0.78 (t, *J* = 7.4 Hz, 3H); ^{13}C NMR (126 MHz, $CDCl_3$) δ 209.7, 151.4, 149.8, 138.6, 136.7, 136.1, 134.8, 129.9, 128.6, 128.3, 126.6, 122.5, 122.4, 121.4, 121.0, 119.2, 117.6, 110.1, 44.4, 42.6, 19.7, 17.3, 13.6; HR-MS [ESI-MS(+)] calcd for $[M + H]^+$: $C_{25}H_{25}N_2O$: 369.1961, found: 369.1948; IR (KBr): 3054, 2959, 2926, 2872, 1711, 1588, 1470, 1458, 1436, 1370, 1189, 1148, 1023, 771, 743, 703 cm^{-1}.

1.5. Detail characterization for the sigmatropic rearrangement products 7

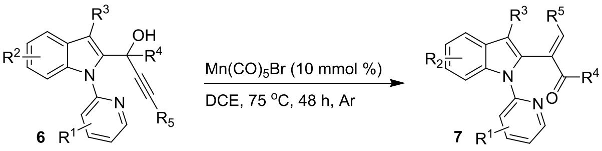

An oven-dried sealed tube charged propargyl alcohols **6** (0.20 mmol), $Mn(CO)_5Br$ (0.02 mmol, 10 mol %) and DCE (2.0 mL) under Ar atmosphere. The reaction mixture was then allowed to stir at 75 ℃ for 48 h. After the reaction mixture was cooled down, the corresponding reaction mixture was purified by flash chromatography on silica gel using ethyl acetate/petroleum ether (1: 10) as eluent to afford the desired products **7.**

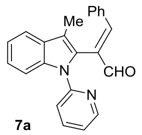

(*E*)-2-(3-Methyl-1-(pyridin-2-yl)-1H-indol-2-yl)-3-phenylacrylaldehyde (7a): The title compound was prepared from propargyl alcohol **6a** (0.20 mmol, 67.6 mg) and was purified by column chromatography (10: 1 = petroleum ether: ethyl acetate) to give a yellow solid; m.p. 135.1 – 137.6 °C; 49.3 mg, 73% yield; ^{1}H NMR (400 MHz, $CDCl_3$) δ 9.68 (s, 1H), 8.45 (d, *J* = 4.8 Hz, 1H), 7.72 – 7.68 (m, 2H), 7.65 (d, *J* = 7.7 Hz, 1H), 7.58 (s, 1H), 7.34 (d, *J* = 8.5 Hz, 3H), 7.31 – 7.28 (m, 2H), 7.25 (t, *J* = 7.5 Hz, 3H), 7.14 – 7.11 (m, 1H), 2.09 (s, 3H); ^{13}C NMR (101 MHz, $CDCl_3$) δ 192.7, 151.4, 150.5, 149.0, 137.8,

136.8, 134.4, 132.9, 130.6, 130.3, 129.5, 128.8, 128.5, 123.4, 121.0, 120.6, 119.5, 119.3, 114.1, 111.2, 9.3; HR-MS [ESI-MS(+)] calcd for $[M + H]^+$: $C_{23}H_{19}N_2O$: 339.1492, found: 339.1479; IR (KBr): 3447, 3055, 2920, 2852, 2713, 1686, 1587, 1470, 1448, 1437, 1363, 1321, 1224, 1149, 1083, 778, 740, 695 cm^{-1}.

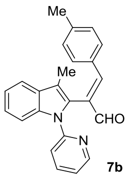

7b

(*E*)-2-(3-Methyl-1-(pyridin-2-yl)-1H-indol-2-yl)-3-(*p*-tolyl)acrylaldehyde (7b): The title compound was prepared from propargyl alcohol **6b** (0.20 mmol, 70.4 mg), the reaction mixture was then allowed to stir at 75 ℃ for 72 h and was purified by column chromatography (10: 1 = petroleum ether: ethyl acetate) to give a yellow oil; 59.1 mg, 84% yield; ^{1}H NMR (400 MHz, $CDCl_3$) δ 9.66 (s, 1H), 8.46 (d, *J* = 4.8 Hz, 1H), 7.74 – 7.64 (m, 3H), 7.56 (s, 1H), 7.31 – 7. 24 (m, 5H), 7.15 – 7.10 (m, 1H), 7.07 (d, *J* = 8.0 Hz, 2H), 2.33 (s, 3H), 2.10 (s, 3H); ^{13}C NMR (101 MHz, $CDCl_3$) δ 192.9, 151.4, 151.2, 149.0, 141.5, 137.8, 136.9, 131.9, 131.5, 130.5, 129.6, 129.5, 128.7, 123.3, 121.0, 120.6, 119.4, 119.3, 113.9, 111.3, 21.6, 9.3; HR-MS [ESI-MS(+)] calcd for $[M + H]^+$: $C_{24}H_{21}N_2O$: 353.1648, found: 353.1632; IR (KBr): 2922, 2854, 1681, 1661, 1558, 1470, 1455, 1437, 1363, 1331, 1224, 1184, 1149, 741 cm^{-1}.

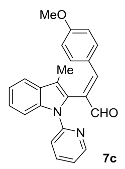

7c

(*E*)-3-(4-Methoxyphenyl)-2-(3-methyl-1-(pyridin-2-yl)-1H-indol-2-yl)acrylaldehyde (7c): The title compound was prepared from propargyl alcohol **6c** (0.20 mmol, 73.6 mg) and was purified by column chromatography (10: 1 = petroleum ether: ethyl acetate) to give a yellow solid; m.p. 151.9 – 154.2 ^{o}C; 51.5 mg, 70% yield; ^{1}H NMR (400 MHz, $CDCl_3$) δ 9.64 (s, 1H), 8.46 (d, *J* = 4.5 Hz, 1H), 7.74 – 7.65 (m, 3H), 7.53 (s, 1H), 7.32 – 7.24 (m, 5H), 7.13 (t, *J* = 6.1 Hz, 1H), 6.78 (d, *J* = 7.9 Hz, 2H), 3.79 (s, 3H), 2.13 (s, 3H); ^{13}C NMR (101 MHz, $CDCl_3$) δ 192.9, 161.8, 151.4, 151.3, 148.9, 137.8, 136.9, 132.5, 130.5, 129.5, 128.8, 126.8, 123.2, 121.1, 120.6, 119.4, 119.4, 114.4, 113.8, 111.4, 55.4, 9.3.; HR-MS [ESI-MS(+)] calcd for $[M + H]^+$: $C_{24}H_{21}N_2O_2$: 369.1598, found: 369.1563; IR (KBr): 3054, 2932, 2837, 2714, 1681, 1598, 1509, 1470, 1457, 1438, 1363, 1306, 1259, 1224, 1176, 1155, 1097, 1028, 831, 778, 741 cm^{-1}.

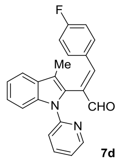

7d

(*E*)-3-(4-Fluorophenyl)-2-(3-methyl-1-(pyridin-2-yl)-1H-indol-2-yl)acrylaldehyde (7d): The title compound was prepared from propargyl alcohol **6d** (0.20 mmol, 71.2 mg) and was purified by column chromatography (10: 1 = petroleum ether: ethyl acetate) to give a yellow oil; 44.9 mg, 63% yield; ^{1}H NMR (400 MHz, $CDCl_3$) δ 9.66 (s, 1H), 8.43 (dd, *J* = 4.8, 1.1 Hz, 1H), 7.74 – 7.70 (m, 1H), 7.66 (t, *J* = 8.1 Hz, 2H), 7.53 (s, 1H), 7.34 – 7.24 (m, 5H), 7.15 – 7.12 (m, 1H), 6.94 (t, *J* = 8.7 Hz, 2H), 2.09 (s, 3H); ^{13}C NMR (101 MHz, $CDCl_3$) δ 192.5, 163.8 (d, *J* = 254.5, $^1J_{CF}$), 151.3, 149.1, 149.0, 137.9, 136.8, 132.6 (d, *J* = 2.0, $^3J_{CF}$), 132.5, 132.4, 130.5 (d, *J* = 4.0, $^3J_{CF}$), 129.4, 128.3, 123.5, 121.1, 120.7, 119.4 (d, *J* = 20.0, $^2J_{CF}$), 116.0 (d, *J* = 21.2, $^2J_{CF}$), 114.1, 111.1, 9.2; HR-MS [ESI-MS(+)] calcd for $[M + H]^+$: $C_{23}H_{18}FN_2O$: 357.1398, found: 357.1386; IR (KBr): 3057, 2921, 2855, 1687, 1597, 1506, 1470, 1457, 1438, 1363, 1234, 1157, 1081, 779 cm^{-1}.

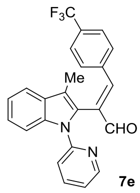

7e

(*E*)-2-(3-Methyl-1-(pyridin-2-yl)-1H-indol-2-yl)-3-(4-(trifluoromethyl)phenyl)acrylaldehyde (7e): The title compound was prepared from propargyl alcohol **6e** (0.20 mmol, 81.2 mg), the reaction mixture was then allowed to stir at 75 ℃ for 72 h and was purified by column chromatography (10: 1 = petroleum ether: ethyl acetate) to give a brown oil; 44.7 mg, 55% yield; ^{1}H NMR (500 MHz, $CDCl_3$) δ 9.66 (s, 1H), 8.38 (d, *J* = 4.4 Hz, 1H), 7.71 (t, *J* = 7.7 Hz, 1H), 7.62 (t, *J* = 7.6 Hz, 2H), 7.54 (s, 1H), 7.46 (d, *J* = 8.3 Hz, 2H), 7.41 (d, *J* = 8.2 Hz, 2H), 7.34 – 7.27 (m, 2H), 7.25 – 7.21 (m, 1H),

7.14 – 7.08 (m, 1H), 2.01 (s, 3H); ^{13}C NMR (126 MHz, $CDCl_3$) δ 192.2, 151.2, 149.0, 147.2, 138.0, 137.8, 136.7, 134.9, 131.5 (d, *J* = 33.1, $^2J_{CF}$), 130.2, 129.4, 128.1, 125.6 (q, *J* = 3.8, $^3J_{CF}$), 123.7 (d, *J* = 273.4, $^1J_{CF}$), 123.7, 121.1, 120.9, 119.6, 119.1, 114.4, 110.9, 9.2; HR-MS [ESI-MS(+)] calcd for $[M + H]^+$: $C_{24}H_{18}F_3N_2O$: 407.1366, found: 407.1351; IR (KBr): 3055, 2923, 1691, 1588, 1471, 1457, 1439, 1364, 1323, 1225, 1168, 1126, 1068, 1015, 899, 831, 778 cm^{-1}.

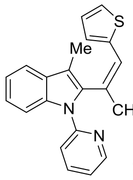

7f

(*E*)-2-(3-Methyl-1-(pyridin-2-yl)-1H-indol-2-yl)-3-(thiophen-2-yl)acrylaldehyde (7f): The title compound was prepared from propargyl alcohol **6f** (0.20 mmol, 68.8 mg) and was purified by column chromatography (10:1 = petroleum ether: ethyl acetate) to give a yellow solid; m.p. 179.3 – 182.1 ^{o}C; 51.6 mg, 75% yield; ^{1}H NMR (400 MHz, $CDCl_3$) δ 9.71 (s, 1H), 8.47 (dd, *J* = 4.9, 1.2 Hz, 1H), 7.82 (s, 1H), 7.74 (d, *J* = 8.2 Hz, 1H), 7.72 – 7.67 (m, 2H), 7.38 (d, *J* = 5.0 Hz, 1H), 7.34 – 7.27 (m, 4H), 7.15 – 7.11 (m, 1H), 7.01 – 6.99 (m, 1H), 2.21 (s, 3H); ^{13}C NMR (101 MHz, $CDCl_3$) δ 191.9, 151.2, 149.0, 142.9, 137.9, 137.1, 134.7, 133.6, 129.8, 129.7, 127.5, 127.3, 123.6, 121.1, 120.7, 119.5, 119.1, 115.3, 111.5, 9.3; HR-MS [ESI-MS(+)] calcd for $[M + H]^+$: $C_{21}H_{17}N_2OS$: 345.1056, found: 345.1044; IR (KBr): 3056, 2920, 2856, 1677, 1587, 1470, 1456, 1437, 1363, 1256, 1223, 1141, 1082, 1048, 857, 778, 741 cm^{-1}.

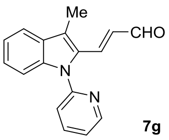

7g

(*E*)-3-(3-Methyl-1-(pyridin-2-yl)-1H-indol-2-yl)acrylaldehyde (7g): The title compound was prepared from propargyl alcohol **6g** (0.20 mmol, 52.4 mg) and was purified by column chromatography (10: 1 = petroleum ether: ethyl acetate) to give a brown oil; 27.8 mg, 38% yield; ^{1}H NMR (500 MHz, $CDCl_3$) δ 9.52 (d, *J* = 7.7 Hz, 1H), 8.70 (d, *J* = 4.7 Hz, 1H), 7.94 (t, *J* = 7.7 Hz, 1H), 7.69 (d, *J* = 8.0 Hz, 1H), 7.59 (d, *J* = 16.1 Hz, 1H), 7.40 (dd, *J* = 11.3, 6.5 Hz, 3H), 7.30 (t, *J* = 7.6 Hz, 1H), 7.22 (t, *J* = 7.5 Hz, 1H), 6.10 (dd, *J* = 16.1, 7.7 Hz, 1H), 2.58 (s, 3H); ^{13}C NMR (126 MHz, $CDCl_3$) δ 193.5, 151.1, 150.0, 140.8, 139.2, 138.7, 130.6, 129.5, 128.9, 128.3, 126.1, 122.8, 121.7, 121.5, 120.2, 110.9, 10.6; HR-MS [ESI-MS(+)] calcd for $[M + H]^+$: $C_{17}H_{15}N_2O$: 263.1179, found: 263.1177; IR (KBr): 2924, 1715, 1618, 1556, 1541, 1506, 1488, 1472, 1455, 1396, 1361, 1338, 1150, 740 cm^{-1}.

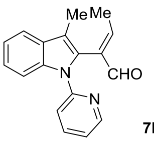

7h

(*E*)-2-(3-Methyl-1-(pyridin-2-yl)-1H-indol-2-yl)but-2-enal (7h): The title compound was prepared from propargyl alcohol **6h** (0.20 mmol, 55.2 mg) and was purified by column chromatography (10: 1 = petroleum ether: ethyl acetate) to give a brown oil; 19.4 mg, 35% yield; ^{1}H NMR (500 MHz, $CDCl_3$) δ 9.50 (s, 1H), 8.50 (d, *J* = 4.7 Hz, 1H), 7.75 – 7.72 (m, 1H), 7.62 – 7.61 (m, 2H), 7.26 – 7.15 (m, 4H), 7.01 (q, *J* = 7.0 Hz, 1H), 2.18 (s, 3H), 1.88 (d, *J* = 7.0 Hz, 3H); ^{13}C NMR (126 MHz, $CDCl_3$) δ 192.2, 153.4, 151.6, 149.1, 137.9, 137.5, 136.9, 129.3, 127.5, 123.2, 121.2, 120.6, 119.6, 119.1, 114.5, 111.0, 16.5, 9.4; HR-MS [ESI-MS(+)] calcd for $[M + H]^+$: $C_{18}H_{17}N_2O$: 277.1335, found: 277.1332; IR (KBr): 3054, 2921, 2854, 1689, 1585, 1468, 1361, 1225, 1152, 745 cm^{-1}.

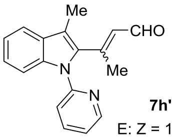

7h'
E: Z = 1: 3.3

3-(3-Methyl-1-(pyridin-2-yl)-1H-indol-2-yl)but-2-enal (7h'): The title compound was prepared from propargyl alcohol **6h** (0.20 mmol, 55.2 mg) and was purified by column chromatography (10: 1 = petroleum ether: ethyl acetate) to give a yellow oil; 34.3 mg, 62% yield (E: Z = 1: 3.3); ^{1}H NMR (500 MHz, $CDCl_3$) δ 10.11 (d, *J* = 7.9 Hz, 0.33H), 9.67 (d, *J* = 8.1 Hz, 1H), 8.62 (d, *J* = 5.0 Hz, 0.33H), 8.60 (d, *J* = 4.2 Hz, 1H), 7.82 (t, *J* = 7.3 Hz, 1.37H), 7.69 (d, *J* = 8.3 Hz, 1H), 7.63 (d, *J* = 7.9 Hz, 1H), 7.33 – 7.21 (m, 6H), 6.24 (d, *J* = 8.1 Hz, 1.33H), 2.42 (s, 1H), 2.30 (s, 3H), 2.10 (s, 1H), 1.89 (s, 3H); ^{13}C NMR (126 MHz, $CDCl_3$) δ 193.3, 190.8, 152.3, 151.6, 149.6, 138.5, 138.5, 137.5, 132.8, 131.9, 131.1, 129.4, 129.1, 124.8, 124.7, 121.6, 121.4, 121.4, 119.9, 119.7, 119.7,

119.2, 117.1, 111.2, 111.1, 24.8, 18.2, 10.3, 9.9; HR-MS [ESI-MS(+)] calcd for $[M + H]^+$: $C_{18}H_{17}N_2O$: 277.1335, found: 277.1333; IR (KBr): 3053, 2922, 2852, 1669, 1583, 1466, 1359, 1222, 1163, 1006, 746 cm^{-1}.

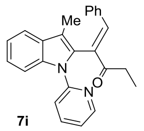

7i

(*E*)-2-(3-Methyl-1-(pyridin-2-yl)-1H-indol-2-yl)-1-phenylpent-1-en-3-one (7i): The title compound was prepared from propargyl alcohol **6i** (0.20 mmol, 73.2 mg) and was purified by column chromatography (10: 1 = petroleum ether: acetone) to give a orange solid; m.p. 165.6 – 166.7 ^oC; 48.3 mg, 66% yield; ^1H NMR (400 MHz, $CDCl_3$) δ 8.39 (dd, *J* = 4.9, 1.3 Hz, 1H), 7.72 – 7.66 (m, 2H), 7.61 – 7.57 (m, 1H), 7.33 – 7.21 (m, 4H), 7.19 – 7.11 (m, 4H), 7.06 – 7.01 (m, 1H), 6.70 (s, 1H), 2.62 – 2.41 (m, 2H), 2.26 (s, 3H), 1.03 (t, *J* = 7.3 Hz, 3H); ^{13}C NMR (101 MHz, $CDCl_3$) δ 202.3, 151.3, 148.9, 143.8, 139.4, 137.6, 137.0, 132.8, 129.5, 129.3, 129.2, 128.3, 127.7, 123.9, 120.9, 119.7, 119.5, 115.9, 111.4, 35.3, 9.5, 8.4; HR-MS [ESI-MS(+)] calcd for $[M + H]^+$: $C_{25}H_{23}N_2O$: 367.1805, found: 367.1792; IR (KBr): 3054, 2970, 2929, 1688, 1657, 1588, 1470, 1455, 1437, 1363, 1223, 1184, 1120, 1041, 779, 742, 694 cm^{-1}.

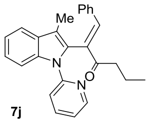

7j

(*E*)-2-(3-Methyl-1-(pyridin-2-yl)-1H-indol-2-yl)-1-phenylhex-1-en-3-one (7j): The title compound was prepared from propargyl alcohol **6j** (0.20 mmol, 76.0 mg) and was purified by column chromatography (10: 1 = petroleum ether: ethyl acetate) to give a orange solid; m.p. 131.9 – 134.7 ^oC; 36.5 mg, 48% yield; ^1H NMR (500 MHz, $CDCl_3$) δ 8.35 (d, *J* = 4.4 Hz, 1H), 7.66 (dd, *J* = 11.5, 8.0 Hz, 2H), 7.56 (t, *J* = 7.7 Hz, 1H), 7.29 – 7.18 (m, 4H), 7.14 (t, *J* = 7.4 Hz, 2H), 7.10 (d, *J* = 7.7 Hz, 2H), 7.02 – 6.98 (m, 1H), 6.65 (s, 1H), 2.56 – 2.47 (m, 1H), 2.38 – 2.32 (m, 1H), 2.22 (s, 3H), 1.60 – 1.51 (m, 2H), 0.82 (t, *J* = 7.4 Hz, 3H); ^{13}C NMR (126 MHz, $CDCl_3$) δ 201.6, 151.4, 148.8, 143.8, 139.4, 137.6, 136.9, 132.8, 129.4, 129.2, 129.2, 128.3, 127.8, 123.9, 120.8, 120.8, 119.7, 119.5, 116.0, 111.4, 44.2, 17.8, 13.8, 9.6; HR-MS [ESI-MS(+)] calcd for $[M + H]^+$: $C_{26}H_{25}N_2O$: 381.1961, found: 381.1948; IR (KBr): 2958, 2924, 1680, 1588, 1470, 1455, 1437, 1362, 1224, 1183, 1054, 779, 742 cm^{-1}.

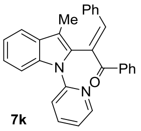

7k

(*E*)-2-(3-Methyl-1-(pyridin-2-yl)-1H-indol-2-yl)-1,3-diphenylprop-2-en-1-one (7k): The title compound was prepared from propargyl alcohol **6k** (0.20 mmol, 82.8 mg) and was purified by column chromatography (10: 1 = petroleum ether: ethyl acetate) to give a brown oil; 63.7 mg, 77% yield; ^1H NMR (400 MHz, $CDCl_3$) δ 8.36 (d, *J* = 3.2 Hz, 1H), 8.02 (d, *J* = 7.9 Hz, 2H), 7.74 – 7.71 (m, 1H), 7.61 – 7.54 (m, 2H), 7.51 – 7.47 (m, 2H), 7.42 (t, *J* = 7.5 Hz, 2H), 7.35 (d, *J* = 0.8 Hz, 1H), 7.26 (s, 2H), 7.24 – 7.19 (m, 5H), 6.99 – 6.96 (m, 1H), 2.17 (d, *J* = 0.9 Hz, 3H); ^{13}C NMR (101 MHz, $CDCl_3$) δ 191.2, 151.7, 148.6, 146.1, 139.7, 137.9, 137.6, 137.1, 133.1, 132.7, 129.6, 129.4, 129.2, 128.5, 128.4, 127.9, 126.2, 123.8, 120.7, 120.6, 120.2, 119.4, 115.8, 111.5, 9.8; HR-MS [ESI-MS(+)] calcd for $[M + H]^+$: $C_{29}H_{23}N_2O$: 415.1805, found: 415.1798; IR (KBr): 3055, 2922, 1656, 1588, 1470, 1448, 1437, 1380, 1363, 1266, 1223, 1017, 777, 743, 695 cm^{-1}.

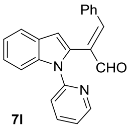

7l

(*E*)-3-Phenyl-2-(1-(pyridin-2-yl)-1H-indol-2-yl)acrylaldehyde (7l): The title compound was prepared from propargyl alcohol **6l** (0.20 mmol, 64.8 mg) and was purified by column chromatography (10: 1 = petroleum ether: ethyl acetate) to give a yellow solid; m.p. 127.8 – 130.4 ^oC; 34.3 mg, 53% yield; ^1H NMR (500 MHz, $CDCl_3$) δ 9.69 (s, 1H), 8.37 (dd, *J* = 4.8, 1.2 Hz, 1H), 7.66 (d, *J* = 7.7 Hz, 1H), 7.63 – 7.59 (m, 2H), 7.42 (s, 1H), 7.27 – 7.23 (m, 2H), 7.22 – 7.19 (m, 1H), 7.19 – 7.15 (m, 5H), 7.10 – 7.07 (m, 1H), 6.70 (s, 1H); ^{13}C NMR (126 MHz, $CDCl_3$) δ 192.6, 150.8, 150.3, 148.9, 137.7, 137.1, 133.9, 133.3,

131.4, 130.4, 130.4, 128.8, 128.8, 123.2, 121.3, 121.3, 121.2, 119.7, 111.3, 107.0; HR-MS [ESI-MS(+)] calcd for $[M + H]^+$: $C_{22}H_{17}N_2O$: 325.1335, found: 325.1342; IR (KBr): 3056, 2924, 2853, 1687, 1586, 1469, 1446, 1437, 1374, 1314, 1213, 1166, 1017, 775, 744 cm^{-1}.

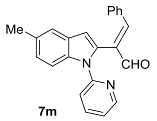

7m

(*E*)-2-(5-Methyl-1-(pyridin-2-yl)-1H-indol-2-yl)-3-phenylacrylaldehyde (7m): The title compound was prepared from propargyl alcohol **6m** (0.20 mmol, 67.6 mg) and was purified by column chromatography (10: 1 = petroleum ether: ethyl acetate) to give a brown oil; 35.2 mg, 52% yield; ^{1}H NMR (400 MHz, $CDCl_3$) δ 9.73 (s, 1H), 8.40 (dd, *J* = 4.9, 1.2 Hz, 1H), 7.67 – 7.62 (m, 1H), 7.54 (d, *J* = 8.5 Hz, 1H), 7.48 (s, 1H), 7.44 (s, 1H), 7.31 – 7.27 (m, 1H), 7.22 – 7.17 (m, 5H), 7.12 – 7.08 (m, 2H), 6.66 (s, 1H), 2.51 (s, 3H); ^{13}C NMR (101 MHz, $CDCl_3$) δ 192.6, 151.0, 149.9, 148.9, 137.6, 135.4, 134.0, 133.4, 131.4, 130.5, 130.4, 129.1, 128.5, 124.8, 121.1, 120.9, 119.5, 111.0, 106.7, 21.4; HR-MS [ESI-MS(+)] calcd for $[M + H]^+$: $C_{23}H_{19}N_2O$: 339.1492, found: 339.1487; IR (KBr): 3019, 2920, 2856, 1686, 1587, 1470, 1437, 1376, 1215, 1179, 1143, 1100, 1005, 771, 744, 691 cm^{-1}.

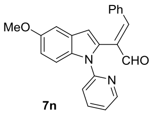

7n

(*E*)-2-(5-Methoxy-1-(pyridin-2-yl)-1H-indol-2-yl)-3-phenylacrylaldehyde (7n): The title compound was prepared from propargyl alcohol **6n** (0.20 mmol, 70.8 mg) and was purified by column chromatography (10: 1 = petroleum ether: ethyl acetate) to give a yellow oil; 42.5 mg, 60% yield; ^{1}H NMR (400 MHz, $CDCl_3$) δ 9.73 (s, 1H), 8.39 (dd, *J* = 4.8, 1.2 Hz, 1H), 7.66 – 7.62 (m, 1H), 7.55 (d, *J* = 9.0 Hz, 1H), 7.44 (s, 1H), 7.31 – 7.27 (m, 1H), 7.23 – 7.18 (m, 4H), 7.16 – 7.07 (m, 3H), 6.94 – 6.91 (m, 1H), 6.65 (s, 1H), 3.90 (s, 3H); ^{13}C NMR (101 MHz, $CDCl_3$) δ 192.6, 155.1, 150.9, 150.2, 148.9, 137.7, 133.9, 133.3, 132.2, 131.6, 130.4, 130.4, 129.3, 128.6, 121.1, 119.4, 113.4, 112.2, 106.8, 102.7, 55.8; HR-MS [ESI-MS(+)] calcd for $[M + H]^+$: $C_{23}H_{19}N_2O_2$: 355.1441, found: 355.1430; IR (KBr): 2923, 2843, 1687, 1588, 1470, 1488, 1403, 1338, 1204, 1175, 1147, 1031, 769 cm^{-1}.

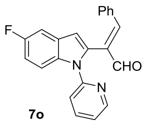

7o

(*E*)-2-(5-Fluoro-1-(pyridin-2-yl)-1H-indol-2-yl)-3-phenylacrylaldehyde (7o): The title compound was prepared from propargyl alcohol **6o** (0.20 mmol, 68.4 mg) and was purified by column chromatography (10: 1 = petroleum ether: ethyl acetate) to give a brown oil; 34.9 mg, 51% yield; ^{1}H NMR (400 MHz, $CDCl_3$) δ 9.72 (s, 1H), 8.40 (dd, *J* = 4.8, 1.1 Hz, 1H), 7.69 – 7.64 (m, 1H), 7.56 (dd, *J* = 9.0, 4.4 Hz, 1H), 7.46 (s, 1H), 7.34 – 7.29 (m, 2H), 7.24 – 7.12 (m, 6H), 7.04 – 6.98 m, 1H), 6.67 (s, 1H); ^{13}C NMR (101 MHz, $CDCl_3$) δ 192.5, 158.6 (d, *J* = 237.4, $^1J_{CF}$), 151.1, 150.7, 149.0, 137.8, 133.7, 133.1, 132.8, 130.7, 130.4, 129.1 (d, *J* = 2.0, $^3J_{CF}$), 128.6, 121.5, 119.6, 112.3 (d, *J* = 10.1, $^2J_{CF}$), 111.5 (d, *J* = 15.2, $^2J_{CF}$), 106.7 (d, *J* = 5.1, $^3J_{CF}$), 106.1, 105.9; HR-MS [ESI-MS(+)] calcd for $[M + H]^+$: $C_{22}H_{16}FN_2O$: 343.1241, found: 343.1233; IR (KBr): 3062, 2925, 2845, 1686, 1588, 1470, 1447, 1437, 1404, 1379, 1180, 1137, 1005, 773, 745, 691 cm^{-1}.

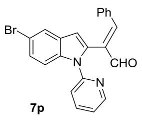

7p

(*E*)-2-(5-Bromo-1-(pyridin-2-yl)-1H-indol-2-yl)-3-phenylacrylaldehyde (7p): The title compound was prepared from propargyl alcohol **6p** (0.20 mmol, 80.4 mg) and was purified by column chromatography (10: 1 = petroleum ether: ethyl acetate) to give a yellow oil; 37.0 mg, 46% yield; ^{1}H NMR (400 MHz, $CDCl_3$) δ 9.70 (s, 1H), 8.40 (dd, *J* = 5.5, 1.8 Hz, 1H), 7.81 (d, *J* = 1.8 Hz, 1H), 7.69 – 7.65 (m, 1H), 7.50 (d, *J* = 8.8 Hz, 1H), 7.47 (s, 1H), 7.35 – 7.28 (m, 2H), 7.24 – 7.12 (m, 6H), 6.65 (s, 1H); ^{13}C NMR (101 MHz, $CDCl_3$) δ 192.4, 151.3, 150.4, 149.1, 137.9, 135.7, 133.6, 132.9, 132.5, 130.7, 130.4, 130.6, 128.6, 126.0, 123.6, 121.7, 119.7, 114.3, 112.9, 106.2; HR-MS [ESI-MS(+)] calcd for $[M + H]^+$: $C_{22}H_{16}BrN_2O$: 403.0441, found: 403.0429; IR (KBr): 3061, 2923, 2849, 1687, 1586, 1537, 1470, 1443, 1379, 1315, 1208, 1160, 1105, 1001, 869, 745, 692 cm^{-1}.

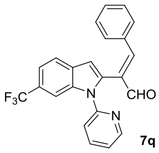

7q

(*E*)-3-Phenyl-2-(1-(pyridin-2-yl)-6-(trifluoromethyl)-1H-indol-2-yl)acrylaldehyde (7q): The title compound was prepared from propargyl alcohol **6q** (0.20 mmol, 78.4 mg) and was purified by column chromatography (10: 1 = petroleum ether: ethyl acetate) to give a yellow oil; 29.8 mg, 38% yield; ^{1}H NMR (400 MHz, $CDCl_3$) δ 9.71 (s, 1H), 8.47 – 8.42 (m, 1H), 7.88 (s, 1H), 7.77 (d, *J* = 8.3 Hz, 1H), 7.74 – 7.69 (m, 1H), 7.50 (s, 1H), 7.47 (d, *J* = 7.4 Hz, 1H), 7.35 – 7.30 (m, 1H), 7.26 – 7.16 (m, 6H), 6.76 (s, 1H); ^{13}C NMR (101 MHz, $CDCl_3$) δ 192.2, 151.7, 150.2, 149.2, 138.1, 136.1, 134.1, 133.5, 132.8, 130.9, 130.8, 130.4, 128.7, 125.2 (d, *J* = 32.3, $^2J_{CF}$), 125.1 (d, *J* = 237.4, $^1J_{CF}$), 122.0, 121.6, 119.8, 117.9 (q, *J* = 4.0, $^3J_{CF}$), 109.0 (q, *J* = 5.1, $^3J_{CF}$), 106.7; HR-MS [ESI-MS(+)] calcd for $[M + H]^+$: $C_{23}H_{16}F_3N_2O$: 393.1209, found: 393.1196; IR (KBr): 3063, 2924, 2852, 1688, 1588, 1470, 1439, 1345, 1331, 1301, 1264, 1231, 1162, 1116, 1058, 948, 830, 747 cm^{-1}.

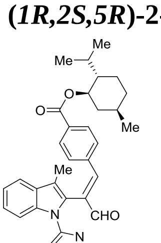

7r
d.r. = 1:1

(*1R,2S,5R*)-2-Isopropyl-5-methylcyclohexyl 4-((*E*)-2-(3-methyl-1-(pyridin-2-yl)-1H-indol-2-yl)-3-oxoprop-1-en-1-yl)benzoate (7r): The title compound was prepared from propargyl alcohol **6r** (0.20 mmol, 104.0 mg) and was purified by column chromatography (5: 1 = petroleum ether: ethyl acetate) to give a yellow oil; 76.0 mg, 73% yield; ^{1}H NMR (400 MHz, $CDCl_3$) δ 9.69 (d, *J* = 8.0 Hz, 1H), 8.45 – 8.42 (m, 1H), 7.94 – 7.89 (m, 2H), 7.77 – 7.73 (m, 1H), 7.69 – 7.63 (m, 2H), 7.60 (d, *J* = 5.9 Hz, 1H), 7.46 – 7.34 (m, 3H), 7.34 – 7.23 (m, 3H), 7.17 – 7.12 (m, 1H), 4.95 – 4.89 (m, 1H), 2.12 (d, *J* = 12.2 Hz, 1H), 2.06 (d, *J* = 12.2 Hz, 3H), 1.98 – 1.89 (m, 1H), 1.74 (d, *J* = 10.8 Hz, 2H), 1.59 – 1.50 (m, 2H), 1.16 – 1.04 (m, 2H), 0.96 – 0.91 (m, 7H), 0.80 (d, *J* = 6.9 Hz, 3H); ^{13}C NMR (101 MHz, $CDCl_3$) δ 192.4, 165.4, 151.3, 151.2, 149.0, 149.0, 148.1, 148.0, 138.5, 138.4, 138.0, 137.9, 136.8, 136.7, 134.6, 134.5, 132.0, 131.9, 123.0, 129.8, 129.8, 129.5, 129.4, 128.4, 128.3, 123.6, 121.1, 121.1, 120.8, 119.6, 119.1, 114.4, 114.3, 111.0, 110.9, 75.2, 47.2, 47.2, 40.9, 34.3, 31.5, 26.5, 26.4, 23.6, 23.6, 22.0, 20.8, 16.5, 16.4, 9.3, 9.2; HR-MS [ESI-MS(+)] calcd for $[M + H]^+$: $C_{34}H_{37}N_2O_3$: 521.2799, found: 521.2791; IR (KBr): 2923, 2862, 1712, 1694, 1588, 1471, 1456, 1438, 1364, 1274, 1109, 1081, 1016, 959, 773, 738 cm^{-1}.

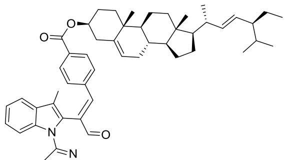

7s

(*3S,8S,9S,10R,13R,14S,17R*)-17-((*2R,5S,E*)-5-Ethyl-6-methylhept-3-en-2-yl)-10,13-dimethyl-2,3,4,7,8,9,10,11,12,13,14,15,16,17-tetradecahydro-1H-cyclopenta[a]phenanthren-3-yl 4-((*E*)-2-(3-methyl-1-(pyridin-2-yl)-1H-indol-2-yl)-3-oxoprop-1-en-1-yl)benzoate (7s): The title compound was prepared from propargyl alcohol **6s** (0.20 mmol, 155.0 mg) and was purified by column chromatography (5: 1 = petroleum ether: ethyl acetate) to give a yellow solid; m.p. 87.7 – 92.0 ^{o}C; 100.1 mg, 64% yield; ^{1}H NMR (400 MHz, $CDCl_3$) δ 9.70 (s, 1H), 8.43 (dd, *J* = 4.8, 1.2 Hz, 1H), 7.92 (d, *J* = 8.4 Hz, 2H), 7.75 – 7.71 (m, 1H), 7.69 – 7.63 (m, 2H), 7.59 (s, 1H), 7.39 (d, *J* = 8.4 Hz, 2H), 7.37 – 7.24 (m, 3H), 7.15 – 7.12 (m, 1H), 5.44 (d, *J* = 4.0 Hz, 1H), 5.24 – 5.18 (m, 1H), 5.10 – 5.05 (m, 1H), 4.90 – 4.81 (m, 1H), 2.46 (d, *J* = 7.4 Hz, 2H), 2.15 – 1.89 (m, 9H), 1.79 – 1.71 (m, 2H), 1.65 – 1.47 (m, 8H), 1.34 – 1.18 (m, 6H), 1.08 (d, *J* = 5.5 Hz, 7H), 0.90 (d, *J* = 6.2 Hz, 3H), 0.86 (t, *J* = 7.2 Hz, 6H), 0.75 (s, 3H); ^{13}C NMR (101 MHz, $CDCl_3$) δ 192.3, 165.2, 151.2, 149.0, 148.0, 139.5, 138.5, 138.4, 137.9, 136.8, 134.6, 131.9, 129.9, 129.8, 129.5, 129.3, 128.3, 123.6, 122.9, 121.1, 120.8, 119.6, 119.1, 114.4, 111.0, 74.9, 56.8, 56.0, 51.3, 50.1, 42.3, 40.5, 39.7, 38.2, 37.0, 36.7, 32.0, 31.9, 29.0, 27.9, 25.5, 24.4, 21.3, 21.2, 21.1, 19.4, 19.1, 12.3, 12.1, 9.3; HR-MS [ESI-MS(+)] calcd for $[M + H]^+$: $C_{53}H_{65}N_2O_3$: 777.4990, found: 777.4979; IR (KBr): 2954, 2867, 1716, 1694, 1588, 1470, 1457, 1438, 1365, 1272, 1226, 1110, 973, 775, 738 cm^{-1}.

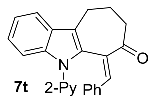

(*E*)-6-Benzylidene-5-(pyridin-2-yl)-6,8,9,10-tetrahydrocyclohepta[b]indol-7(5H)-one (7t): The title compound was prepared from propargyl alcohol **6t** (0.20 mmol, 72.8 mg) and was purified by column chromatography (10: 1 = petroleum ether: ethyl acetate) to give a light yellow solid; m.p. 187.1 – 192.9 ^oC; 29.1 mg, 40% yield; ^1H NMR (400 MHz, $CDCl_3$) δ 8.23 (dd, *J* = 4.9, 1.2 Hz, 1H), 7.71 – 7.65 (m, 1H), 7.55 – 7.50 (m, 1H), 7.47 – 7.42 (m, 1H), 7.32 (s, 1H), 7.27 – 7.23 (m, 2H), 7.14 (t, *J* = 7.4 Hz, 1H), 7.02 (t, *J* = 7.7 Hz, 2H), 6.97 – 6.92 (m, 1H), 6.75 – 6.69 (m, 3H), 3.32 – 3.16 (m, 2H), 3.08 – 2.97 (m, 1H), 2.86 – 2.75 (m, 1H), 2.41 – 2.25 (m, 2H); ^{13}C NMR (101 MHz, $CDCl_3$) δ 203.4, 150.3, 148.4, 137.7, 137.6, 136.8, 134.7, 131.1, 129.7, 129.4, 128.7, 128.7, 127.9, 123.9, 121.0, 120.6, 120.4, 118.7, 117.4, 111.4, 40.6, 23.8, 22.0; HR-MS [ESI-MS(+)] calcd for $[M + H]^+$: $C_{25}H_{21}N_2O$: 365.1648, found: 365.1660; IR (KBr): 3045, 2927, 1688, 1587, 1468, 1438, 1372, 1246, 1127, 930, 745, 694 cm^{-1}.

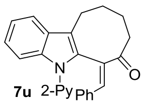

(*E*)-6-Benzylidene-5-(pyridin-2-yl)-5,6,8,9,10,11-hexahydro-7H-cycloocta[b]indol-7-one (7u): The title compound was prepared from propargyl alcohol **6u** (0.20 mmol, 75.6 mg) and was purified by column chromatography (10: 1 = petroleum ether: ethyl acetate) to give a yellow solid; m.p. 180.0 – 183.4 ^oC; 25.7 mg, 34% yield; ^1H NMR (400 MHz, $CDCl_3$) δ 8.28 (dd, *J* = 4.8, 1.3 Hz, 1H), 7.74 – 7.68 (m, 1H), 7.61 (s, 1H), 7.50 – 7.41 (m, 2H), 7.30 – 7.24 (m, 2H), 7.16 (t, *J* = 7.4 Hz, 1H), 7.07 – 6.97 (m, 3H), 6.78 (d, *J* = 7.6 Hz, 2H), 6.71 (d, *J* = 8.0 Hz, 1H), 3.30 – 3.17 (m, 2H), 2.86 – 2.80 (m, 1H), 2.58 – 2.46 (m, 1H), 2.29 – 2.09 (m, 2H), 2.03 – 1.90 (m, 1H), 1.81 – 1.73 (m, 1H); ^{13}C NMR (101 MHz, $CDCl_3$) δ 203.5, 150.4, 148.7, 139.9, 137.0, 136.9, 134.7, 131.3, 130.2, 129.9, 129.3, 128.4, 128.1, 123.4, 120.8, 120.7, 120.2, 118.8, 118.1, 111.0, 39.0, 28.8, 26.1, 24.2; HR-MS [ESI-MS(+)] calcd for $[M + H]^+$: $C_{26}H_{23}N_2O$: 379.1805, found: 379.1793; IR (KBr): 3055, 2925, 2858, 1681, 1588, 1470, 1438, 1369, 1317, 1223, 1162, 1093, 1016, 748, 693 cm^{-1}.

1.6. Post-synthetic applications of the 3a, 3q and 7a

1) Removal of the pyrimidyl group of 3q [7, 8]

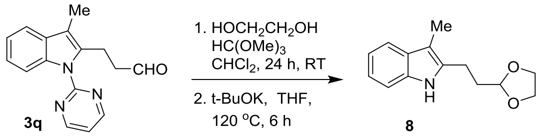

A suspension of **3q** (79.5 mg, 0.3 mmol), ethylene glycol (186.0 mg, 3 mmol), *p*-TsOH (10.3 mg, 20 mol %) and $HC(OMe)_3$ (127.2 mg, 1.2 mmol) in CH_2Cl_2 for 24 h at room temperature, the solvent was removed *in vacuo*. the crude product was used for the next step without purification. To an oven-dried sealed tube charged with crude product and *t*-BuOK (202 mg, 1.8 mmol) followed by addition of anhydrous THF (3 mL) through syringe. After stirring at 120 ^oC for 6 h, saturated NH_4Cl (5 mL) was added and resulting mixture was extracted with EtOAc (3 × 5 mL), and the combined organic layers were dried over Na_2SO_4, the solvent was removed in vacuo and the resultant residue was purified by flash chromatography on silica gel using ethyl acetate/petroleum ether = 1: 5 as eluent to afford the desired product **8**.

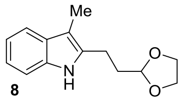

2-(2-(1,3-Dioxolan-2-yl)ethyl)-3-methyl-1H-indole (8): pale yellow oil, 52.0 mg, 75% yield; ^1H NMR (400 MHz, $CDCl_3$) δ 8.18 (s, 1H), 7.53 (d, *J* = 7.5 Hz, 1H), 7.31 (d, *J* = 7.4 Hz, 1H), 7.19 – 7.11 (m, 2H), 4.97 (t, *J* = 4.4 Hz, 1H), 4.10 – 4.06 (m, 2H), 3.96 – 3.93 (m, 2H), 2.92 (t, *J* = 7.4 Hz, 2H), 2.30 (s, 3H), 2.09 – 2.05 (m, 2H); ^{13}C NMR (101 MHz, $CDCl_3$) δ 135.3, 134.3, 129.3, 121.0, 118.9, 118.1, 110.3, 106.9, 103.7,

65.1, 33.2, 20.1, 8.4; HR-MS (ESI) calcd for $[M + H]^+$: $C_{14}H_{18}NO_2$: 232.1332, found: 232.1330; IR (KBr): 3398, 3050, 2926, 2885, 1580, 1463, 1407, 1333, 1240, 1141, 1026, 943, 892, 743 cm^{-1}.

2) Alkynylayion of aldehyde 3a with α-diazo-phosphonate [9]

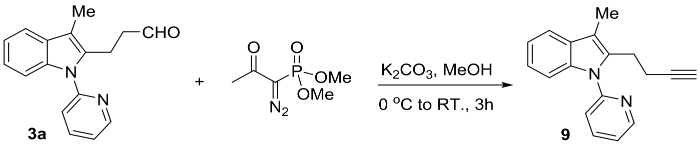

To a solution of aldehyde **3a** (0.079 g, 0.3 mmol) in anhydrous MeOH (1 mL) was added a solution of Ohira-Bestmann Reagent (0.144 g, 0.75 mmol) in MeOH (1 mL) and K_2CO_3 (0.124 g, 0.9 mmol) at 0 ℃. The resulting mixture was stirred at room temperature for 3 h before quenched with an aqueous solution of NH_4Cl. The aqueous phase was extracted with ethyl acetate, and the combined organic layers were washed with brine, dried over anhydrous Na_2SO_4, filtered, and concentrated in vacuo to give an oil. Purification by column chromatography (petroleum ether /ethyl acetate = 10: 1) afforded alkyne **9**.

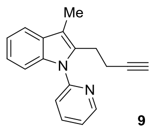

2-(But-3-yn-1-yl)-3-methyl-1-(pyridin-2-yl)-1H-indole (9): colorless oil, 55.0 mg, 71% yield ; ^{1}H NMR (400 MHz, CDCl3) δ 8.71 – 8.66 (m, 1H), 7.93 – 7.88 (m, 1H), 7.63 – 7.61 (m, 1H), 7.51 (d, J = 8.0 Hz, 1H), 7.40 – 7.36 (m, 1H), 7.35 – 7.31 (m, 1H), 7.24 – 7.19 (m, 2H), 3.22 (t, J = 7.7 Hz, 2H), 2.46 – 2.42 (m, 5H), 1.96 - 194 (m, 1H); ^{13}C NMR (101 MHz, CDCl3) δ 151.6, 149.7, 138.3, 136.5, 135.1, 129.4, 122.2, 121.8, 120.8, 120.4, 118.6, 111.1, 109.9, 83.8, 68.8, 24.6, 19.1, 8.9; HR-MS (ESI) calcd for $[M + H]^+$: $C_{18}H_{17}N_2$: 261.1386, found: 261.1394; IR (KBr): 3292, 3052, 2921, 2120, 1585, 1472, 1458, 1437, 1363, 1316, 1224, 1181, 1148, 780, 742, 639 cm^{-1}.

3) Cyanation of aldehyde 7a with acetonitrile [10]

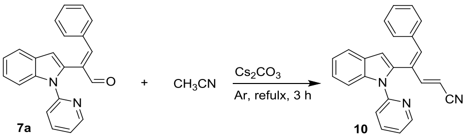

An oven-dried sealed tube charged **7a** (64.8 mg, 0.2 mmol), Cs_2CO_3 (195.0 mg, 0.6 mmol) and CH_3CN (2.0 mL) under Ar atmosphere. The reaction mixture was then allowed to refulx for 3 h. After the reaction mixture was cooled down, the corresponding reaction mixture was removed in vacuo and the resultant residue was purified by flash chromatography on silica gel using ethyl acetate/petroleum (1: 20) as eluent to afford the desired products **10**.

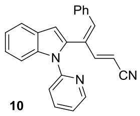

(2*E*,4*Z*)-5-Phenyl-4-(1-(pyridin-2-yl)-1H-indol-2-yl)penta-2,4-dienenitrile (10): brown solid, m.p. 144.2 – 146.8 °C; 25.7 mg, 37% yield ; ^{1}H NMR (500 MHz, $CDCl_3$) δ 8.40 (dd, *J* = 4.8, 1.3 Hz, 1H), 7.70 – 7.68(m, 1H), 7.61 – 7.55 (m, 2H), 7.32 (d, *J* = 16.0 Hz, 1H), 7.28 – 7.22 (m, 3H), 7.16 (d, *J* = 7.3 Hz, 1H), 7.12 – 7.07 (m, 3H), 6.91 (d, *J* = 8.0 Hz, 1H), 6.88 (d, *J* = 7.5 Hz, 2H), 6.85 (s, 1H), 6.67 (s, 1H), 5.41 (d, *J* = 16.0 Hz, 1H); ^{13}C NMR (126 MHz, $CDCl_3$) δ 153.6, 150.3, 149.0, 141.8, 137.7, 137.0, 134.6, 131.8, 129.1, 129.3, 128.4, 128.4, 123.5, 121.6, 121.5, 121.1, 119.7, 118.6, 111.7, 106.7, 98.5; HR-MS (ESI) calcd for $[M + H]^+$: $C_{24}H_{18}N_3$: 348.1495, found: 348.1502; IR (KBr): 3055, 2923, 2212, 1586, 1468, 1444, 1395, 1314, 965, 780, 744 cm^{-1}.

4) Arylvinylation of aldehyde 7a with diethyl benzylphosphonate [11]

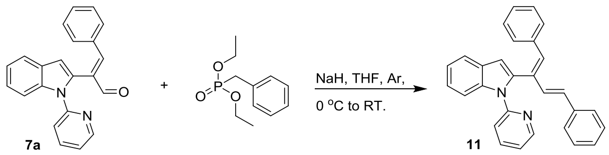

A 50 mL round bottom flask equipped with a magnetic stir bar was charged with diethyl benzylphosphonate (1.1 equiv) and dry THF (0.3 M). The solution was cooled to 0 ℃ with an ice bath followed by NaH addition (1.2 equiv). After stirring for 30 min at 0 ℃ the aldehyde **7a** (0.28 mmol) was slowly added. The reaction was allowed to warm to room temperature and stirred until judged complete by TLC analysis (12 h). The reaction mixture was then cooled to 0 ℃ and quenched with aqueous ammonium chloride. The solution was extracted with ethyl acetate. The combined organic phases were washed with brine, dried over Na_2SO_4 and concentrated under reduced pressure. The crude product was purified by flash chromatography on silica gel using ethyl acetate/petroleum (1: 20) as eluent to afford the desired products **11.**

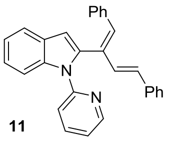

2-((1*Z*,3*E*)-1,4-Diphenylbuta-1,3-dien-2-yl)-1-(pyridin-2-yl)-1H-indole (11): brown solid, m.p. 171.3 – 174.4 °C; 59.0 mg, 67% yield; ^{1}H NMR (400 MHz, $CDCl_3$) δ 8.44 – 8.38 (m, 1H), 7.86 – 7.82 (m, 1H), 7.77 (d, *J* = 6.8 Hz, 1H), 7.61 – 7.57 (m, 1H), 7.44 – 7.41 (m, 2H), 7.36 – 7.32 (m, 4H), 7.29 – 7.26 (m, 1H), 7.20 – 7.07 (m, 6H), 6.94 – 6.90 (m, 2H), 6.81 (s, 1H), 6.78 (d, *J* = 0.7 Hz, 1H), 6.65 (d, *J* = 15.9 Hz, 1H); ^{13}C NMR (101 MHz, $CDCl_3$) δ 151.1, 148.6, 137.4, 137.3, 137.0, 136.2, 135.2, 134.6, 132.8, 132.0, 131.8, 129.0, 128.8, 128.6, 128.2, 127.7, 127.6, 126.7, 122.9, 121.2, 121.1, 120.8, 119.8, 112.4, 106.4; HR-MS (ESI) calcd for $[M + H]^+$: $C_{29}H_{23}N_2$: 399.1856, found: 399.1857; IR (KBr): 3054, 2956, 1585, 1467, 1452, 1443, 1436, 1390, 1343, 1312, 1208, 1150, 1017, 962, 743, 692 cm^{-1}.

5) Reduction of carbon-carbon double bonds and carbonyl group from 7a [10]

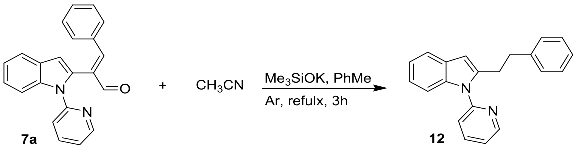

An oven-dried sealed tube charged **7a** (105.0 mg, 0.32 mmol), CH_3CN (65.6 mg, 1.6 mmol), Me_3SiOK (122.9 mg, 0.96 mmol) and PhMe (1.0 mL) under Ar atmosphere. The reaction mixture was then allowed to refulx for 3 h. After the reaction mixture was cooled down, the corresponding reaction mixture was removed in vacuo and the resultant residue was purified by flash chromatography on silica gel using ethyl acetate/petroleum (1: 20) as eluent to afford the desired products **12**.

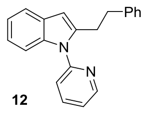

2-Phenethyl-1-(pyridin-2-yl)-1H-indole (12) [12]: yellow oil, 39.1 mg, 44% yield; ^{1}H NMR (400 MHz, $CDCl_3$) δ 8.72 – 8.68 (m, 1H), 7.92 – 7.88 (m, 1H), 7.65 – 7.59 (m, 1H), 7.44 (d, *J* = 8.0 Hz, 1H), 7.39 – 7.33 (m, 2H), 7.30 – 7.26 (m, 2H), 7.23 – 7.13 (m, 5H), 6.53 (s, 1H), 3.23 – 3.16 (m, 2H), 2.99 – 2.91 (m, 2H); ^{13}C NMR (101 MHz, $CDCl_3$) δ 151.5, 149.7, 141.5, 140.8, 138.3, 137.3, 128.6, 128.4, 126.0, 122.1, 121.8, 121.1, 120.7, 120.1, 110.1, 102.5, 35.3, 29.7; HR-MS (ESI) calcd for $[M + H]^+$: $C_{21}H_{19}N_2$: 299.1543, found: 299.1550;

6) Regioselective Reduction of α, β-unsaturated aldehyde 7a by $NaBH_4$ [13]

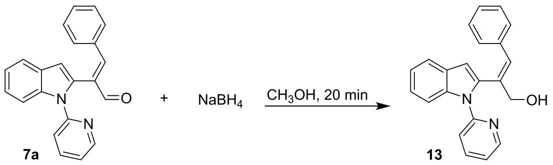

A 50 mL round bottom flask equipped with a magnetic stir bar was charged with **7a** (64.8 mg, 0.2 mmol) and CH_3OH (2 mL). The solution was cooled to 0 ℃ with an ice bath followed by $NaBH_4$ addition (15.0 mg, 2.0 equiv). After stirring for 3 h, the reaction mixture was quenched with water and extracted with ethyl acetate. The combined organic phases were dried over Na_2SO_4 and concentrated under reduced pressure. The crude product was purified by flash chromatography on silica gel using ethyl acetate/petroleum (1: 6) as eluent to afford the desired products **13**.

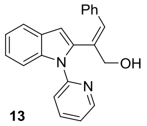

(*E*)-3-Phenyl-2-(1-(pyridin-2-yl)-1H-indol-2-yl)prop-2-en-1-ol (13): white solid, m.p. 110.9 – 115.5 °C; 59.0 mg, 82% yield; ^{1}H NMR (400 MHz, $CDCl_3$) δ 8.42 (dd, *J* = 4.9, 1.2 Hz, 1H), 7.75 – 7.73 (m, 1H), 7.57 – 7.53 (m, 1H), 7.44 – 7.37 (m, 1H), 7.27 – 7.20 (m, 2H), 7.15 – 7.10 (m, 1H), 7.07 (t, *J* = 7.3 Hz, 1H), 6.99 (t, *J* = 7.4 Hz, 2H), 6.93 (d, *J* = 8.0 Hz, 1H), 6.82 (s, 1H), 6.67 (d, *J* = 7.4 Hz, 2H), 6.61 (s, 1H), 5.23 (s, 1H), 4.64 (s, 2H); ^{13}C NMR (101 MHz, $CDCl_3$) δ 149.9, 148.1, 137.9, 136.9, 136.4, 135.7, 132.3, 131.6, 129.3, 128.3, 127.9, 127.3, 122.7, 121.4, 121.3, 121.2, 120.2, 110.2, 104.5, 70.3; HR-MS (ESI) calcd for $[M + H]^+$: $C_{22}H_{19}N_2O$: 327.1494, found: 327.1492; IR (KBr): 3748, 3055, 2920, 2854, 1589, 1470, 1452, 1438, 1386, 1345, 1313, 1211, 1151, 1028, 779, 743, 695 cm^{-1}.

7) Coupling-cyclization of 7a with phenylhydrazine [14]

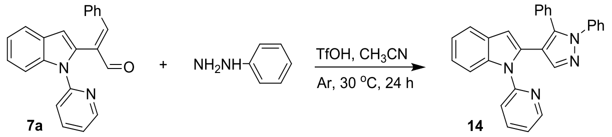

Into the reaction mixture of **7a** (64.8 mg, 0.2 mmol) and acetonitrile (1 mL) were added phenylhydrazine (21.6 mg, 0.2 mmol). The reaction mixture then was stirred for 10 min. at 30 °C followed by TfOH (30.0 g, 0.4 mmol) were added. The mixture was stirred at the same temperature till completion of the reaction which was monitored by TLC (24 h). After completion of the reaction, the crude was quenched by sat. solution of sodiumbicarbonate and extracted with ethylacetate. The organic layer was separated and dried with anhydrous Na_2SO_4 and then concentrated under reduced pressure to get crude compound. The crude product was purified by flash chromatography on silica gel using ethyl acetate/petroleum (1: 10) as eluent to afford the desired products **14.**

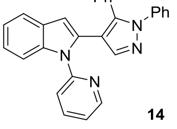

2-(1,5-Diphenyl-1H-pyrazol-4-yl)-1-(pyridin-2-yl)-1H-indole (14): brown solid; m.p. 63.7 – 68.6 °C; 37.0 mg, 42% yield; ^{1}H NMR (400 MHz, $CDCl_3$) δ 8.38 (dd, *J* = 4.6, 1.4 Hz, 1H), 7.71 (s, 1H), 7.64 – 7.62 (m, 1H), 7.58 – 7.54 (m, 1H), 7.52 – 7.50 (m, 1H), 7.30 – 7.26 (m, 3H), 7.24 – 7.16 (m, 5H), 7.14 – 7.07 (m, 3H), 6.90 (d, *J* = 8.0 Hz, 1H), 6.83 (d, *J* = 7.4 Hz, 2H), 6.64 (s, 1H); ^{13}C NMR (101 MHz, $CDCl_3$) δ 151.7, 151.3, 148.9, 141.1, 140.5, 139.7, 137.5, 131.2, 129.7, 129.4, 128.7, 128.7, 128.3, 128.2, 127.4, 122.6, 121.2, 121.1, 121.0, 120.3, 114.3, 111.2, 105.8; HR-MS (ESI) calcd for $[M + H]^+$: $C_{28}H_{21}N_4$: 413.1761, found: 413.1772; IR (KBr): 3055, 2921, 2856, 1588, 1499, 1468, 1454, 1435, 1379, 1315, 1208, 1152, 1069, 1023, 962, 772, 697 cm^{-1}.

1.7. Control experiments for the mechanism studies

1) H/D exchange experiment of α-(2-indolyl)alcohol 1a

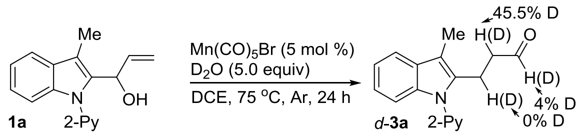

An oven-dried sealed tube charged allylicalcohol **1a** (26.4 mg, 0.10 mmol), Mn(CO)$_5$Br (2.0 mg, 5 mol %)，D_2O (10.0 mg, 0.50 mmol) and DCE (1.0 mL) under Ar atmosphere. The reaction mixture was then allowed to stir at 75 ℃ for 24 h. After the reaction mixture was cooled down, the corresponding reaction mixture was purified by flash chromatography on silica gel using ethyl acetate/petroleum ether (1: 10) as eluent to afford the desired products *d*-**3a** in which 45.5 D% was incorporated into the α-position of aldehyde *d*-**3a** (see **Supplementary Fig. 1**).

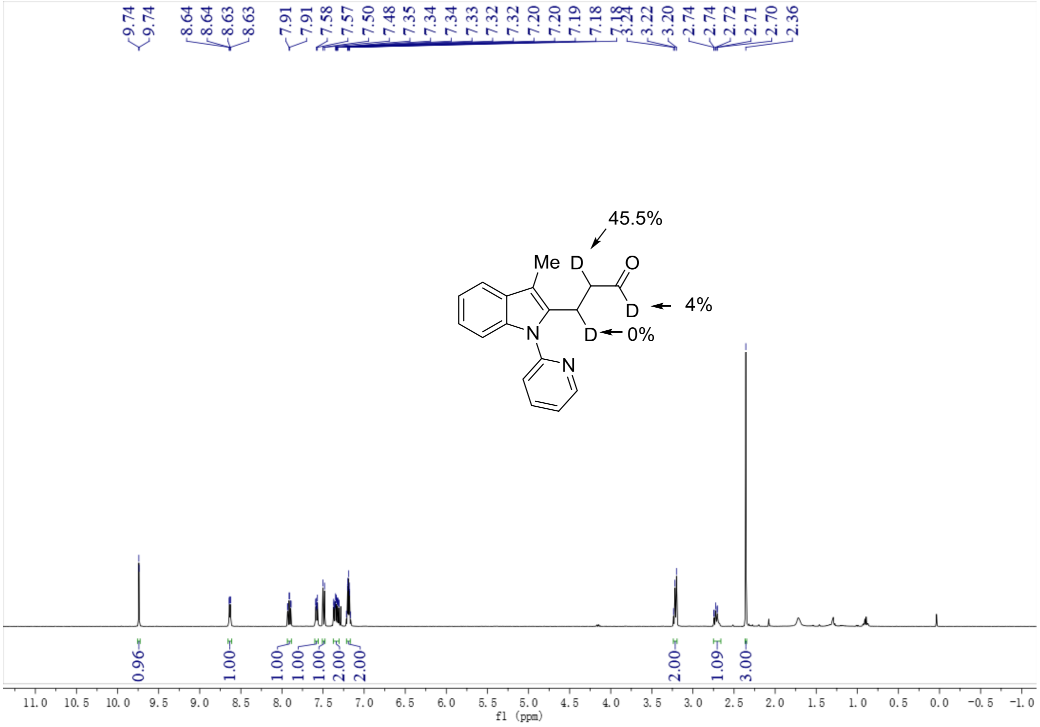

Supplementary Fig. 1 | The ^{1}H NMR spectra of *d*-**3a**.

2) H/D exchange experiment of aldehyde 3a

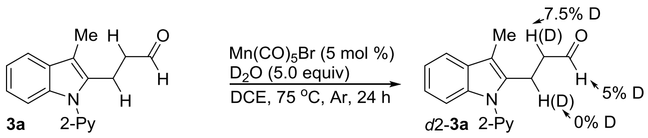

An oven-dried sealed tube charged **3a** (26.4 mg, 0.10 mmol), Mn(CO)$_5$Br (2.0 mg, 5 mol %)，D_2O (10.0 mg, 0.50 mmol) and DCE (1.0 mL) under Ar atmosphere. The reaction mixture was then allowed to stir at 75 ℃ for 24 h. After the reaction mixture was cooled down, the corresponding reaction mixture was purified by flash chromatography on silica gel using ethyl acetate/petroleum ether (1: 10) as eluent to afford the desired products *d2*-**3a** in which 7.5 D% was incorporated into the α-position of aldehyde *d2*-**3a** (see **Supplementary Fig. 2**).

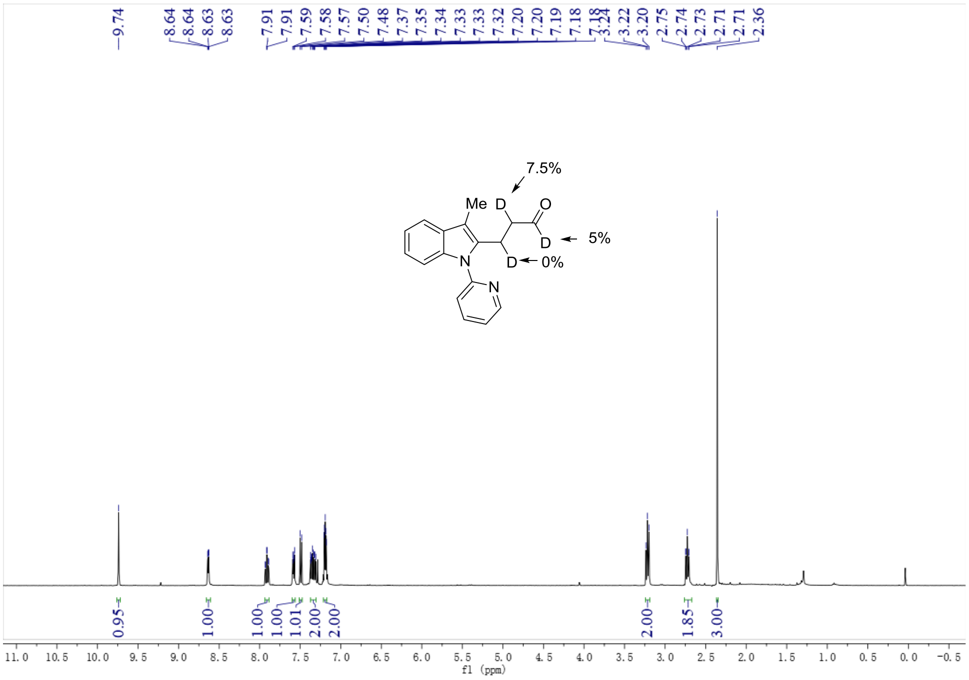

Supplementary Fig. 2 | The ^{1}H NMR spectra of *d2*-**3a**.

3) Intramolecular cyclization of α-[2-(*N*-phenyl) indolyl]allylic alcohol 1u

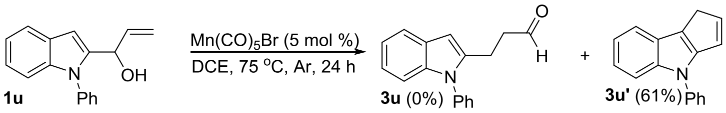

An oven-dried sealed tube charged allylic alcohol **1u** (49.8 mg, 0.20 mmol), $Mn(CO)_5Br$ (0.01 mmol, 5 mol %), and DCE (2.0 mL) under Ar atmosphere. The reaction mixture was then allowed to stir at 75 ℃ for 24 h. The intramolecular cyclization product **3u'** instead of in-situ group-inversion rearrangement product **3u** was obtained. **3u'** was purified by flash chromatography on silica gel using ethyl acetate/petroleum ether (1: 50) as eluent to afford the **3u'.**

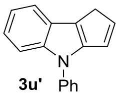
3u'

4-Phenyl-1,4-dihydrocyclopenta[b]indole (3u'): light yellow solid; m.p. 213.7 – 216.5 ^{o}C; 28.2 mg, 61% yield; ^{1}H NMR (500 MHz, $CDCl_3$) δ 7.48 – 7.41 (m, 3H), 7.31 – 7.25 (m, 3H), 7.09 – 7.05 (m, 3H), 6.48 (d, *J* = 16.0 Hz, 1H), 5.76 – 5.69 (m, 1H), 3.66 (d, *J* = 6.8 Hz, 2H); ^{13}C NMR (126 MHz, $CDCl_3$) δ 138.6, 137.9, 134.5, 134.0, 129.3, 128.2, 127.9, 127.3, 122.3, 119.8, 118.5, 117.9, 112.7, 110.0, 29.6; HR-MS (ESI) calcd for [M + H]+: $C_{17}H_{14}N$: 232.1121, found: 232.1118; IR (KBr): 3047, 2921, 2855, 1596, 1499, 1452, 1368, 1220, 1019, 963, 739, 697 cm^{-1}.

4) Csp^2-Csp^3 σ bond activation-based carbonylethylation of α-(2-indolyl)allylicalcohol 1a

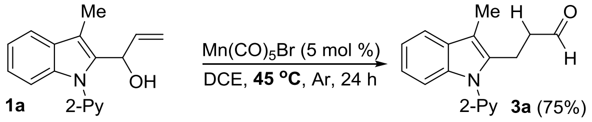

An oven-dried sealed tube charged allylic alcohol **1a** (52.8 mg, 0.20 mmol), $Mn(CO)_5Br$ (0.01 mmol, 5 mol %)， and DCE (2.0 mL) under Ar atmosphere. The reaction mixture was then allowed to stir at 45 ℃ for 24 h. After the reaction mixture was cooled down, the corresponding reaction mixture was purified by flash chromatography on silica gel using ethyl acetate/petroleum

ether (1: 10) as eluent to afford the desired products **3a** (39.6 mg, 75% yield).

5) Csp^2-H bond activation-based carbonylethylation of 2-unsubstituted indole 7

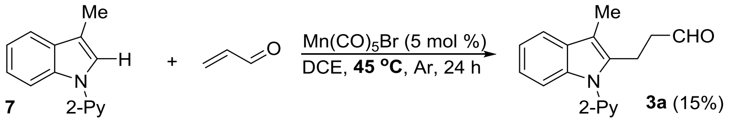

An oven-dried sealed tube charged 2-unsubstituted indole **7** (41.6 mg, 0.20 mmol), acrylaldehyde (11.2 mg, 0.20 mmol), $Mn(CO)_5Br$ (0.01 mmol, 5 mol %) and DCE (2.0 mL) under Ar atmosphere. The reaction mixture was then allowed to stir at 45 ℃ for 24 h. After the reaction mixture was cooled down, the corresponding reaction mixture was purified by flash chromatography on silica gel using ethyl acetate/petroleum ether (1: 10) as eluent to afford the desired products **3a** (7.9 mg, 15% yield).

6) Mn(I)-catalyzed cross-coupling reaction between different α-(2-indolyl)allylicalcohols 1g and 4a

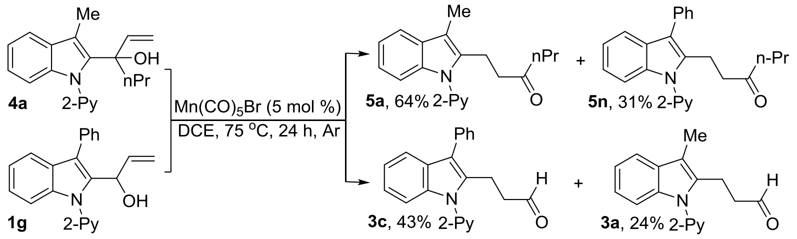

An oven-dried sealed tube charged allylicalcohol **4a** (30.6 mg, 0.10 mmol), **1g** (32.6 mg, 0.10 mmol), $Mn(CO)_5Br$ (3.0 mg, 0.01 mmol) and DCE (2.0 mL) under Ar atmosphere. The reaction mixture was then allowed to stir at 75 ℃ for 24 h. After the reaction mixture was cooled down, the corresponding reaction mixture was purified by flash chromatography on silica gel using ethyl acetate/petroleum ether (1: 10) as eluent to afford the products **5a** (19.5 mg, 64% yield); **5n** (11.5 mg, 31% yield); **3c** (14.1 mg, 43% yield); **3a** (6.3 mg, 24% yield).

1.8. Single crystal structure and data

1) The single crystal structure and data of **5m**

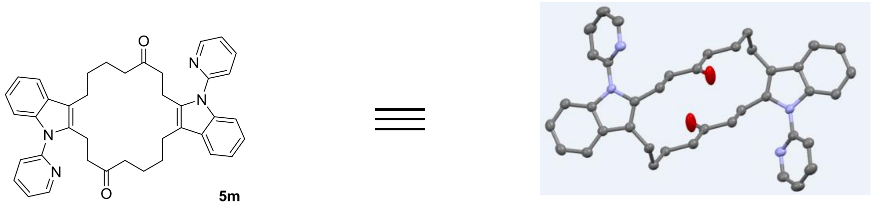

The crystal structure of **5m**

Supplementary Fig. 3 | The single crystal structure of **5m** (the ellipsoid contour probability level is 30%).

Supplementary Table 5 | Crystal data and structure refinement for 5m

Identification code	**5m**
Empirical formula	$C_{40}H_{40}N_4O_2$
Formula weight	608.76
Temperature/K	149.99(10) K

Wavelength	1.54178 Å
Crystal system	Monoclinic
Space group	C2/c
a/Å	21.8876(11)
b/Å	5.8545(3)
c/Å	27.6957(16)
α/°	90
β/°	100.788(6)
γ/°	90
Volume/$Å^3$	3486.2(3)
Z，Calculated density	4, 1.160 Mg/m^3
Absorption coefficient	0.565 mm^{-1}
F (000)	1296
Crystal size/mm^3	0.150 * 0.100 * 0.080
Theta range for data collection	3.249 to 73.761 deg
Limiting indices	-23<=h<=26, -7<=k<=4, -34<=l<=26
Reflections collected / unique	5895 / 3386 [R(int) = 0.0398]
Completeness to theta = 25.242	99.8 %
Absorption correction	Semi-empirical from equivalents
Max. and min. transmission	1.00000 and 0.74784
Refinement method	Full-matrix least-squares on F^2
Data / restraints / parameters	3386 / 0 / 208
Goodness-of-fit on F^2	1.071
Final R indices [I>2sigma(I)]	R1 = 0.0607, wR2 = 0.1713
R indices (all data)	R1 = 0.0842, wR2 = 0.1955
Extinction coefficient	n/a
Largest diff. peak and hole	0.820 and -0.230 $e.A^{-3}$

2) The single crystal structure and data of **7f**

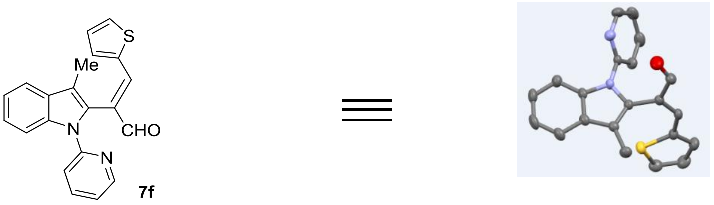

The crystal structure of **7f**

Supplementary Fig. 4 | The single crystal structure of **7f** (the ellipsoid contour probability level is 30%)

Supplementary Table 6 | Crystal data and structure refinement for 7f

Identification code	**7f**

Empirical formula	$C_{21}H_{16}N_2OS$
Formula weight	344.42
Temperature/K	199.98(10) K
Wavelength	0.71073 Å
Crystal system	monoclinic
Space group	P2(1)/n
a/Å	9.9522(8)
b/Å	17.0333(13)
c/Å	10.1226(8)
α/°	90
β/°	96.085(7)
γ/°	90
Volume/Å^3	1706.3(2)
Z，Calculated density	4，1.341 Mg/m^3
Absorption coefficient	0.200 mm^{-1}
F (000)	720.0
Crystal size/mm^3	0.140 * 0.130 * 0.120
Theta range for data collection	2.350 to 29.340 deg
Limiting indices	-13<=h<=8, -22<=k<=23, -13<=l<=12
Reflections collected / unique	9813 / 4052 [R(int) = 0.0253]
Completeness to theta = 25.242	100.0 %
Absorption correction	Semi-empirical from equivalents
Max. and min. transmission	1.00000 and 0.40333
Refinement method	Full-matrix least-squares on F^2
Data / restraints / parameters	4052 / 0 / 227
Goodness-of-fit on F^2	0.996
Final R indices [I>2sigma(I)]	R1 = 0.0444, wR2 = 0.1334
R indices (all data)	R1 = 0.0595, wR2 = 0.1490
Extinction coefficient	n/a
Largest diff. peak and hole	0.233 and -0.343 e.A^{-3}

3) The single crystal structure and data of **7t**

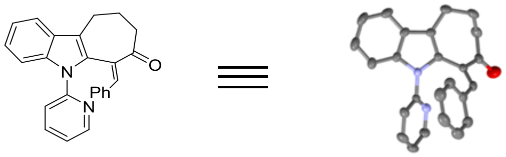

7t The crystal structure of **7t**

Supplementary Fig. 5 | The single crystal structure of **7t** (the ellipsoid contour probability level is 30%).

Supplementary Table 7 | Crystal data and structure refinement for 7t.

Identification code	**7t**
Empirical formula	$C_{25}H_{20}N_2O$
Formula weight	364.43
Temperature/K	200.01(10) K
Wavelength	0.71073 Å
Crystal system	monoclinic
Space group	P2(1)/n
a/Å	8.3992(6)
b/Å	20.9553(16)
c/Å	11.0063
α/°	90
β/°	100.347(7)
γ/°	90
Volume/Å^3	1905.7(2)
Z，Calculated density	4, 1.270 Mg/m^3
Absorption coefficient	0.078 mm^{-1}
F (000)	768
Crystal size/mm^3	0.140 * 0.130 * 0.120
Theta range for data collection	2.117 to 25.008 deg
Limiting indices	-8<=h<=9, -24<=k<=24, -13<=l<=10
Reflections collected / unique	8289 / 3339 [R(int) = 0.0245]
Completeness to theta = 25.242	100.0 %
Absorption correction	Semi-empirical from equivalents
Max. and min. transmission	1.00000 and 0.88392
Refinement method	Full-matrix least-squares on F^2
Data / restraints / parameters	3339 / 0 / 253
Goodness-of-fit on F^2	0.991
Final R indices [I>2sigma(I)]	R1 = 0.0452, wR2 = 0.1295
R indices (all data)	R1 = 0.0583, wR2 = 0.1432
Extinction coefficient	n/a
Largest diff. peak and hole	0.337 and -0.251 e.A^{-3}

1.9. Computational detail

All the calculations were accomplished using Gaussian 09D package[15]. The geometries were calculated by density functional theory (DFT). All the structures optimization and frequency calculation were performed by the M06-L/Def2-SVP level at 298.15 K and 1 atm[16-19]. The ultrafine integration grid was used in the structures optimization and frequency calculation. The intrinsic reaction coordinate (IRC)[20] was prepared at the same level as above. To further accurate energies, the single-point energy calculation with solvation effects was performed by M06-L

functional. A larger mixed basis set Def2-tzvp[19, 21] was in used single-point energy calculation. Solvation effects were performed in dichloroethane solvent with SMD method[22]. Because of the experiment condition at 348.15 K, the entropic contribution has been overrated in the solution. The correction of entropy was considered for all the species[23]. The orbital figures were performed by Multiwfn and VMD programs[24].

The general reaction mechanism is firstly proposed in **Supplementary Fig. 6**. Meanwhile, the possible mechanism for the formation of **5l** and **5m** was also shown in **Supplementary Fig. 7**, in which the interaction between allylic alcohol **4l** or **4m** with $Mn(CO)_5Br$ produced cyclomanganated species **A-4**, followed by chelation-assisted β-aryl elimination to give six-coordination Mn-carbonyl complexes **B-4**. Subsequently, bimolecular migratory insertion of Mn-intermediate **B-4** and protonation of carbon-Mn bonds of complexes **B-5** afforded the macrocyclic products **5l** and **5m**.

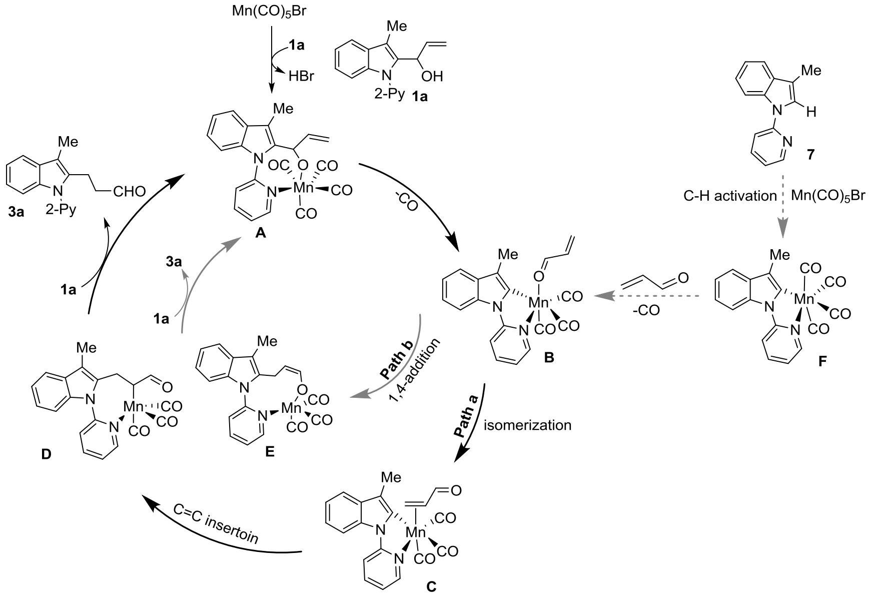

Supplementary Fig. 6 | Proposed reaction mechanism. Path a Mn(I)-catalyzed 1,3-STR *via* intramolecular ligand exchange and migratory insertion. **Path b** Mn(I)-catalyzed 1,3-STR *via* intramolecular Michael-addition.

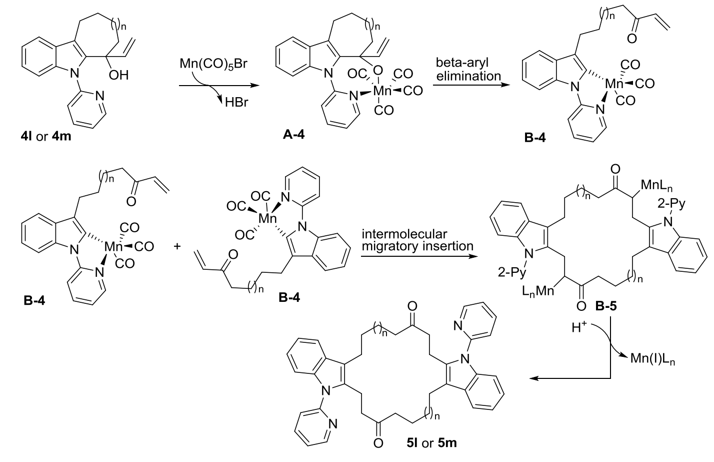

Supplementary Fig. 7 | Proposed reaction mechanism for the formation of 5l and 5m.

1) Discussion about the influence of different functions

The key intermediates and transition states ware calculated using M06-L, M06, B3LYP(D3BJ), PBE0(D3BJ), and MN15 functional. All the structures optimization and frequency calculations were done with the ultrafine integration grid. All the result lead to the same conclusion that the Gibbs activation energies of the β-aryl elimination step via ***cisoid*-TS1** or ***transoid*-TS1** with are higher, to the same extent, than those of the 1,4-addition and the C=C insertion steps (see table below). For the β-aryl elimination step, the Gibbs activation energies with B3LYP(D3BJ), PBE0(D3BJ), and MN15 functional are slightly higher than that with M06-L, M06(D3) functional. While the Gibbs activation energies of the C=C insertion via the ***cisoid*-TS2** and ***transoid*-TS2** with B3LYP(D3BJ) functional are slightly higher than that with M06-L, M06(D3), PBE0(D3BJ), and MN15 functional. The Gibbs activation energy of the ***cisoid*-TS3** with B3LYP(D3BJ) functional is also lower than that of PBE0(D3BJ) and MN15 functional. The results suggest that Minnesota functional (M06-L, M06, and MN15) can well present the dispersion effect. The calculated results with M06-L, M06, and MN15 functional are consistent with the experimental results.

Overall, the DFT computational results with B3LYP(D3BJ) and PBE0(D3BJ) functional and Minnesota functional with ultrafine integration grid do not change the mechanism and the discussion.

Supplementary Table 8 | The comparison of the Gibbs free energies of the key intermediates and transition states using different functions

	M06-L	M06-L(D3)	B3LYP(D3BJ)	PBE0(D3BJ)	MN15	M06(D3)	M06
***cisoid*-A**	0.0	0.0	0.0	0.0	0.0	0.0	0.0
***transoid*-A**	2.5	2.5	0.5	0.4	1.6	0.4	1.2
***cisoid*-TS1**	16.2	16.6	18.9	18.8	18.2	16.3	15.4
***transoid*-TS1**	18.5	18.8	20.3	19.9	19.4	17.2	16.7
***cisoid*-B**	1.4	1.1	0.5	3.3	4.5	1.4	0.7
***transoid*-B**	-1.2	-0.4	-1.9	1.1	3.8	0.0	-2.0
***cisoid*-C**	4.8	5.2	6.9	5.1	4.6	2.1	1.3
***transoid*-C**	2.7	3.2	4.9	3.5	2.7	0.7	0.2
***cisoid*-TS2**	11.4	10.6	13.9	13.1	12.3	8.0	7.5
***transoid*-TS2**	10.3	11.9	12.3	12.1	10.9	7.0	6.3
***cisoid*-TS3**	18.7	18.8	15.5	25.4	23.0	19.2	18.8

2) The full version of the computed potential energy surface

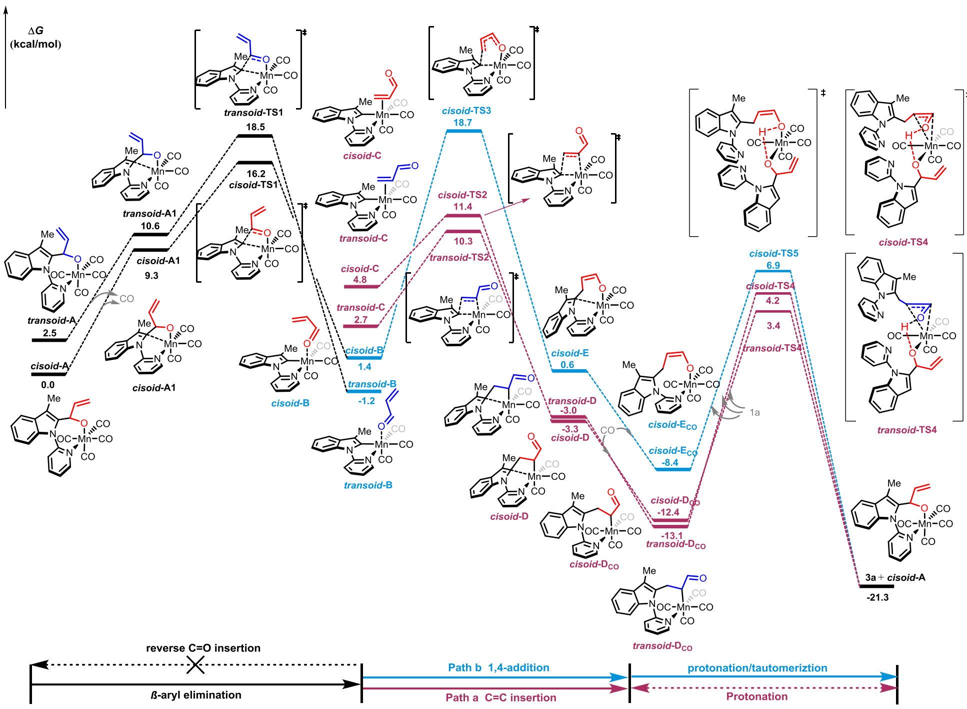

Supplementary Fig. 8 | Computed potential energy surface for the Mn(I)-catalyzed carbon-skeleton rearrangement *via* C-C bond activation at the SMD(DCE)/M06L/Def2tzvp//M06L/def2svp level of theory. Energies are in kcal/mol. Purple line The DFT-computed energy surfaces of Path a. **Blue line** The DFT-computed energy surfaces of Path b.

3) Discussion about the formation of the active species from the $Mn(CO)_5Br$ and 1a.

The generation of cyclomanganated complex **A** from the $Mn(CO)_5Br$ and **1a** has been investigated. The first step is the ligand-exchange of $Mn(CO)_5Br$ generating the six-coordination intermediates ***cisoid*-A$_{pre}$** (-13.4 kcal/mol) or ***transoid*-A$_{pre}$** (-12.1 kcal/mol), in which the pyridine coordinate with Mn center. Then, with the assistance of the bromide, the alcohol is deprotonated via transition states ***cisoid*-TS6** or ***transoid*-TS6**, respectively. The Gibbs free energy of activation of ***cisoid*-TS6** is 5.8 kcal/mol, which is lower than that of ***transoid*-TS6** by 2.5 kcal/mol. The formation of the active species has to overcome relatively high Gibbs free energy of activation.

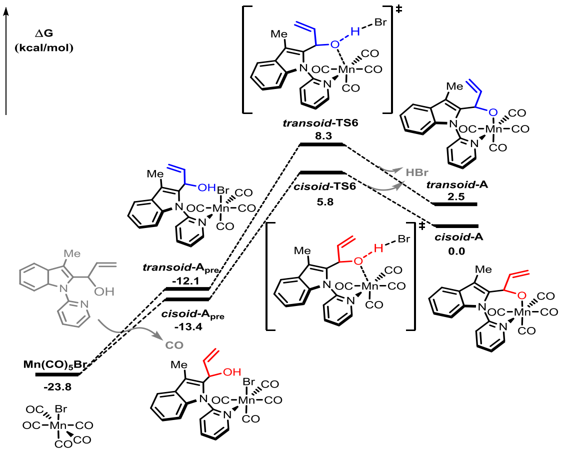

Supplementary Fig. 9 | Computed potential energy surface for the reaction between $Mn(CO)_5Br$ and 1a at the SMD(DCE)/M06L/Def2tvzp//M06L/def2svp level of theory.

4) Discussion about the formation of the active species from the $Mn(CO)_5Br$ and 1a.

Supplementary Table 9 | Absolute energies of all optimized structures

Species	E_c	H_c	G_c	$G_{c(T=348.15K)}$	*E*/(Hartree)	*H*/(Hartree)	*G*/(Hartree)	Solvation Energy/(Hartree)	G_{sol} /(Hartree)
Mn(CO)5Br	0.043528	0.058250	0.002114	-0.007300	-4290.830790	-4290.816068	-4290.872204	-4292.005367	-4292.012668
CO	0.005078	0.008383	-0.014052	-0.017815	-113.203742	-113.200437	-113.222872	-113.338245	-113.356061
HBr	0.006080	0.009385	-0.013143	-0.016920	-2574.346434	-2574.343129	-2574.365656	-2574.668463	-2574.685383
**1a**	0.292213	0.310442	0.247014	0.236377	-841.146017	-841.127788	-841.191217	-842.351544	-842.115167
**3a**	0.292301	0.310416	0.246349	0.235606	-841.178914	-841.160799	-841.224866	-842.384847	-842.149241
cisoid-**A**	0.315735	0.343906	0.257579	0.243101	-2444.385984	-2444.357813	-2444.444140	-2446.291606	-2446.048505
transoid-**A**	0.316206	0.344215	0.258646	0.244296	-2444.381845	-2444.353836	-2444.439405	-2446.288885	-2446.044589
cisoid-**A1**	0.308001	0.333271	0.254297	0.241053	-2331.151046	-2331.125776	-2331.204750	-2332.918748	-2332.677695
transoid-**A1**	0.307914	0.333398	0.253404	0.239989	-2331.146435	-2331.120952	-2331.200946	-2332.915539	-2332.675550
cisoid-**TS1**	0.305984	0.331287	0.251835	0.238511	-2331.140625	-2331.115323	-2331.194775	-2332.905070	-2332.666560
transoid-**TS1**	0.306135	0.331263	0.252771	0.239608	-2331.137416	-2331.112288	-2331.190780	-2332.902583	-2332.662975
cisoid-**B**	0.305878	0.332544	0.248502	0.234408	-2331.158405	-2331.131740	-2331.215782	-2332.924683	-2332.690275
transoid-**B**	0.305981	0.332568	0.248758	0.234703	-2331.161113	-2331.134525	-2331.218336	-2332.929109	-2332.694406
cisoid-**C**	0.306832	0.332647	0.252458	0.239010	-2331.155721	-2331.129905	-2331.210095	-2332.923822	-2332.684813
transoid-**C**	0.306942	0.332716	0.251989	0.238452	-2331.156934	-2331.131161	-2331.211887	-2332.926525	-2332.688073
cisoid-**TS2**	0.306945	0.331866	0.254124	0.241086	-2331.148320	-2331.123399	-2331.201142	-2332.915325	-2332.674239
transoid-**TS2**	0.306890	0.331803	0.253791	0.240709	-2331.147688	-2331.122775	-2331.200787	-2332.916802	-2332.676093
cisoid-**TS3**	0.306280	0.331271	0.253421	0.240366	-2331.141054	-2331.116063	-2331.193912	-2332.902979	-2332.662613
cisoid-**D**	0.308885	0.333836	0.256265	0.243257	-2331.174379	-2331.149428	-2331.226998	-2332.941030	-2332.697774
cisoid-$\mathbf{D}_{CO}$	0.317912	0.345275	0.262128	0.248185	-2444.410088	-2444.382725	-2444.465871	-2446.316484	-2446.068299
transoid-**D**	0.308211	0.333462	0.254535	0.241299	-2331.167321	-2331.142070	-2331.220997	-2332.938458	-2332.697159

Species	E_c	H_c	G_c	$G_{c(T=348.15K)}$	*E*/(Hartree)	*H*/(Hartree)	*G*/(Hartree)	Solvation Energy/(Hartree)	G_{sol}/(Hartree)
transoid-$\mathbf{D}_{CO}$	0.317509	0.345033	0.261225	0.247171	-2444.406354	-2444.378831	-2444.462638	-2446.316614	-2446.069443
cisoid-**E**	0.308292	0.333594	0.254877	0.241676	-2331.169358	-2331.144055	-2331.222772	-2332.933135	-2332.691459
cisoid-$\mathbf{E}_{CO}$	0.316443	0.344308	0.259317	0.245065	-2444.402086	-2444.374222	-2444.459212	-2446.307026	-2446.061961
cisoid-**TS4**	0.610282	0.655504	0.532012	0.511302	-3285.568685	-3285.523462	-3285.646955	-3288.668236	-3288.156934
transoid-**TS4**	0.608329	0.653798	0.528859	0.507906	-3285.569671	-3285.524202	-3285.649141	-3288.666203	-3288.158297
cisoid-**TS5**	0.606568	0.652351	0.526853	0.505807	-3285.562126	-3285.516343	-3285.641841	-3288.658472	-3288.152665
cisoid-**TS6**	0.328427	0.358498	0.267291	0.251995	-5018.739804	-5018.709733	-5018.800940	-5020.976572	-5020.724577
cisoid-$\mathbf{A}_{pre}$	0.329348	0.360113	0.267567	0.252046	-5018.772845	-5018.742080	-5018.834627	-5021.007271	-5020.755225
transoid-**TS6**	0.328874	0.358745	0.268699	0.253598	-5018.737661	-5018.707791	-5018.797836	-5020.974321	-5020.720723
transoid-$\mathbf{A}_{pre}$	0.329534	0.360269	0.267977	0.252500	-5018.770656	-5018.739922	-5018.832213	-5021.005711	-5020.753211

II. ^{1}H NMR and ^{13}C NMR Spectrum of All Products.

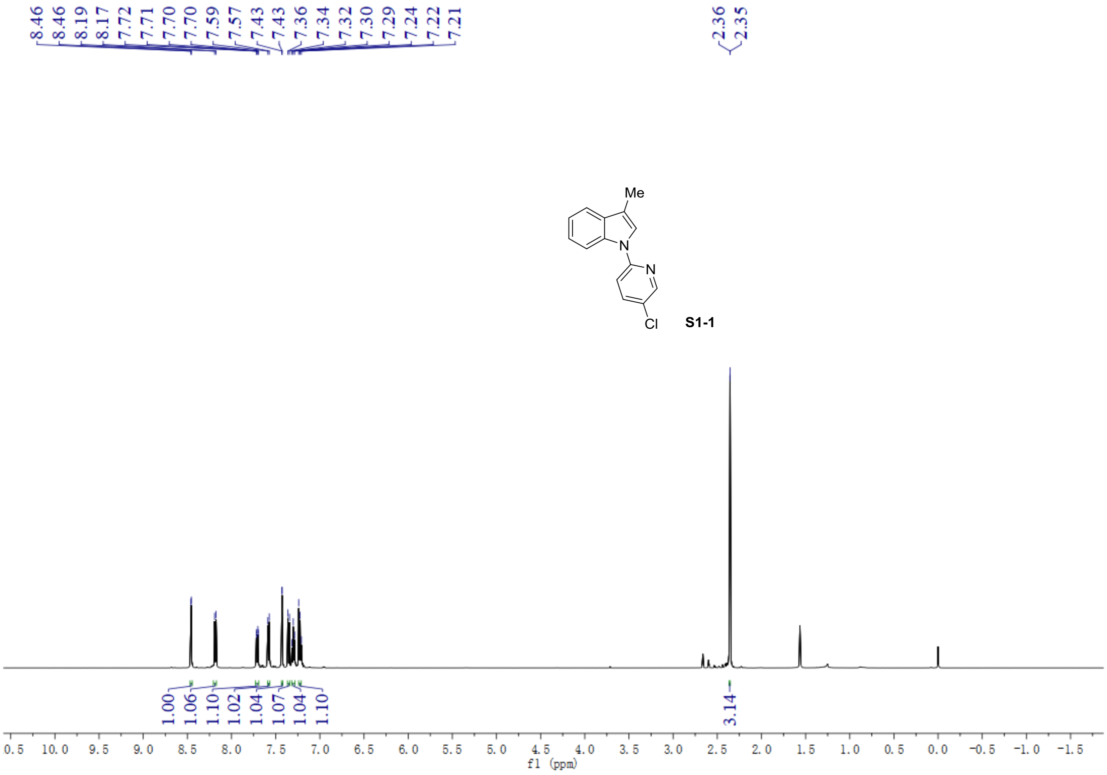

Supplementary Fig. 10 | ^{1}H NMR (500 MHz) of compound S1-1 (using $CDCl_3$ as solvent)

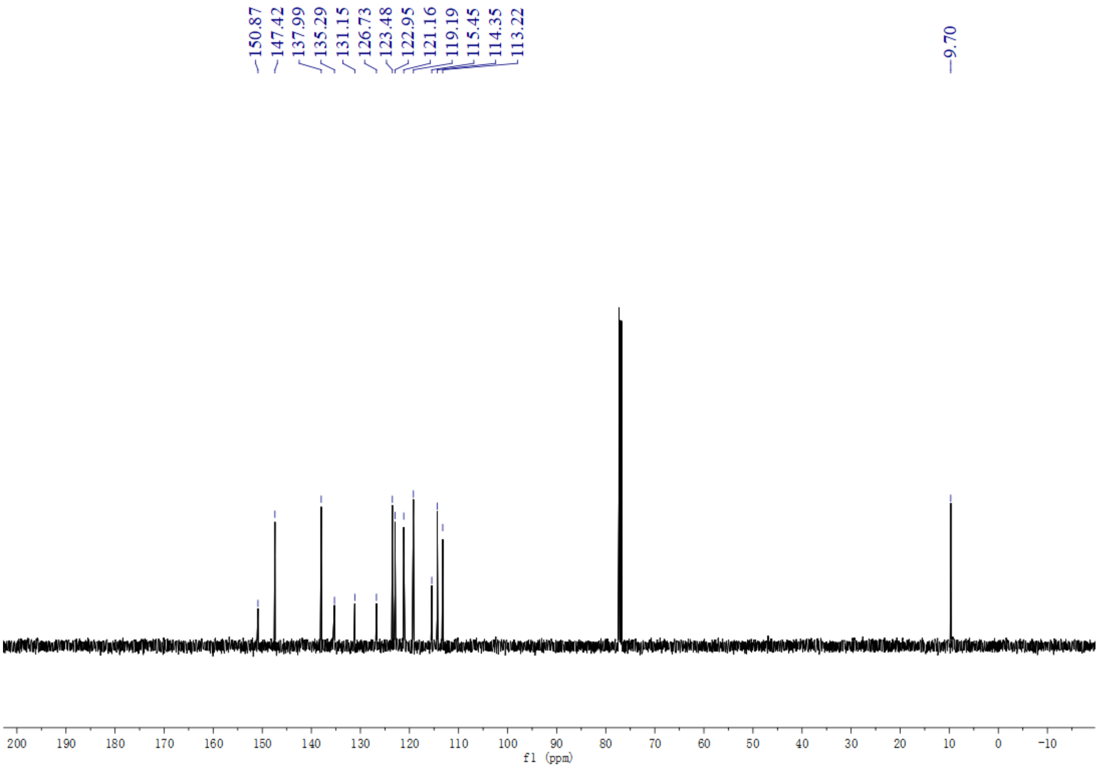

Supplementary Fig. 11 | ^{13}C NMR (126 MHz) of compound S1-1 (using $CDCl_3$ as solvent)

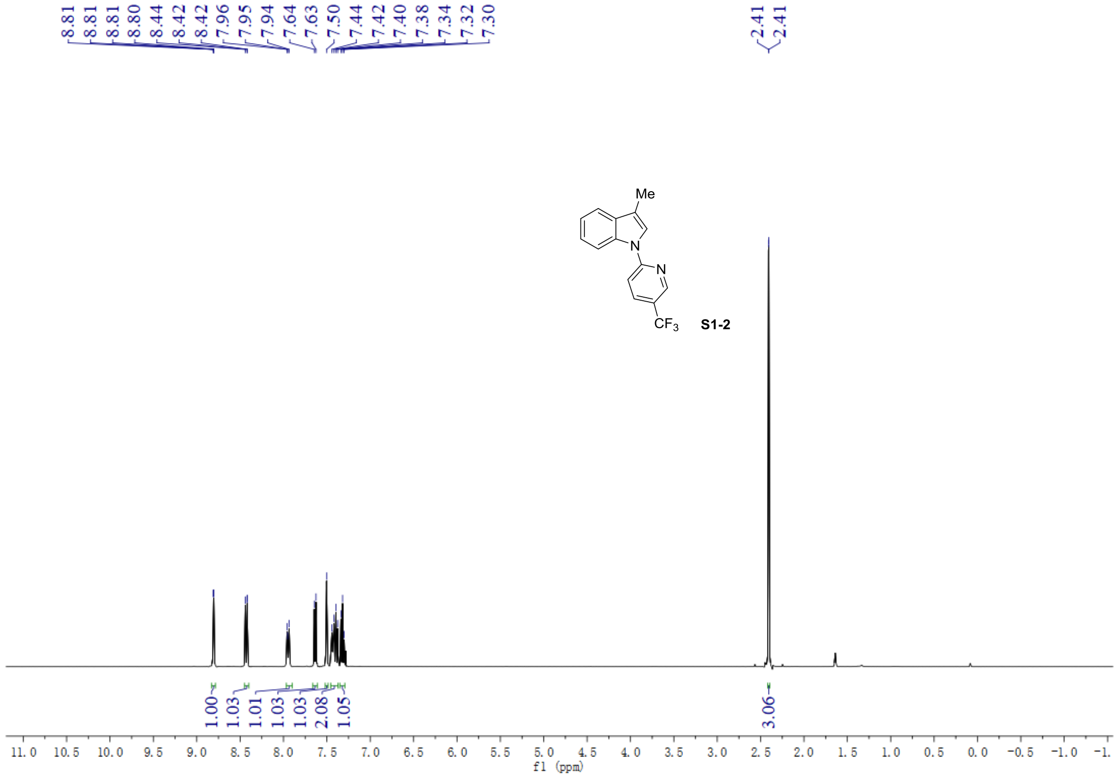

Supplementary Fig. 12 | ^{1}H NMR (400 MHz) of compound S1-2 (using $CDCl_3$ as solvent)

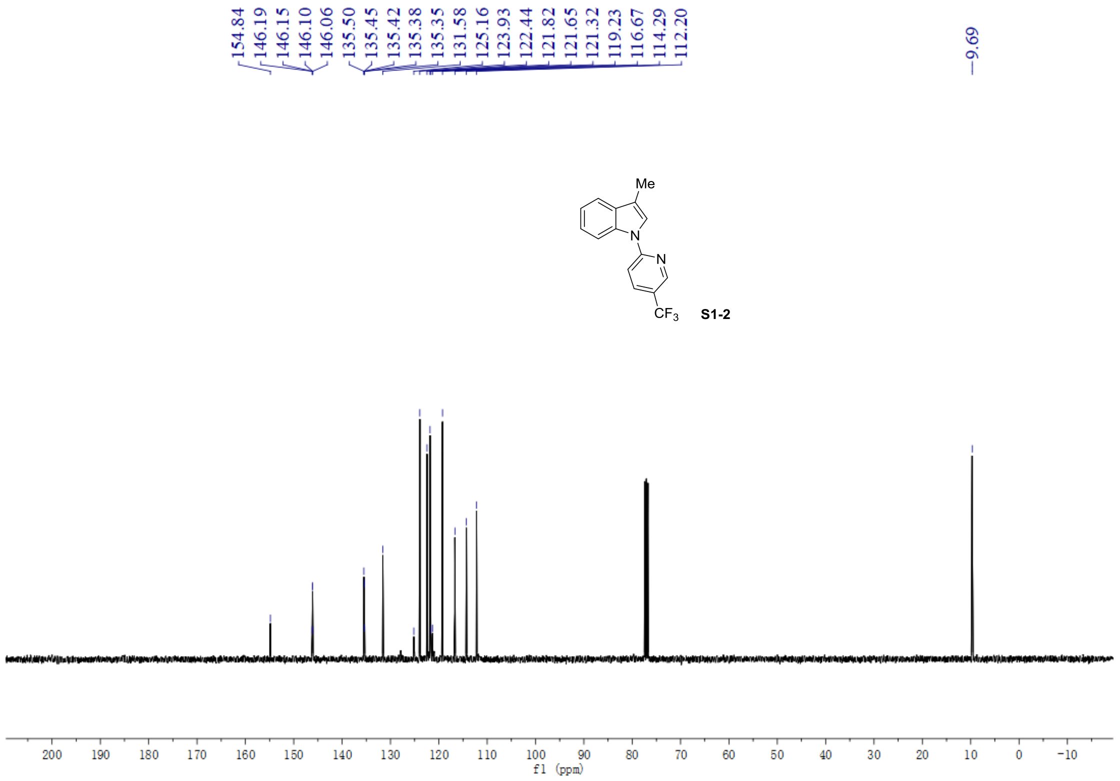

Supplementary Fig. 13 | ^{13}C NMR (101 MHz) of compound S1-2 (using $CDCl_3$ as solvent)

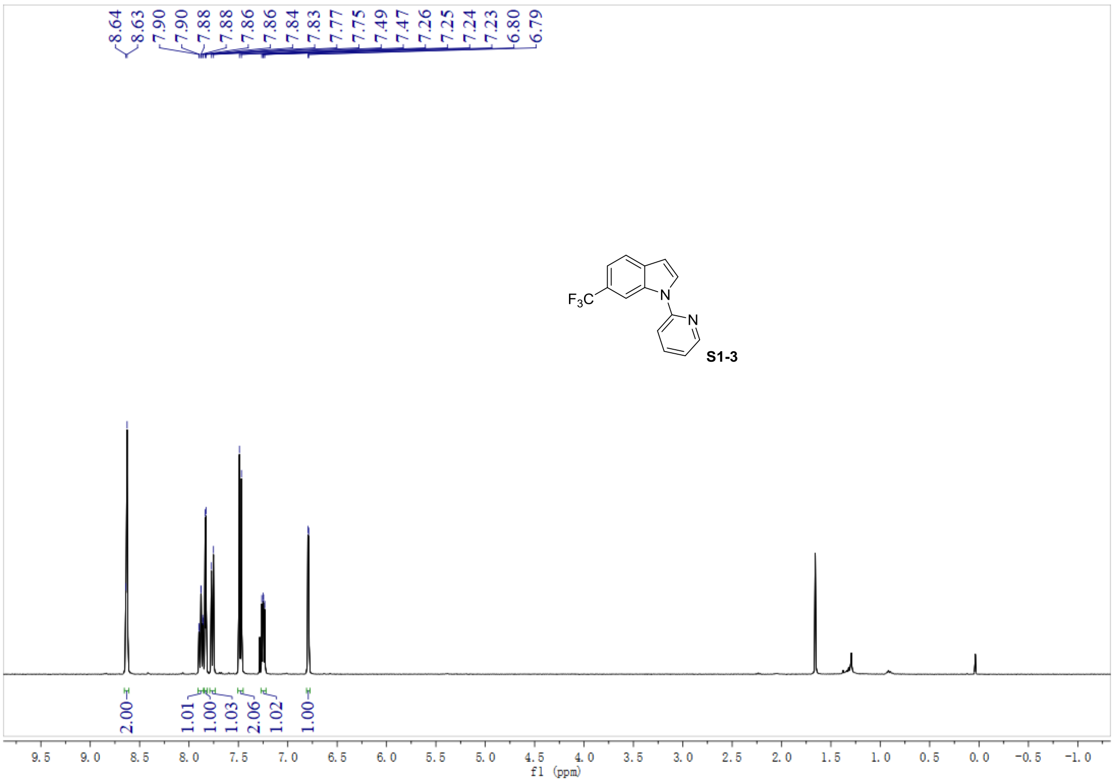

Supplementary Fig. 14 | ^{1}H NMR (400 MHz) of compound S1-3 (using $CDCl_3$ as solvent)

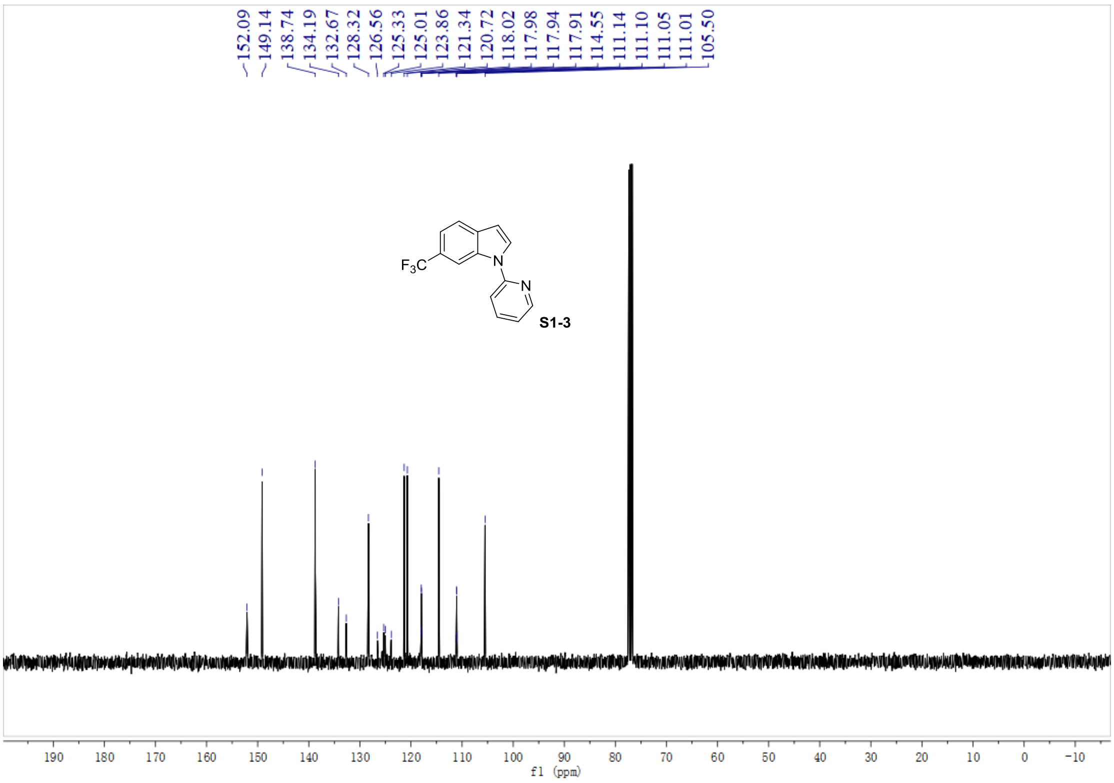

Supplementary Fig. 15 | ^{13}C NMR (101 MHz) of compound S1-3 (using $CDCl_3$ as solvent)

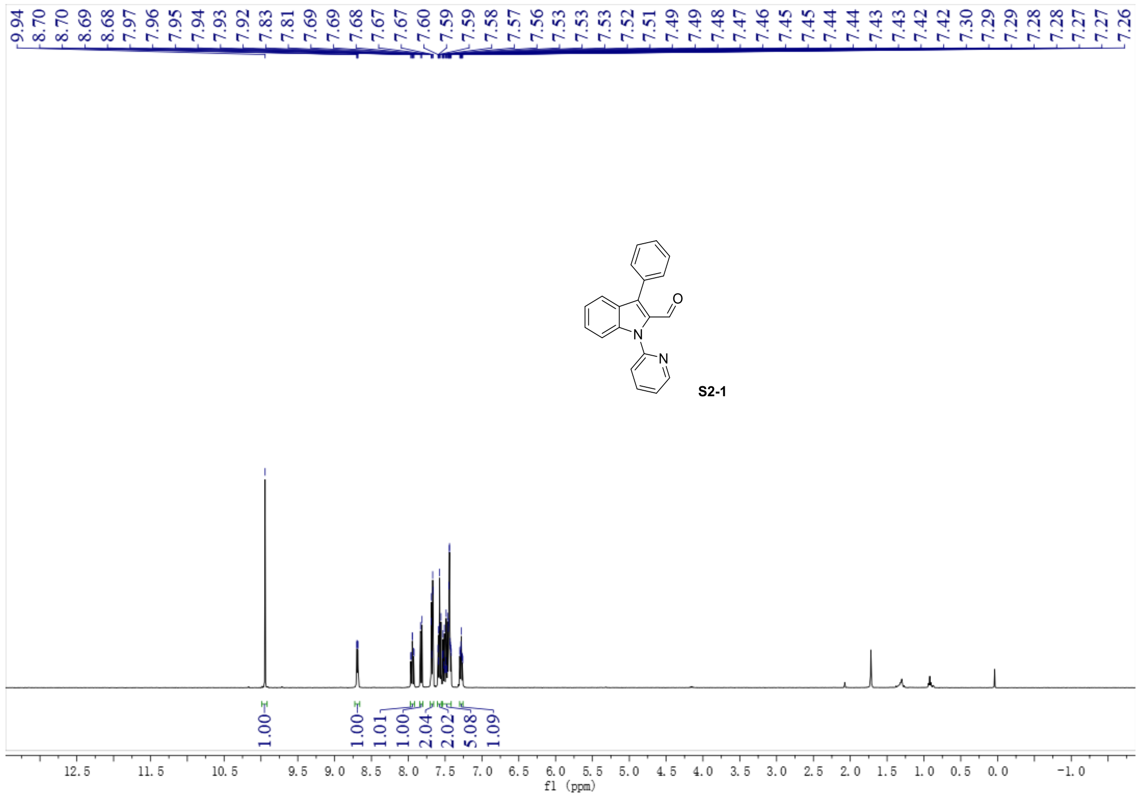

Supplementary Fig. 16 | ^{1}H NMR (400 MHz) of compound S2-1 (using $CDCl_3$ as solvent)

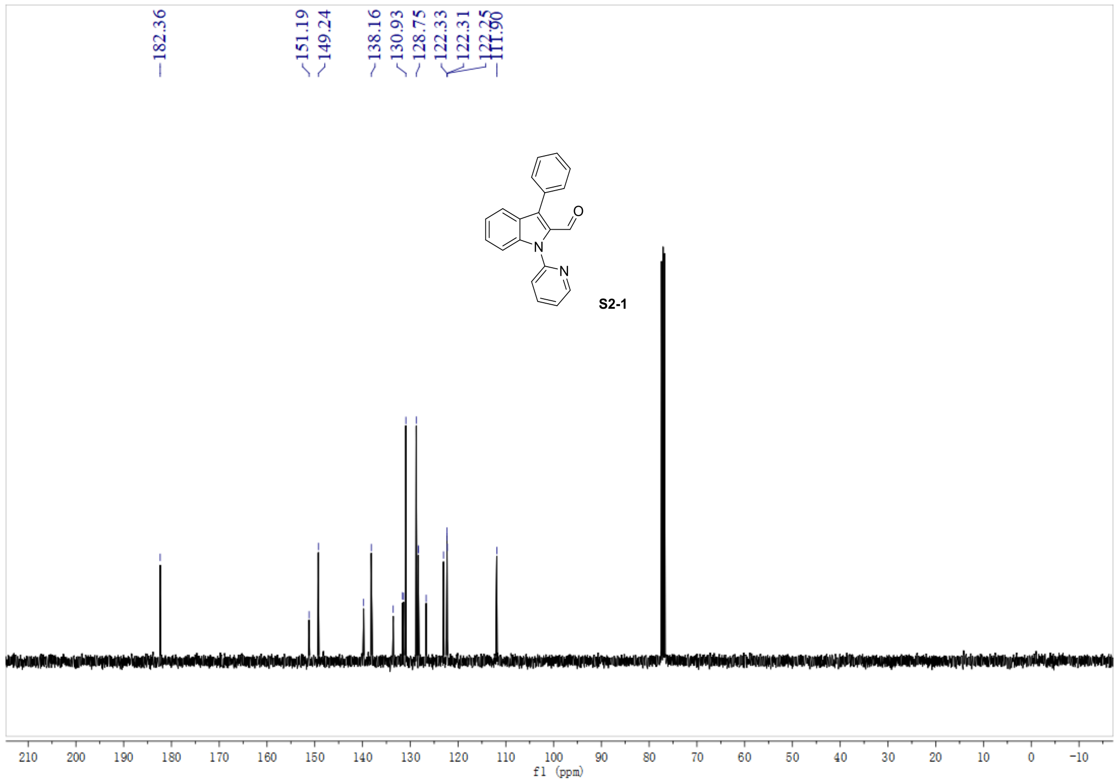

Supplementary Fig. 17 | ^{13}C NMR (101 MHz) of compound S2-1 (using $CDCl_3$ as solvent)

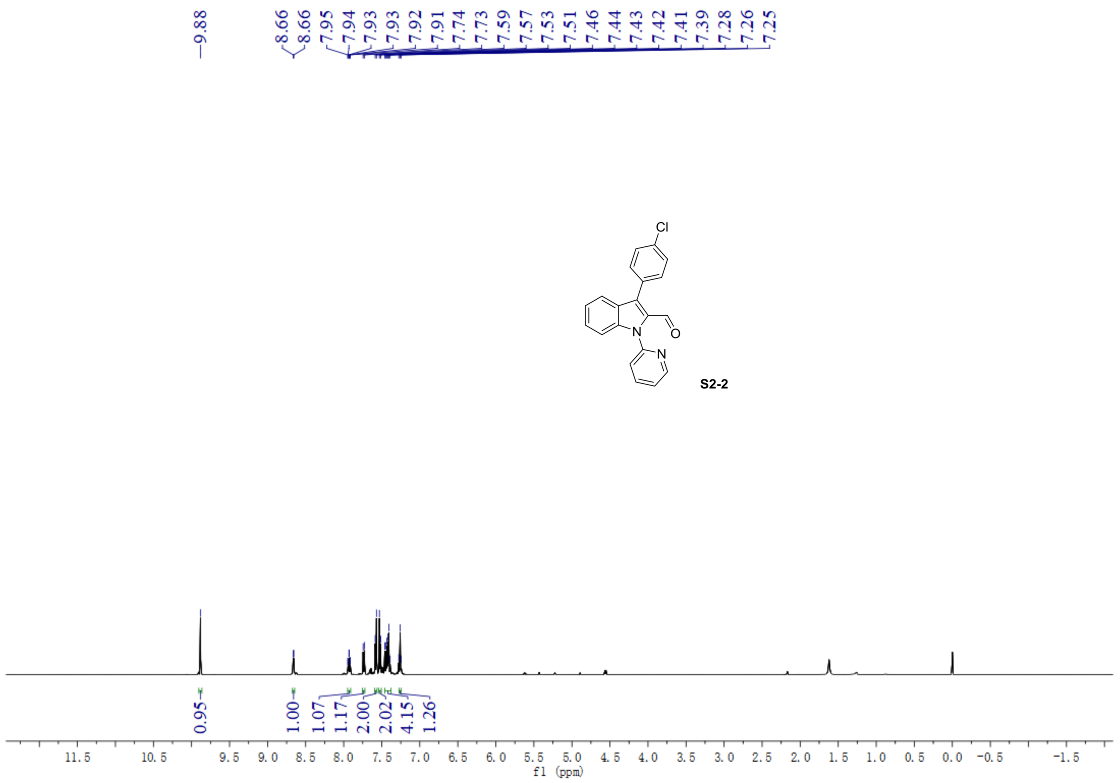

Supplementary Fig. 18 | ^{1}H NMR (500 MHz) of compound S2-2 (using $CDCl_3$ as solvent)

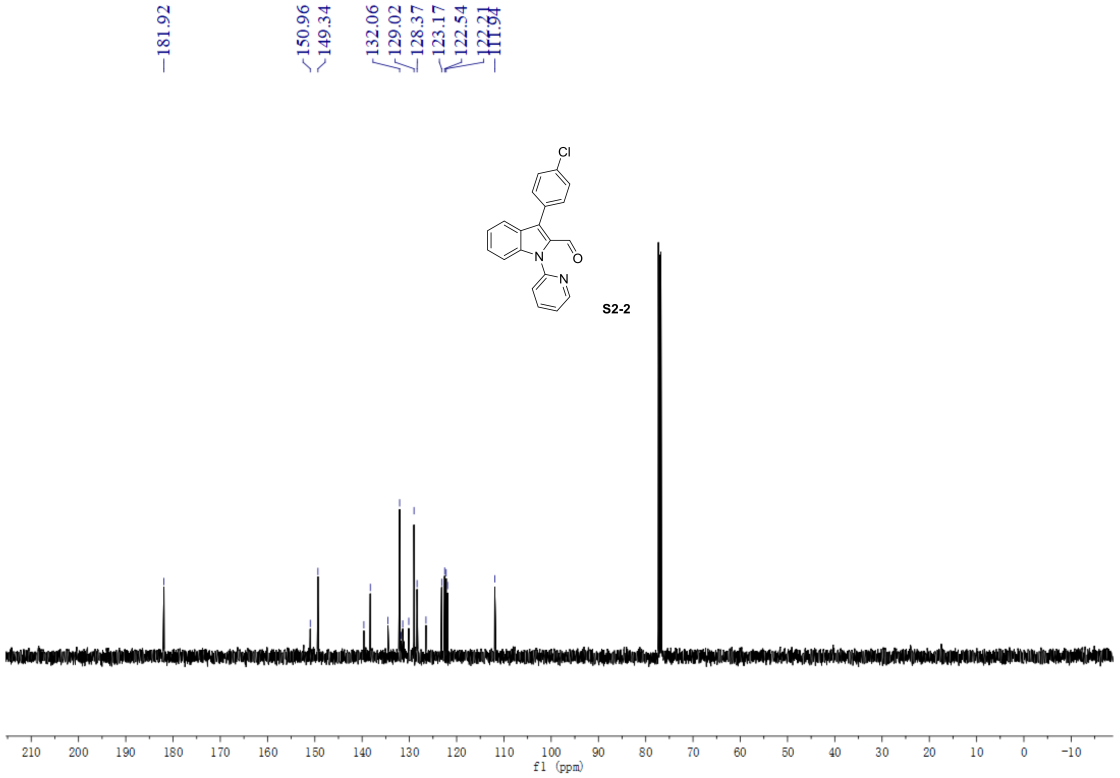

Supplementary Fig. 19 | ^{13}C NMR (126 MHz) of compound S2-2 (using $CDCl_3$ as solvent)

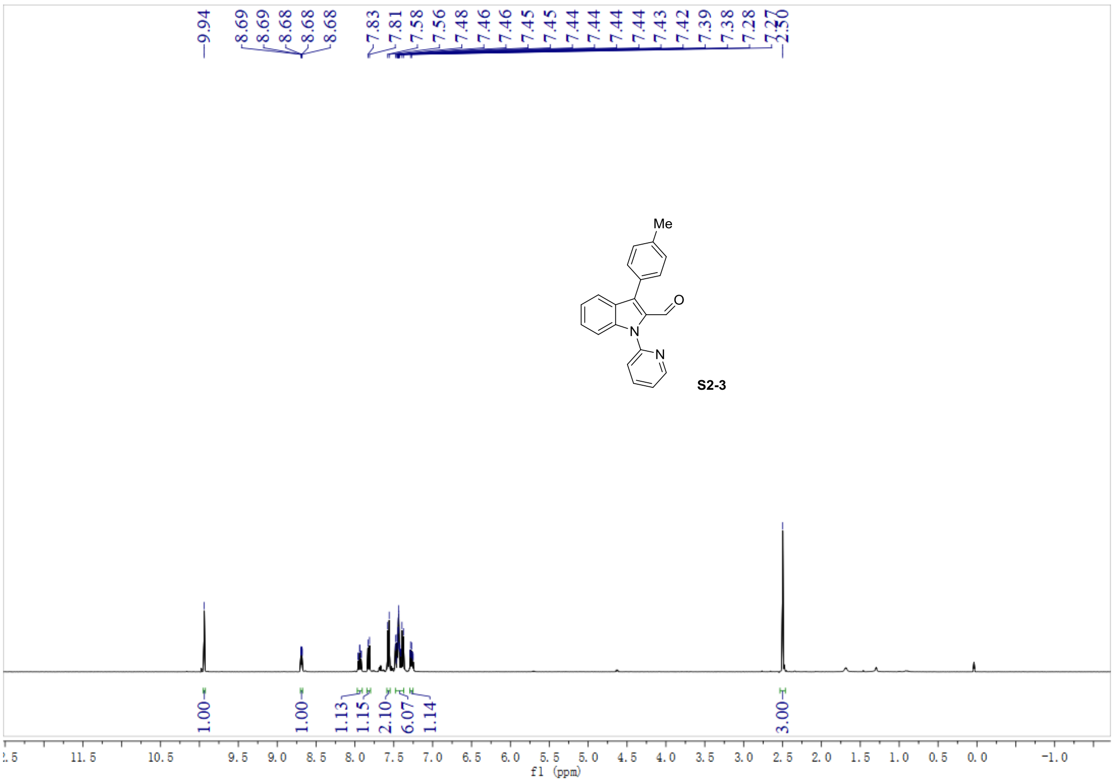

Supplementary Fig. 20 | ^{1}H NMR (400 MHz) of compound S2-3 (using $CDCl_3$ as solvent)

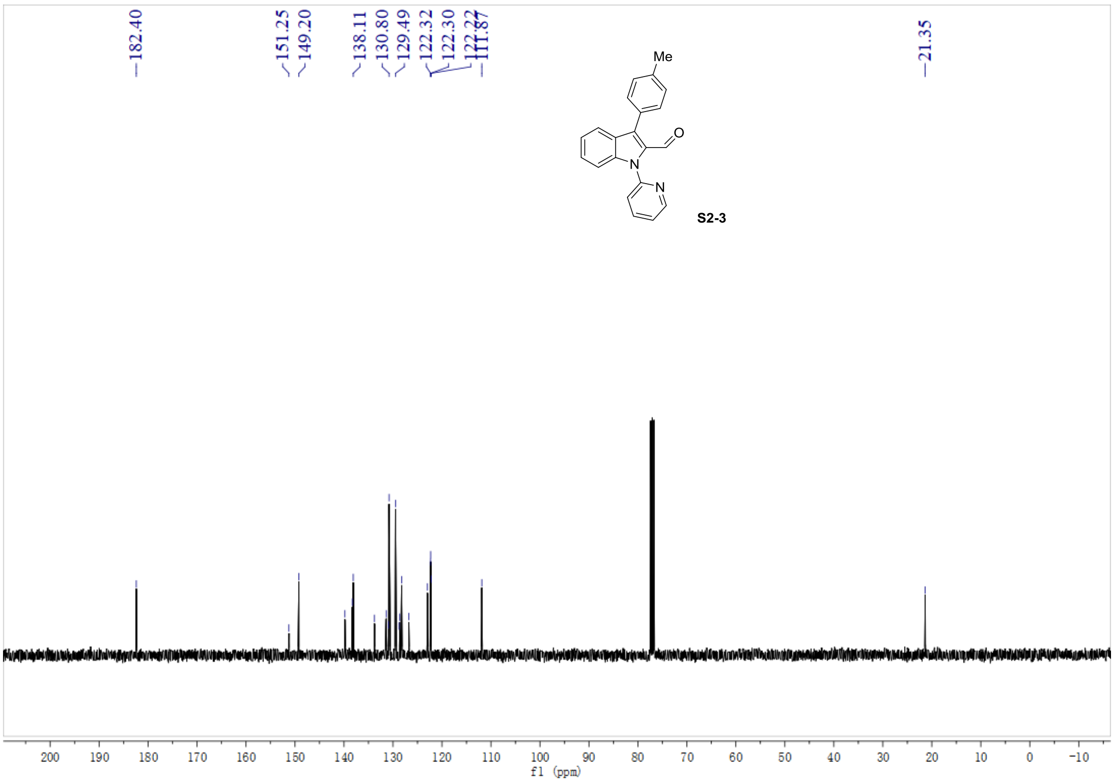

Supplementary Fig. 21 | ^{13}C NMR (101 MHz) of compound S2-3 (using $CDCl_3$ as solvent)

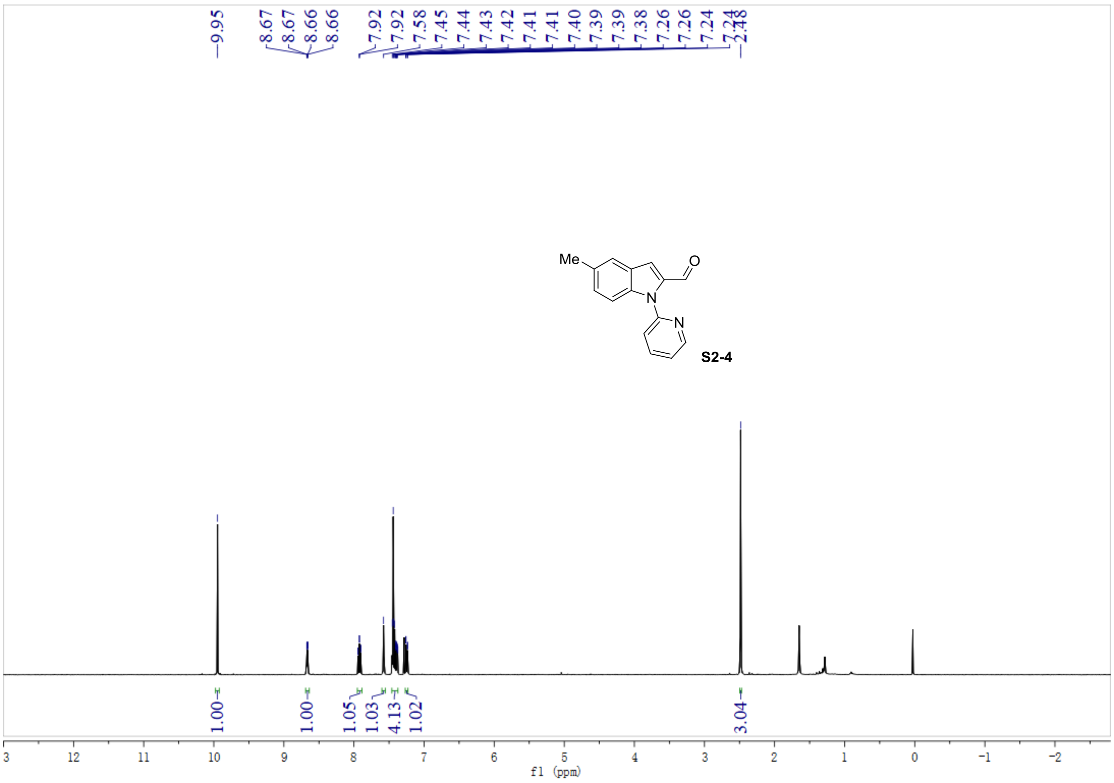

Supplementary Fig. 22 | ^{1}H NMR (400 MHz) of compound S2-4 (using $CDCl_3$ as solvent)

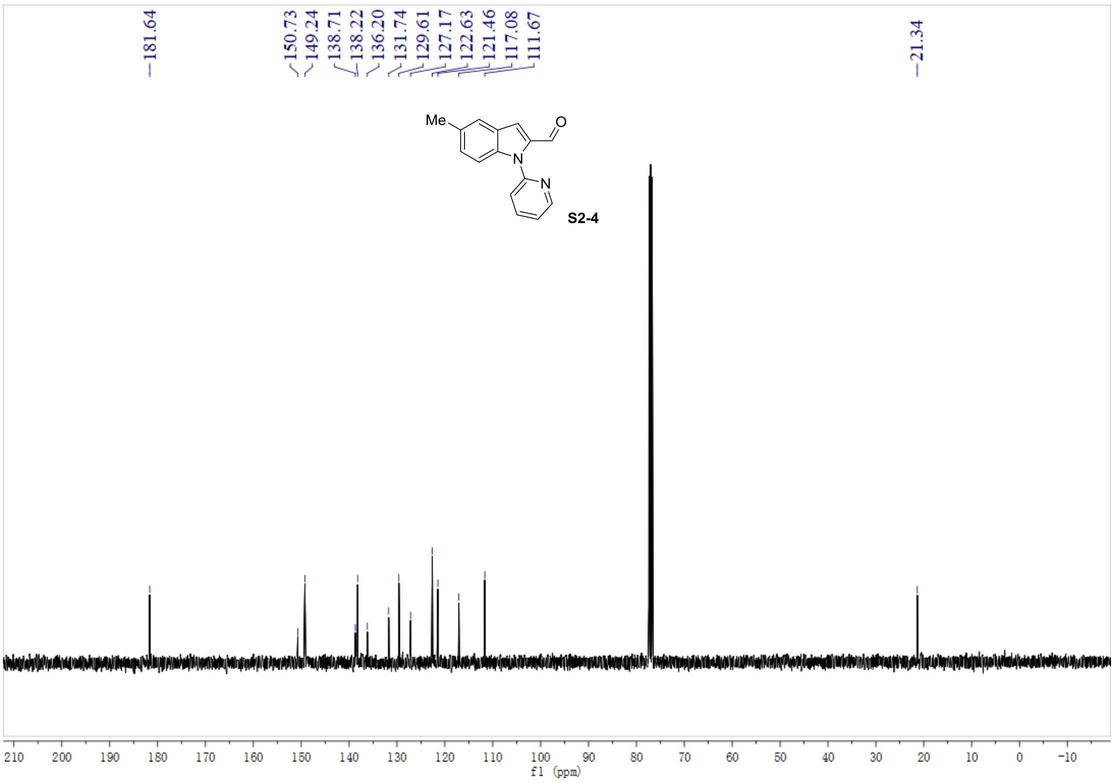

Supplementary Fig. 23 | ^{13}C NMR (101 MHz) of compound S2-4 (using $CDCl_3$ as solvent)

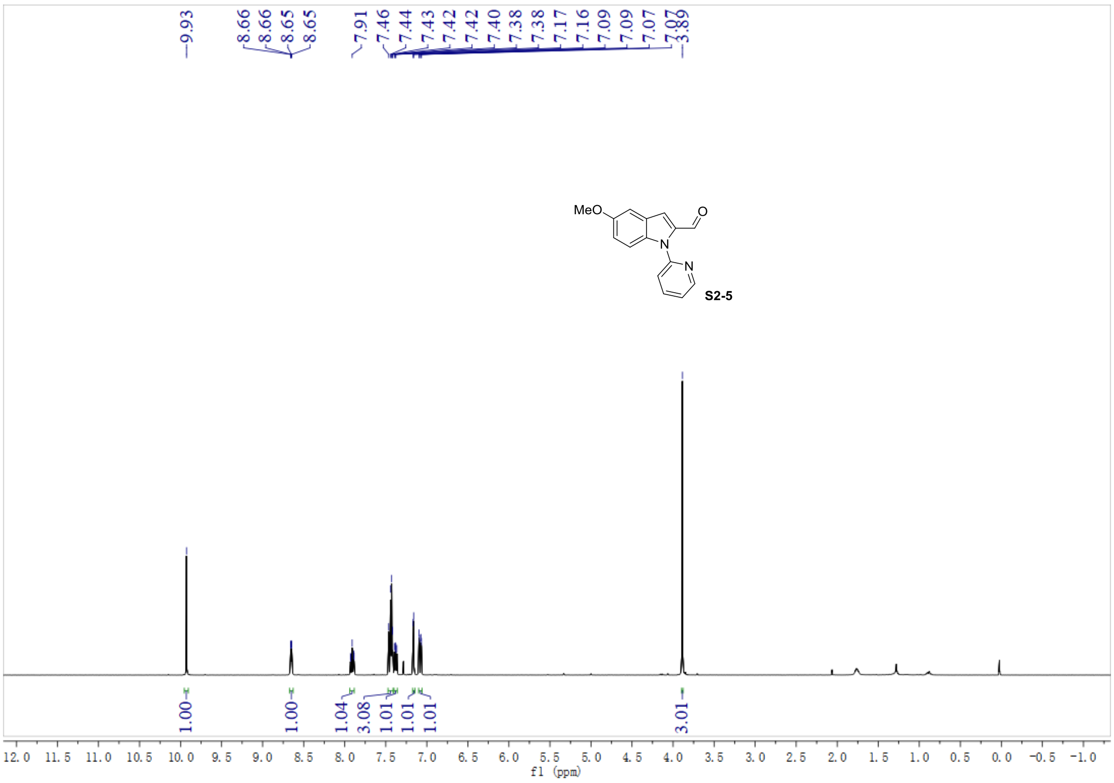

Supplementary Fig. 24 | ^{1}H NMR (400 MHz) of compound S2-5 (using $CDCl_3$ as solvent)

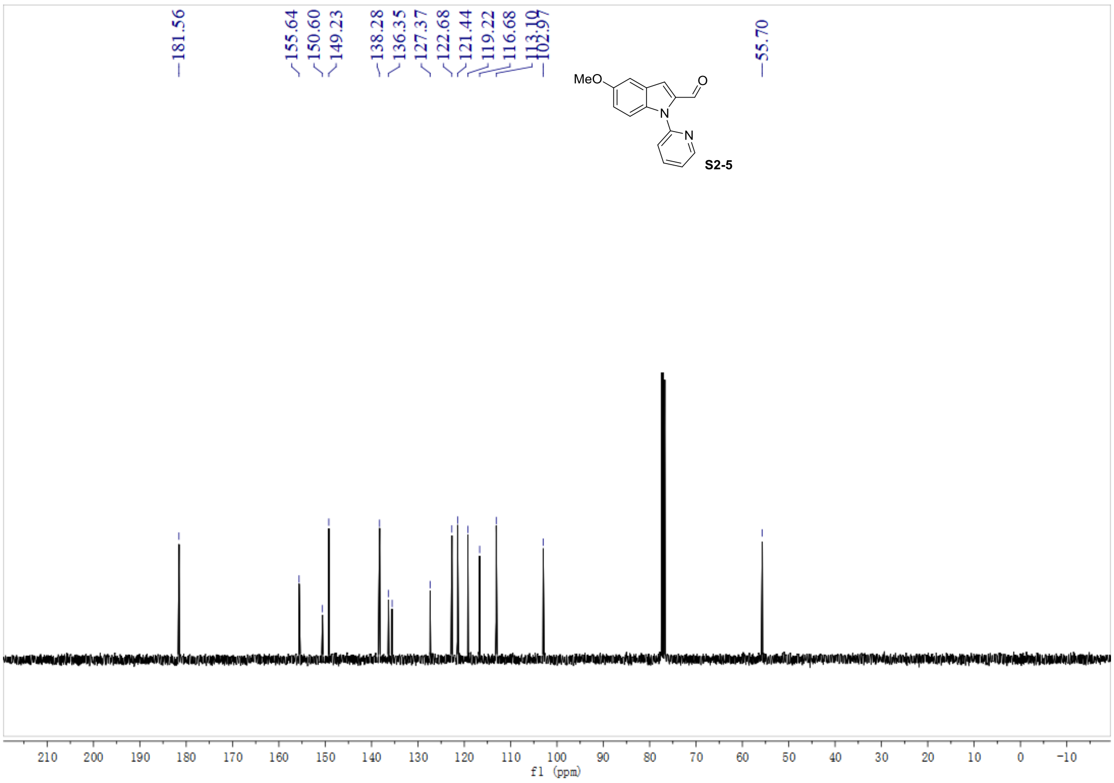

Supplementary Fig. 25 | ^{13}C NMR (101 MHz) of compound S2-5 (using $CDCl_3$ as solvent)

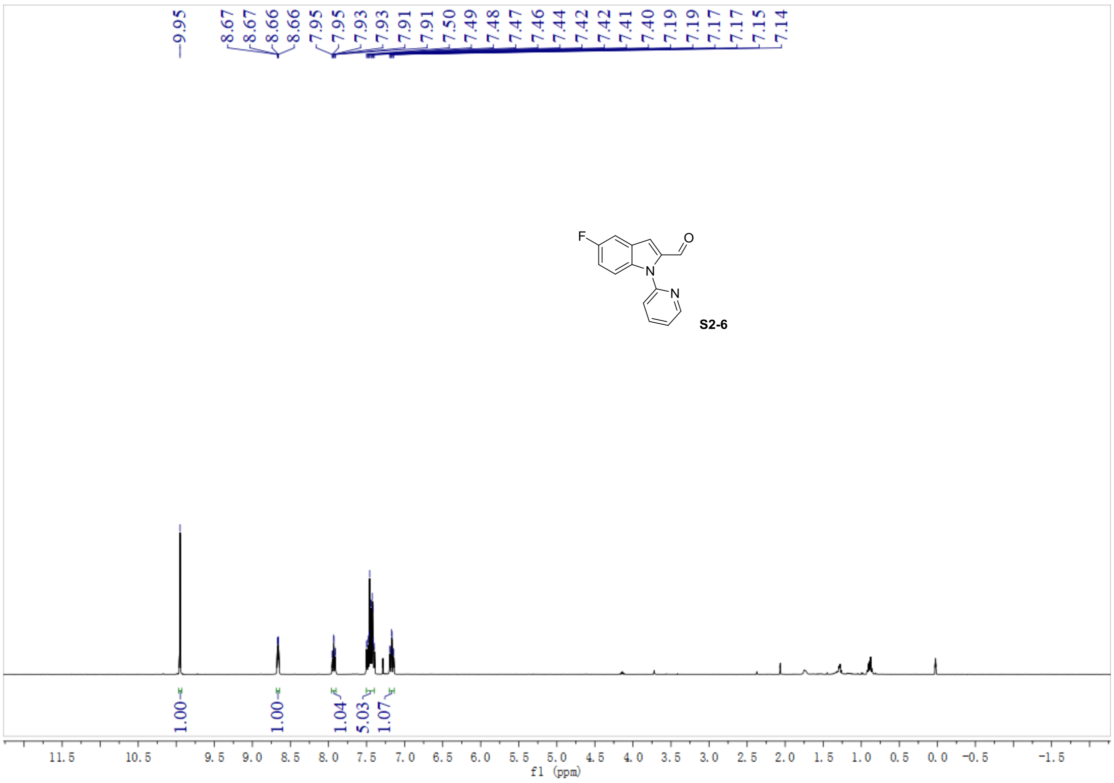

Supplementary Fig. 26 | ^{1}H NMR (400 MHz) of compound S2-6 (using $CDCl_3$ as solvent)

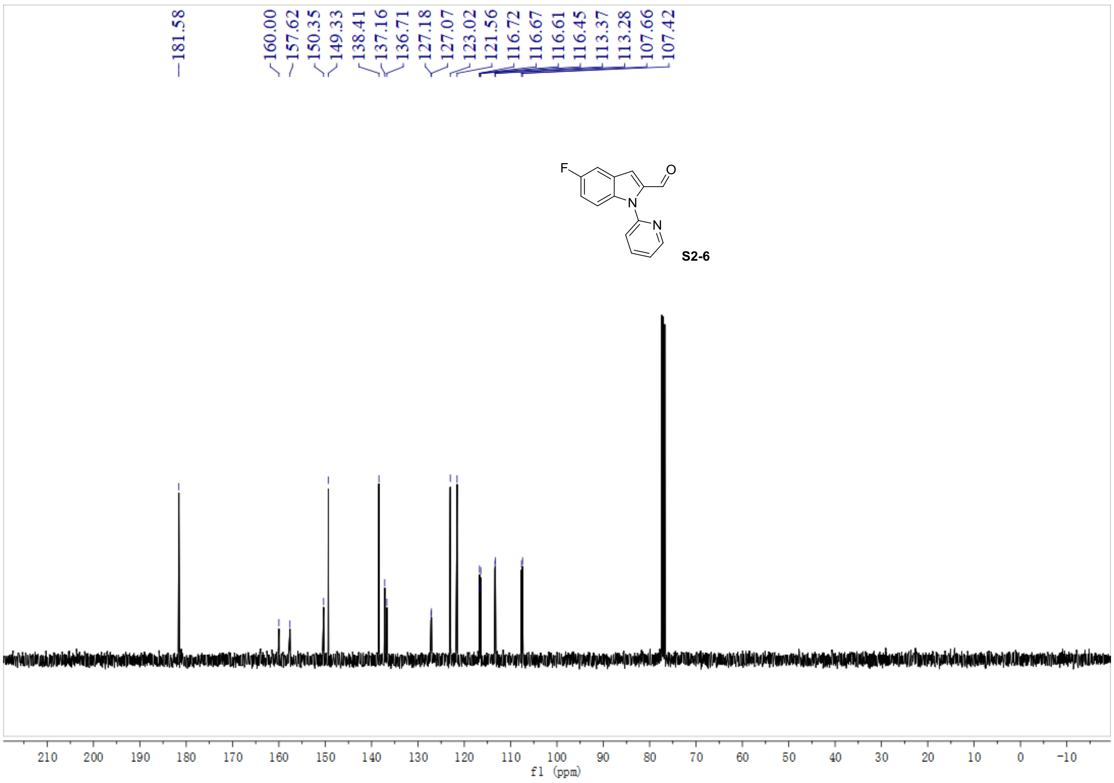

Supplementary Fig. 27 | ^{13}C NMR (101 MHz) of compound S2-6 (using $CDCl_3$ as solvent)

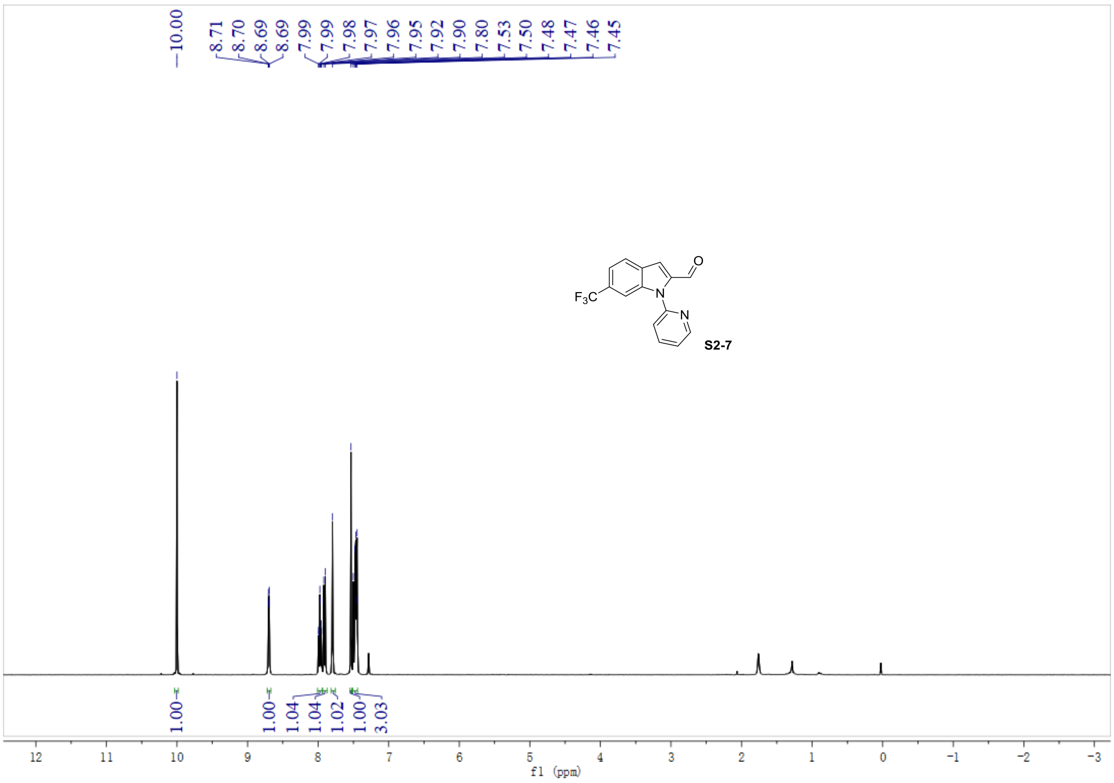

Supplementary Fig. 28 | ^{1}H NMR (400 MHz) of compound S2-7 (using $CDCl_3$ as solvent)

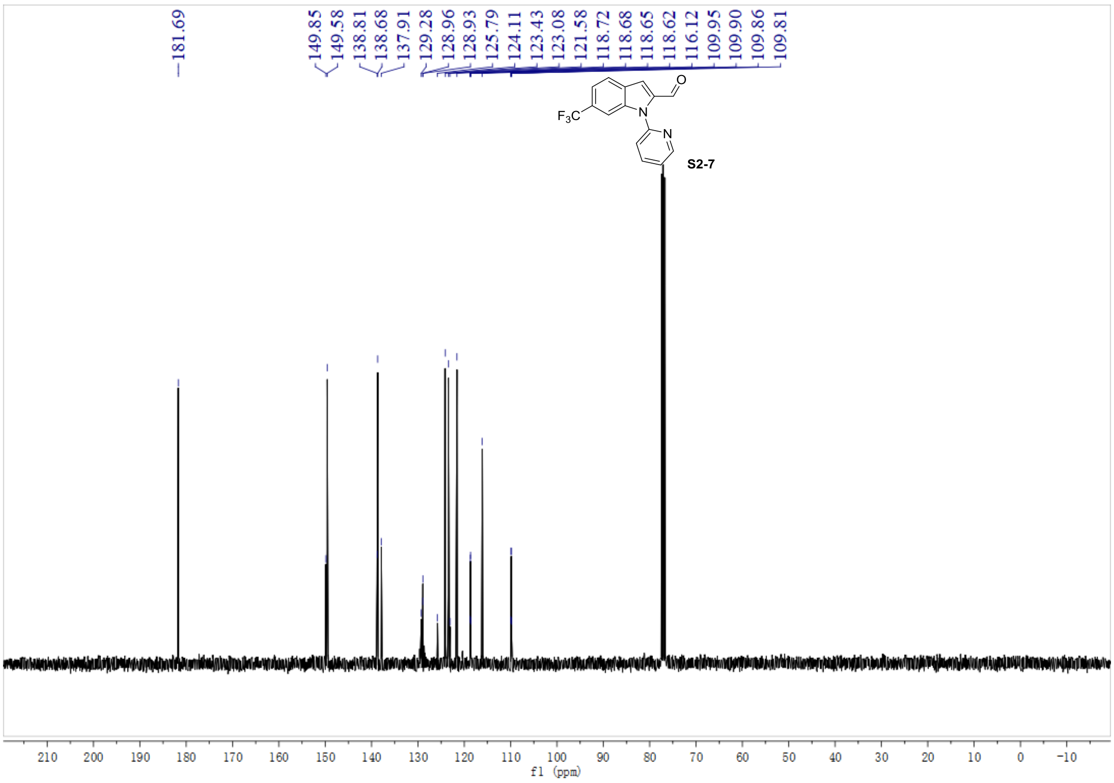

Supplementary Fig. 29 | ^{13}C NMR (101 MHz) of compound S2-7 (using $CDCl_3$ as solvent)

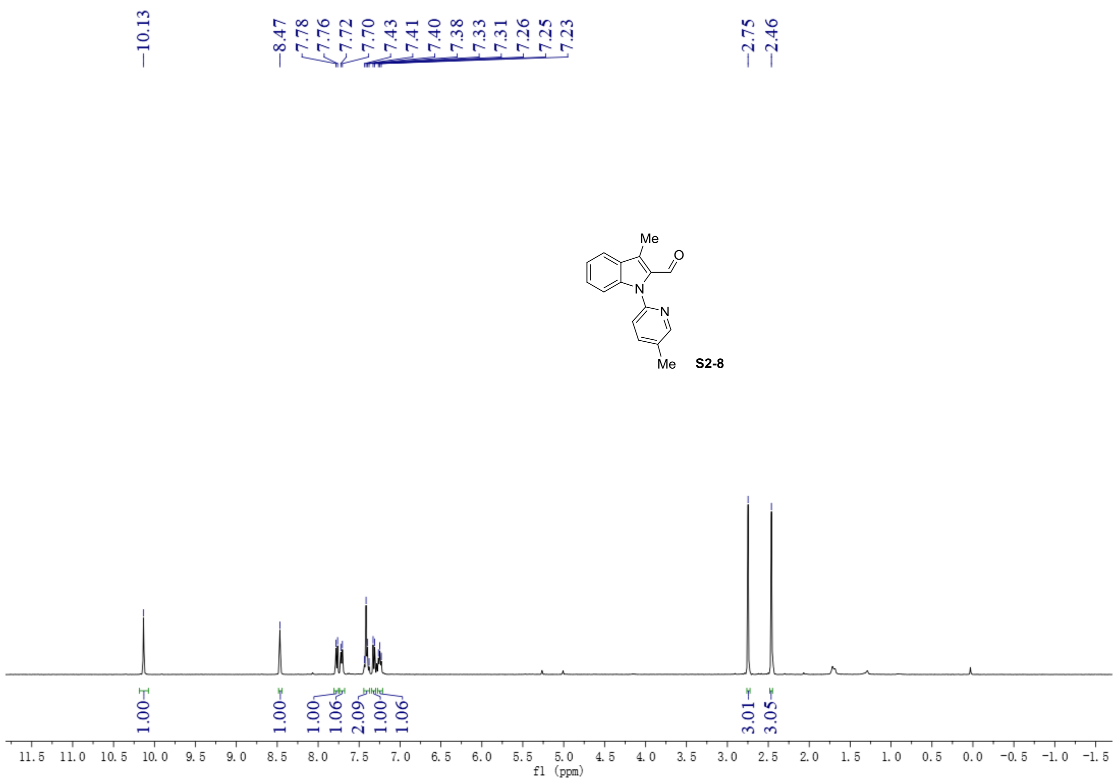

Supplementary Fig. 30 | ^{1}H NMR (400 MHz) of compound S2-8 (using $CDCl_3$ as solvent)

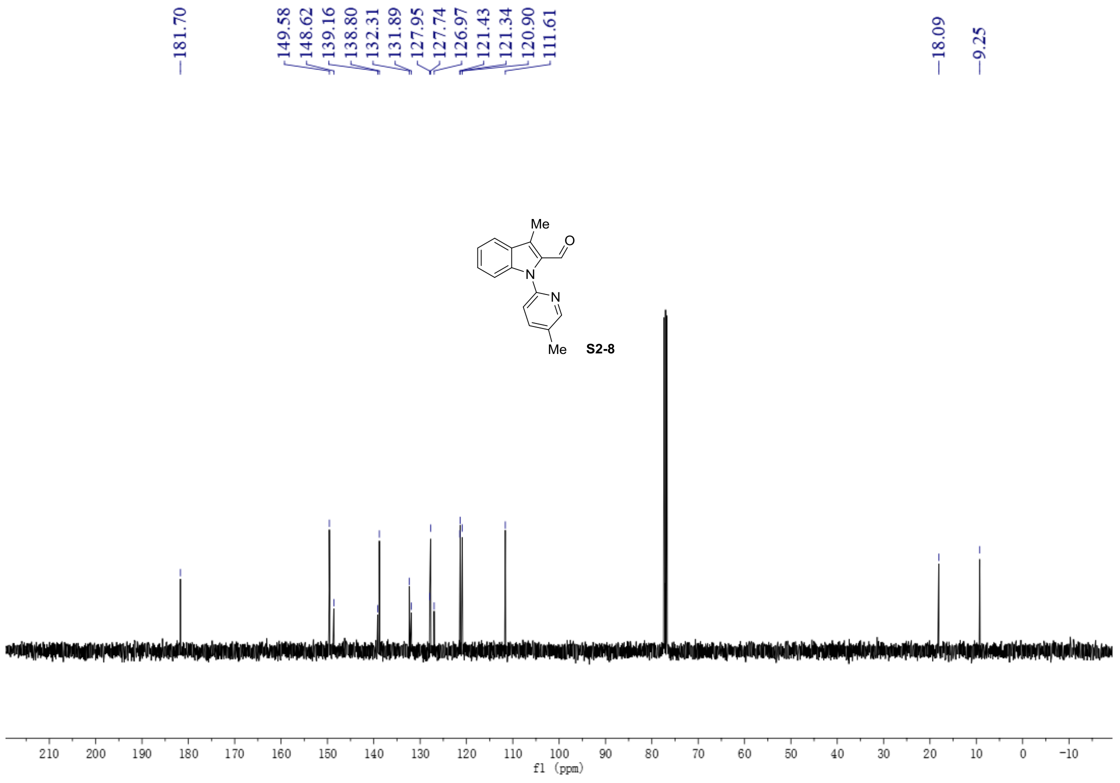

Supplementary Fig. 31 | ^{13}C NMR (101 MHz) of compound S2-8 (using $CDCl_3$ as solvent)

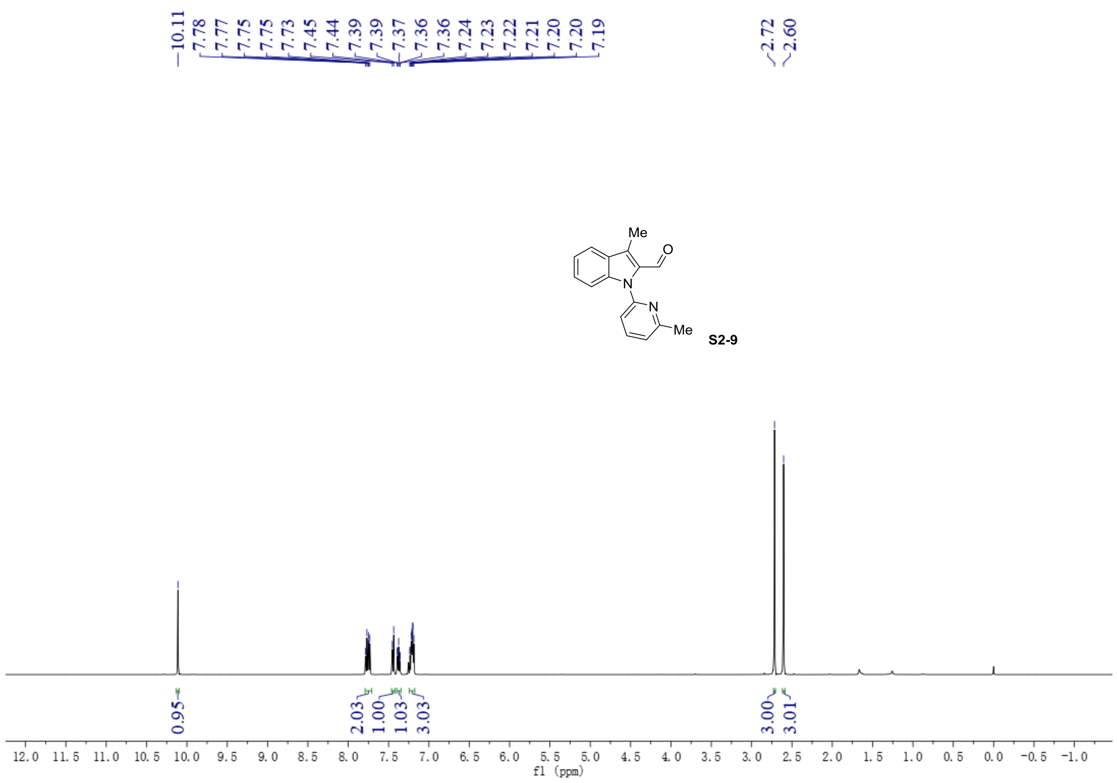

Supplementary Fig. 32 | ^{1}H NMR (500 MHz) of compound S2-9 (using $CDCl_3$ as solvent)

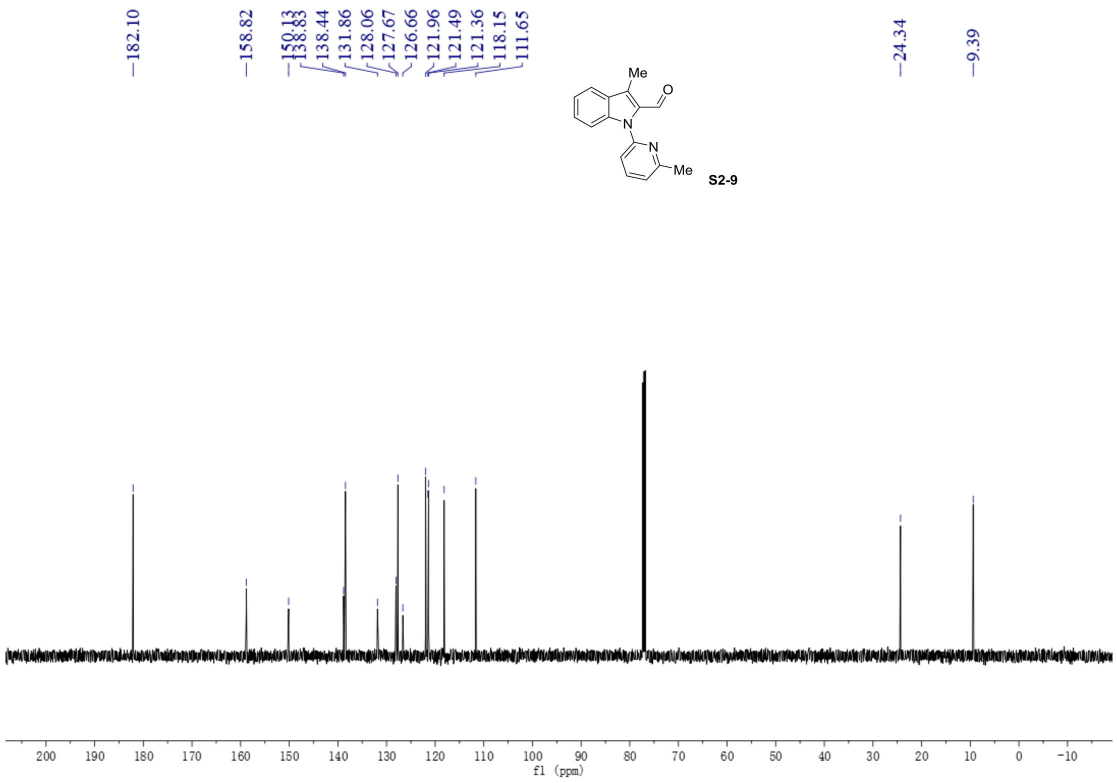

Supplementary Fig. 33 | ^{13}C NMR (126 MHz) of compound S2-9 (using $CDCl_3$ as solvent)

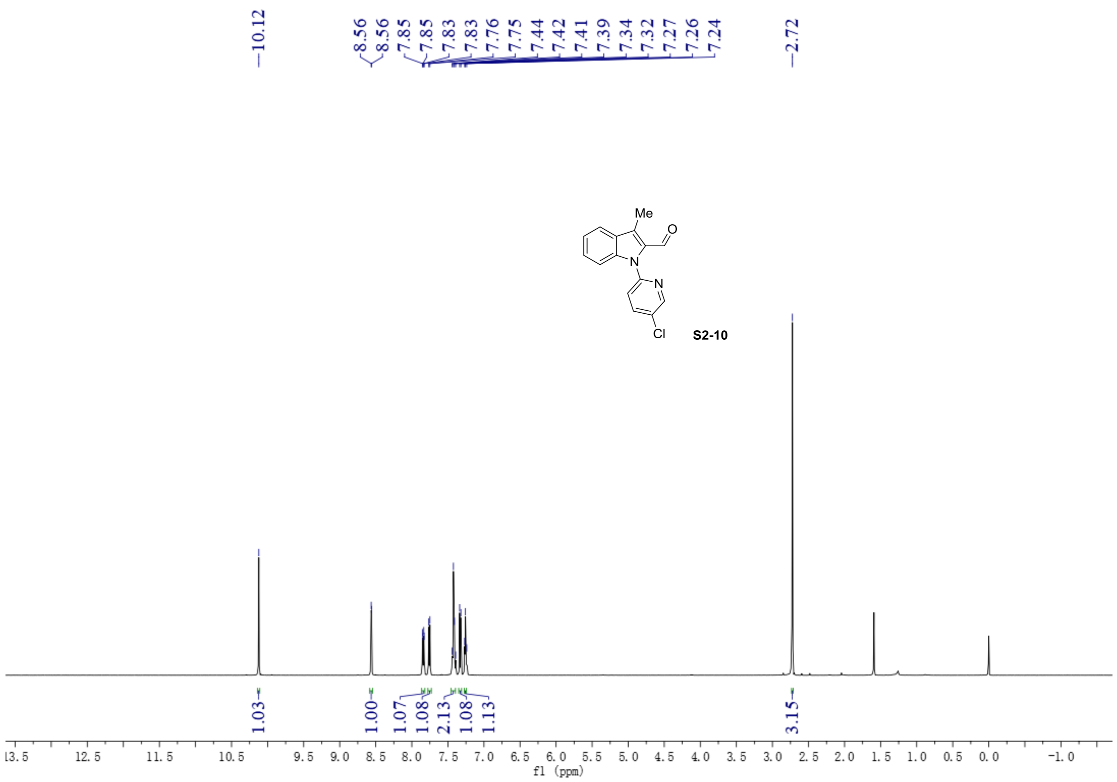

Supplementary Fig. 34 | ^{1}H NMR (500 MHz) of compound S2-10 (using $CDCl_3$ as solvent)

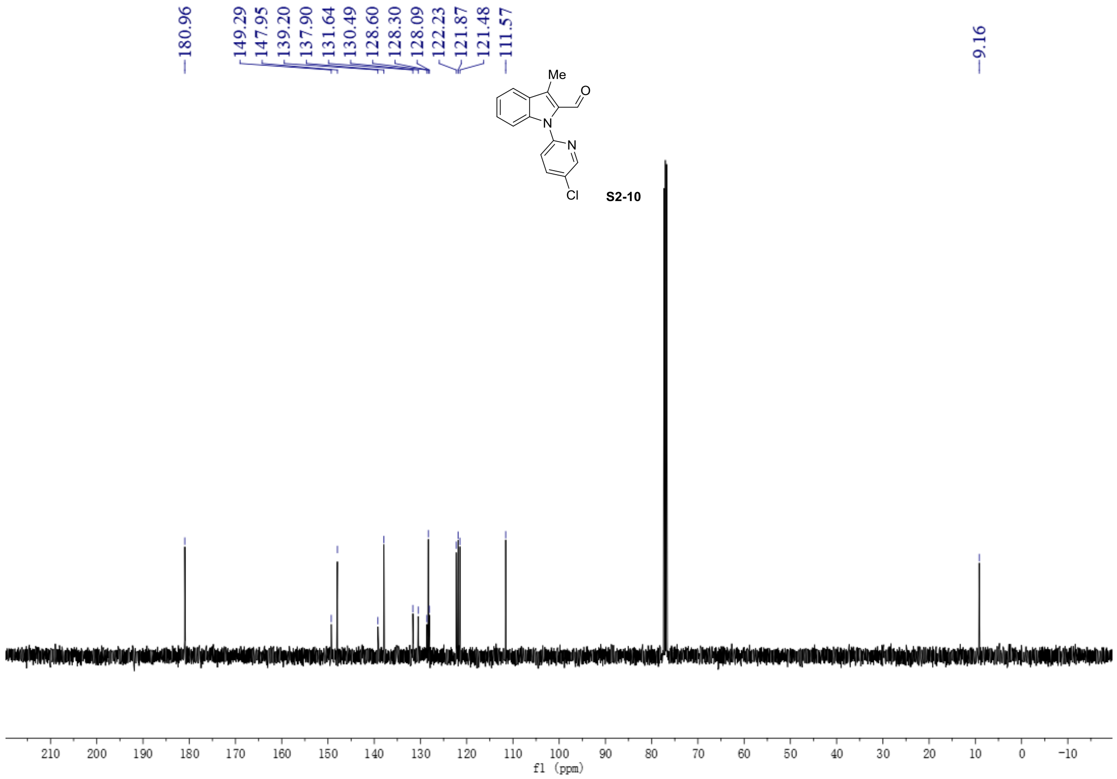

Supplementary Fig. 35 | ^{1}H NMR (126 MHz) of compound S2-10 (using $CDCl_3$ as solvent)

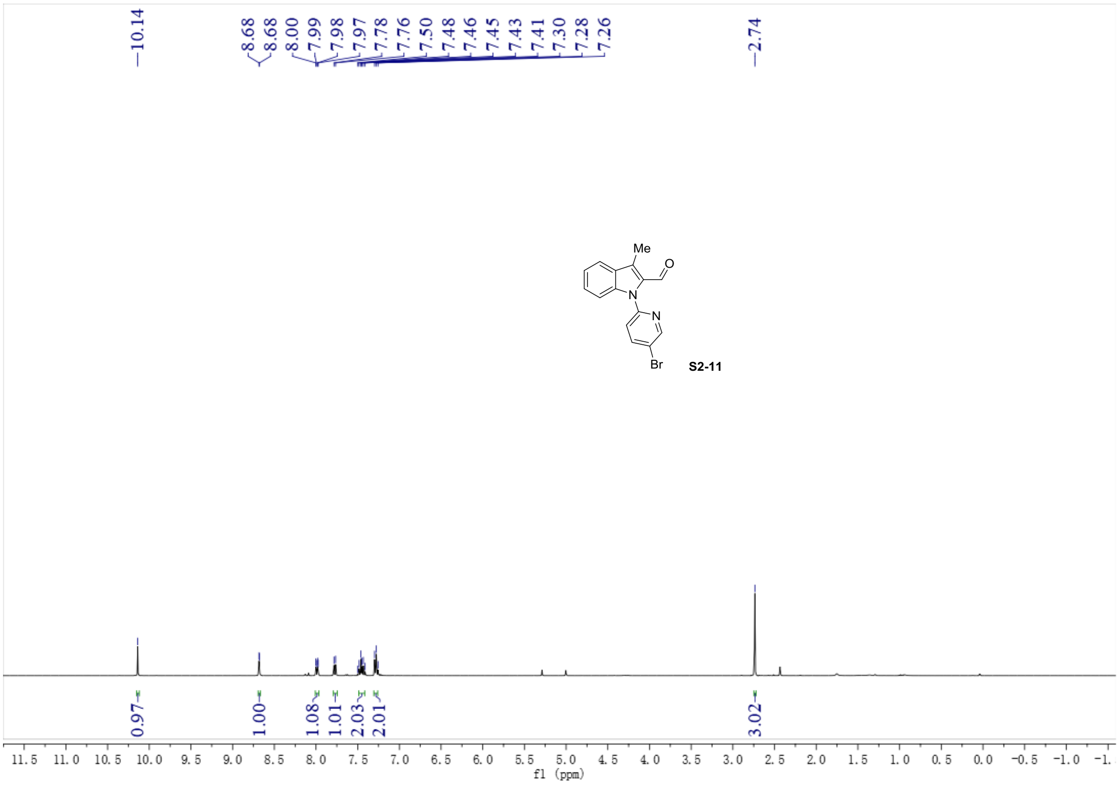

Supplementary Fig. 36 | ^{1}H NMR (400 MHz) of compound S2-11 (using $CDCl_3$ as solvent)

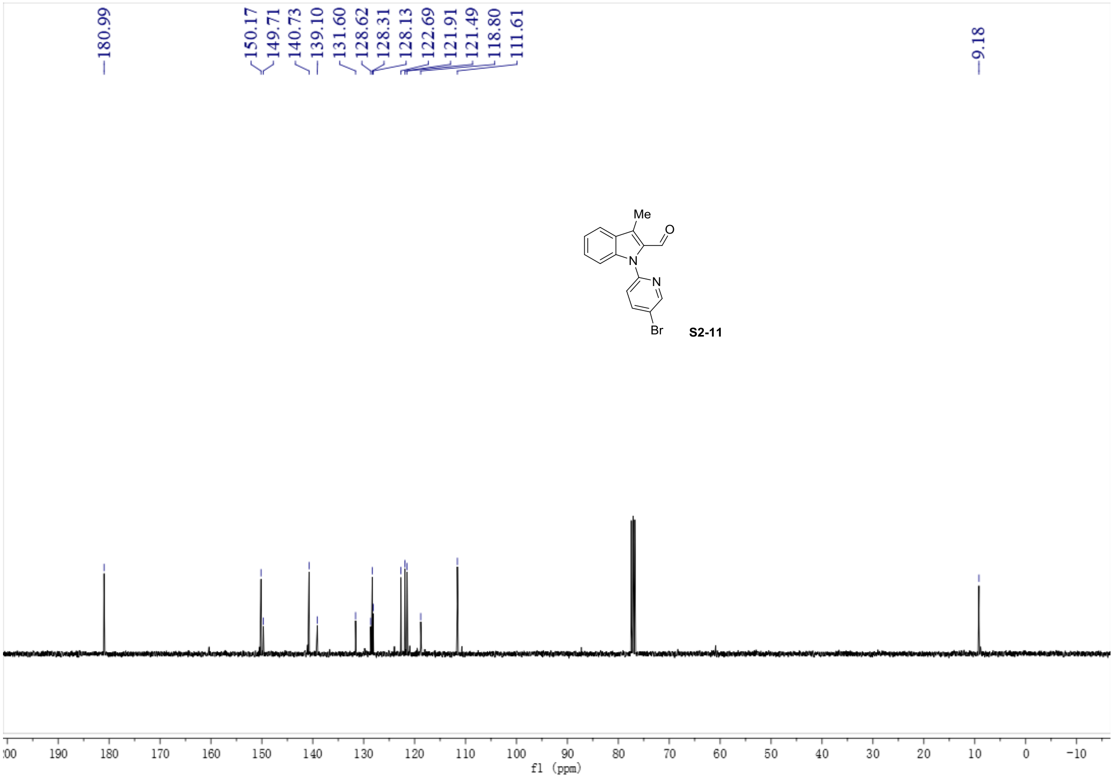

Supplementary Fig. 37 | ^{13}C NMR (101 MHz) of compound S2-11 (using $CDCl_3$ as solvent)

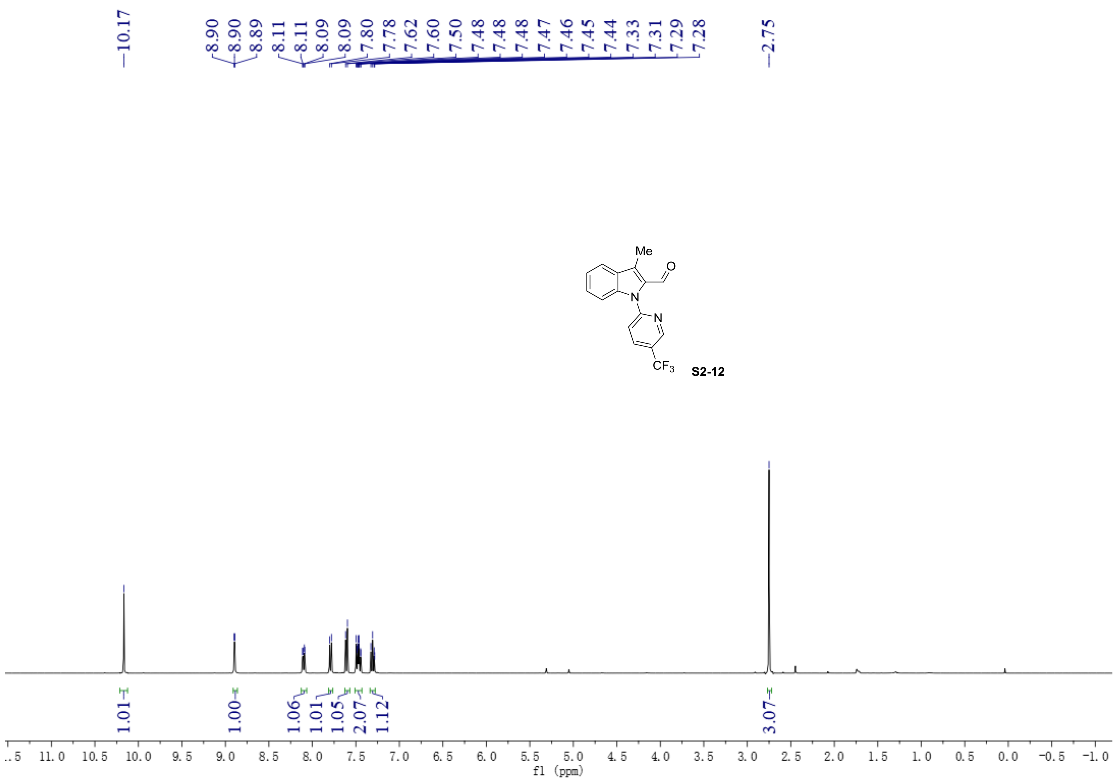

Supplementary Fig. 38 | ^{1}H NMR (400 MHz) of compound S2-12 (using $CDCl_3$ as solvent)

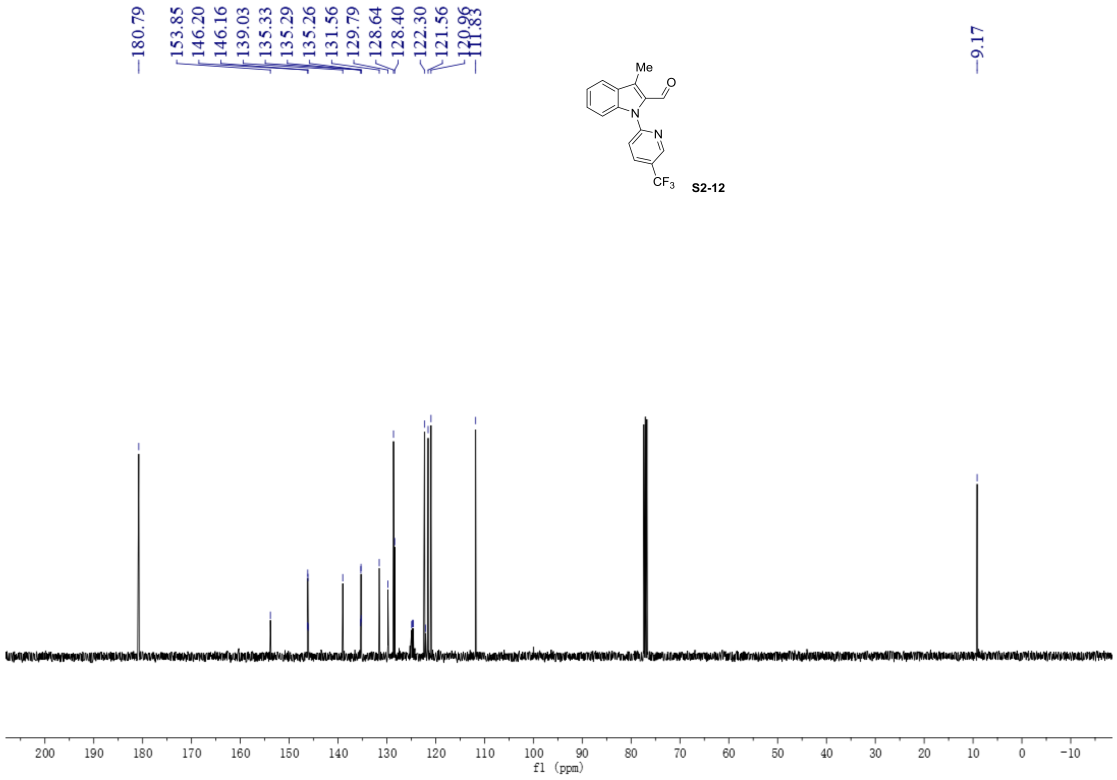

Supplementary Fig. 39 | ^{13}C NMR (101 MHz) of compound S2-12 (using $CDCl_3$ as solvent)

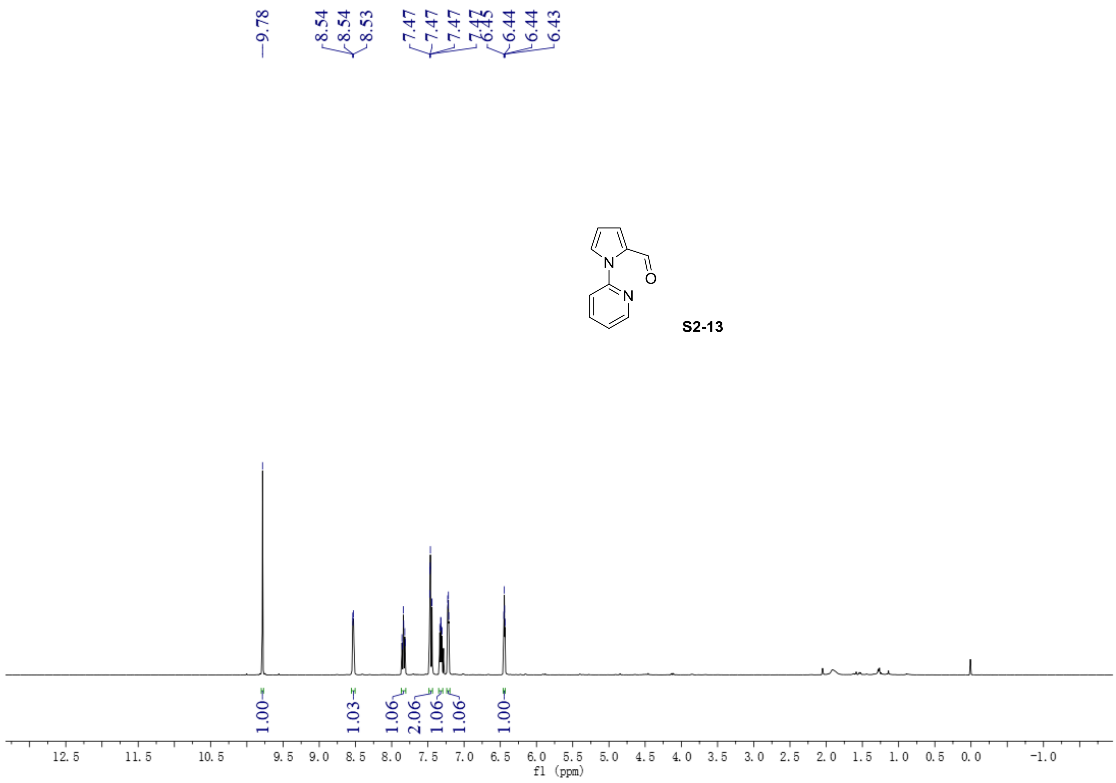

Supplementary Fig. 40 | ^{1}H NMR (400 MHz) of compound S2-13 (using $CDCl_3$ as solvent)

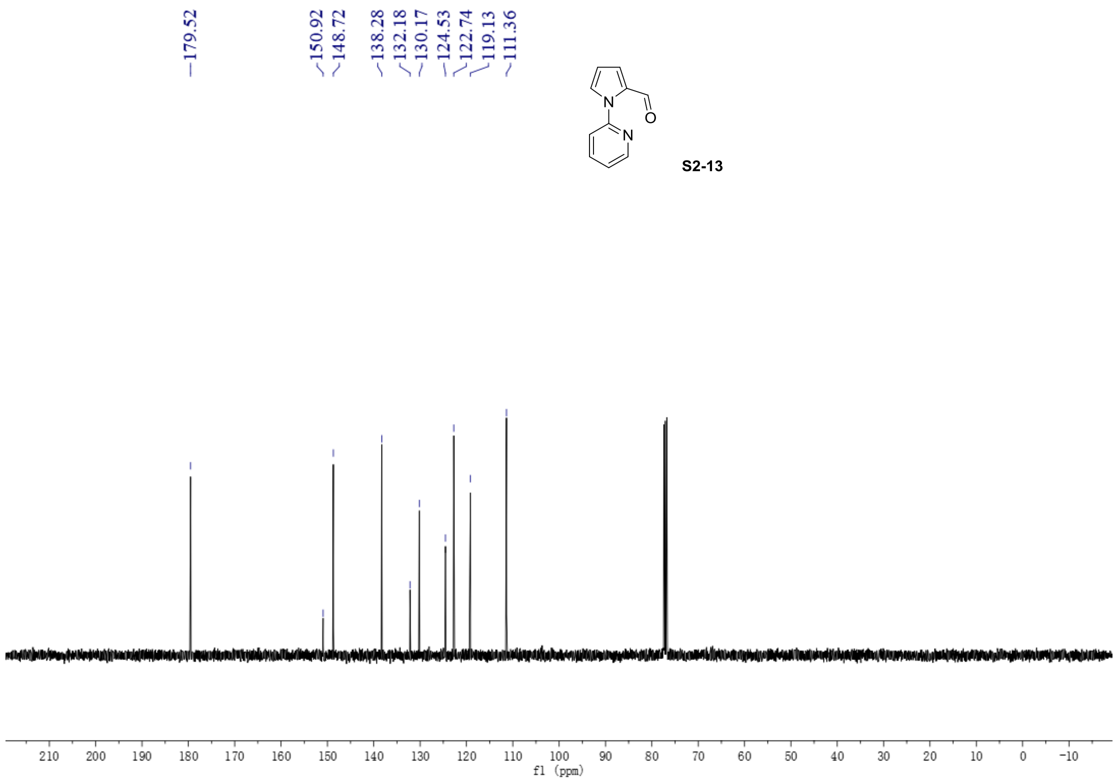

Supplementary Fig. 41 | ^{1}H NMR (101 MHz) of compound S2-13 (using $CDCl_3$ as solvent)

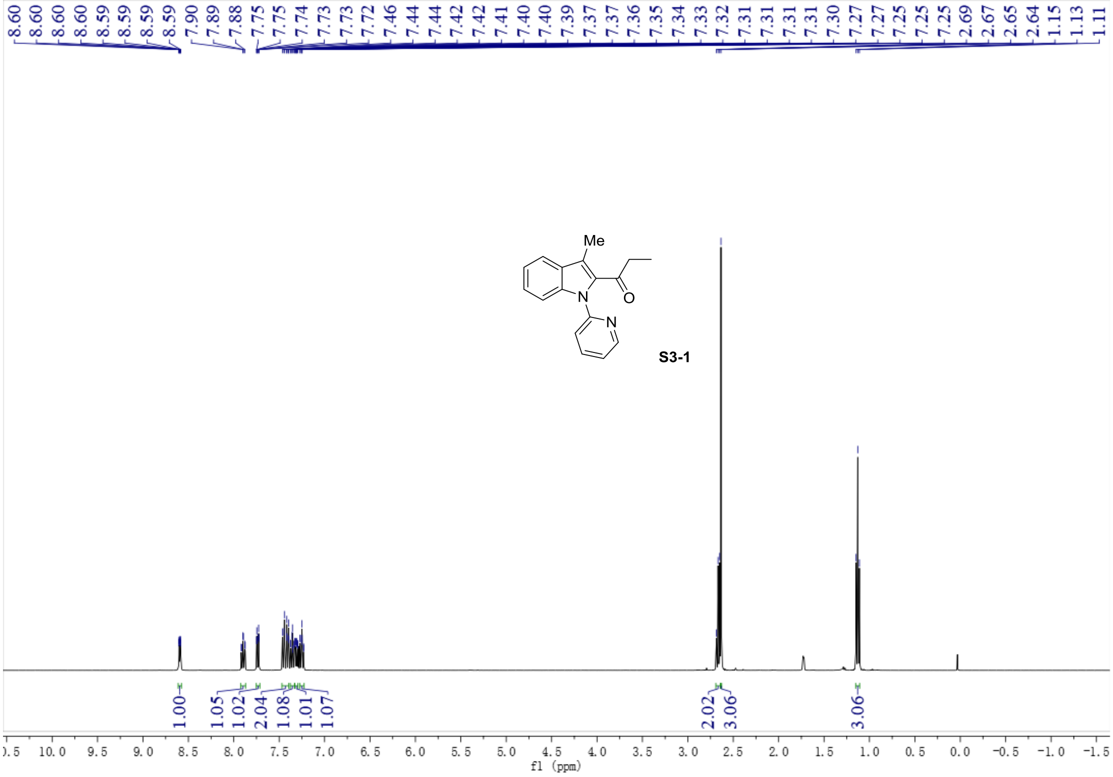

Supplementary Fig. 42 | ^{1}H NMR (400 MHz) of compound S3-1 (using $CDCl_3$ as solvent)

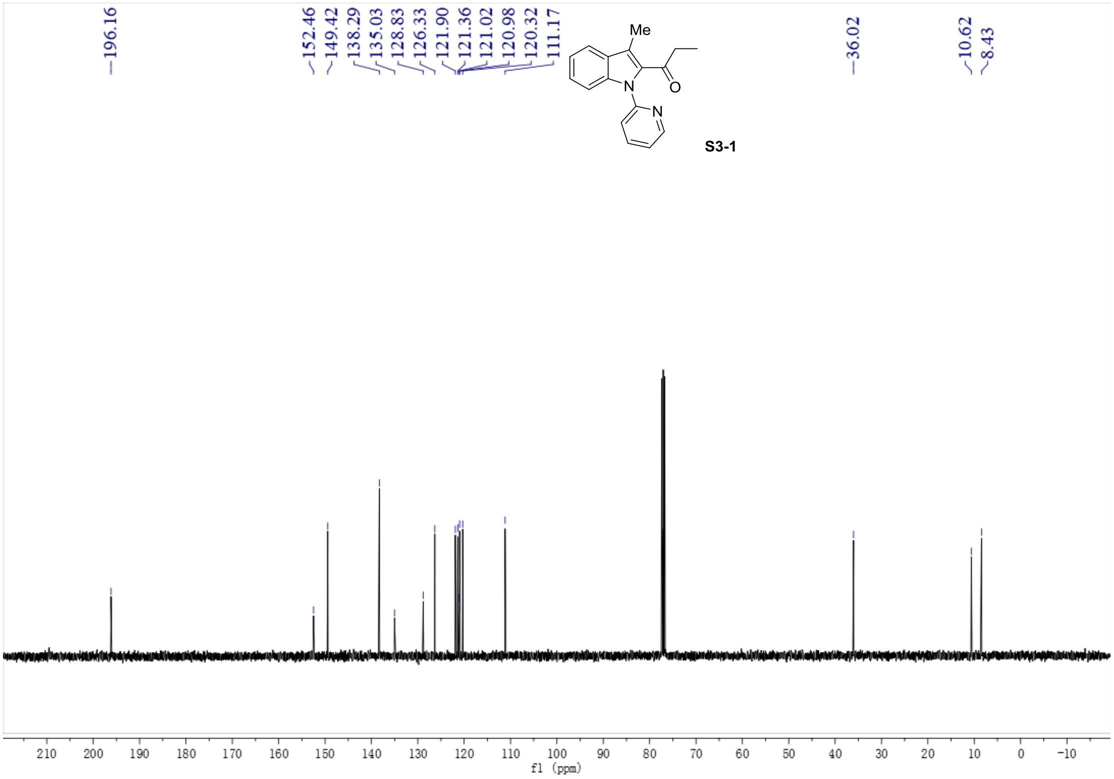

Supplementary Fig. 43 | ^{1}H NMR (101 MHz) of compound S3-1 (using $CDCl_3$ as solvent)

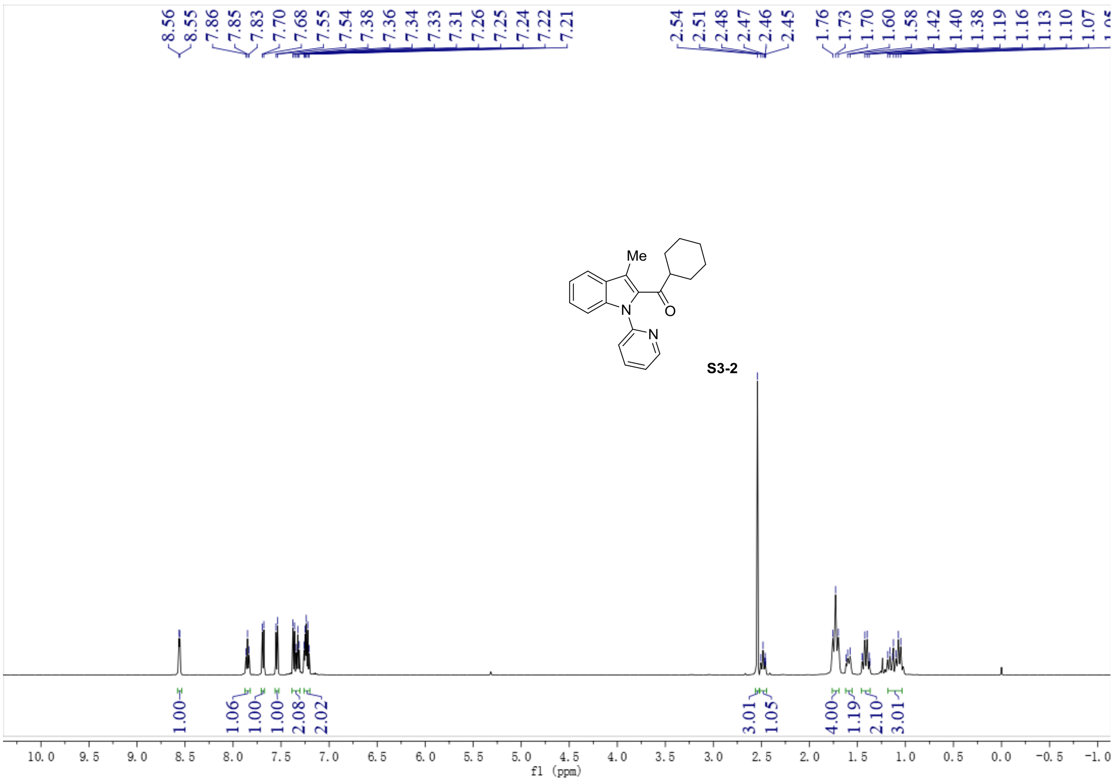

Supplementary Fig. 44 | ^{1}H NMR (400 MHz) of compound S3-2 (using $CDCl_3$ as solvent)

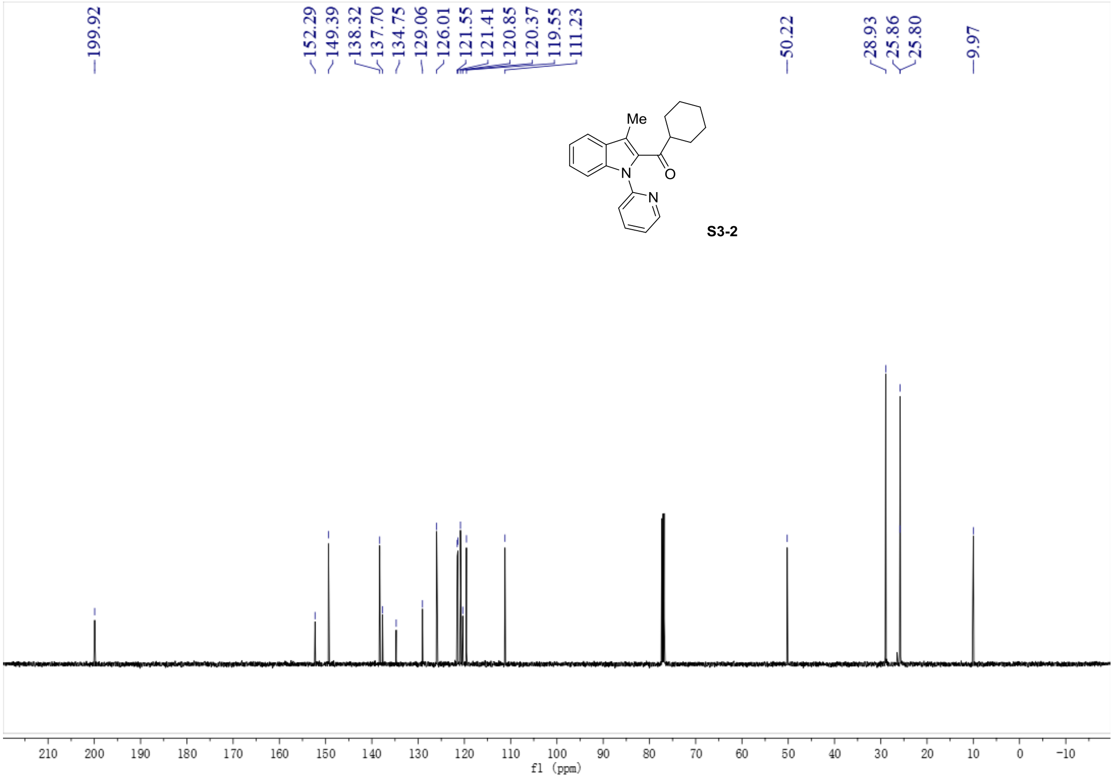

Supplementary Fig. 45 | ^{13}C NMR (101 MHz) of compound S3-2 (using $CDCl_3$ as solvent)

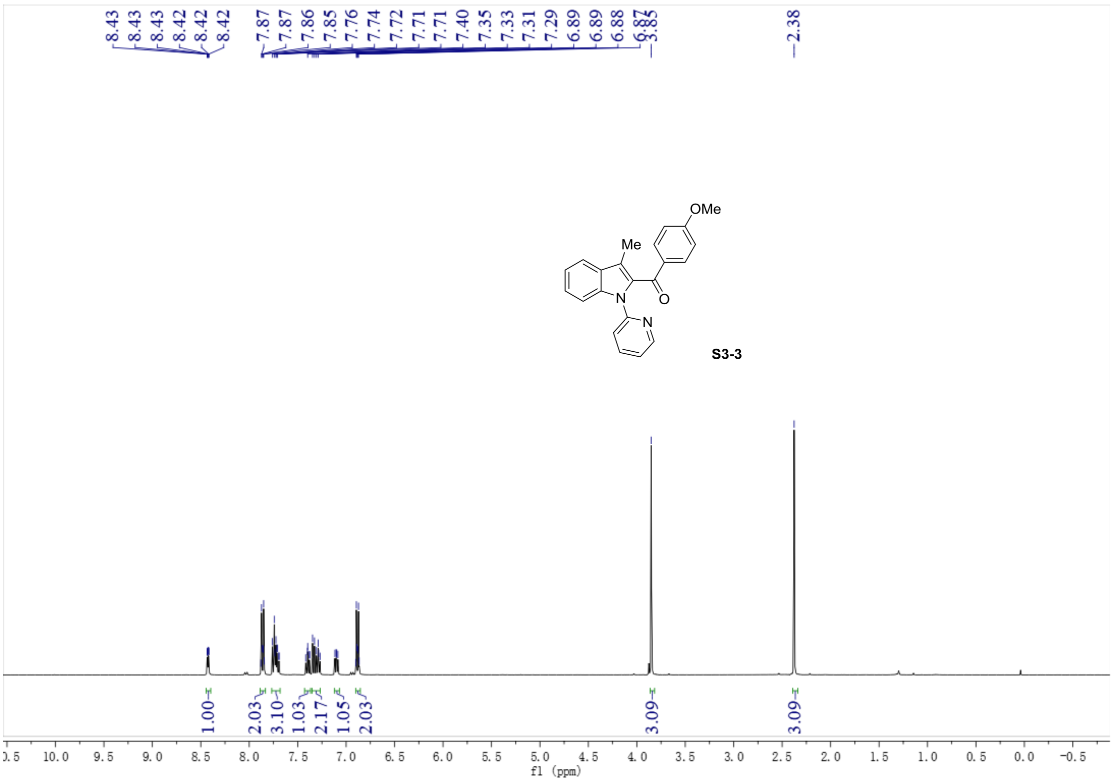

Supplementary Fig. 46 | ^{1}H NMR (400 MHz) of compound S3-3 (using $CDCl_3$ as solvent)

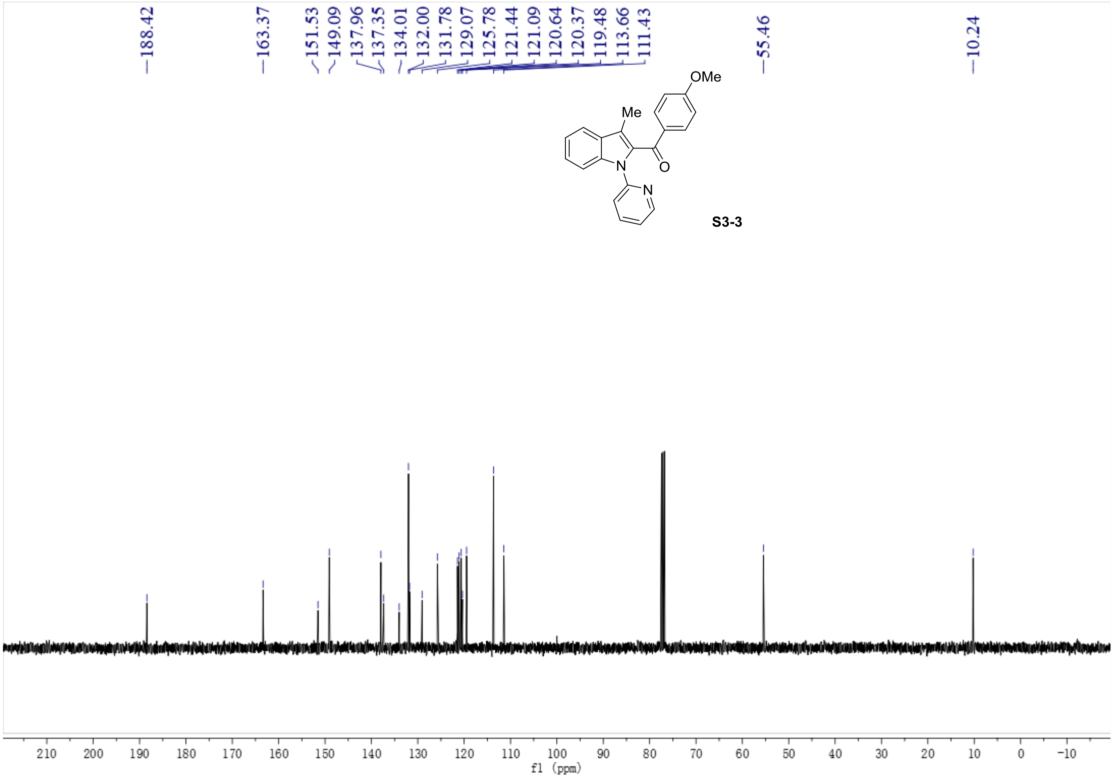

Supplementary Fig. 47 | ^{13}C NMR (101 MHz) of compound S3-3 (using $CDCl_3$ as solvent)

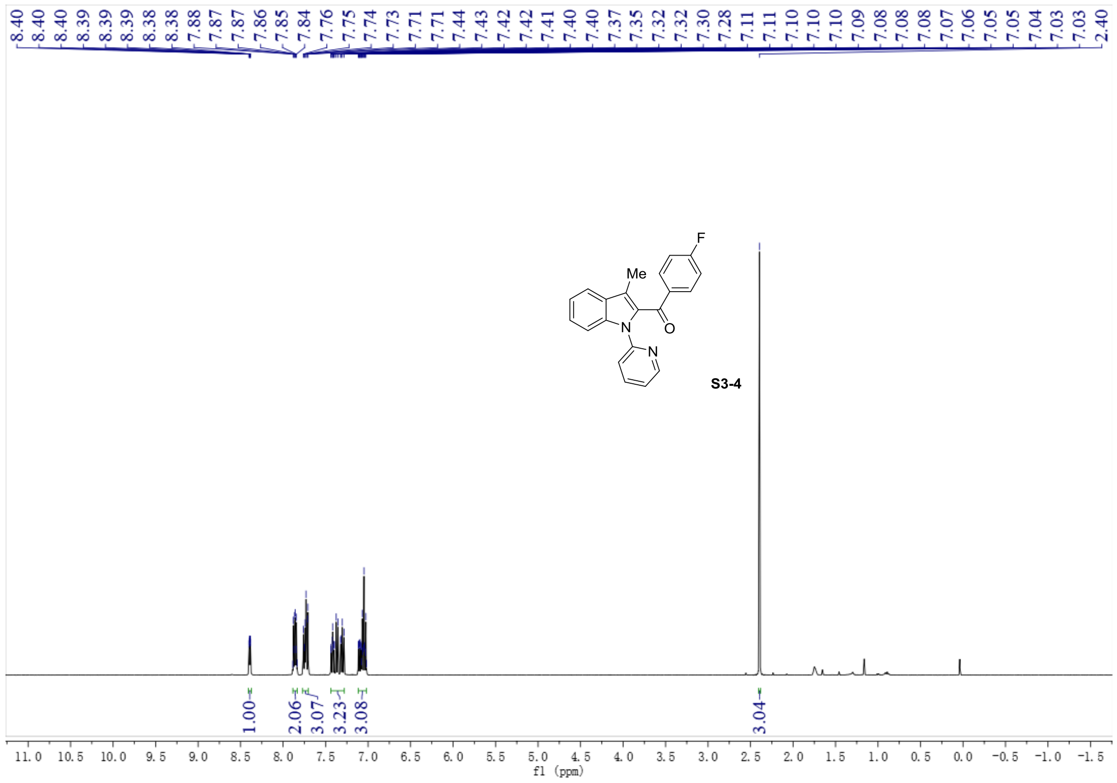

Supplementary Fig. 48 | ^{1}H NMR (400 MHz) of compound S3-4 (using $CDCl_3$ as solvent)

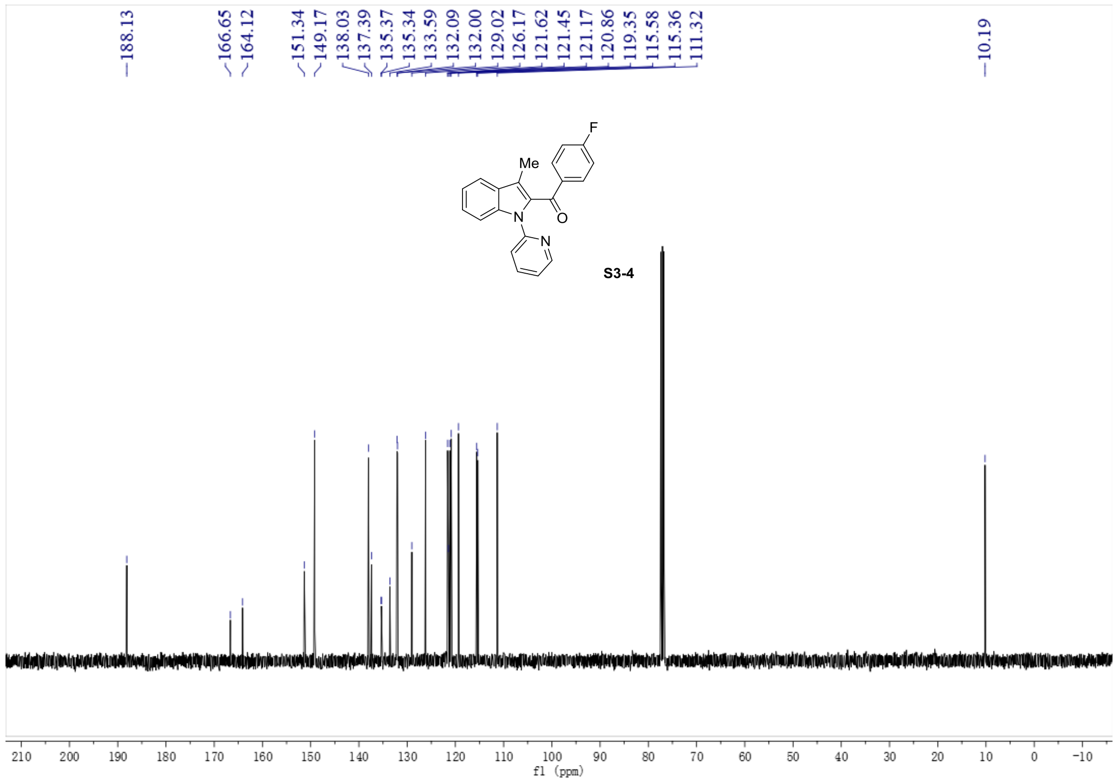

Supplementary Fig. 49 | ^{13}C NMR (101 MHz) of compound S3-4 (using $CDCl_3$ as solvent)

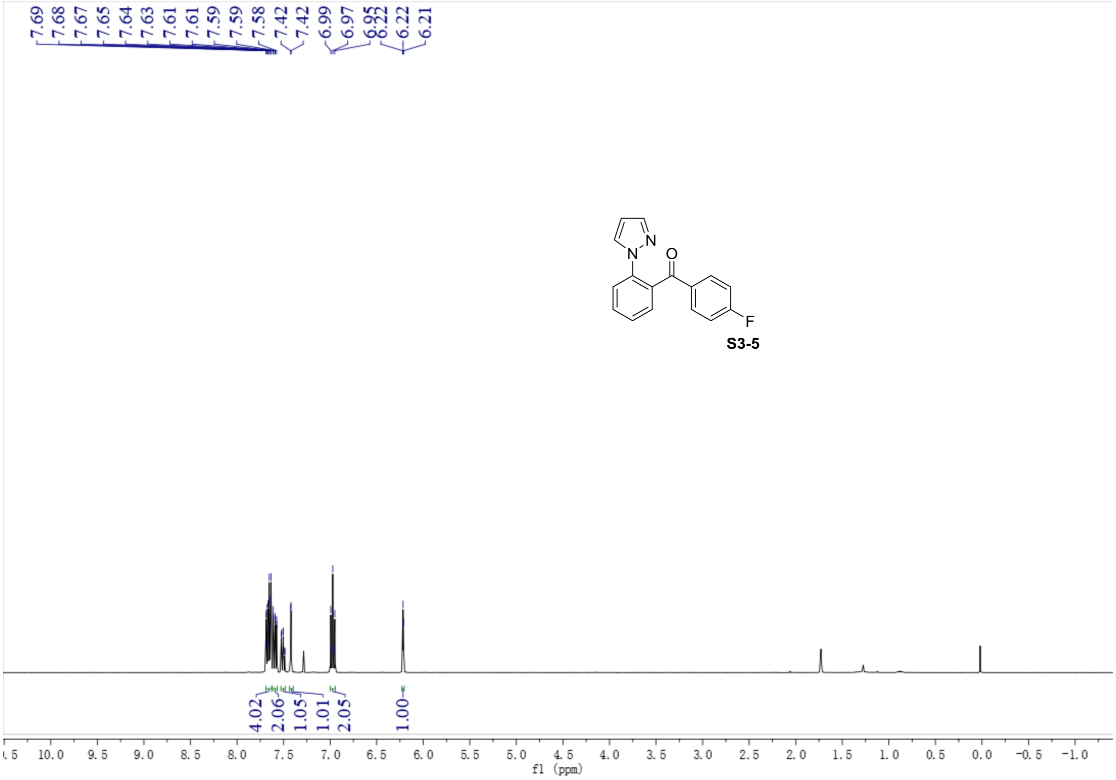

Supplementary Fig. 50 | ^{1}H NMR (400 MHz) of compound S3-5 (using $CDCl_3$ as solvent)

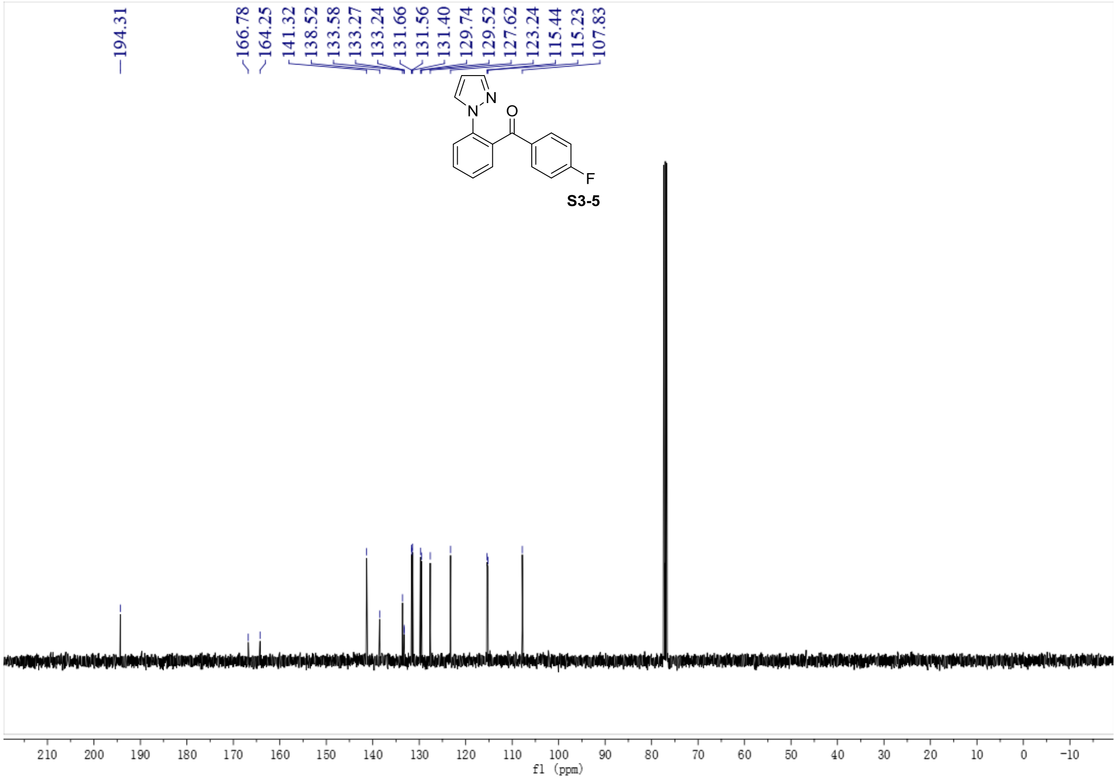

Supplementary Fig. 51 | ^{13}C NMR (101 MHz) of compound S3-5 (using $CDCl_3$ as solvent)

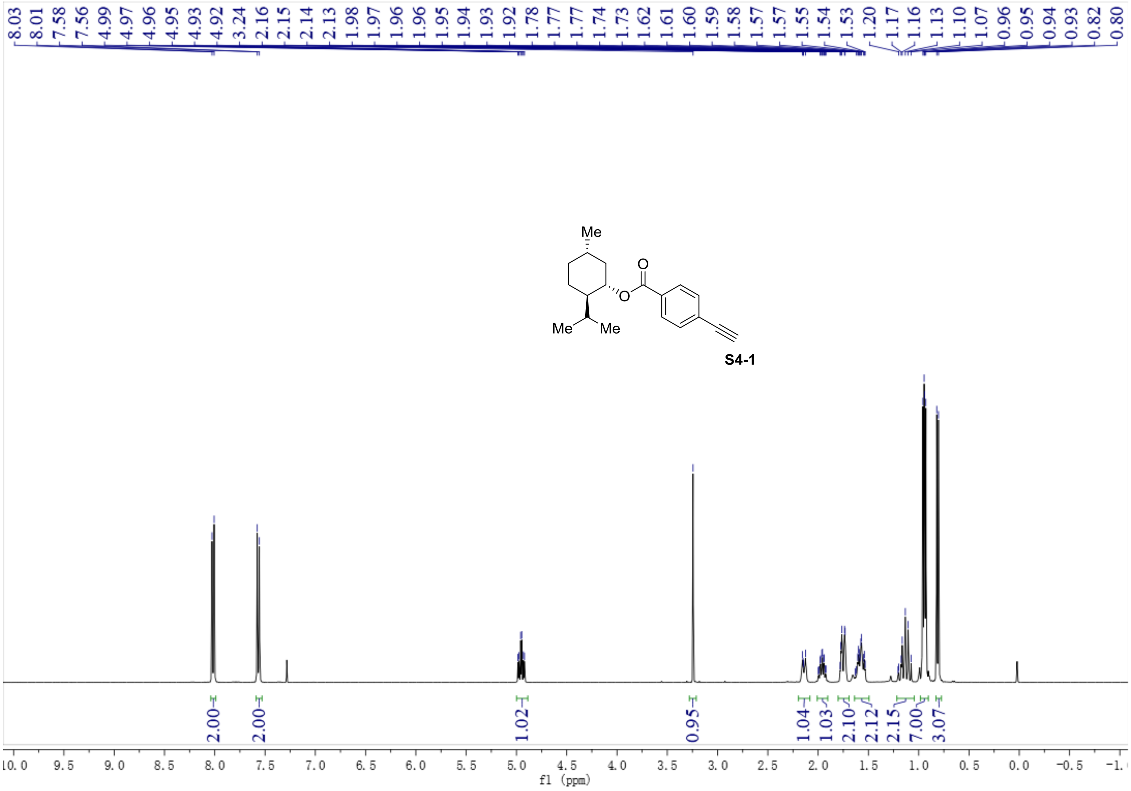

Supplementary Fig. 52 | ^{1}H NMR (400 MHz) of compound S4-1 (using $CDCl_3$ as solvent)

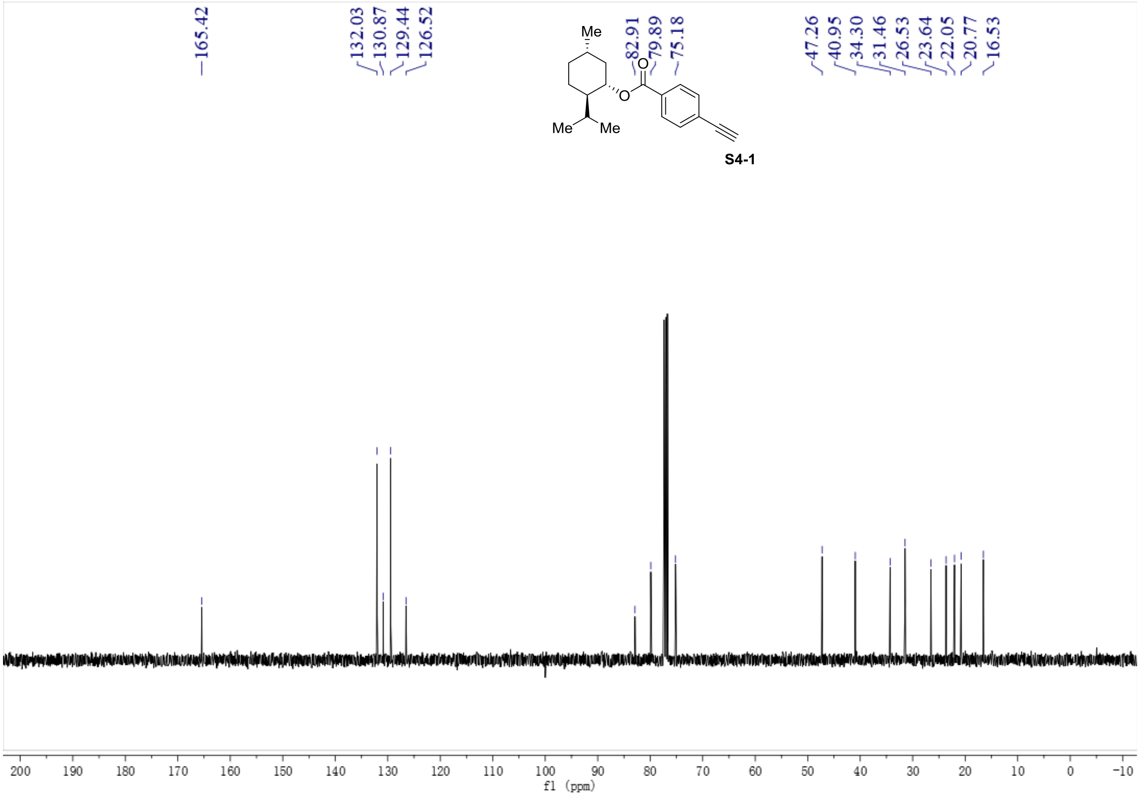

Supplementary Fig. 53 | ^{13}C NMR (101 MHz) of compound S4-1 (using $CDCl_3$ as solvent)

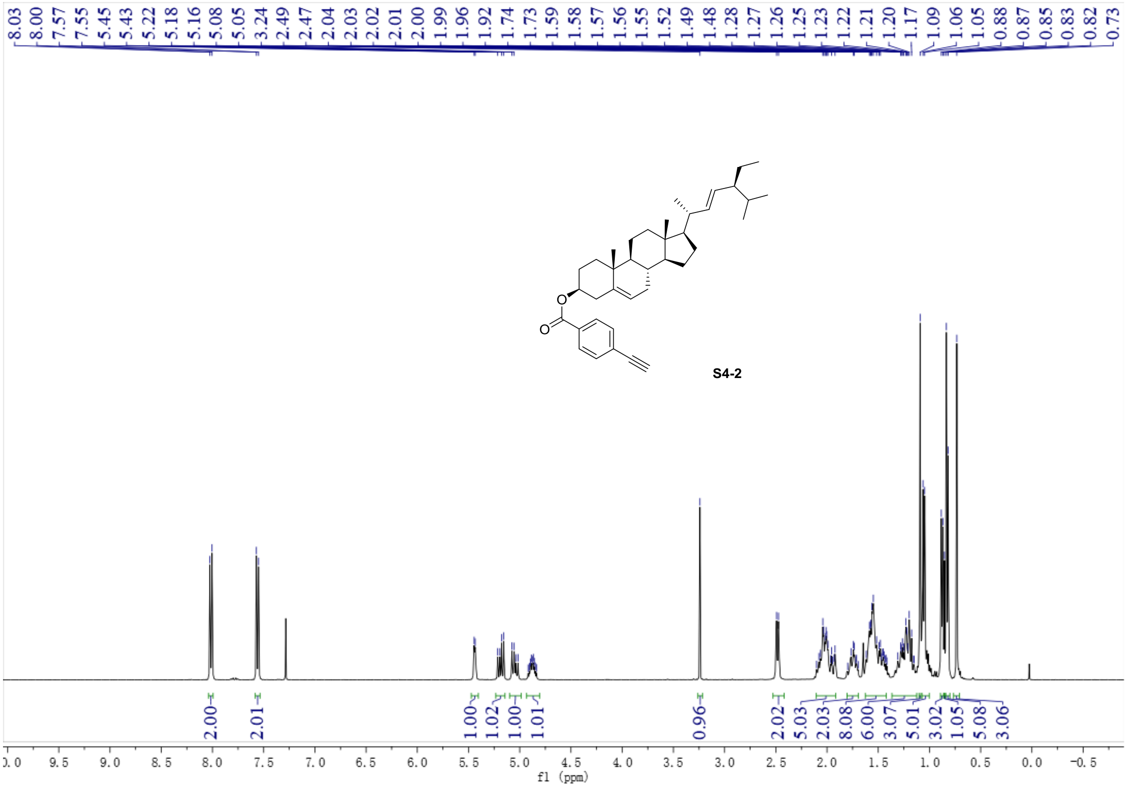

Supplementary Fig. 54 | ^{1}H NMR (400 MHz) of compound S4-2 (using $CDCl_3$ as solvent)

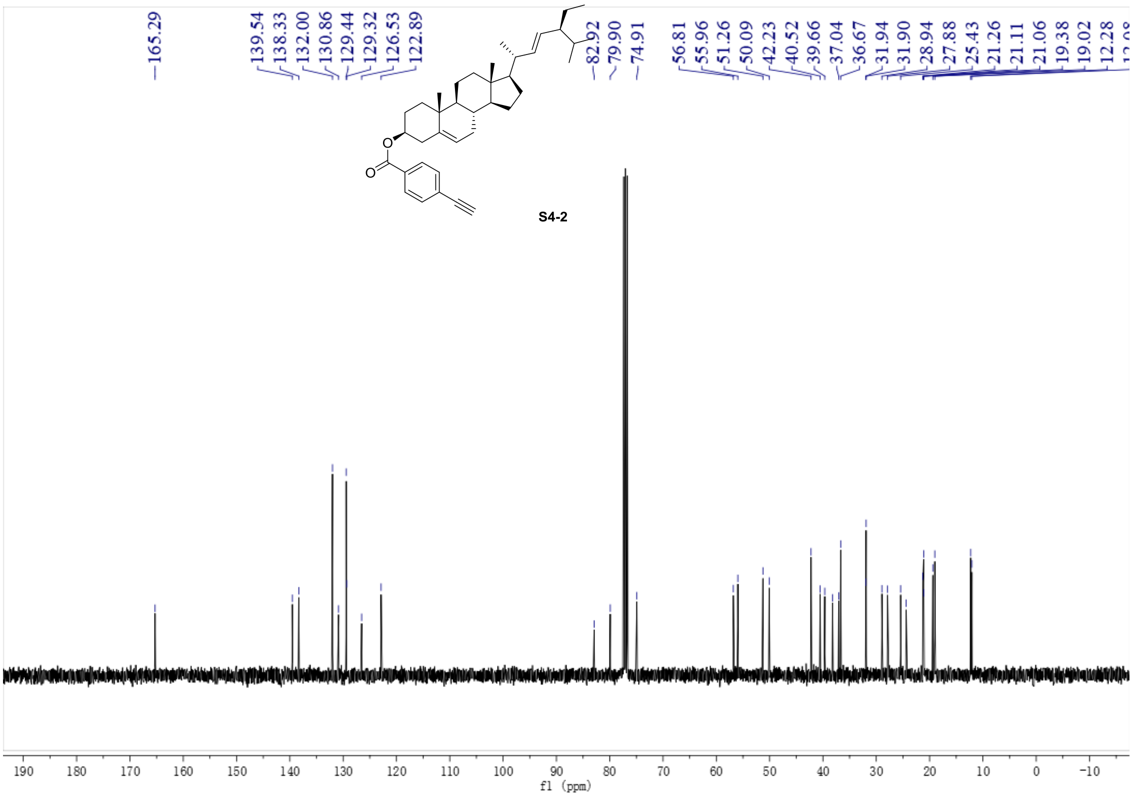

Supplementary Fig. 55 | ^{13}C NMR (101 MHz) of compound S4-2 (using $CDCl_3$ as solvent)

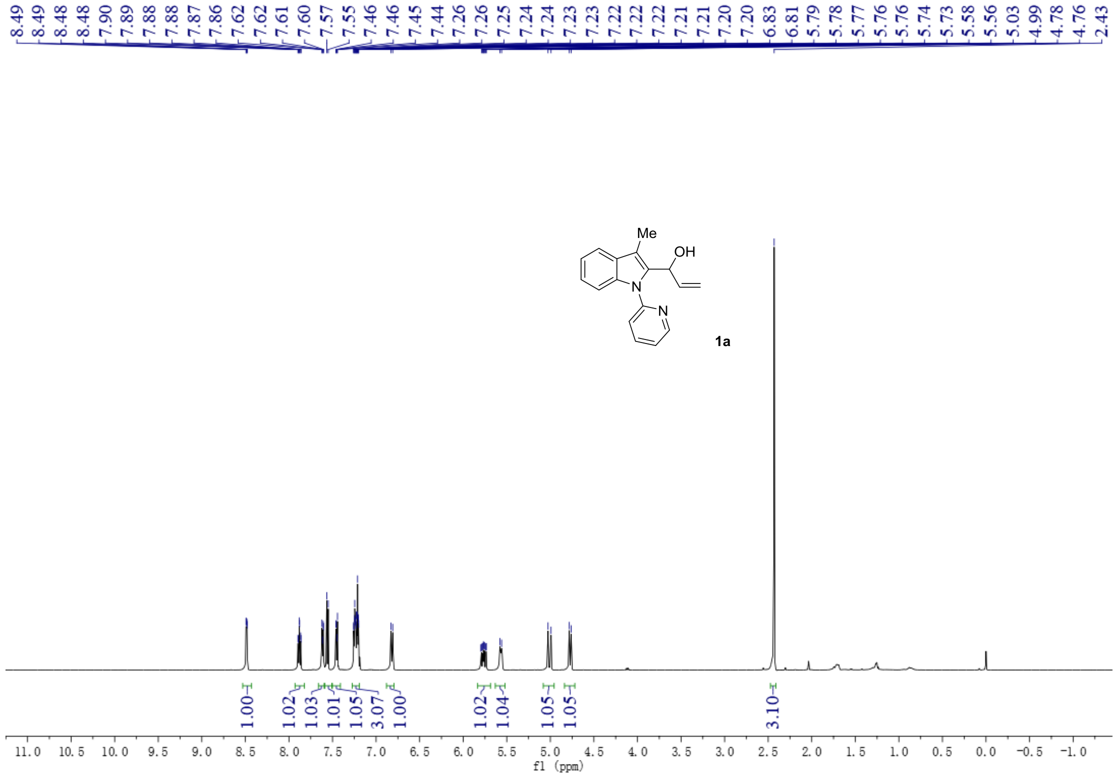

Supplementary Fig. 56 | ^{1}H NMR (500 MHz) of compound 1a (using $CDCl_3$ as solvent)

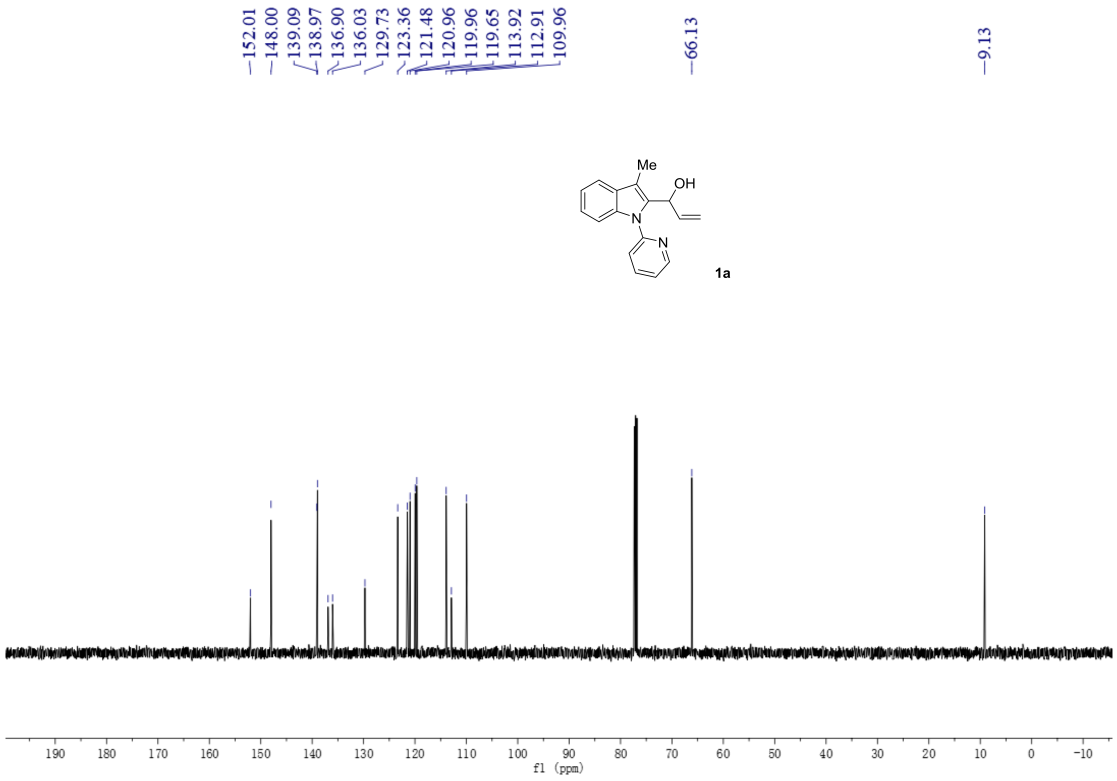

Supplementary Fig. 57 | ^{1}H NMR (126 MHz) of compound 1a (using $CDCl_3$ as solvent)

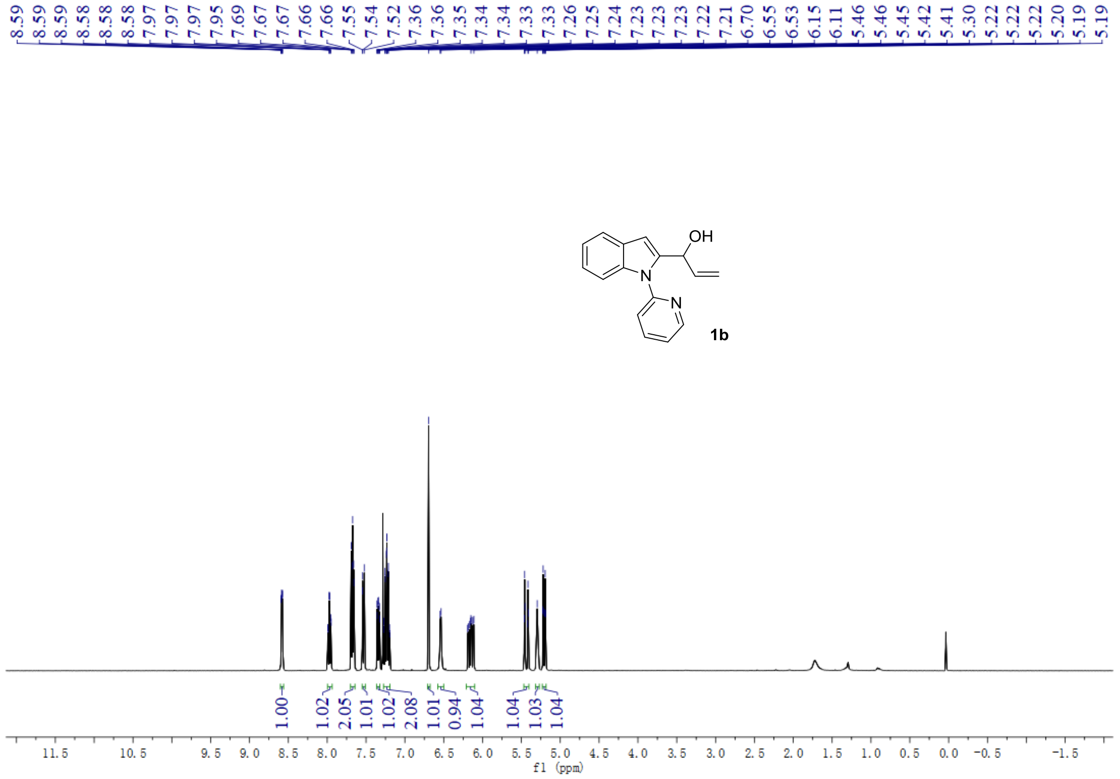

Supplementary Fig. 58 | ^{1}H NMR (400 MHz) of compound 1b (using $CDCl_3$ as solvent)

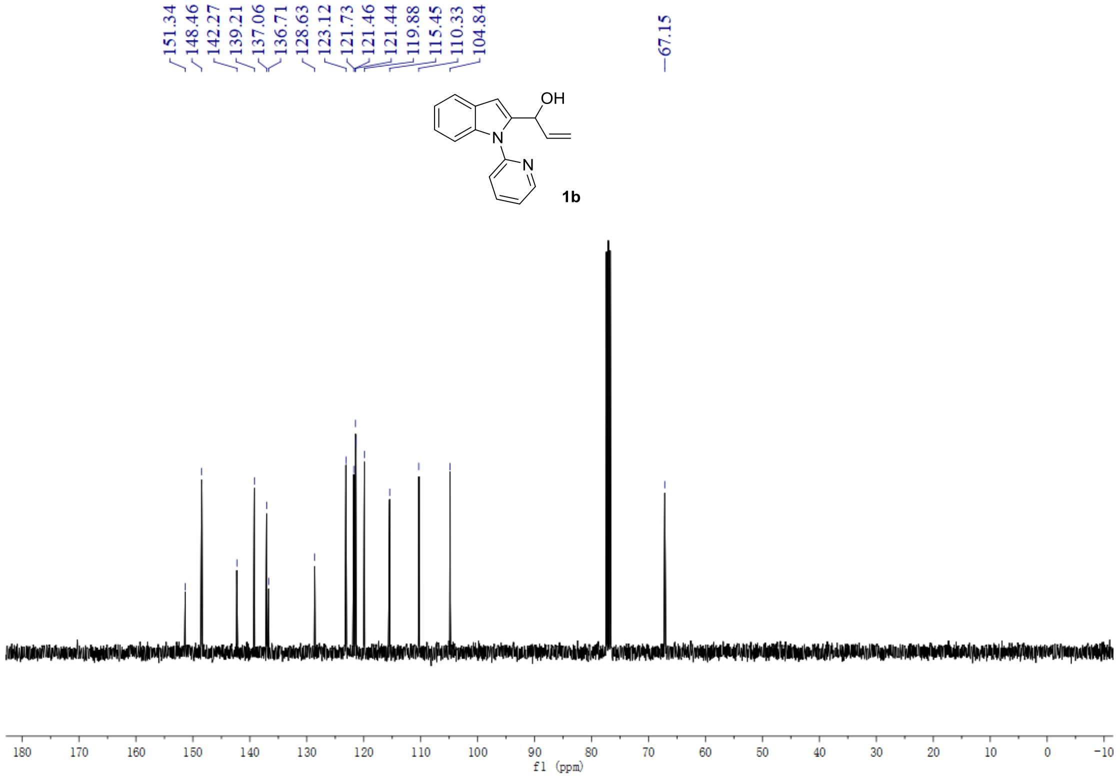

Supplementary Fig. 59 | ^{13}C NMR (101 MHz) of compound 1b (using $CDCl_3$ as solvent)

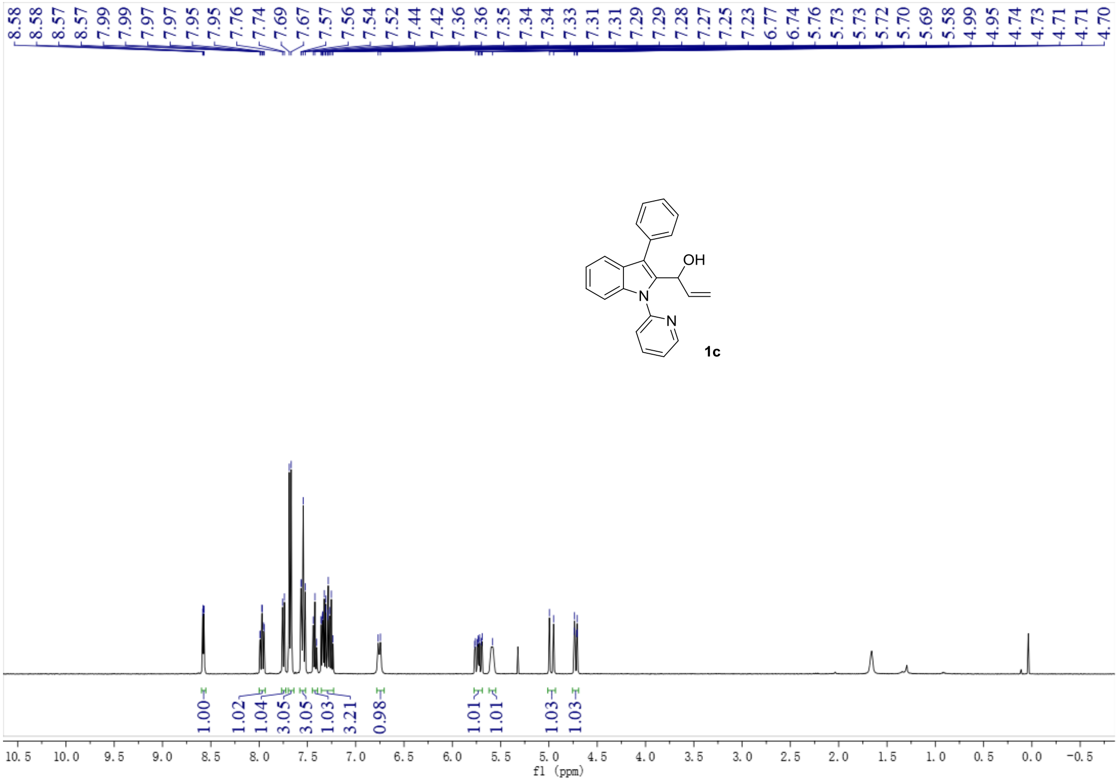

Supplementary Fig. 60 | ^{1}H NMR (400 MHz) of compound 1c (using $CDCl_3$ as solvent)

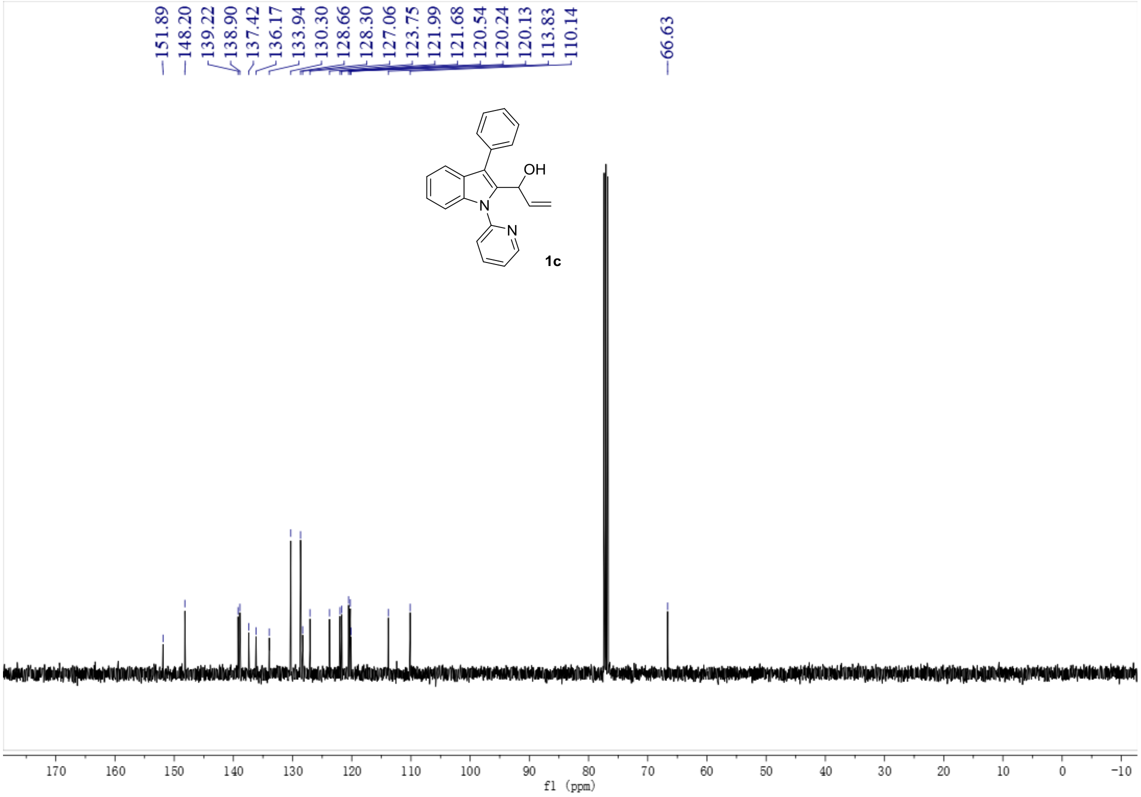

Supplementary Fig. 61 | ^{13}C NMR (101 MHz) of compound 1c (using $CDCl_3$ as solvent)

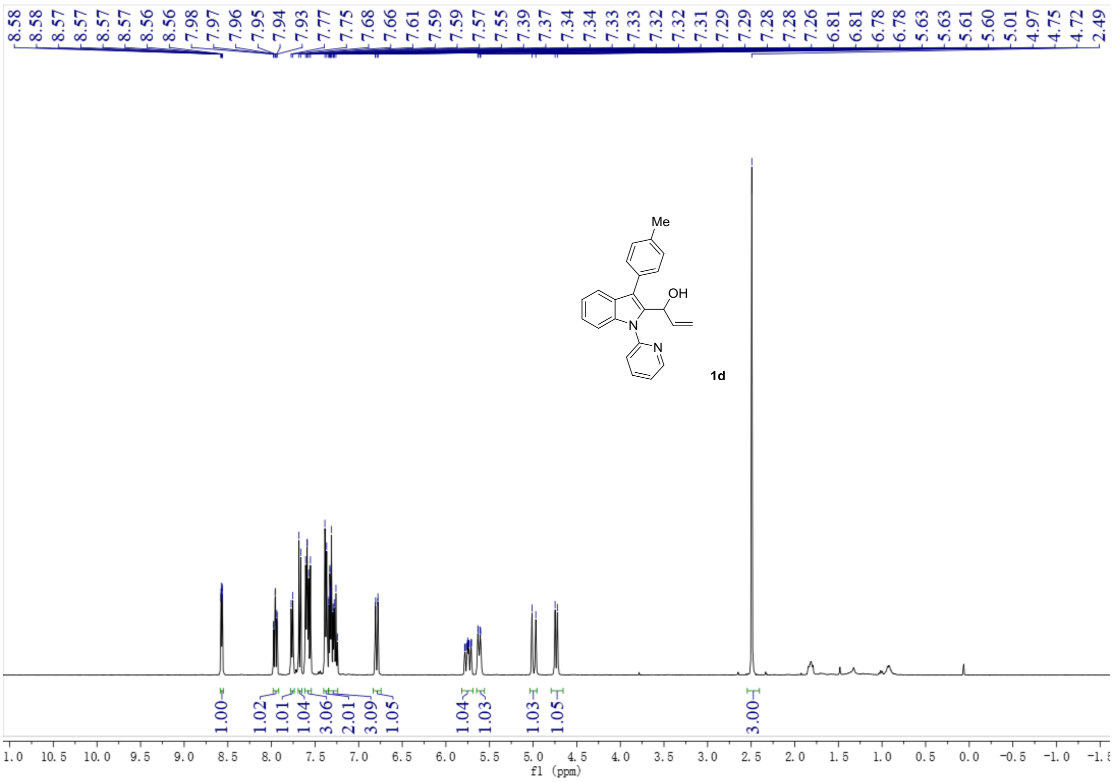

Supplementary Fig. 62 | ^{1}H NMR (500 MHz) of compound 1d (using $CDCl_3$ as solvent)

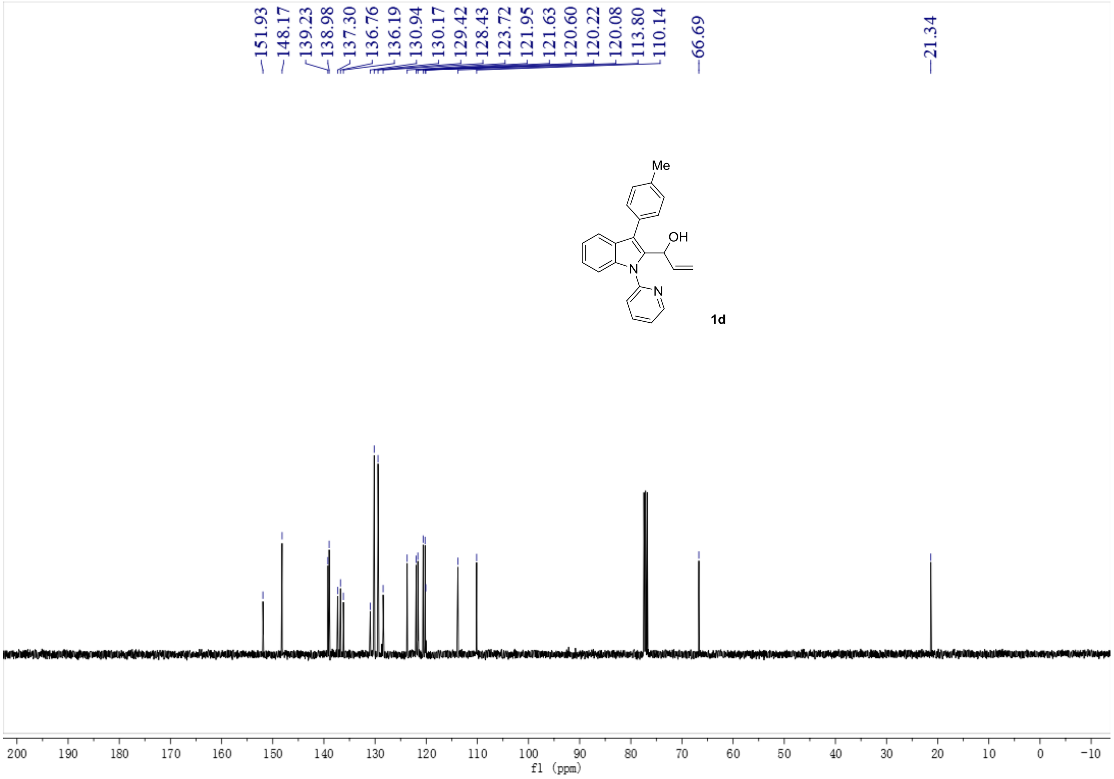

Supplementary Fig. 63 | ^{13}C NMR (126 MHz) of compound 1d (using $CDCl_3$ as solvent)

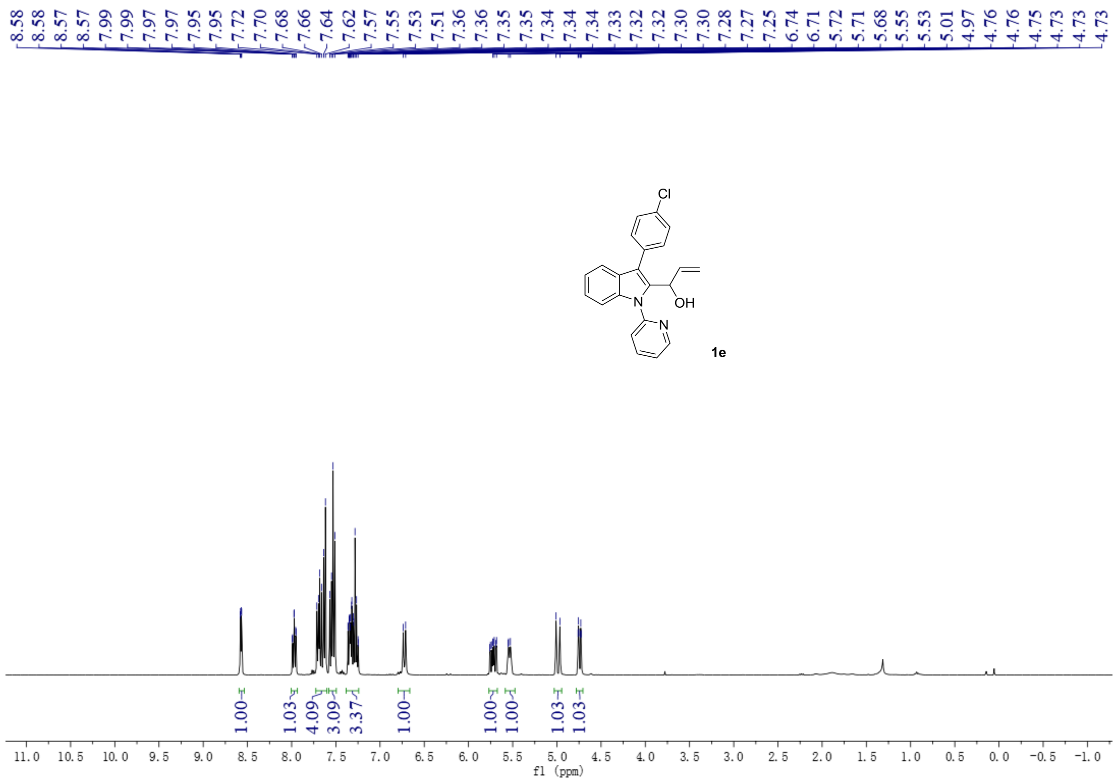

Supplementary Fig. 64 | ^{1}H NMR (400 MHz) of compound 1e (using $CDCl_3$ as solvent)

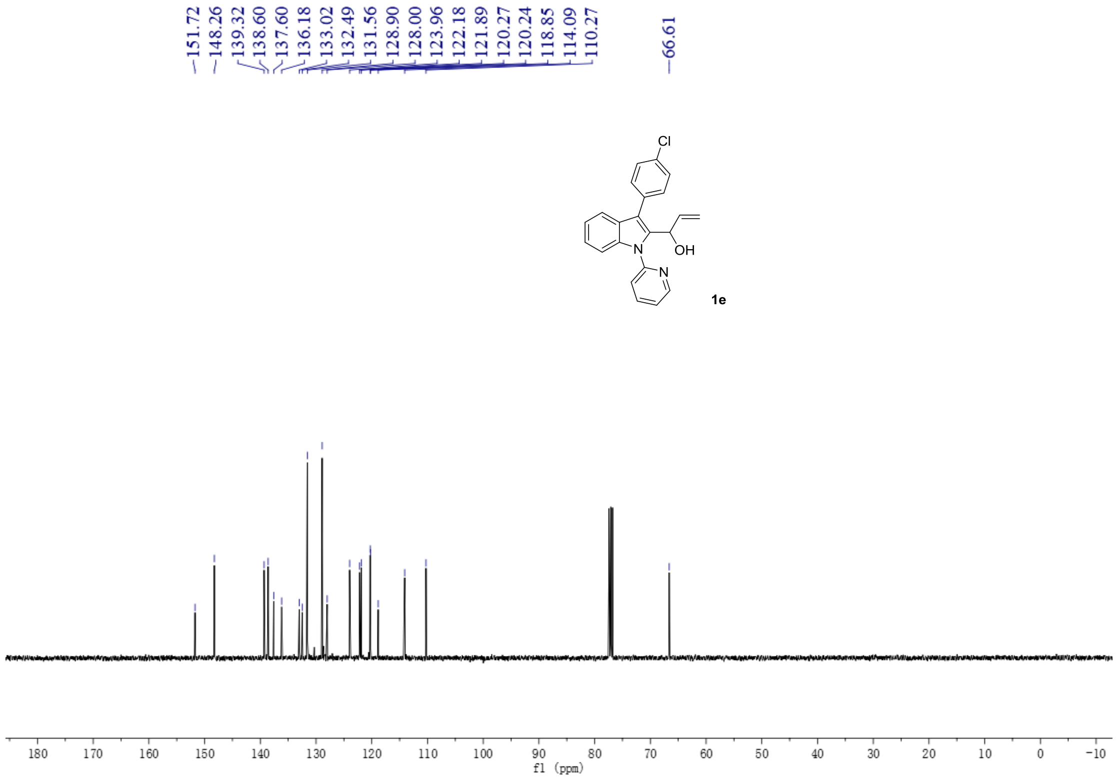

Supplementary Fig. 65 | ^{13}C NMR (101 MHz) of compound 1e (using $CDCl_3$ as solvent)

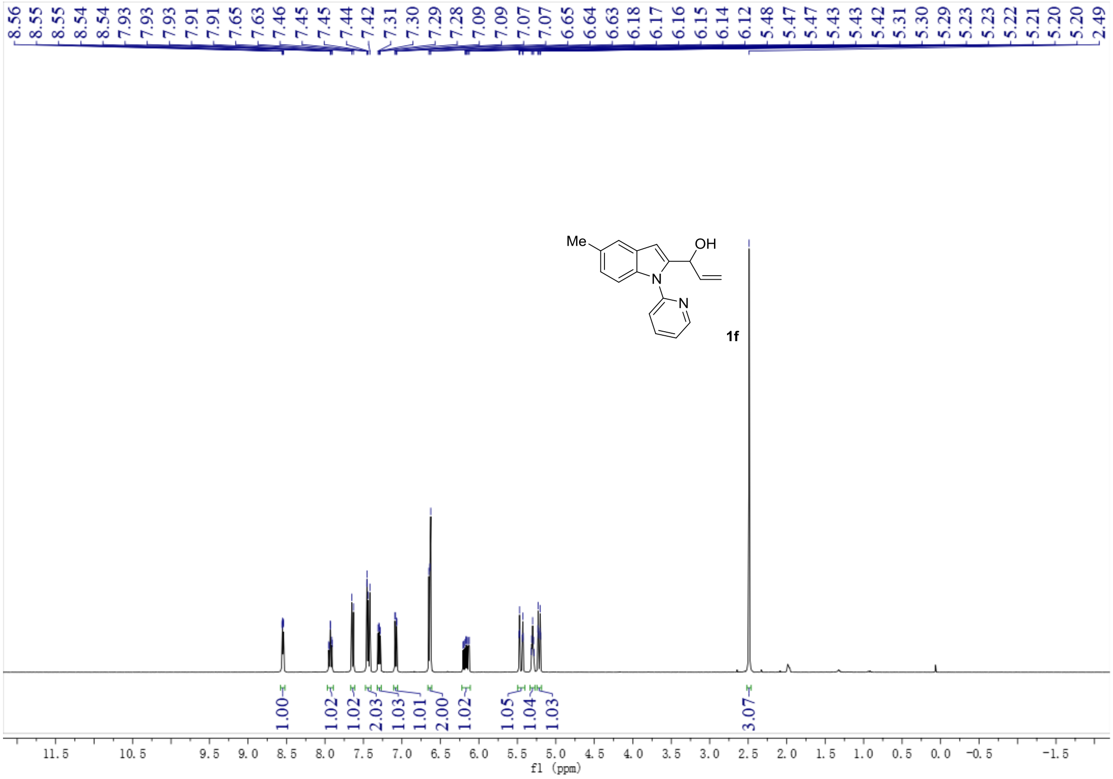

Supplementary Fig. 66 | ^{1}H NMR (400 MHz) of compound 1f (using $CDCl_3$ as solvent)

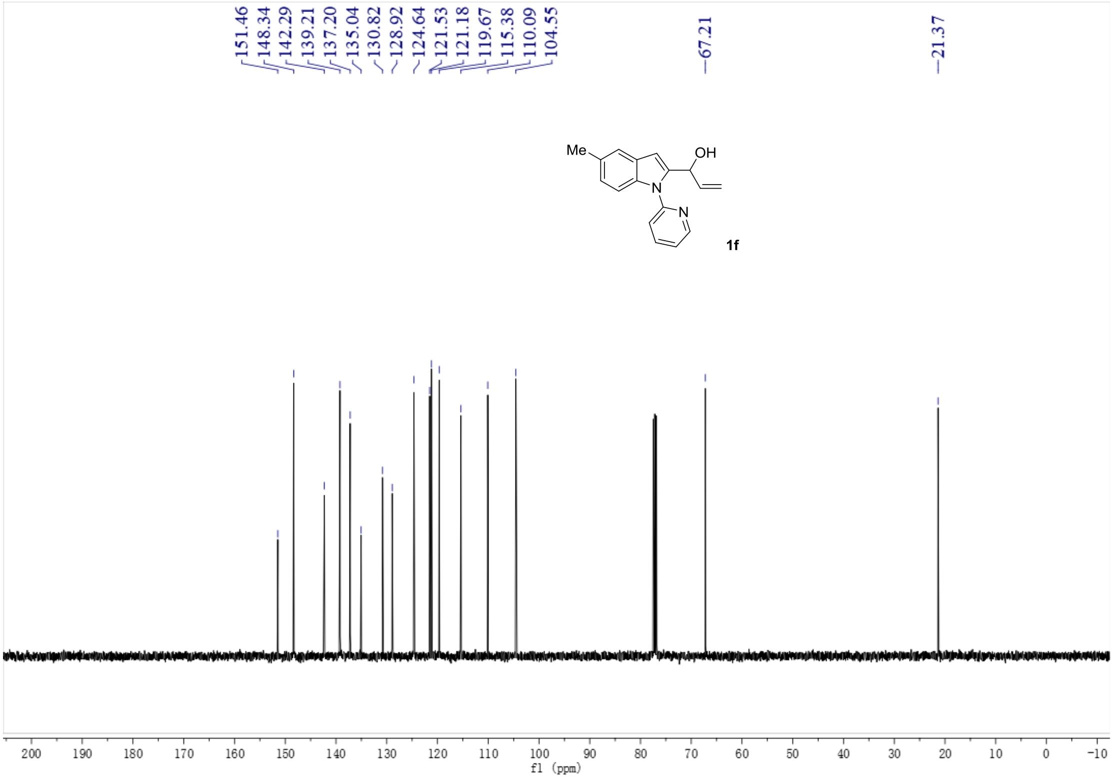

Supplementary Fig. 67 | ^{13}C NMR (101 MHz) of compound 1f (using $CDCl_3$ as solvent)

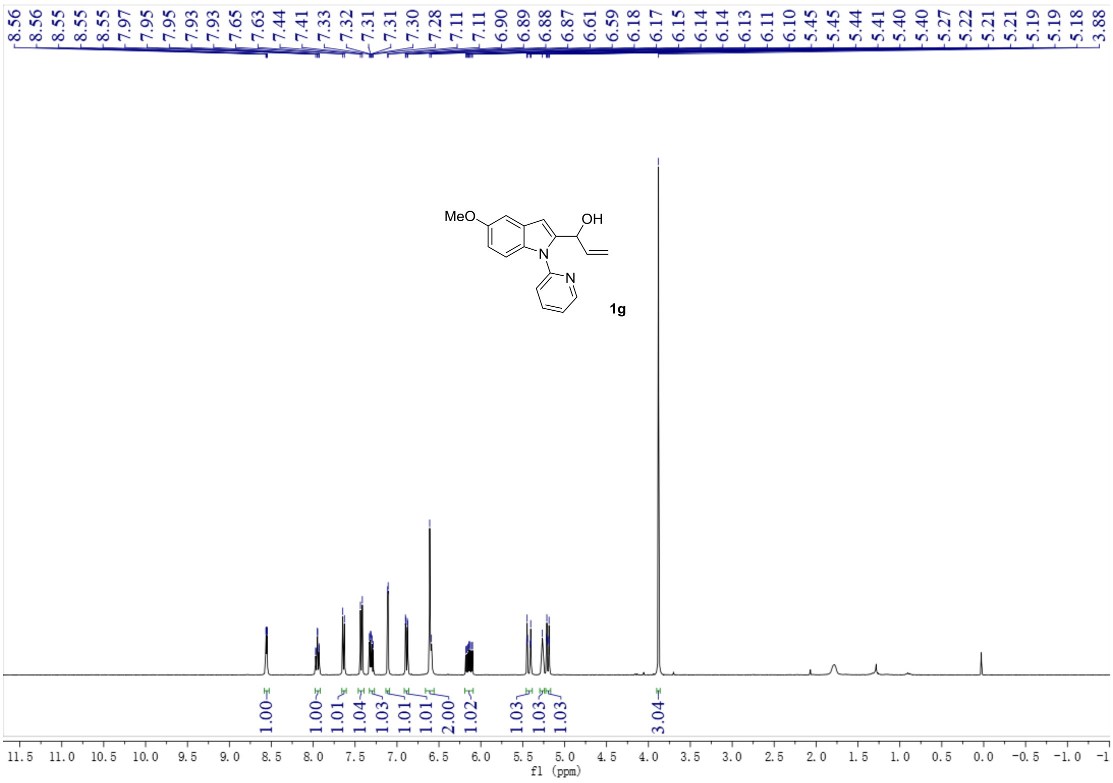

Supplementary Fig. 68 | ^{1}H NMR (400 MHz) of compound 1g (using $CDCl_3$ as solvent)

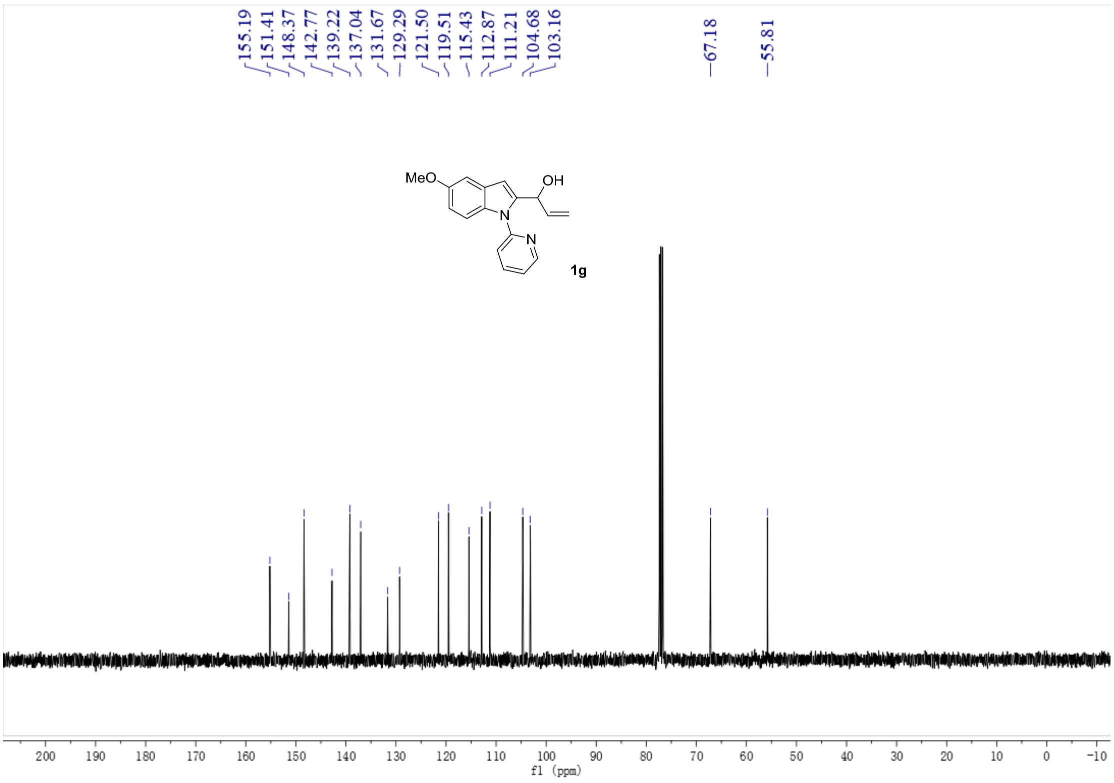

Supplementary Fig. 69 | ^{13}C NMR (101 MHz) of compound 1g (using $CDCl_3$ as solvent)

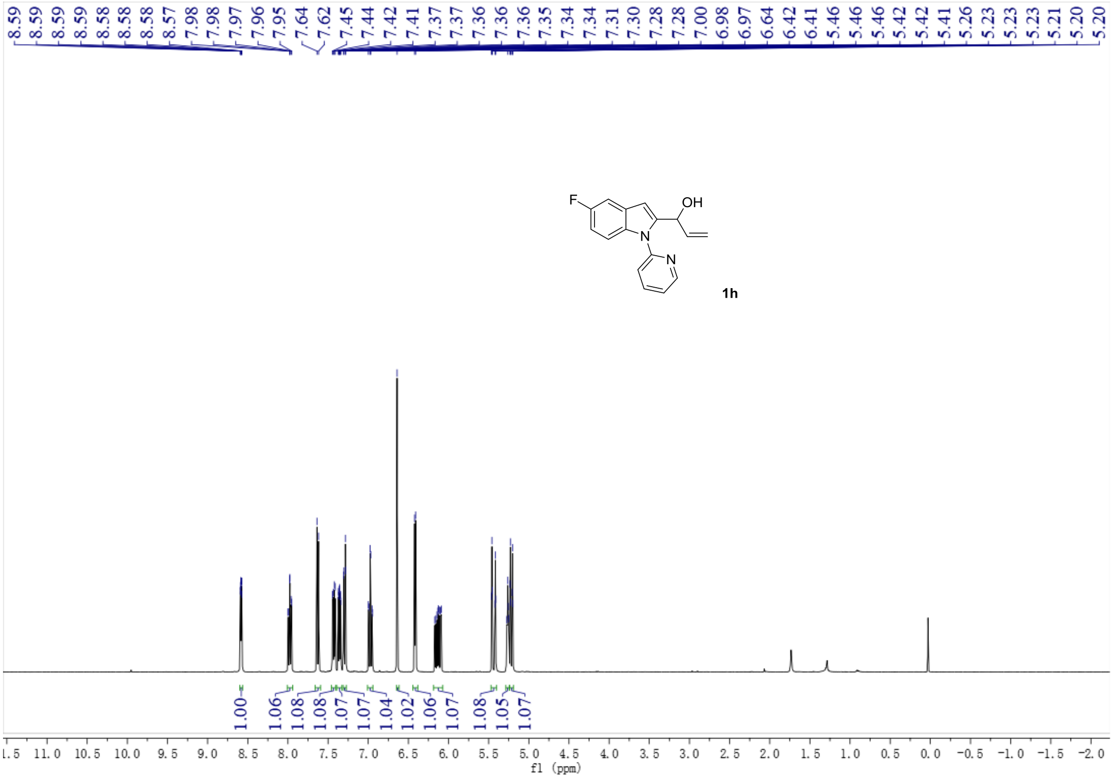

Supplementary Fig. 70 | ^{1}H NMR (400 MHz) of compound 1h (using $CDCl_3$ as solvent)

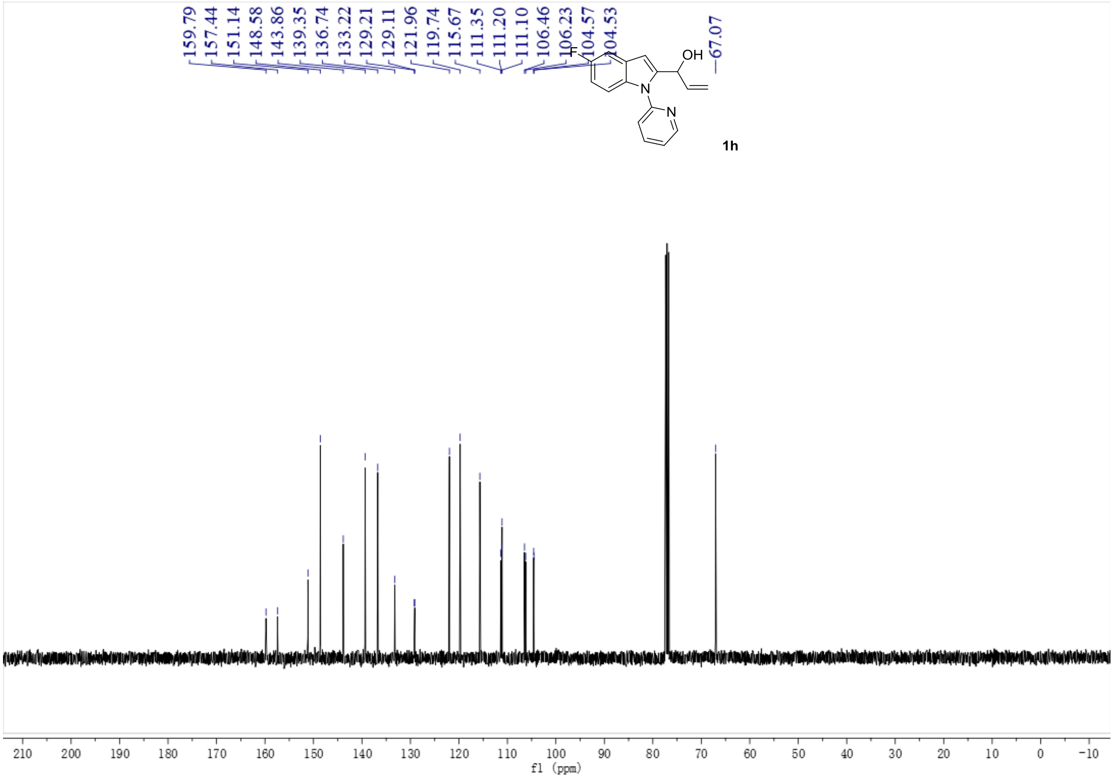

Supplementary Fig. 71 | ^{13}C NMR (101 MHz) of compound 1h (using $CDCl_3$ as solvent)

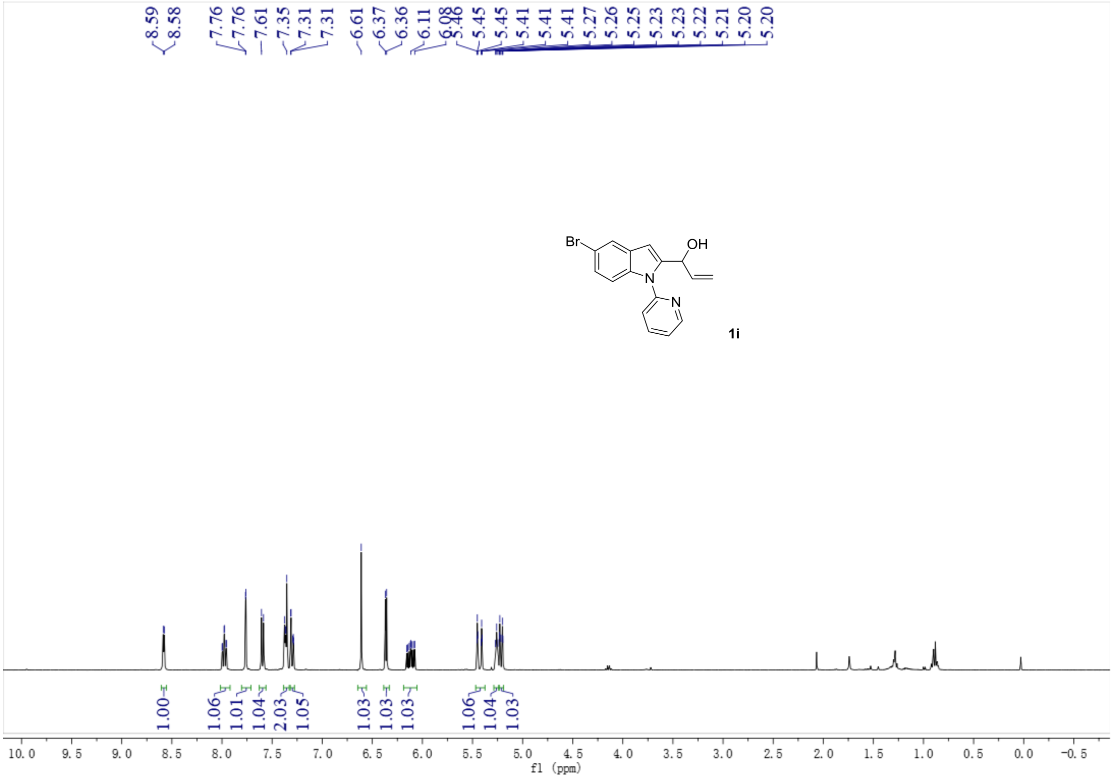

Supplementary Fig. 72 | ^{1}H NMR (400 MHz) of compound 1i (using $CDCl_3$ as solvent)

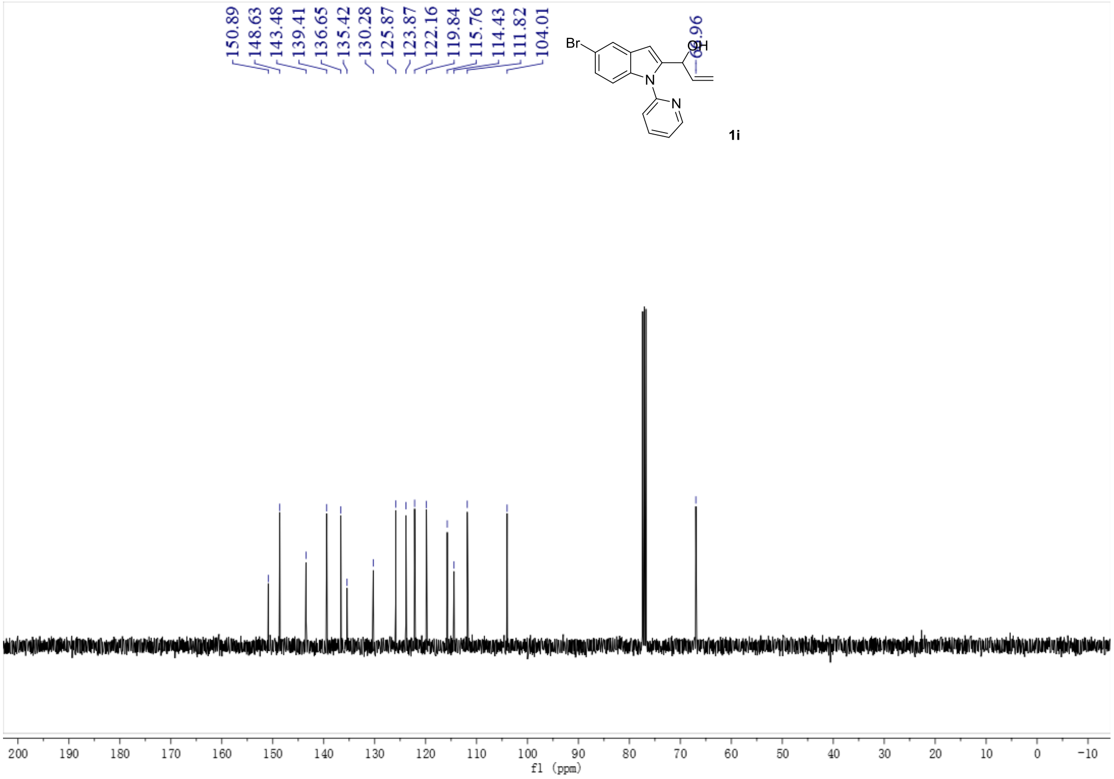

Supplementary Fig. 73 | ^{13}C NMR (101 MHz) of compound 1i (using $CDCl_3$ as solvent)

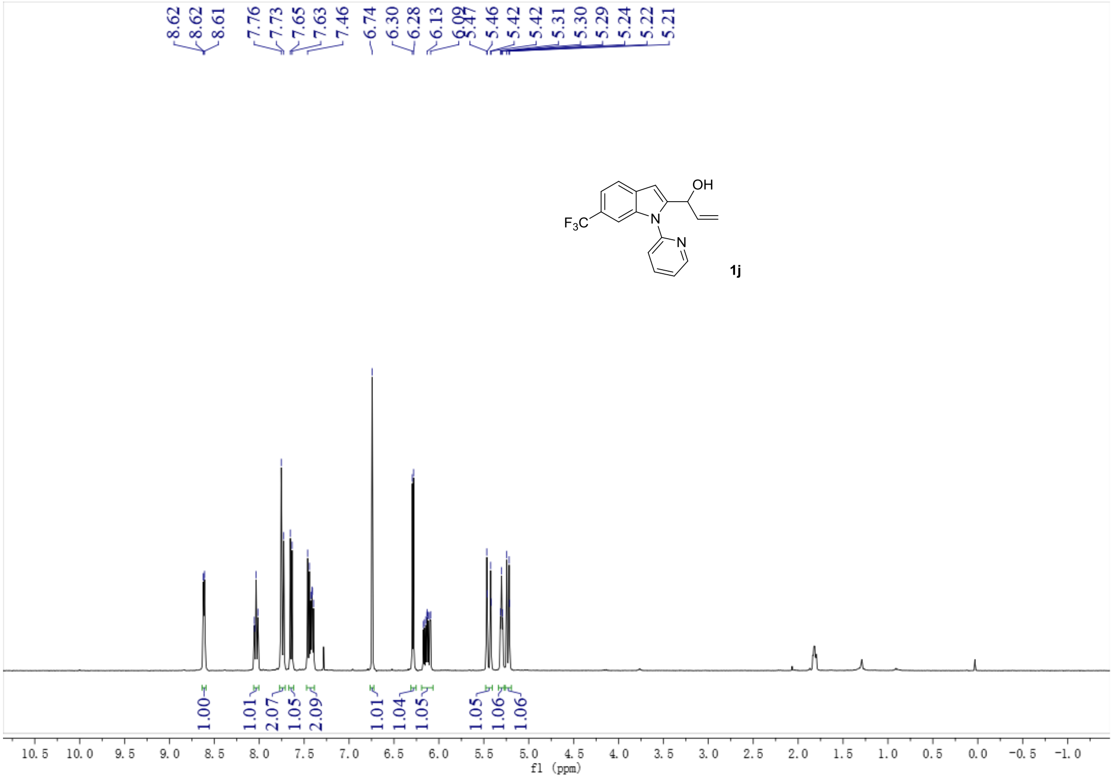

Supplementary Fig. 74 | ^{1}H NMR (400 MHz) of compound 1j (using $CDCl_3$ as solvent)

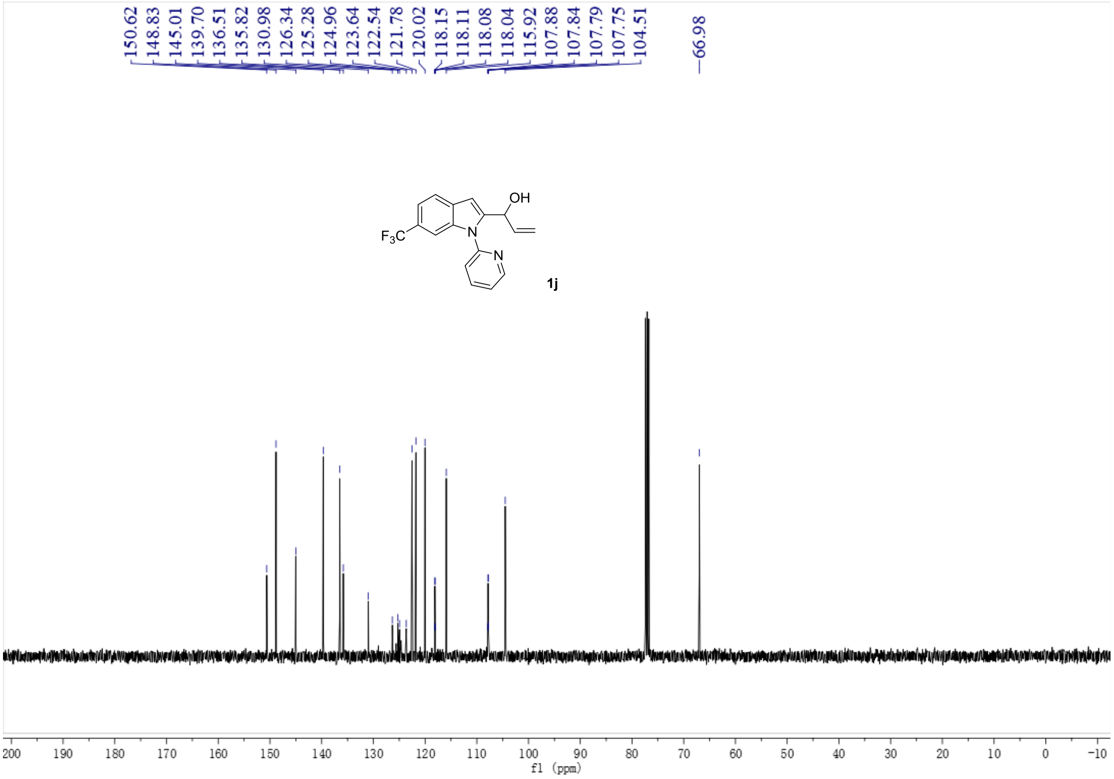

Supplementary Fig. 75 | ^{13}C NMR (101 MHz) of compound 1j (using $CDCl_3$ as solvent)

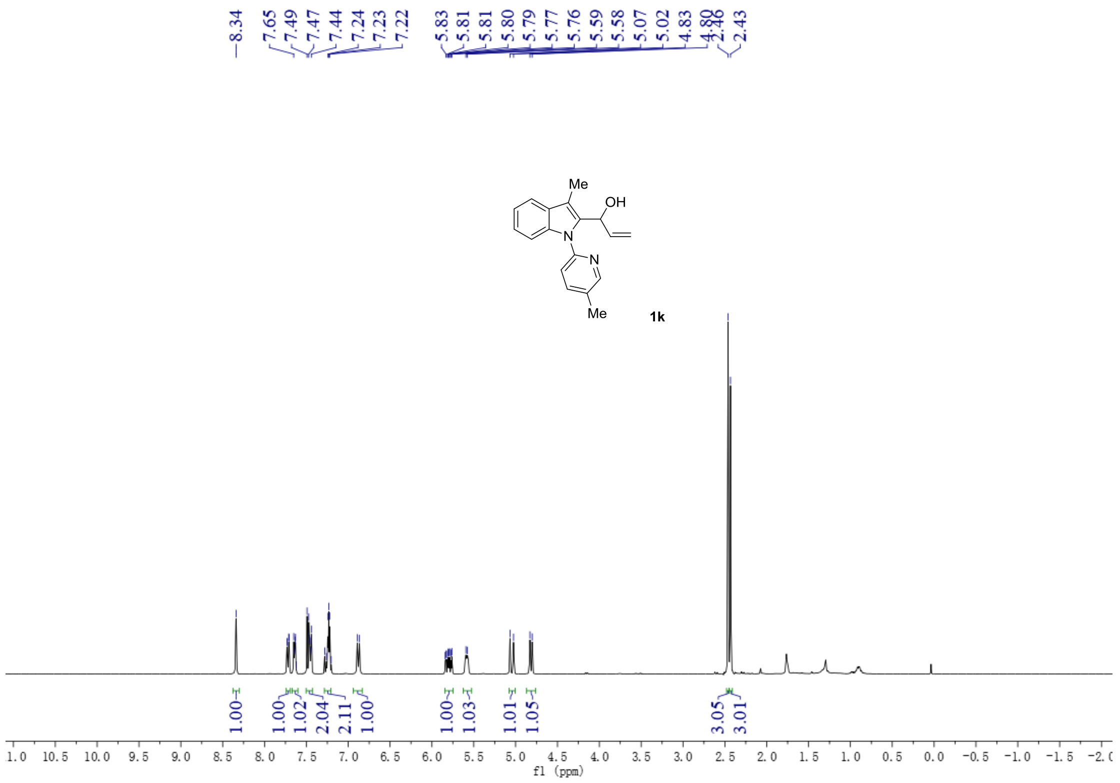

Supplementary Fig. 76 | ^{1}H NMR (400 MHz) of compound 1k (using $CDCl_3$ as solvent)

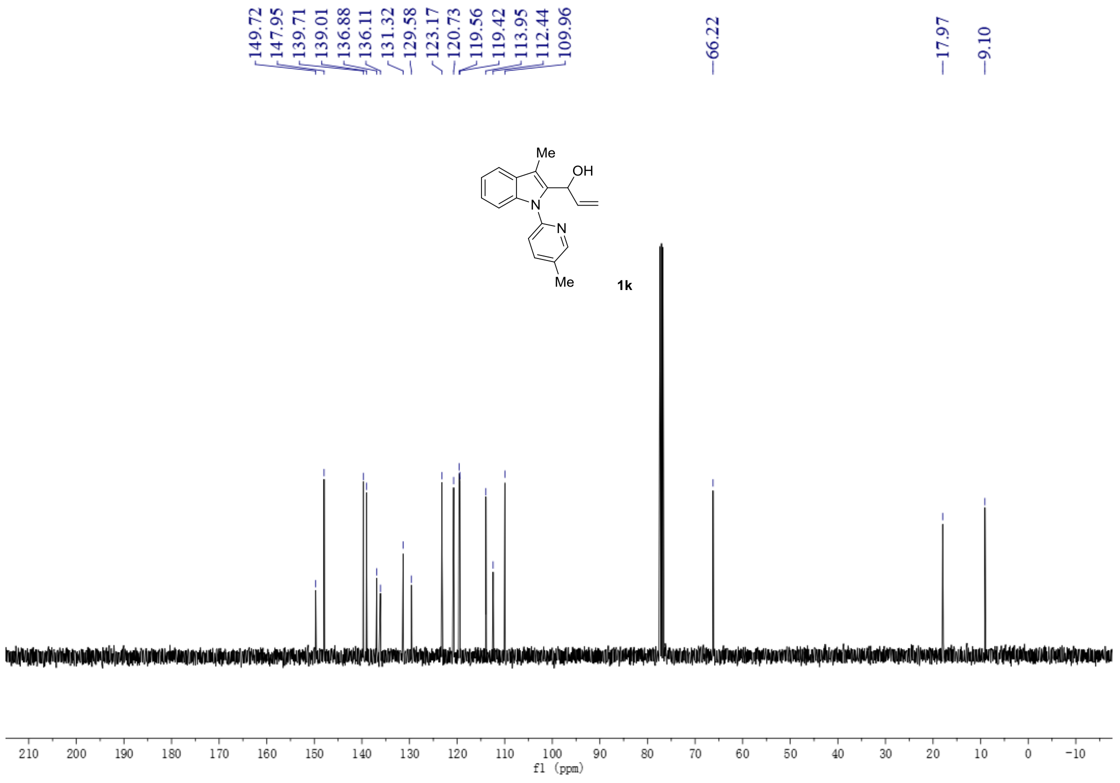

Supplementary Fig. 77 | ^{13}C NMR (101 MHz) of compound 1k (using $CDCl_3$ as solvent)

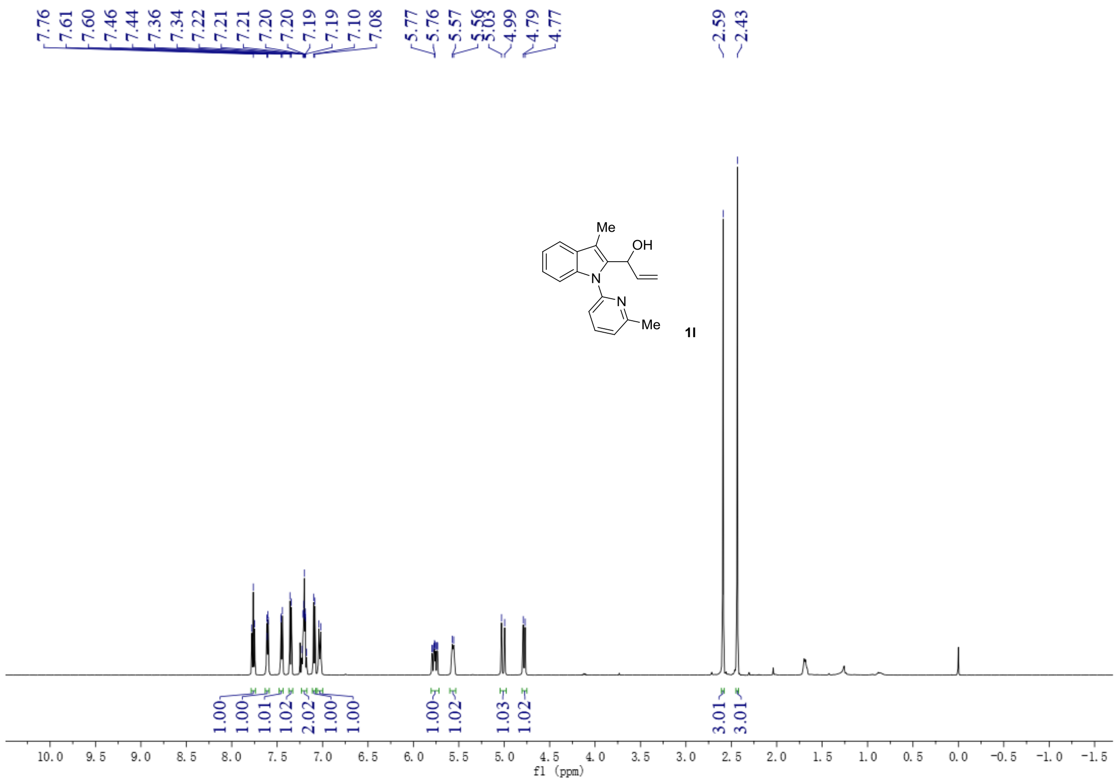

Supplementary Fig. 78 | ^{1}H NMR (500 MHz) of compound 1l (using $CDCl_3$ as solvent)

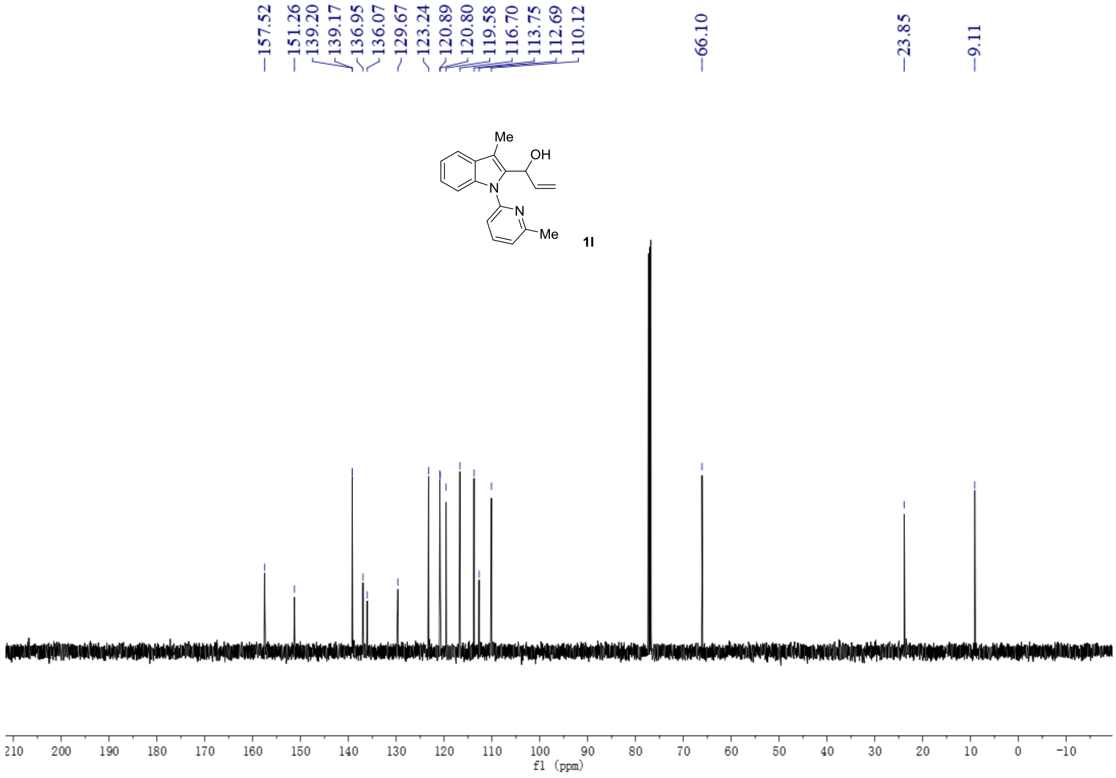

Supplementary Fig. 79 | ^{13}C NMR (126 MHz) of compound 1l (using $CDCl_3$ as solvent)

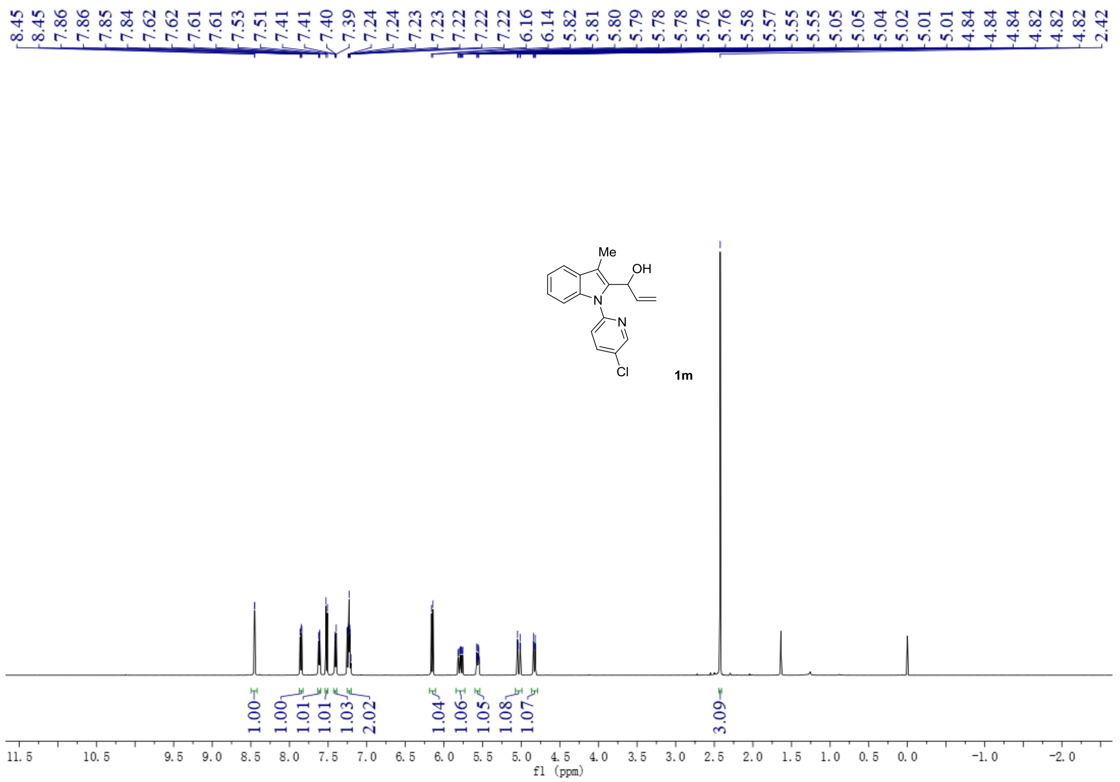

Supplementary Fig. 80 | ^{1}H NMR (500 MHz) of compound 1m (using $CDCl_3$ as solvent)

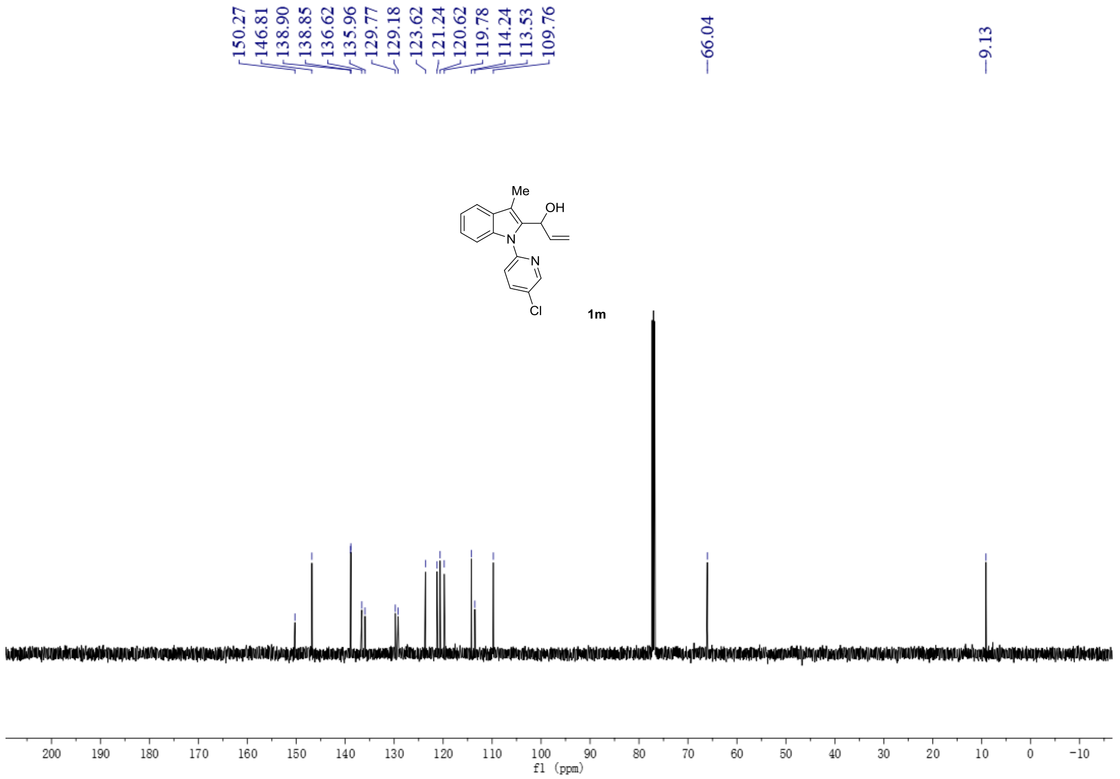

Supplementary Fig. 81 | ^{13}C NMR (126 MHz) of compound 1m (using $CDCl_3$ as solvent)

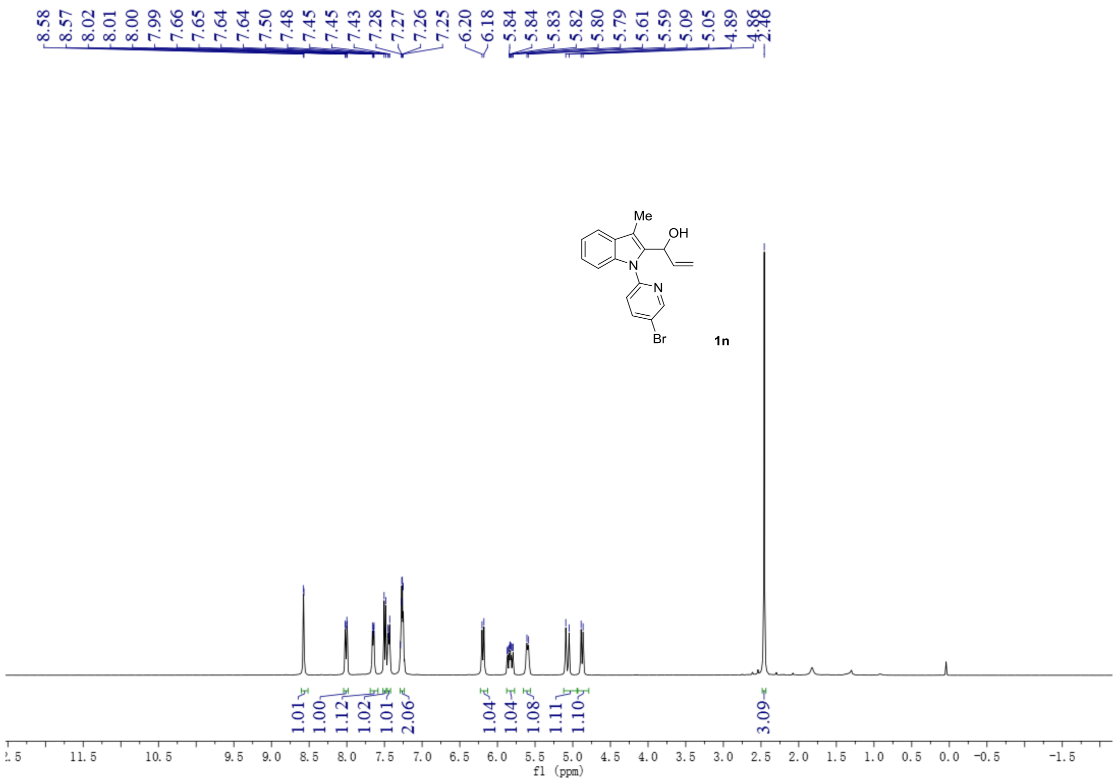

Supplementary Fig. 82 | ^{1}H NMR (400 MHz) of compound 1n (using $CDCl_3$ as solvent)

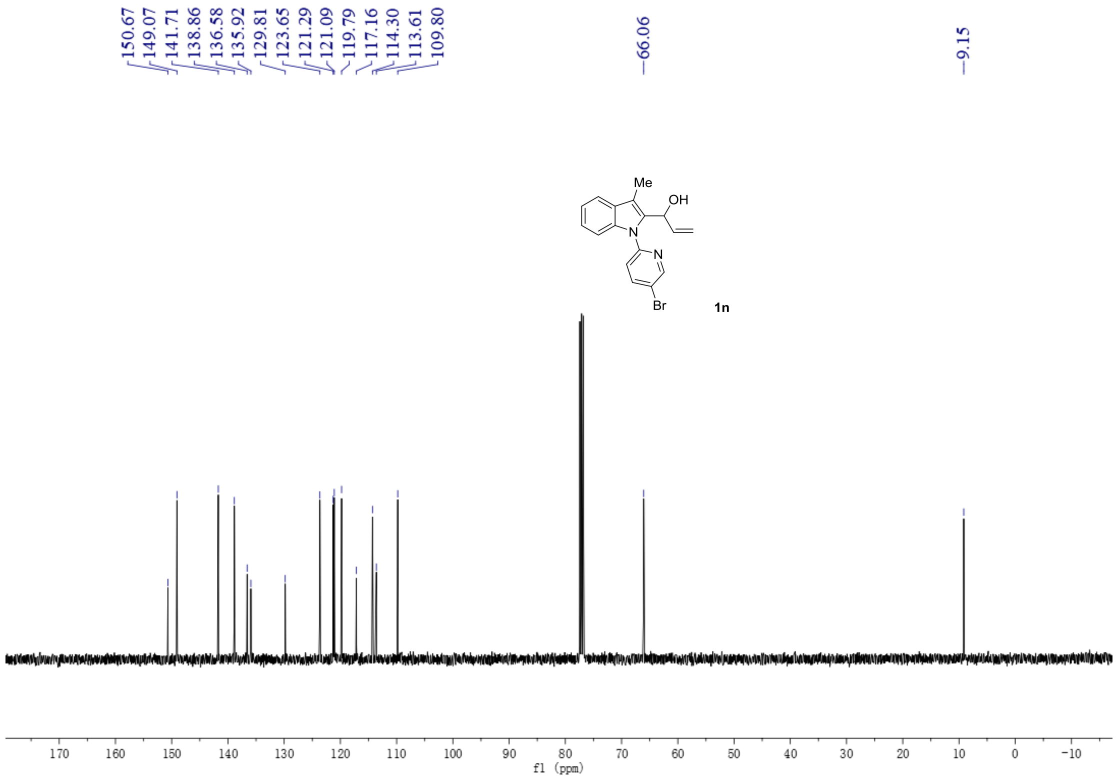

Supplementary Fig. 83 | ^{13}C NMR (101 MHz) of compound 1n (using $CDCl_3$ as solvent)

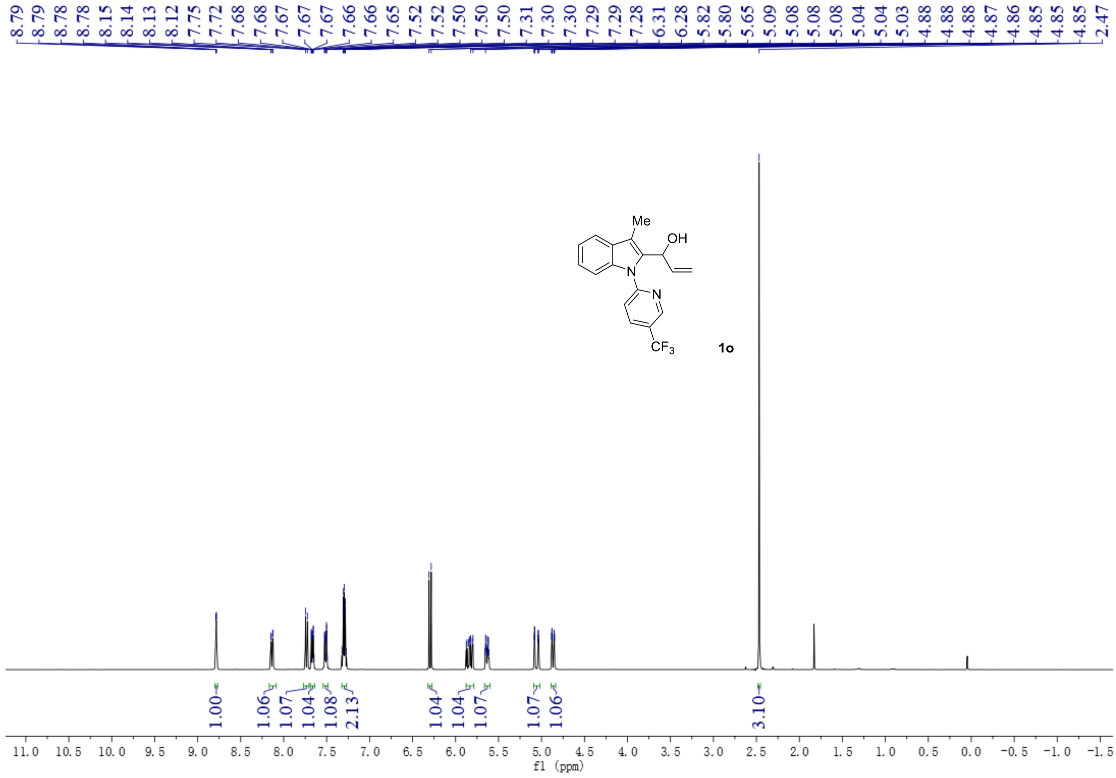

Supplementary Fig. 84 | ^{1}H NMR (400 MHz) of compound 1o (using $CDCl_3$ as solvent)

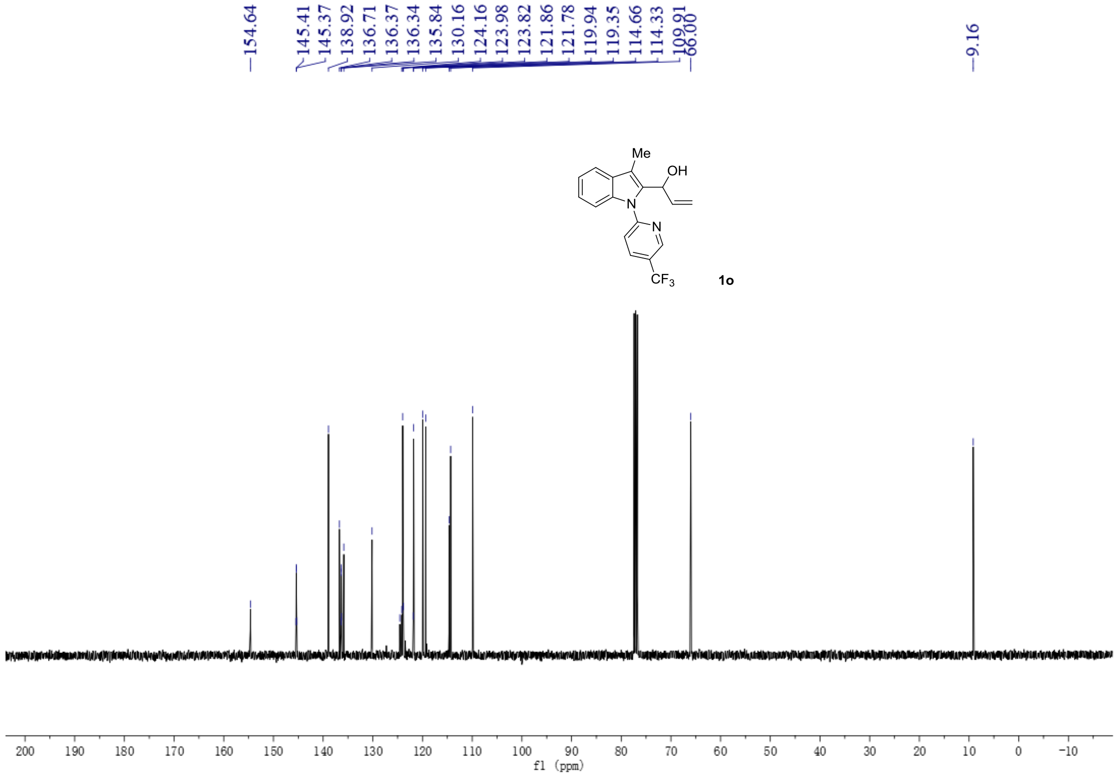

Supplementary Fig. 85 | ^{13}C NMR (101 MHz) of compound 1o (using $CDCl_3$ as solvent)

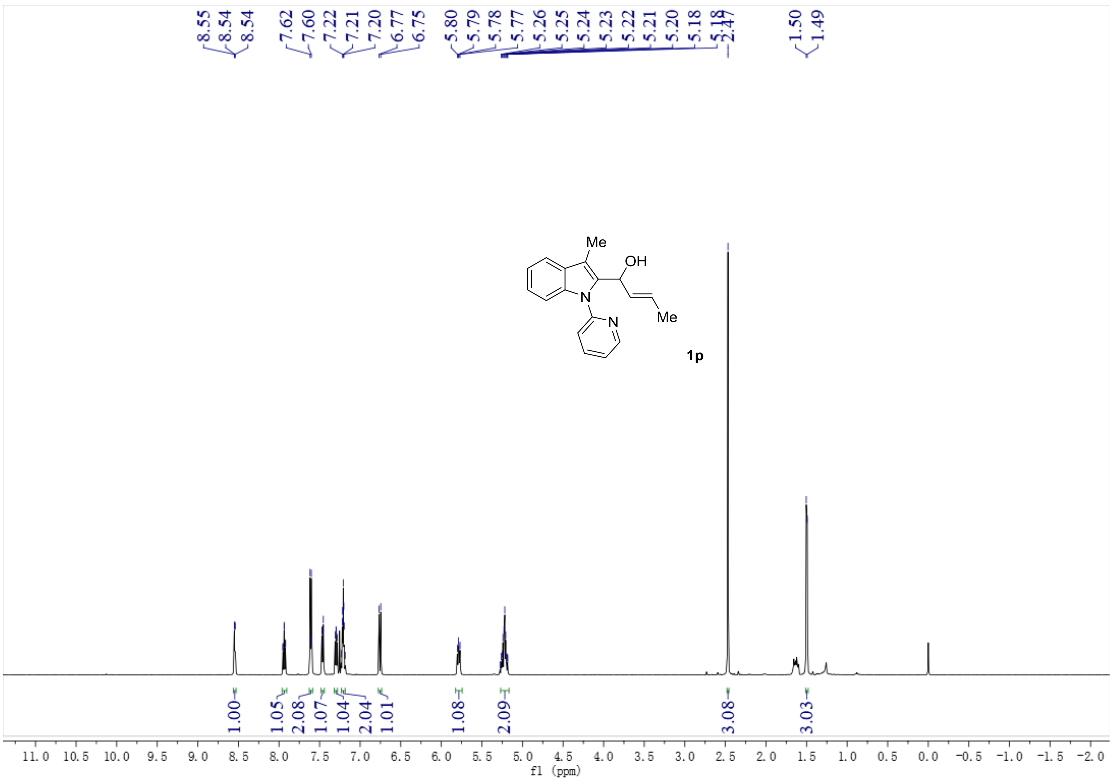

Supplementary Fig. 86 | ^{1}H NMR (500 MHz) of compound 1p (using $CDCl_3$ as solvent)

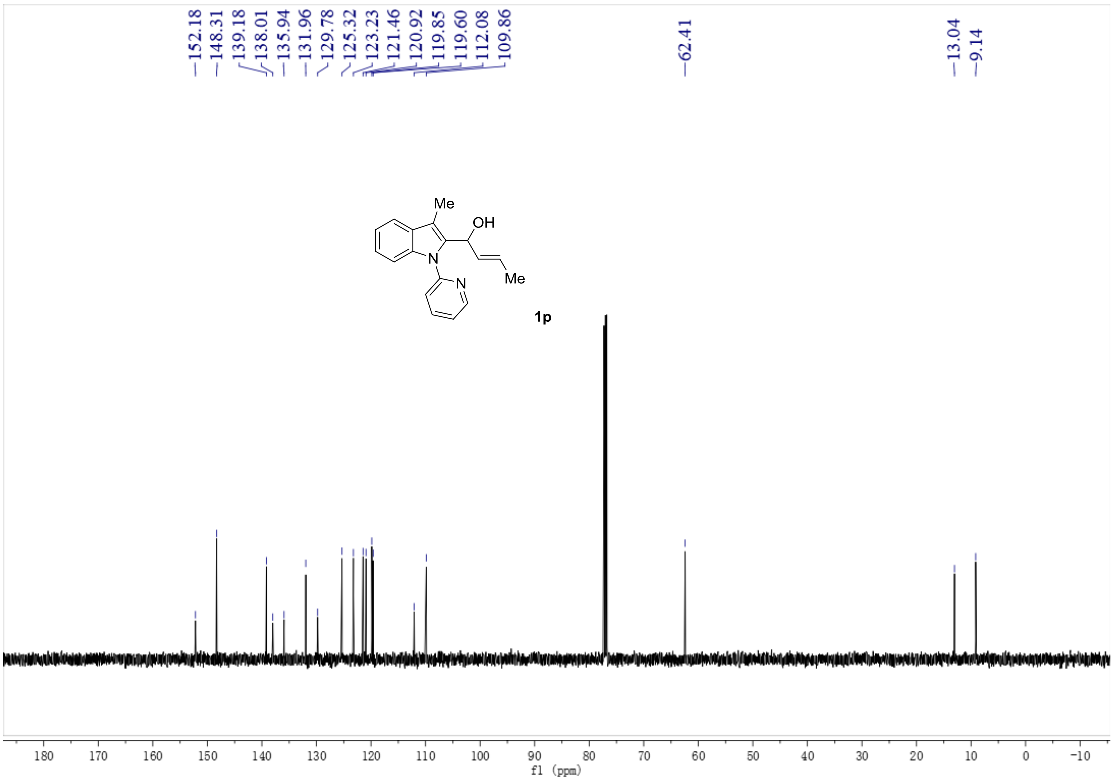

Supplementary Fig. 87 | ^{13}C NMR (126 MHz) of compound 1p (using $CDCl_3$ as solvent)

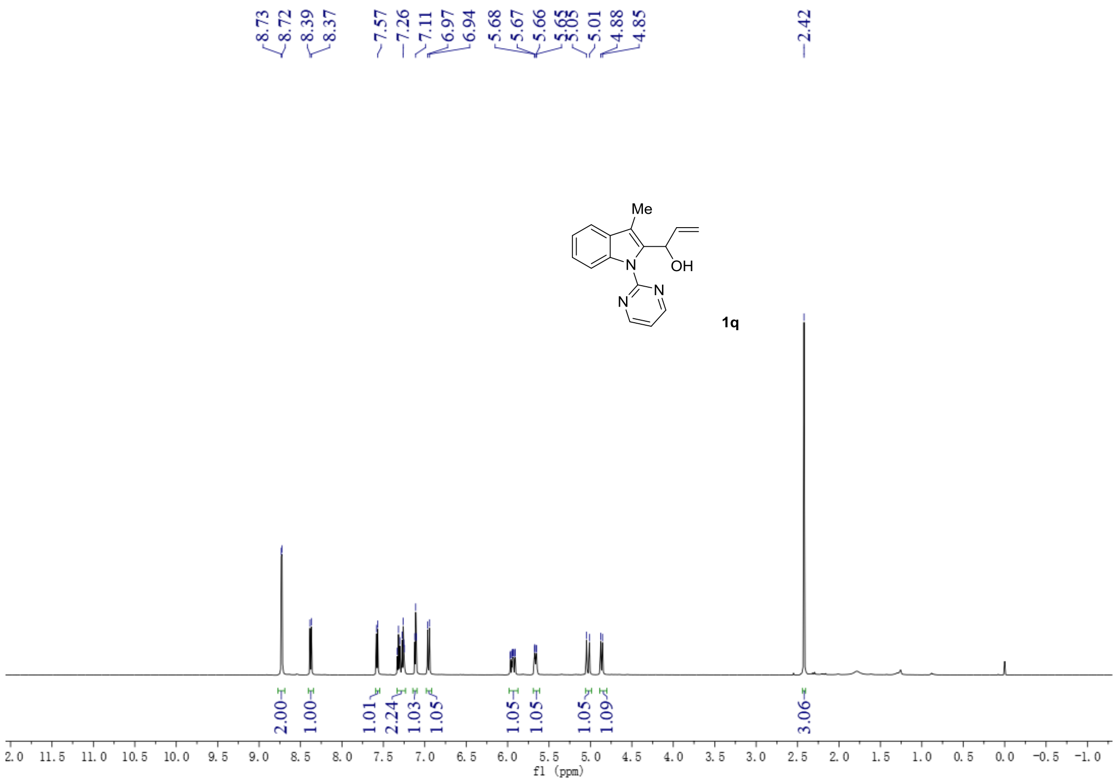

Supplementary Fig. 88 | ^{1}H NMR (500 MHz) of compound 1q (using $CDCl_3$ as solvent)

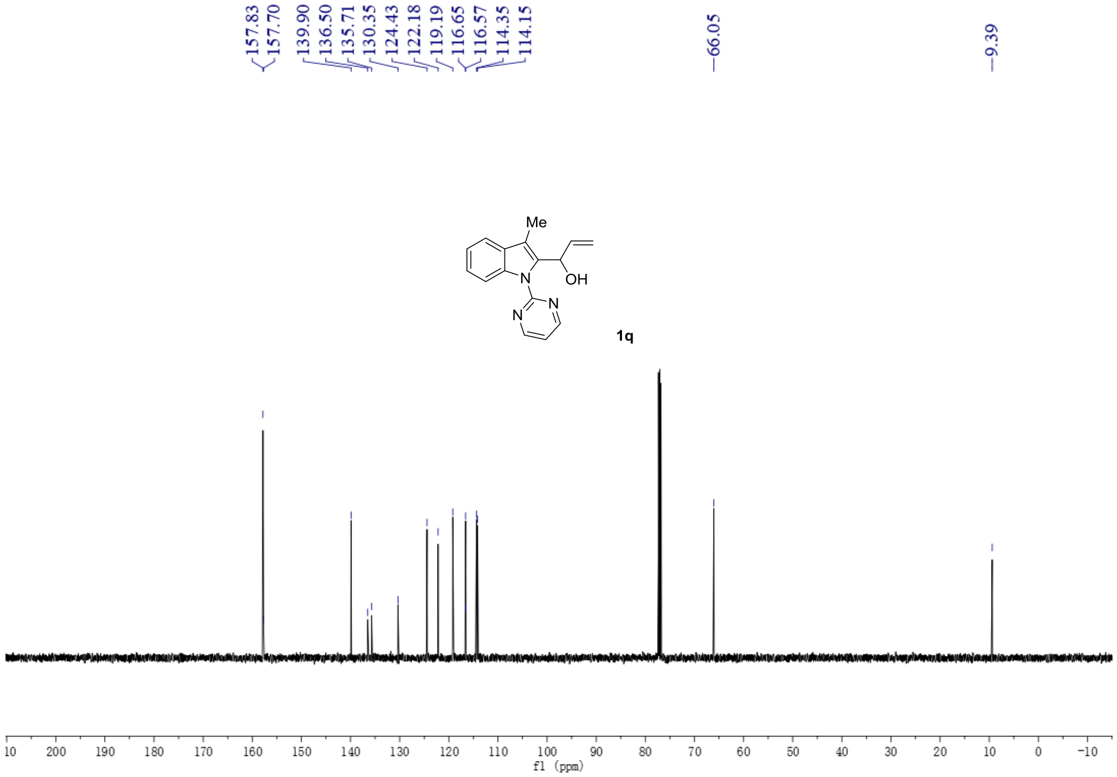

Supplementary Fig. 89 | ^{13}C NMR (126 MHz) of compound 1q (using $CDCl_3$ as solvent)

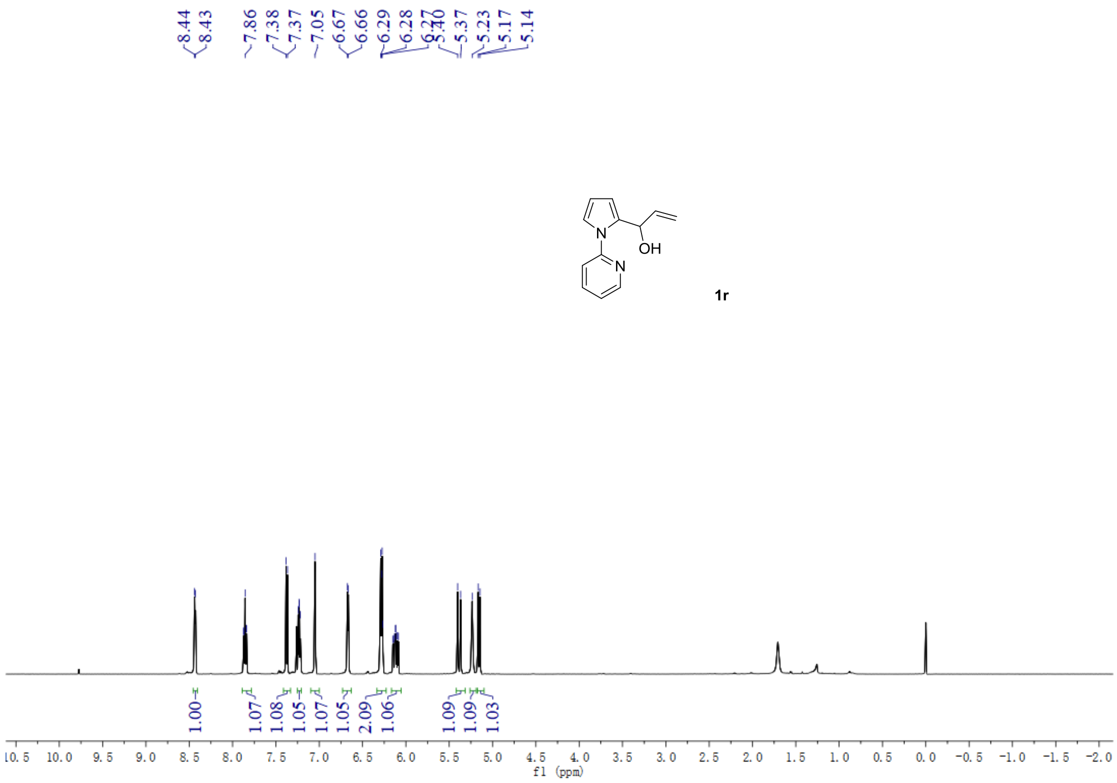

Supplementary Fig. 90 | ^{1}H NMR (500 MHz) of compound 1r (using $CDCl_3$ as solvent)

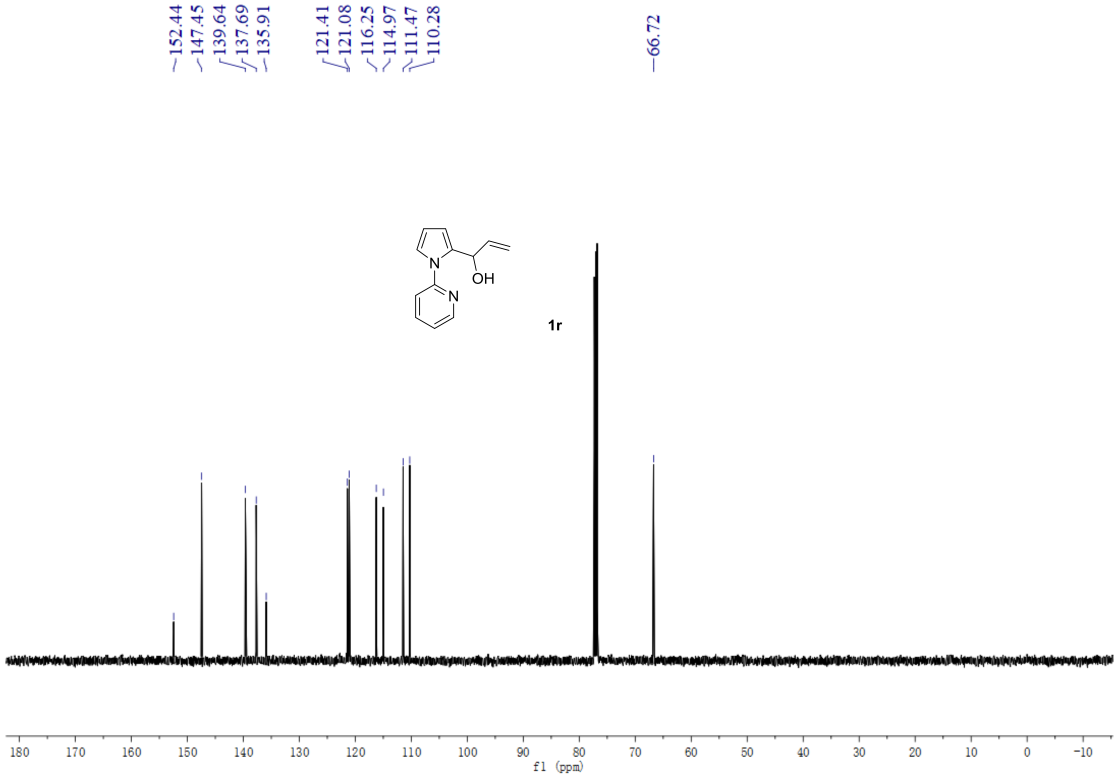

Supplementary Fig. 91 | ^{13}C NMR (126 MHz) of compound 1r (using $CDCl_3$ as solvent)

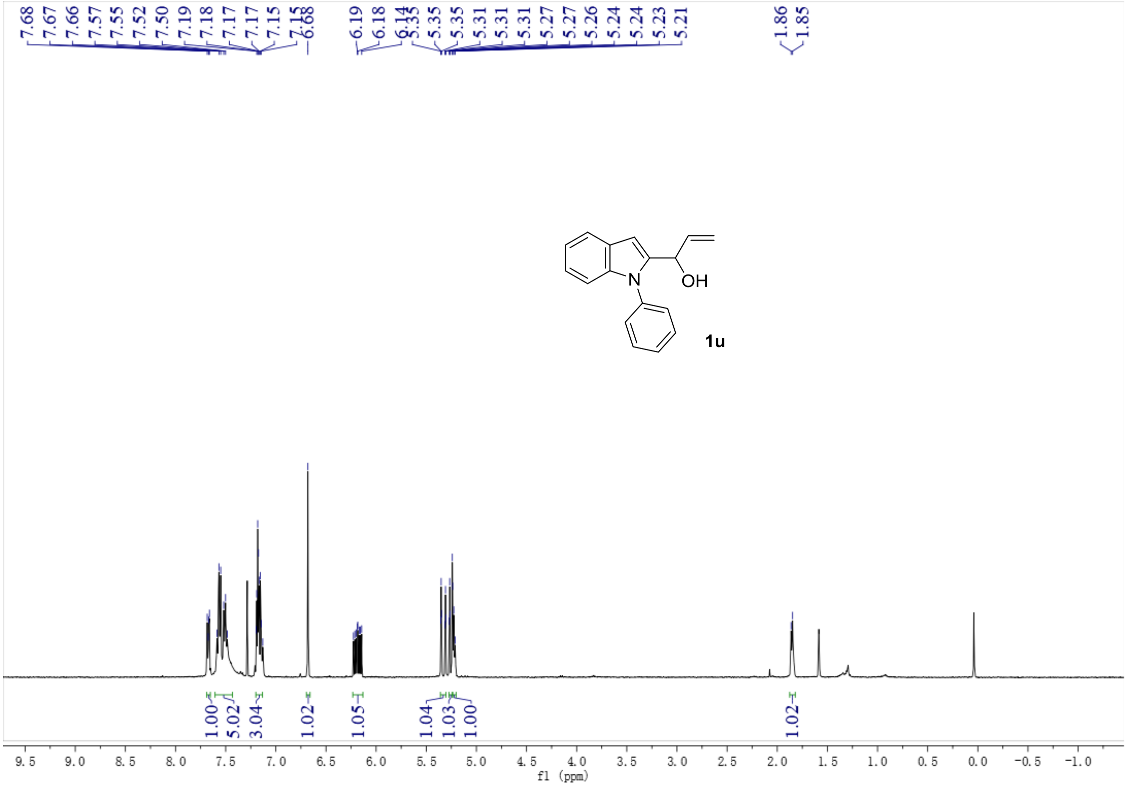

Supplementary Fig. 92 | ^{1}H NMR (400 MHz) of compound 1u (using $CDCl_3$ as solvent)

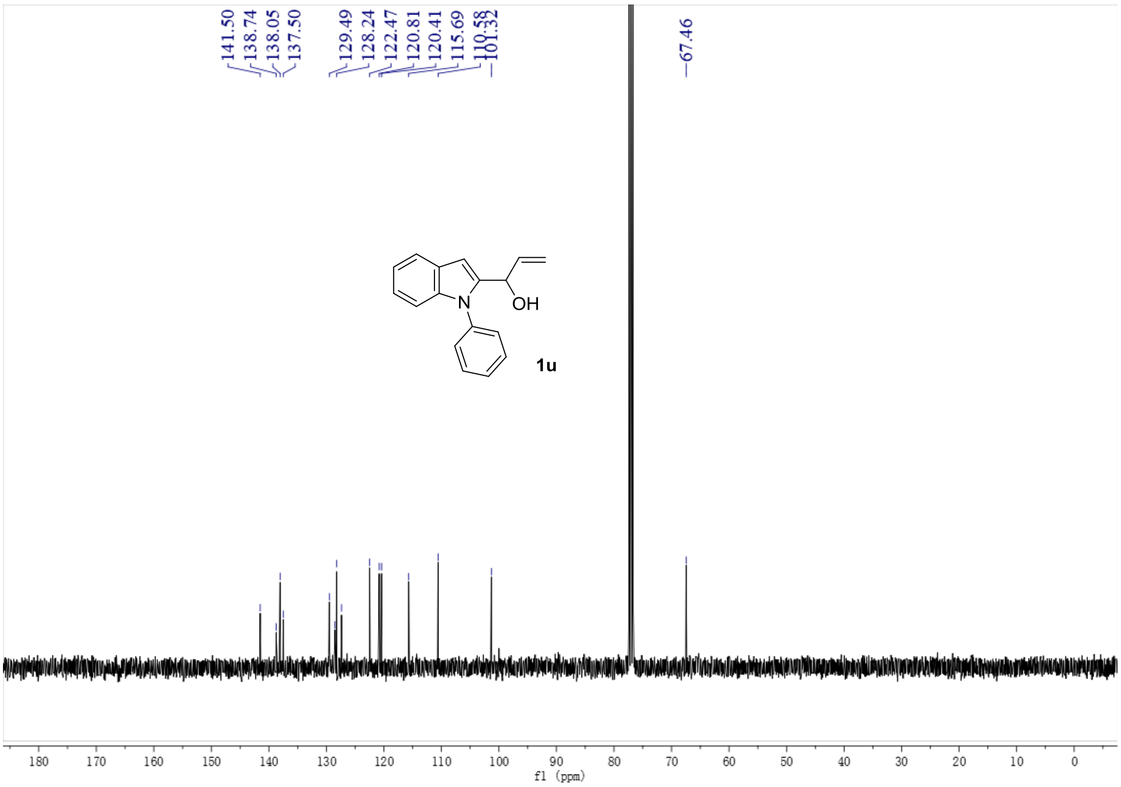

Supplementary Fig. 93 | ^{13}C NMR (101 MHz) of compound 1u (using $CDCl_3$ as solvent)

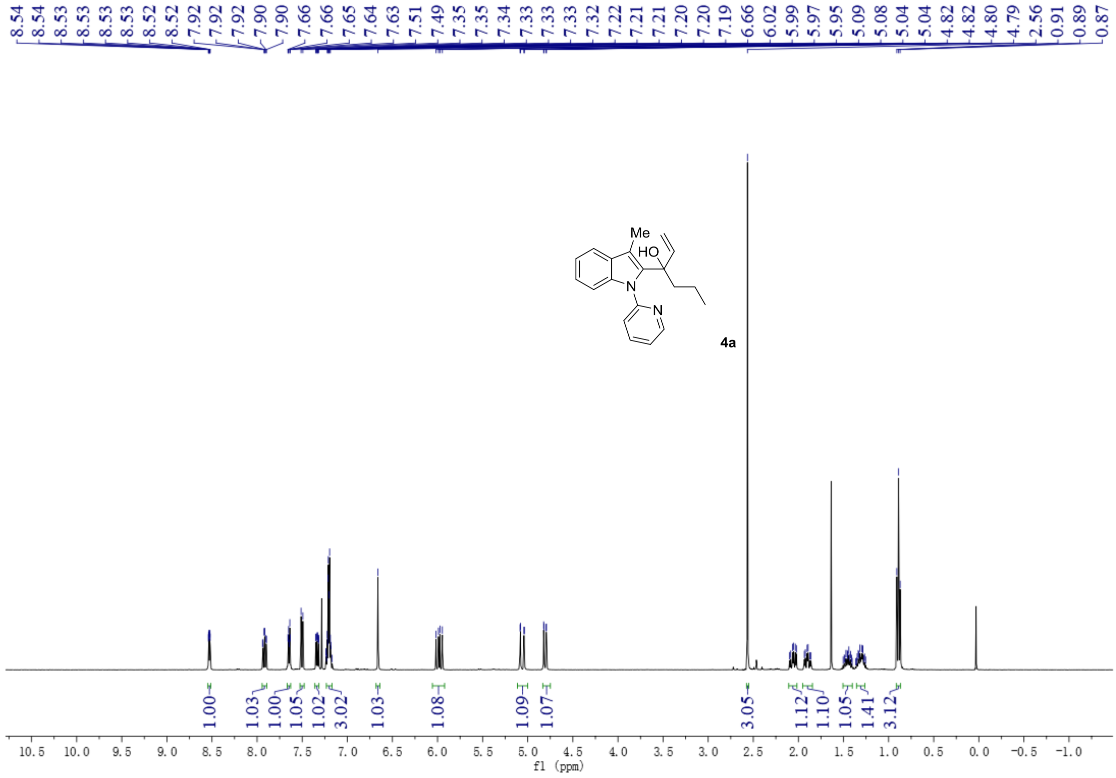

Supplementary Fig. 94 | ^{1}H NMR (400 MHz) of compound 4a (using $CDCl_3$ as solvent)

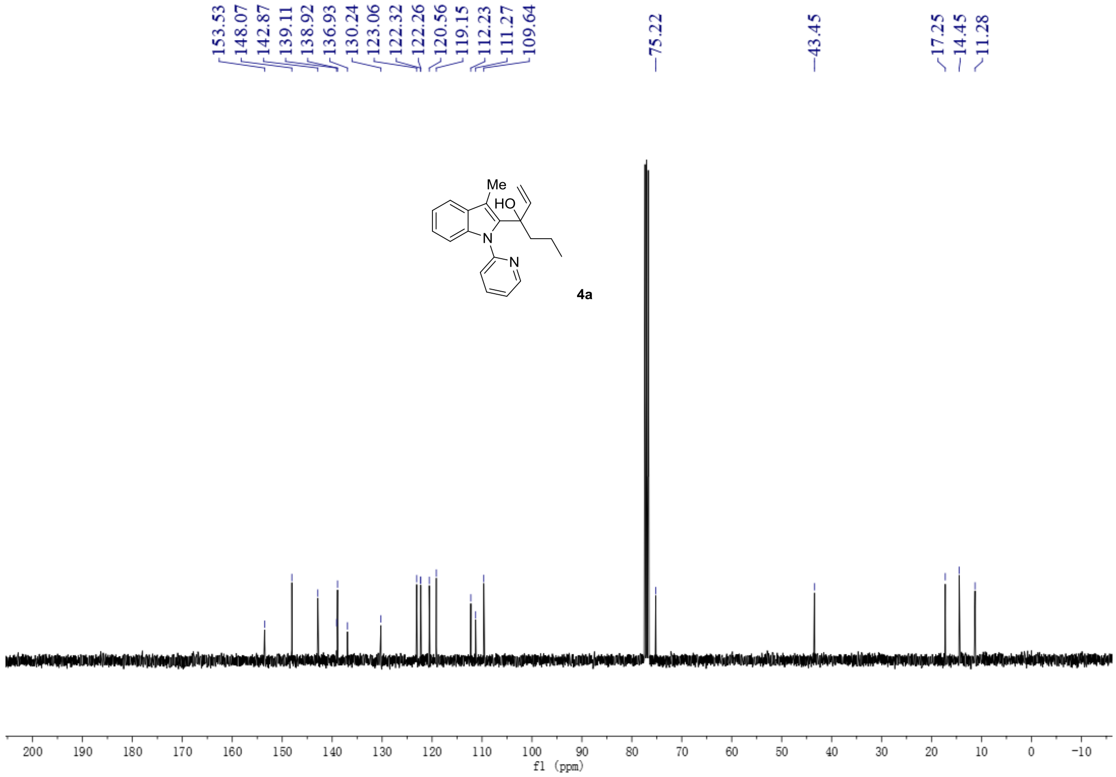

Supplementary Fig. 95 | ^{13}C NMR (101 MHz) of compound 4a (using $CDCl_3$ as solvent)

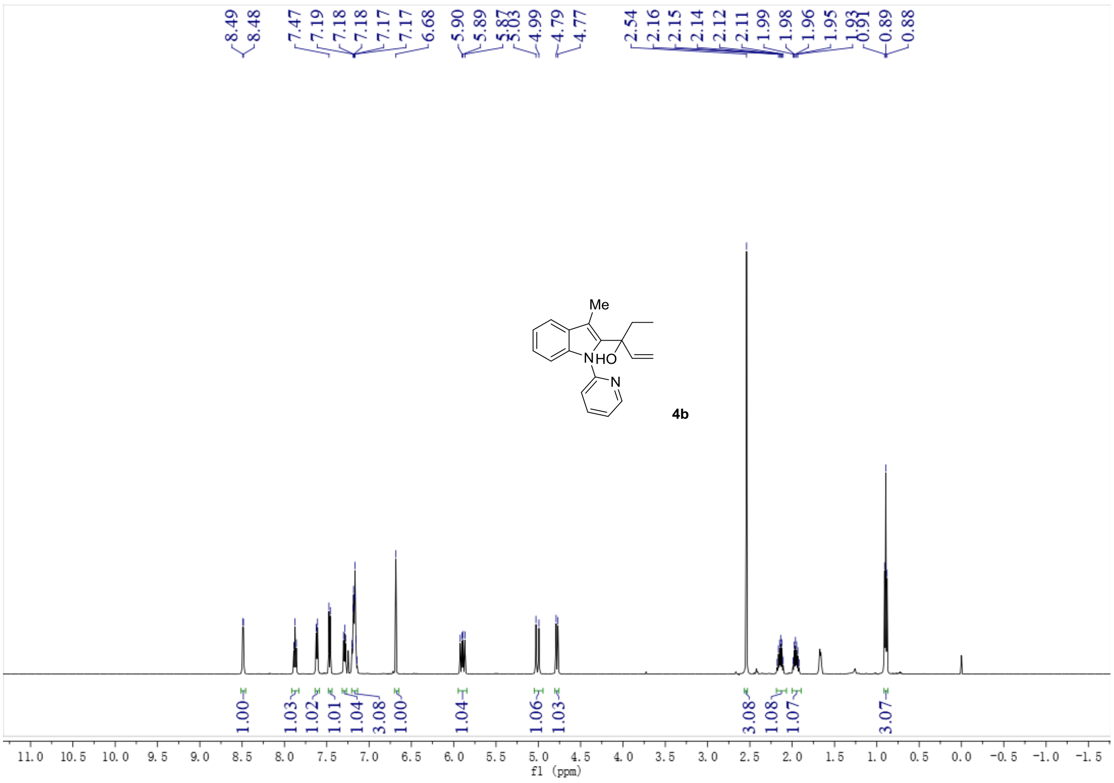

Supplementary Fig. 96 | ^{1}H NMR (500 MHz) of compound 4b (using $CDCl_3$ as solvent)

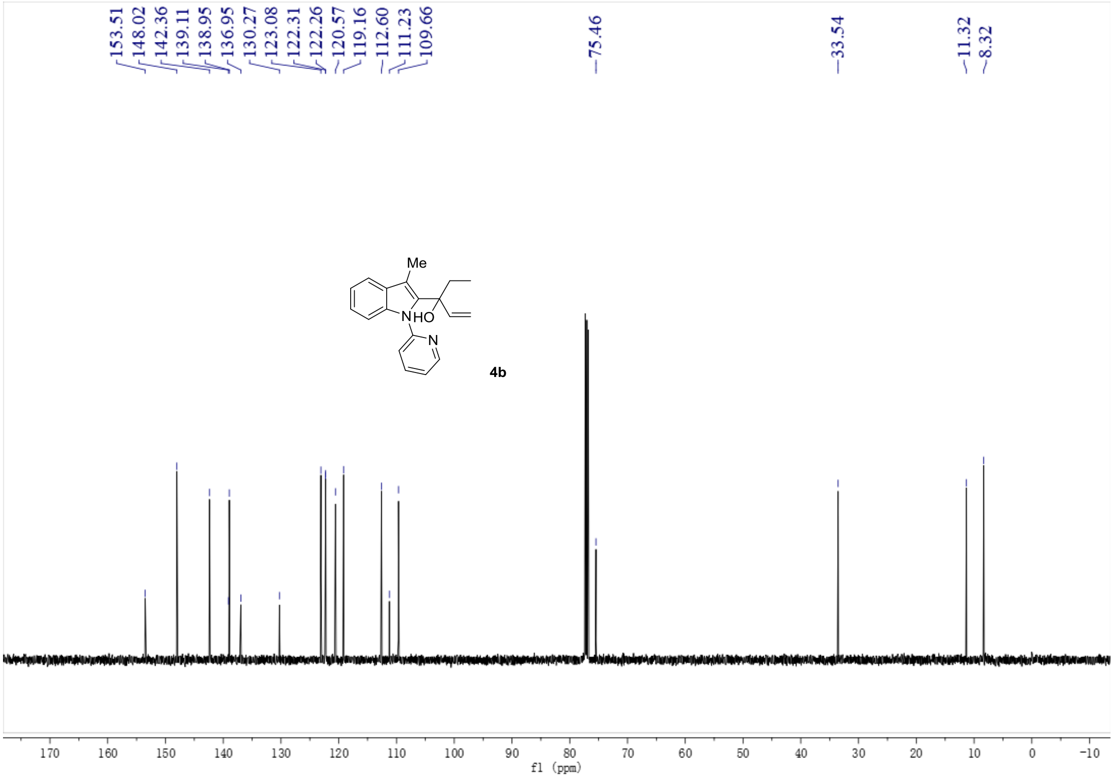

Supplementary Fig. 97 | ^{13}C NMR (126 MHz) of compound 4b (using $CDCl_3$ as solvent)

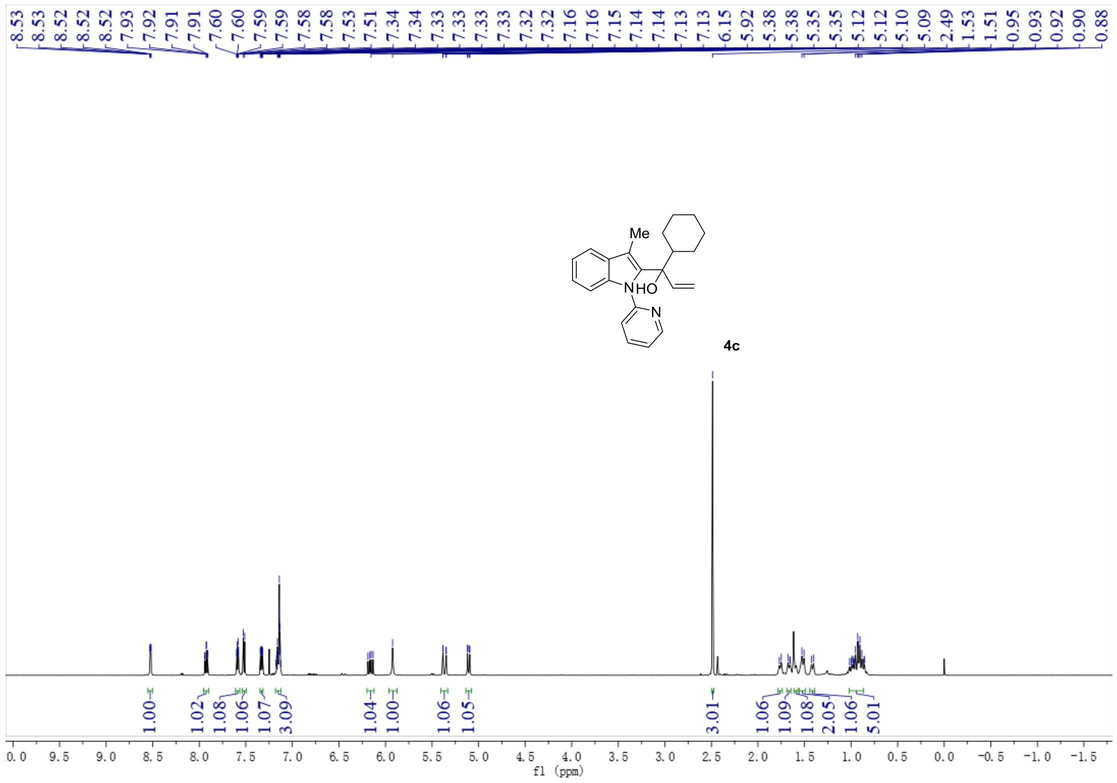

Supplementary Fig. 98 | ^{1}H NMR (500 MHz) of compound 4c (using $CDCl_3$ as solvent)

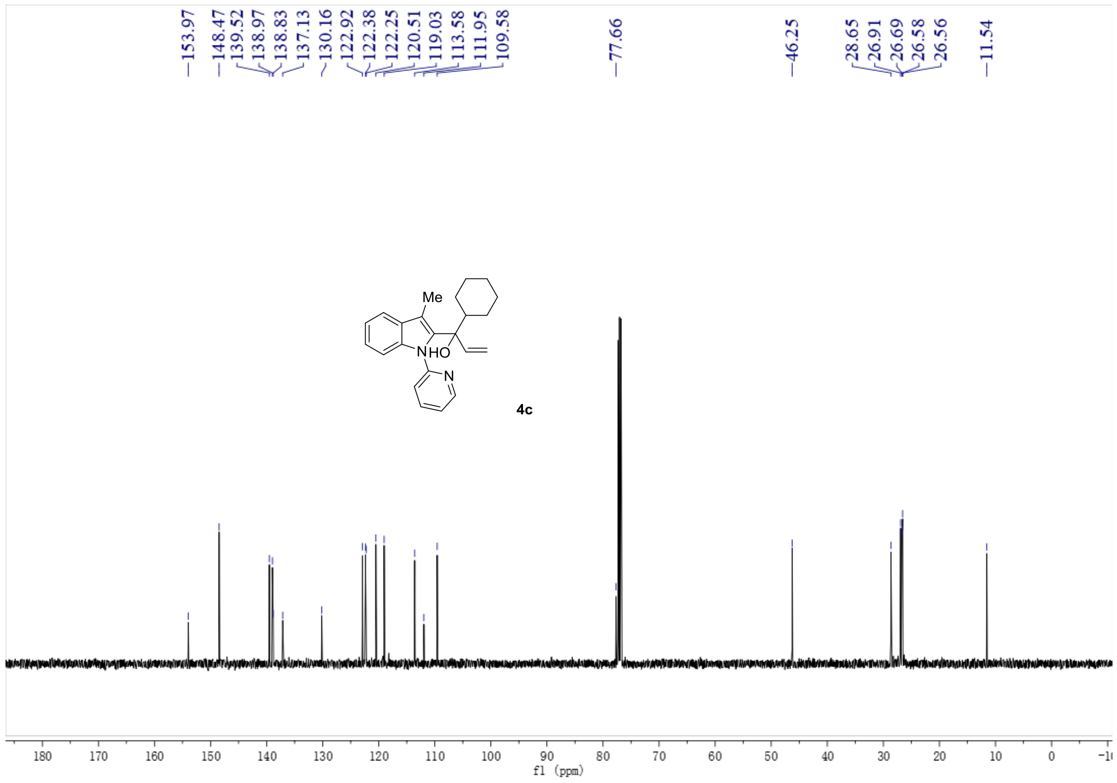

Supplementary Fig. 99 | ^{13}C NMR (126 MHz) of compound 4c (using $CDCl_3$ as solvent)

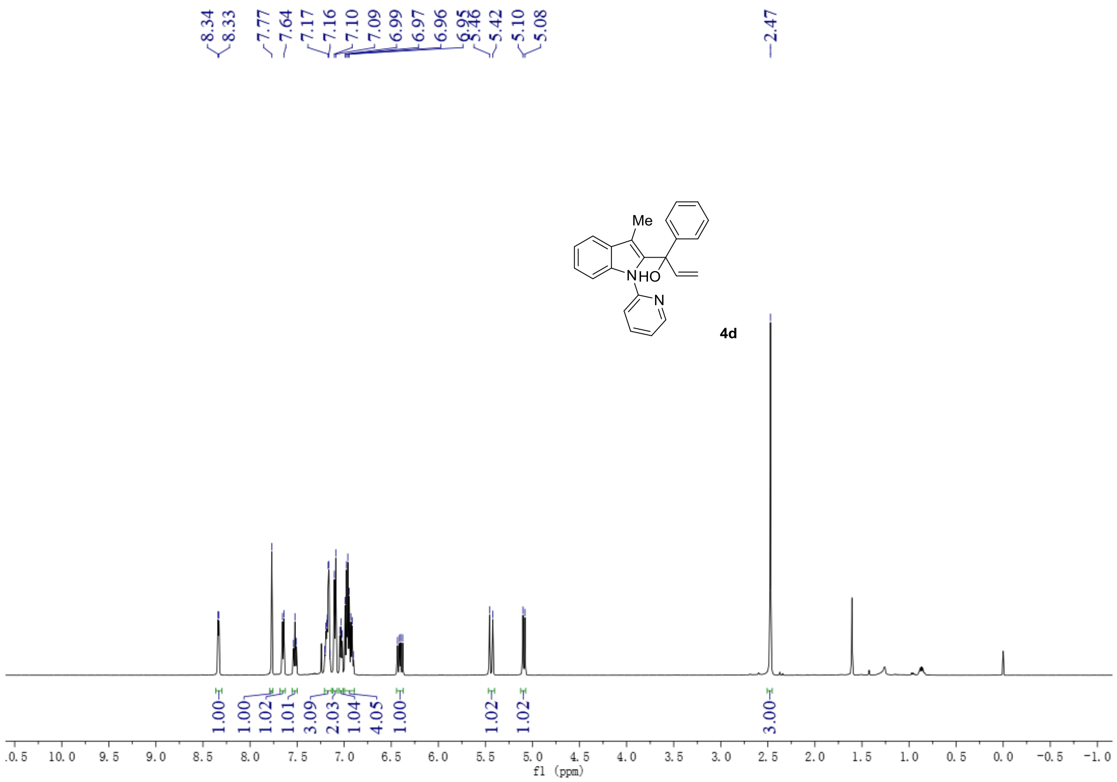

Supplementary Fig. 100 | ^{1}H NMR (500 MHz) of compound 4d (using $CDCl_3$ as solvent)

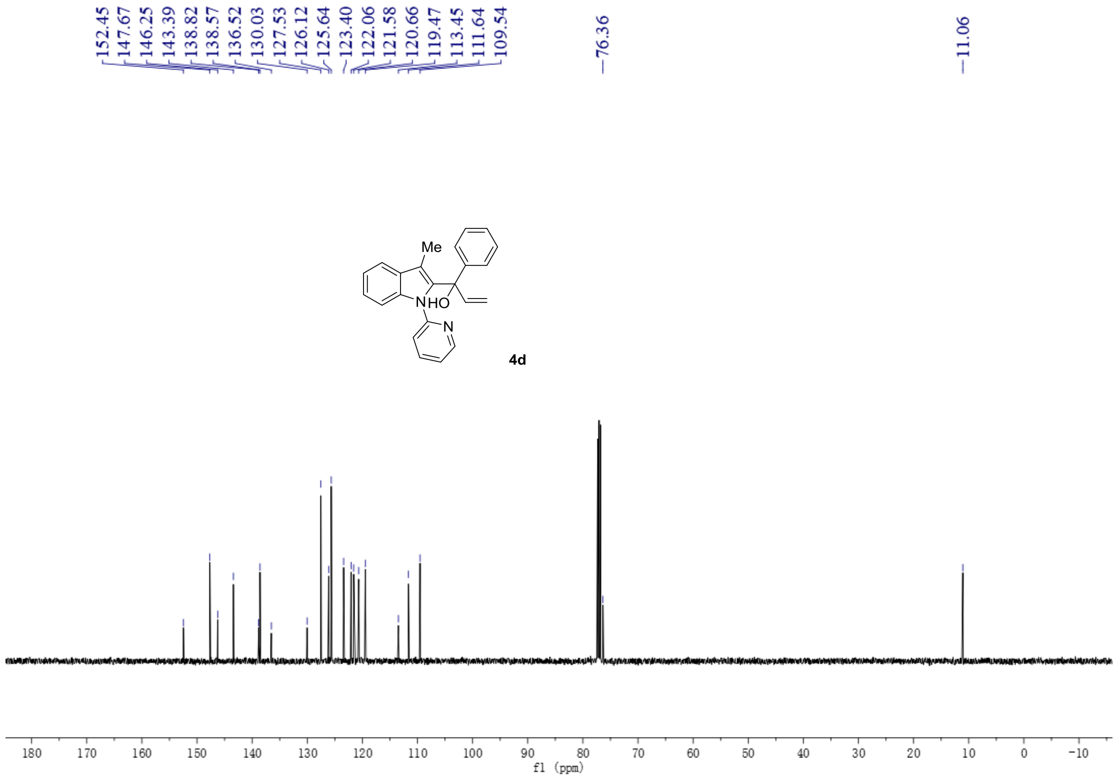

Supplementary Fig. 101 | ^{13}C NMR (126 MHz) of compound 4d (using $CDCl_3$ as solvent)

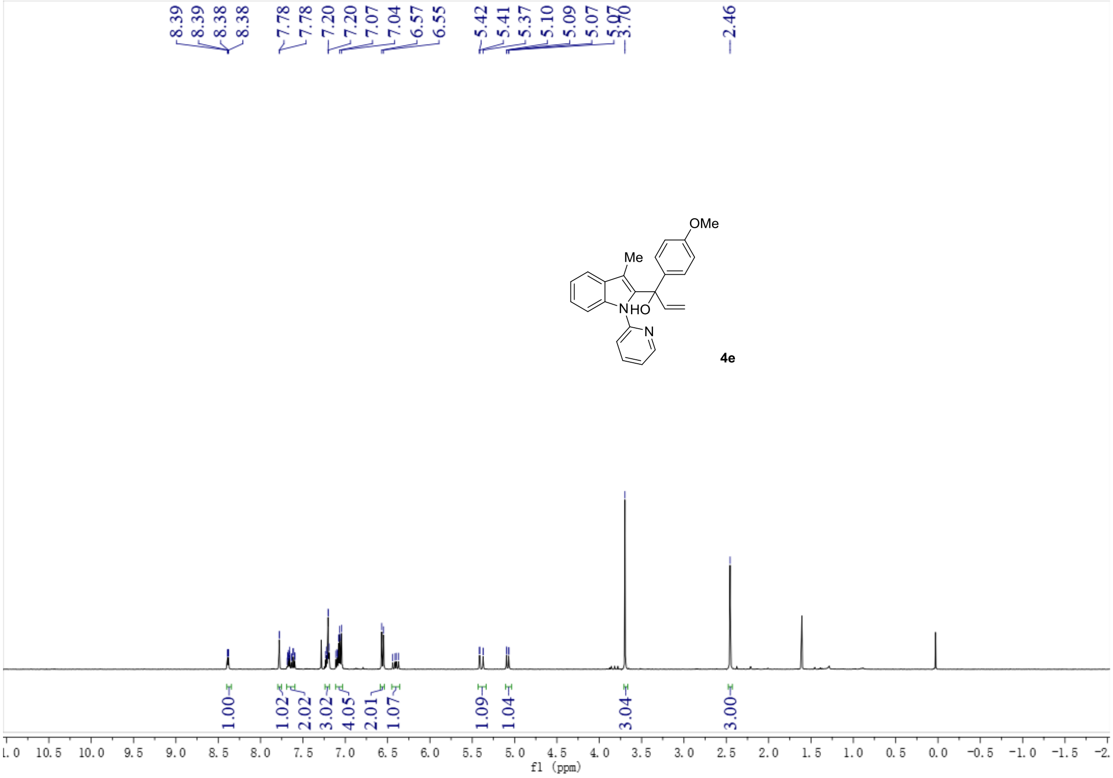

Supplementary Fig. 102 | ^{1}H NMR (400 MHz) of compound 4e (using $CDCl_3$ as solvent)

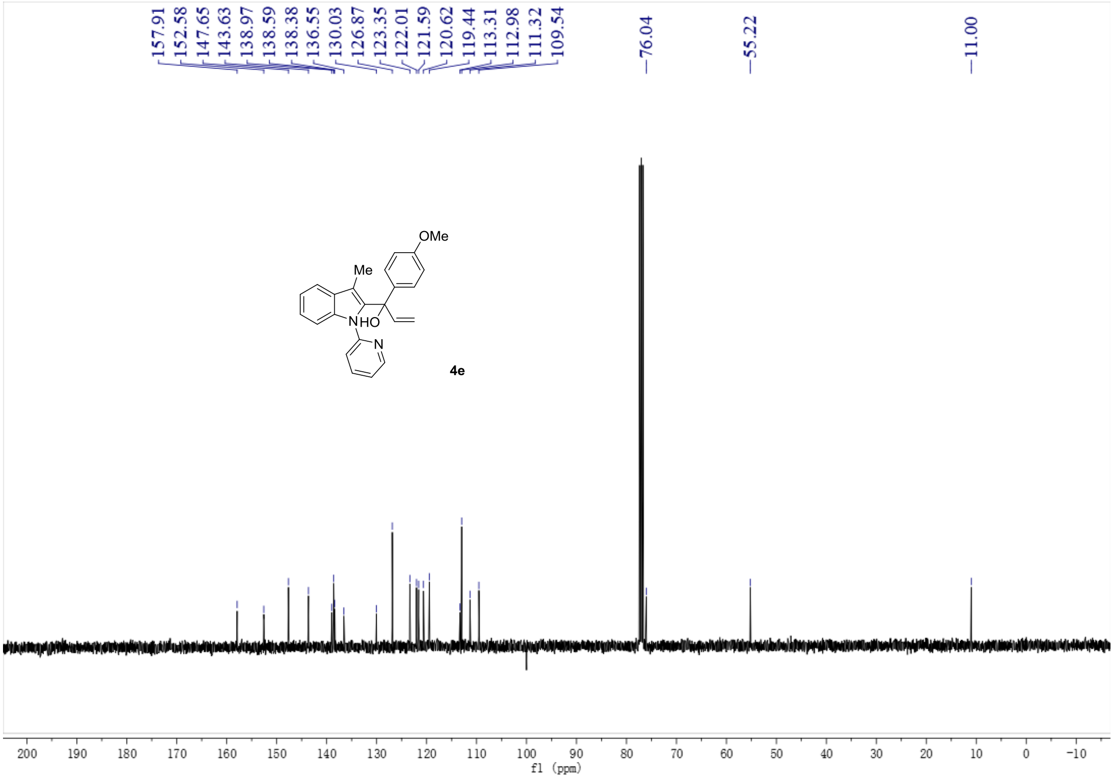

Supplementary Fig. 103 | ^{13}C NMR (101 MHz) of compound 4e (using $CDCl_3$ as solvent)

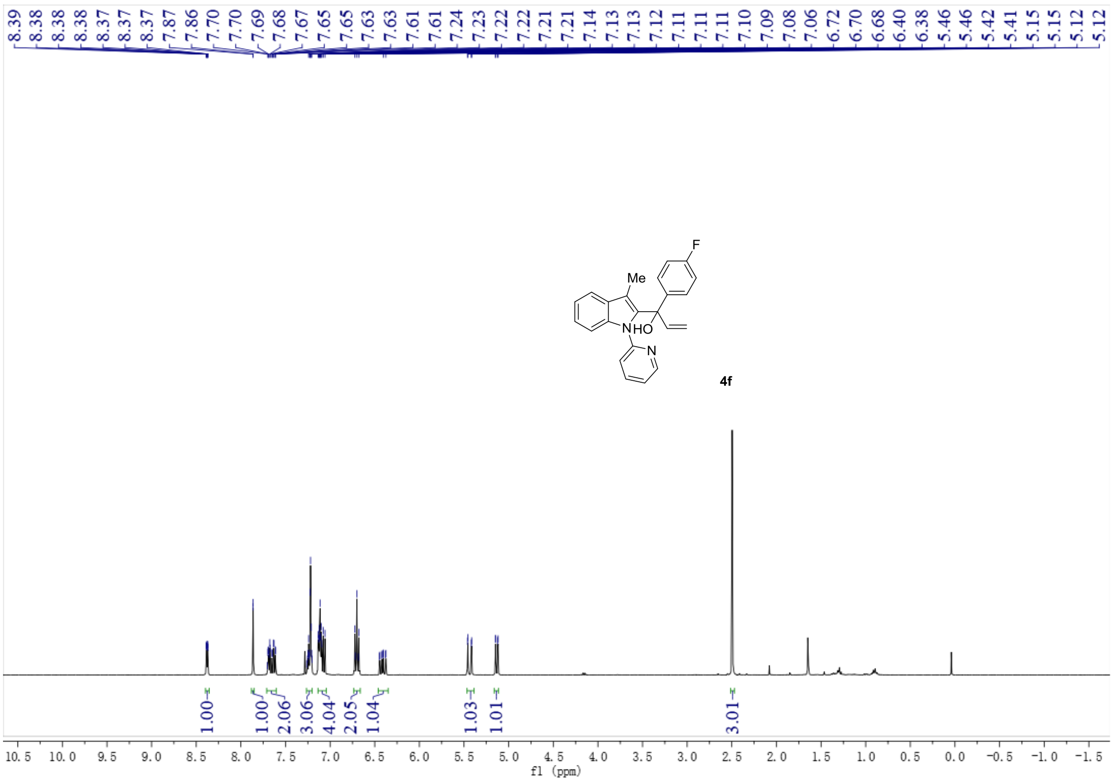

Supplementary Fig. 104 | ^{1}H NMR (400 MHz) of compound 4f (using $CDCl_3$ as solvent)

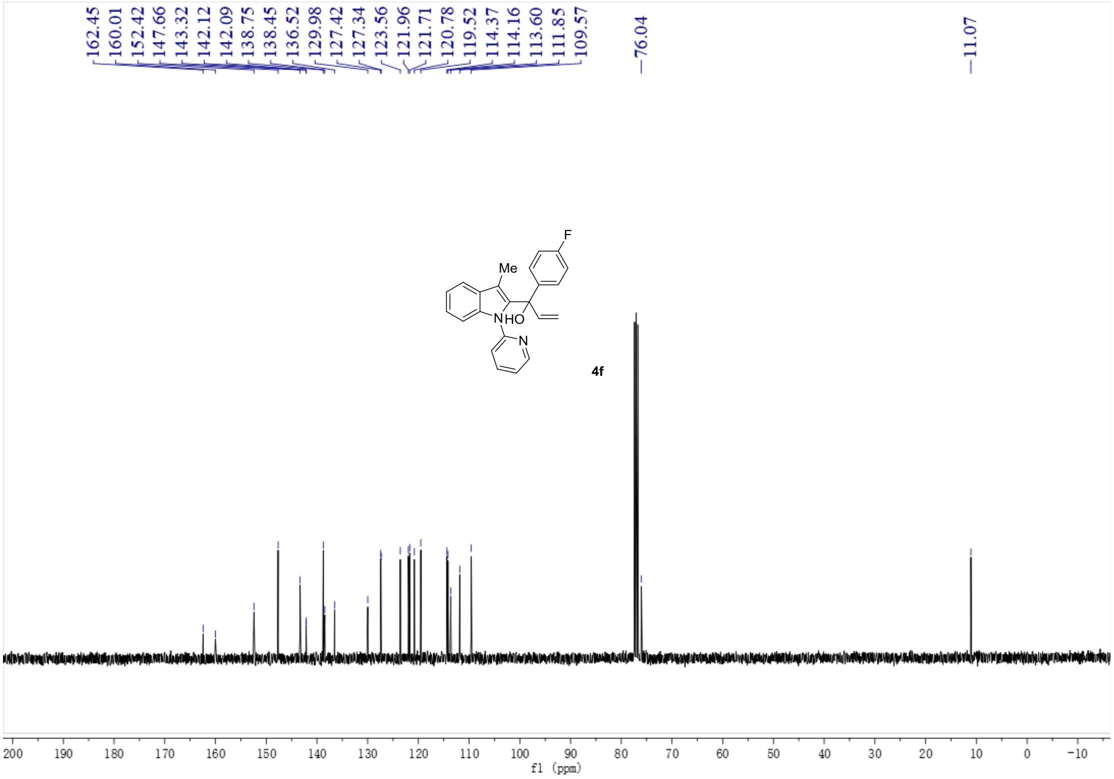

Supplementary Fig. 105 | ^{13}C NMR (101 MHz) of compound 4f (using $CDCl_3$ as solvent)

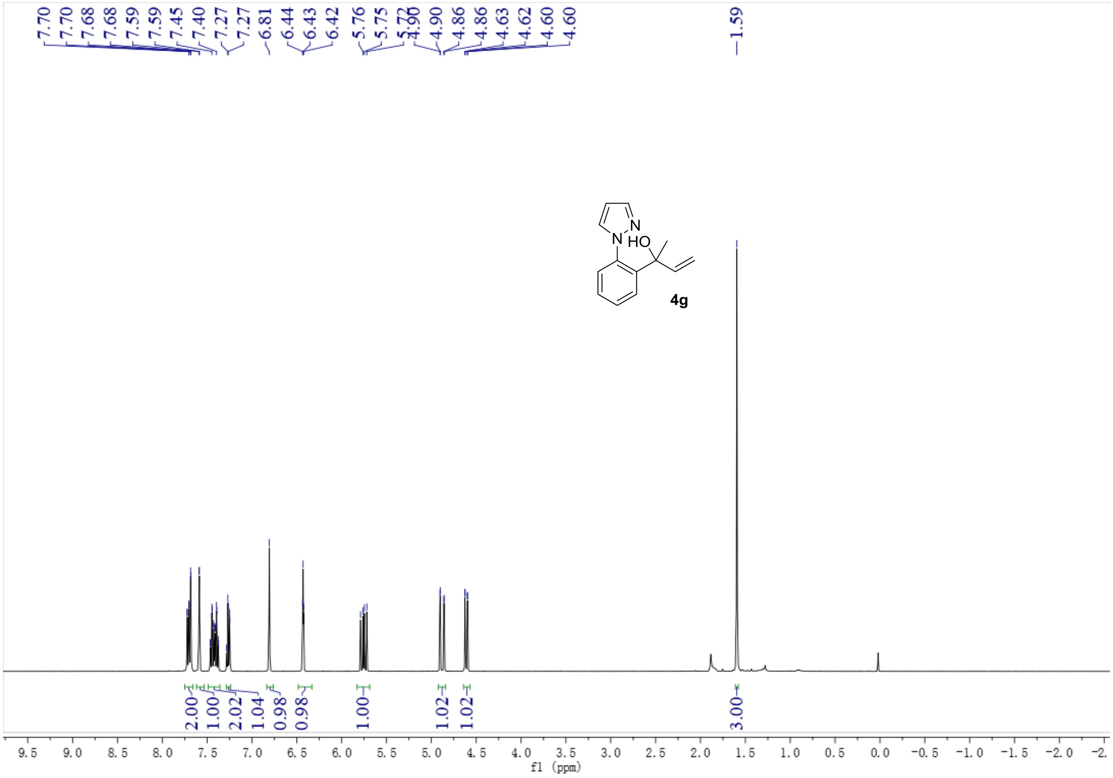

Supplementary Fig. 106 | ^{1}H NMR (400 MHz) of compound 4g (using $CDCl_3$ as solvent)

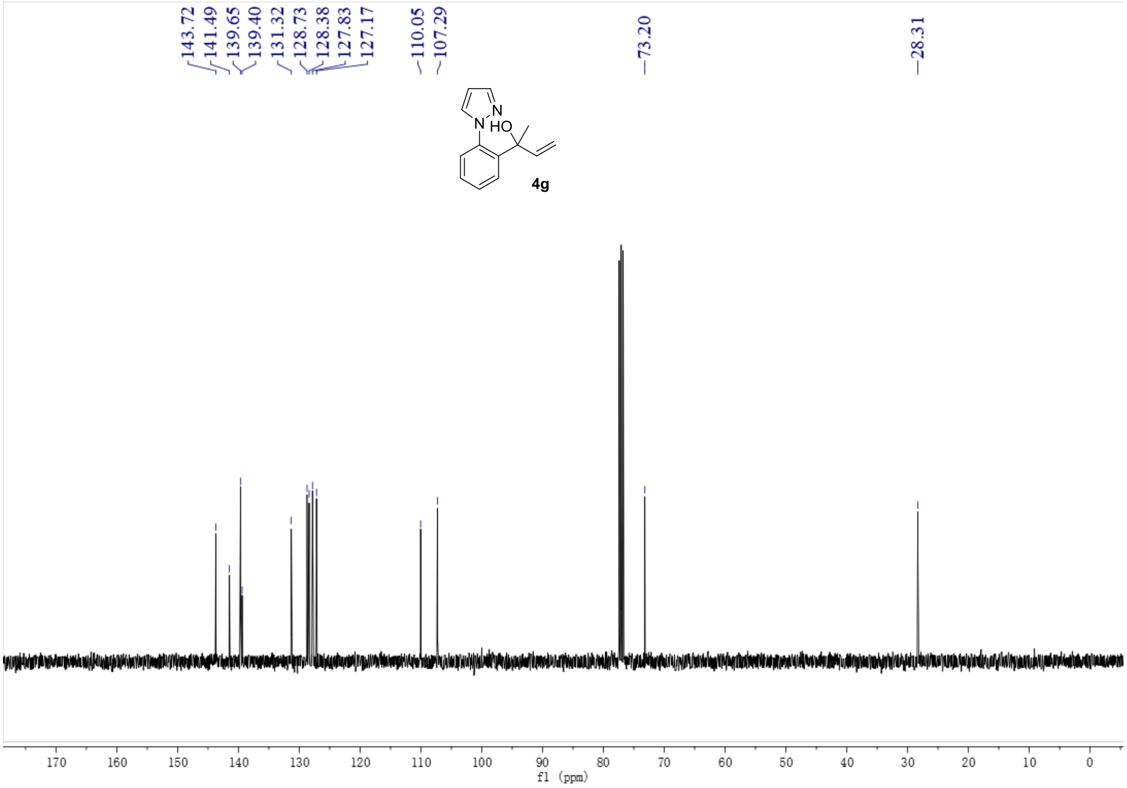

Supplementary Fig. 107 | ^{13}C NMR (101 MHz) of compound 4g (using $CDCl_3$ as solvent)

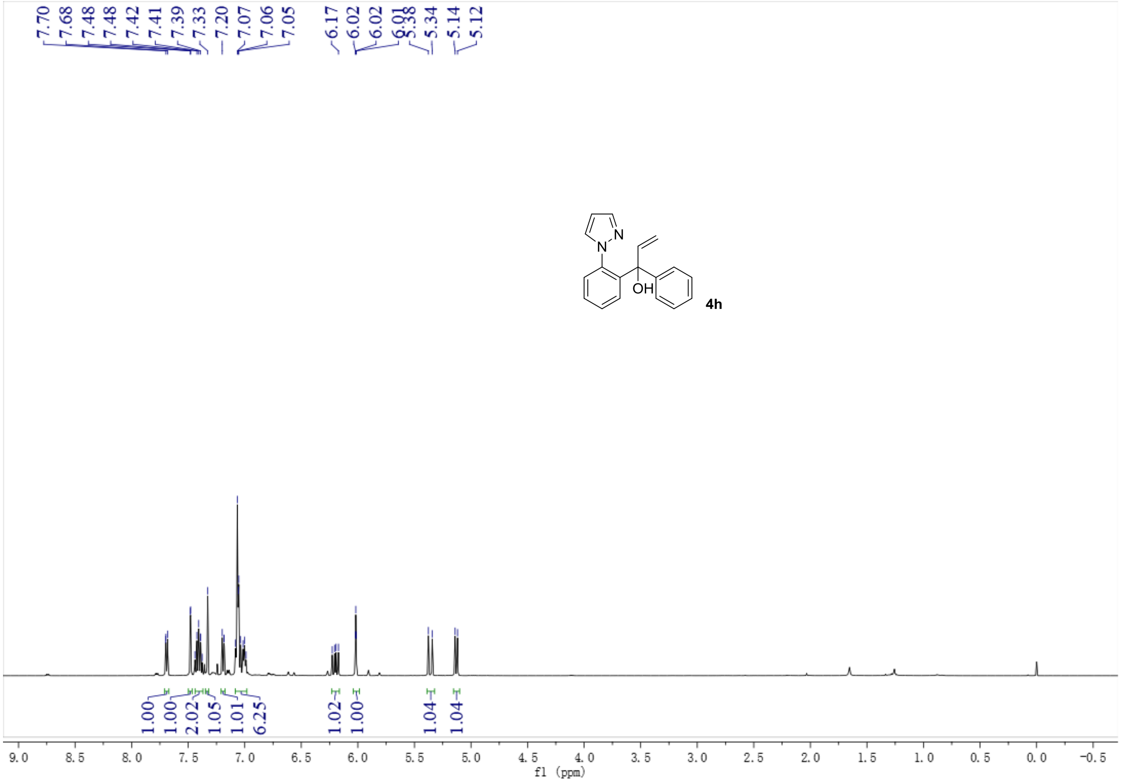

Supplementary Fig. 108 | ^{1}H NMR (500 MHz) of compound 4h (using $CDCl_3$ as solvent)

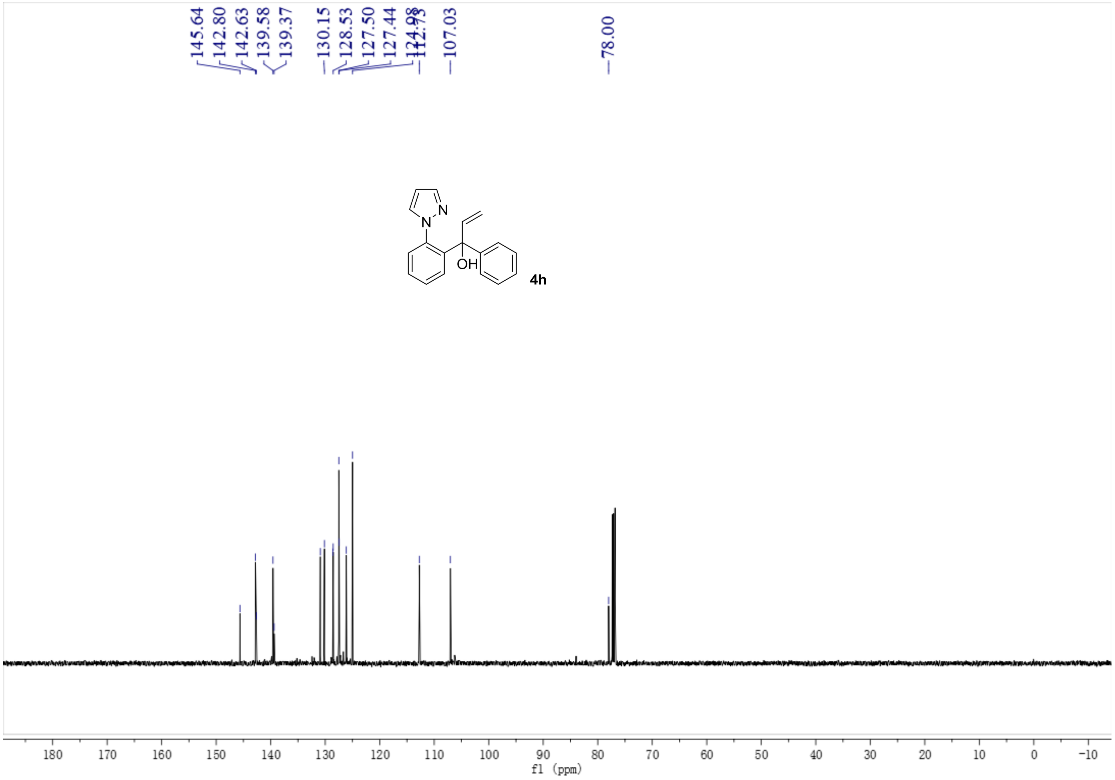

Supplementary Fig. 109 | ^{13}C NMR (126 MHz) of compound 4h (using $CDCl_3$ as solvent)

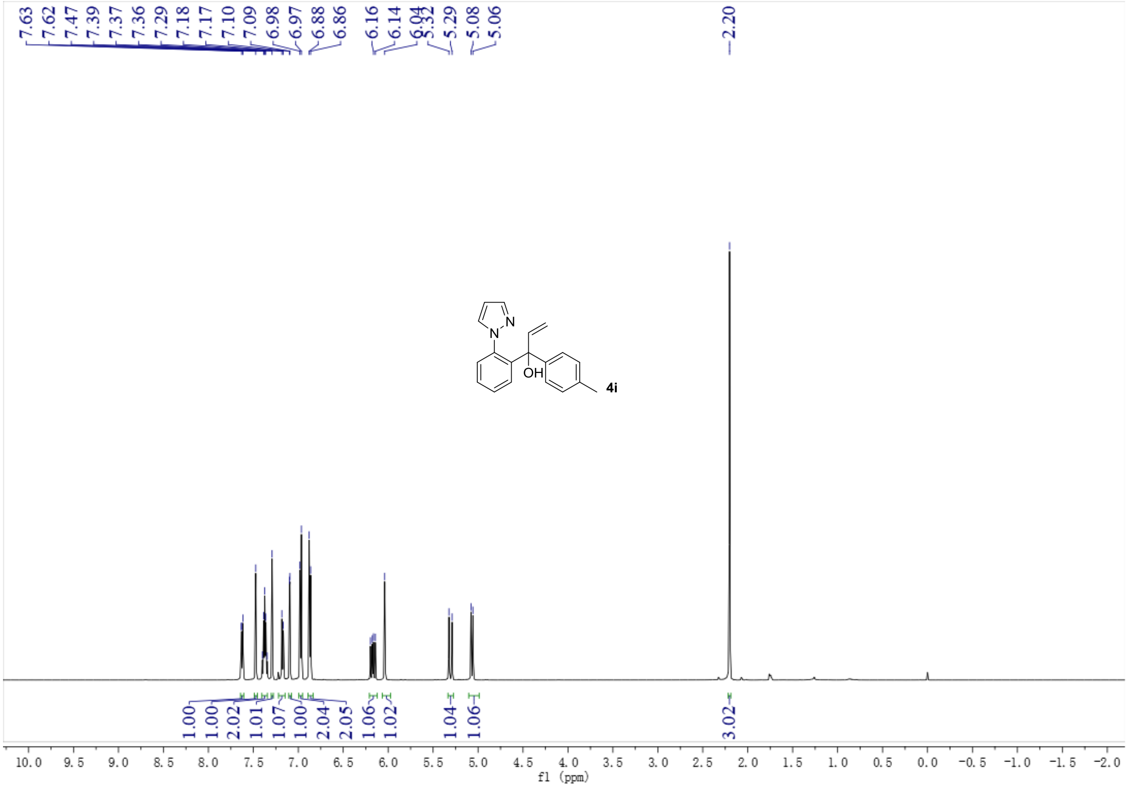

Supplementary Fig. 110 | ^{1}H NMR (500 MHz) of compound 4i (using $CDCl_3$ as solvent)

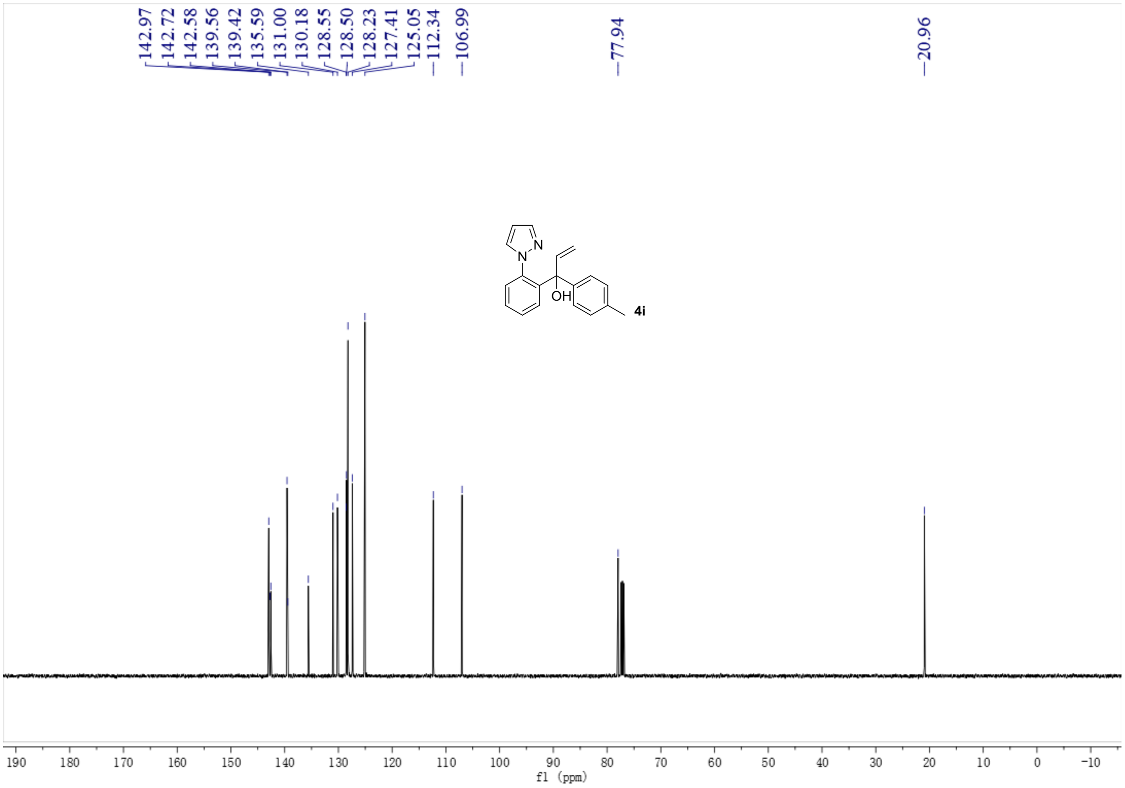

Supplementary Fig. 111 | ^{13}C NMR (126 MHz) of compound 4i (using $CDCl_3$ as solvent)

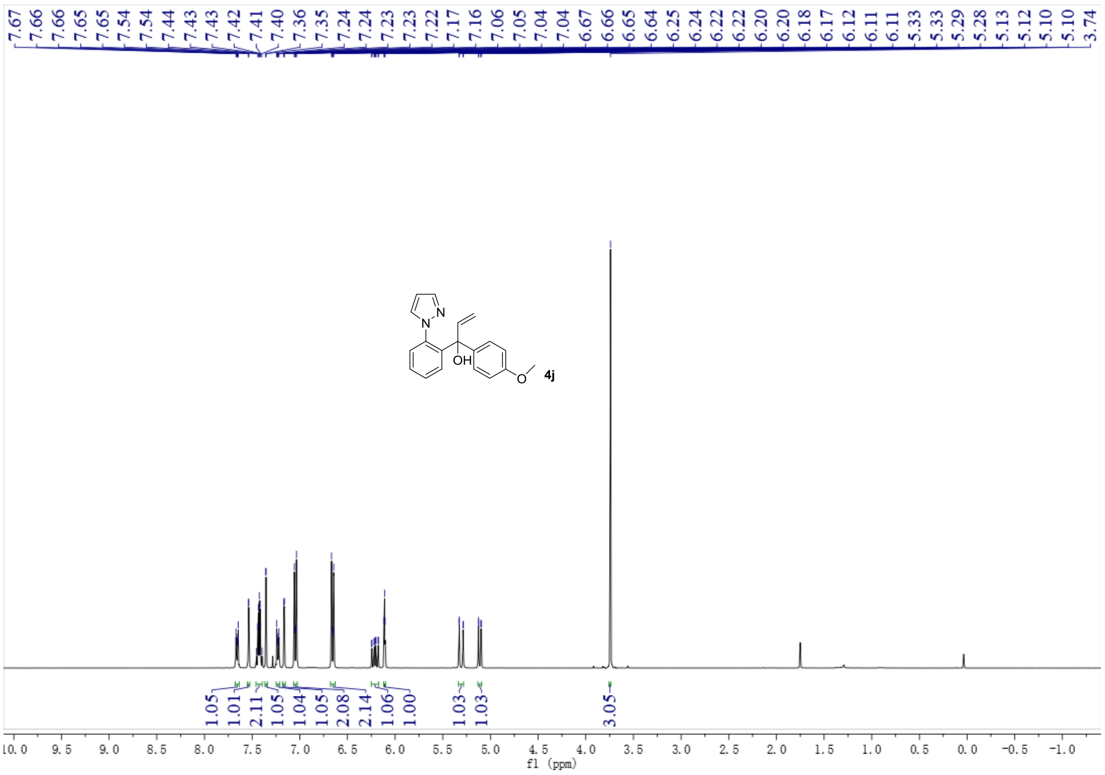

Supplementary Fig. 112 | ^{1}H NMR (400 MHz) of compound 4j (using $CDCl_3$ as solvent)

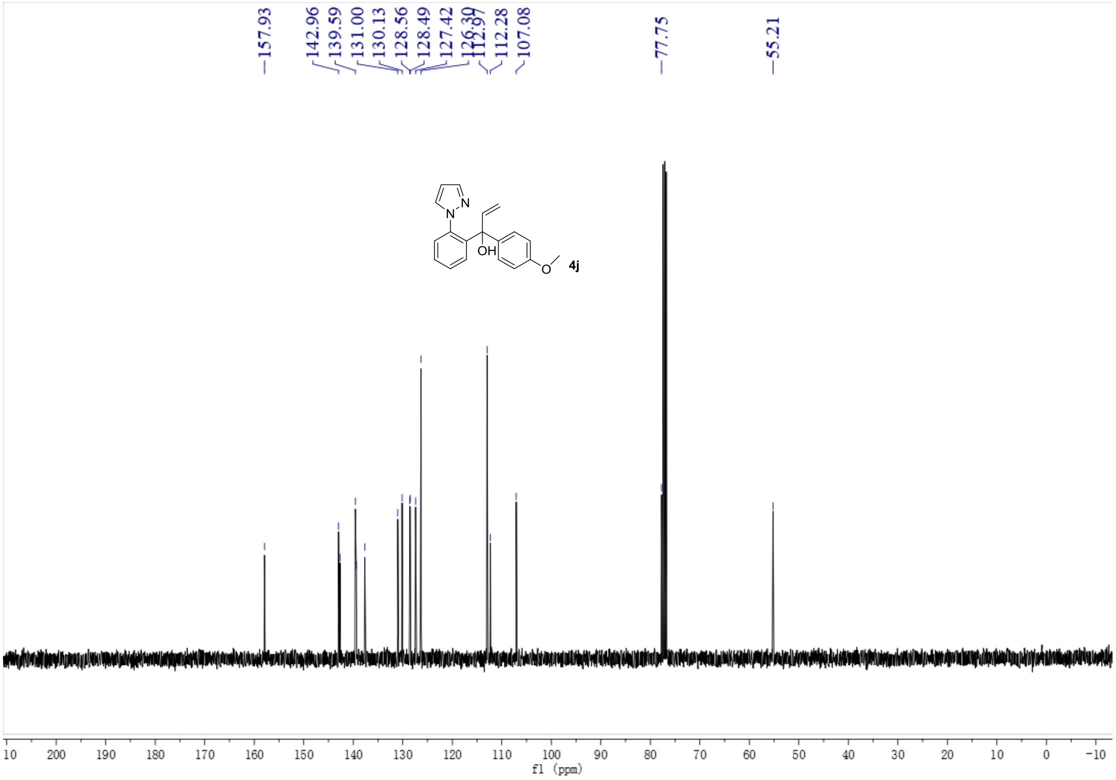

Supplementary Fig. 113 | ^{13}C NMR (101 MHz) of compound 4j (using $CDCl_3$ as solvent)

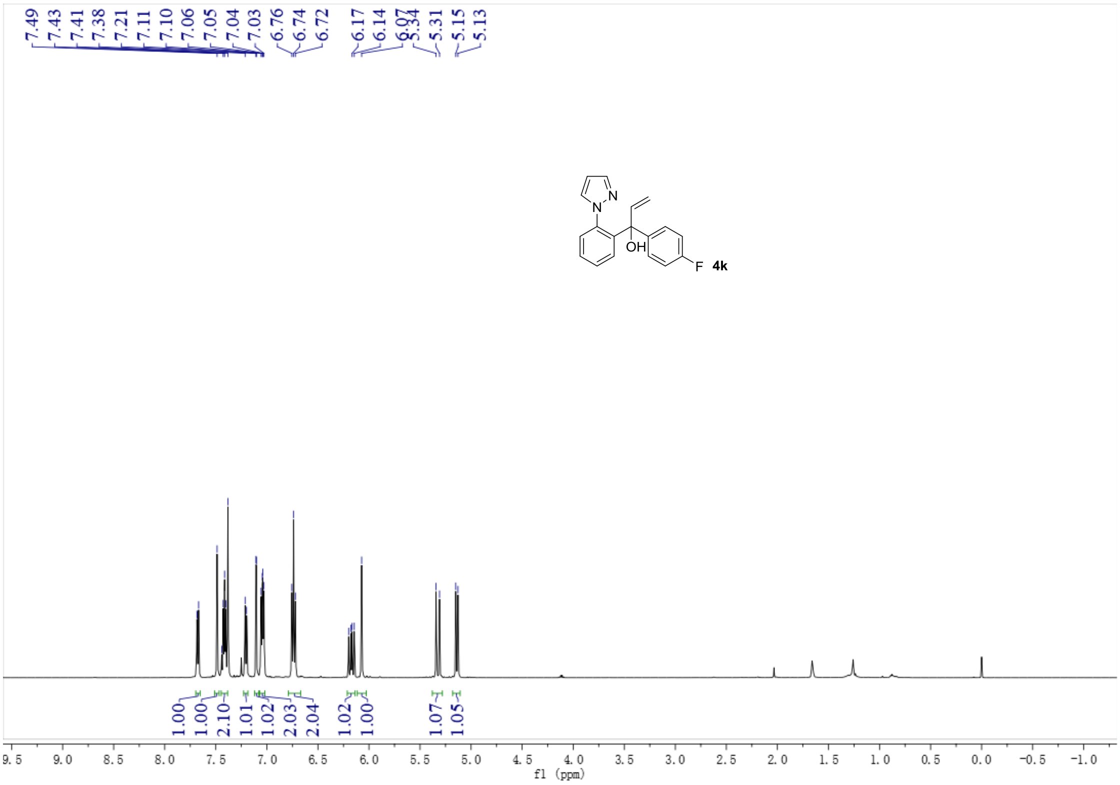

Supplementary Fig. 114 | ^{1}H NMR (500 MHz) of compound 4k (using $CDCl_3$ as solvent)

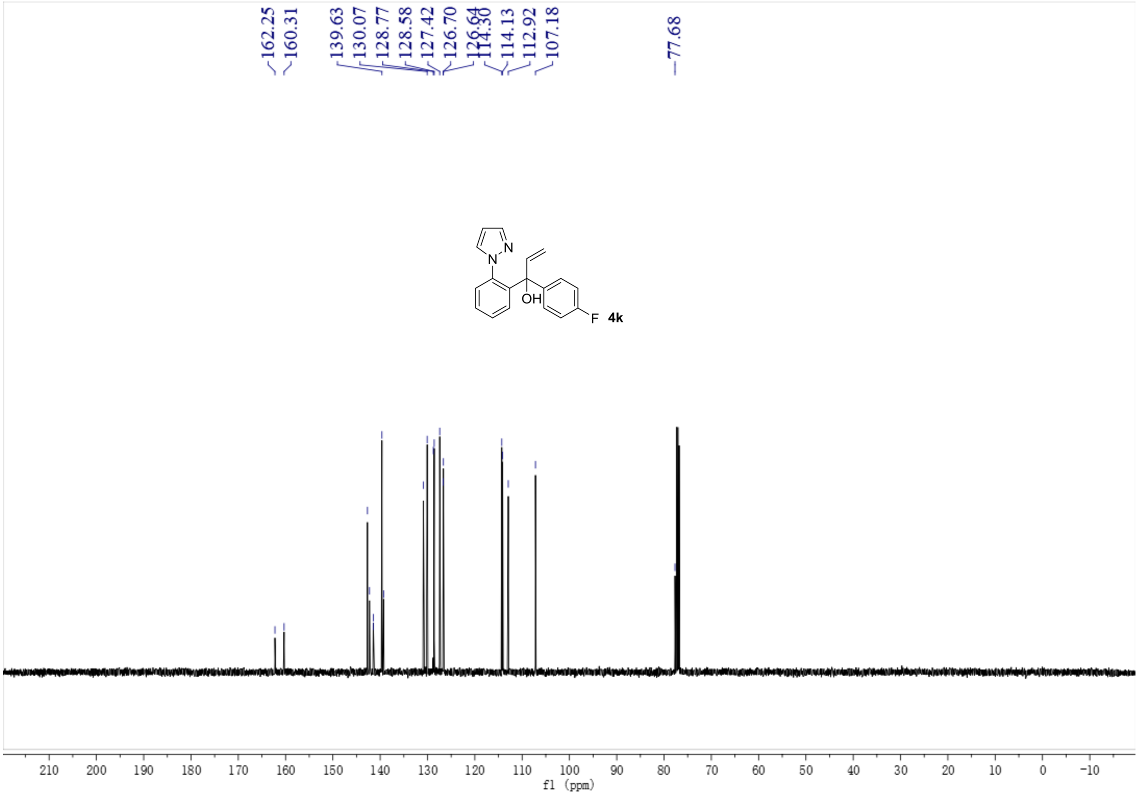

Supplementary Fig. 115 | ^{13}C NMR (126 MHz) of compound 4k (using $CDCl_3$ as solvent)

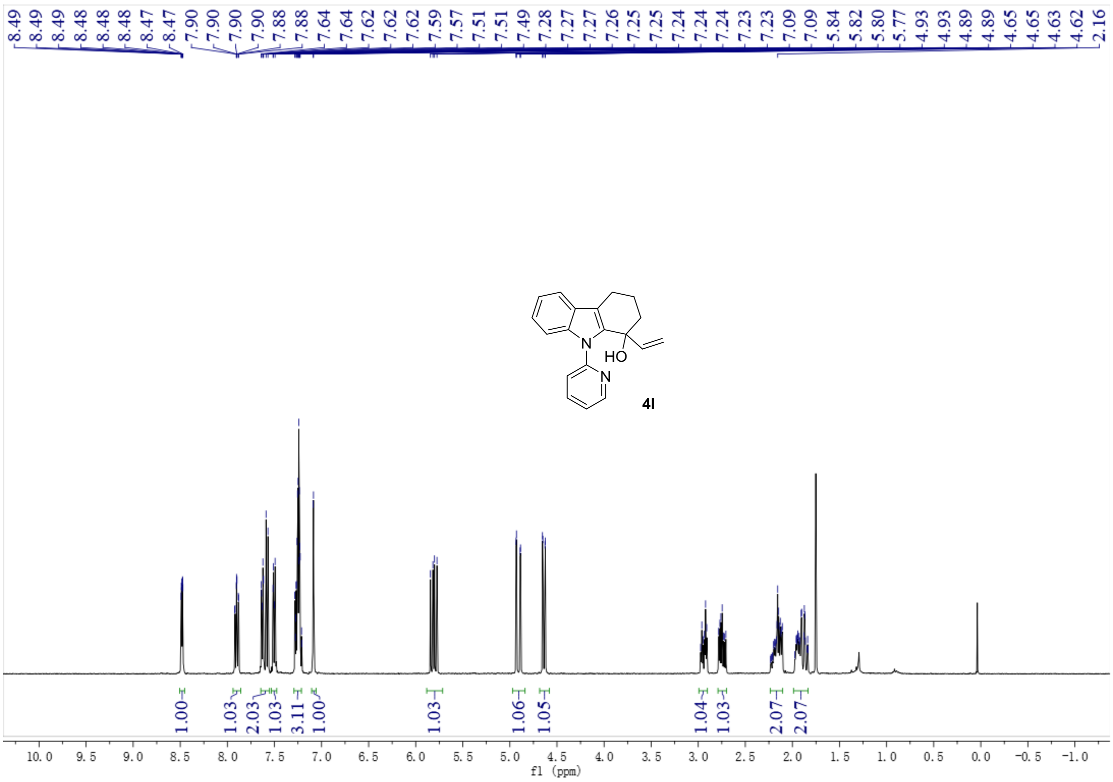

Supplementary Fig. 116 | ^{1}H NMR (400 MHz) of compound 4l (using $CDCl_3$ as solvent)

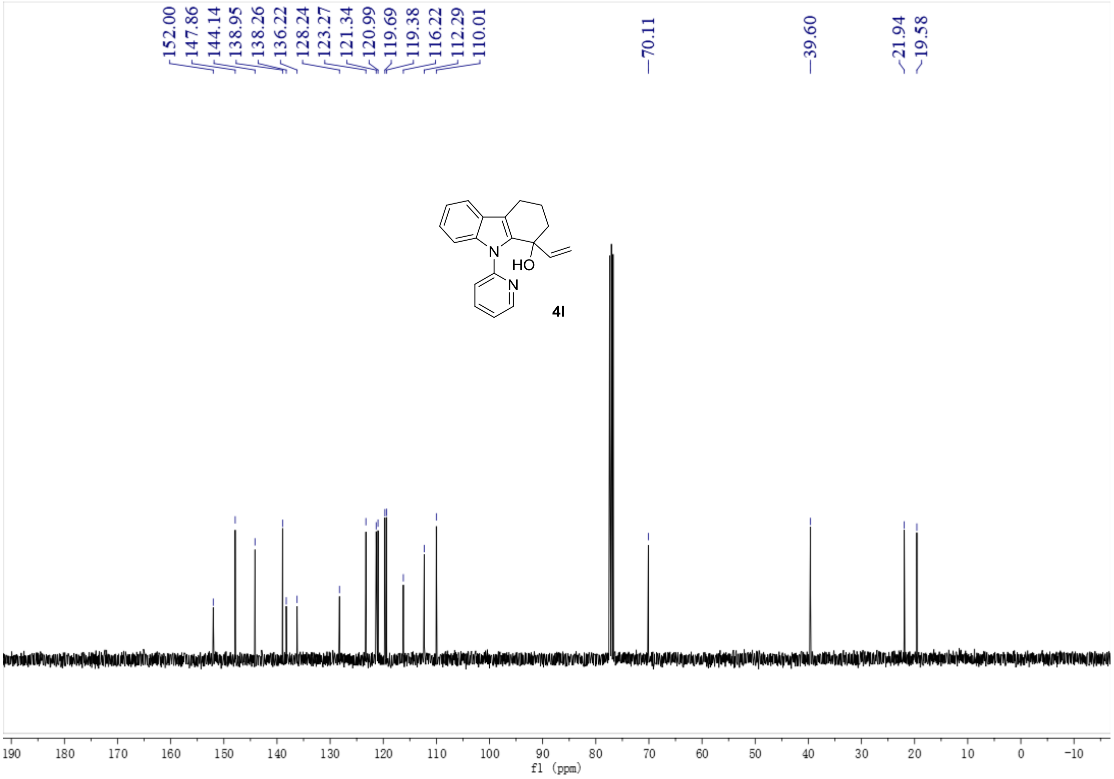

Supplementary Fig. 117 | ^{13}C NMR (101 MHz) of compound 4l (using $CDCl_3$ as solvent)

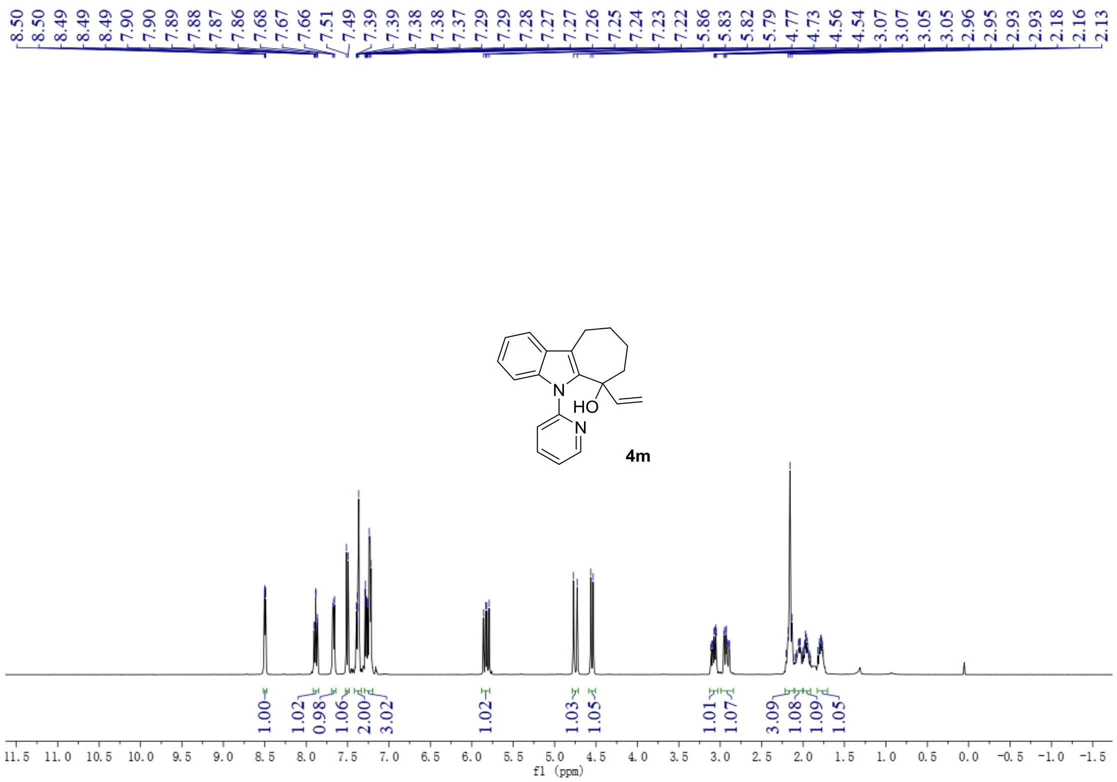

Supplementary Fig. 118 | ^{1}H NMR (400 MHz) of compound 4m (using $CDCl_3$ as solvent)

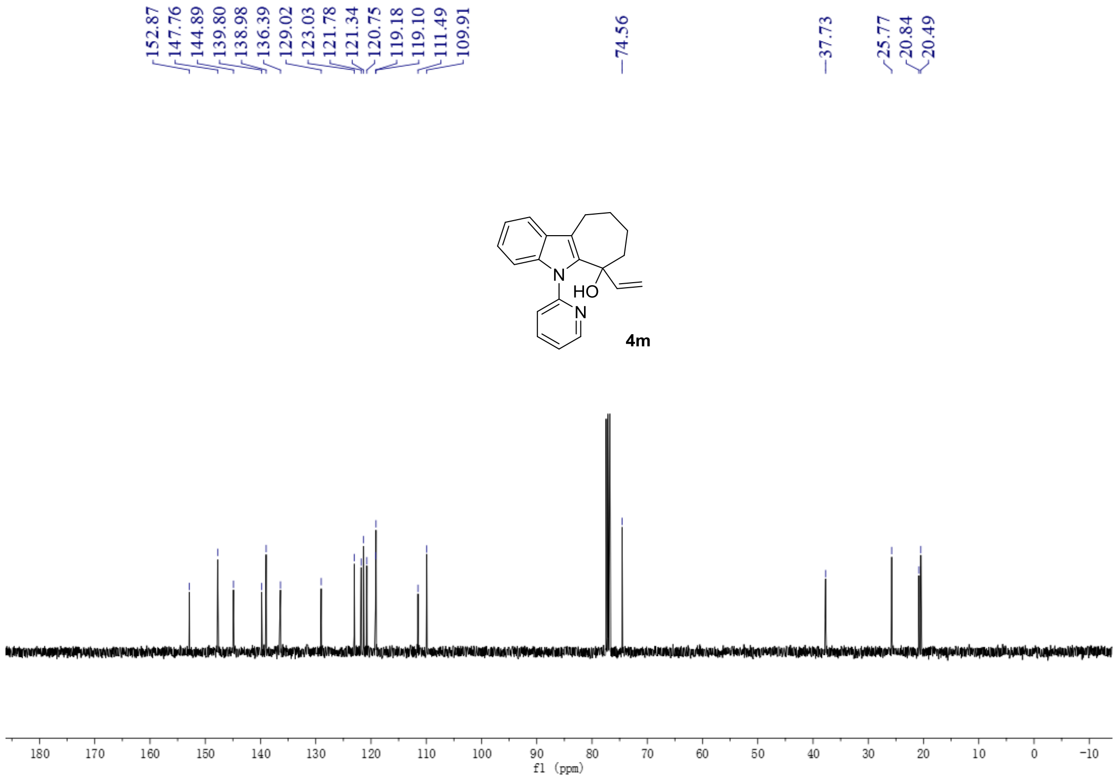

Supplementary Fig. 119 | ^{13}C NMR (101 MHz) of compound 4m (using $CDCl_3$ as solvent)

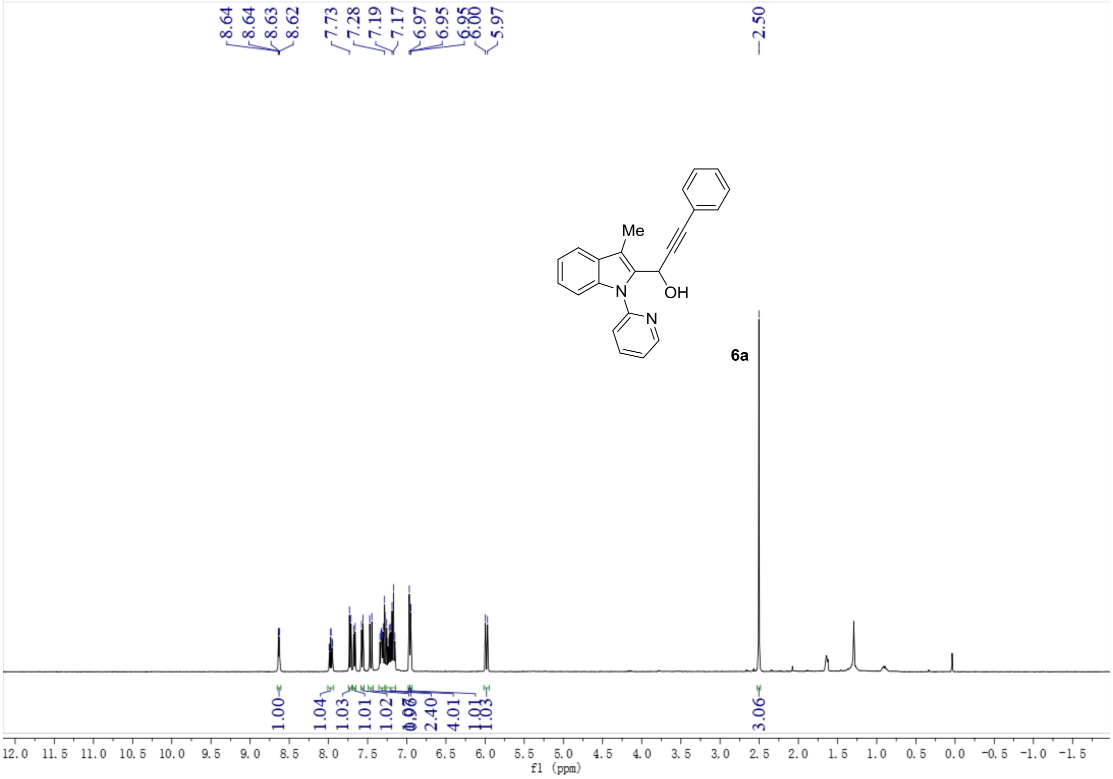

Supplementary Fig. 120 | ^{1}H NMR (400 MHz) of compound 6a (using $CDCl_3$ as solvent)

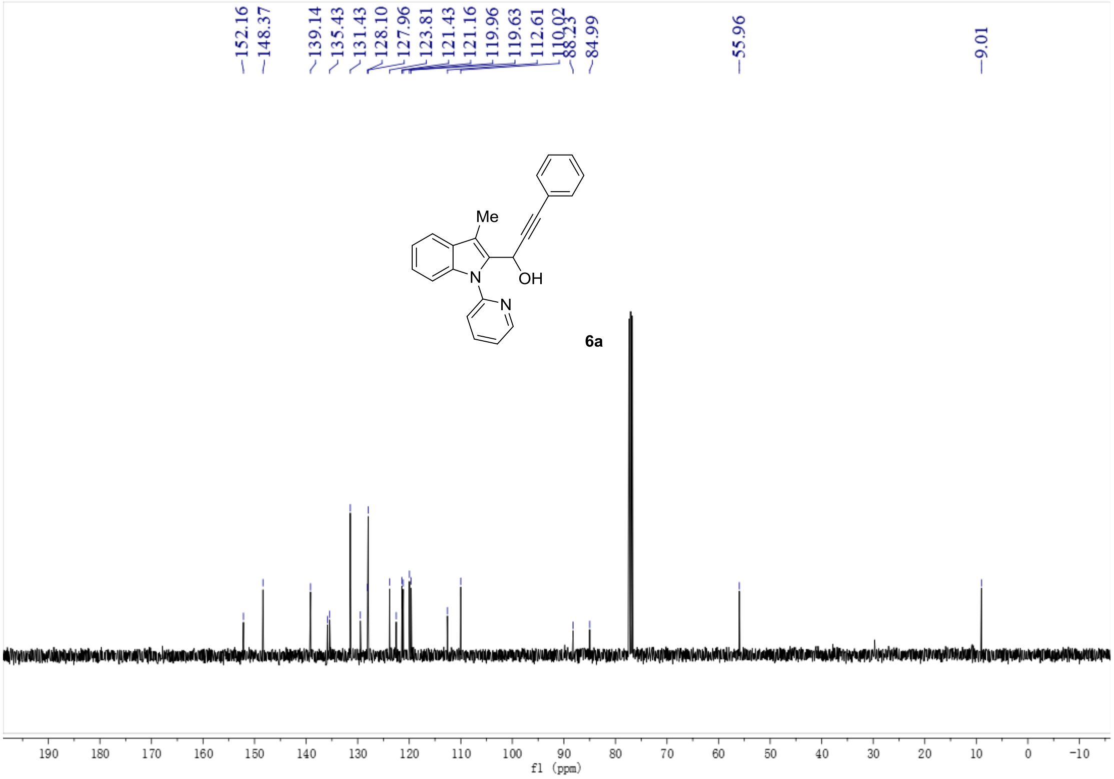

Supplementary Fig. 121 | ^{13}C NMR (101 MHz) of compound 6a (using $CDCl_3$ as solvent)

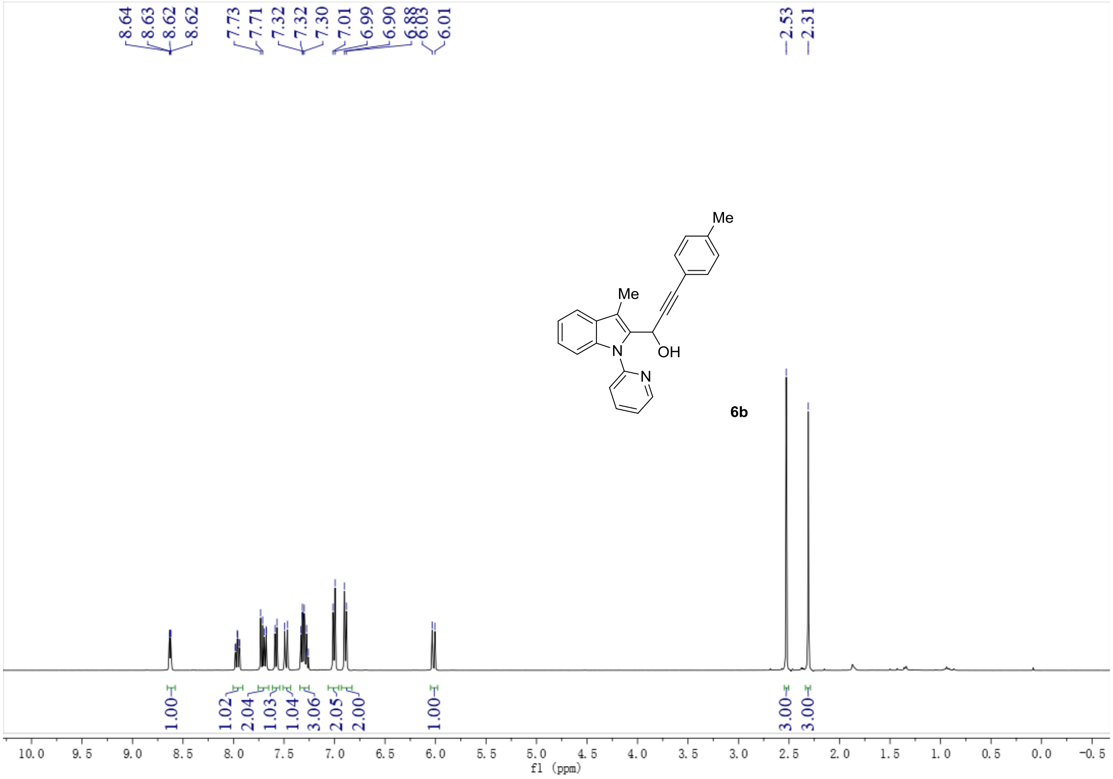

Supplementary Fig. 122 | ^{1}H NMR (400 MHz) of compound 6b (using $CDCl_3$ as solvent)

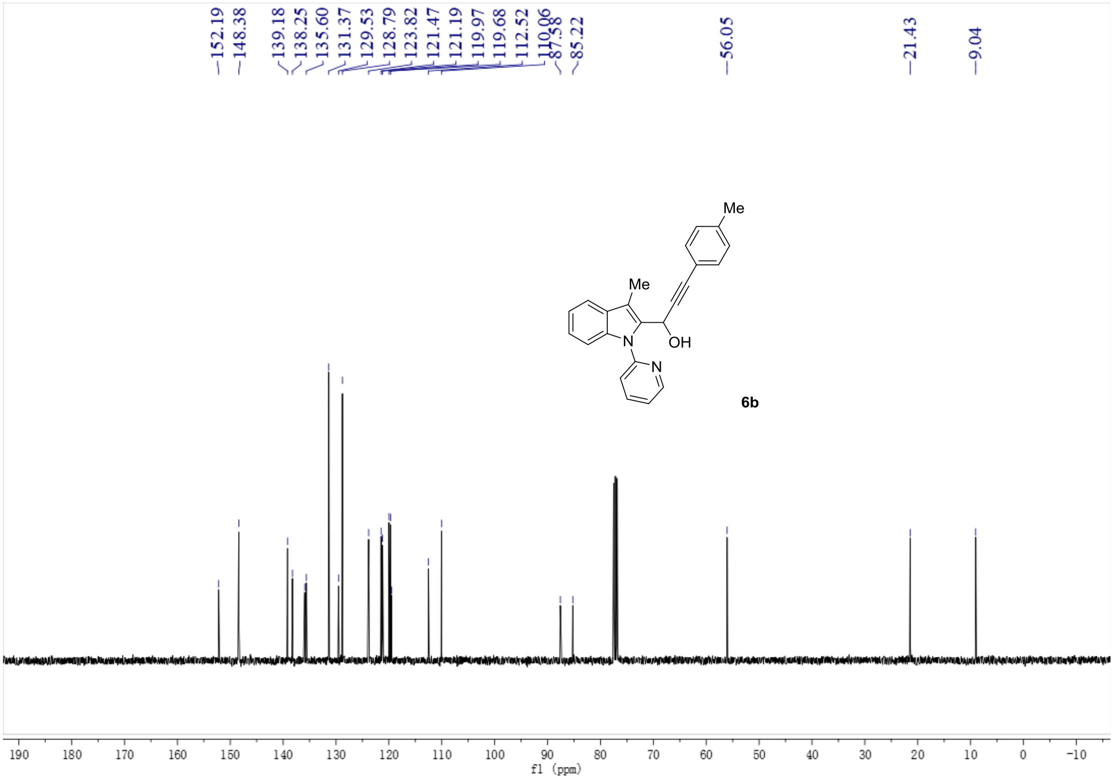

Supplementary Fig. 123 | ^{13}C NMR (101 MHz) of compound 6b (using $CDCl_3$ as solvent)

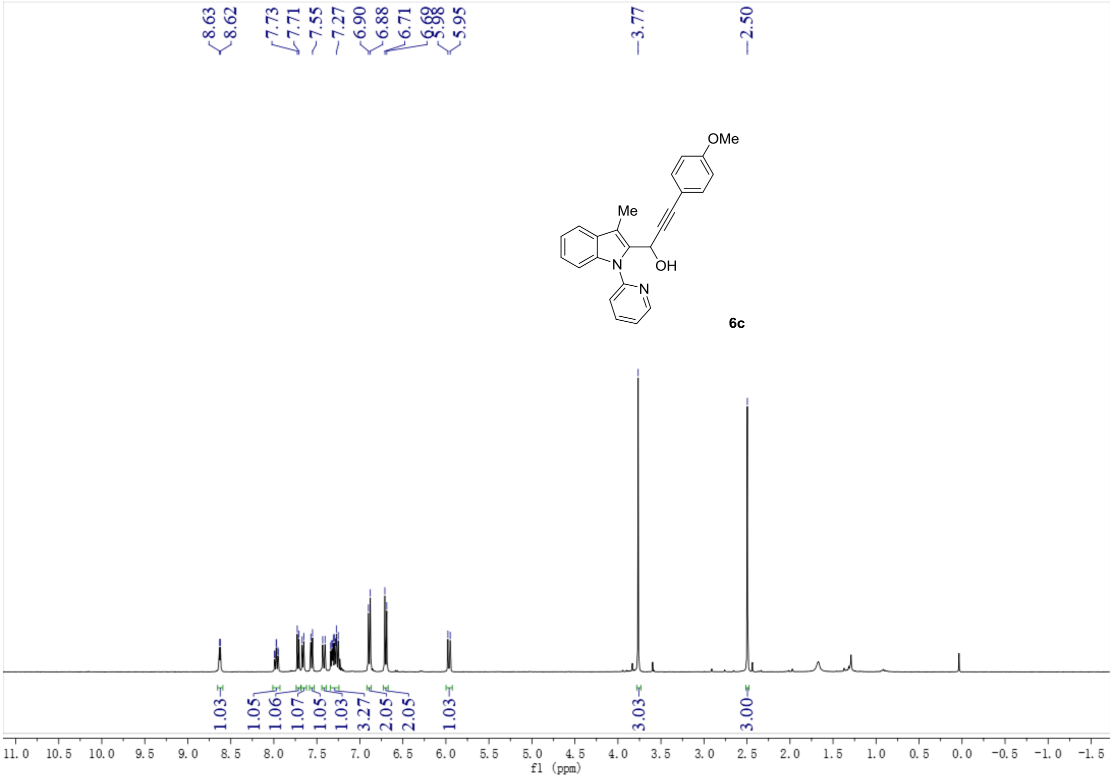

Supplementary Fig. 124 | ^{1}H NMR (400 MHz) of compound 6c (using $CDCl_3$ as solvent)

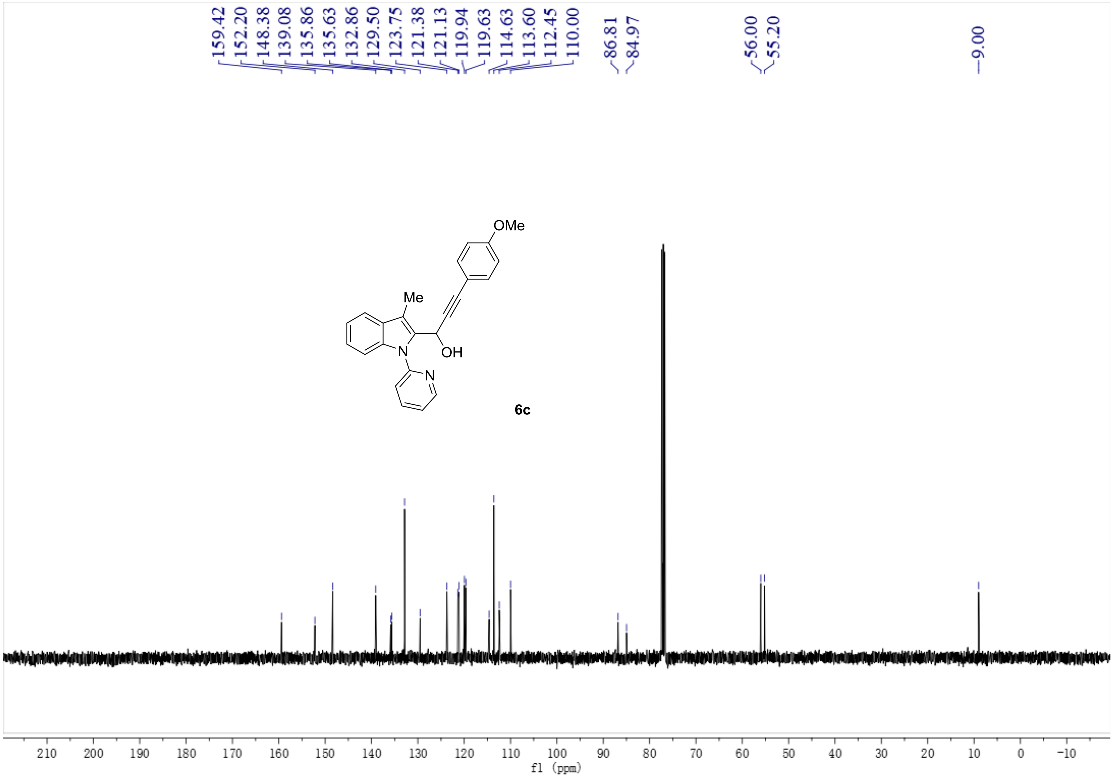

Supplementary Fig. 125 | ^{13}C NMR (101 MHz) of compound 6c (using $CDCl_3$ as solvent)

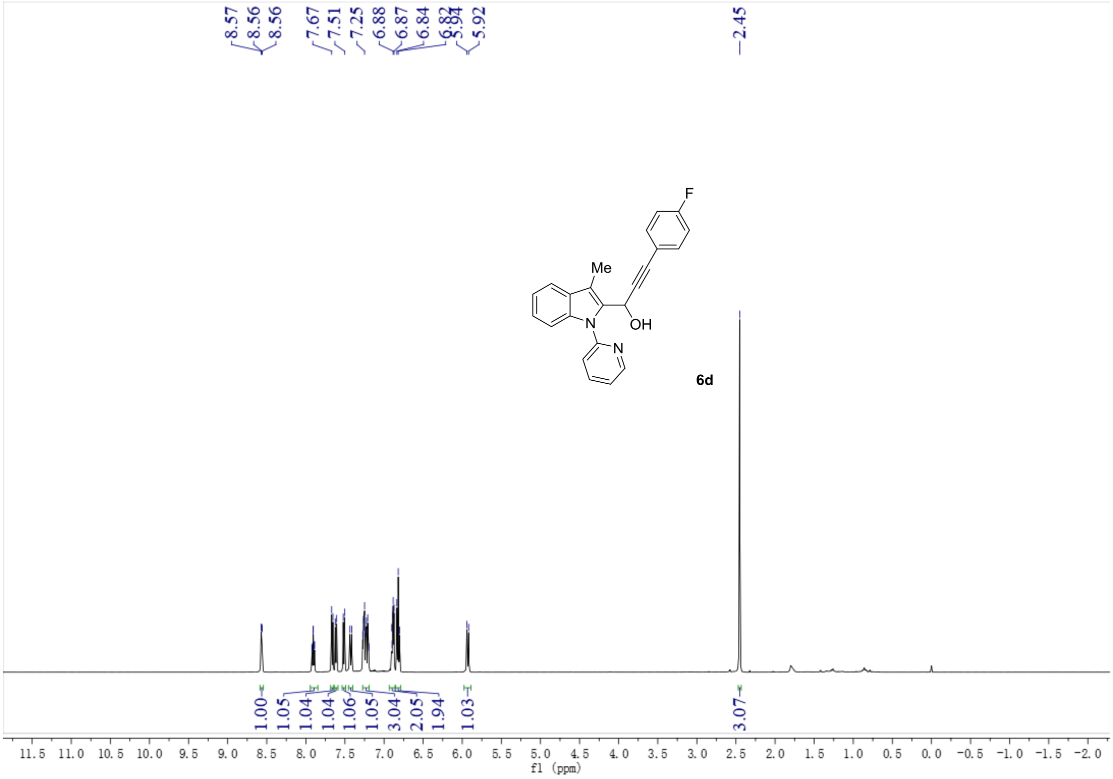

Supplementary Fig. 126 | ^{1}H NMR (500 MHz) of compound 6d (using $CDCl_3$ as solvent)

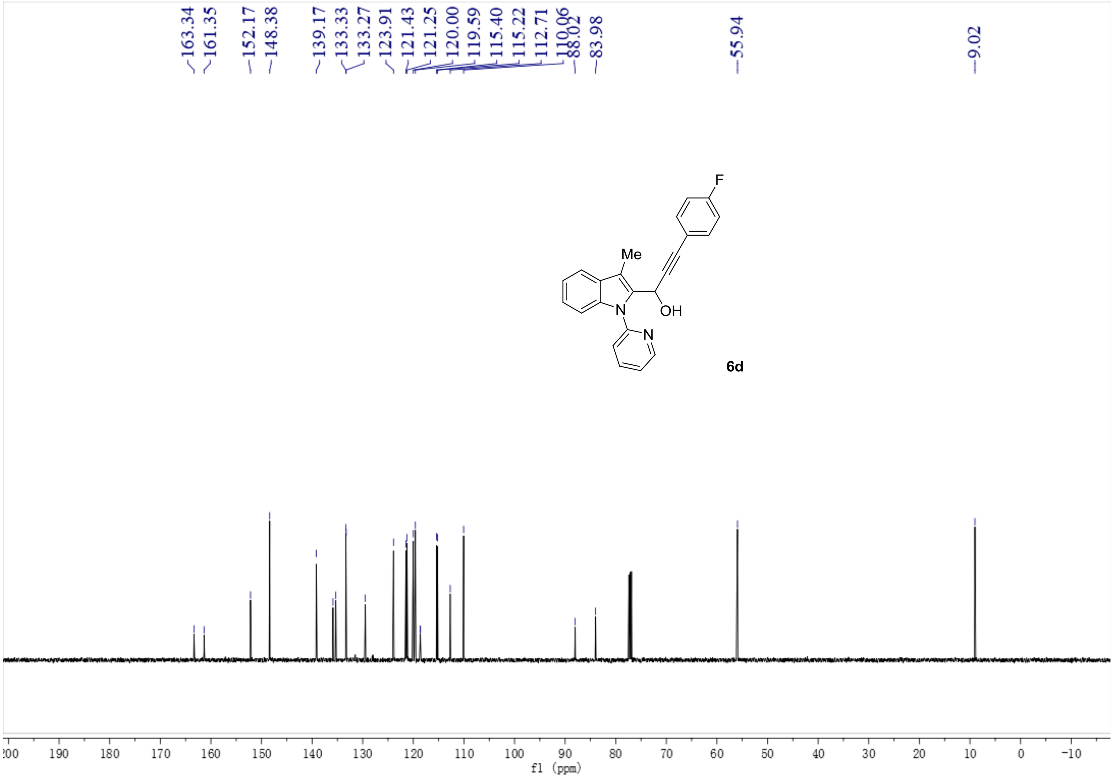

Supplementary Fig. 127 | ^{13}C NMR (126 MHz) of compound 6d (using $CDCl_3$ as solvent)

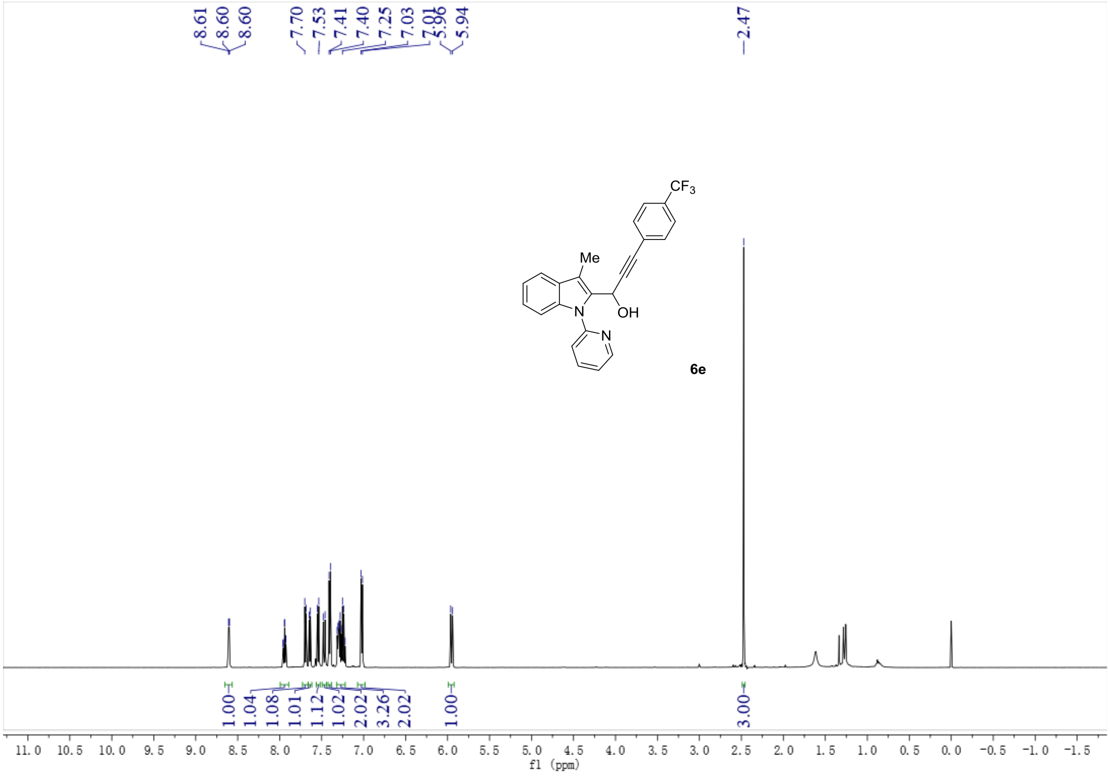

Supplementary Fig. 128 | ^{1}H NMR (500 MHz) of compound 6e (using $CDCl_3$ as solvent)

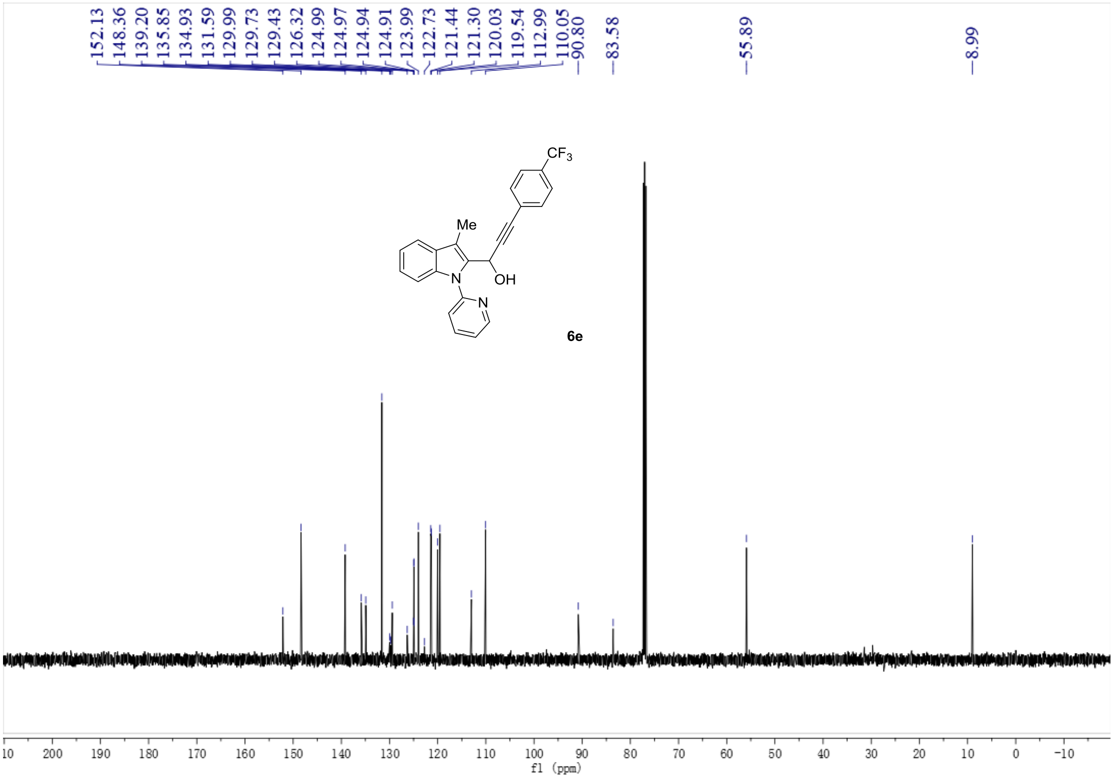

Supplementary Fig. 129 | ^{13}C NMR (126 MHz) of compound 6e (using $CDCl_3$ as solvent)

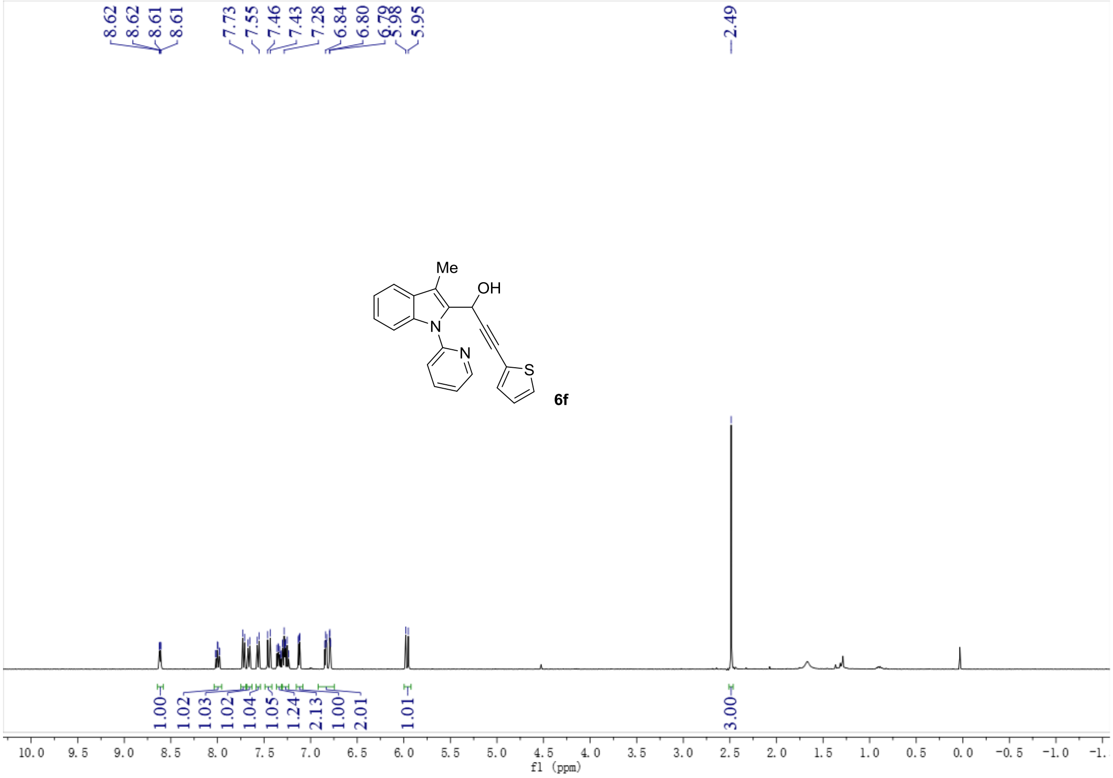

Supplementary Fig. 130 | ^{1}H NMR (400 MHz) of compound 6f (using $CDCl_3$ as solvent)

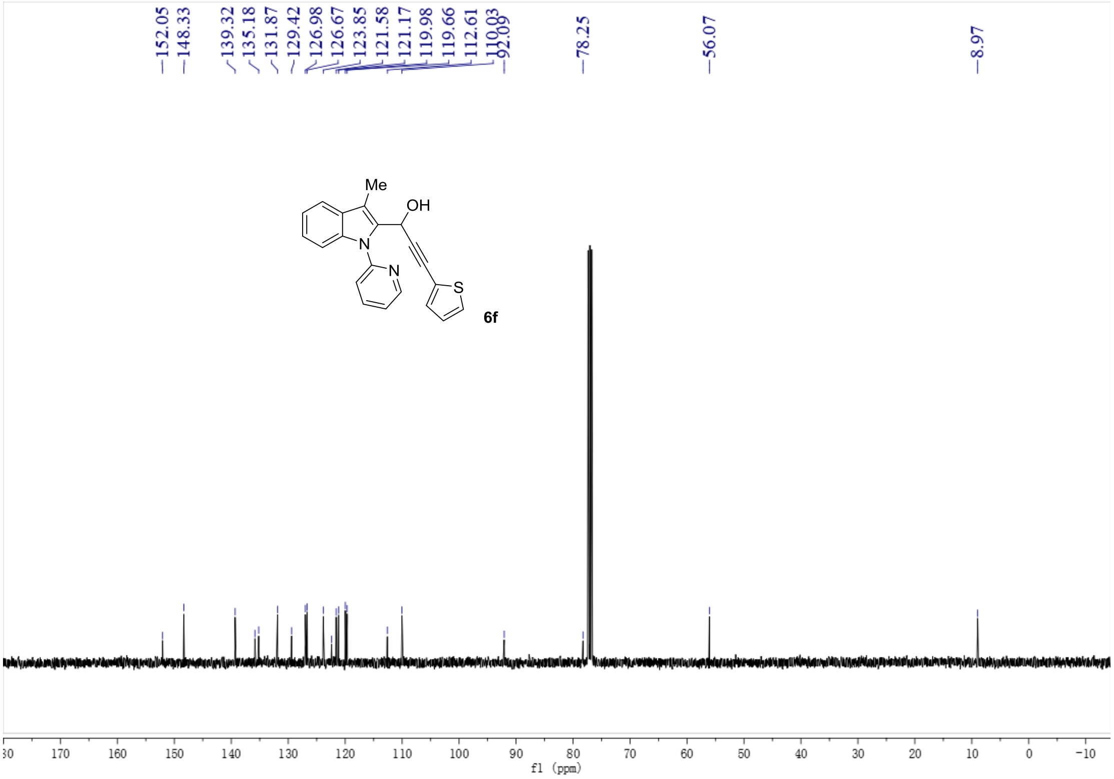

Supplementary Fig. 131 | ^{13}C NMR (101 MHz) of compound 6f (using $CDCl_3$ as solvent)

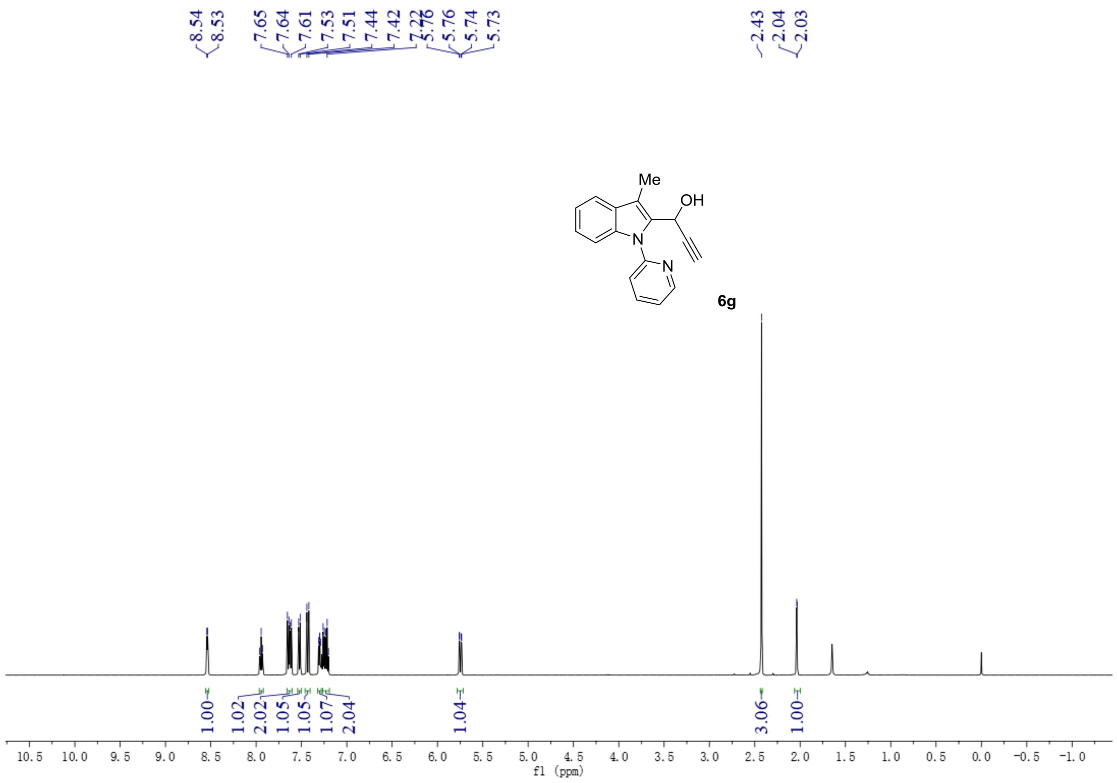

Supplementary Fig. 132 | ^{1}H NMR (500 MHz) of compound 6g (using $CDCl_3$ as solvent)

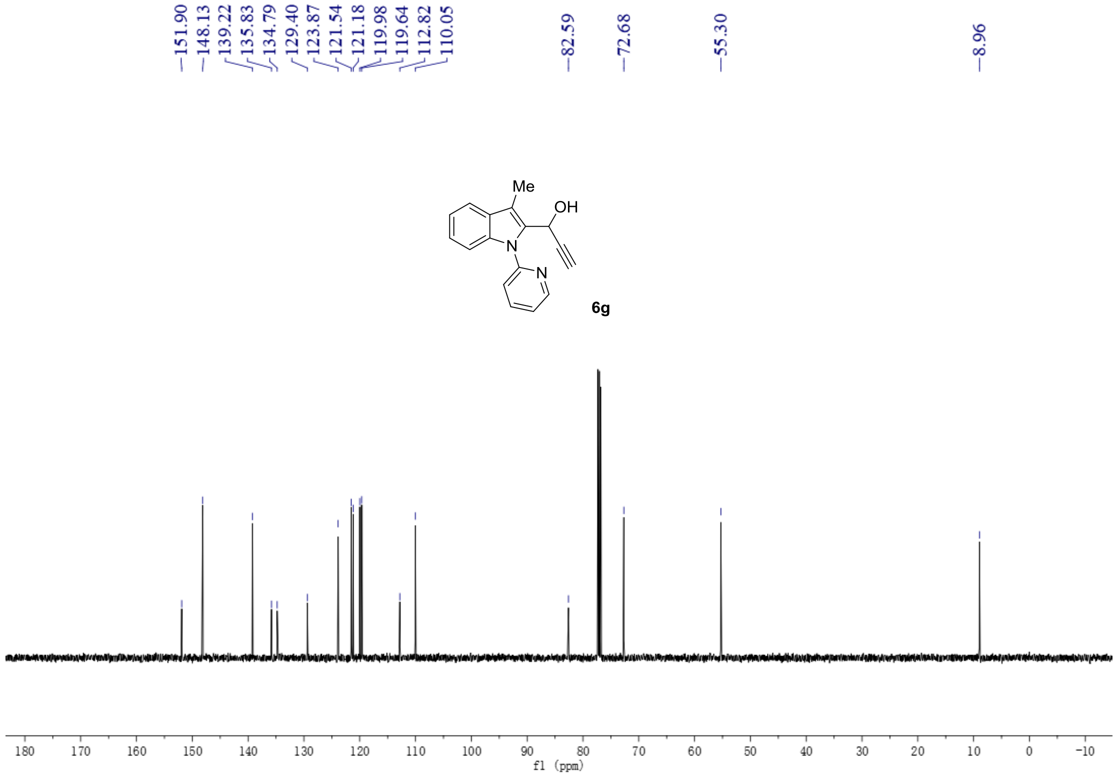

Supplementary Fig. 133 | ^{13}C NMR (126 MHz) of compound 6g (using $CDCl_3$ as solvent)

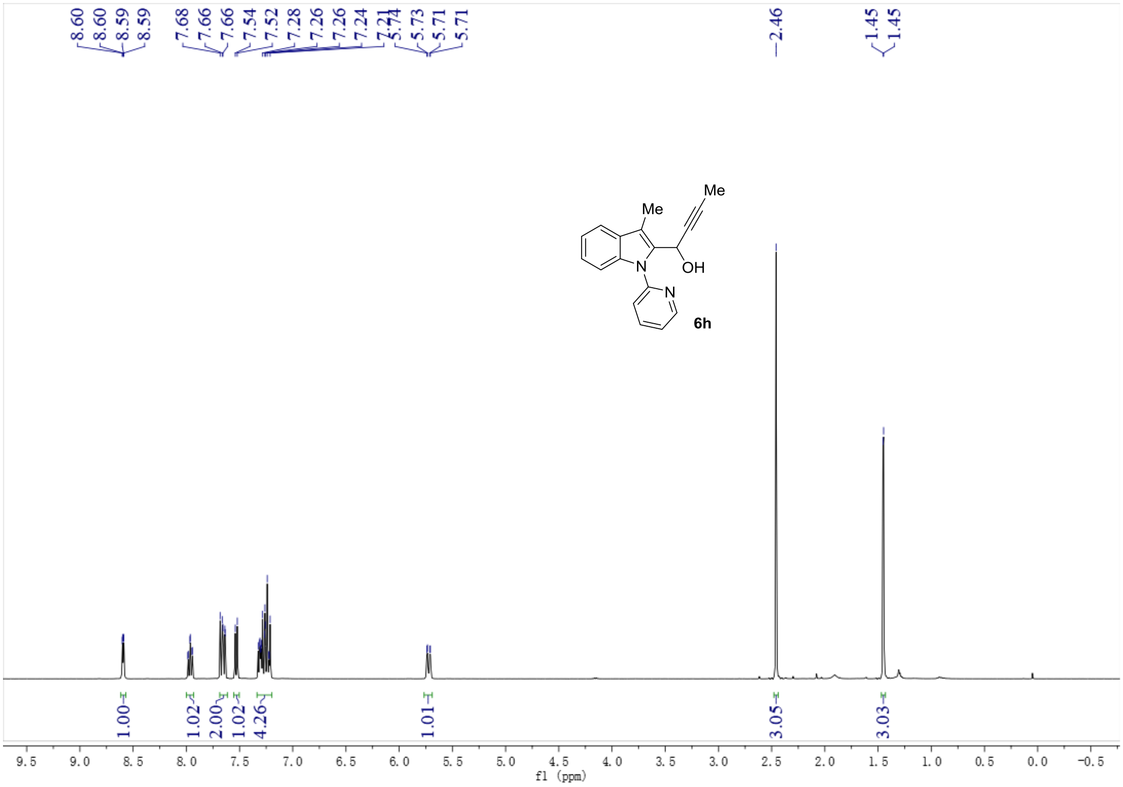

Supplementary Fig. 134 | ^{1}H NMR (400 MHz) of compound 6h (using $CDCl_3$ as solvent)

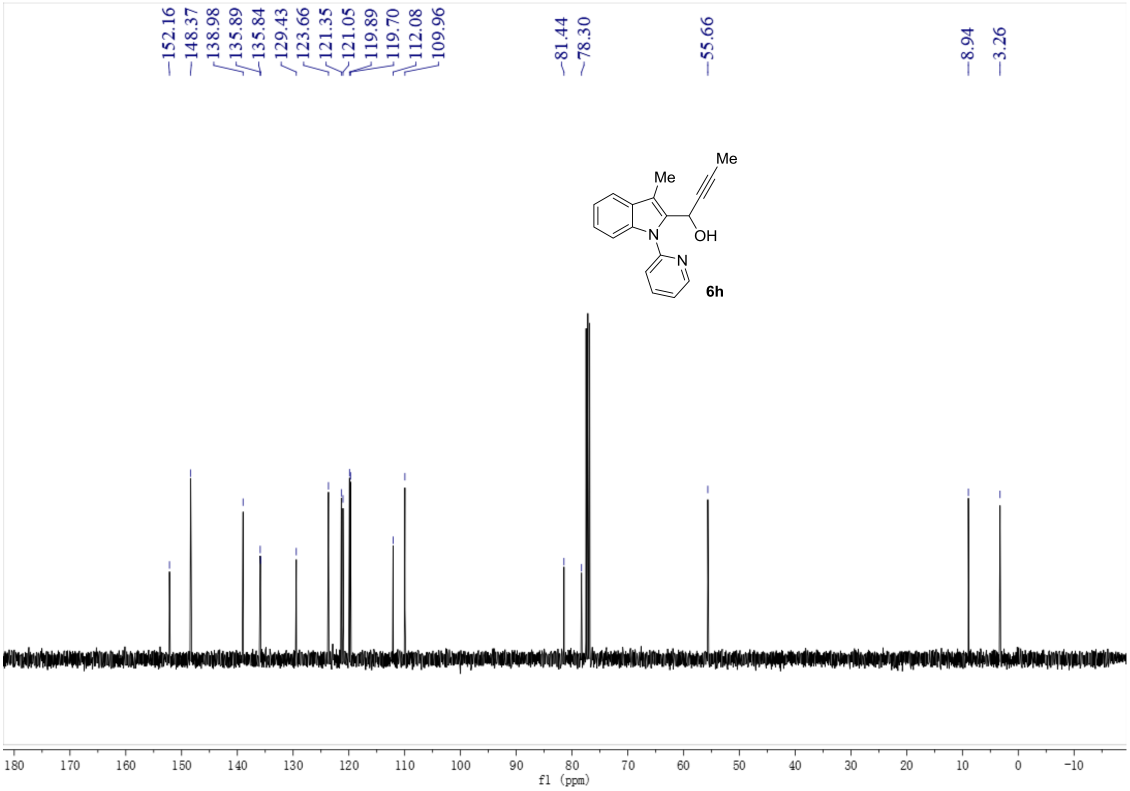

Supplementary Fig. 135 | ^{13}C NMR (101 MHz) of compound 6h (using $CDCl_3$ as solvent)

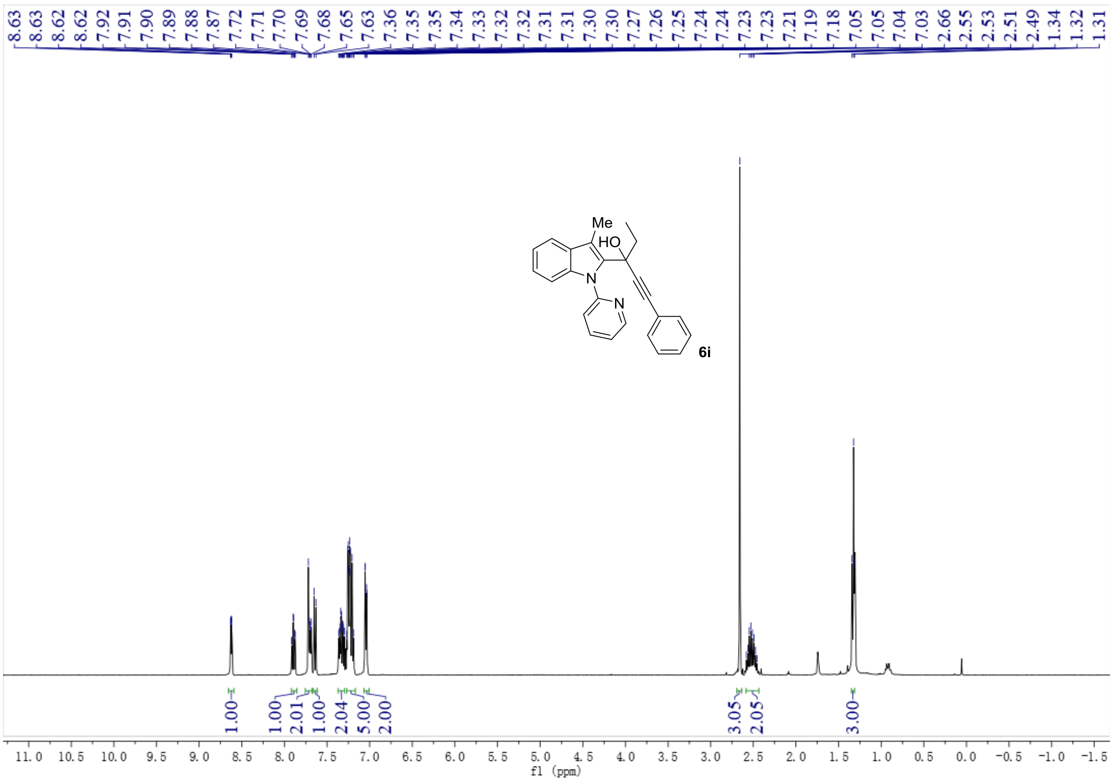

Supplementary Fig. 136 | ^{1}H NMR (400 MHz) of compound 6i (using $CDCl_3$ as solvent)

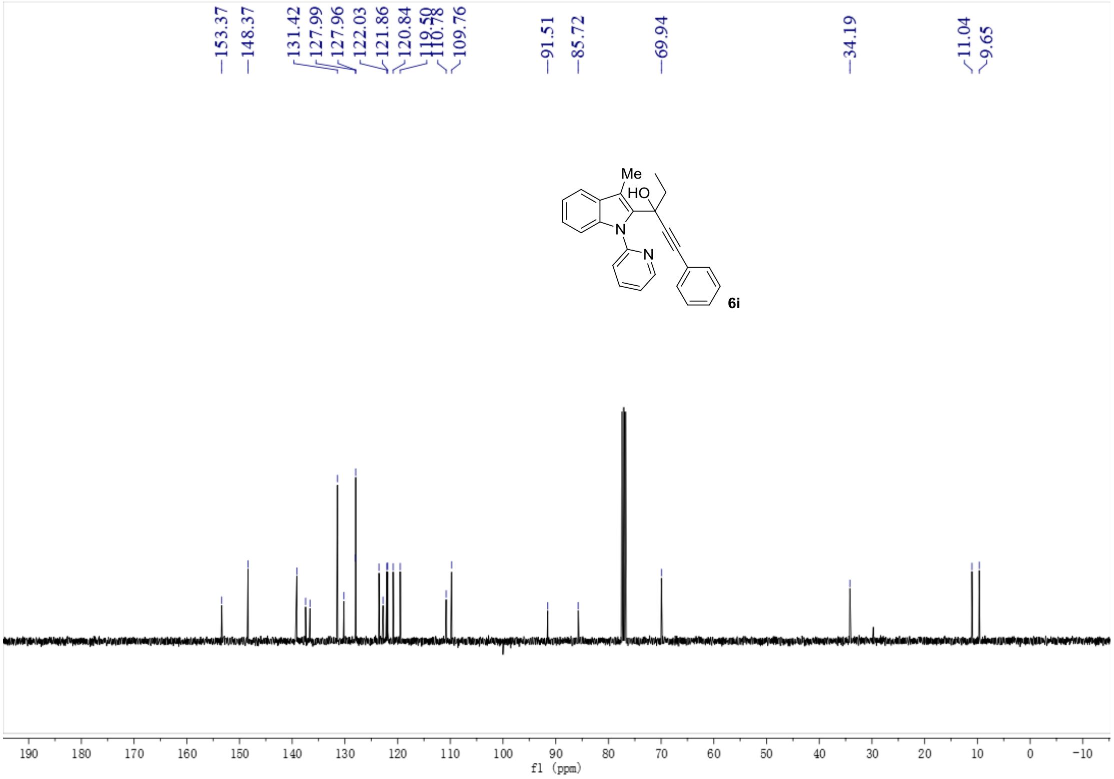

Supplementary Fig. 137 | ^{13}C NMR (101 MHz) of compound 6i (using $CDCl_3$ as solvent)

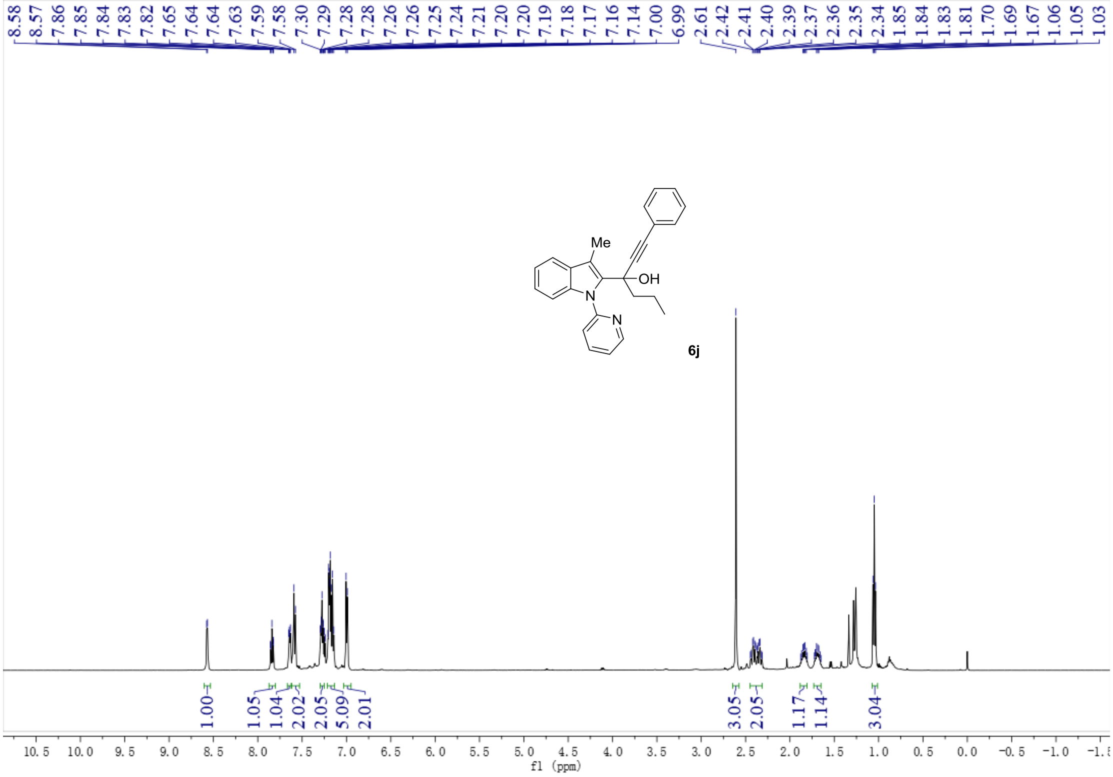

Supplementary Fig. 138 | ^{1}H NMR (500 MHz) of compound 6j (using $CDCl_3$ as solvent)

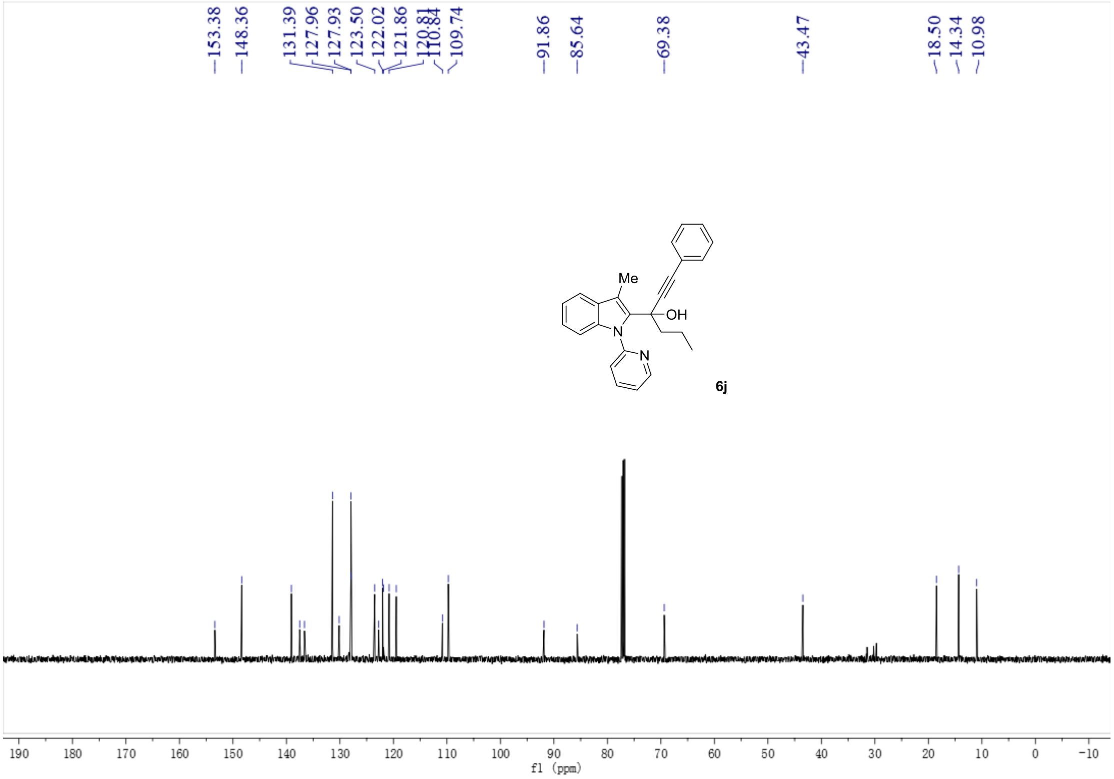

Supplementary Fig. 139 | ^{13}C NMR (126 MHz) of compound 6j (using $CDCl_3$ as solvent)

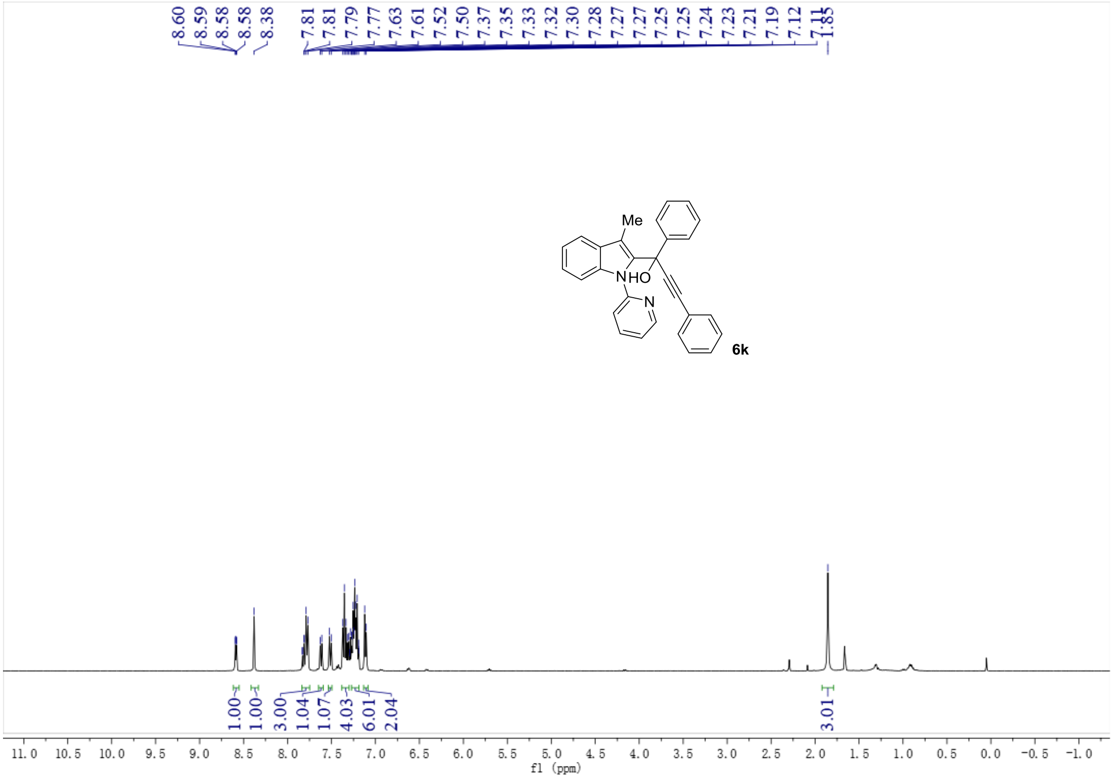

Supplementary Fig. 140 | ^{1}H NMR (500 MHz) of compound 6k (using $CDCl_3$ as solvent)

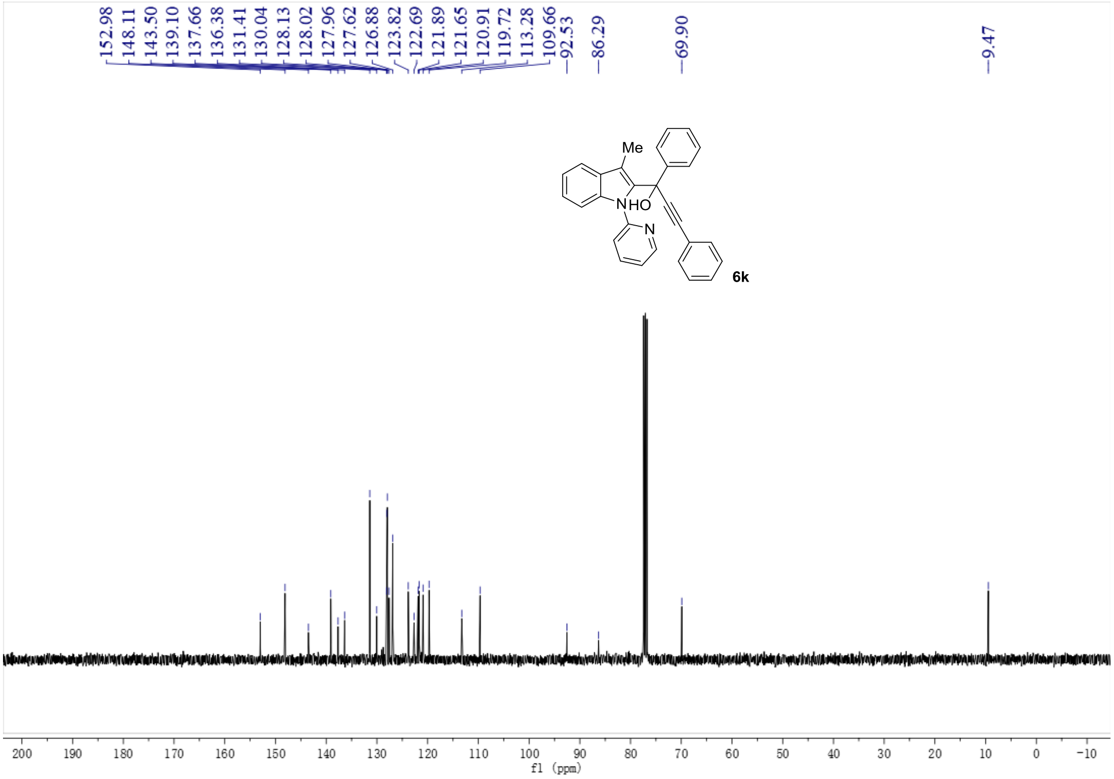

Supplementary Fig. 141 | ^{13}C NMR (126 MHz) of compound 6k (using $CDCl_3$ as solvent)

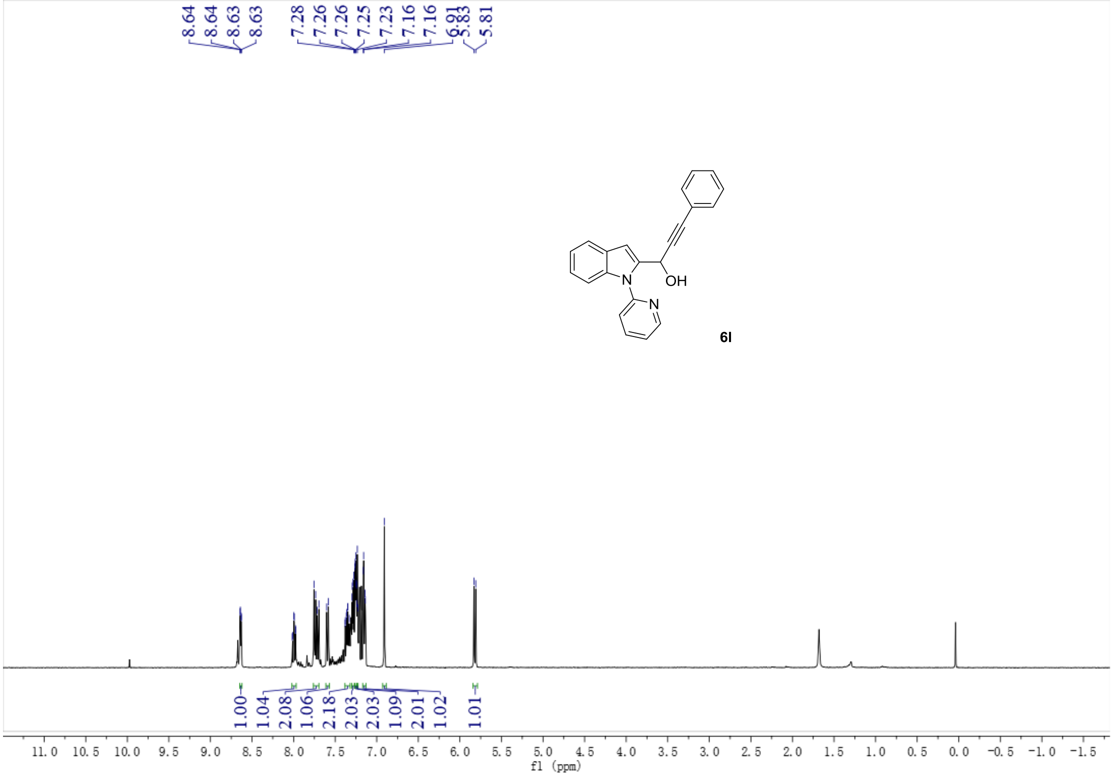

Supplementary Fig. 142 | ^{1}H NMR (400 MHz) of compound 6l (using $CDCl_3$ as solvent)

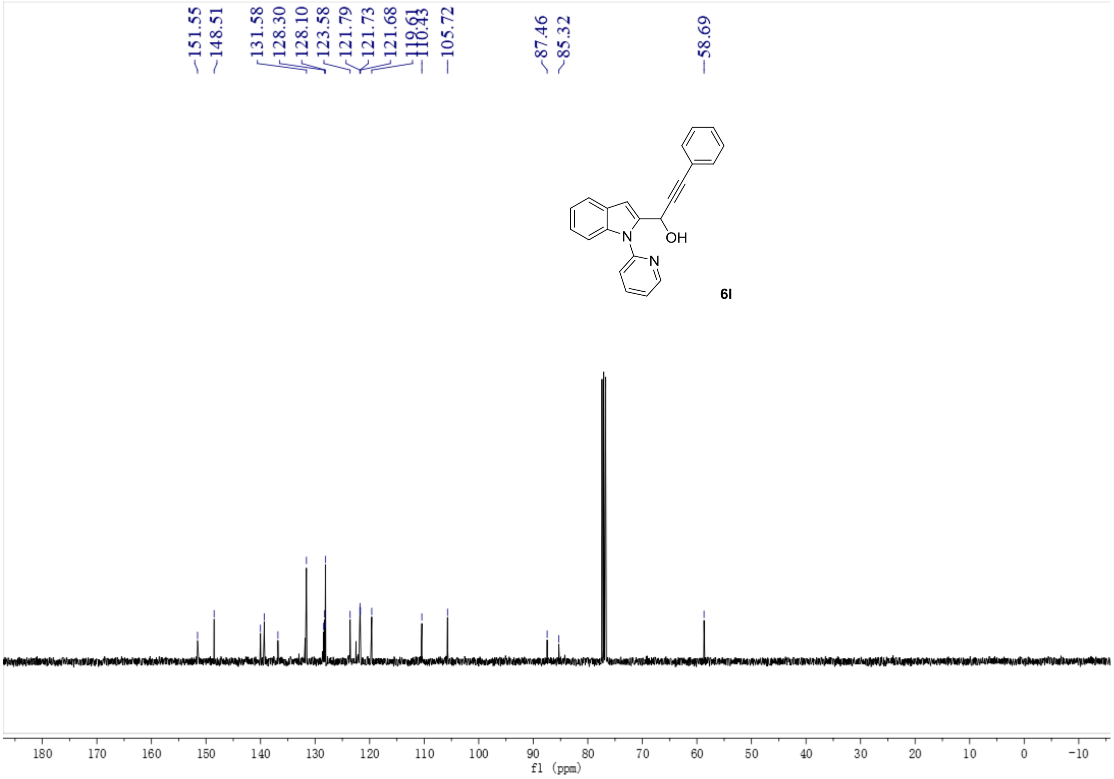

Supplementary Fig. 143 | ^{13}C NMR (101 MHz) of compound 6l (using $CDCl_3$ as solvent)

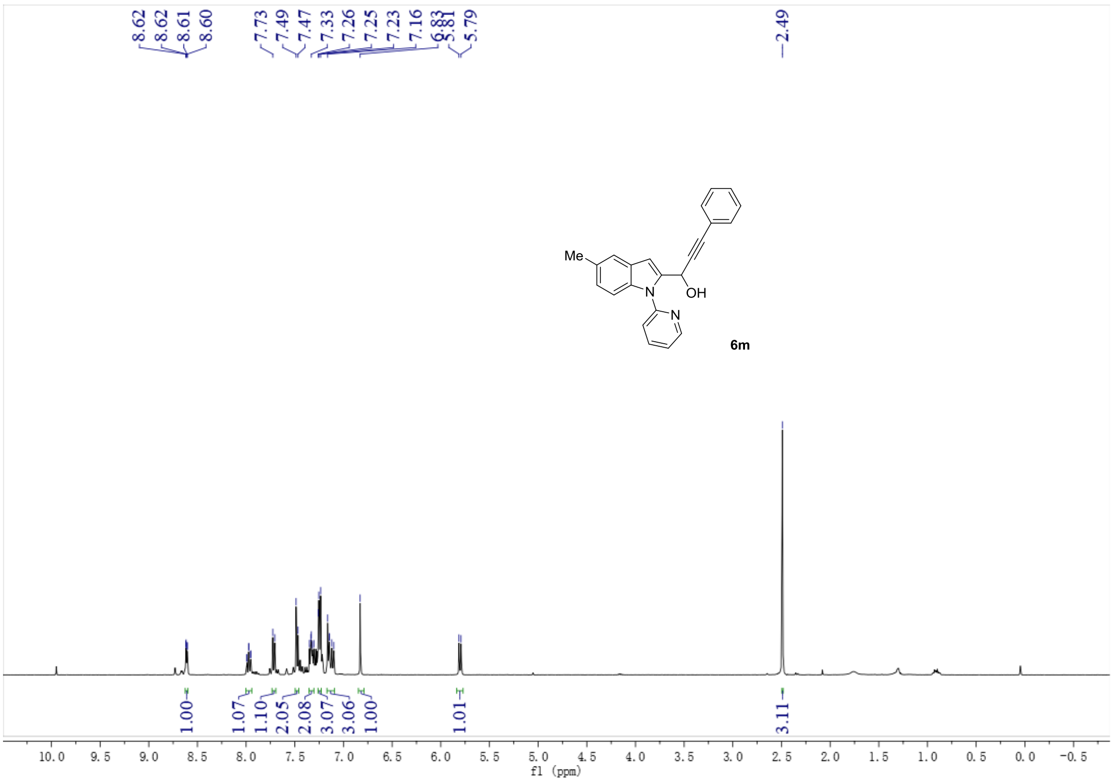

Supplementary Fig. 144 | ^{1}H NMR (400 MHz) of compound 6m (using $CDCl_3$ as solvent)

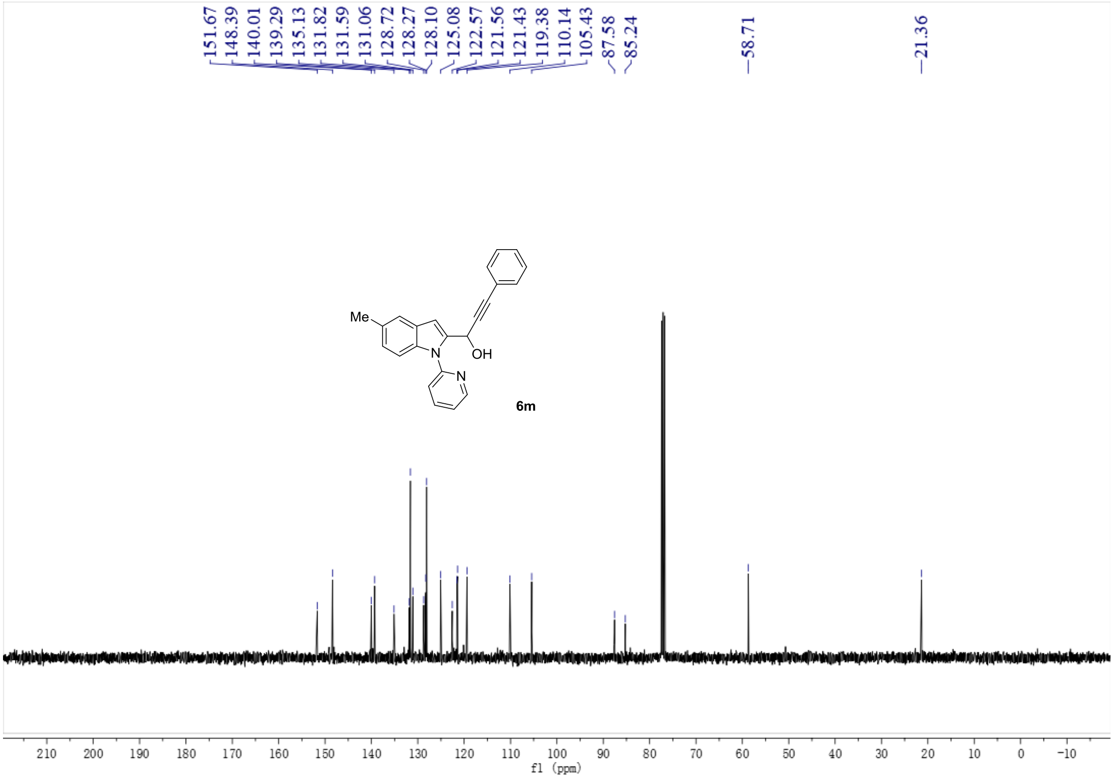

Supplementary Fig. 145 | ^{13}C NMR (101 MHz) of compound 6m (using $CDCl_3$ as solvent)

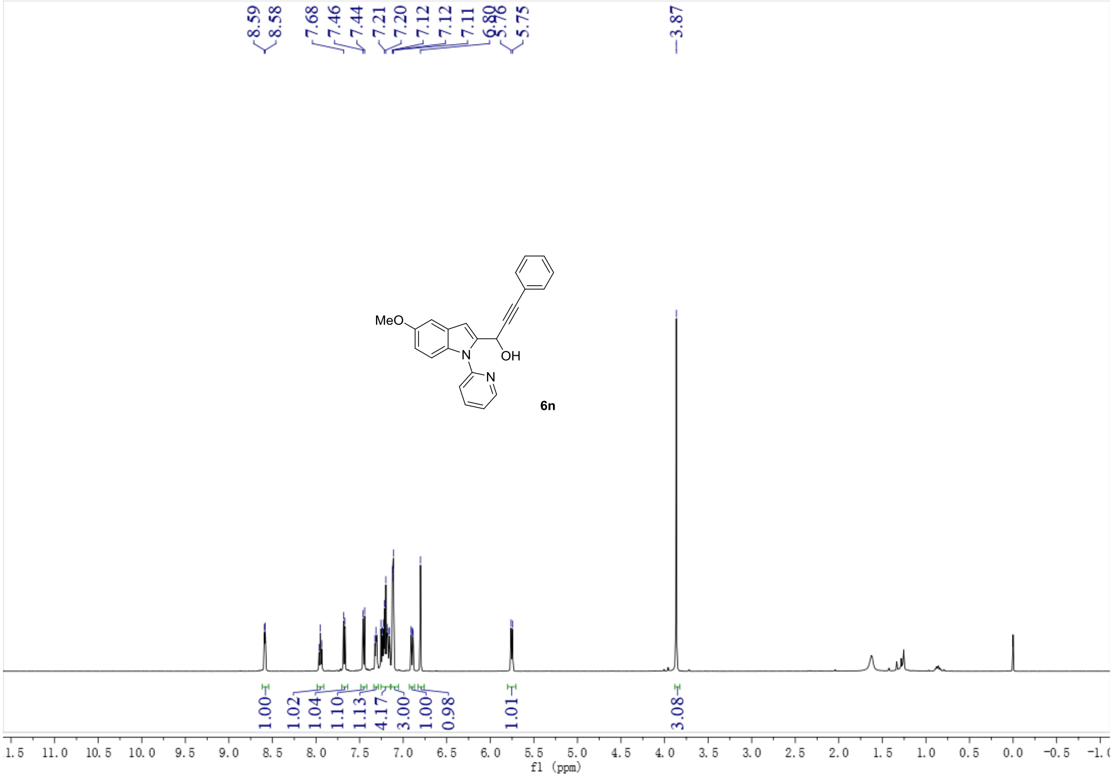

Supplementary Fig. 146 | ^{1}H NMR (500 MHz) of compound 6n (using $CDCl_3$ as solvent)

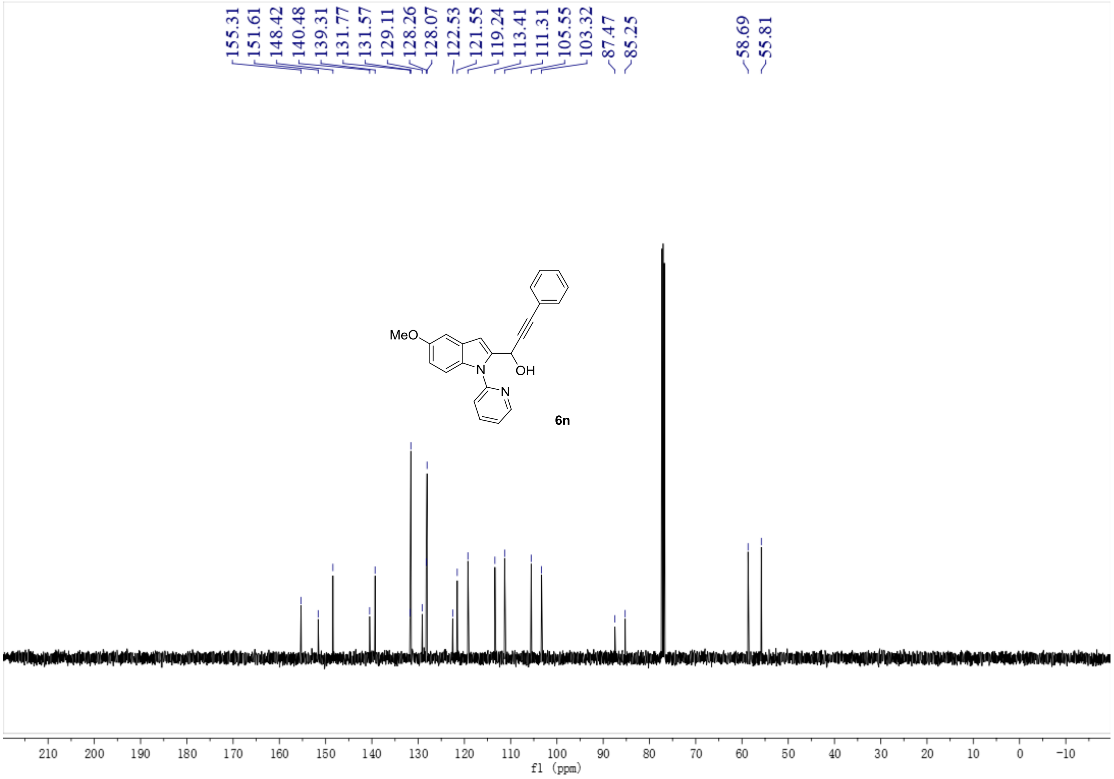

Supplementary Fig. 147 | ^{13}C NMR (126 MHz) of compound 6n (using $CDCl_3$ as solvent)

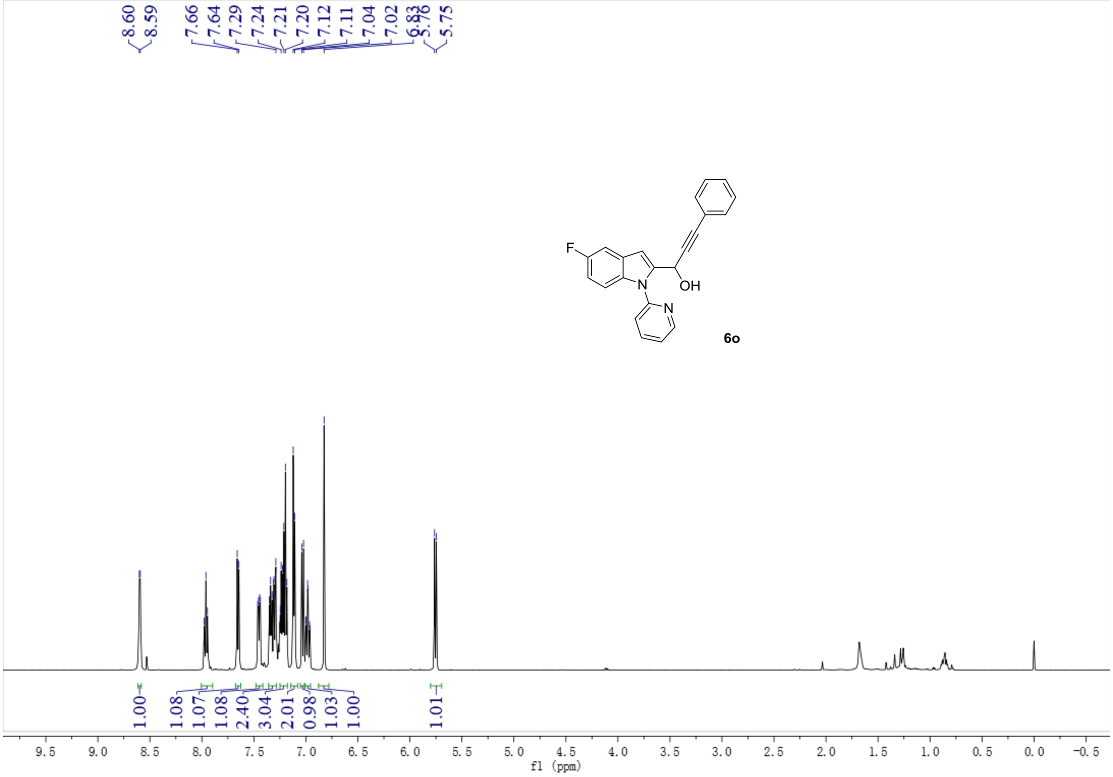

Supplementary Fig. 148 | ^{1}H NMR (500 MHz) of compound 6o (using $CDCl_3$ as solvent)

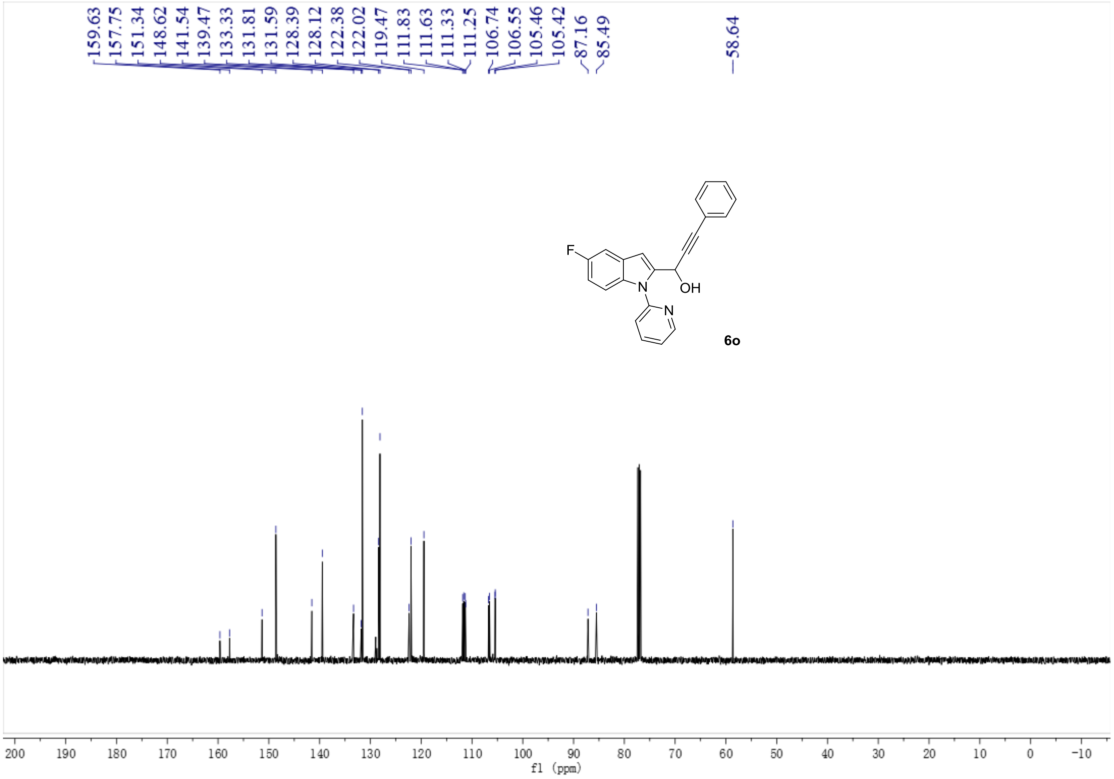

Supplementary Fig. 149 | ^{13}C NMR (126 MHz) of compound 6o (using $CDCl_3$ as solvent)

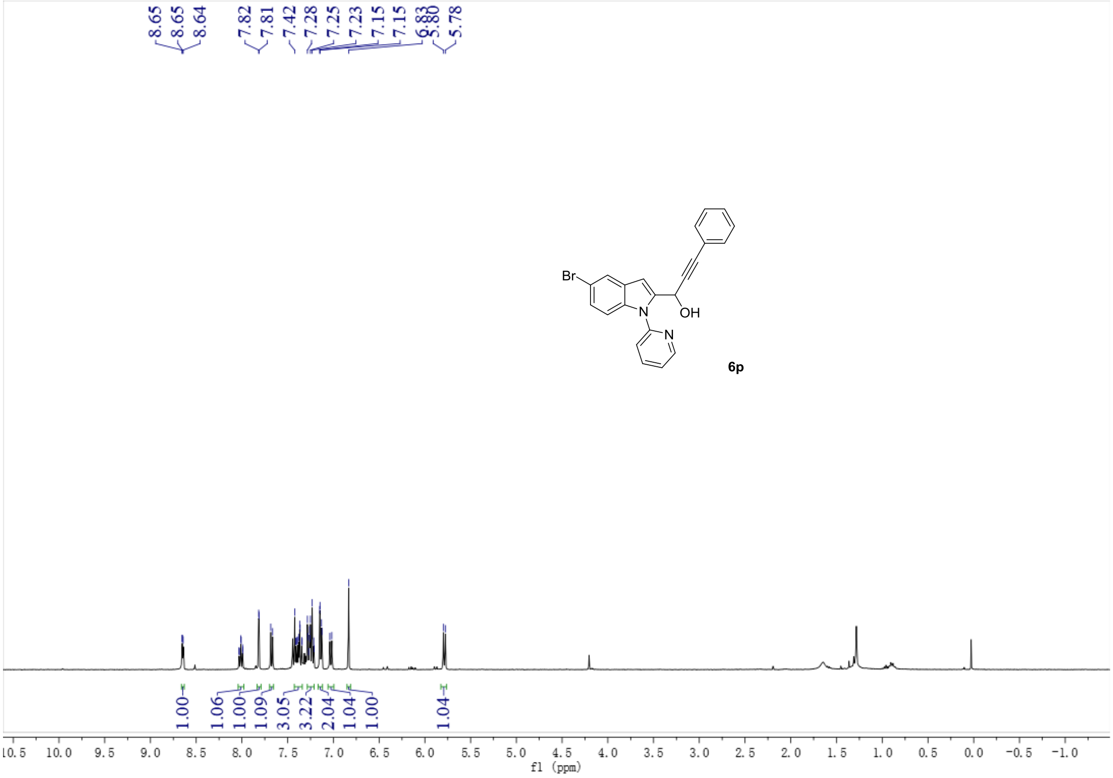

Supplementary Fig. 150 | ^{1}H NMR (400 MHz) of compound 6p (using $CDCl_3$ as solvent)

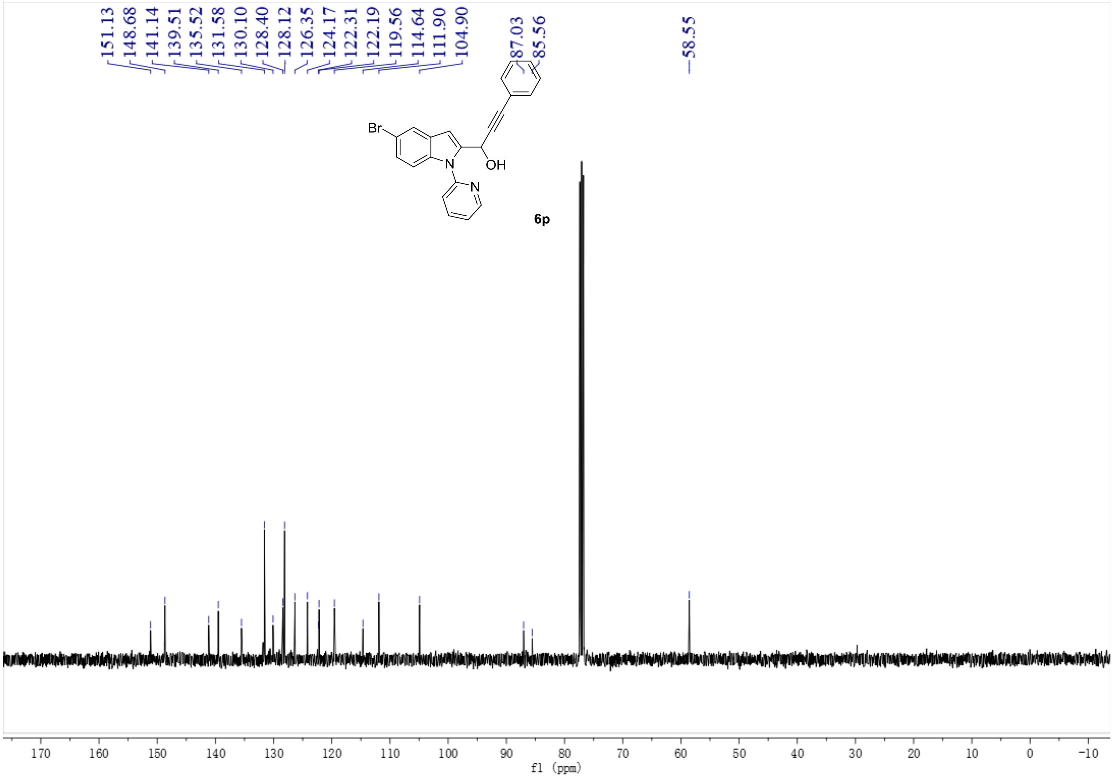

Supplementary Fig. 151 | ^{13}C NMR (101 MHz) of compound 6p (using $CDCl_3$ as solvent)

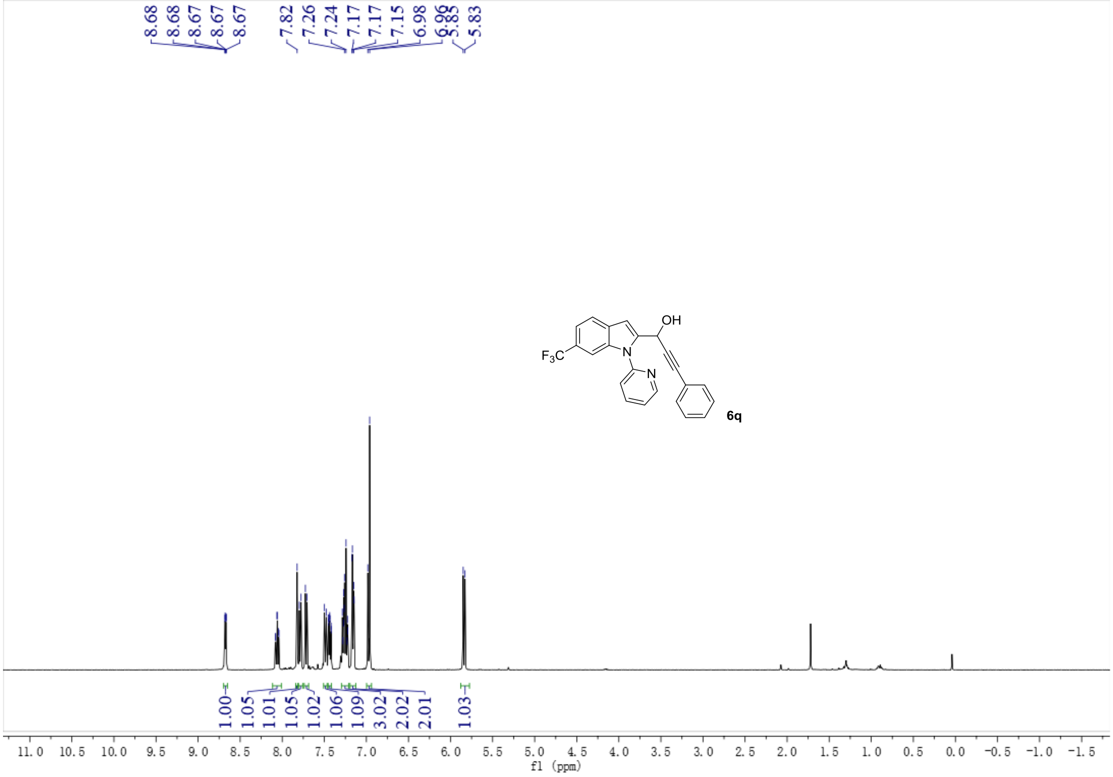

Supplementary Fig. 152 | ^{1}H NMR (400 MHz) of compound 6q (using $CDCl_3$ as solvent)

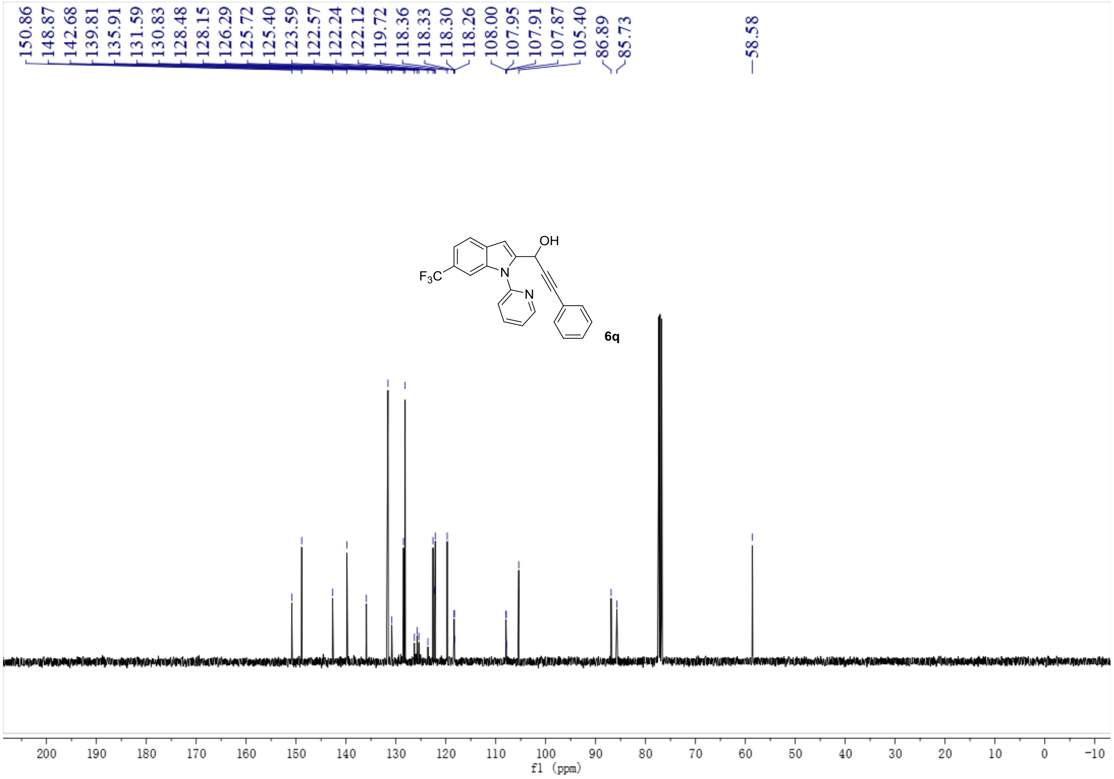

Supplementary Fig. 153 | ^{13}C NMR (101 MHz) of compound 6q (using $CDCl_3$ as solvent)

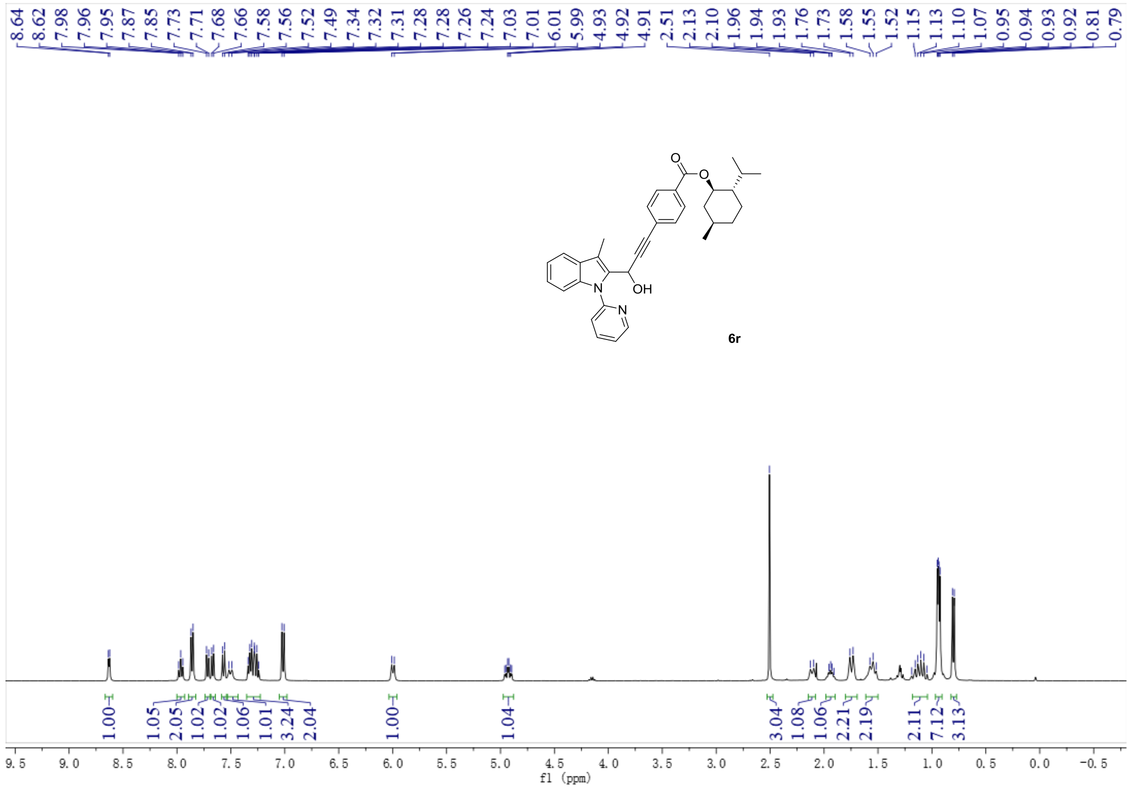

Supplementary Fig. 154 | ^{1}H NMR (400 MHz) of compound 6r (using $CDCl_3$ as solvent)

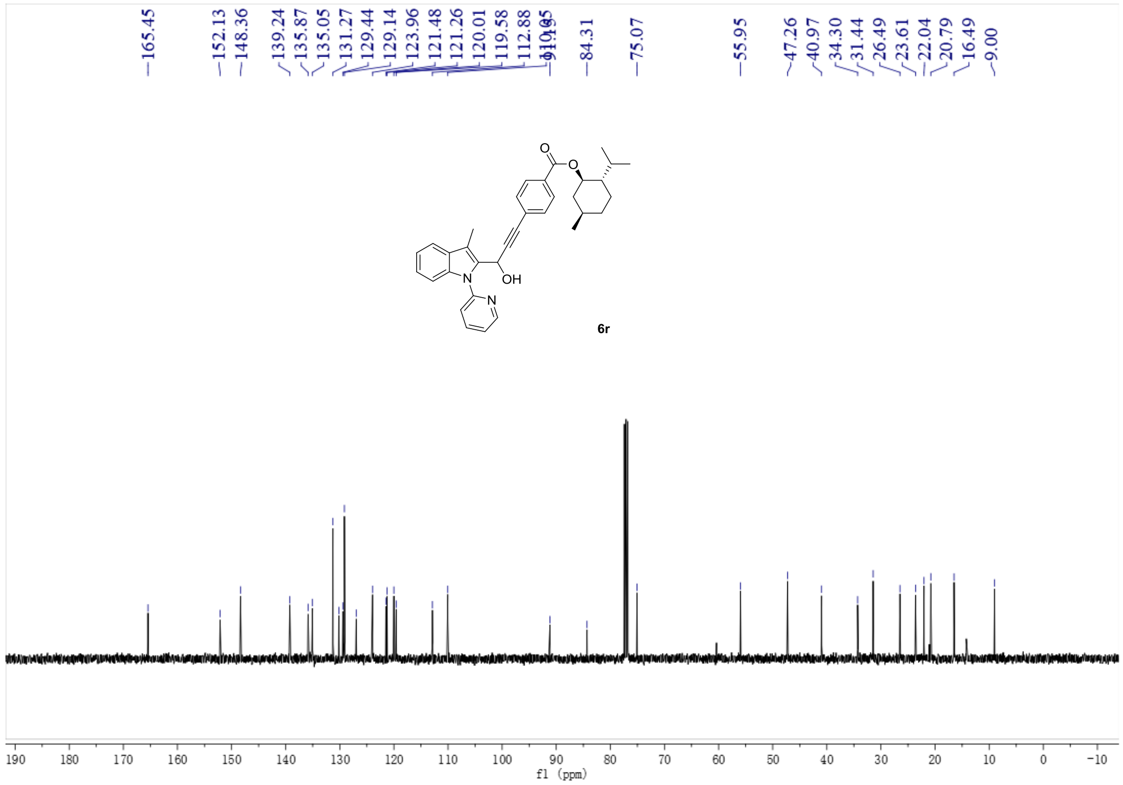

Supplementary Fig. 155 | ^{13}C NMR (101 MHz) of compound 6r (using $CDCl_3$ as solvent)

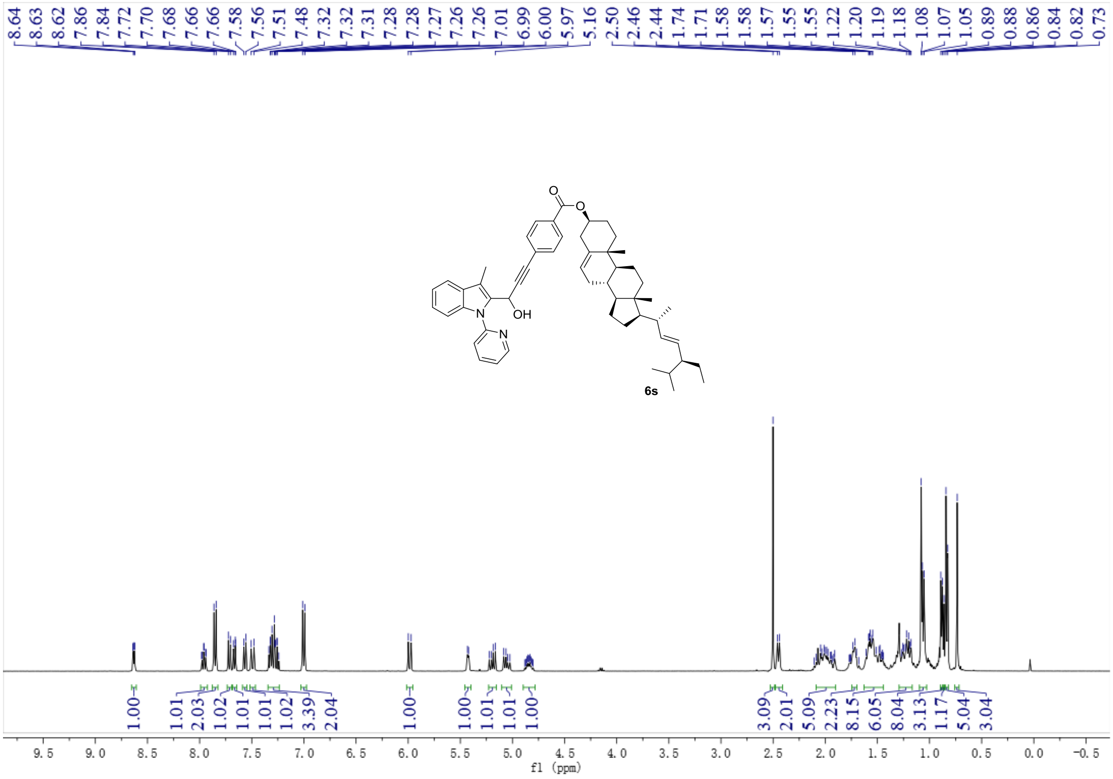

Supplementary Fig. 156 | ^{1}H NMR (400 MHz) of compound 6s (using $CDCl_3$ as solvent)

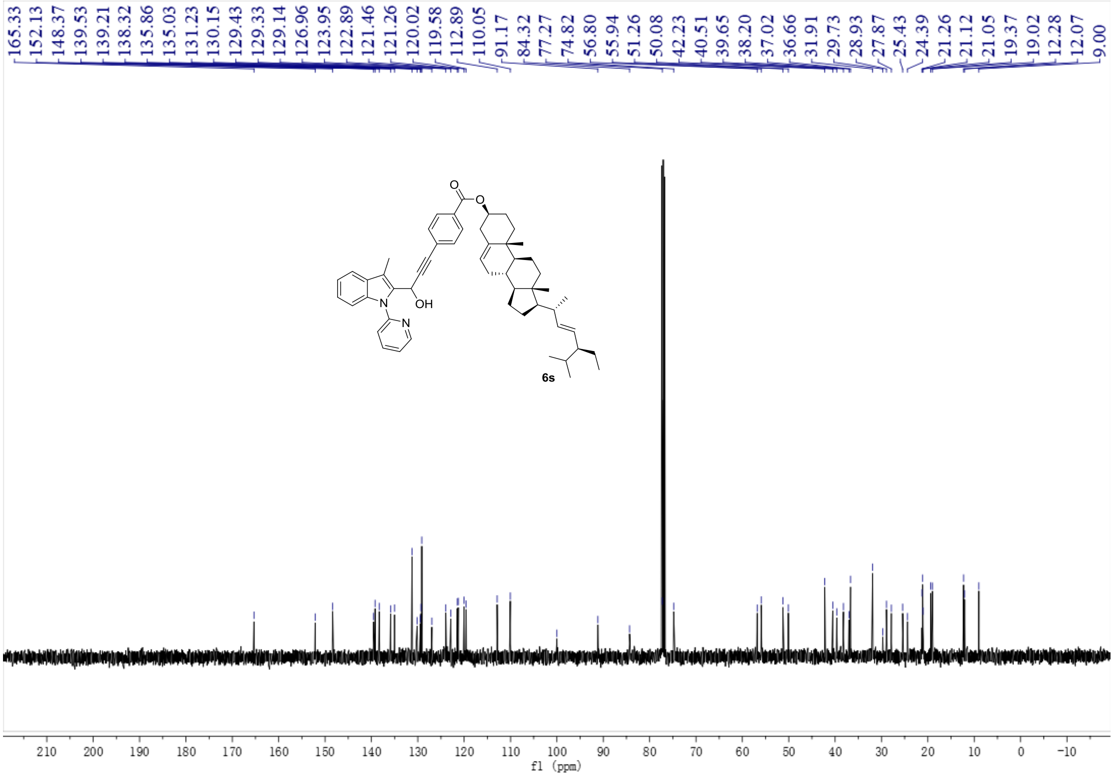

Supplementary Fig. 157 | ^{13}C NMR (101 MHz) of compound 6s (using $CDCl_3$ as solvent)

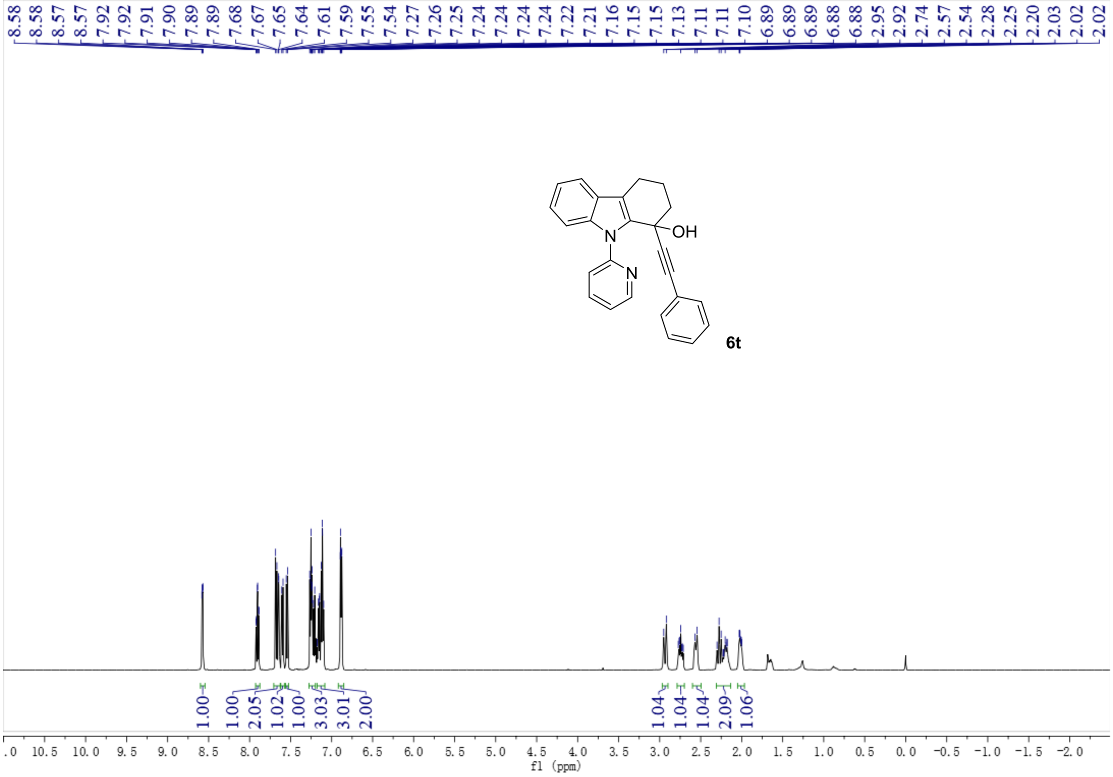

Supplementary Fig. 158 | ^{1}H NMR (500 MHz) of compound 6t (using $CDCl_3$ as solvent)

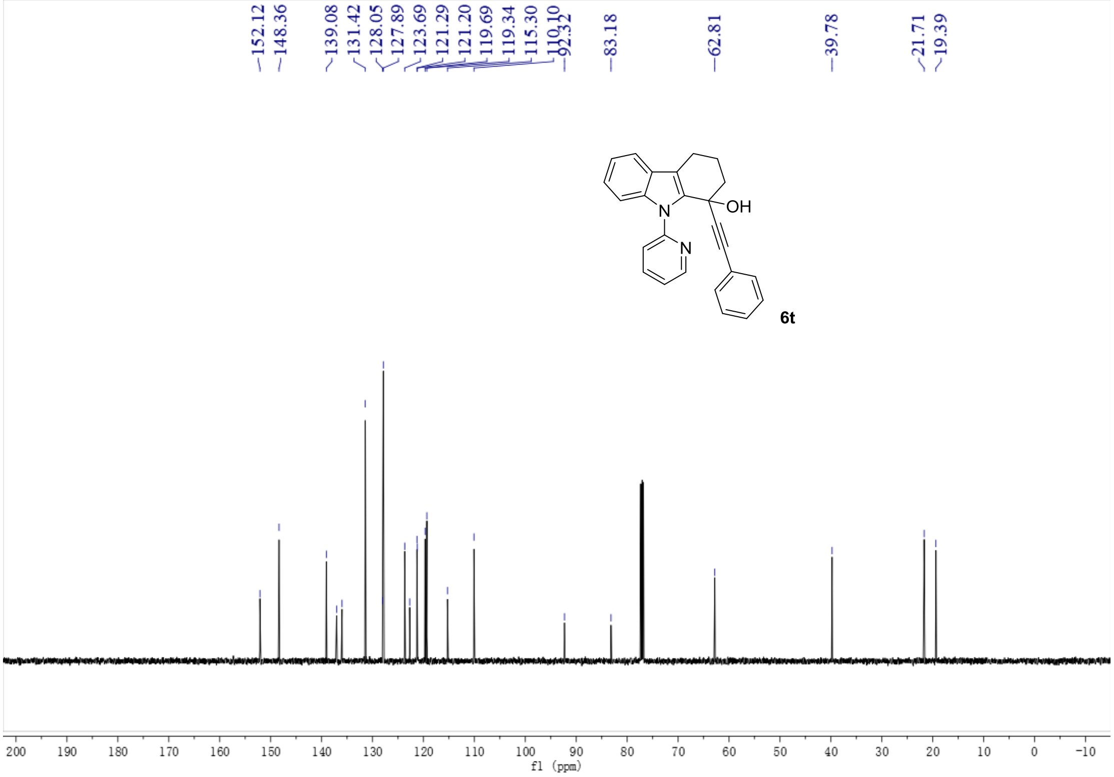

Supplementary Fig. 159 | ^{13}C NMR (126 MHz) of compound 6t (using $CDCl_3$ as solvent)

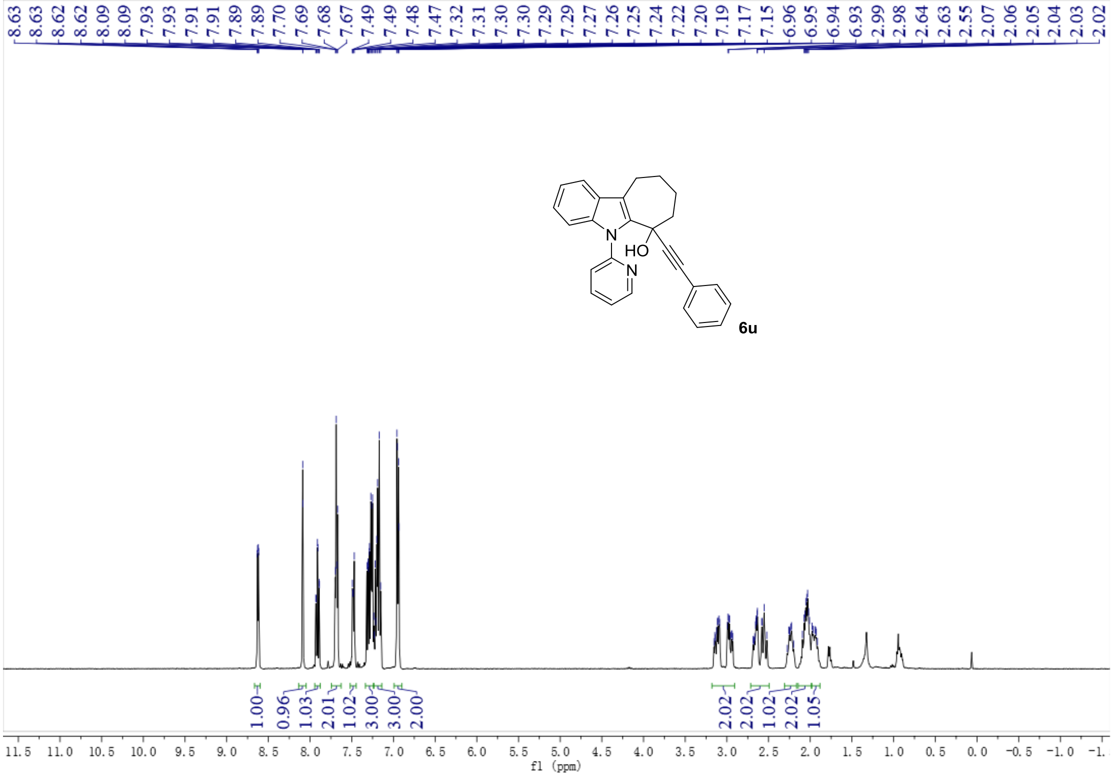

Supplementary Fig. 160 | ^{1}H NMR (400 MHz) of compound 6u (using $CDCl_3$ as solvent)

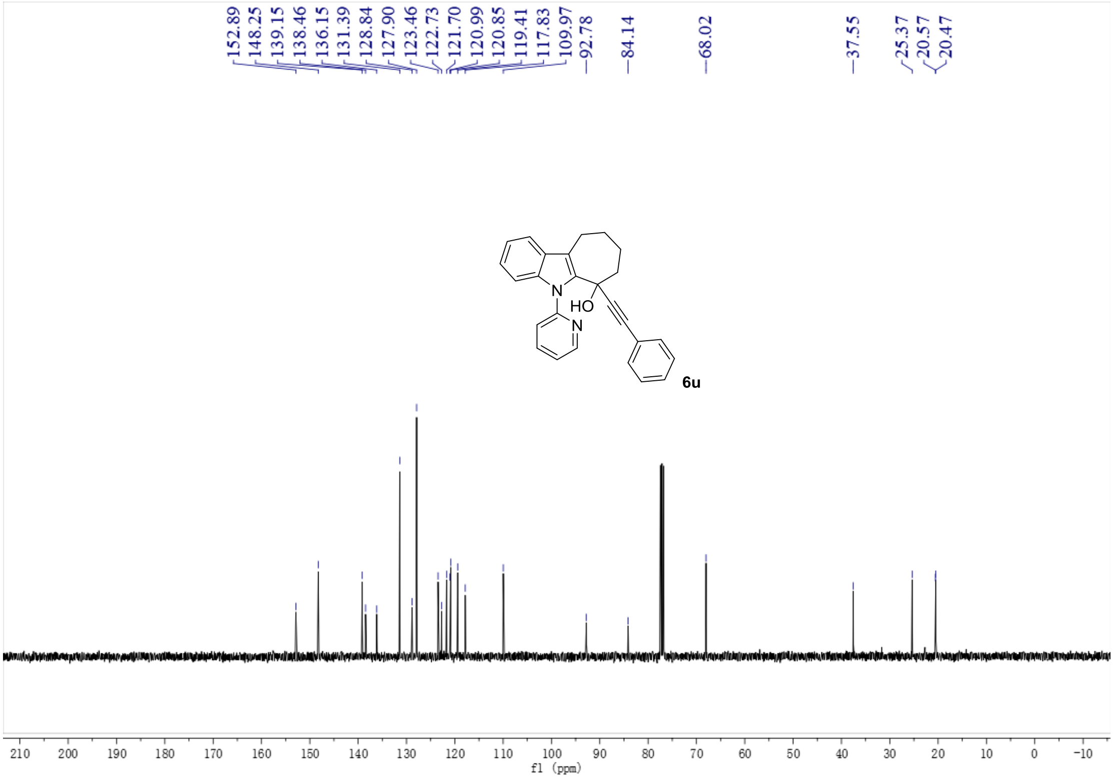

Supplementary Fig. 161 | ^{13}C NMR (101 MHz) of compound 6u (using $CDCl_3$ as solvent)

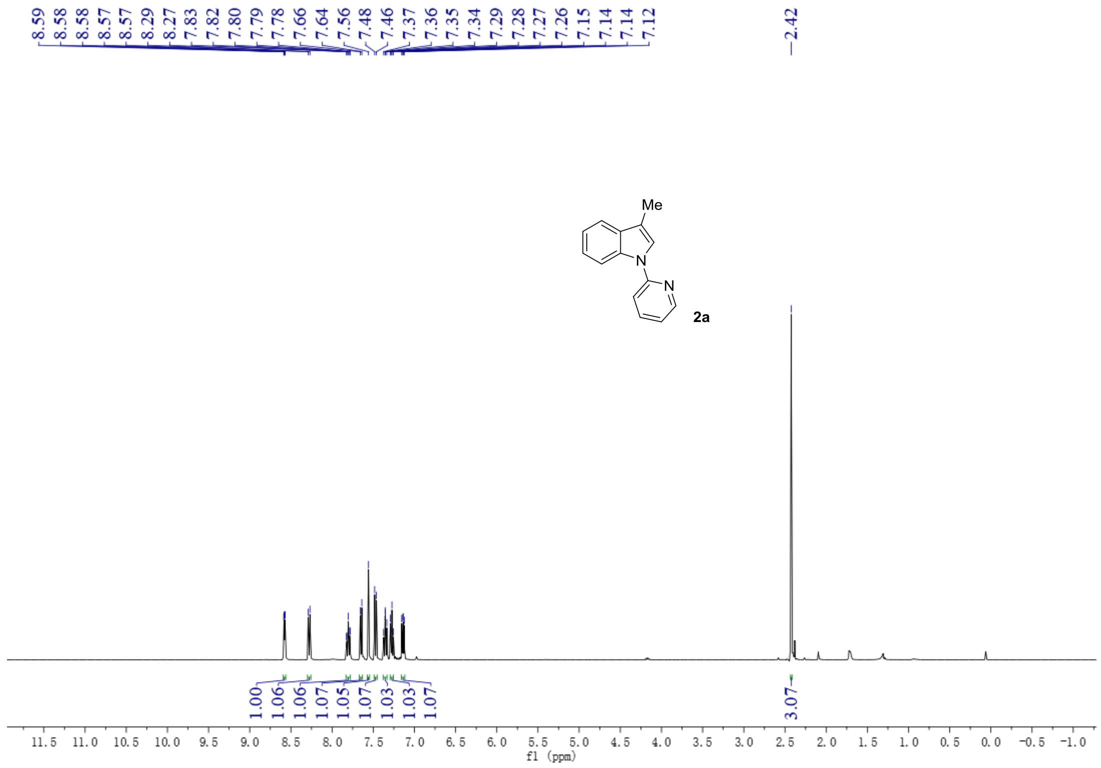

Supplementary Fig. 162 | ^{1}H NMR (400 MHz) of compound 2a (using $CDCl_3$ as solvent)

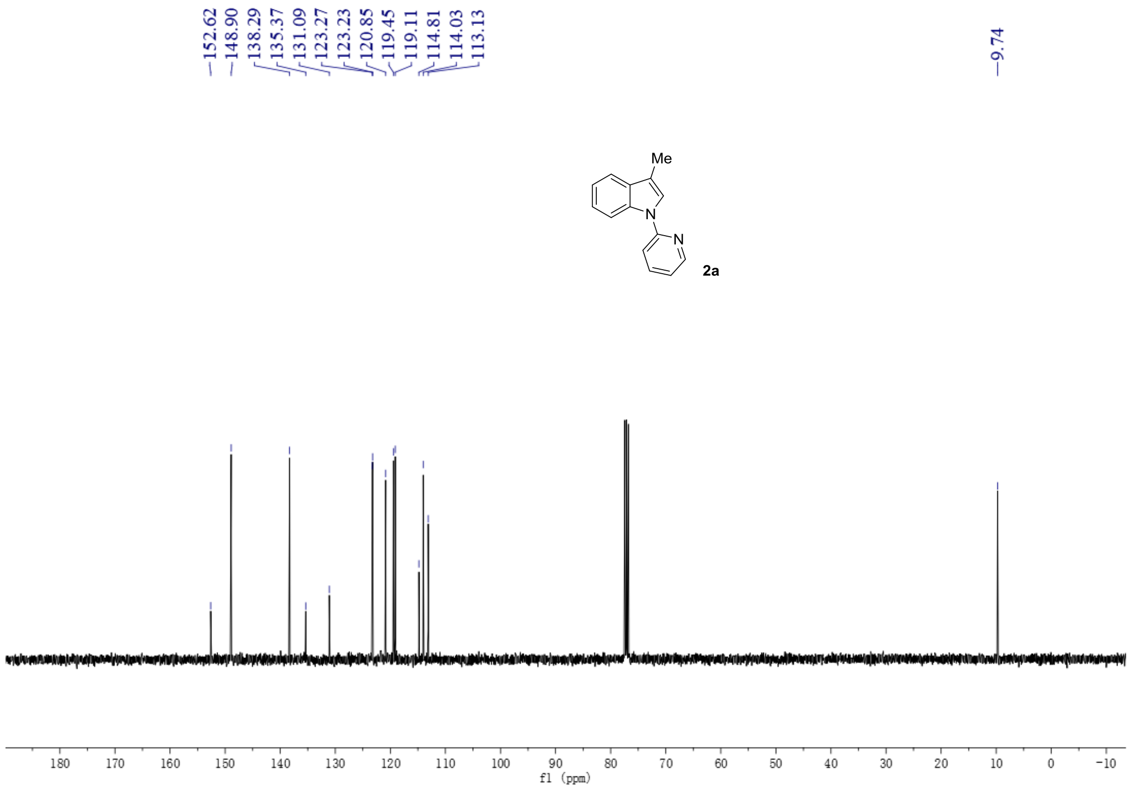

Supplementary Fig. 163 | ^{13}C NMR (101 MHz) of compound 2a (using $CDCl_3$ as solvent)

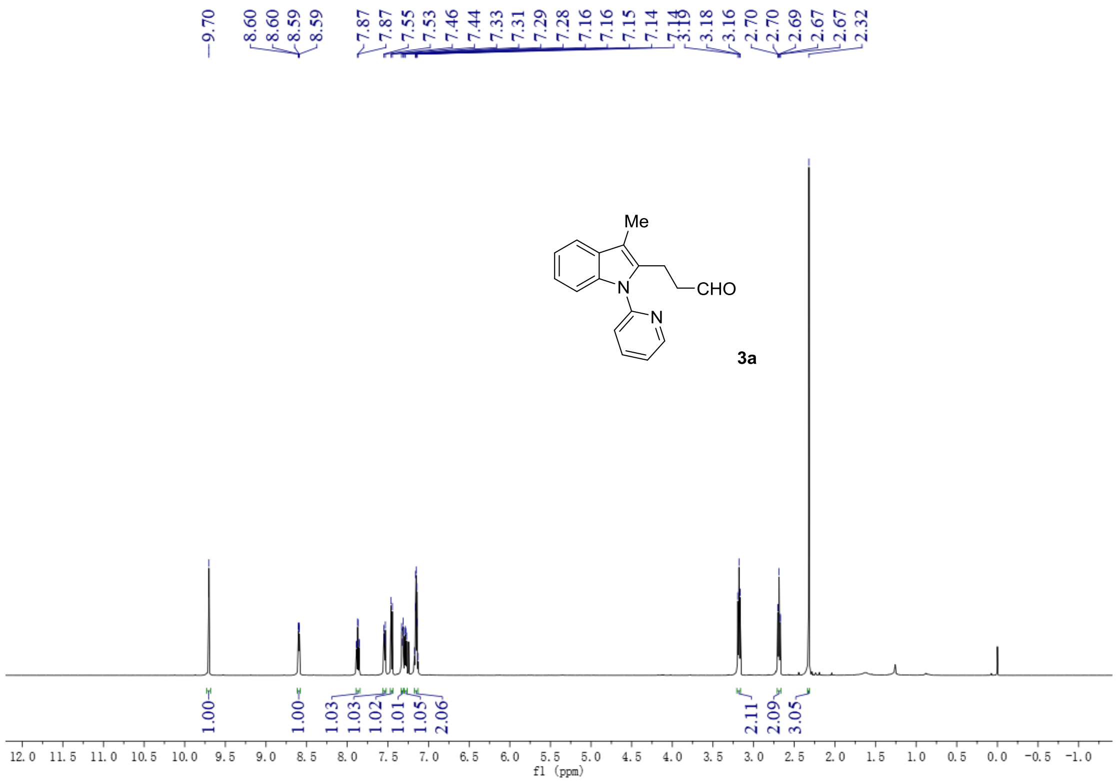

Supplementary Fig. 164 | ^{1}H NMR (500 MHz) of compound 3a (using $CDCl_3$ as solvent)

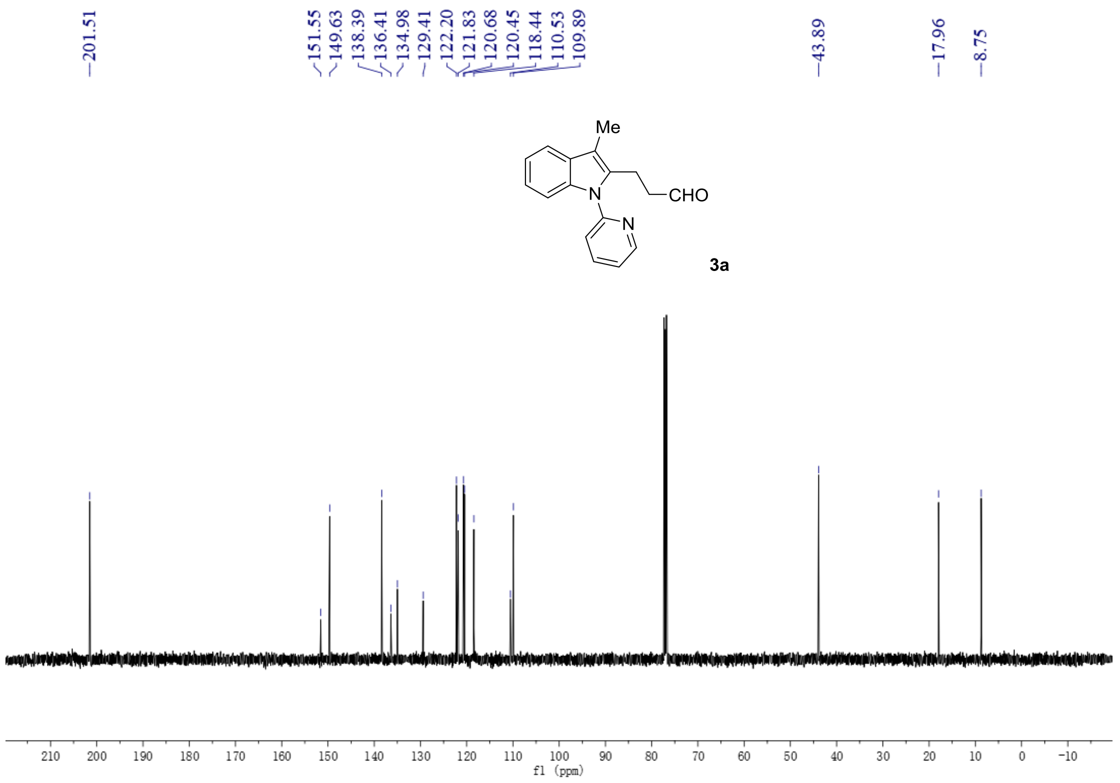

Supplementary Fig. 165 | ^{13}C NMR (126 MHz) of compound 3a (using $CDCl_3$ as solvent)

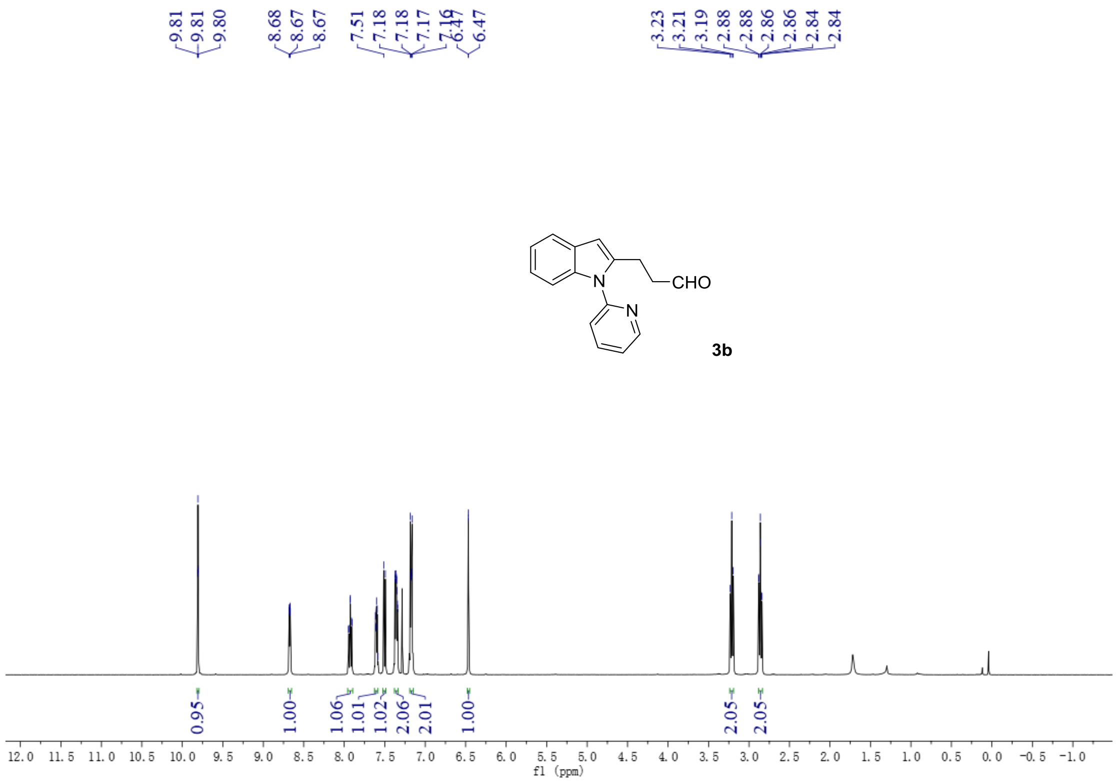

Supplementary Fig. 166 | ^{1}H NMR (400 MHz) of compound 3b (using $CDCl_3$ as solvent)

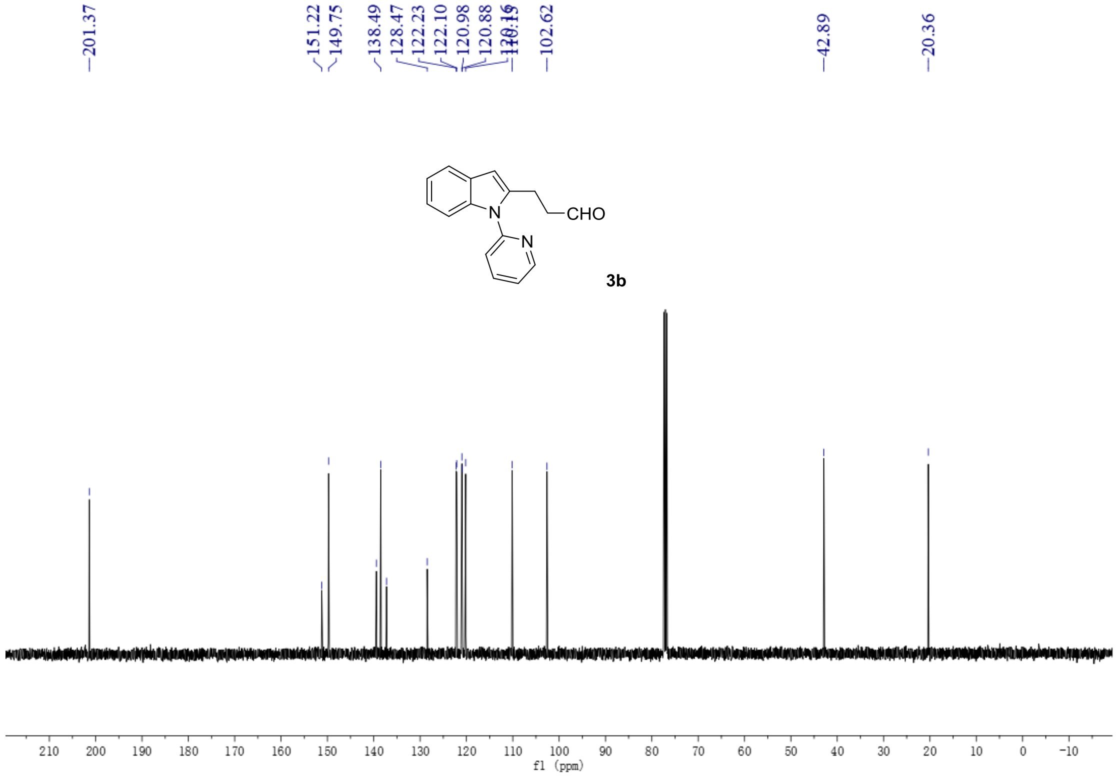

Supplementary Fig. 167 | ^{13}C NMR (101 MHz) of compound 3b (using $CDCl_3$ as solvent)

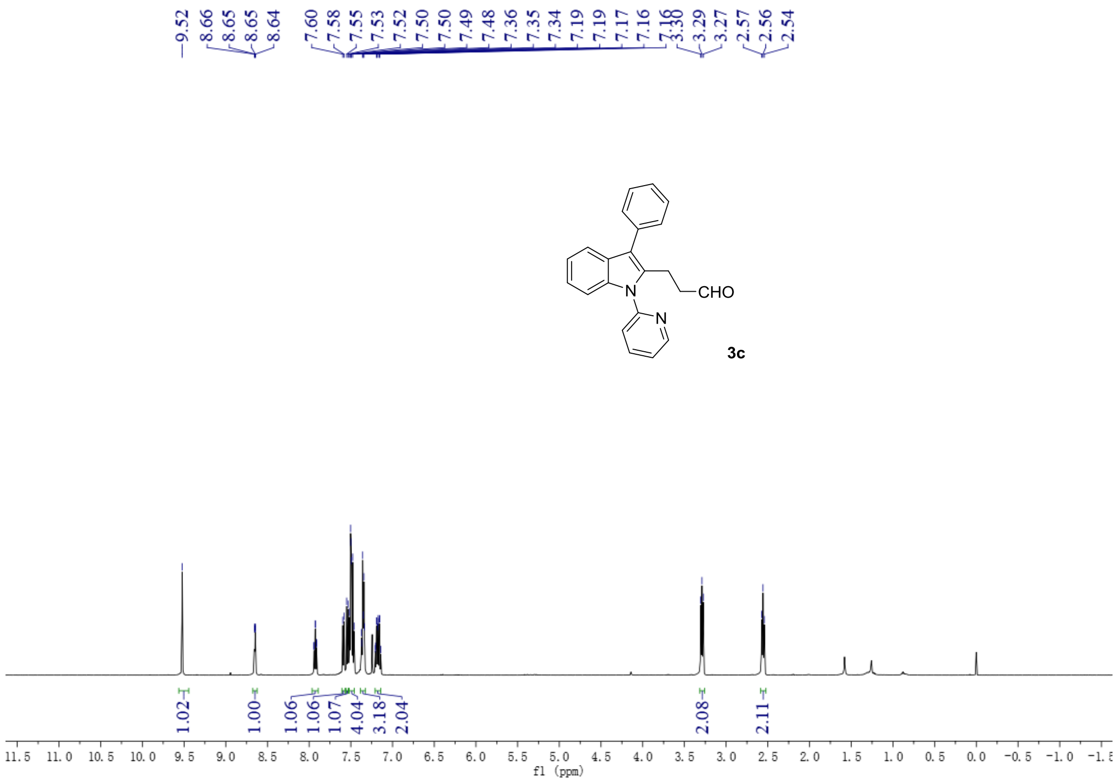

Supplementary Fig. 168 | ^{1}H NMR (500 MHz) of compound 3c (using $CDCl_3$ as solvent)

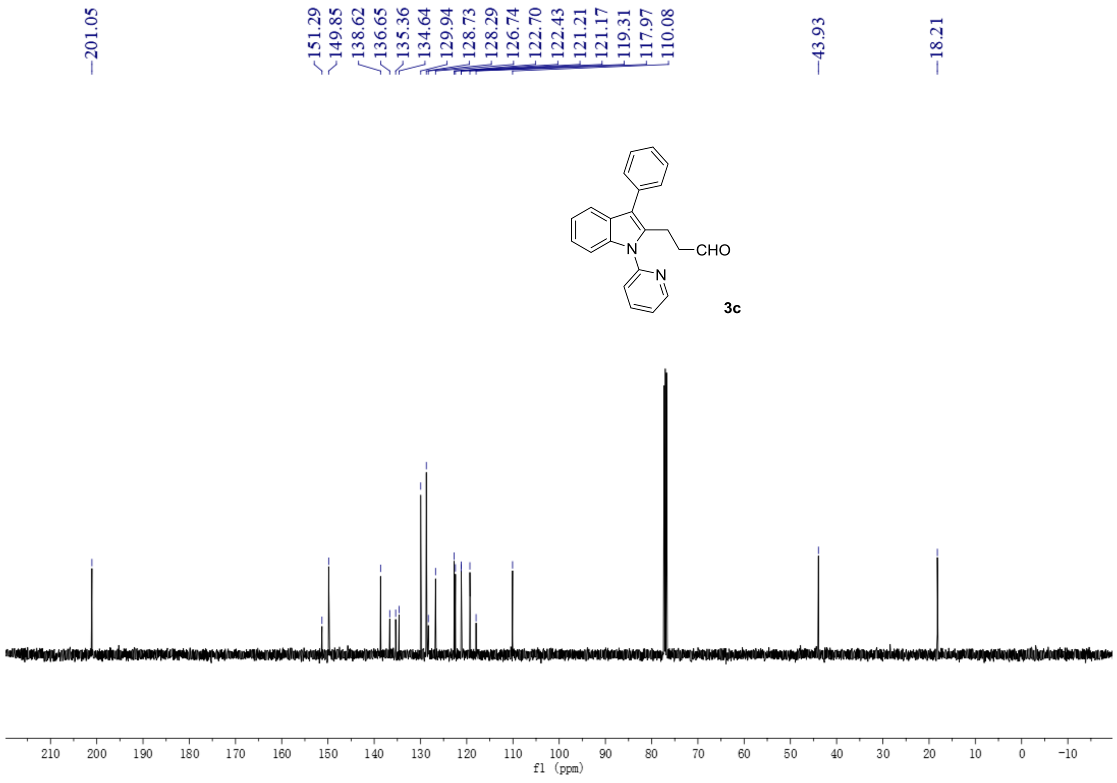

Supplementary Fig. 169 | ^{13}C NMR (126 MHz) of compound 3c (using $CDCl_3$ as solvent)

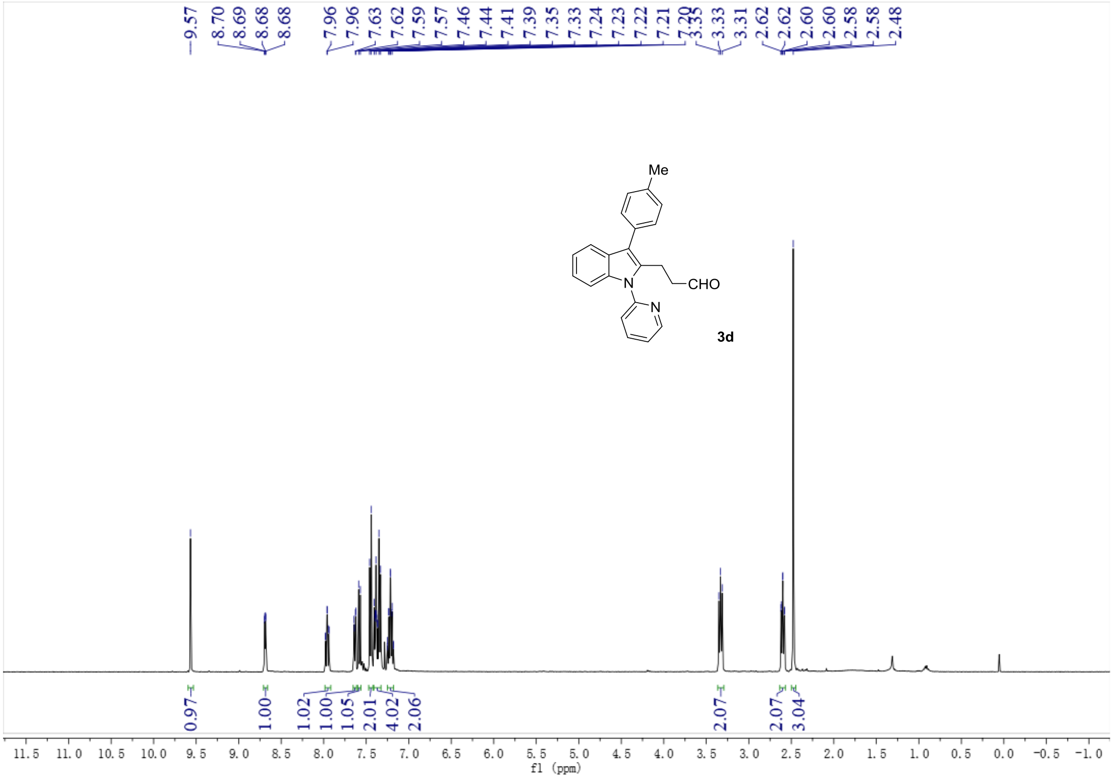

Supplementary Fig. 170 | ^{1}H NMR (400 MHz) of compound 3d (using $CDCl_3$ as solvent)

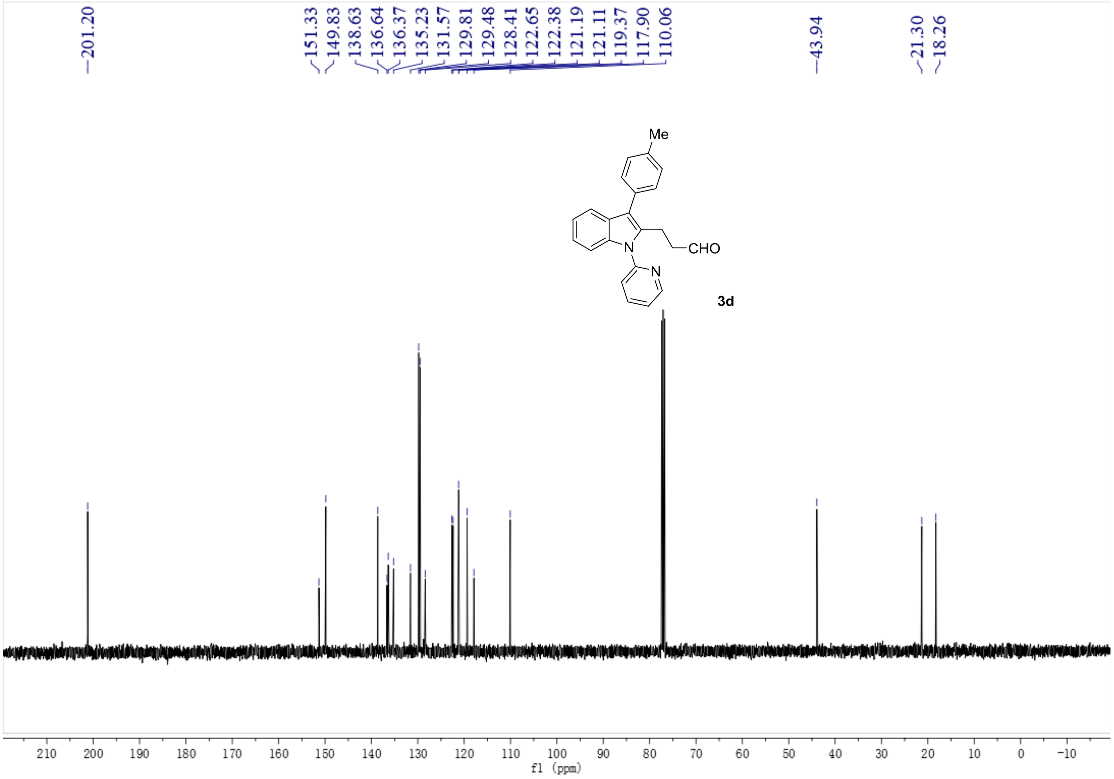

Supplementary Fig. 171 | ^{13}C NMR (101 MHz) of compound 3d (using $CDCl_3$ as solvent)

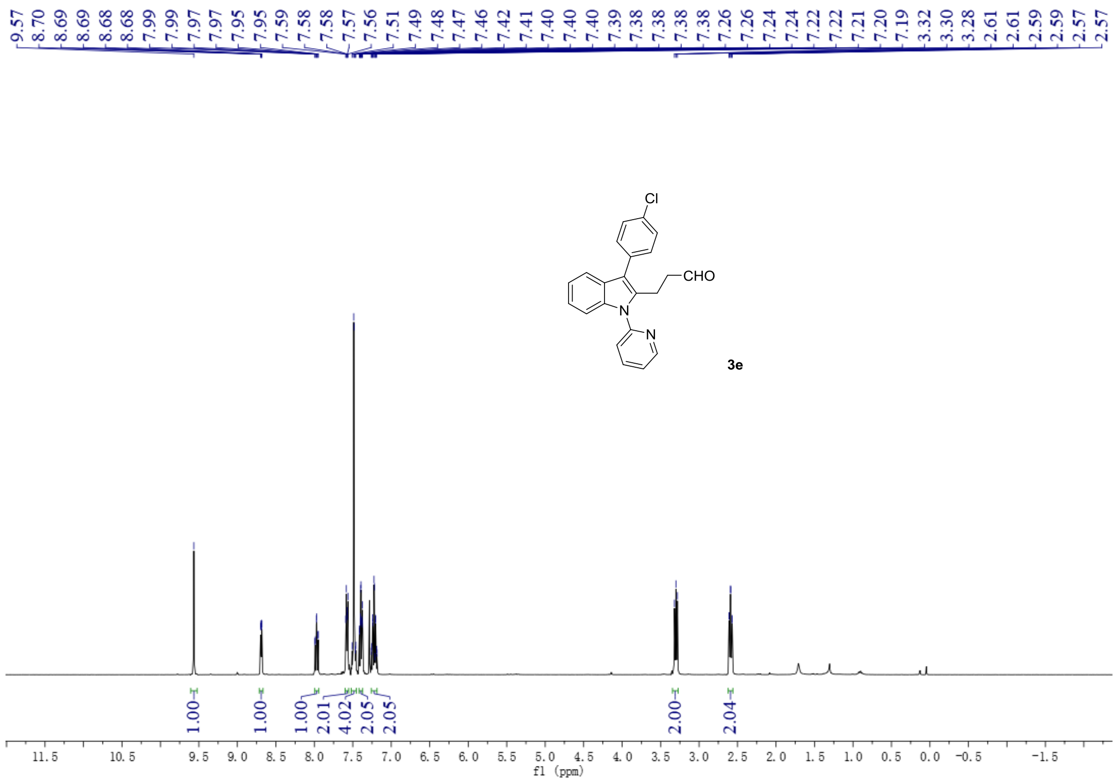

Supplementary Fig. 172 | ^{1}H NMR (400 MHz) of compound 3e (using $CDCl_3$ as solvent)

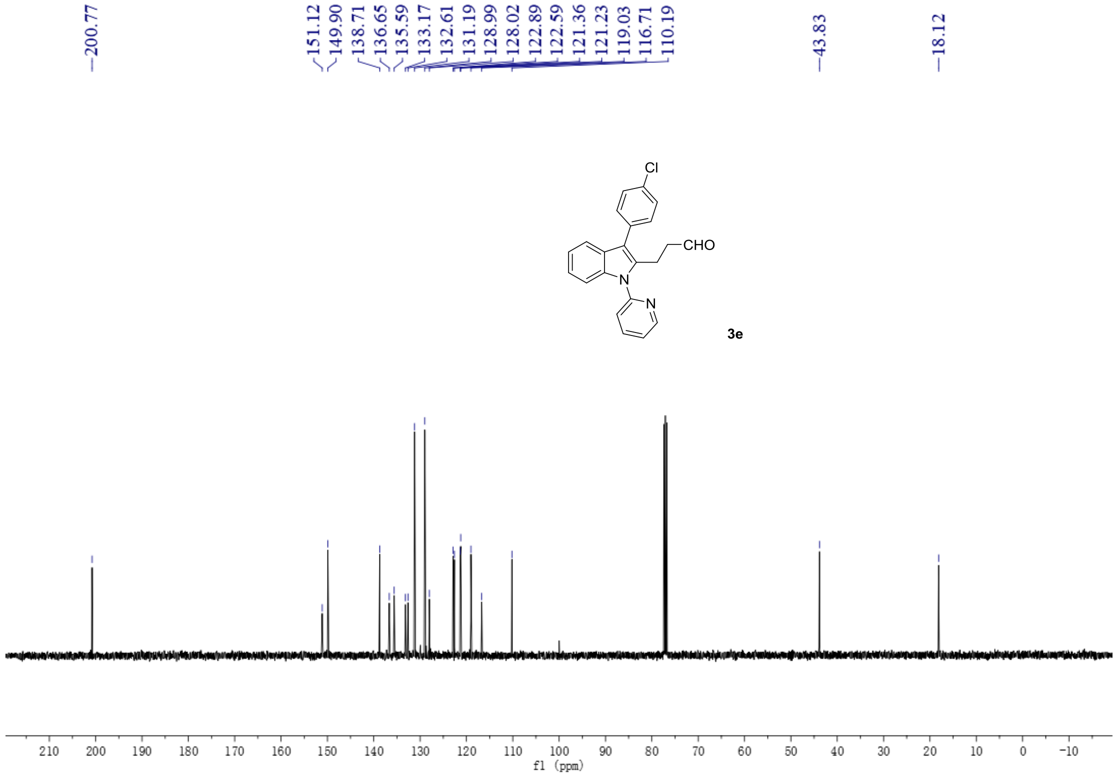

Supplementary Fig. 173 | ^{13}C NMR (101 MHz) of compound 3e (using $CDCl_3$ as solvent)

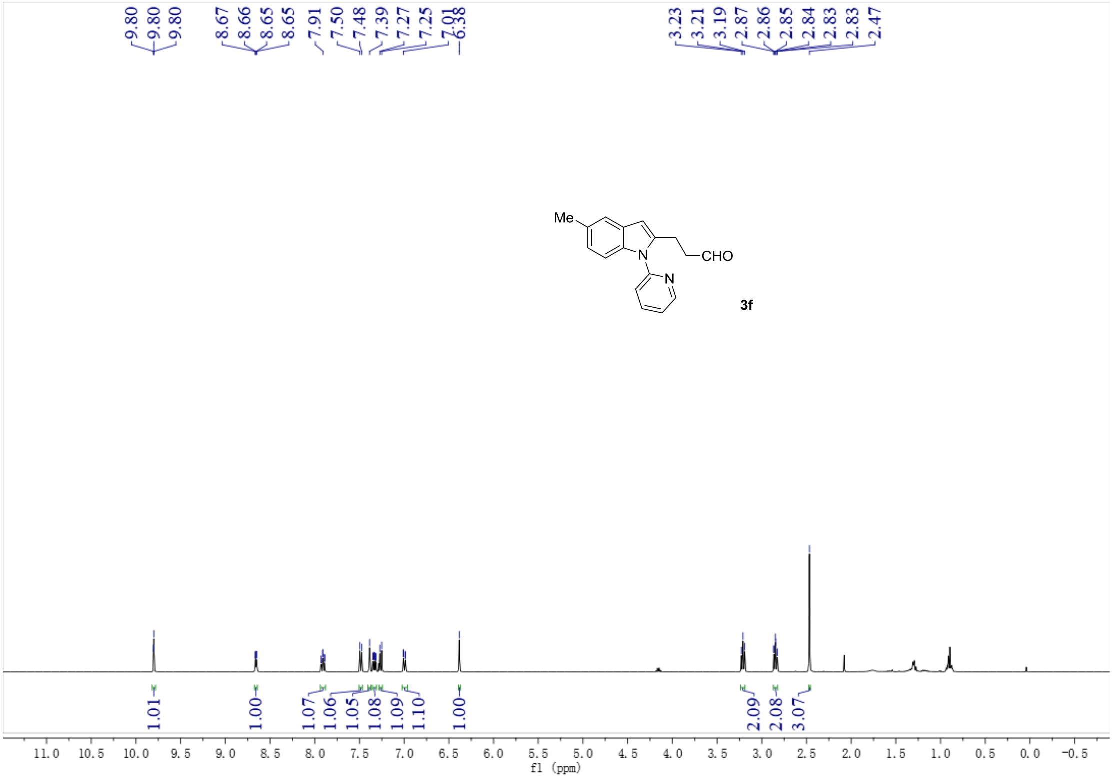

Supplementary Fig. 174 | ^{1}H NMR (400 MHz) of compound 3f (using $CDCl_3$ as solvent)

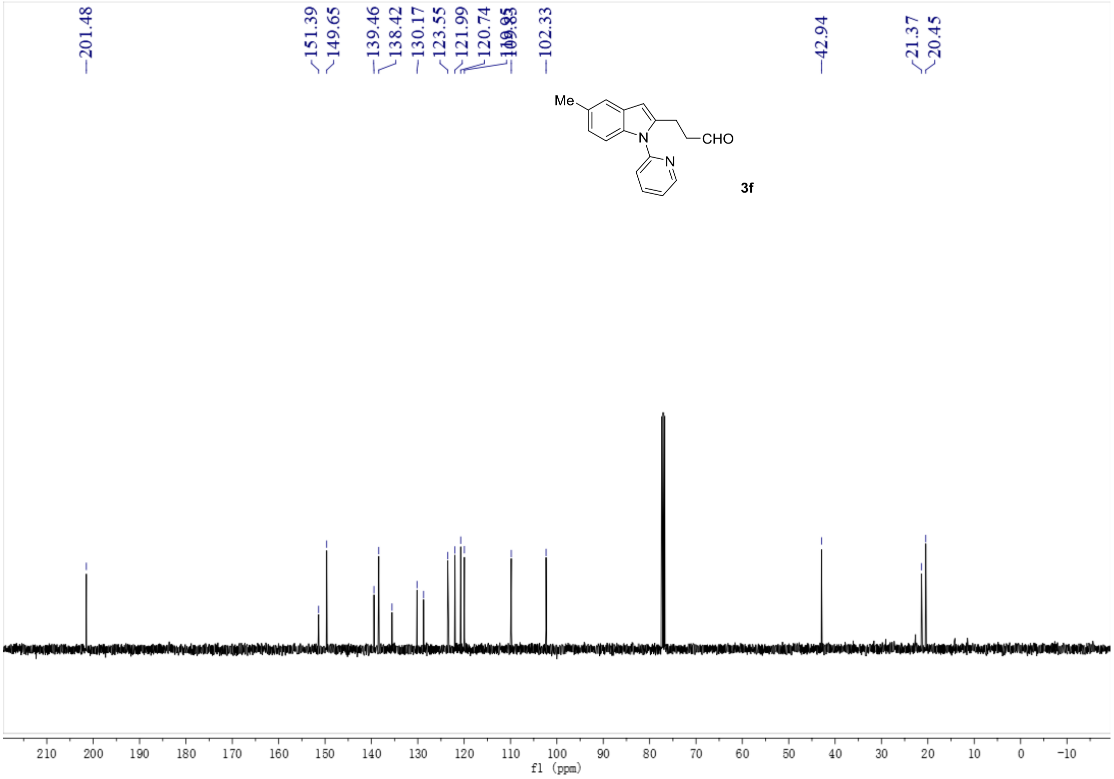

Supplementary Fig. 175 | ^{13}C NMR (101 MHz) of compound 3f (using $CDCl_3$ as solvent)

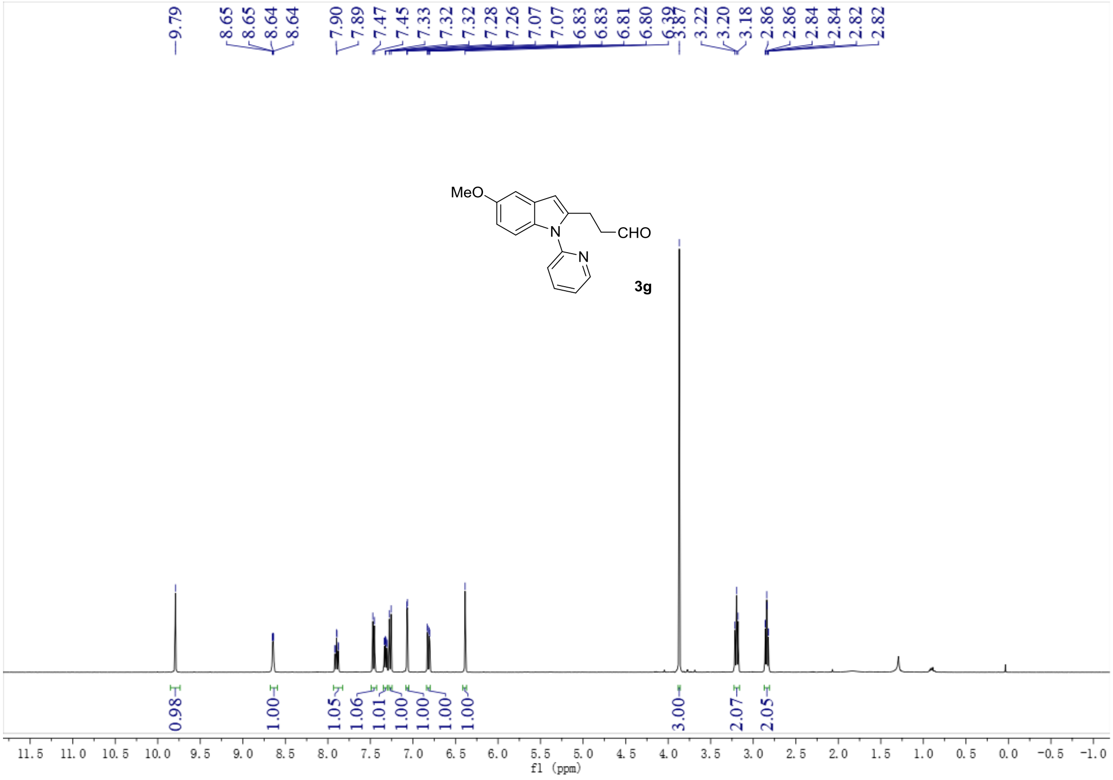

Supplementary Fig. 176 | ^{1}H NMR (400 MHz) of compound 3g (using $CDCl_3$ as solvent)

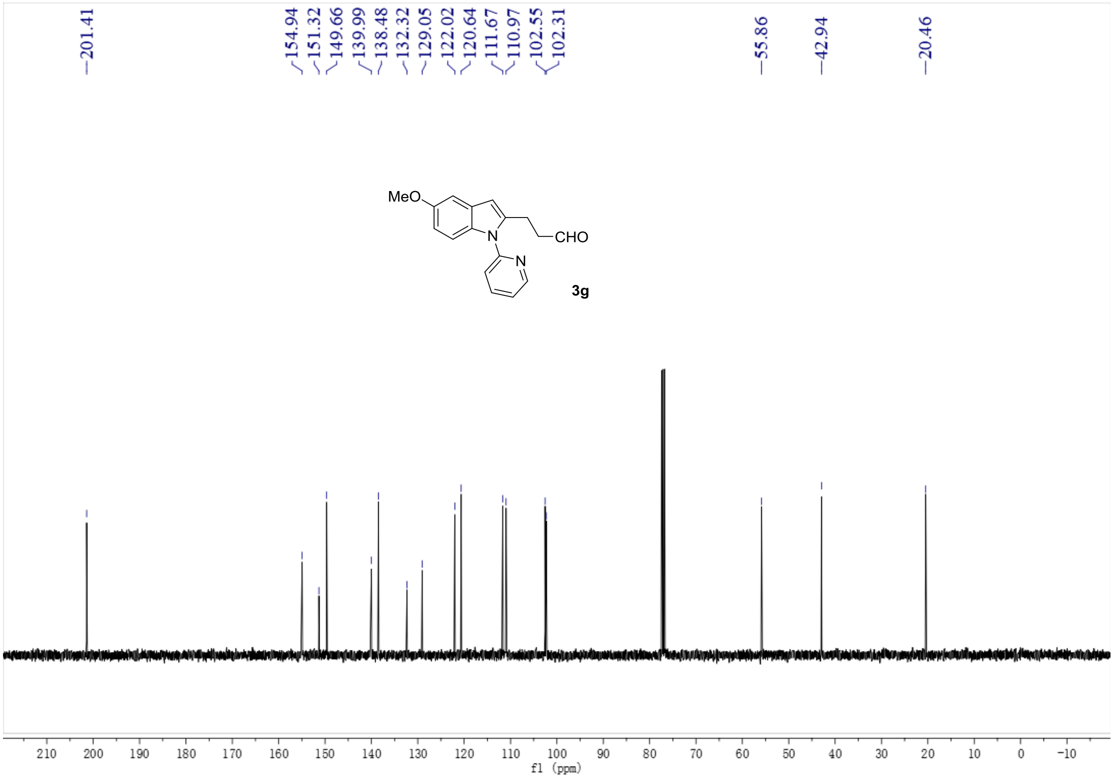

Supplementary Fig. 177 | ^{13}C NMR (101 MHz) of compound 3g (using $CDCl_3$ as solvent)

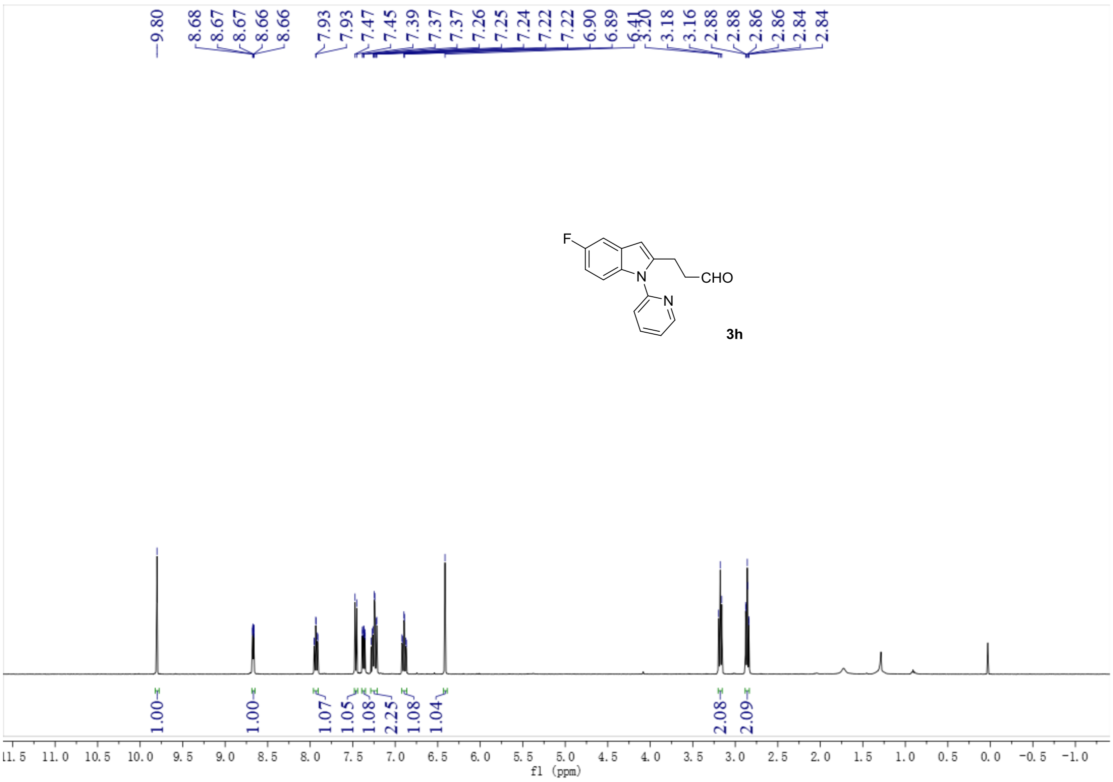

Supplementary Fig. 178 | ^{1}H NMR (400 MHz) of compound 3h (using $CDCl_3$ as solvent)

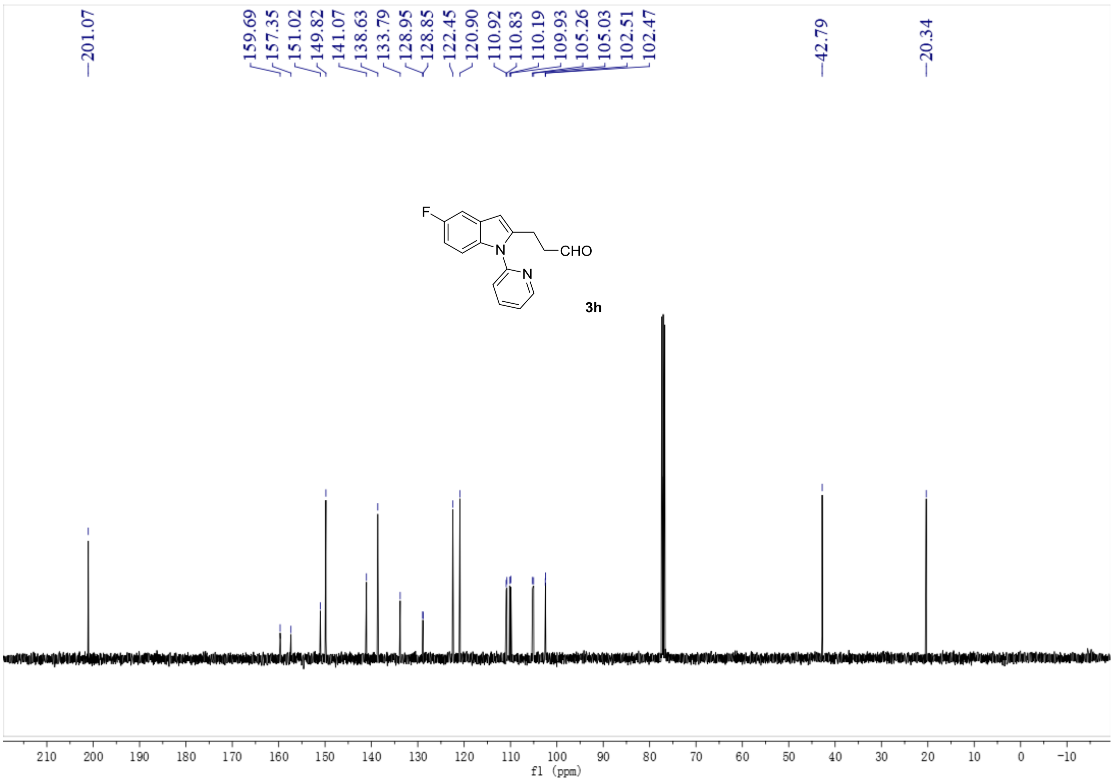

Supplementary Fig. 179 | ^{13}C NMR (101 MHz) of compound 3h (using $CDCl_3$ as solvent)

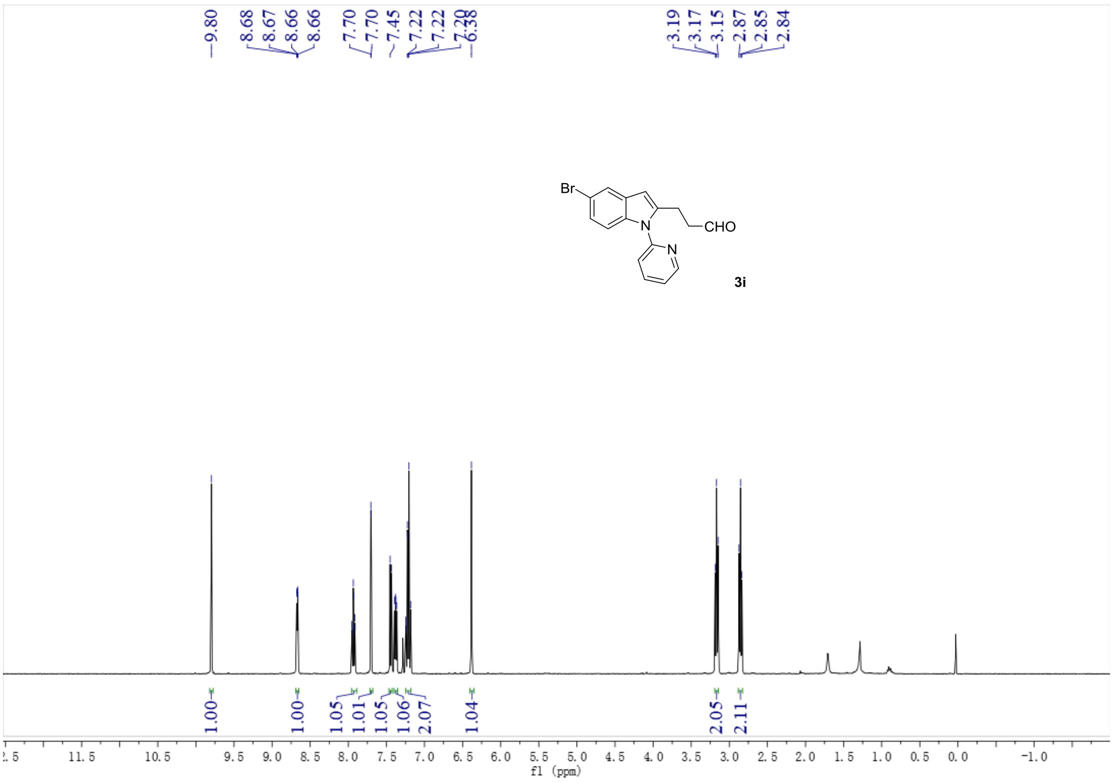

Supplementary Fig. 180 | ^{1}H NMR (400 MHz) of compound 3i (using $CDCl_3$ as solvent)

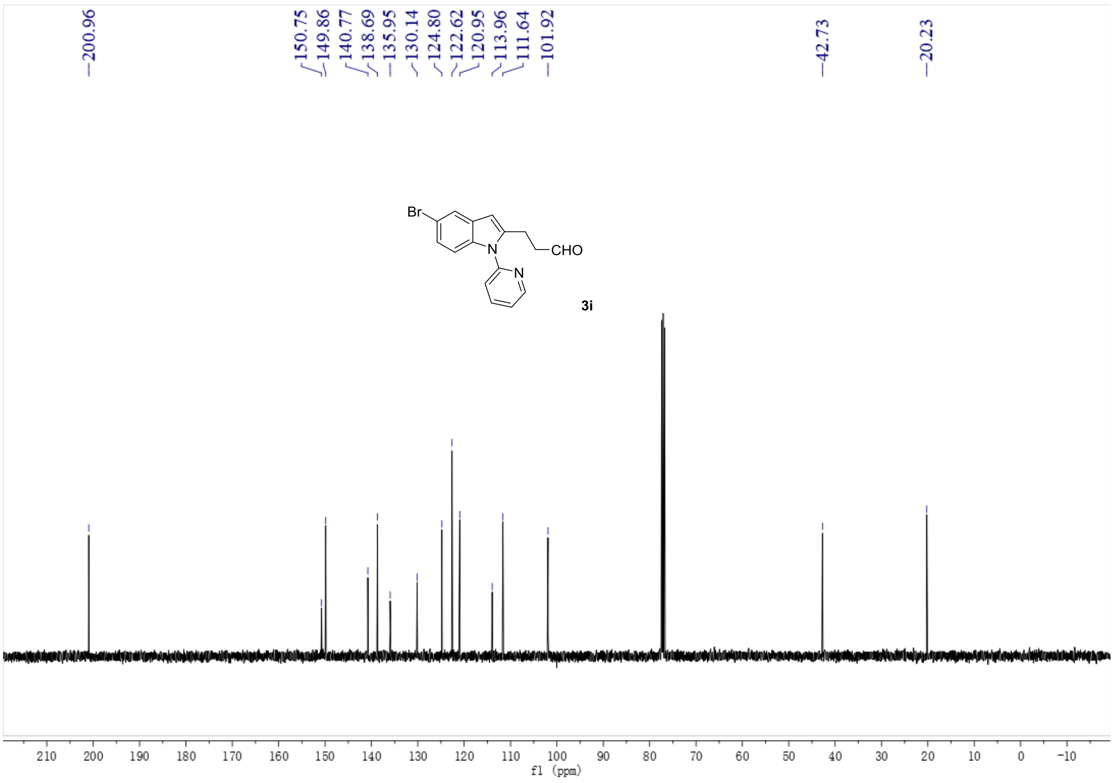

Supplementary Fig. 181 | ^{13}C NMR (101 MHz) of compound 3i (using $CDCl_3$ as solvent)

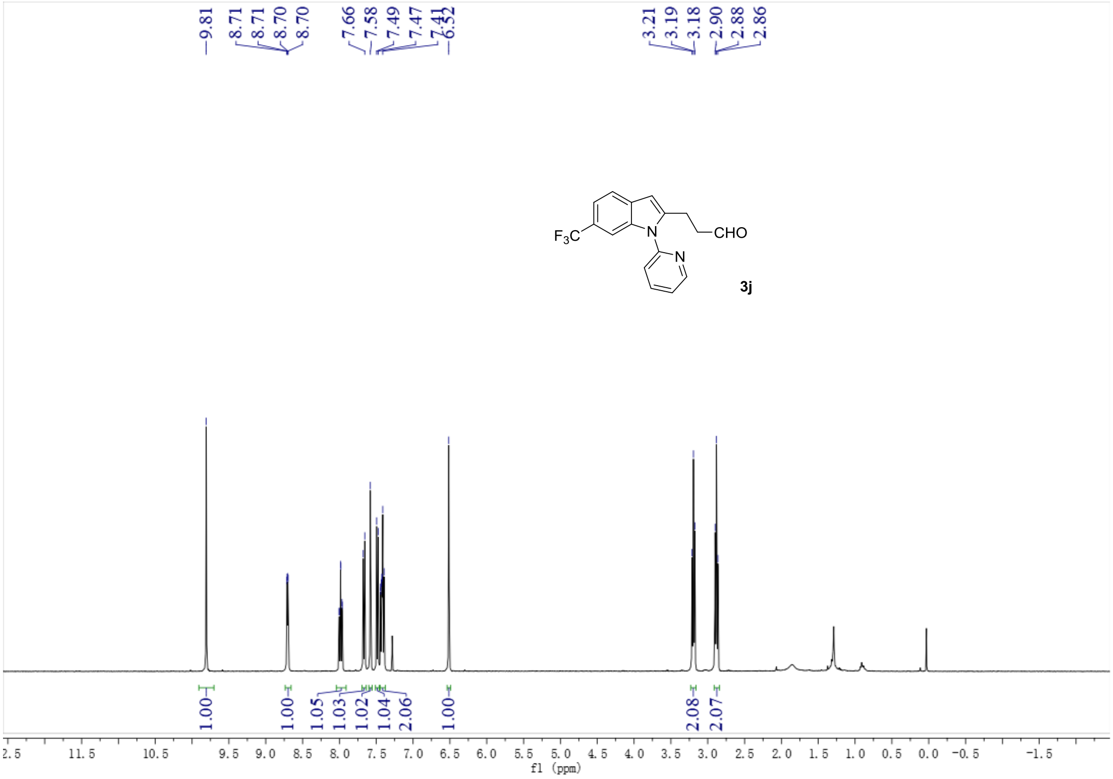

Supplementary Fig. 182 | ^{1}H NMR (400 MHz) of compound 3j (using $CDCl_3$ as solvent)

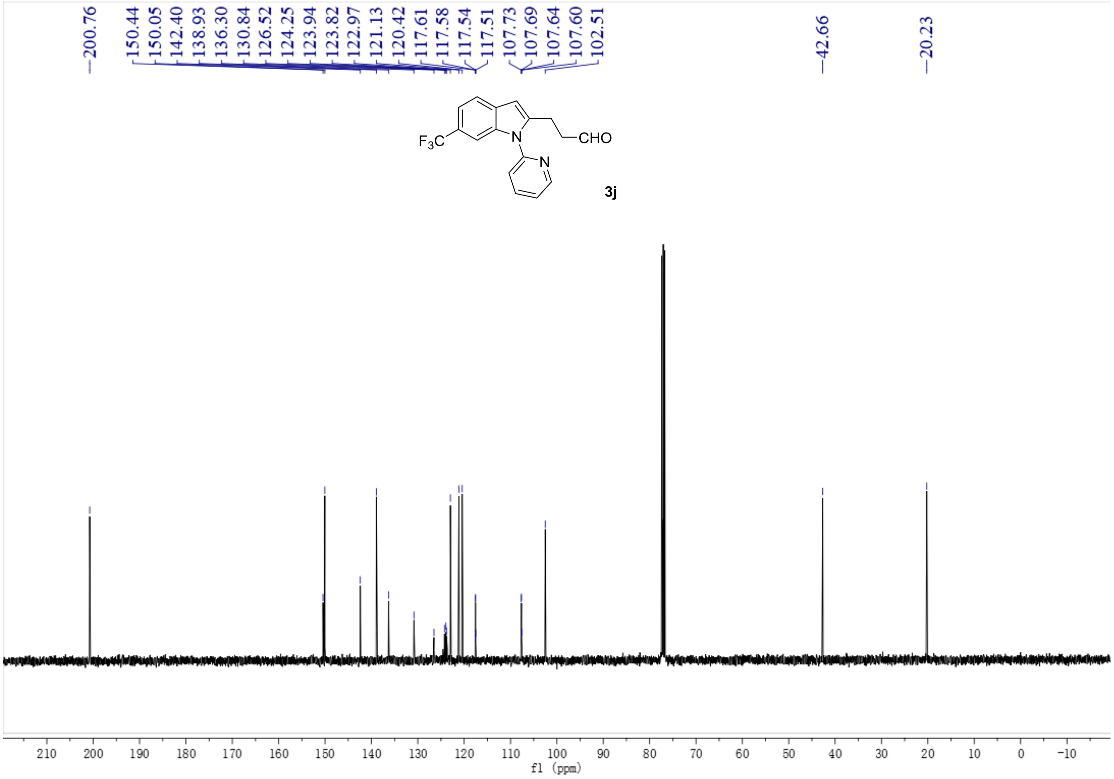

Supplementary Fig. 183 | ^{13}C NMR (101 MHz) of compound 3j (using $CDCl_3$ as solvent)

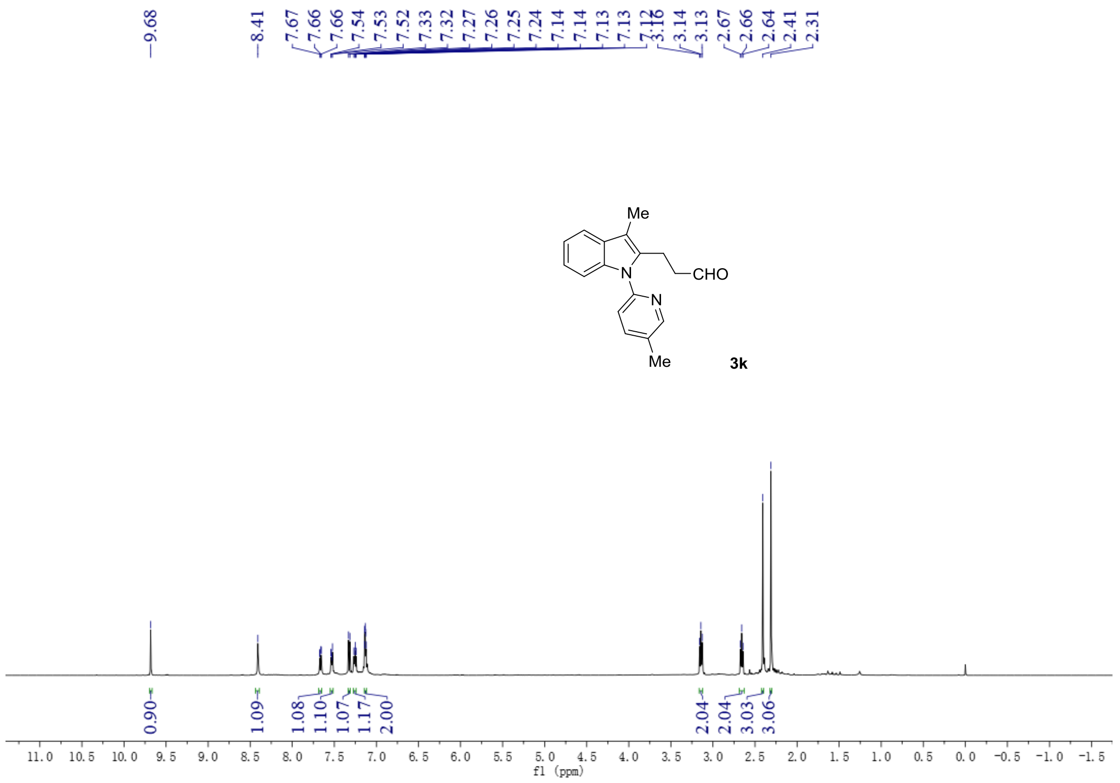

Supplementary Fig. 184 | ^{1}H NMR (500 MHz) of compound 3k (using $CDCl_3$ as solvent)

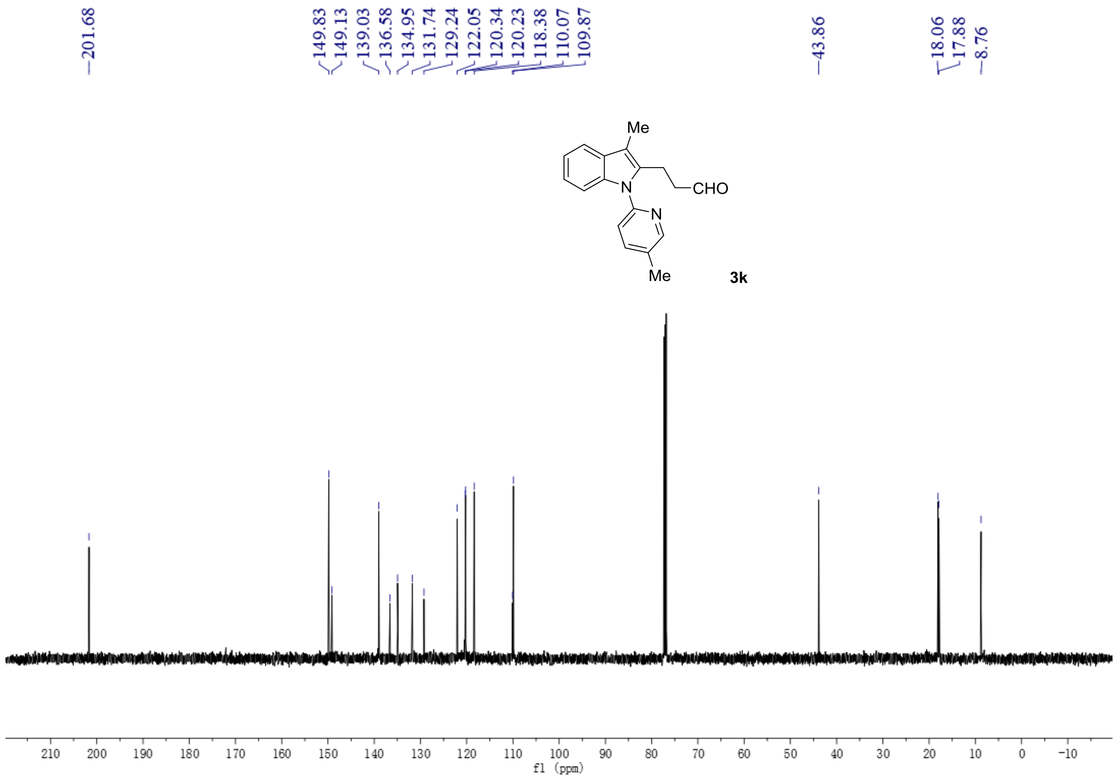

Supplementary Fig. 185 | ^{13}C NMR (126 MHz) of compound 3k (using $CDCl_3$ as solvent)

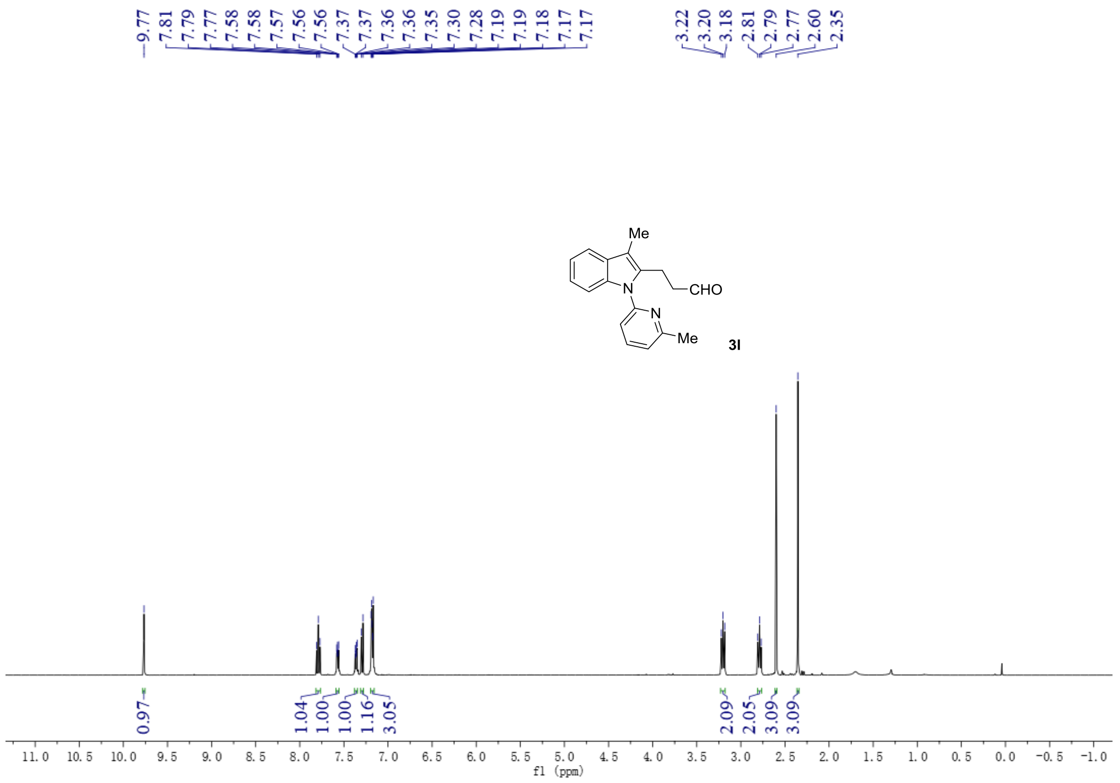

Supplementary Fig. 186 | ^{1}H NMR (400 MHz) of compound 3l (using $CDCl_3$ as solvent)

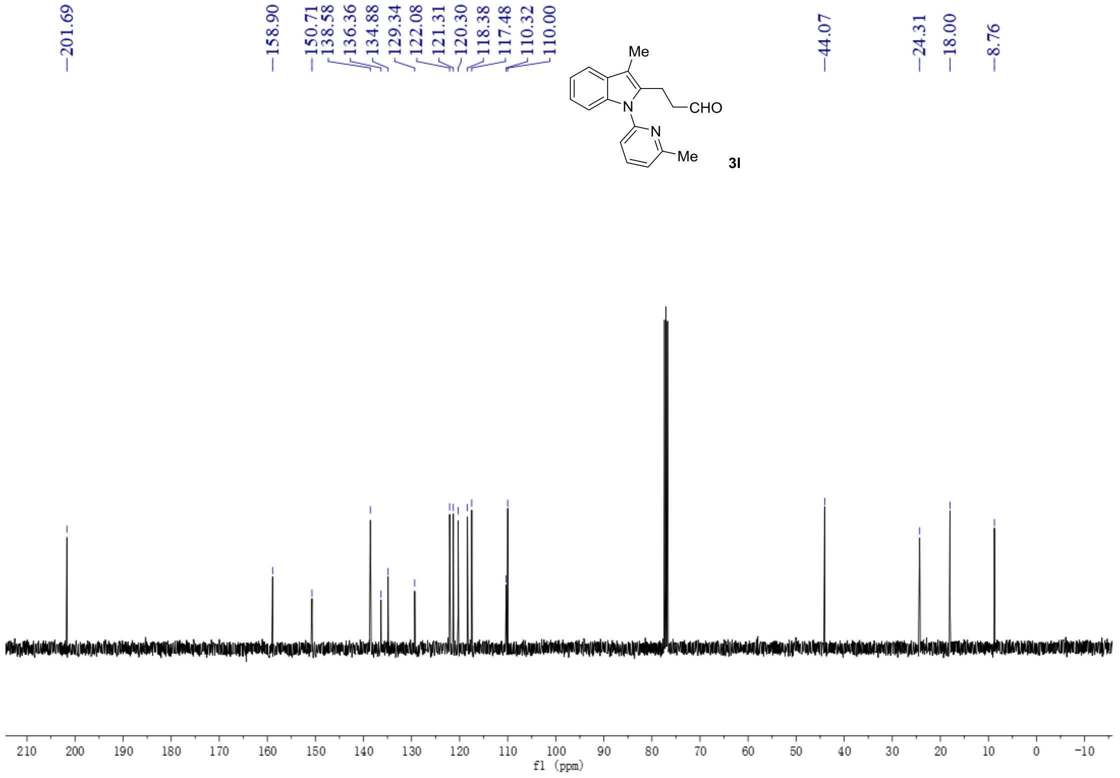

Supplementary Fig. 187 | ^{13}C NMR (101 MHz) of compound 3l (using $CDCl_3$ as solvent)

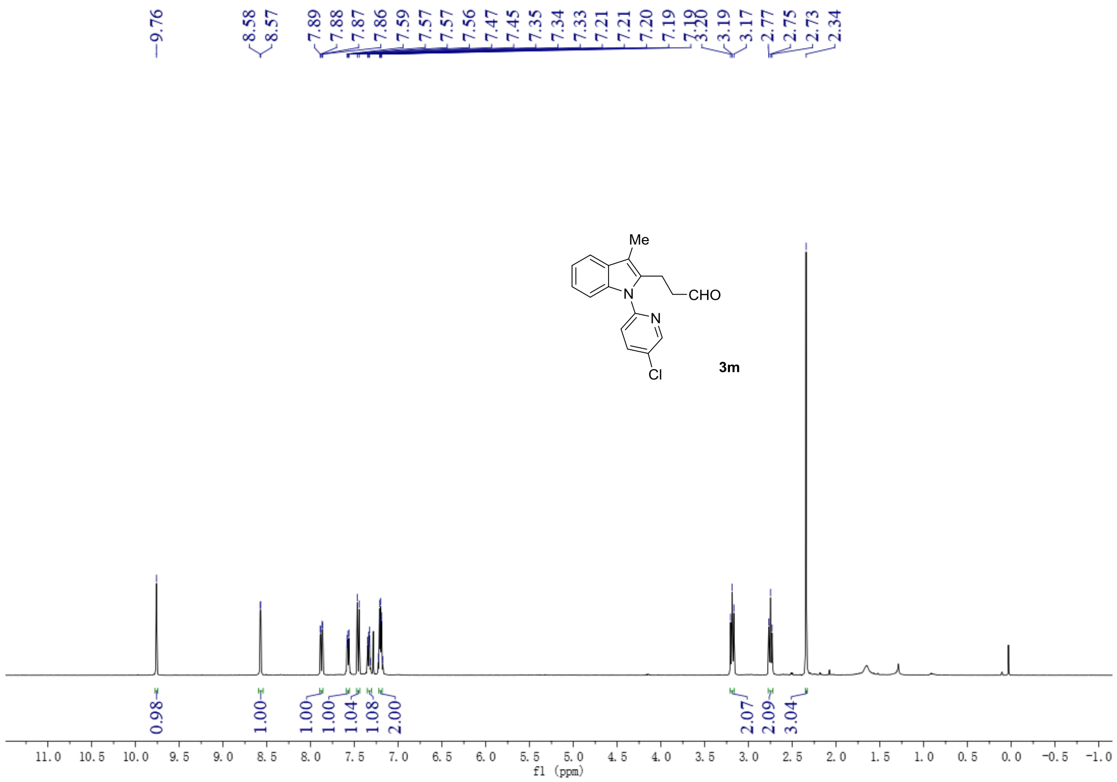

Supplementary Fig. 188 | ^{1}H NMR (400 MHz) of compound 3m (using $CDCl_3$ as solvent)

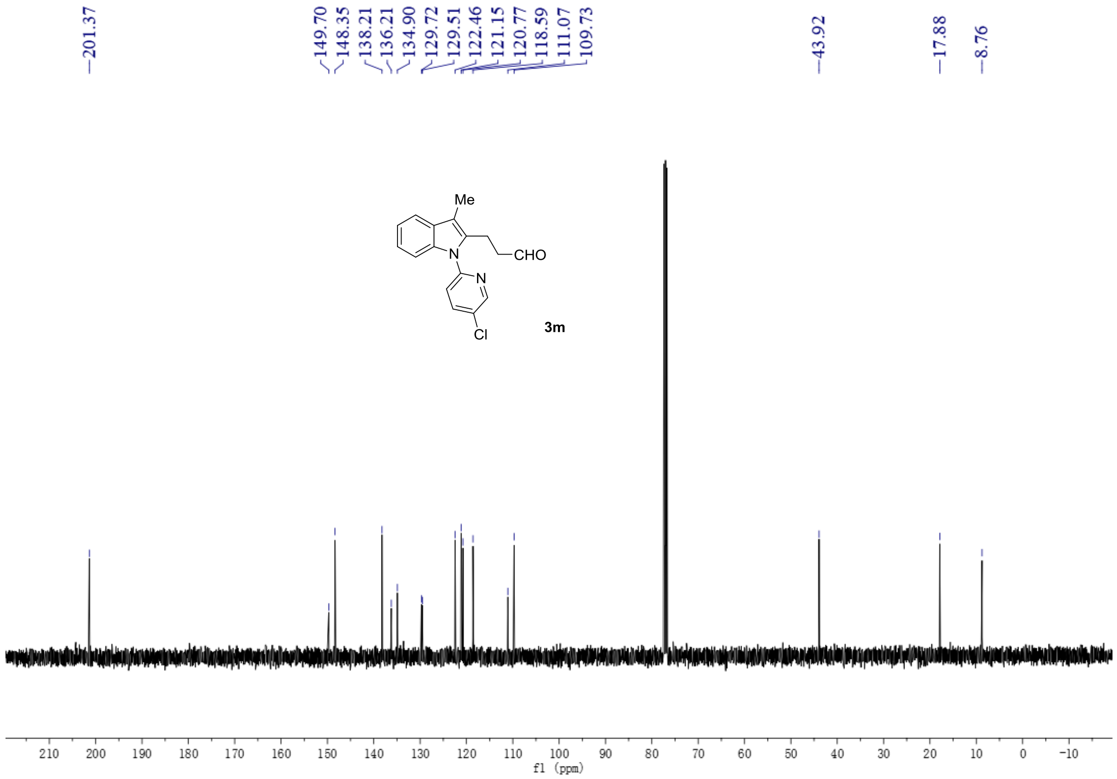

Supplementary Fig. 189 | ^{13}C NMR (101 MHz) of compound 3m (using $CDCl_3$ as solvent)

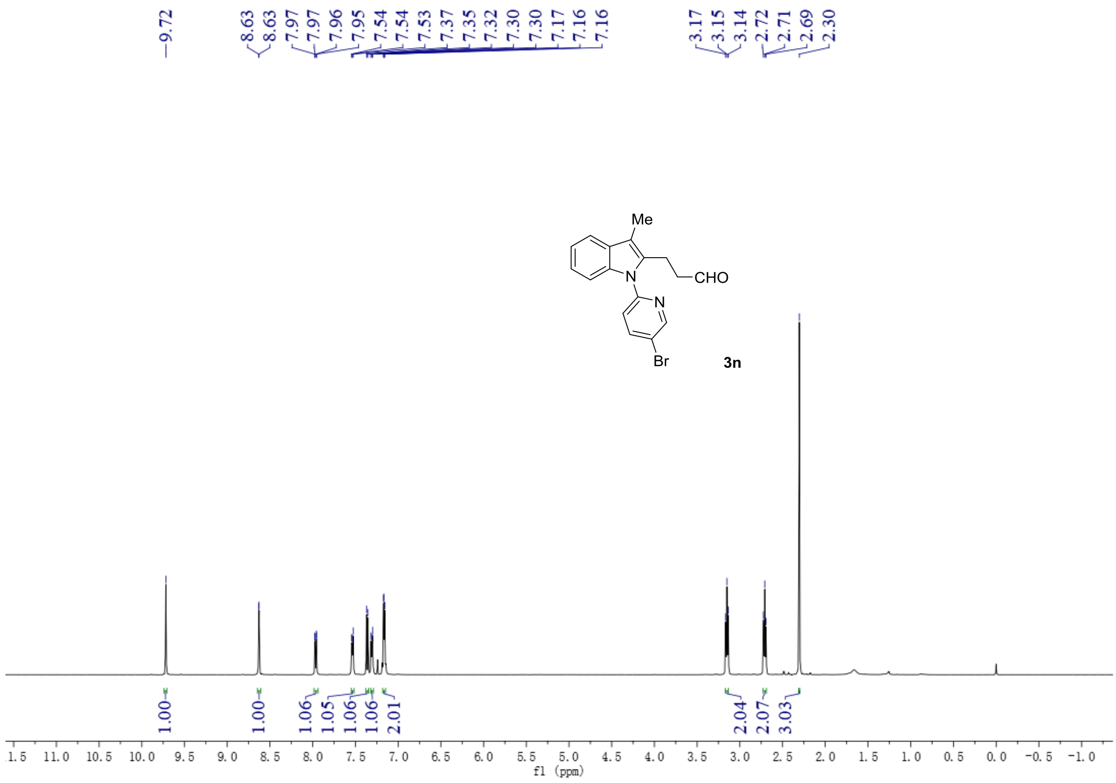

Supplementary Fig. 190 | ^{1}H NMR (500 MHz) of compound 3n (using $CDCl_3$ as solvent)

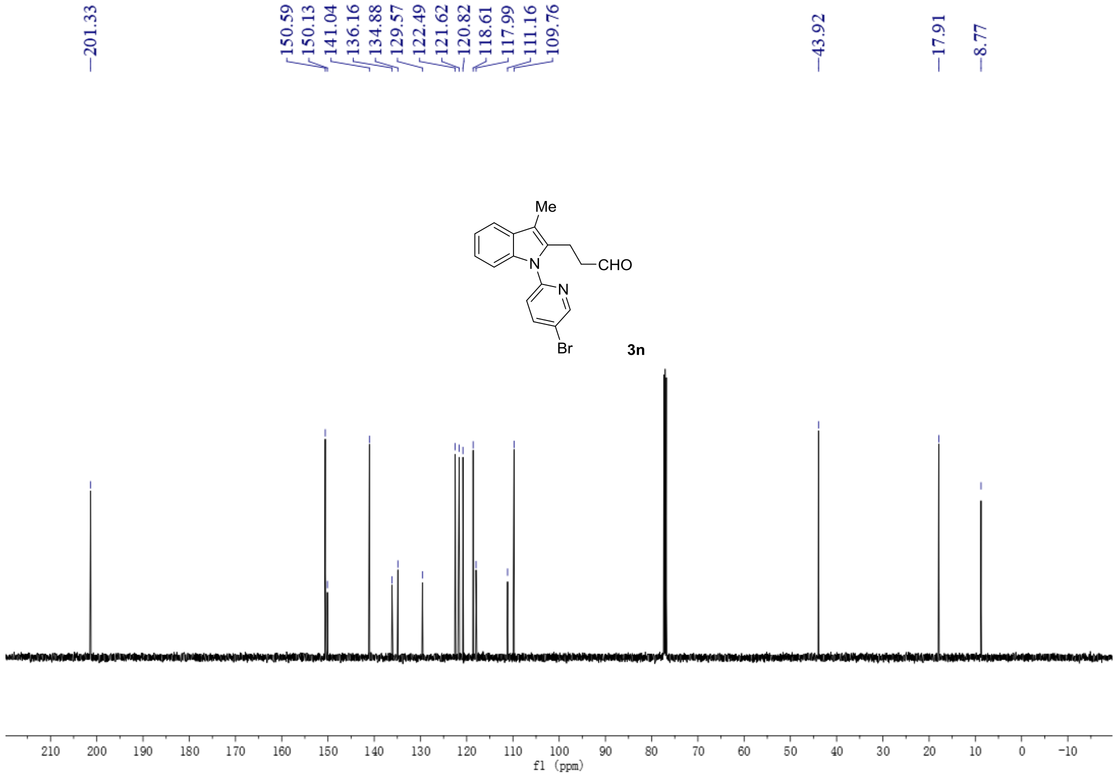

Supplementary Fig. 191 | ^{13}C NMR (126 MHz) of compound 3n (using $CDCl_3$ as solvent)

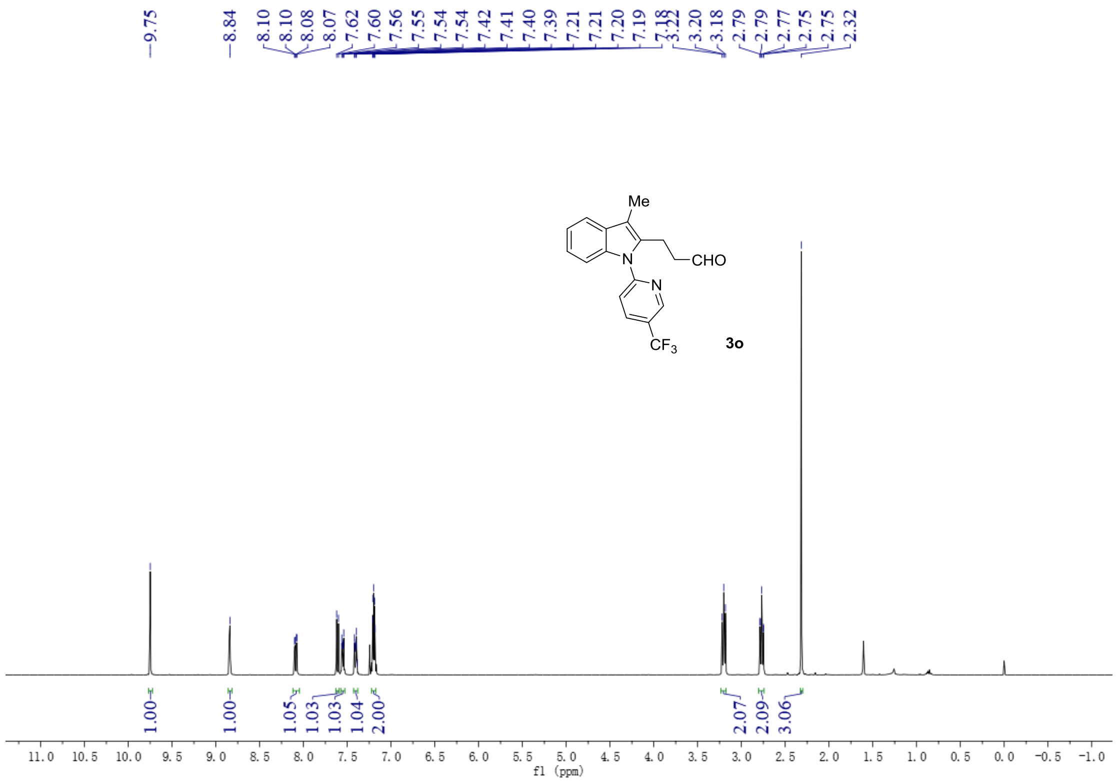

Supplementary Fig. 192 | ^{1}H NMR (400 MHz) of compound 3o (using $CDCl_3$ as solvent)

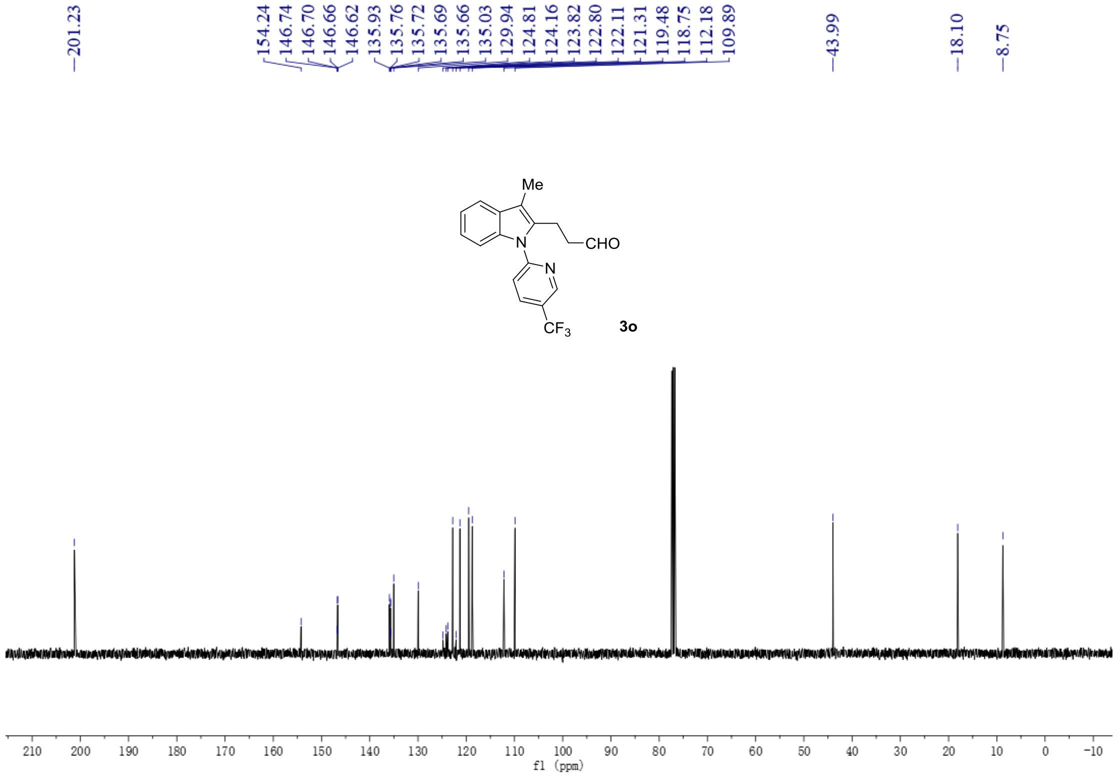

Supplementary Fig. 193 | ^{13}C NMR (101 MHz) of compound 3o (using $CDCl_3$ as solvent)

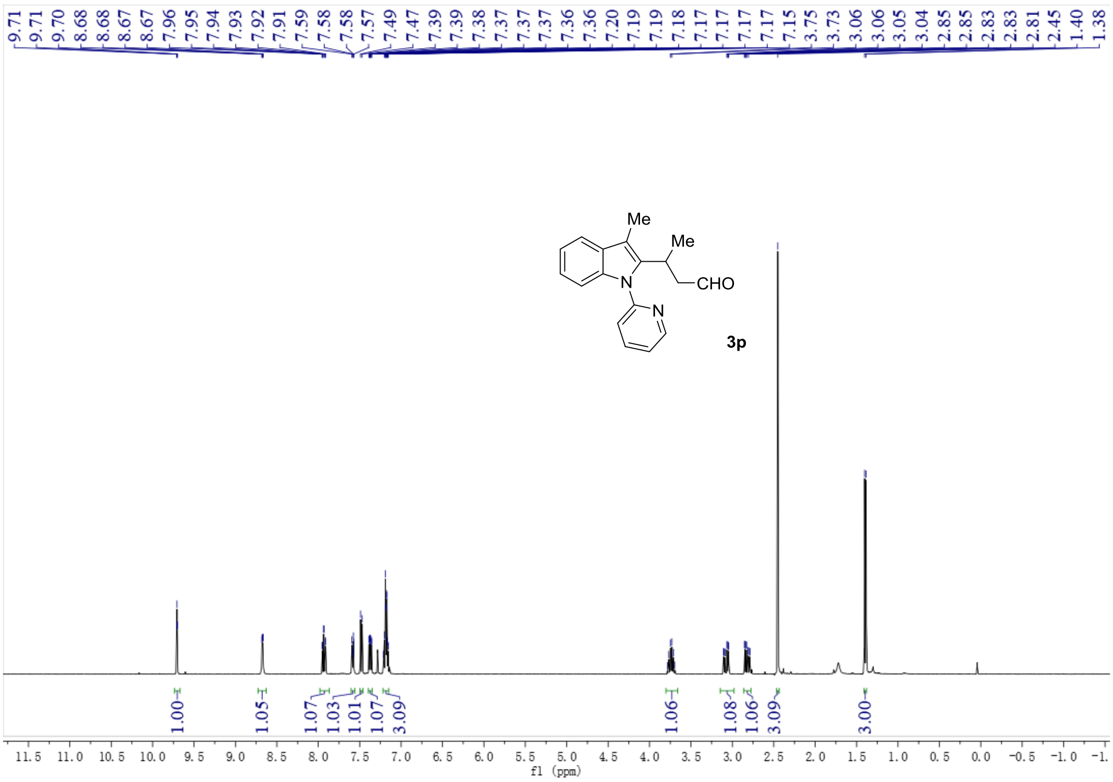

Supplementary Fig. 194 | ^{1}H NMR (400 MHz) of compound 3p (using $CDCl_3$ as solvent)

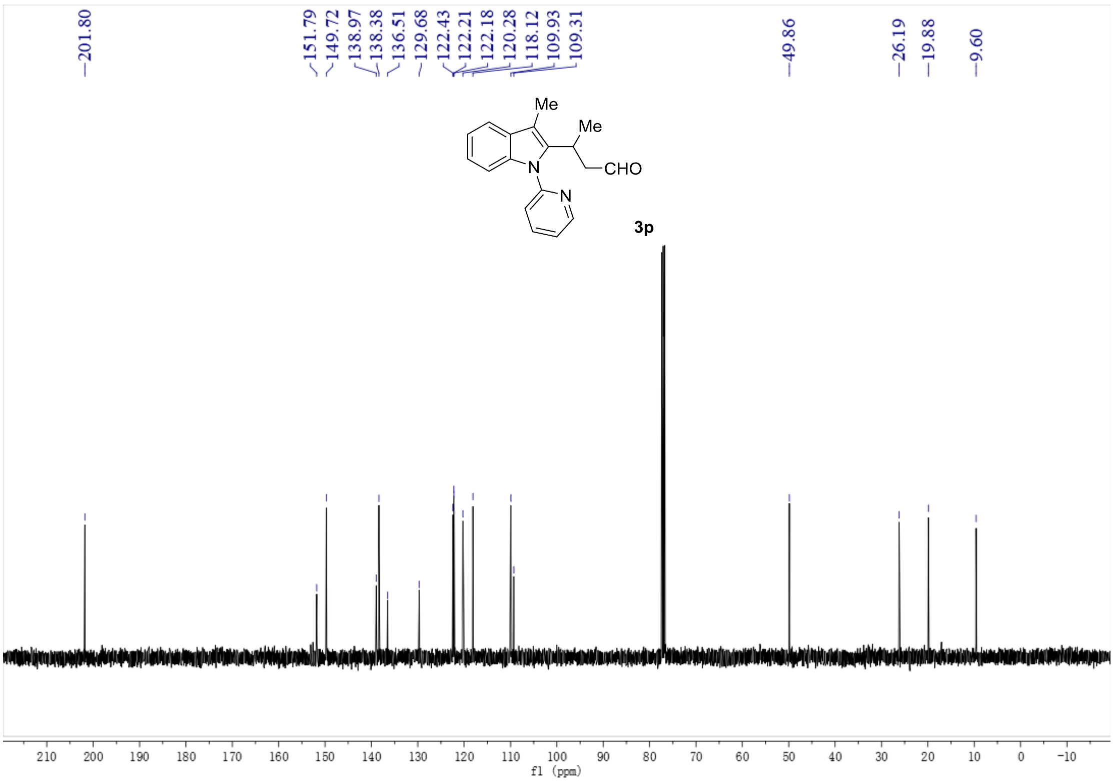

Supplementary Fig. 195 | ^{13}C NMR (101 MHz) of compound 3p (using $CDCl_3$ as solvent)

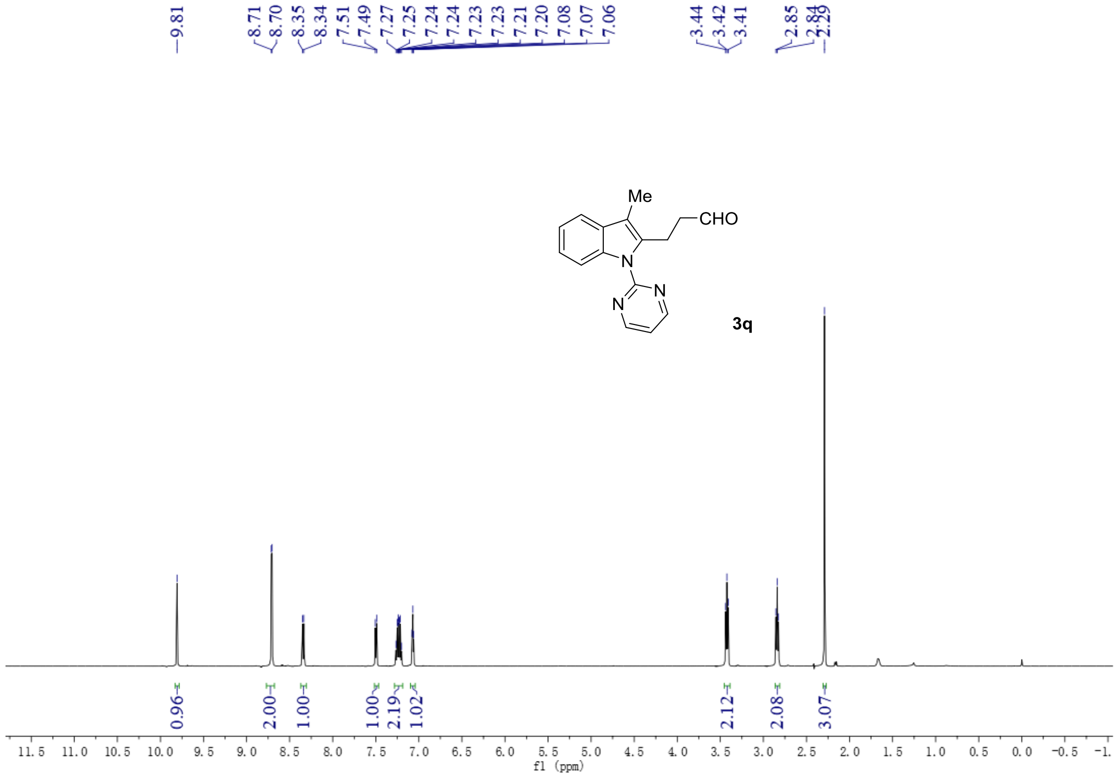

Supplementary Fig. 196 | ^{1}H NMR (500 MHz) of compound 3q (using $CDCl_3$ as solvent)

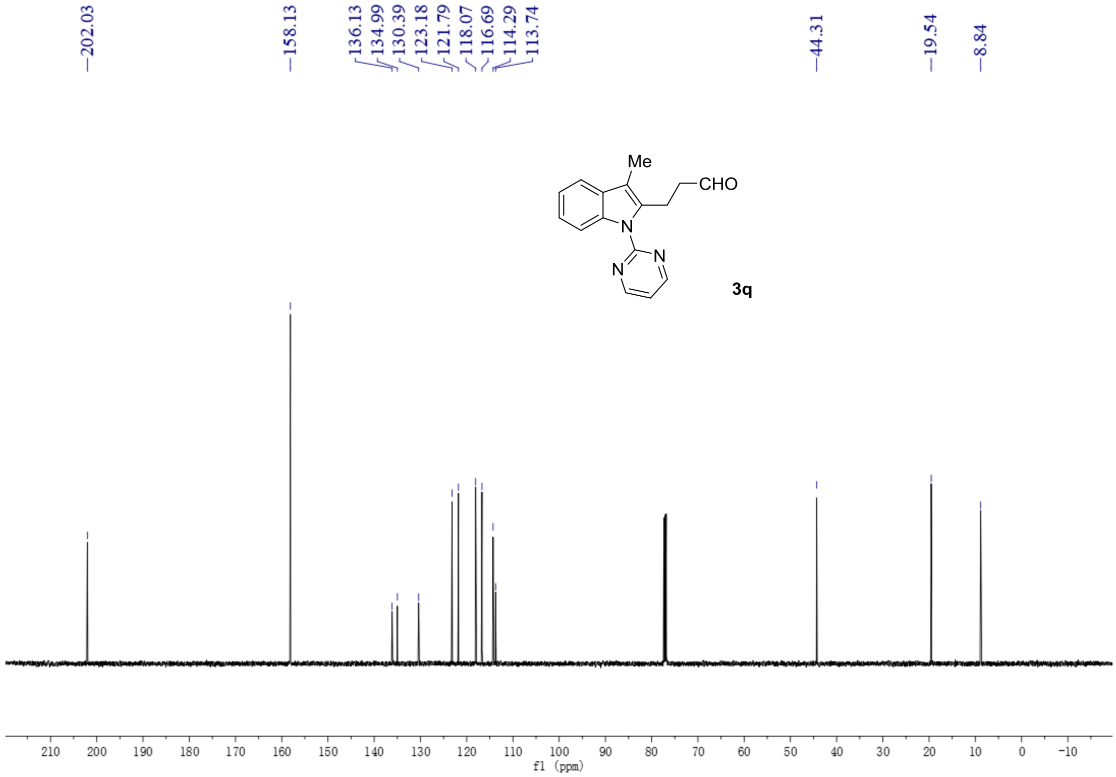

Supplementary Fig. 197 | ^{13}C NMR (126 MHz) of compound 3q (using $CDCl_3$ as solvent)

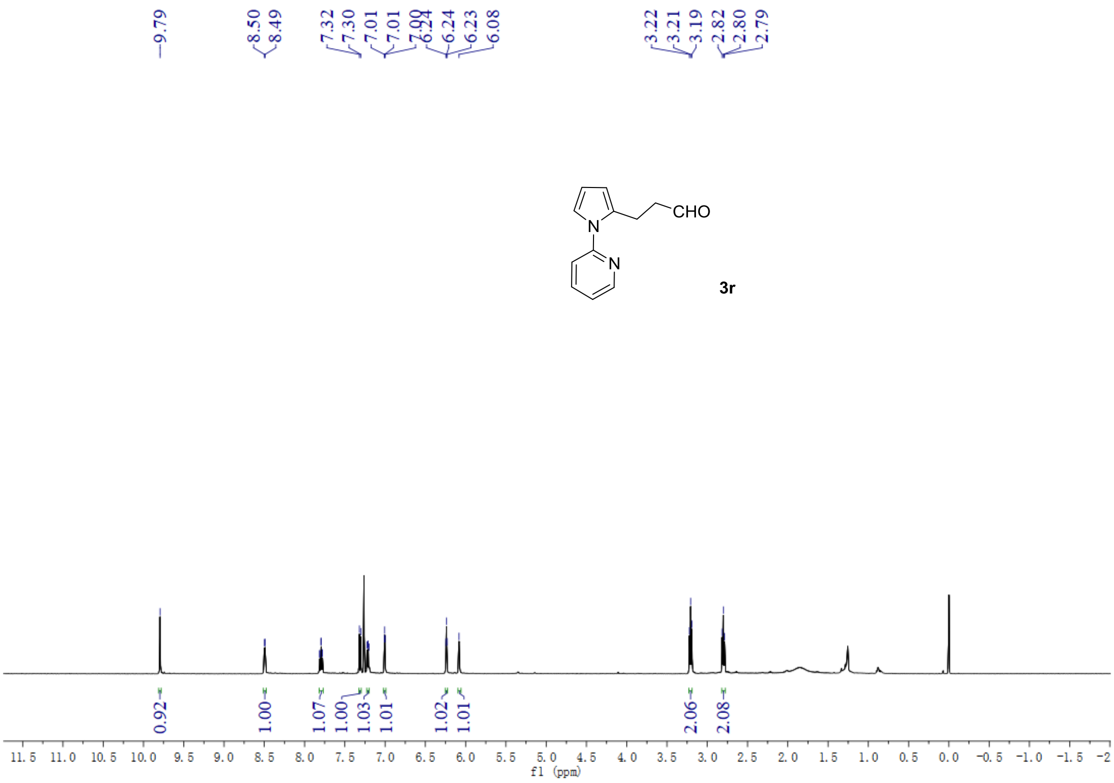

Supplementary Fig. 198 | ^{1}H NMR (500 MHz) of compound 3r (using $CDCl_3$ as solvent)

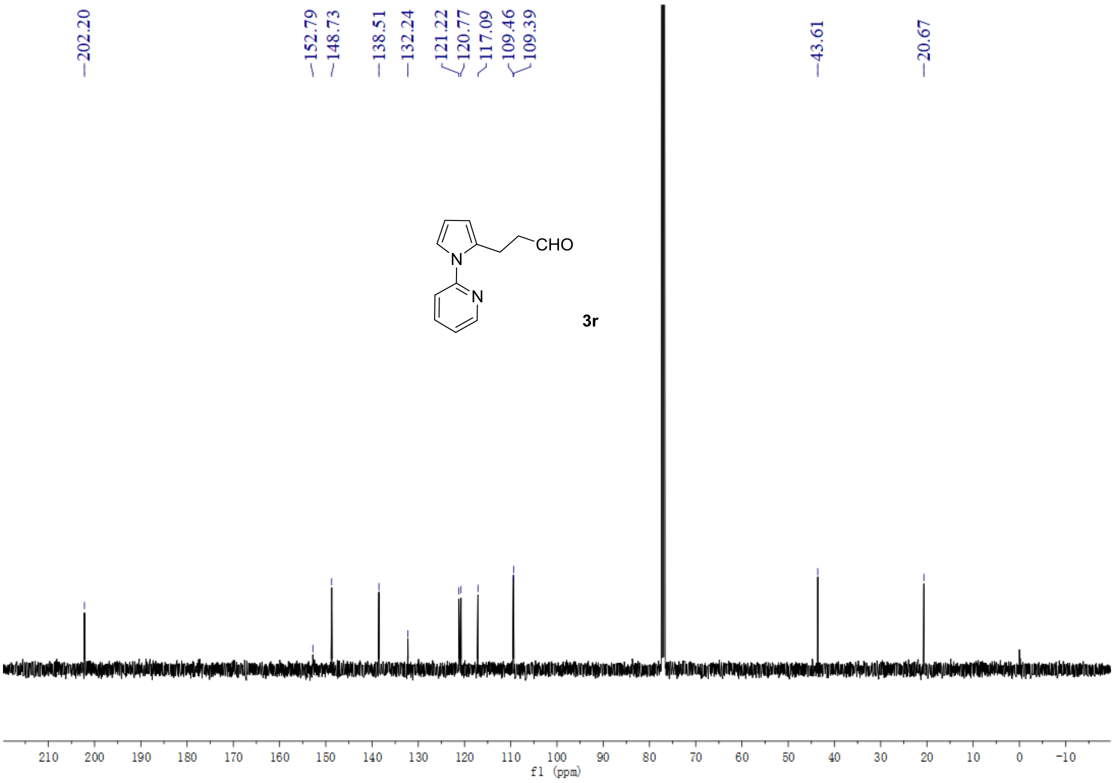

Supplementary Fig. 199 | ^{13}C NMR (126 MHz) of compound 3r (using $CDCl_3$ as solvent)

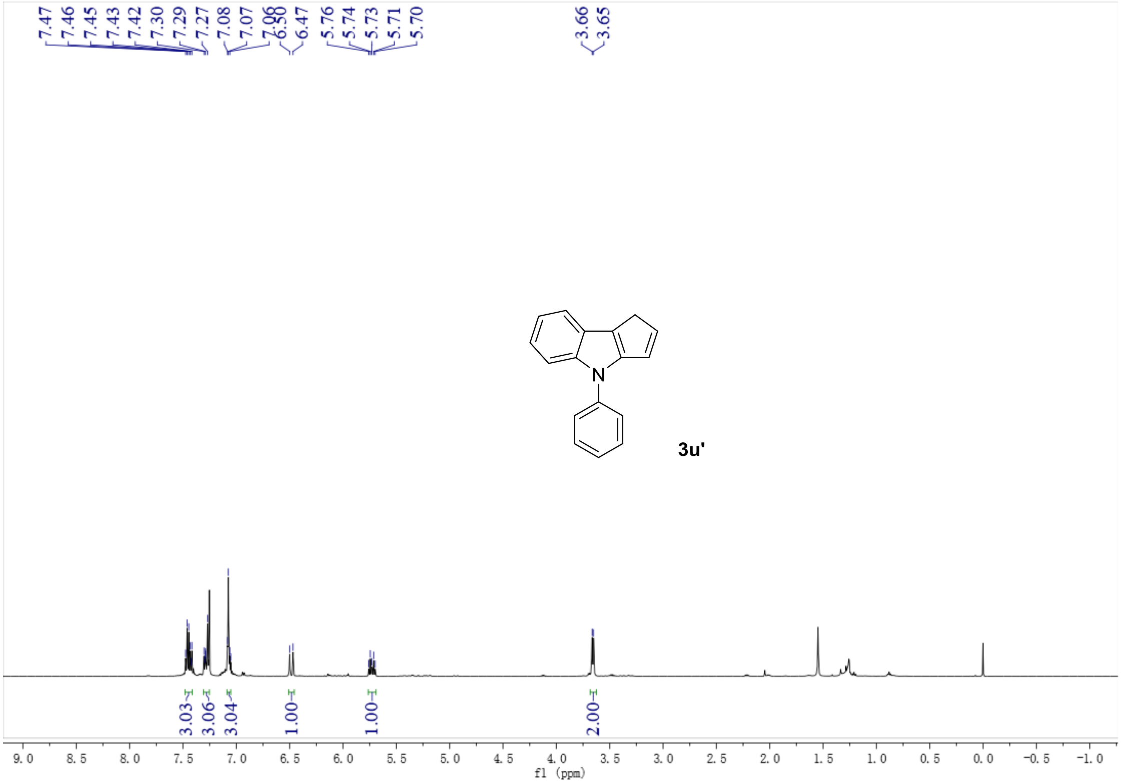

Supplementary Fig. 200 | ^{1}H NMR (500 MHz) of compound 3u′ (using $CDCl_3$ as solvent)

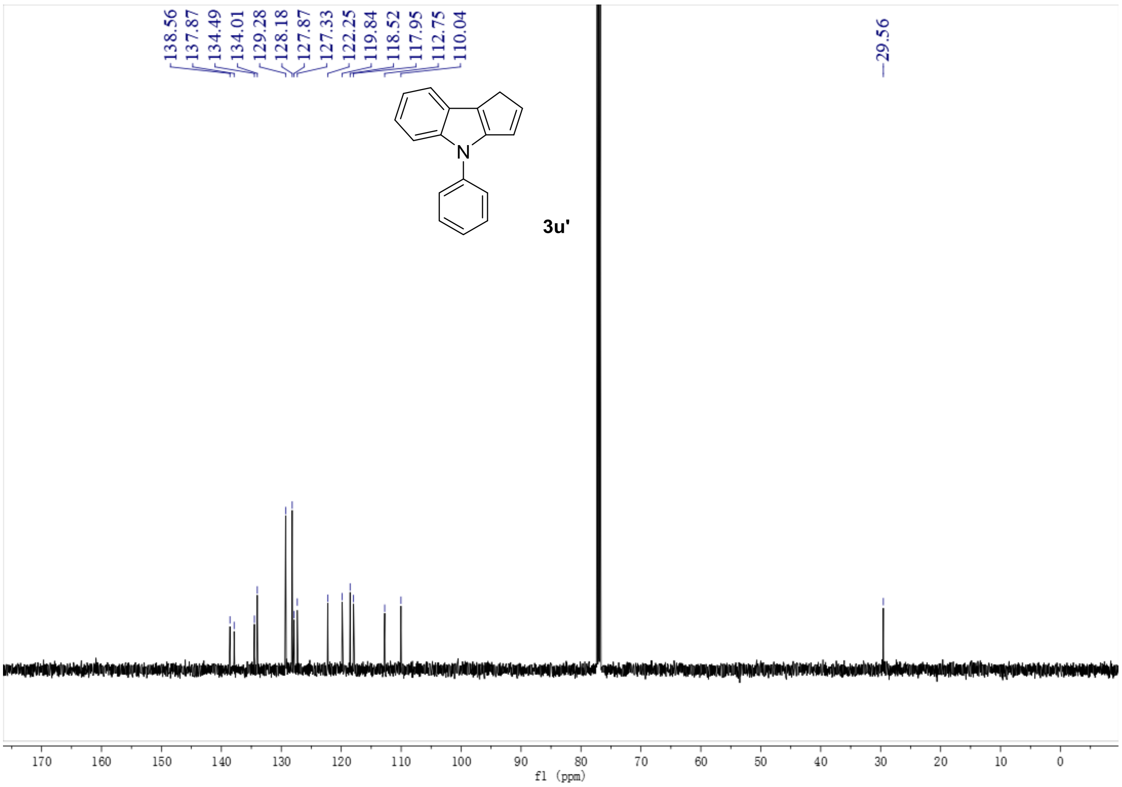

Supplementary Fig. 201 | ^{13}C NMR (126 MHz) of compound 3u′ (using $CDCl_3$ as solvent)

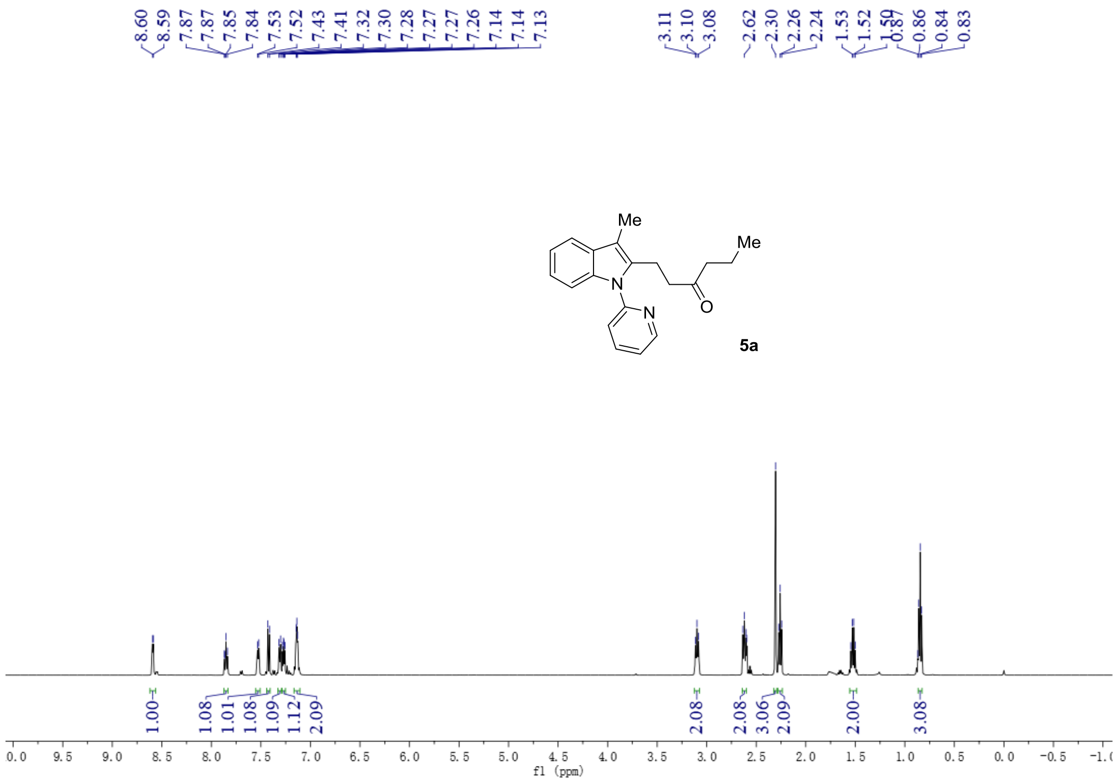

Supplementary Fig. 202 | ^{1}H NMR (500 MHz) of compound 5a (using $CDCl_3$ as solvent)

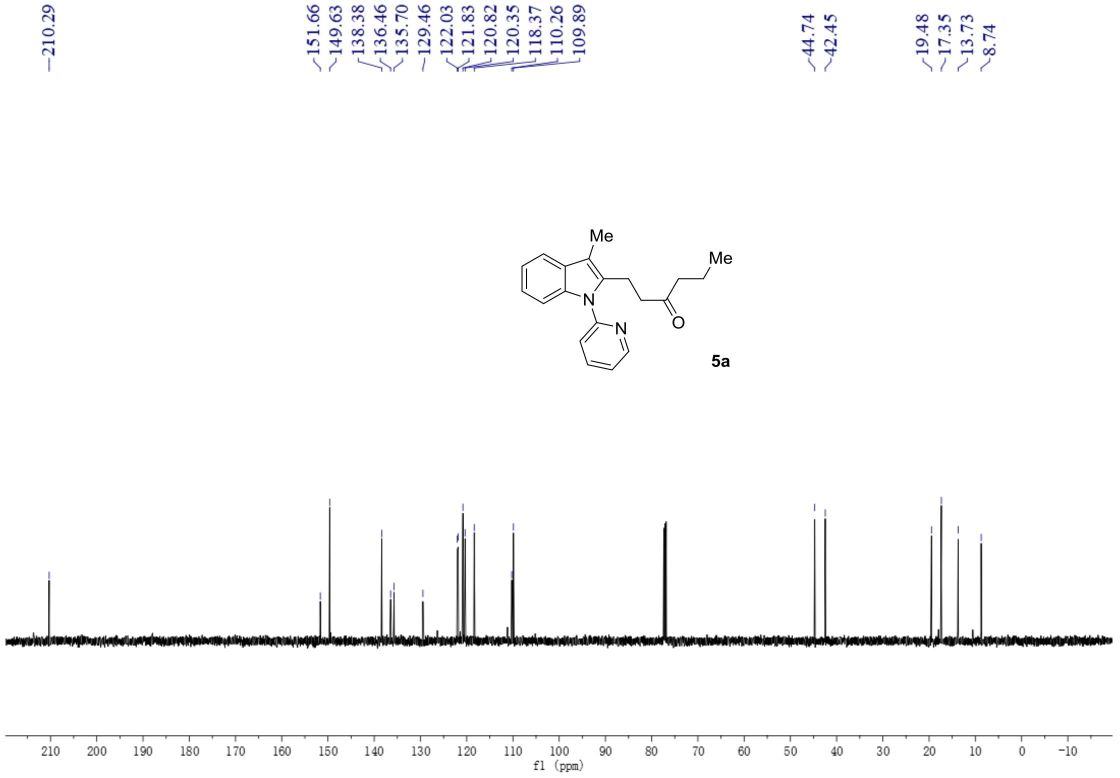

Supplementary Fig. 203 | ^{13}C NMR (126 MHz) of compound 5a (using $CDCl_3$ as solvent)

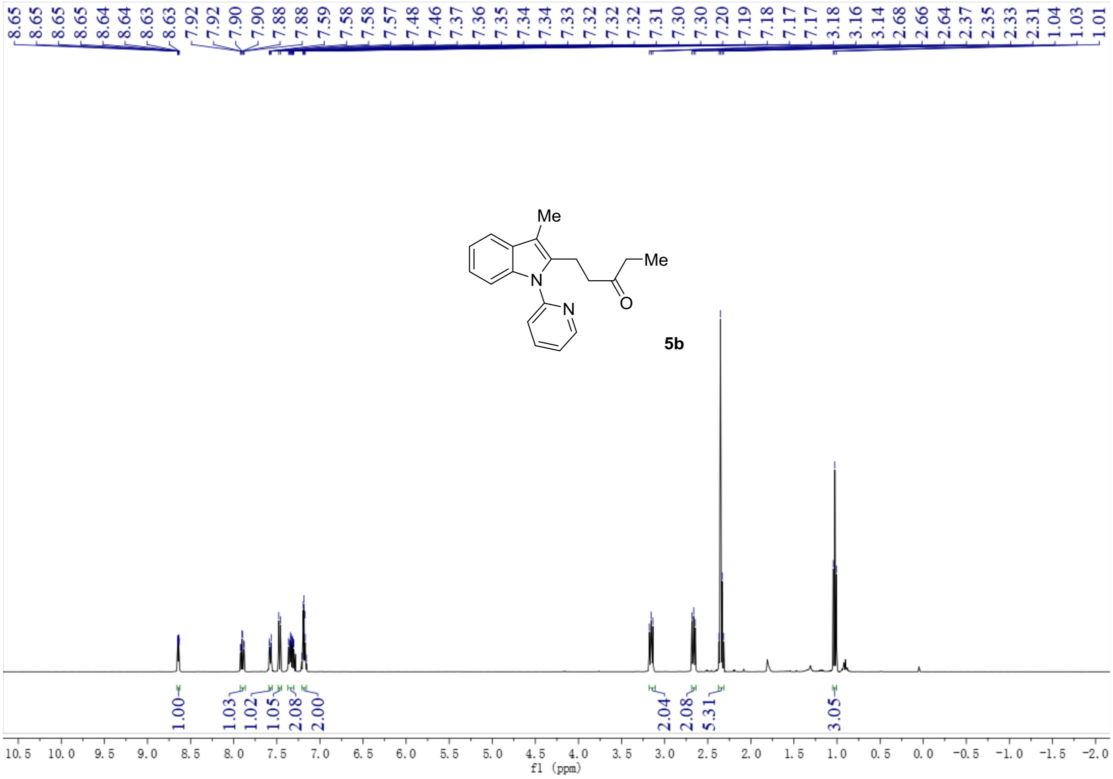

Supplementary Fig. 204 | ^{1}H NMR (400 MHz) of compound 5b (using $CDCl_3$ as solvent)

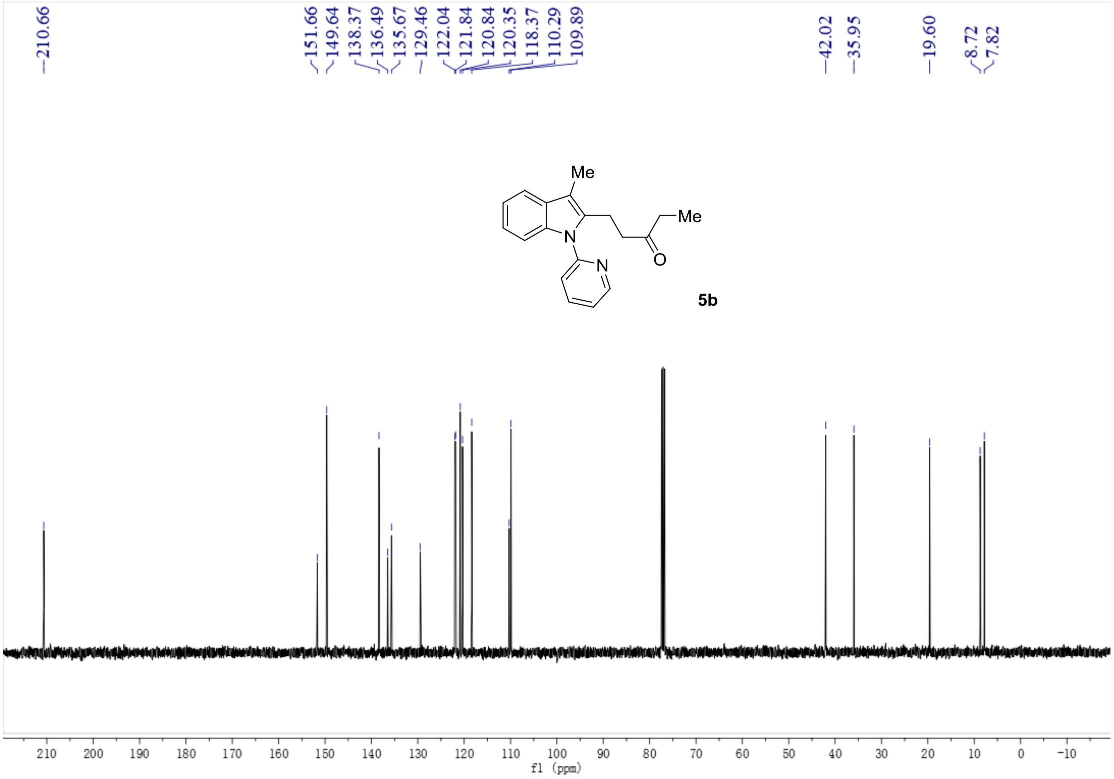

Supplementary Fig. 205 | ^{13}C NMR (101 MHz) of compound 5b (using $CDCl_3$ as solvent)

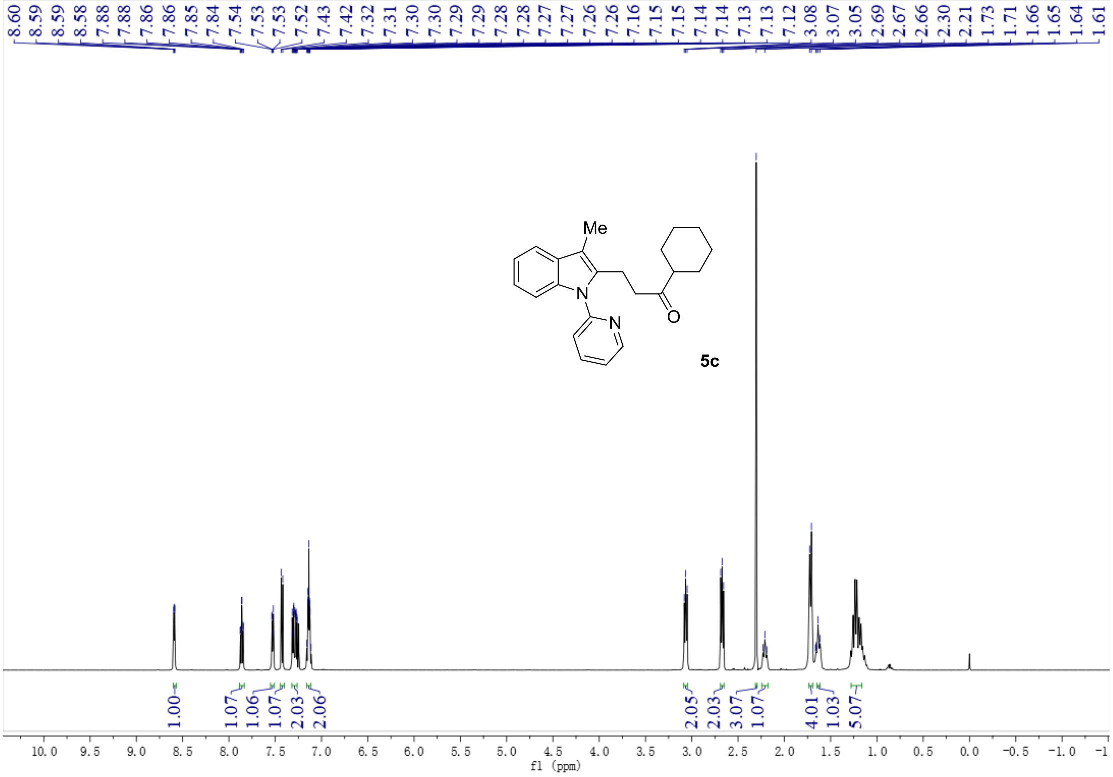

Supplementary Fig. 206 | ^{1}H NMR (500 MHz) of compound 5c (using $CDCl_3$ as solvent)

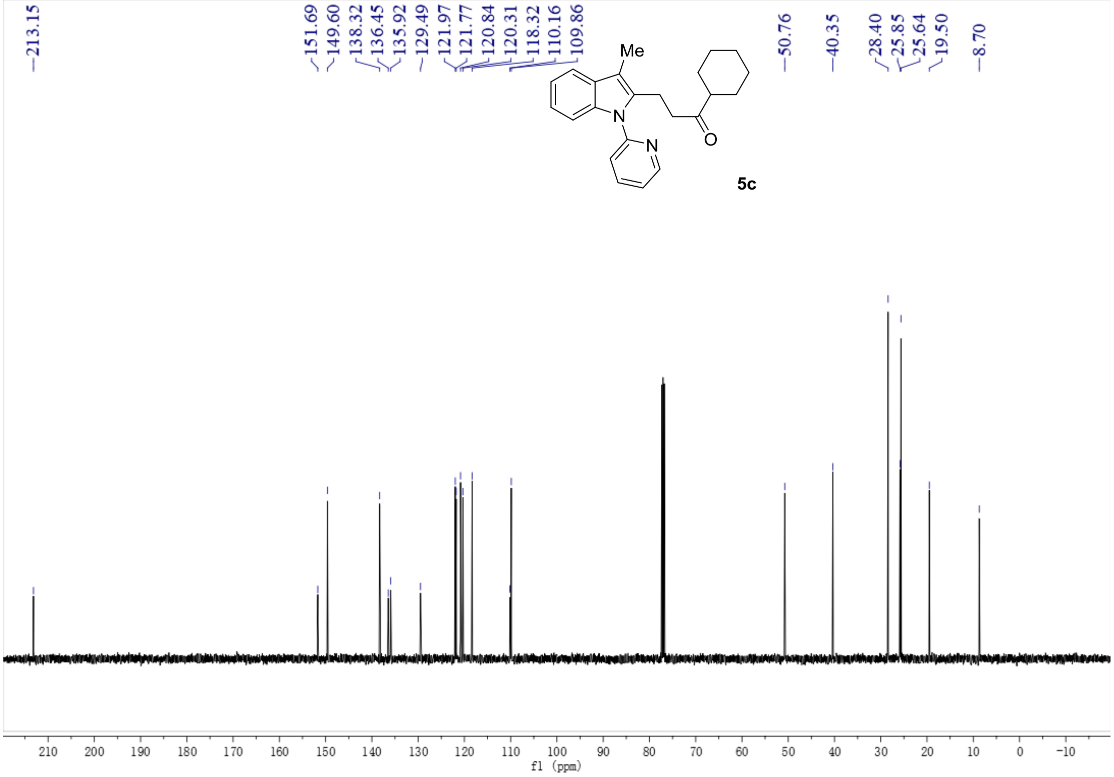

Supplementary Fig. 207 | ^{13}C NMR (126 MHz) of compound 5c (using $CDCl_3$ as solvent)

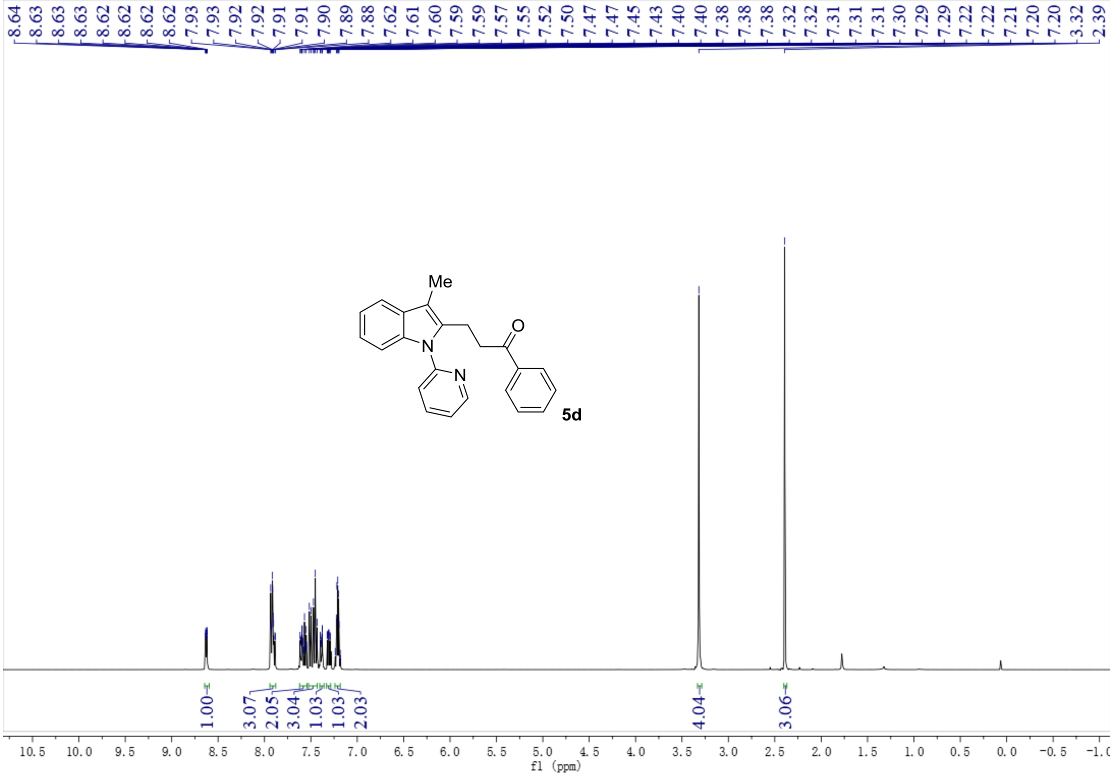

Supplementary Fig. 208 | ^{1}H NMR (400 MHz) of compound 5d (using $CDCl_3$ as solvent)

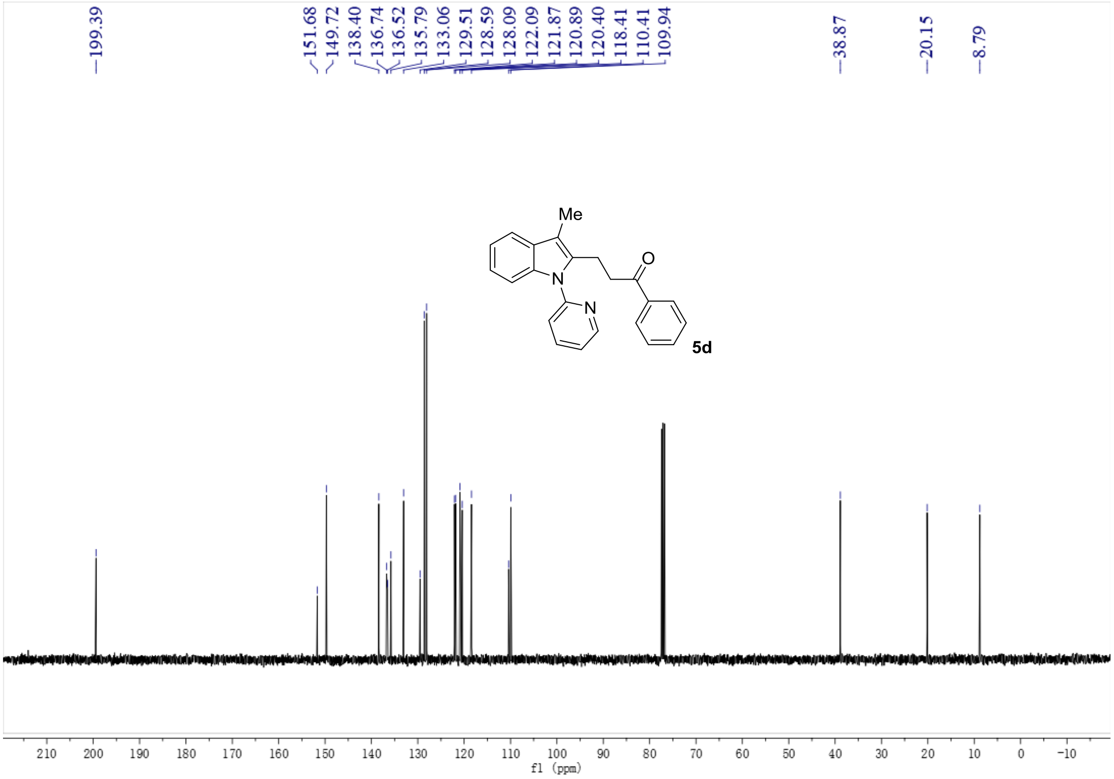

Supplementary Fig. 209 | ^{13}C NMR (101 MHz) of compound 5d (using $CDCl_3$ as solvent)

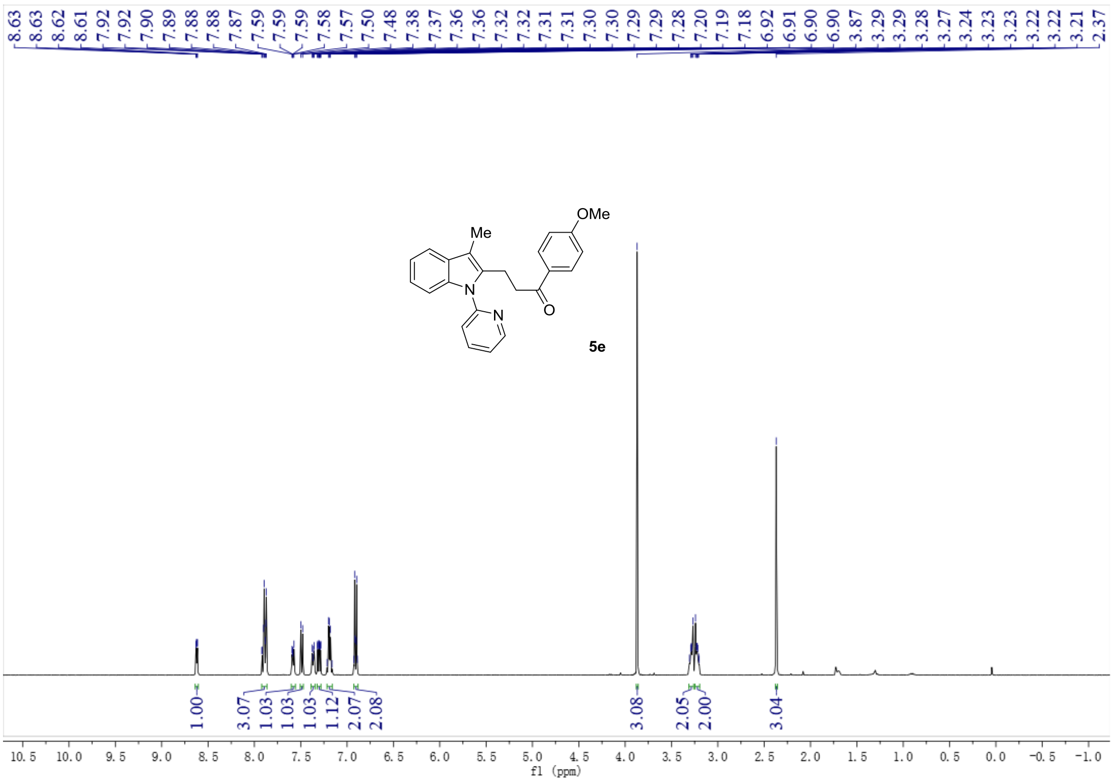

Supplementary Fig. 210 | ^{1}H NMR (400 MHz) of compound 5e (using $CDCl_3$ as solvent)

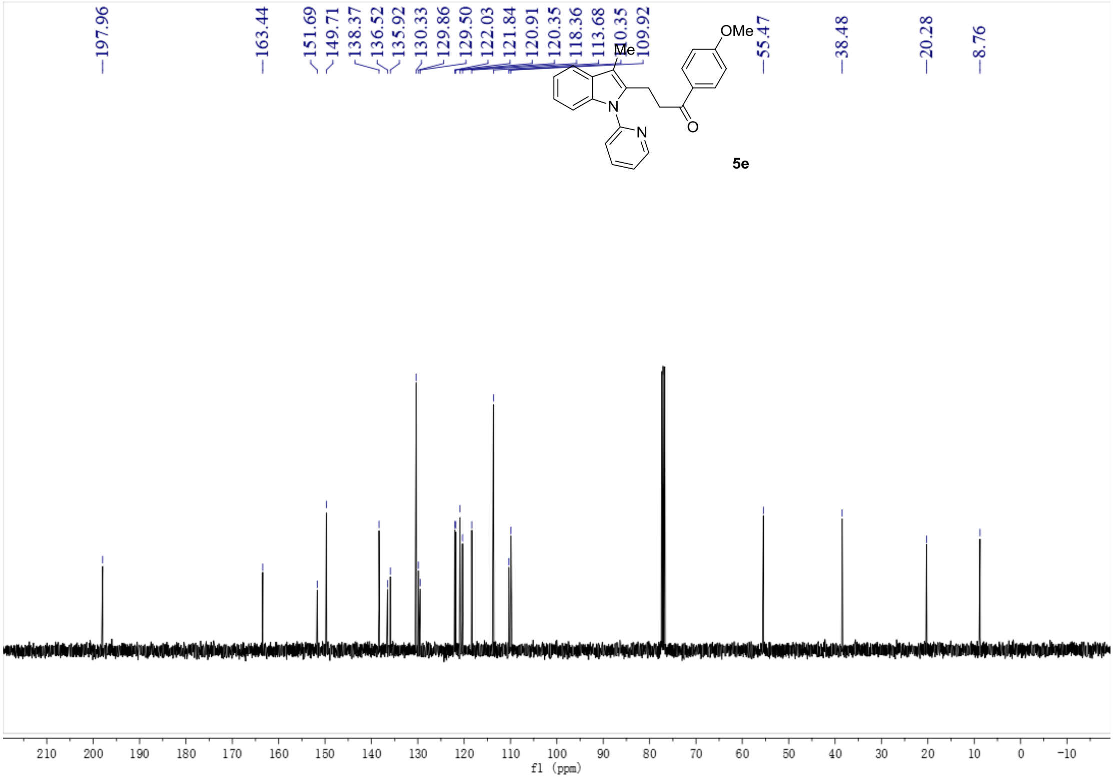

Supplementary Fig. 211 | ^{13}C NMR (101 MHz) of compound 5e (using $CDCl_3$ as solvent)

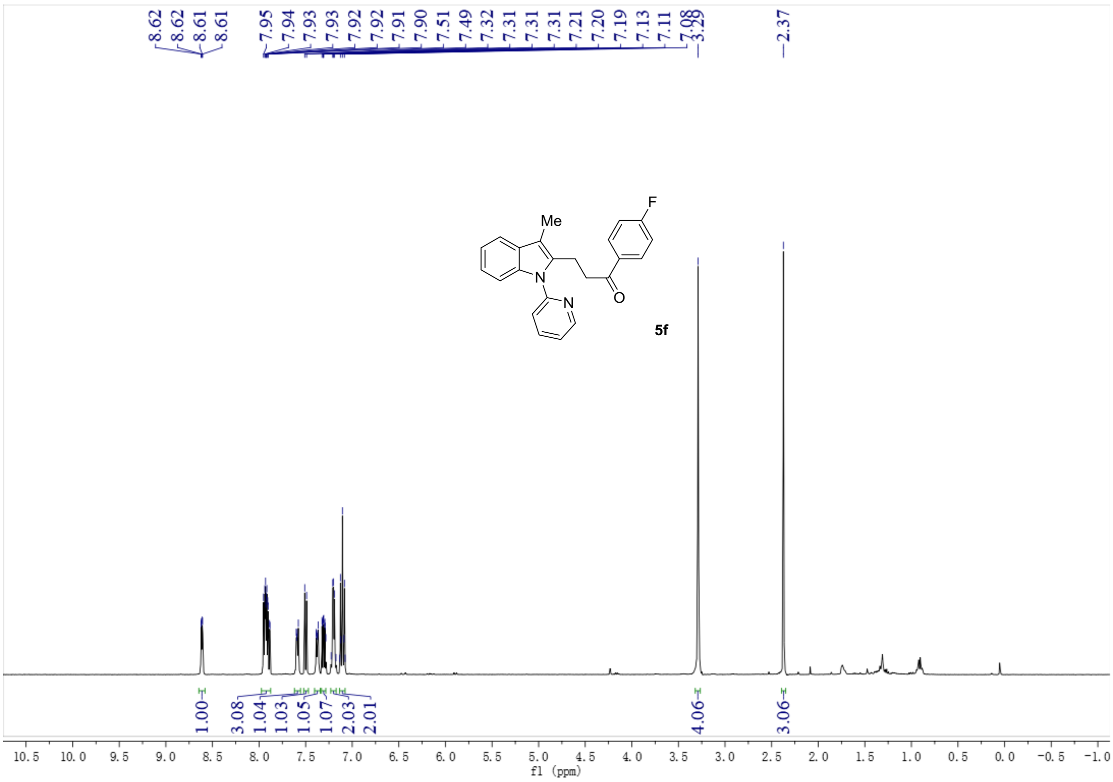

Supplementary Fig. 212 | ^{1}H NMR (400 MHz) of compound 5f (using $CDCl_3$ as solvent)

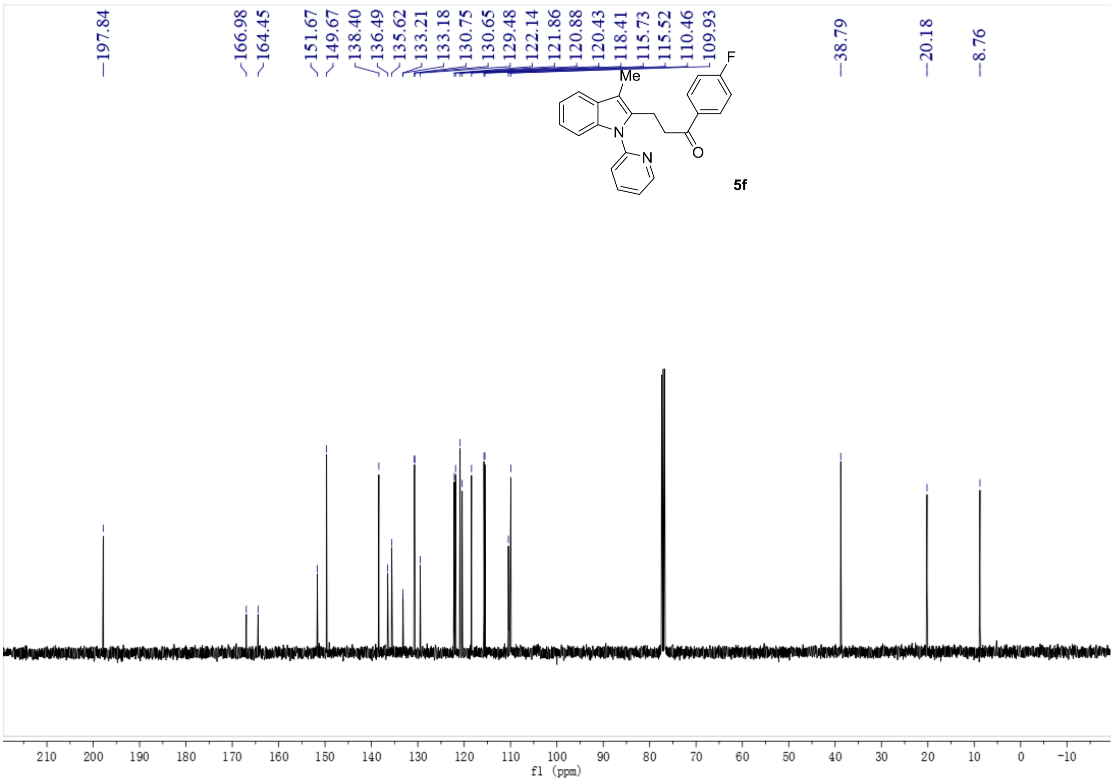

Supplementary Fig. 213 | ^{13}C NMR (101 MHz) of compound 5f (using $CDCl_3$ as solvent)

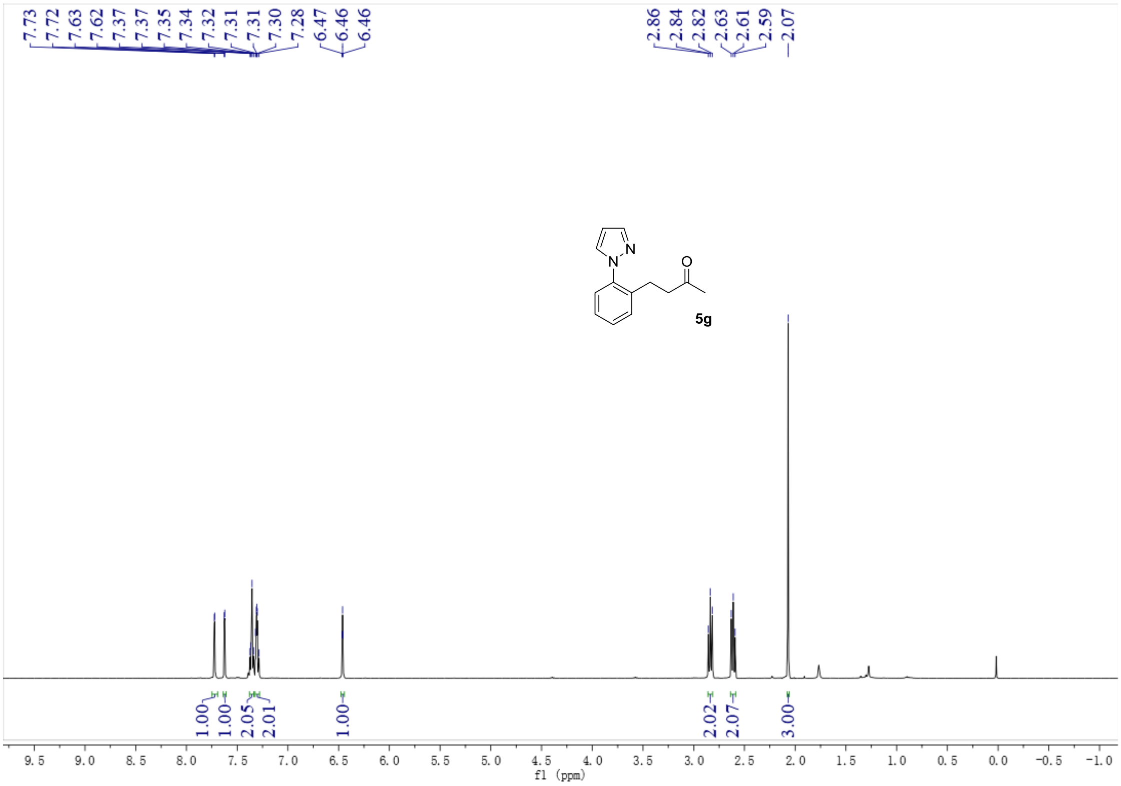

Supplementary Fig. 214 | ^{1}H NMR (400 MHz) of compound 5g (using $CDCl_3$ as solvent)

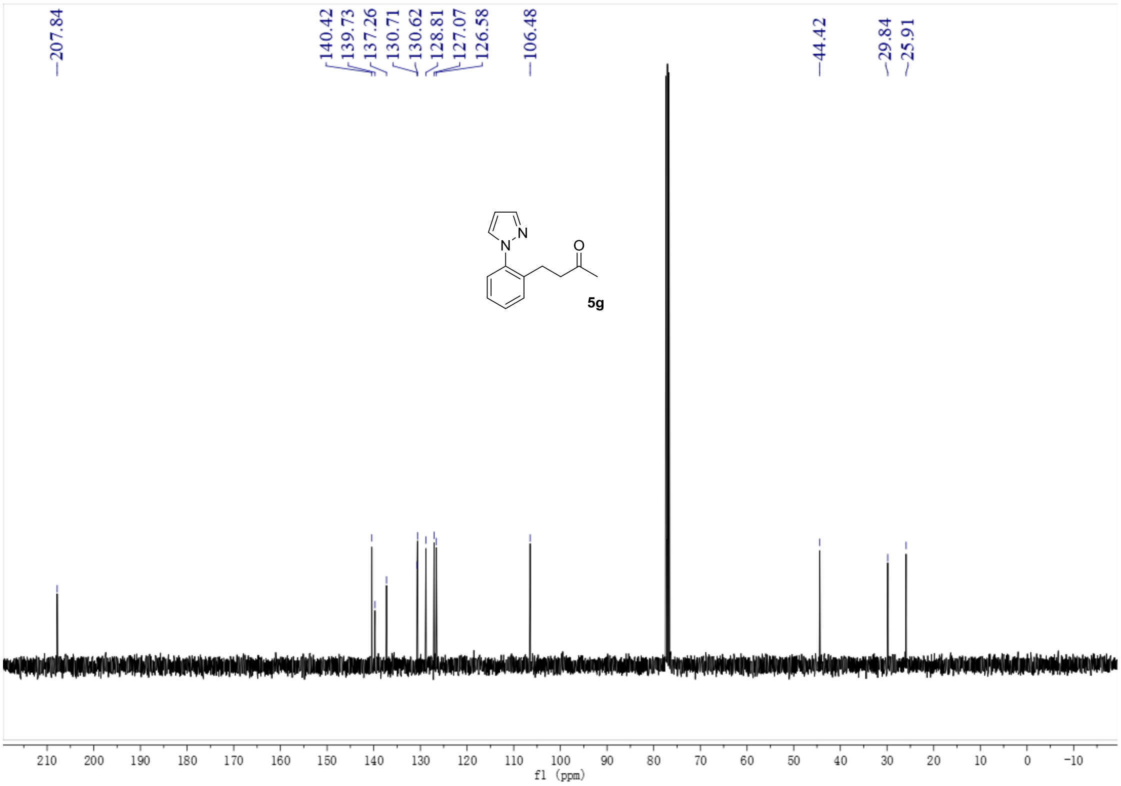

Supplementary Fig. 215 | ^{13}C NMR (101 MHz) of compound 5g (using $CDCl_3$ as solvent)

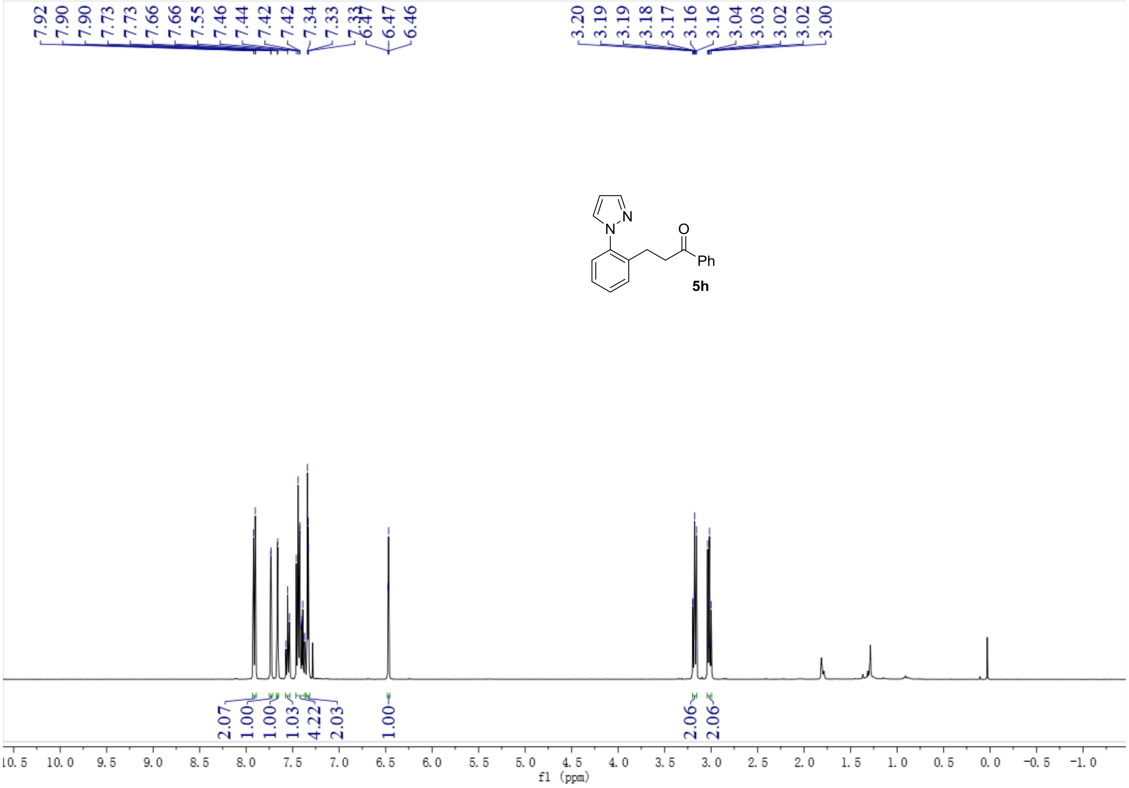

Supplementary Fig. 216 | ^{1}H NMR (400 MHz) of compound 5h (using $CDCl_3$ as solvent)

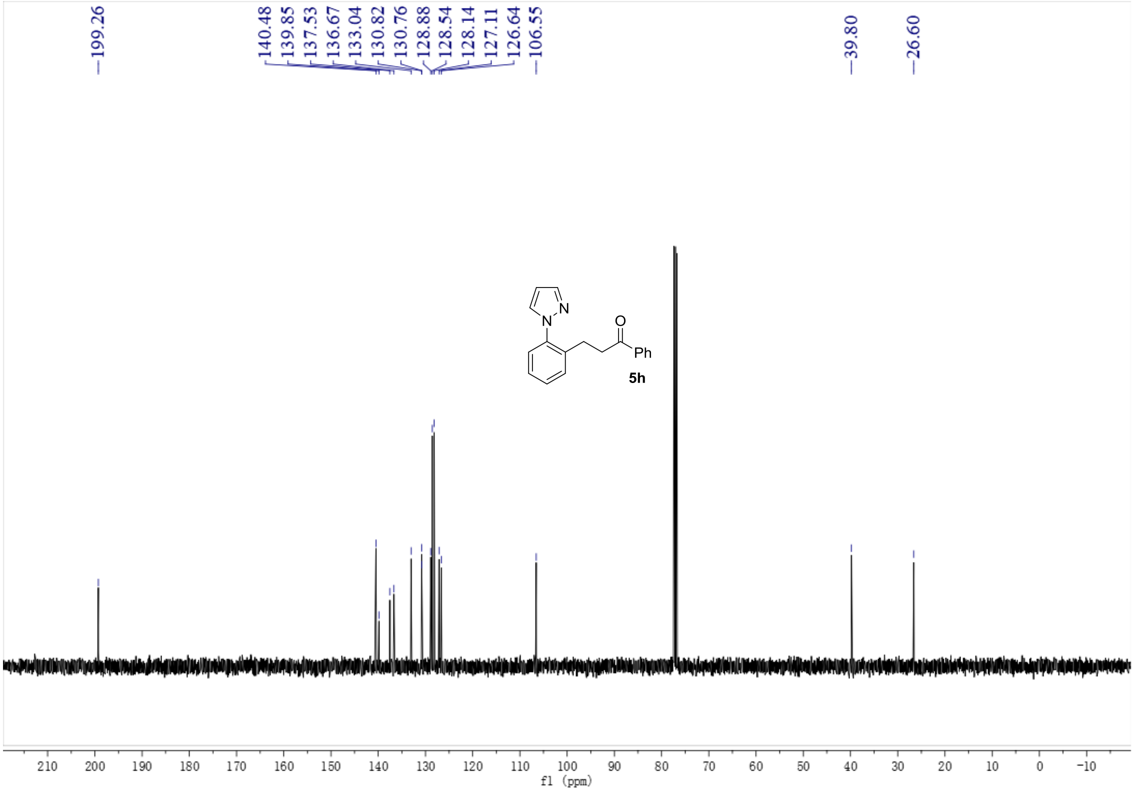

Supplementary Fig. 217 | ^{13}C NMR (101 MHz) of compound 5h (using $CDCl_3$ as solvent)

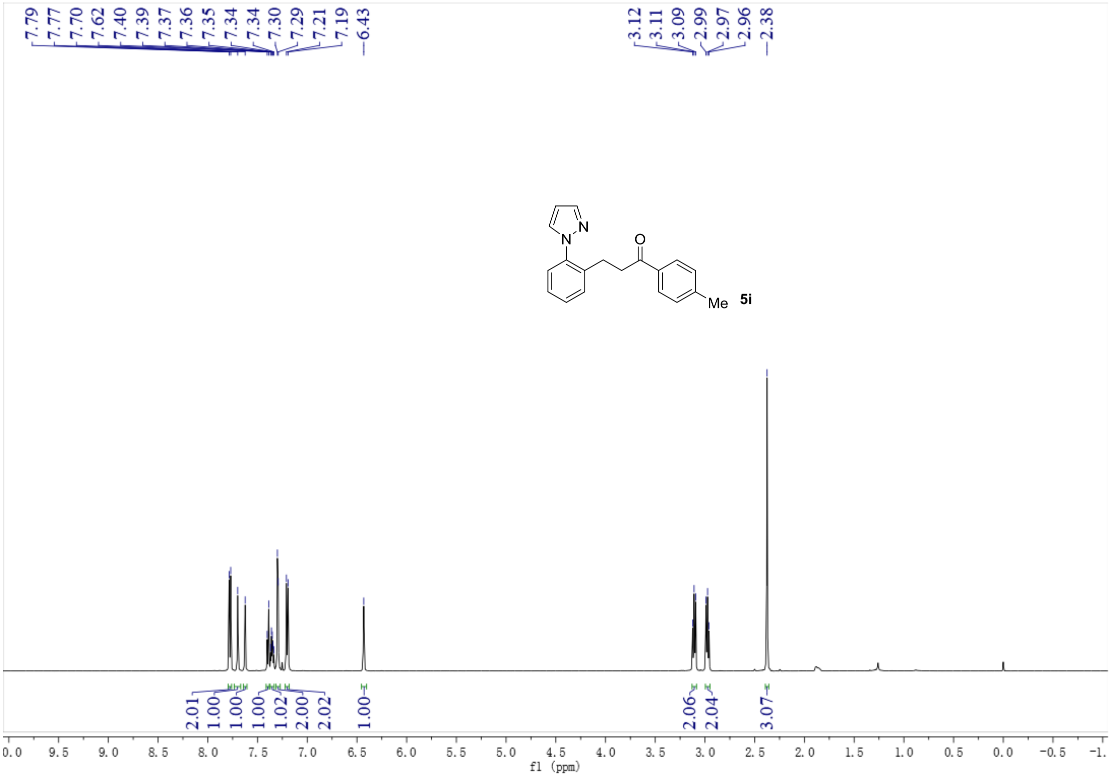

Supplementary Fig. 218 | ^{1}H NMR (500 MHz) of compound 5i (using $CDCl_3$ as solvent)

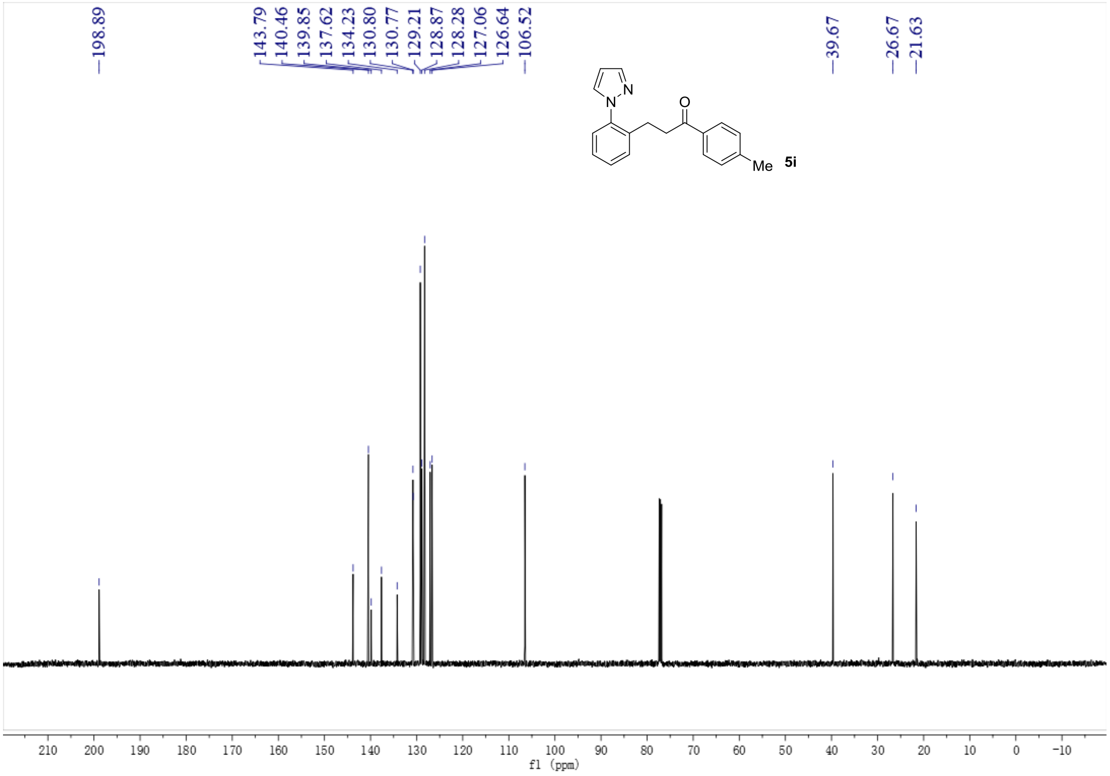

Supplementary Fig. 219 | ^{13}C NMR (126 MHz) of compound 5i (using $CDCl_3$ as solvent)

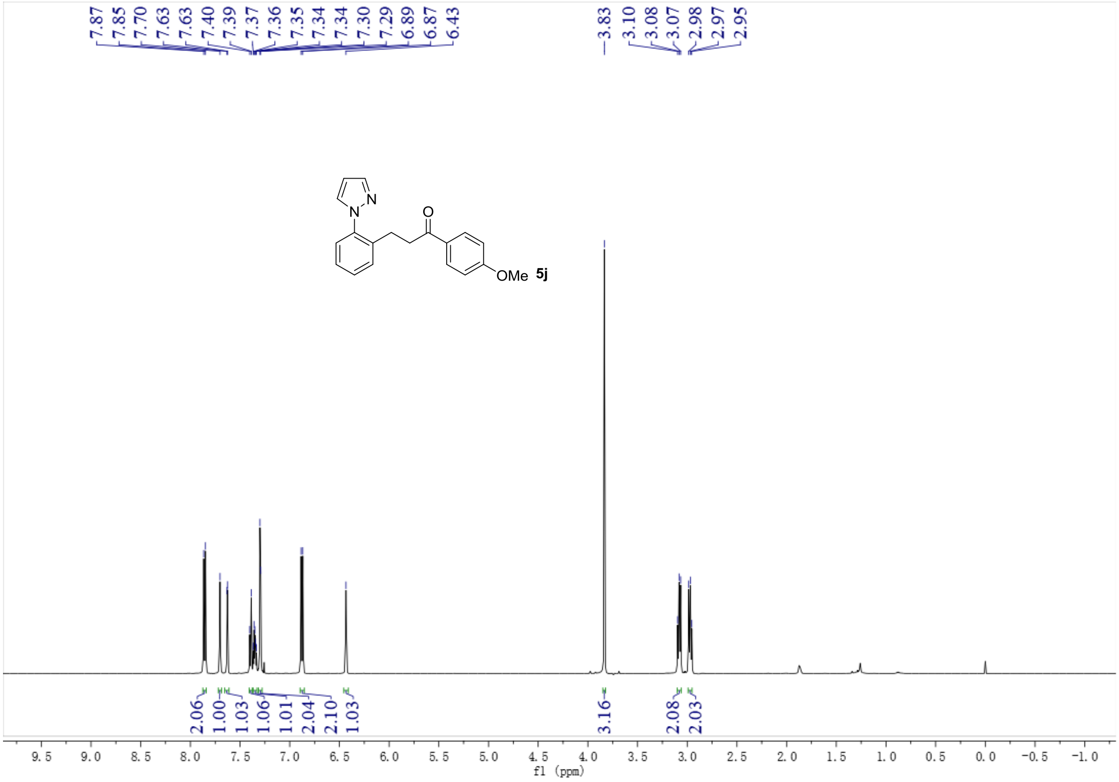

Supplementary Fig. 220 | ^{1}H NMR (500 MHz) of compound 5j (using $CDCl_3$ as solvent)

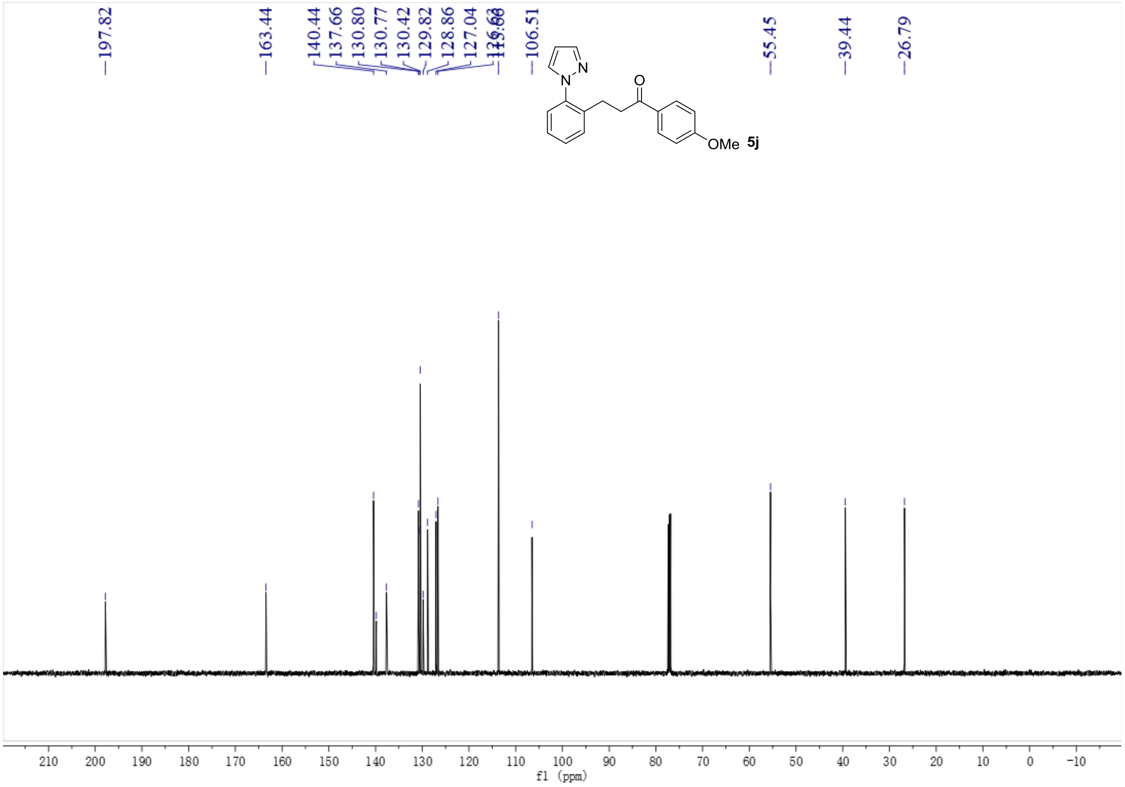

Supplementary Fig. 221 | ^{13}C NMR (126 MHz) of compound 5j (using $CDCl_3$ as solvent)

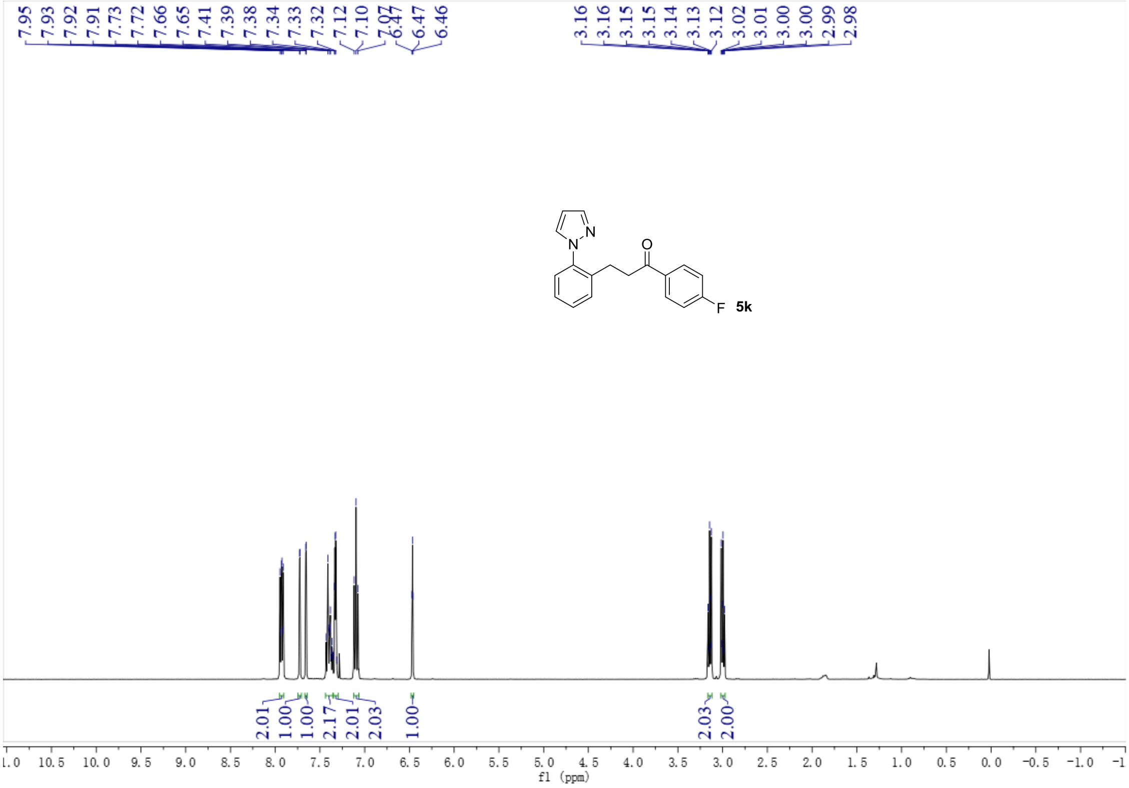

Supplementary Fig. 222 | ^{1}H NMR (400 MHz) of compound 5k (using $CDCl_3$ as solvent)

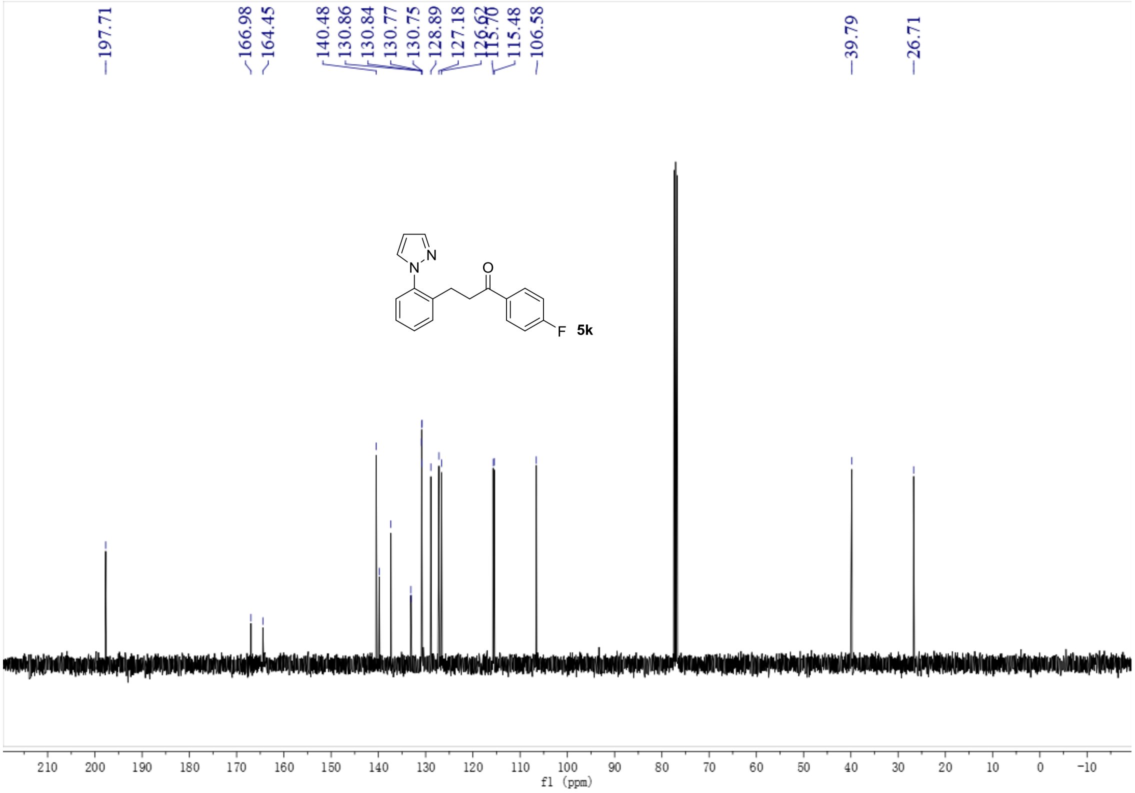

Supplementary Fig. 223 | ^{13}C NMR (101 MHz) of compound 5k (using $CDCl_3$ as solvent)

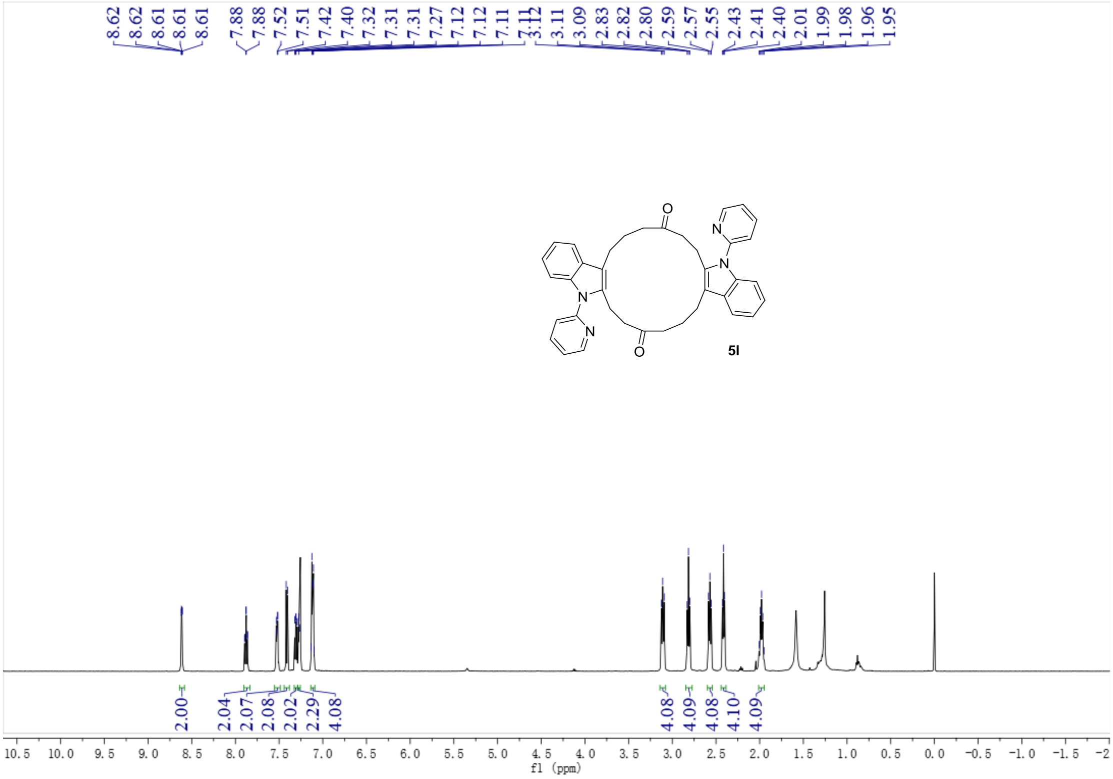

Supplementary Fig. 224 | ^{1}H NMR (500 MHz) of compound 5l (using $CDCl_3$ as solvent)

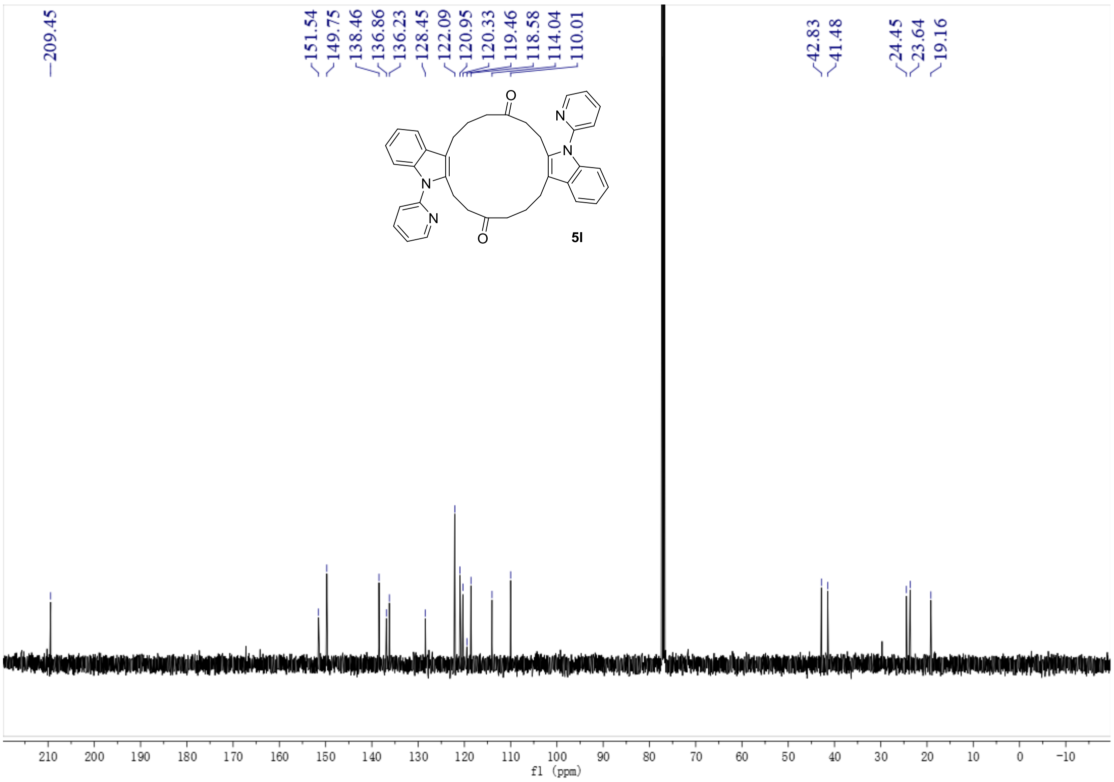

Supplementary Fig. 225 | ^{13}C NMR (126 MHz) of compound 5l (using $CDCl_3$ as solvent)

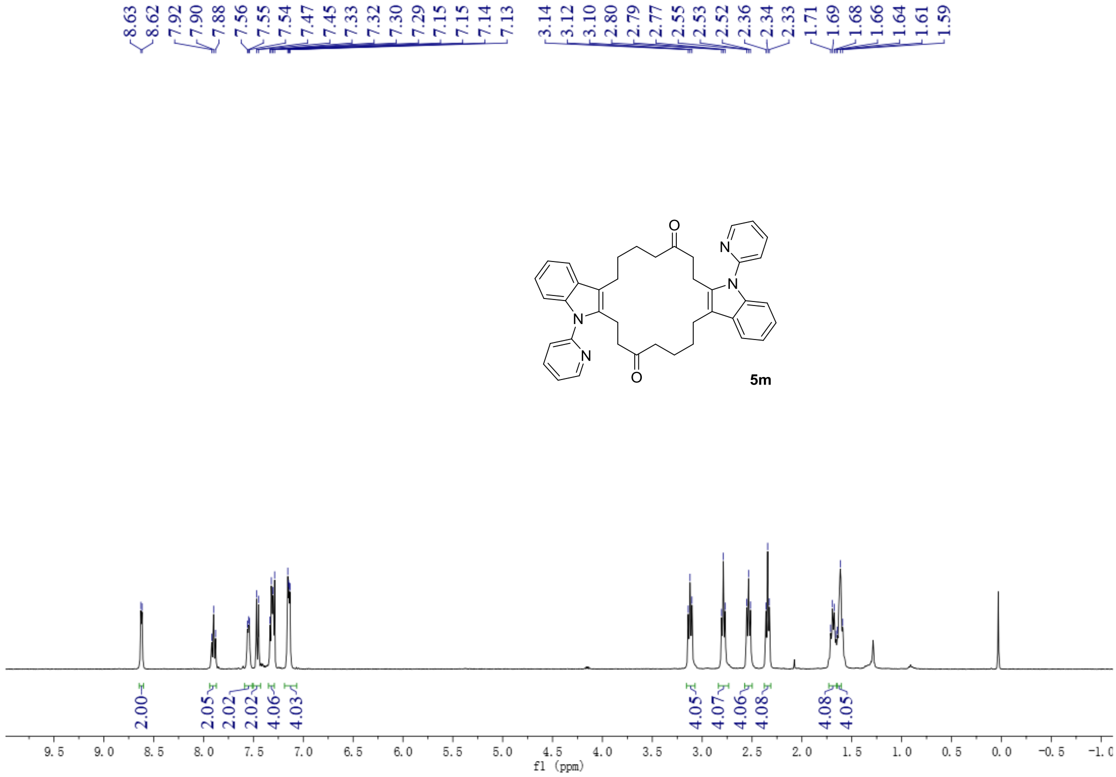

Supplementary Fig. 226 | ^{1}H NMR (400 MHz) of compound 5m (using $CDCl_3$ as solvent)

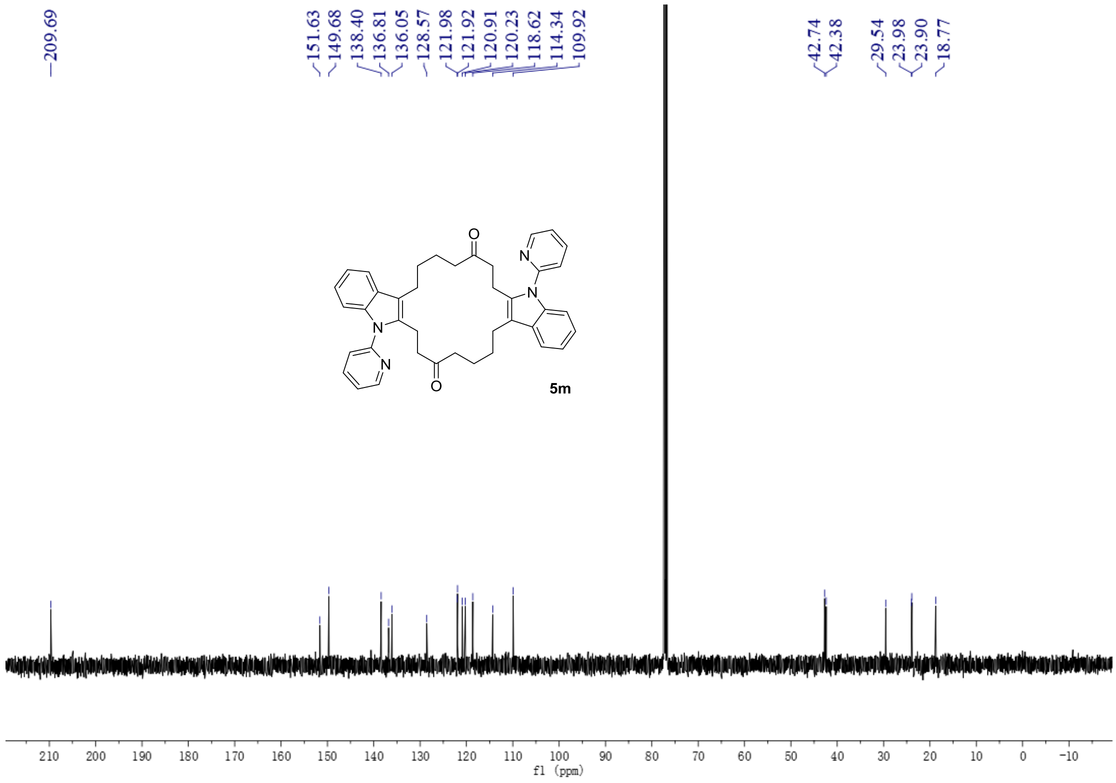

Supplementary Fig. 227 | ^{13}C NMR (101 MHz) of compound 5m (using $CDCl_3$ as solvent)

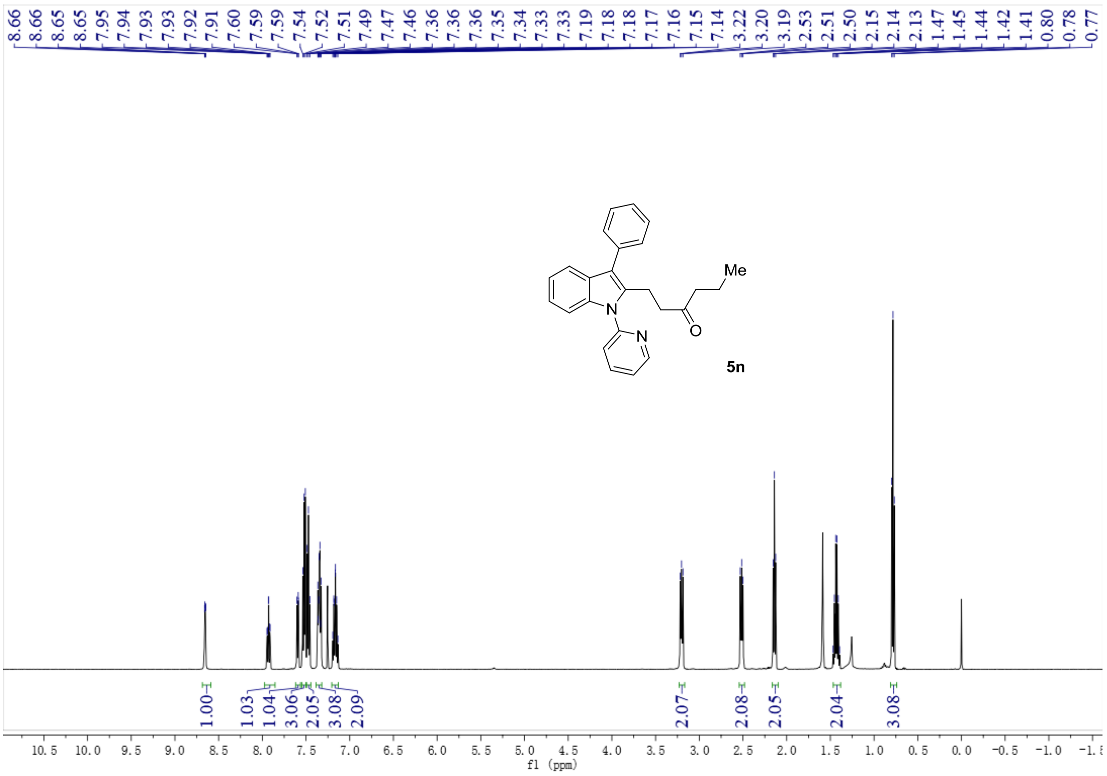

Supplementary Fig. 228 | ^{1}H NMR (500 MHz) of compound 5n (using $CDCl_3$ as solvent)

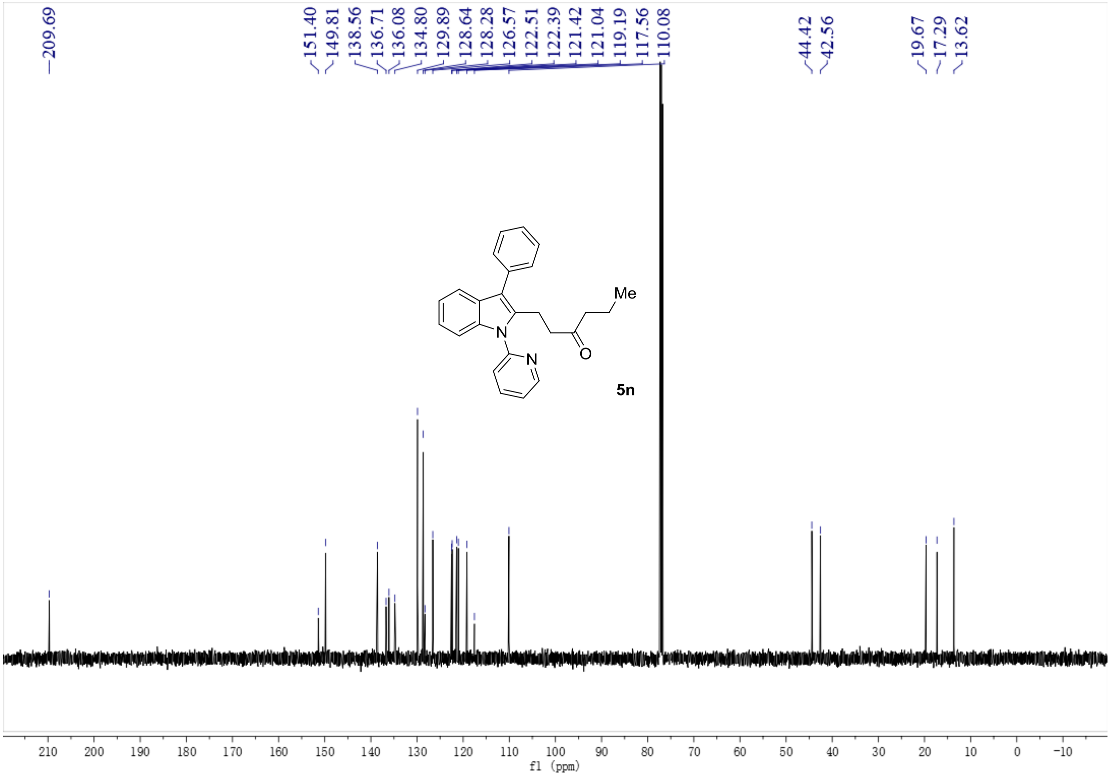

Supplementary Fig. 229 | ^{13}C NMR (126 MHz) of compound 5n (using $CDCl_3$ as solvent)

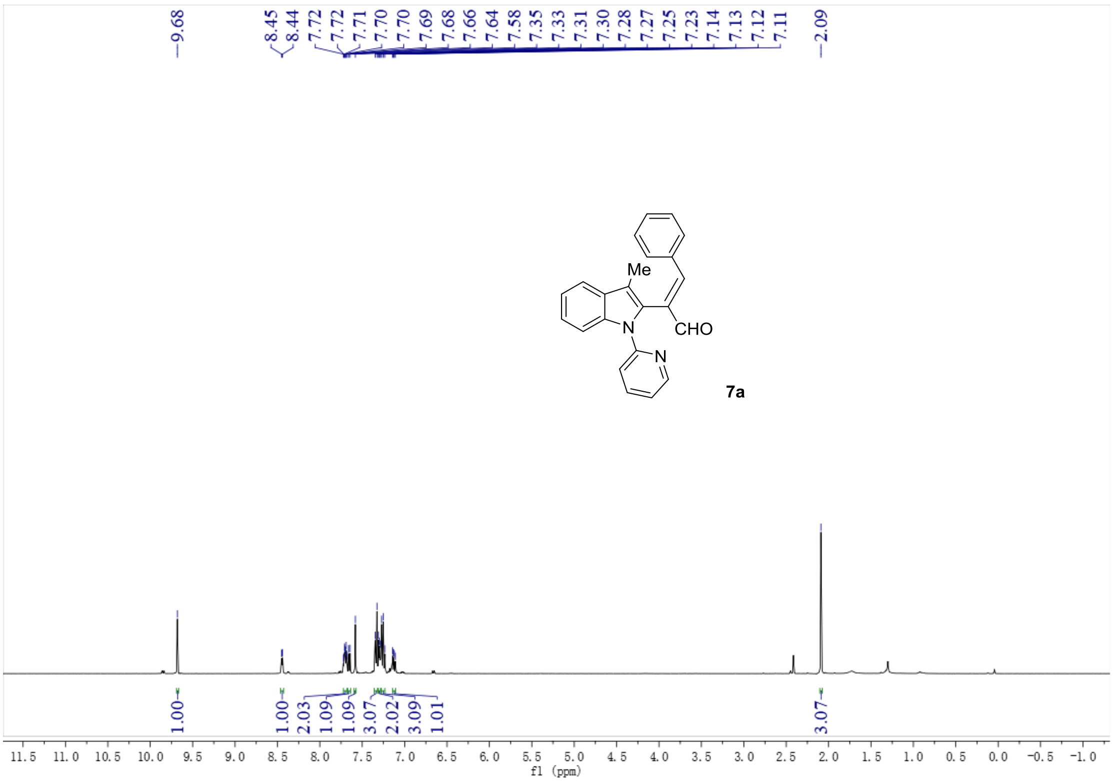

Supplementary Fig. 230 | ^{1}H NMR (400 MHz) of compound 7a (using $CDCl_3$ as solvent)

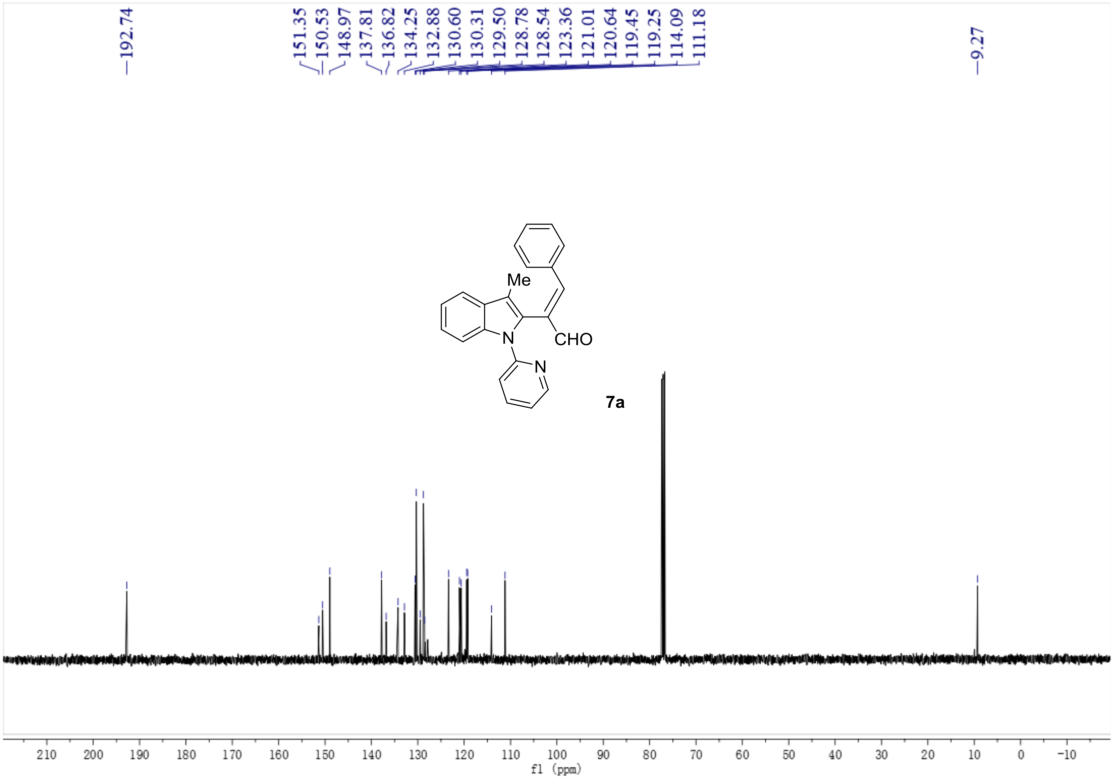

Supplementary Fig. 231 | ^{13}C NMR (101 MHz) of compound 7a (using $CDCl_3$ as solvent)

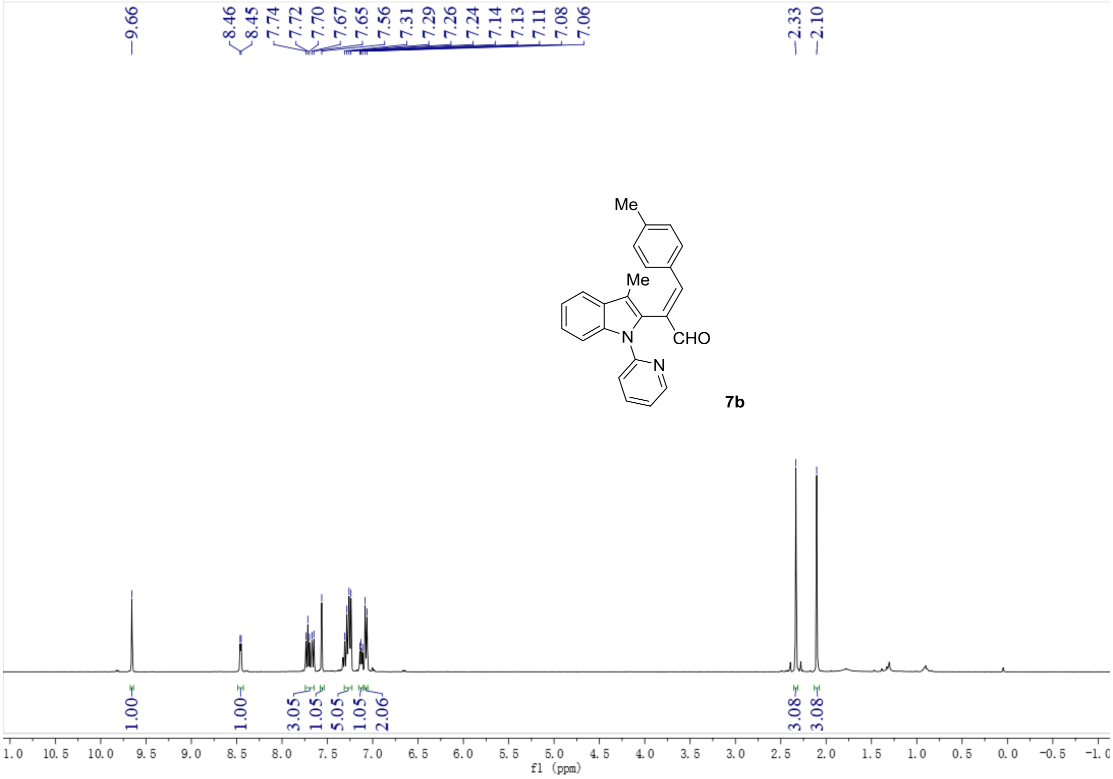

Supplementary Fig. 232 | ^{1}H NMR (400 MHz) of compound 7b (using $CDCl_3$ as solvent)

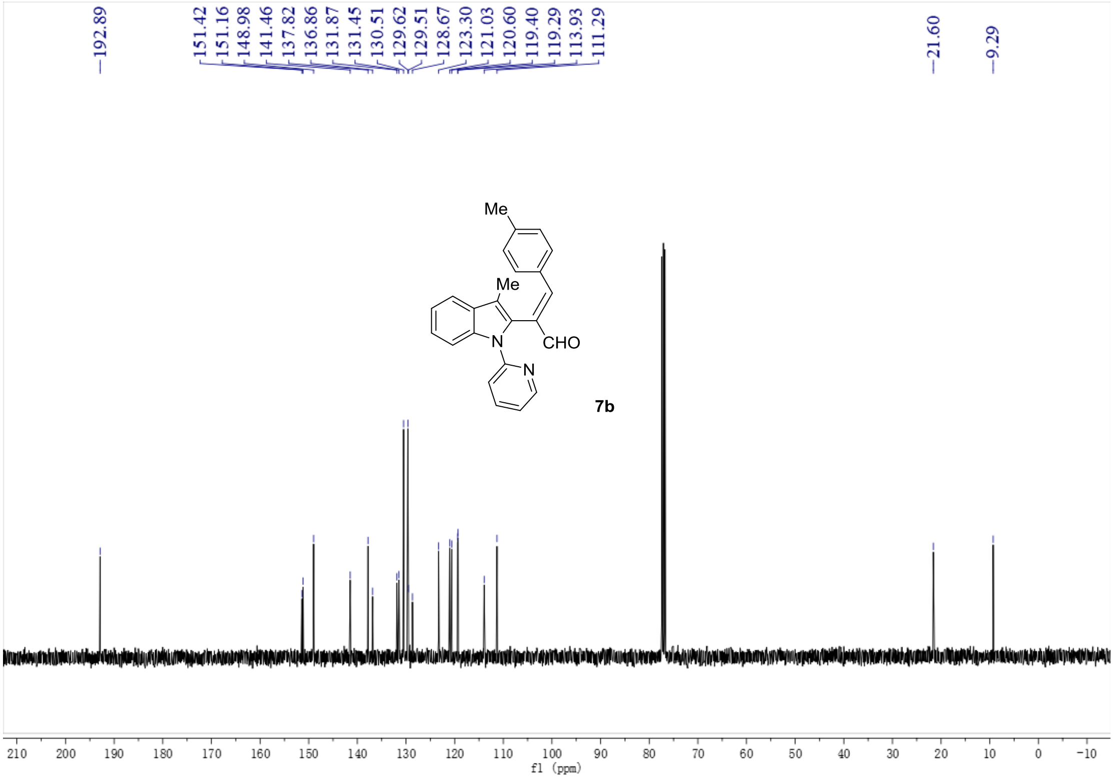

Supplementary Fig. 233 | ^{13}C NMR (101 MHz) of compound 7b (using $CDCl_3$ as solvent)

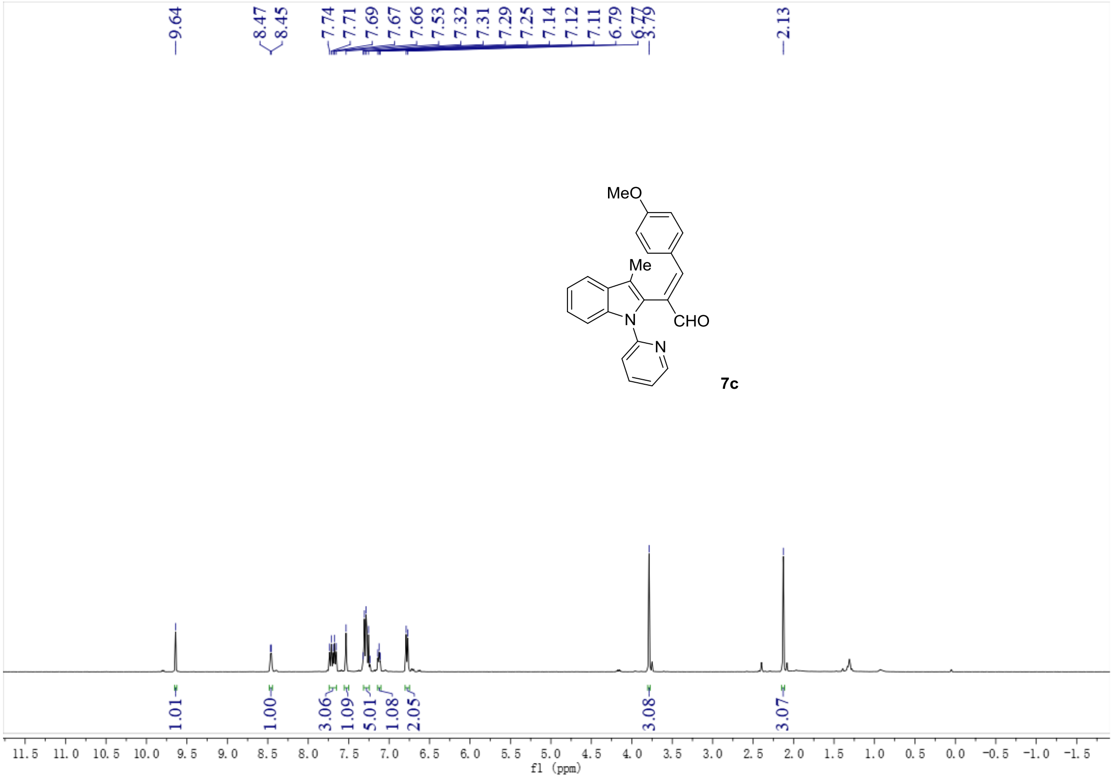

Supplementary Fig. 234 | ^{1}H NMR (400 MHz) of compound 7c (using $CDCl_3$ as solvent)

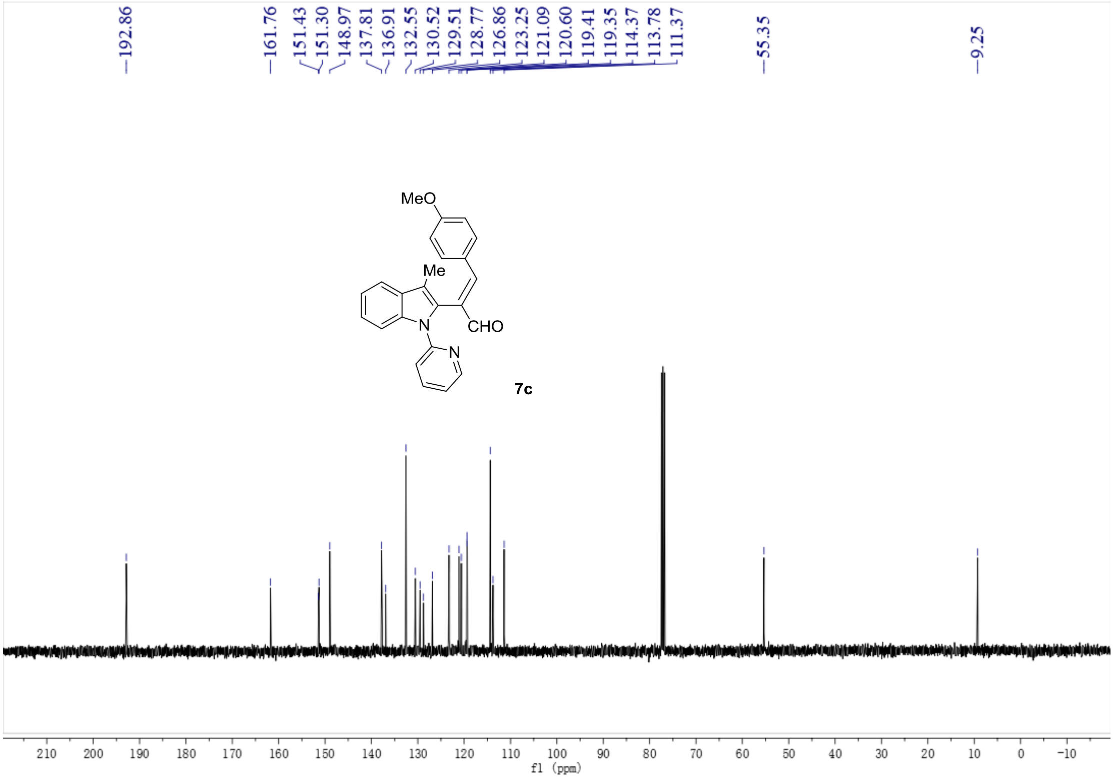

Supplementary Fig. 235 | ^{13}C NMR (101 MHz) of compound 7c (using $CDCl_3$ as solvent)

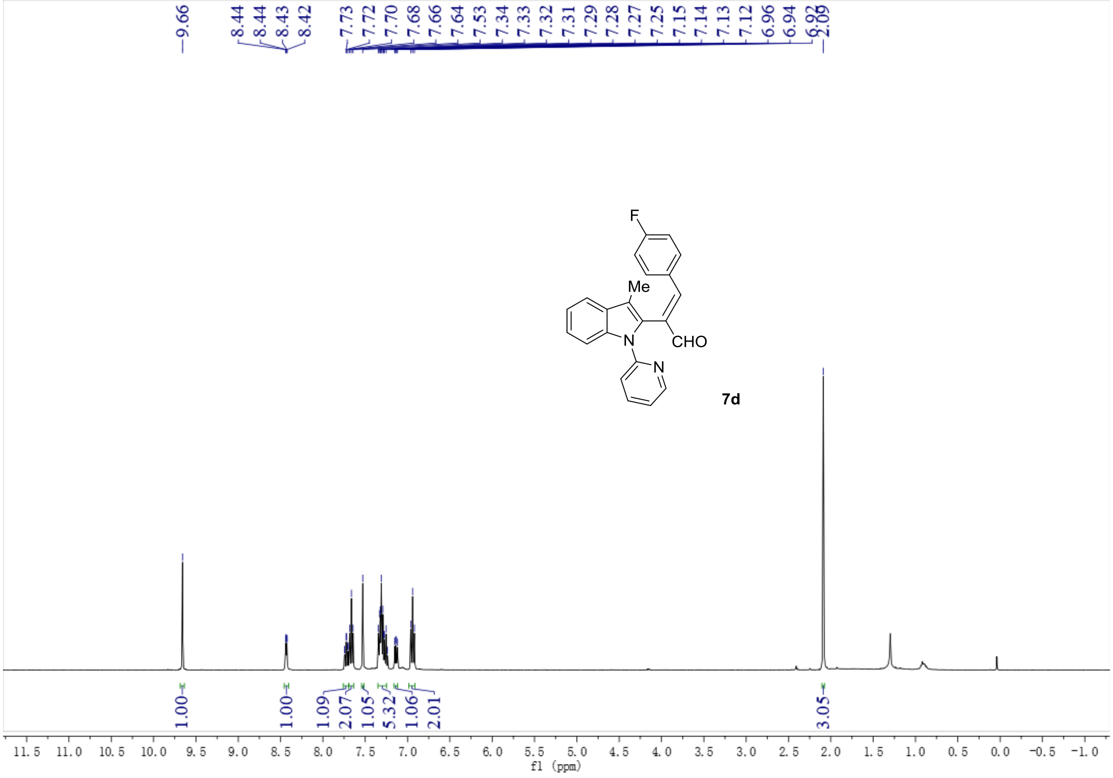

Supplementary Fig. 236 | ^{1}H NMR (400 MHz) of compound 7d (using $CDCl_3$ as solvent)

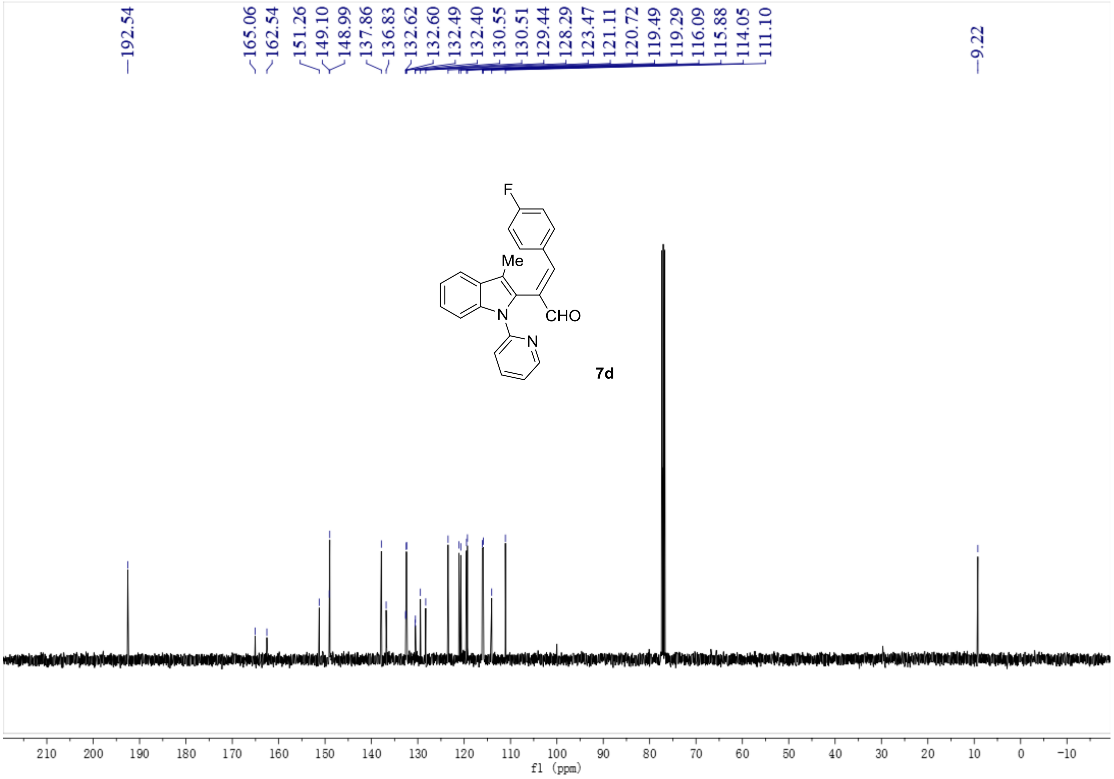

Supplementary Fig. 237 | ^{13}C NMR (101 MHz) of compound 7d (using $CDCl_3$ as solvent)

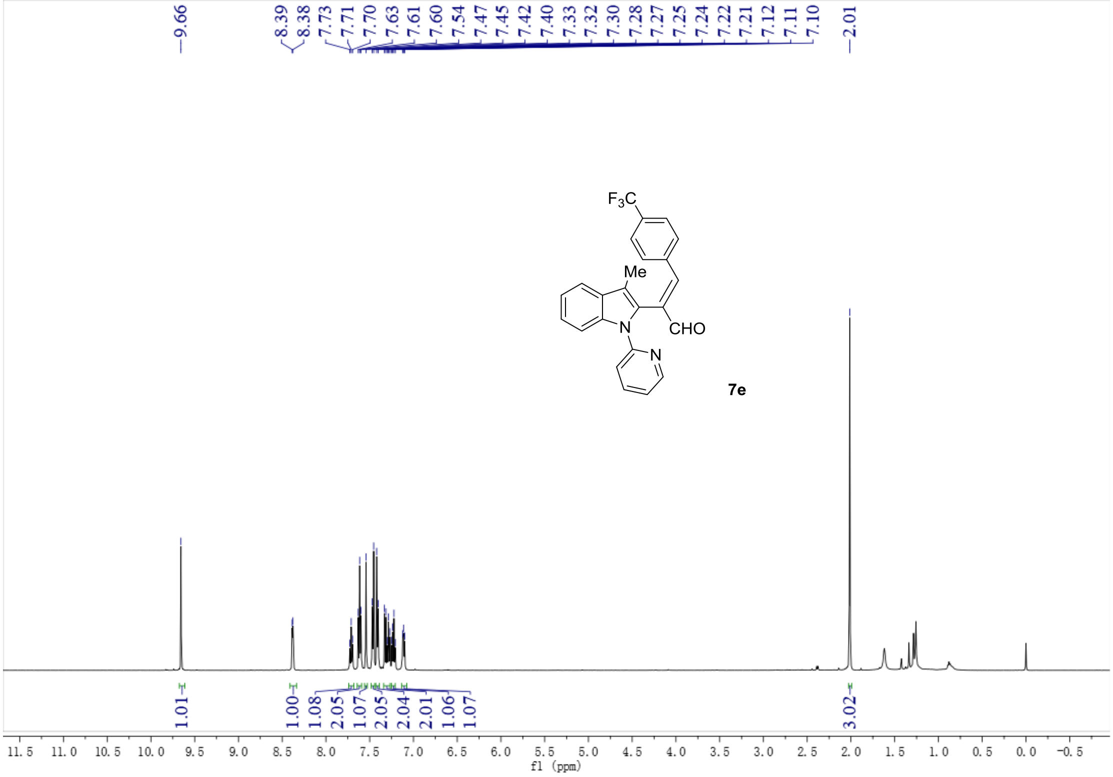

Supplementary Fig. 238 | ^{1}H NMR (500 MHz) of compound 7e (using $CDCl_3$ as solvent)

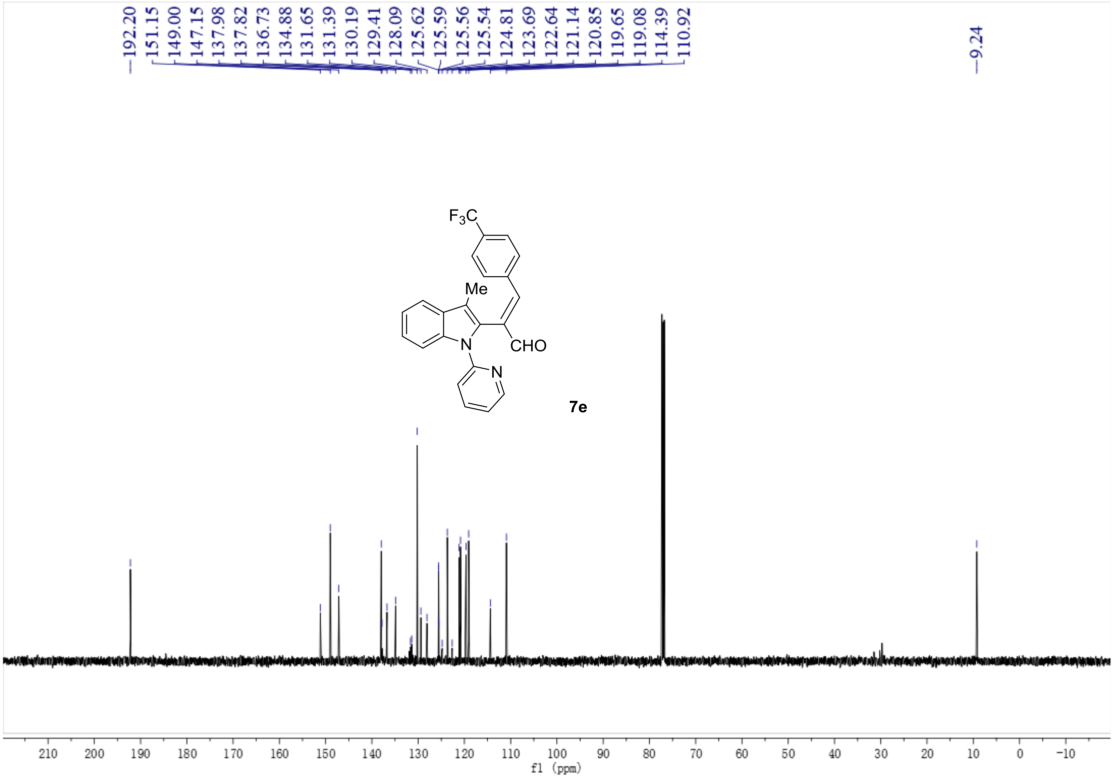

Supplementary Fig. 239 | ^{13}C NMR (126 MHz) of compound 7e (using $CDCl_3$ as solvent)

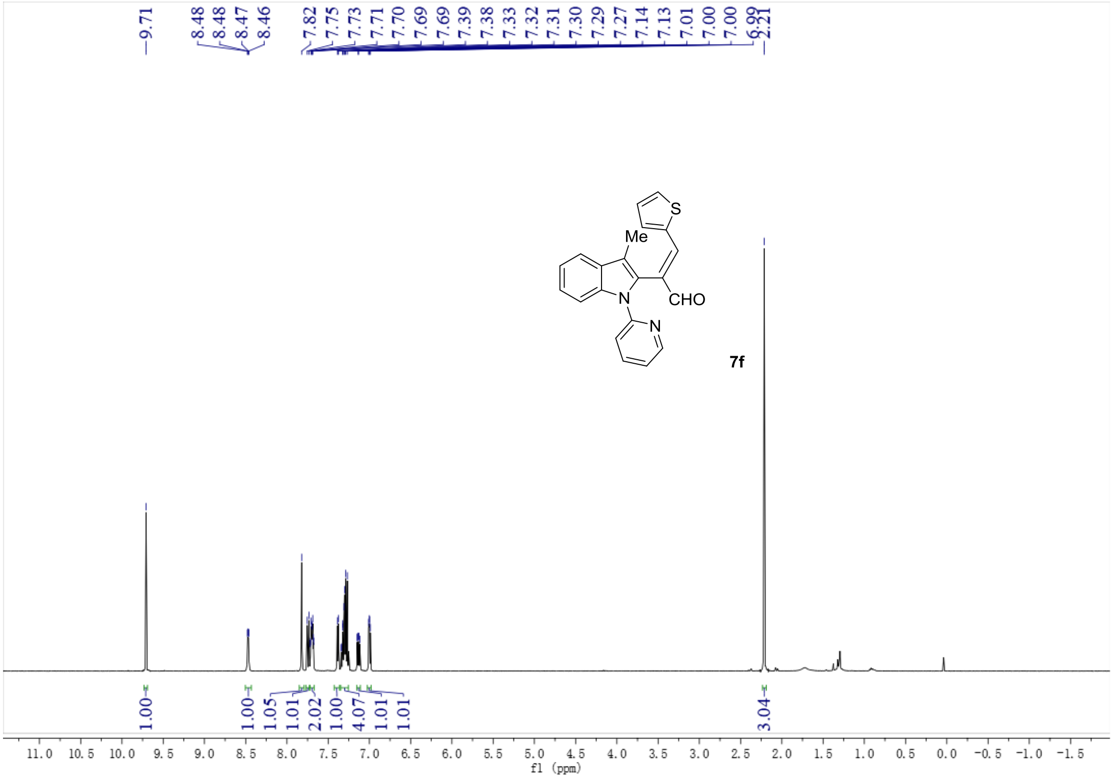

Supplementary Fig. 240 | ^{1}H NMR (400 MHz) of compound 7f (using $CDCl_3$ as solvent)

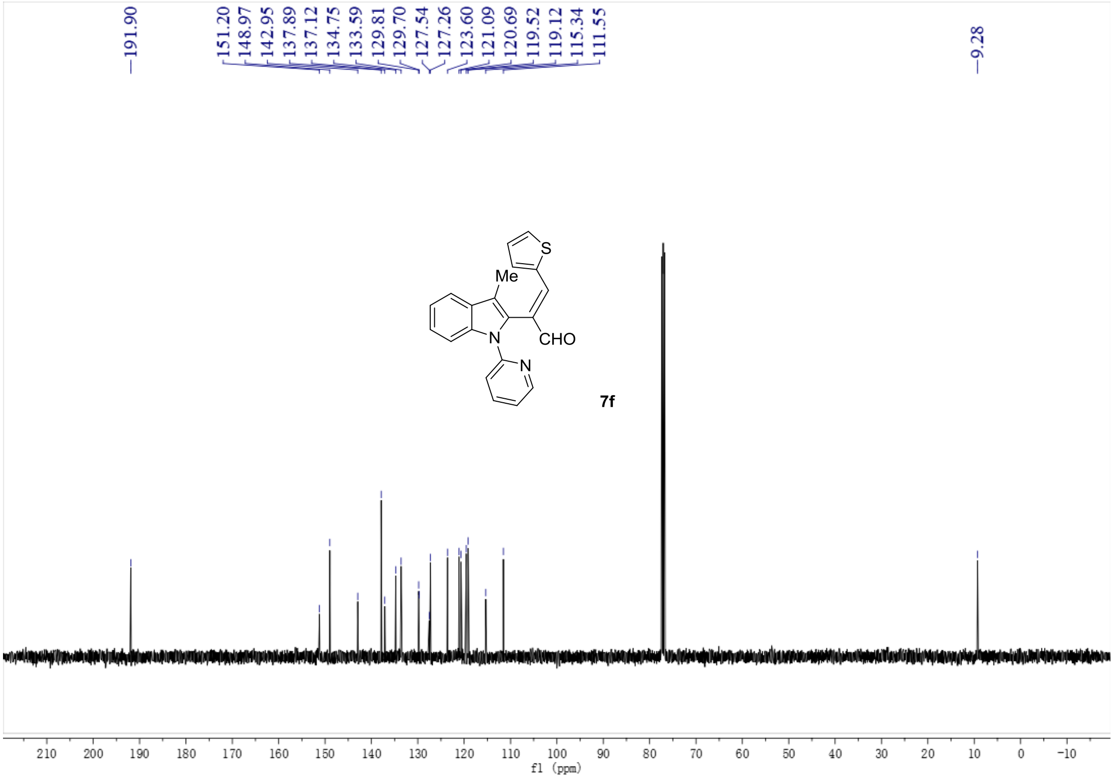

Supplementary Fig. 241 | ^{13}C NMR (101 MHz) of compound 7f (using $CDCl_3$ as solvent)

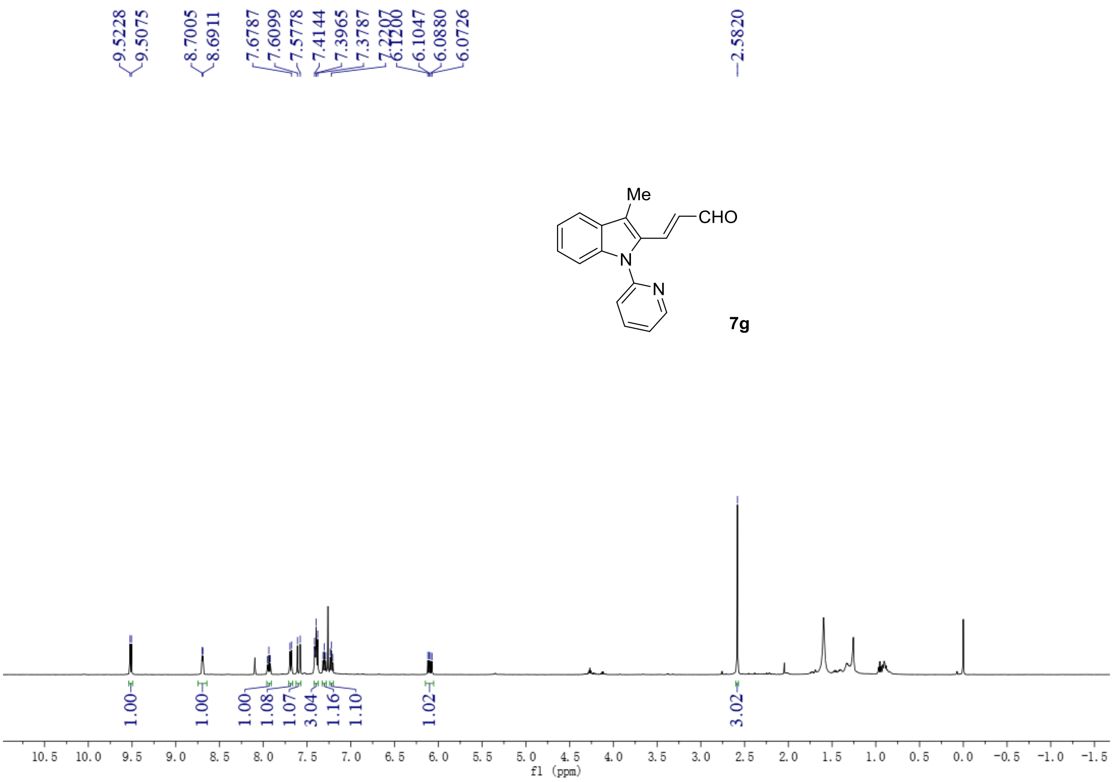

Supplementary Fig. 242 | ^{1}H NMR (500 MHz) of compound 7g (using $CDCl_3$ as solvent)

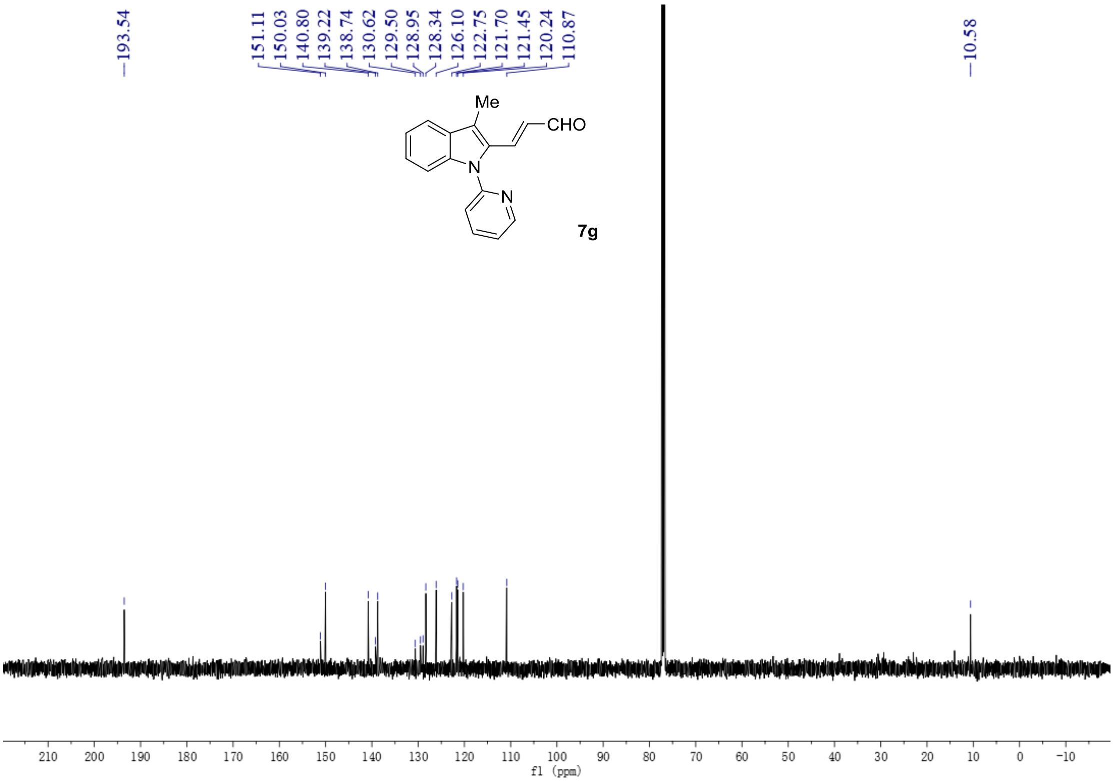

Supplementary Fig. 243 | ^{13}C NMR (126 MHz) of compound 7g (using $CDCl_3$ as solvent)

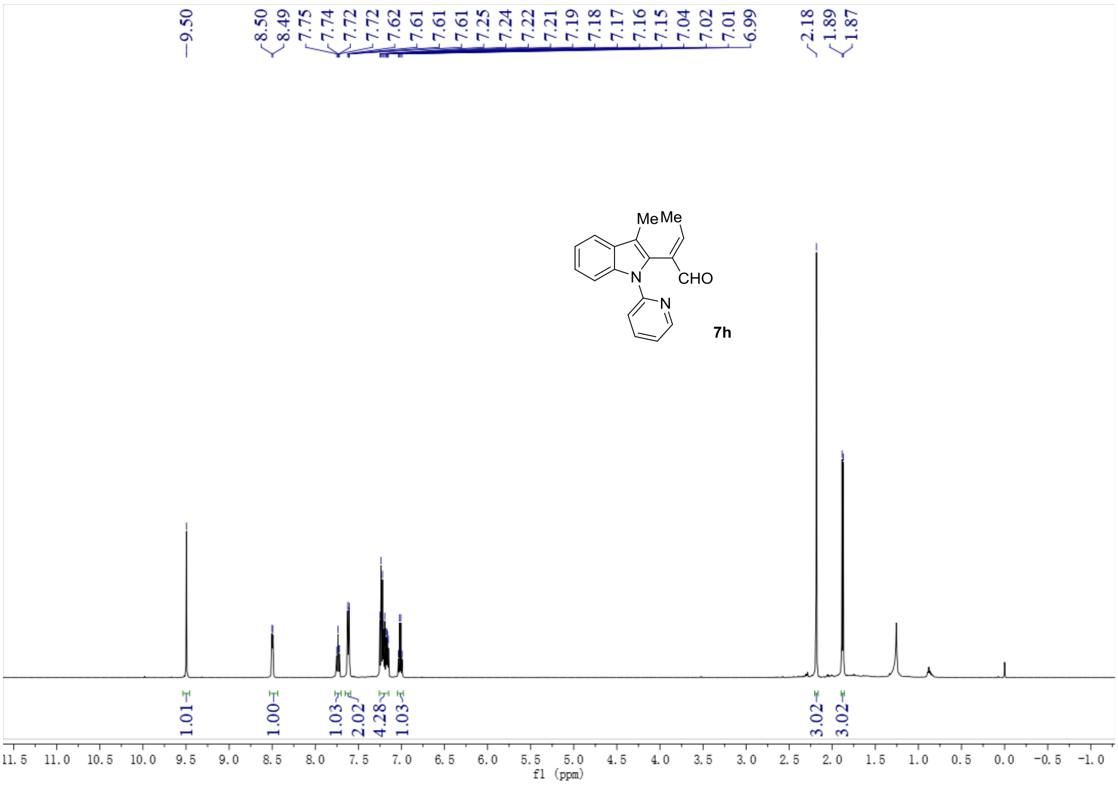

Supplementary Fig. 244 | ^{1}H NMR (500 MHz) of compound 7h (using $CDCl_3$ as solvent)

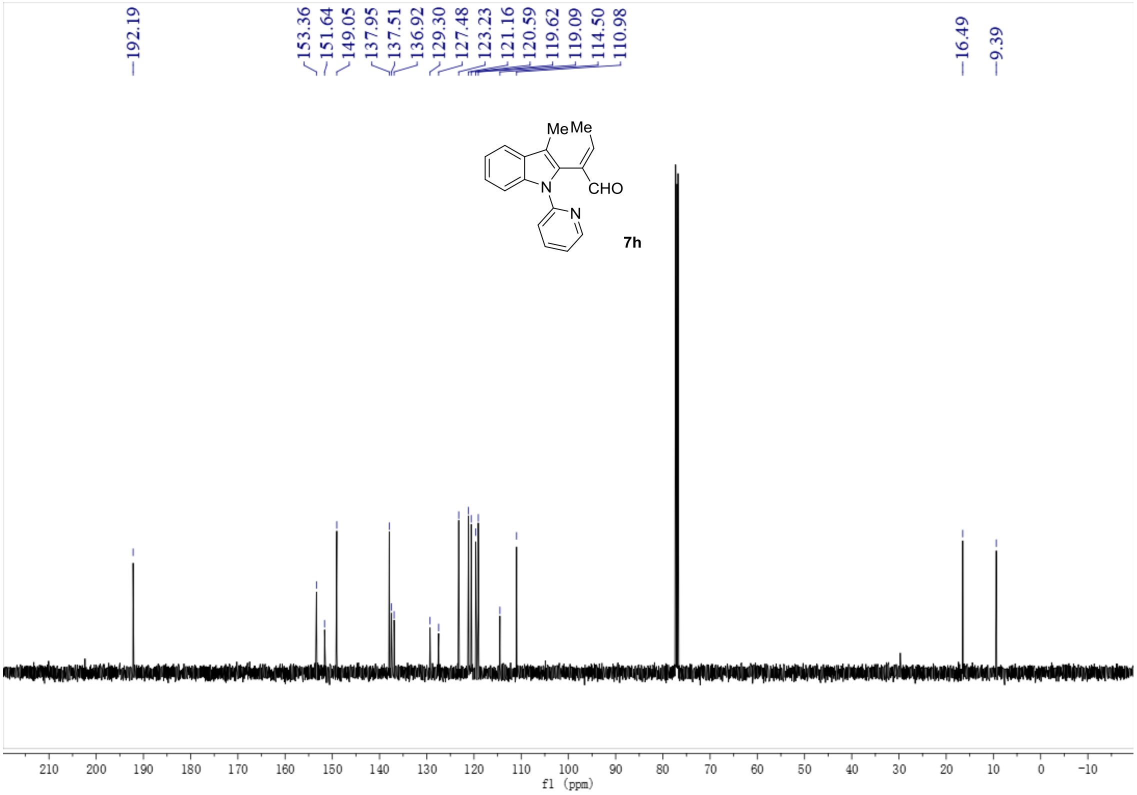

Supplementary Fig. 245 | ^{13}C NMR (126 MHz) of compound 7h (using $CDCl_3$ as solvent)

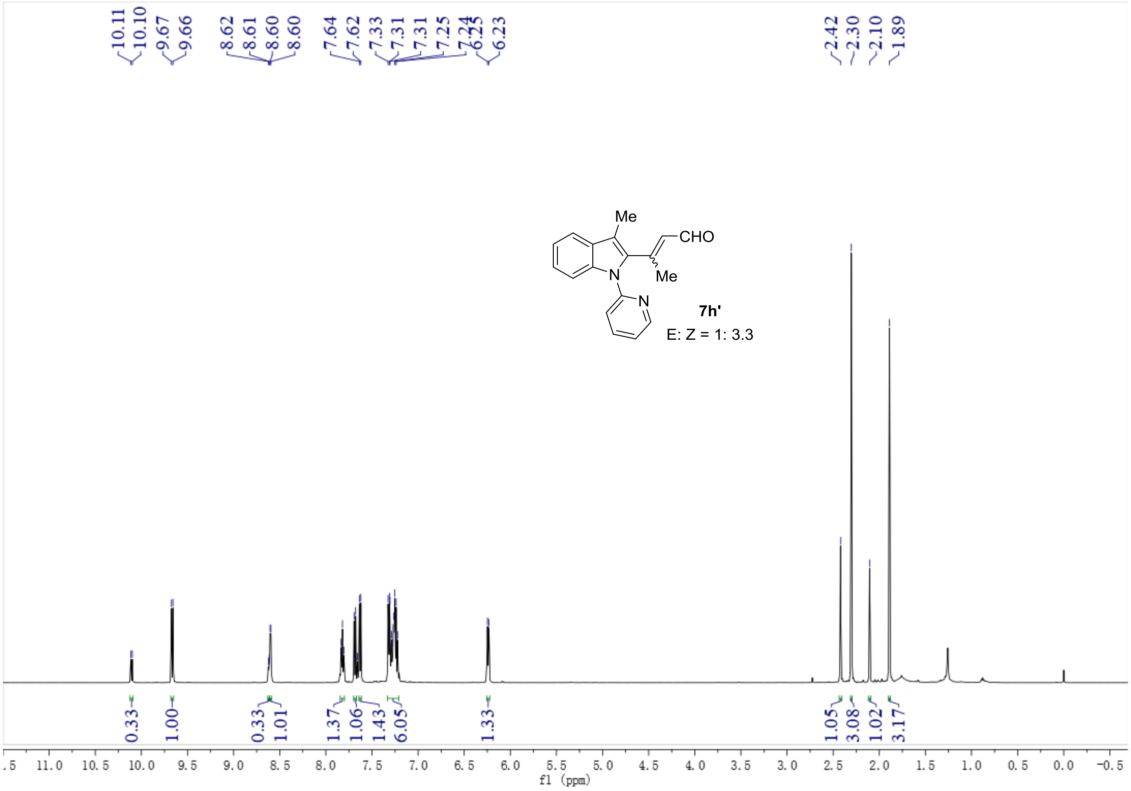

Supplementary Fig. 246 | ^{1}H NMR (500 MHz) of compound 7h′ (using $CDCl_3$ as solvent)

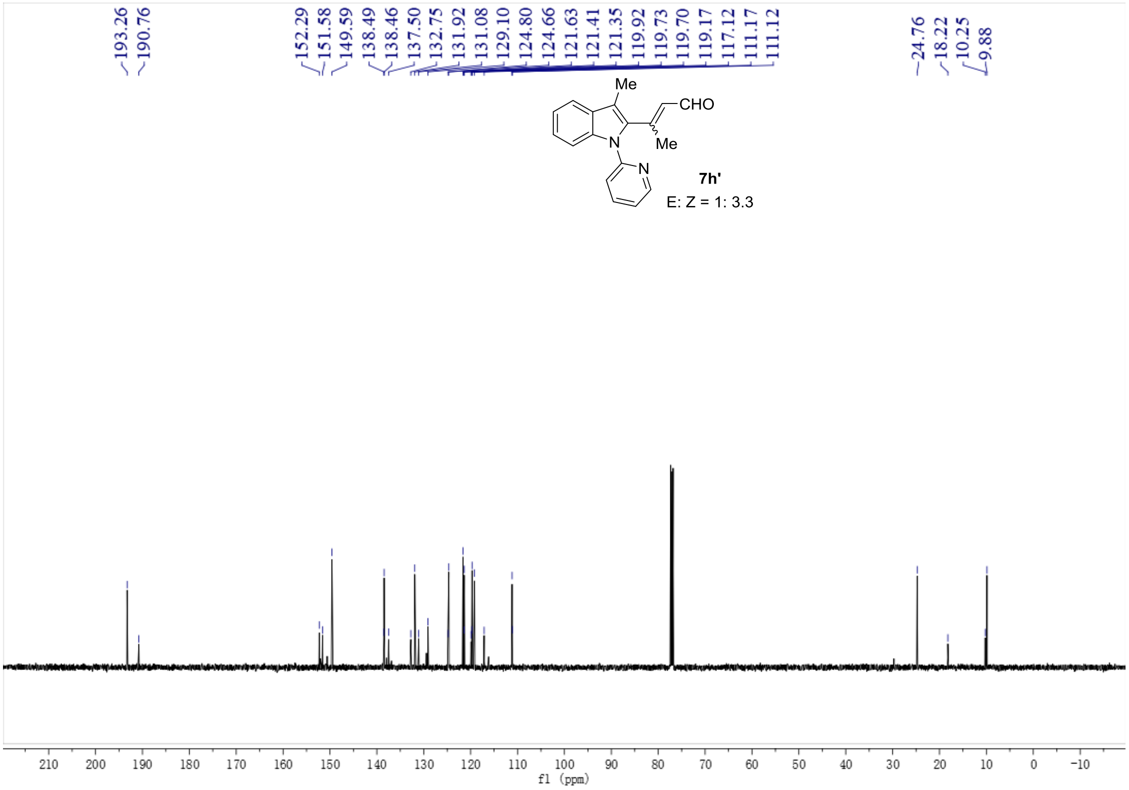

Supplementary Fig. 247 | ^{13}C NMR (126 MHz) of compound 7h′ (using $CDCl_3$ as solvent)

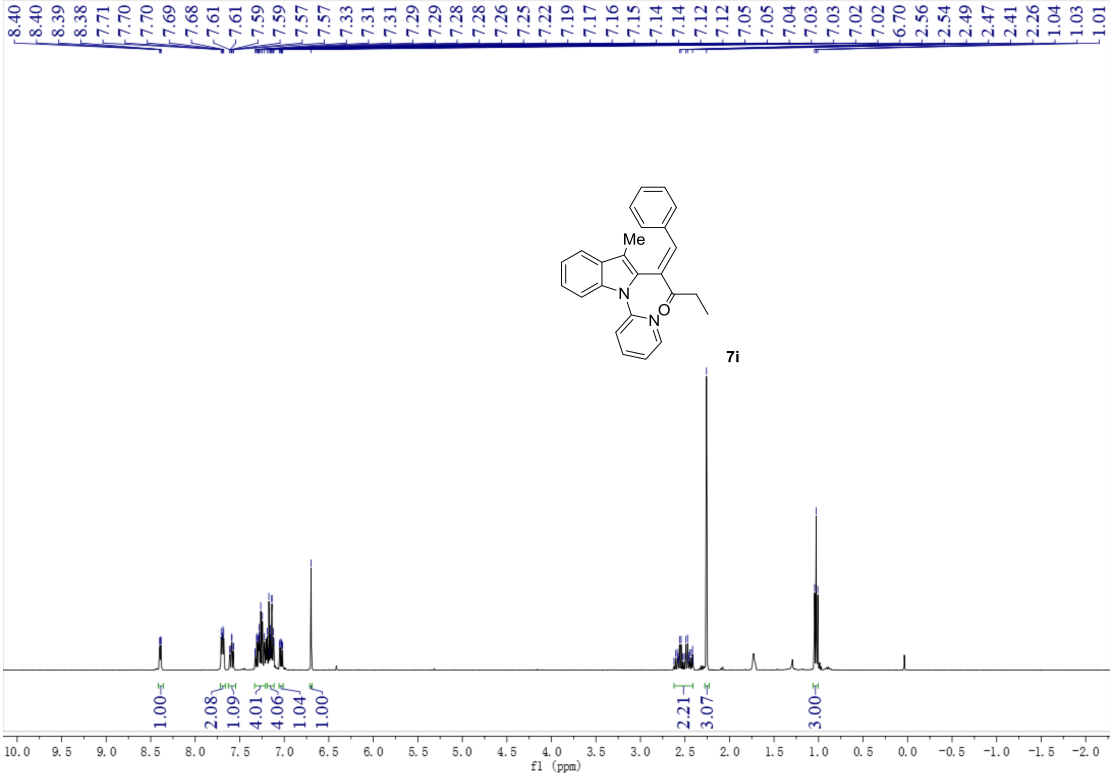

Supplementary Fig. 248 | ^{1}H NMR (400 MHz) of compound 7i (using $CDCl_3$ as solvent)

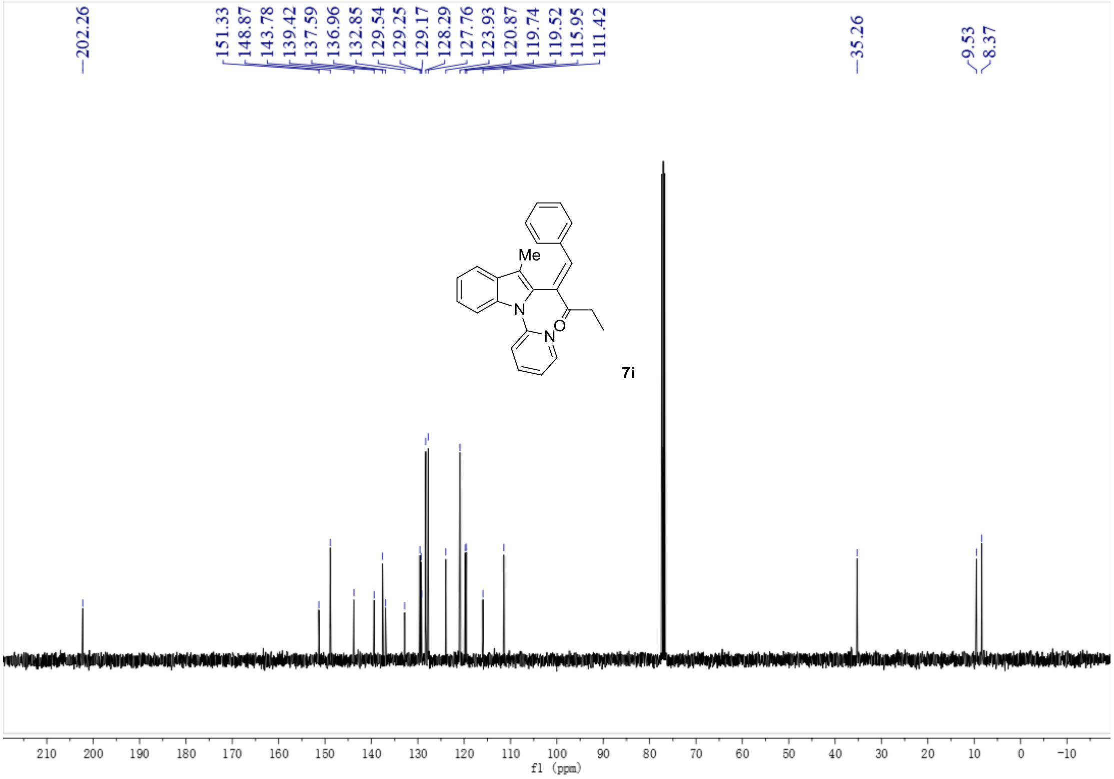

Supplementary Fig. 249 | ^{13}C NMR (101 MHz) of compound 7i (using $CDCl_3$ as solvent)

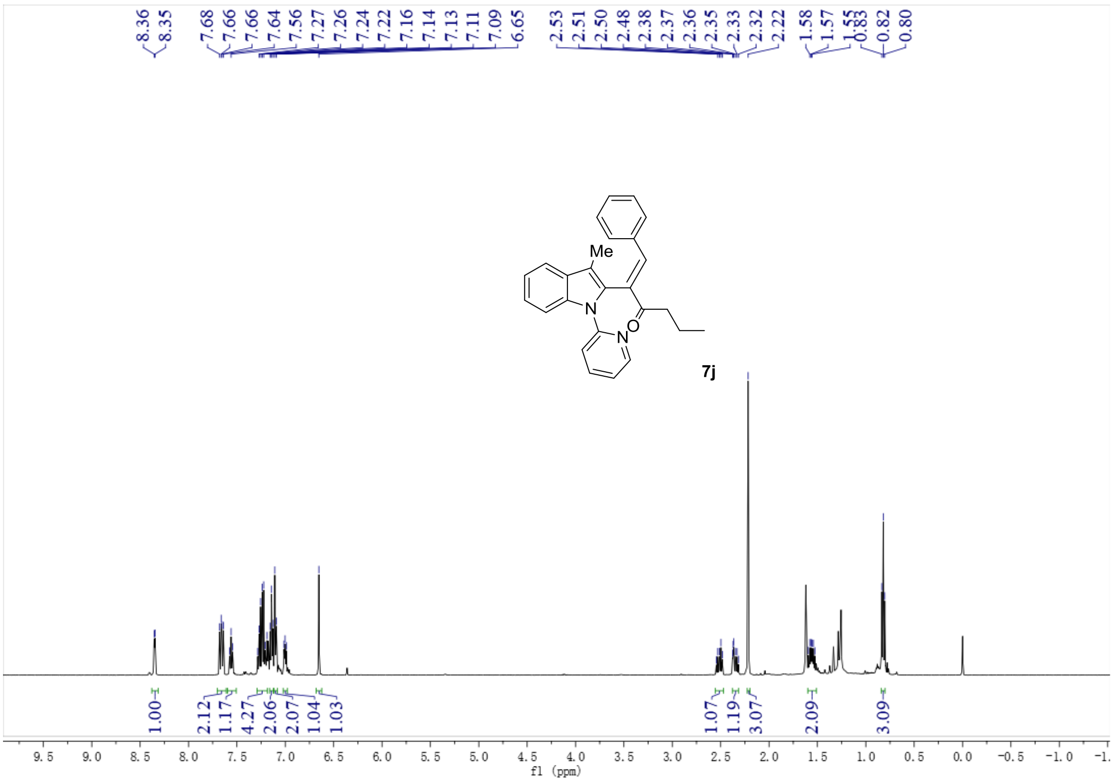

Supplementary Fig. 250 | ^{1}H NMR (500 MHz) of compound 7j (using $CDCl_3$ as solvent)

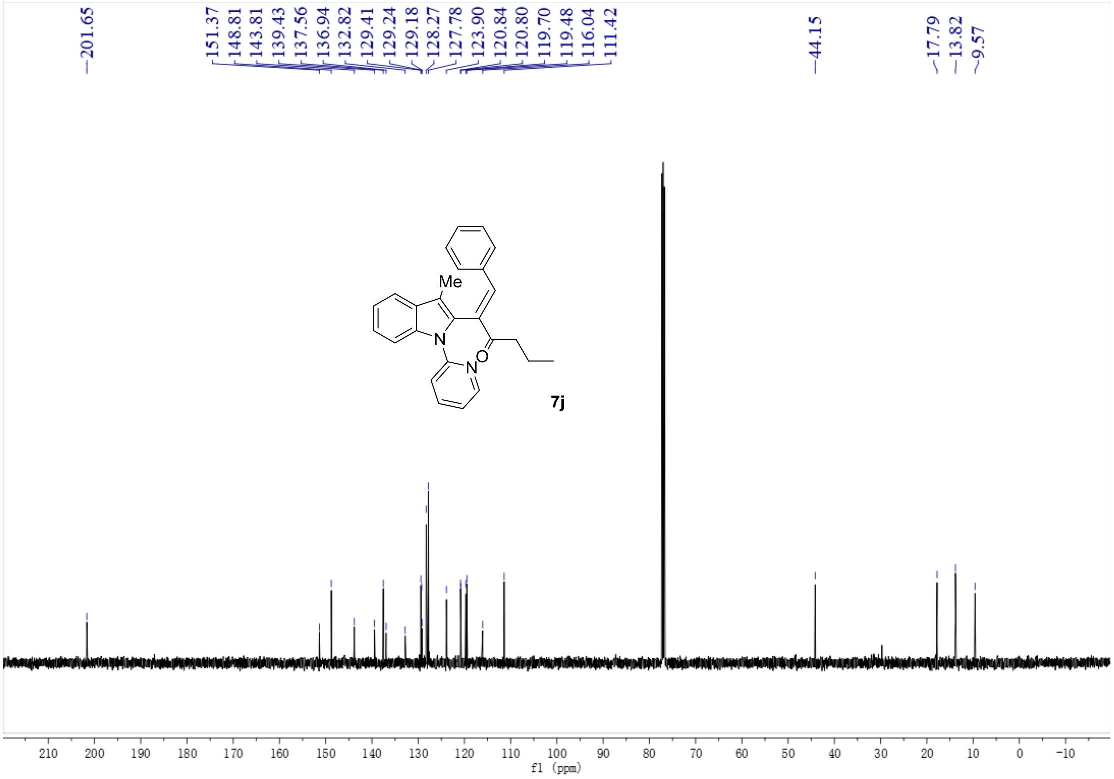

Supplementary Fig. 251 | ^{13}C NMR (126 MHz) of compound 7j (using $CDCl_3$ as solvent)

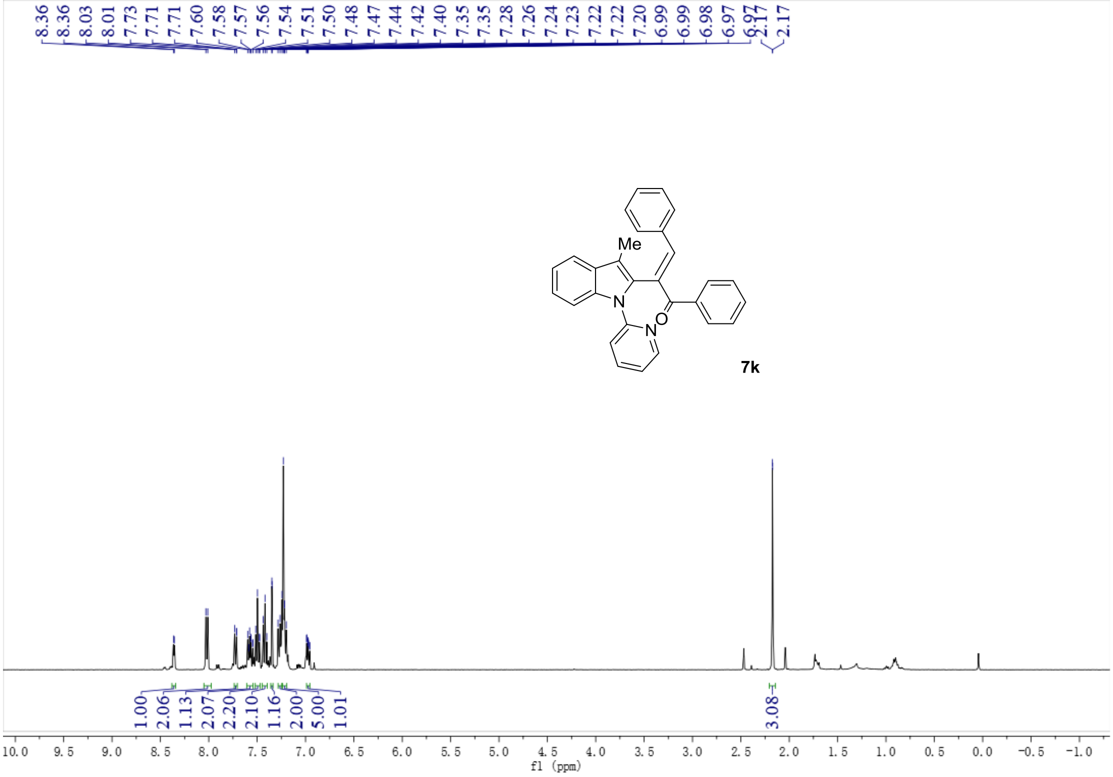

Supplementary Fig. 252 | ^{1}H NMR (400 MHz) of compound 7k (using $CDCl_3$ as solvent)

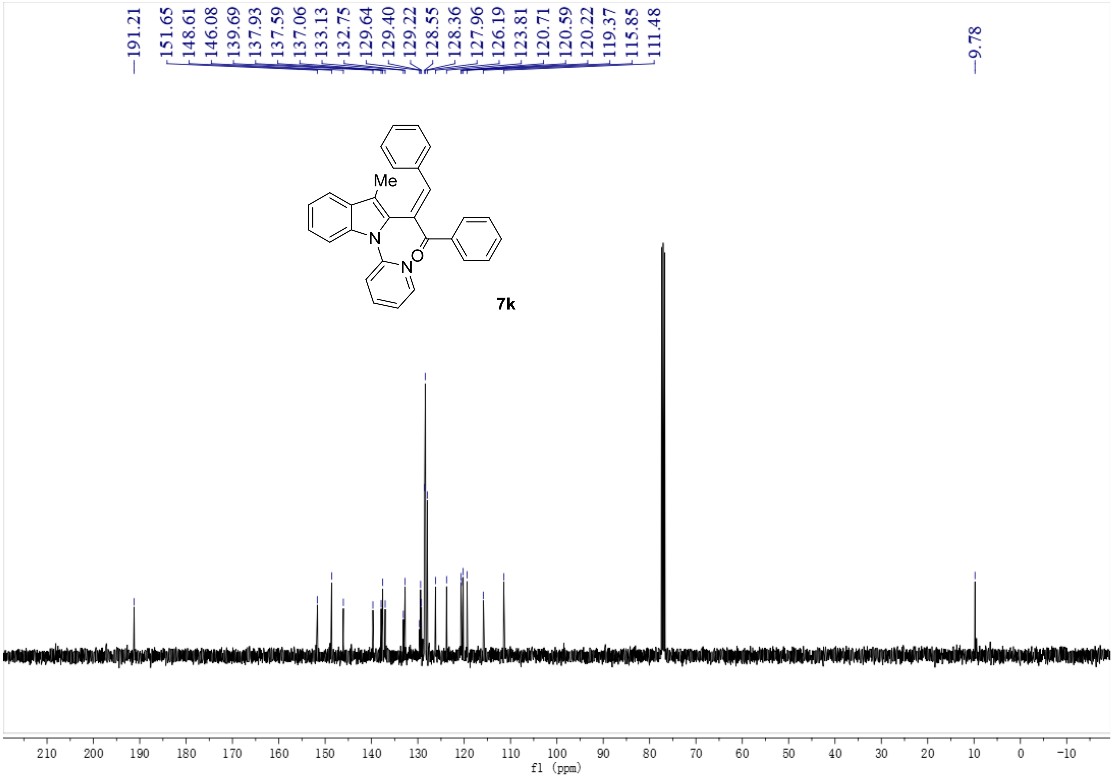

Supplementary Fig. 253 | ^{13}C NMR (101 MHz) of compound 7k (using $CDCl_3$ as solvent)

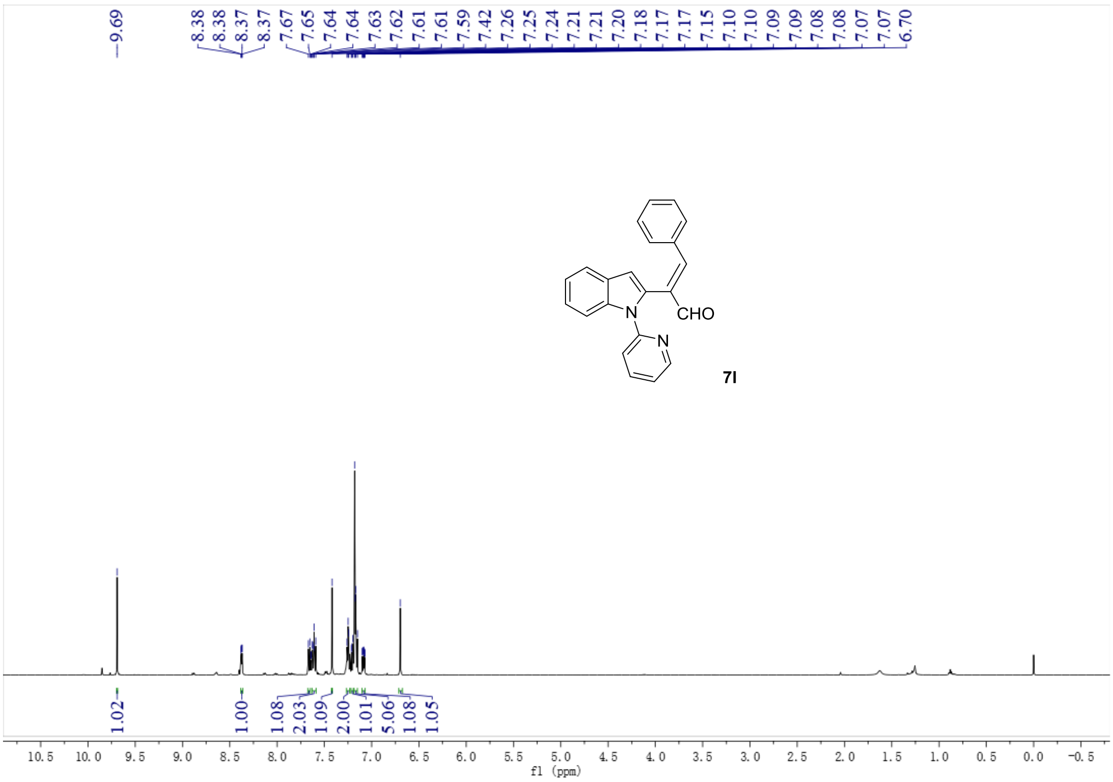

Supplementary Fig. 254 | ^{1}H NMR (500 MHz) of compound 7l (using $CDCl_3$ as solvent)

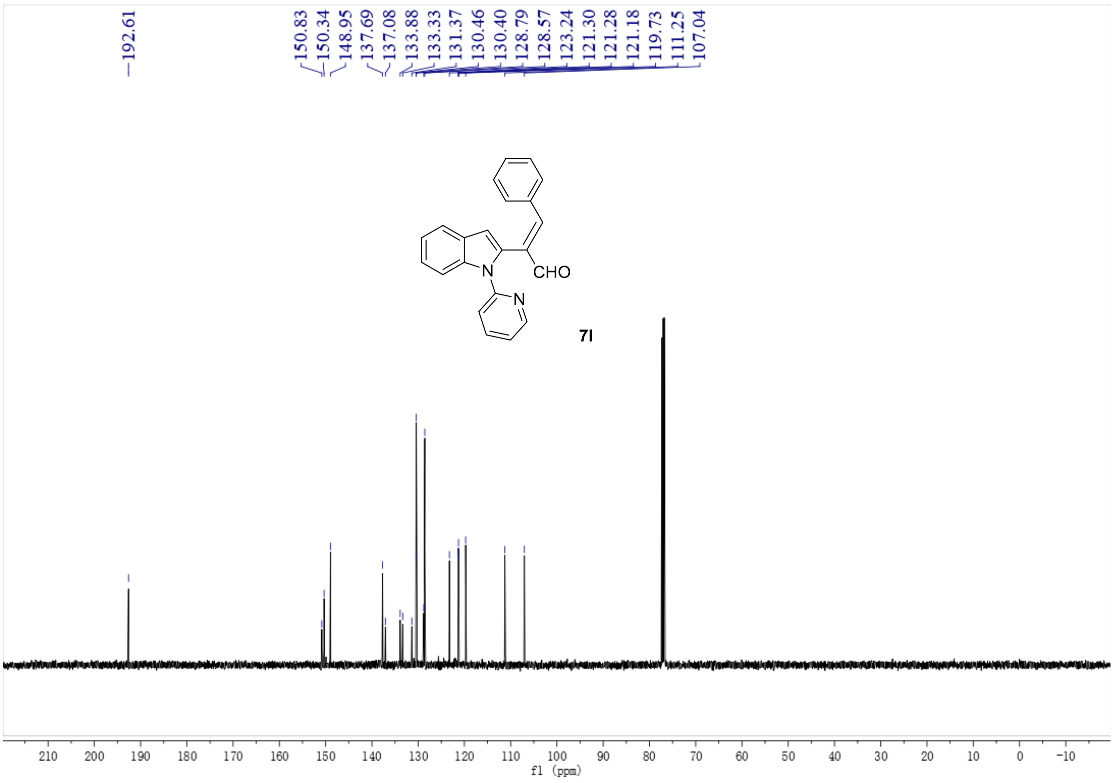

Supplementary Fig. 255 | ^{13}C NMR (126 MHz) of compound 7l (using $CDCl_3$ as solvent)

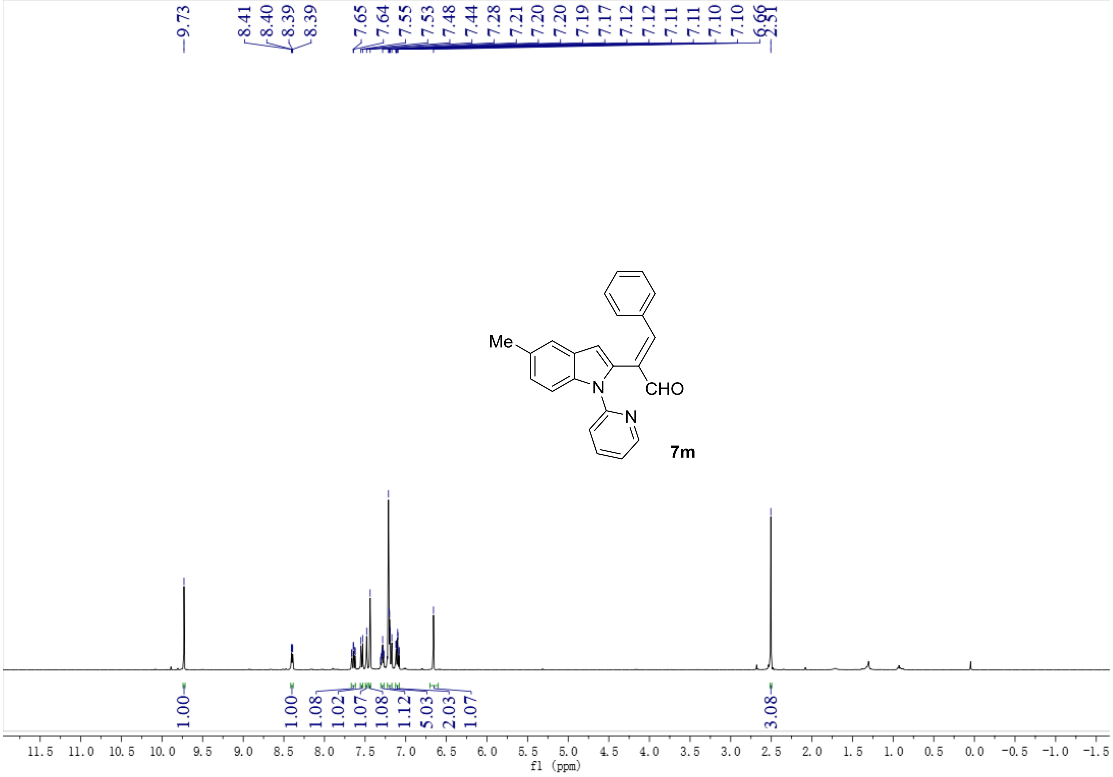

Supplementary Fig. 256 | ^{1}H NMR (400 MHz) of compound 7m (using $CDCl_3$ as solvent)

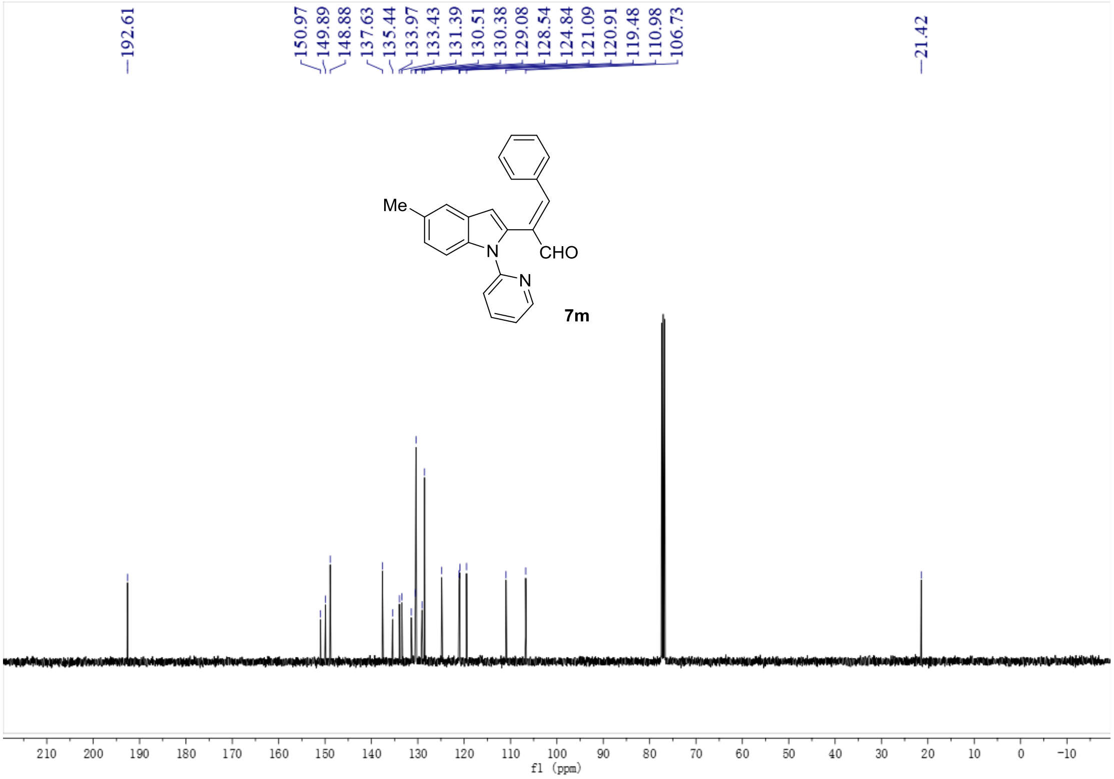

Supplementary Fig. 257 | ^{13}C NMR (101 MHz) of compound 7m (using $CDCl_3$ as solvent)

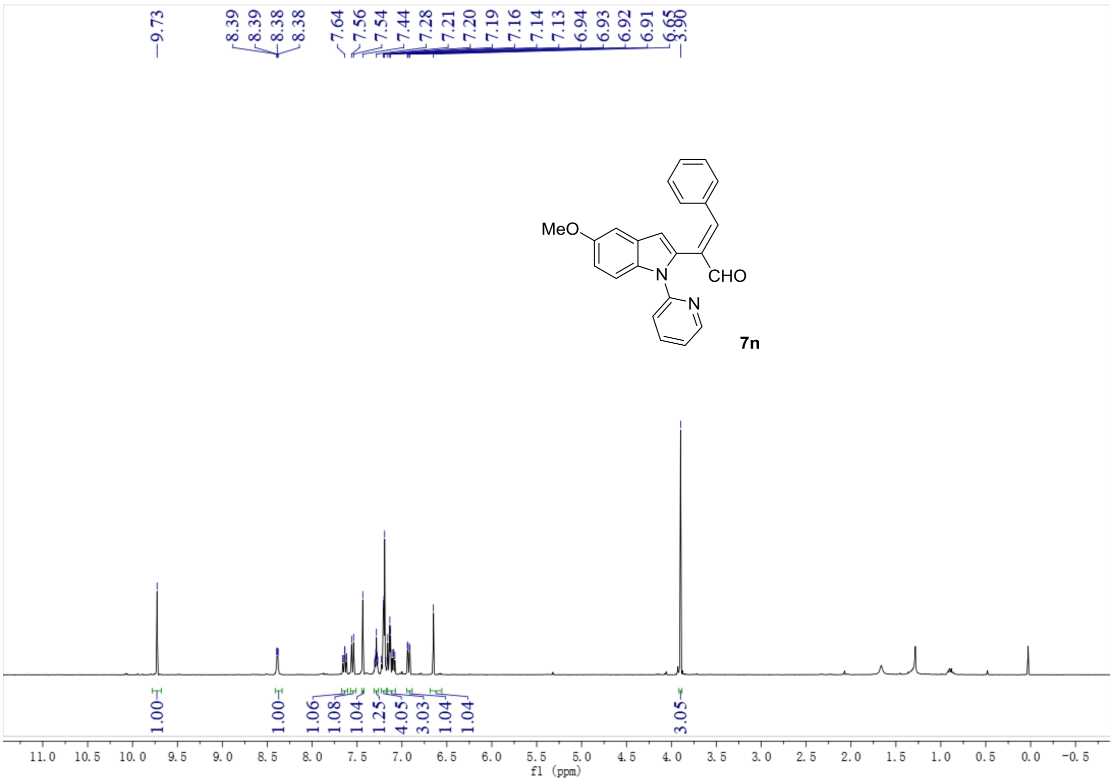

Supplementary Fig. 258 | ^{1}H NMR (400 MHz) of compound 7n (using $CDCl_3$ as solvent)

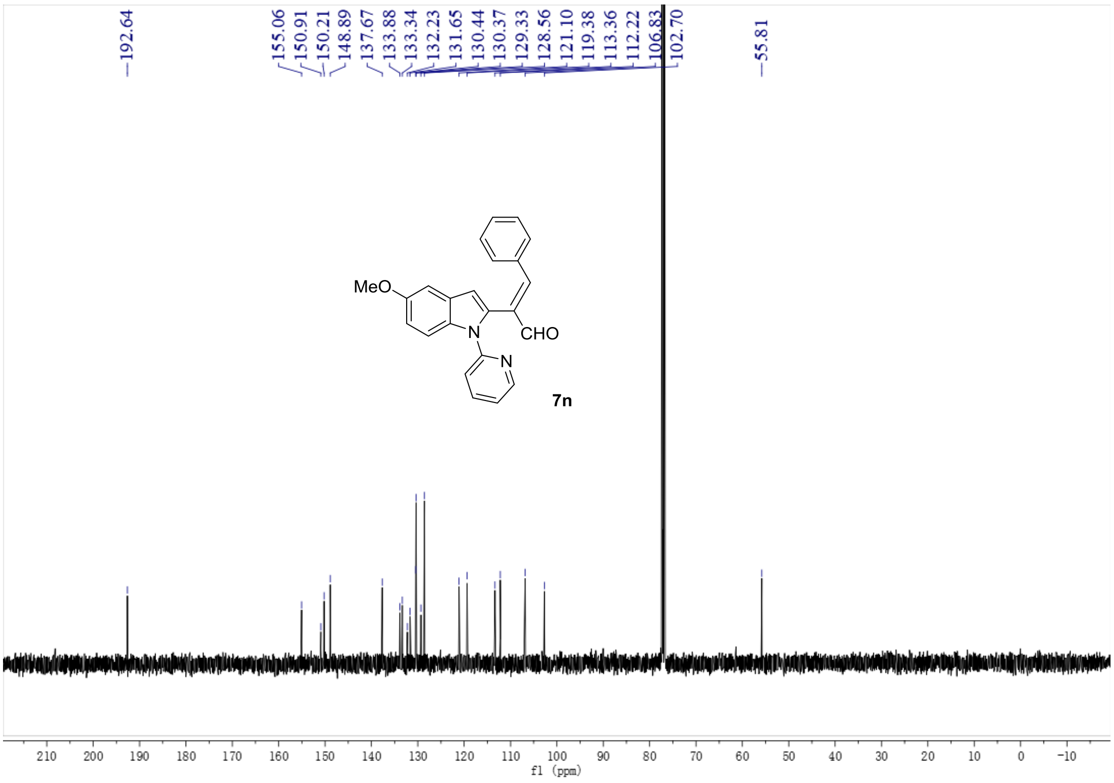

Supplementary Fig. 259 | ^{13}C NMR (101 MHz) of compound 7n (using $CDCl_3$ as solvent)

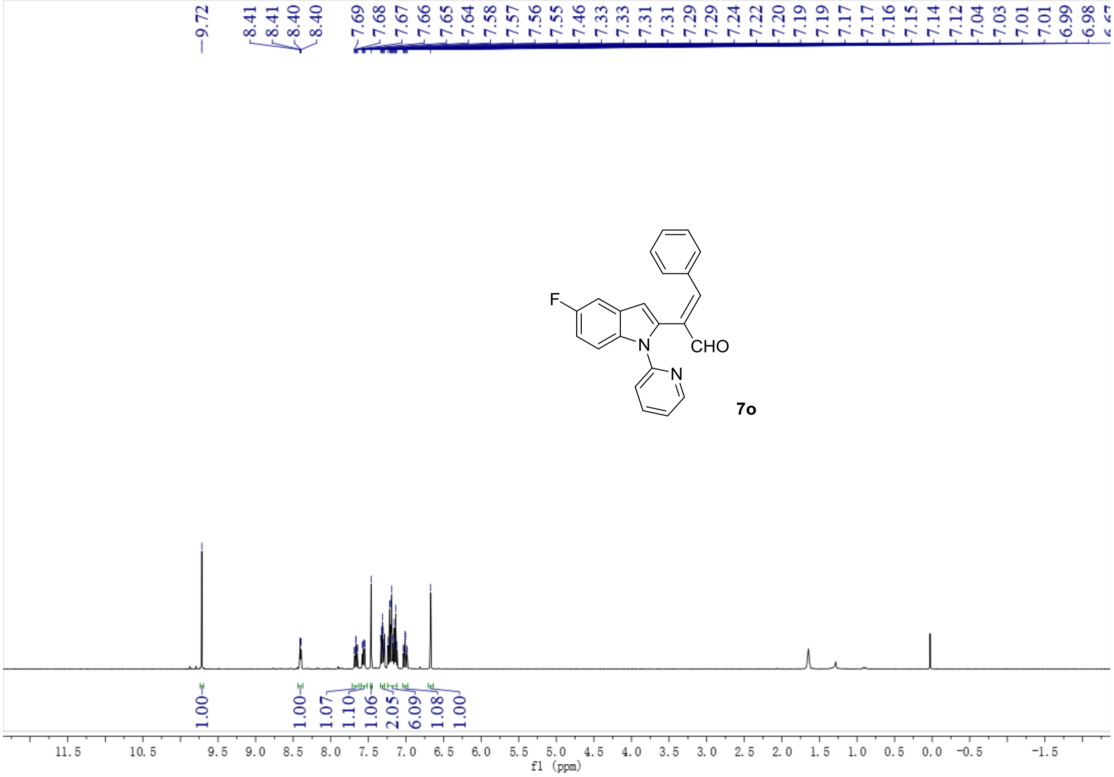

Supplementary Fig. 260 | ^{1}H NMR (400 MHz) of compound 7o (using $CDCl_3$ as solvent)

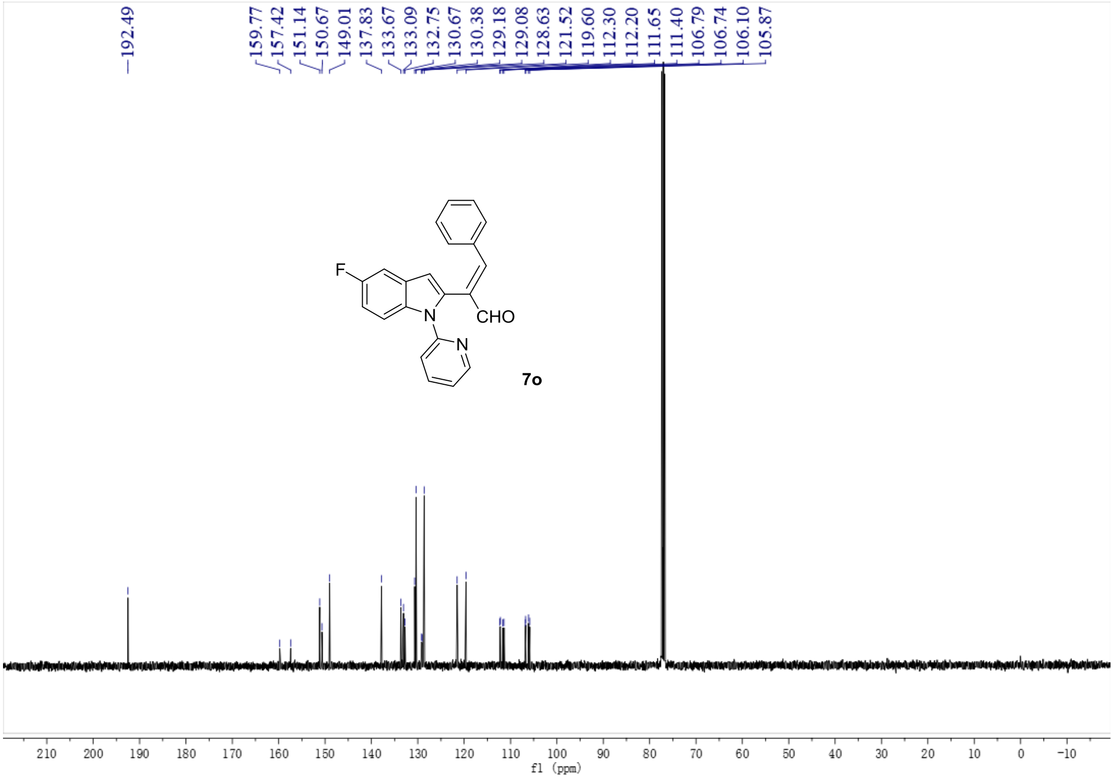

Supplementary Fig. 261 | ^{13}C NMR (101 MHz) of compound 7o (using $CDCl_3$ as solvent)

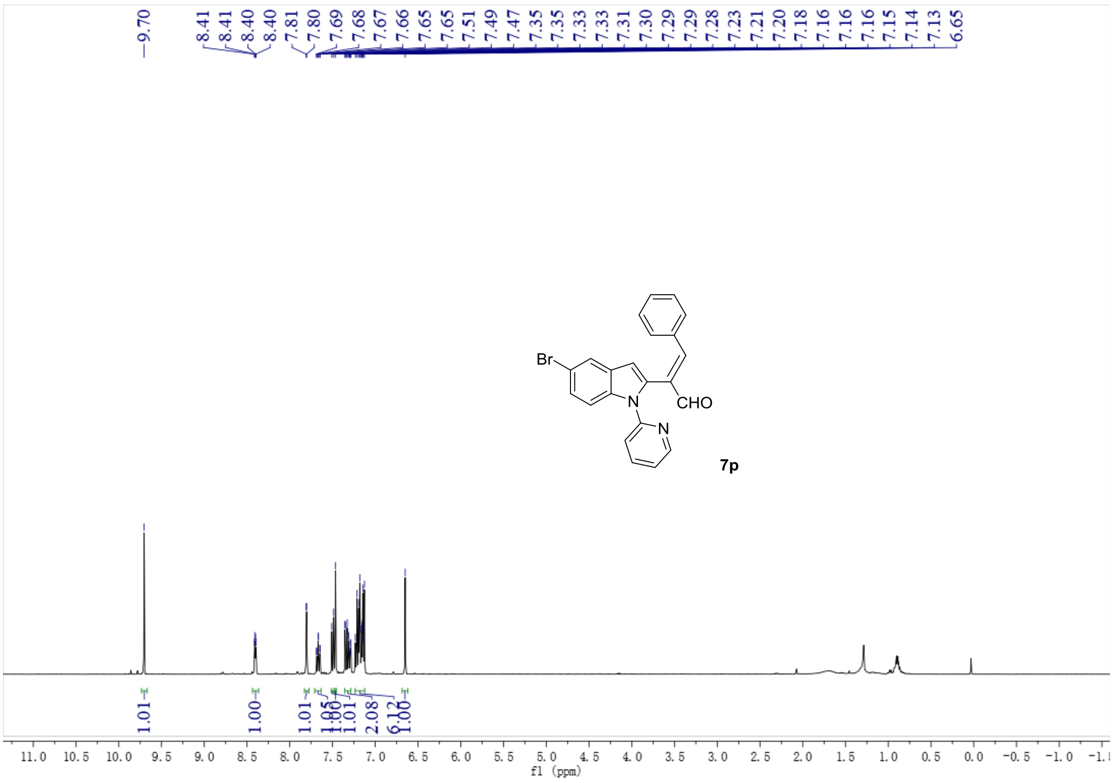

Supplementary Fig. 262 | ^{1}H NMR (400 MHz) of compound 7p (using $CDCl_3$ as solvent)

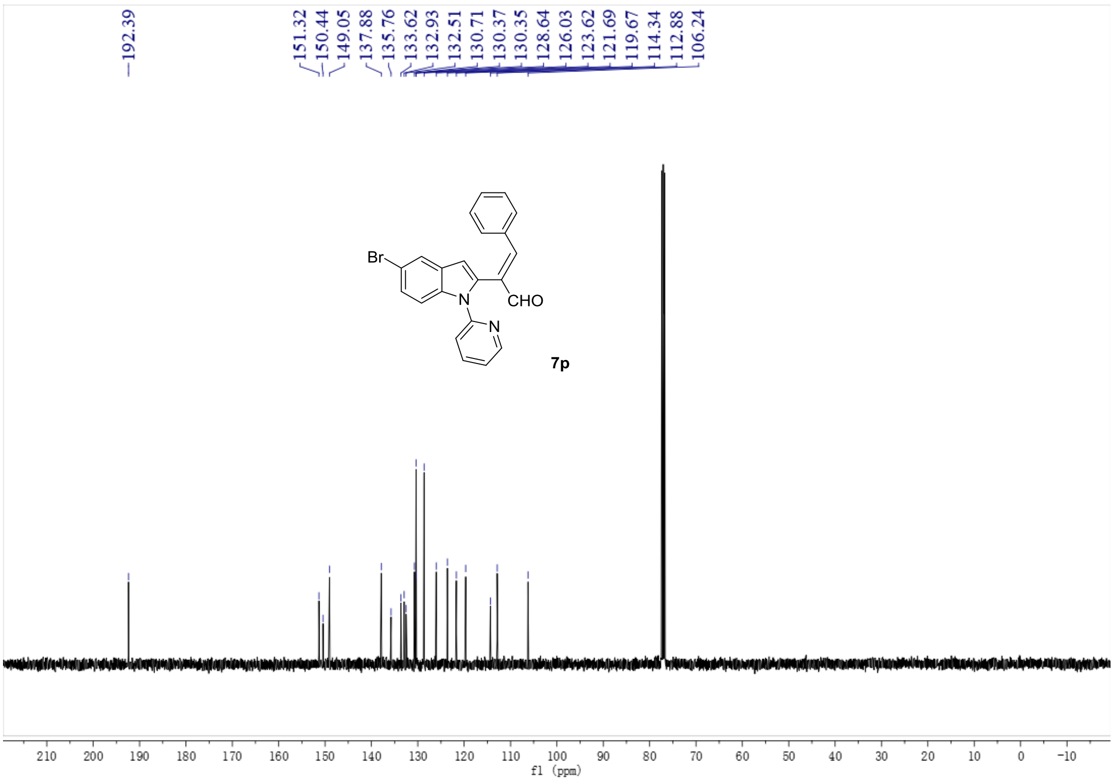

Supplementary Fig. 263 | ^{13}C NMR (101 MHz) of compound 7p (using $CDCl_3$ as solvent)

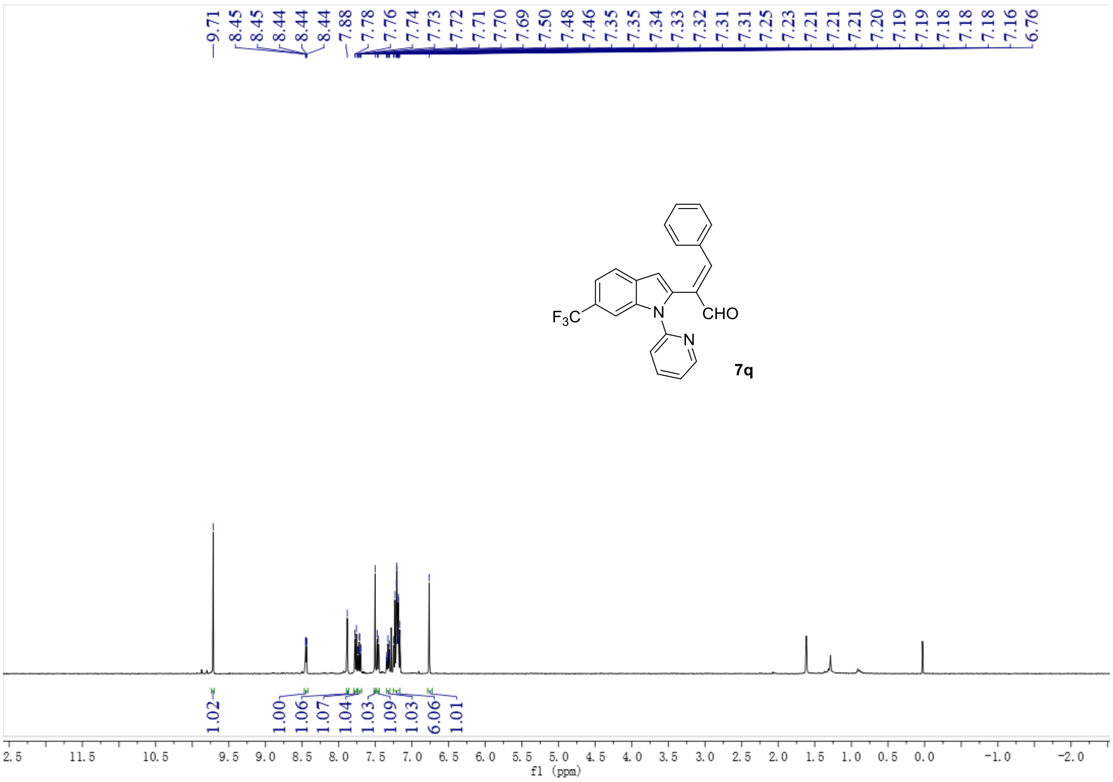

Supplementary Fig. 264 | ^{1}H NMR (400 MHz) of compound 7q (using $CDCl_3$ as solvent)

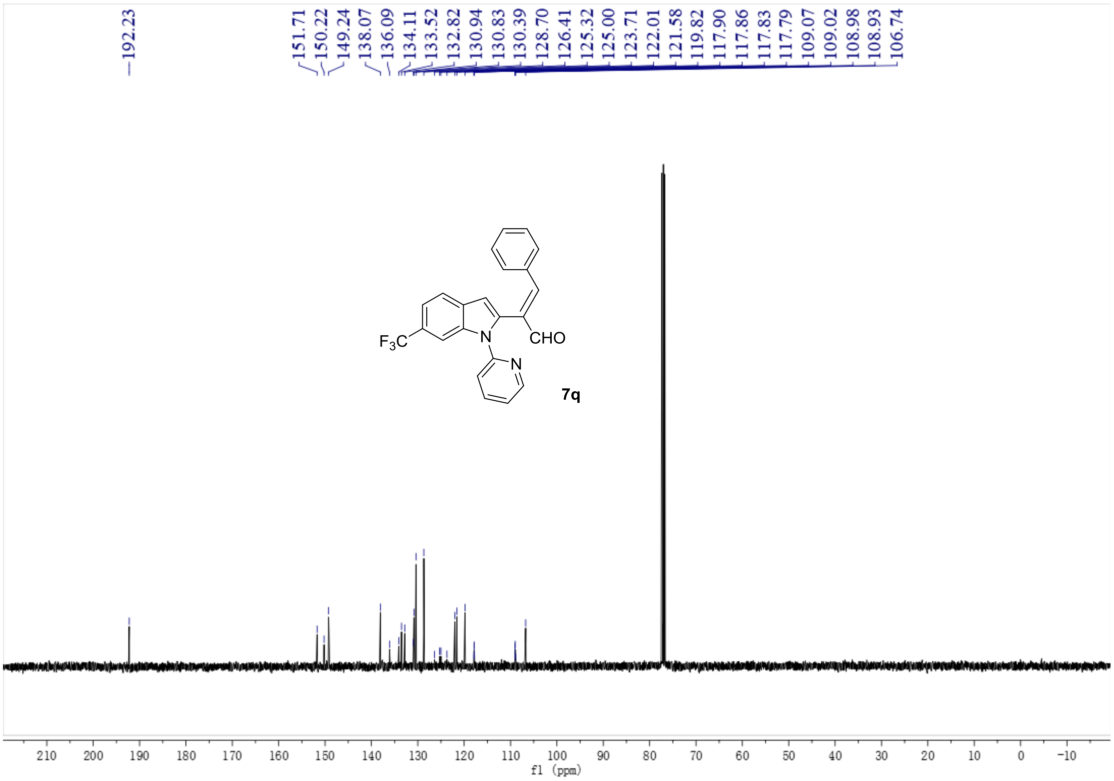

Supplementary Fig. 265 | ^{13}C NMR (101 MHz) of compound 7q (using $CDCl_3$ as solvent)

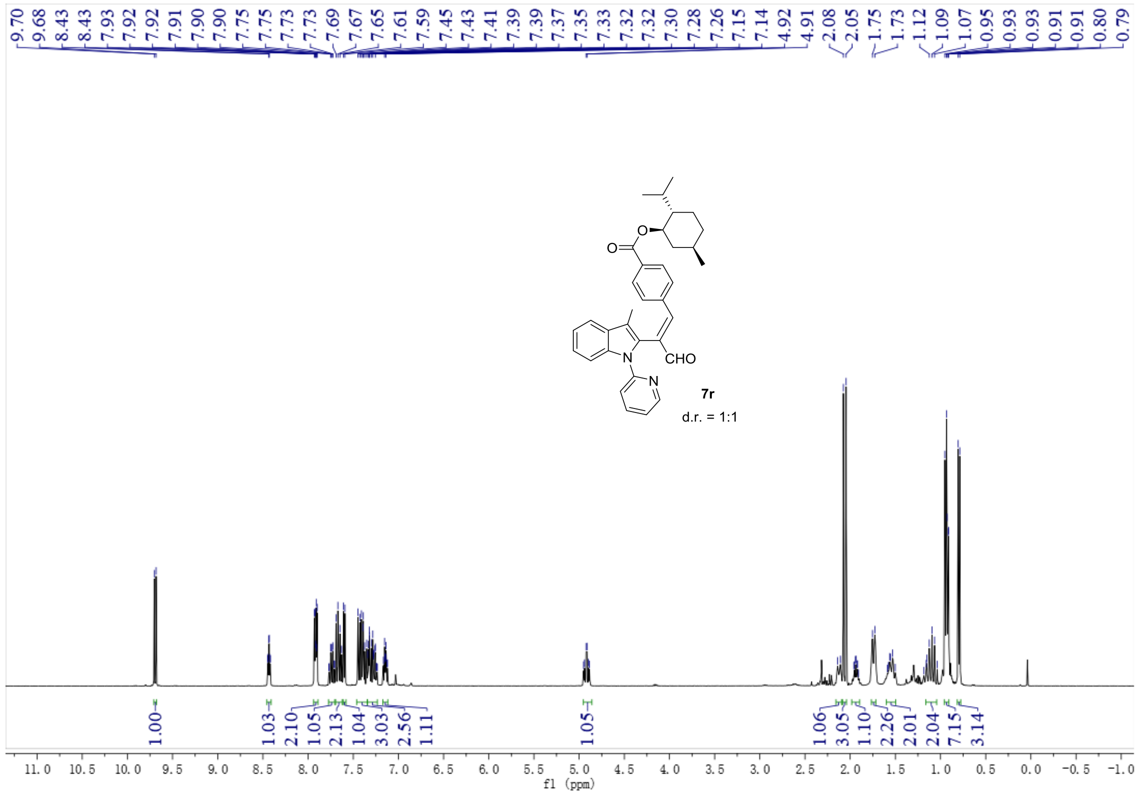

Supplementary Fig. 266 | ^{1}H NMR (400 MHz) of compound 7r (using $CDCl_3$ as solvent)

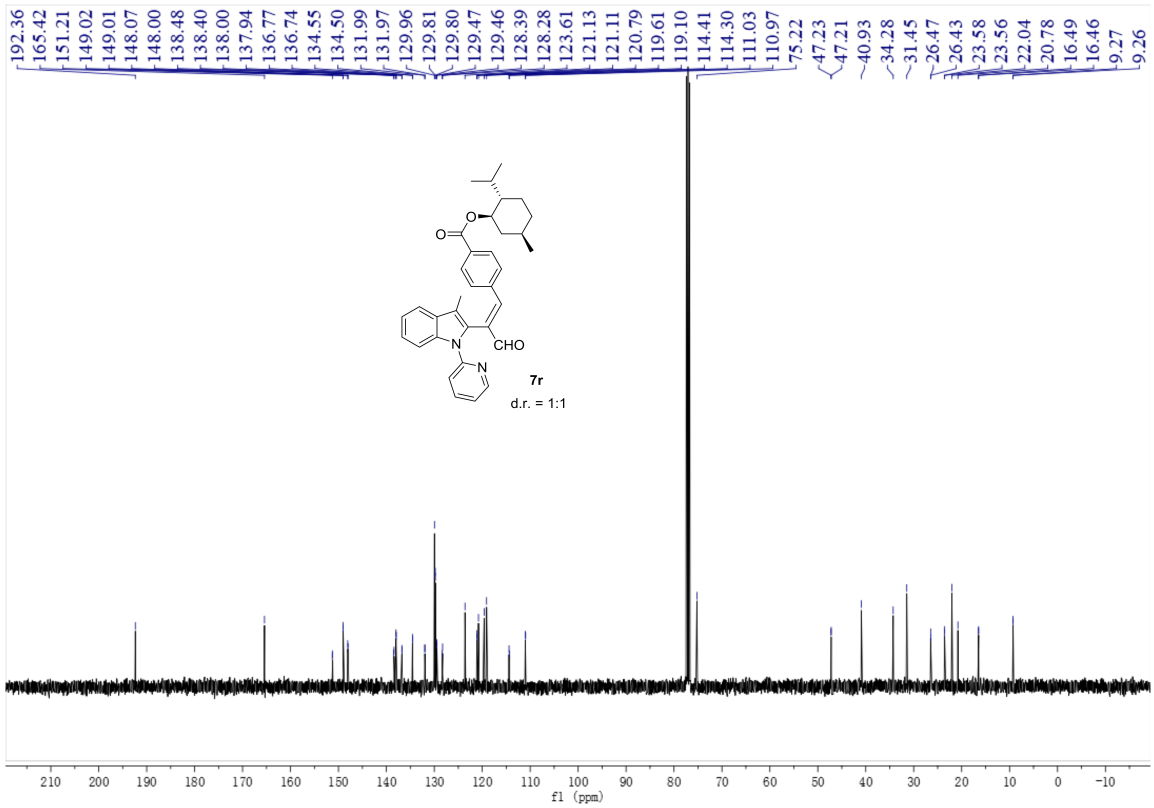

Supplementary Fig. 267 | ^{13}C NMR (101 MHz) of compound 7r (using $CDCl_3$ as solvent)

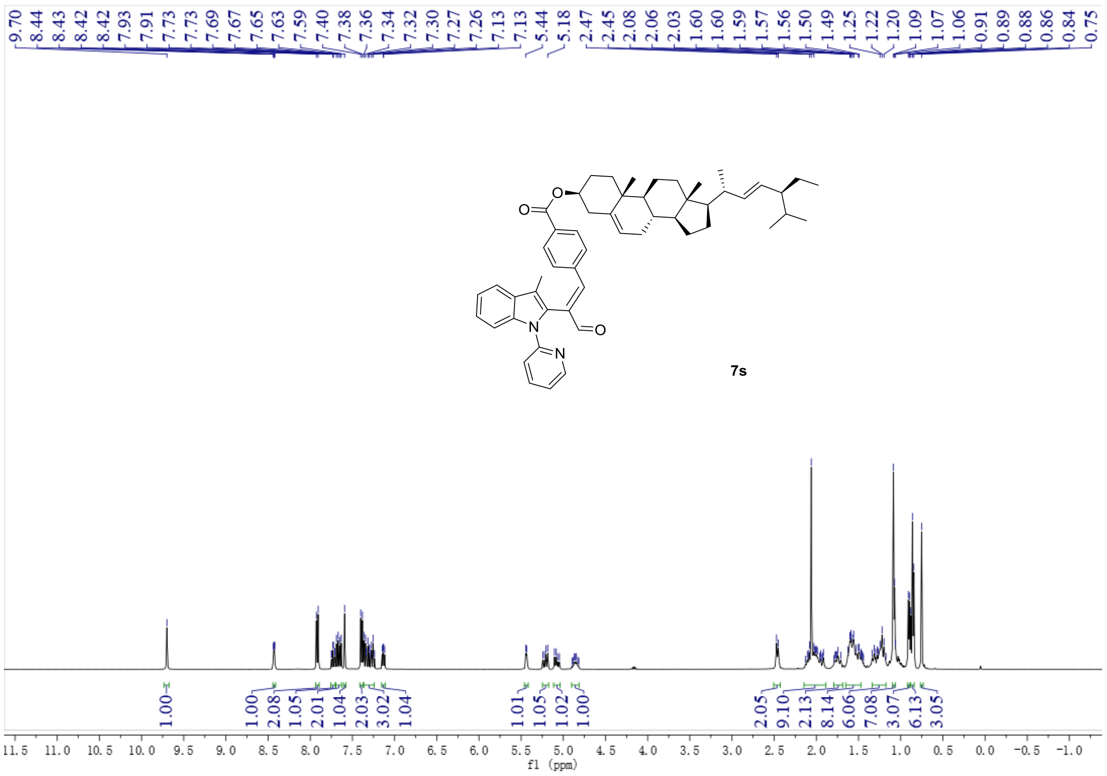

Supplementary Fig. 268 | ^{1}H NMR (400 MHz) of compound 7s (using $CDCl_3$ as solvent)

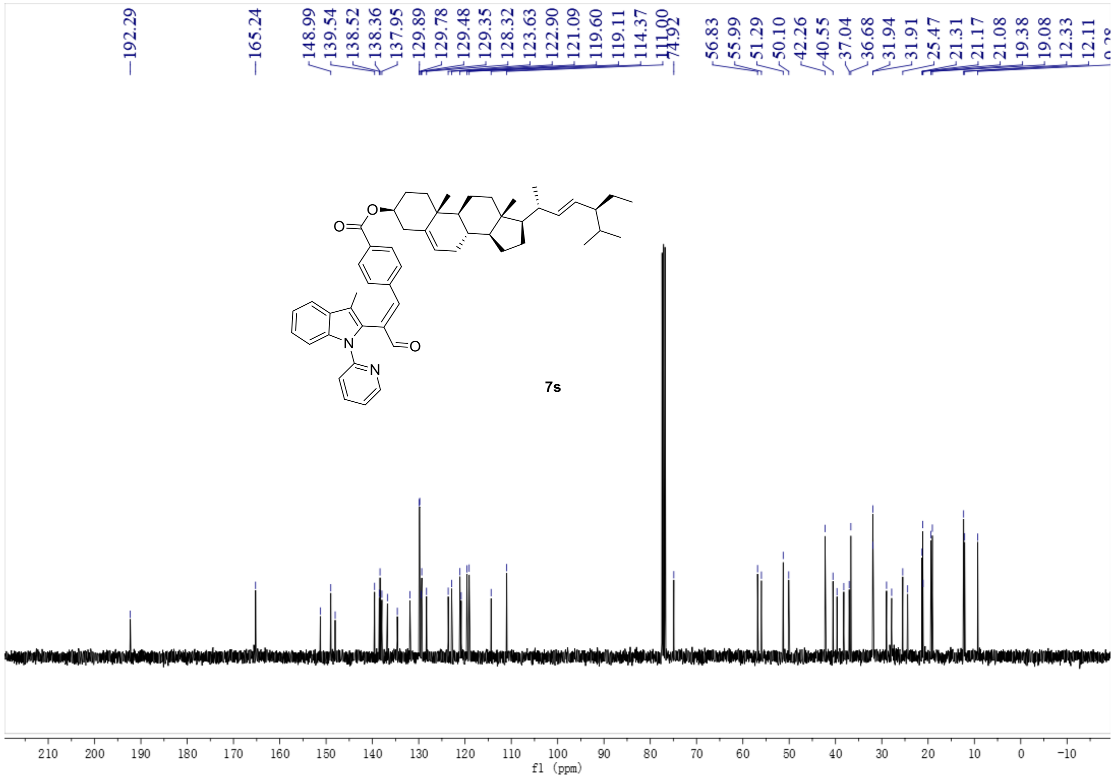

Supplementary Fig. 269 | ^{13}C NMR (101 MHz) of compound 7s (using $CDCl_3$ as solvent)

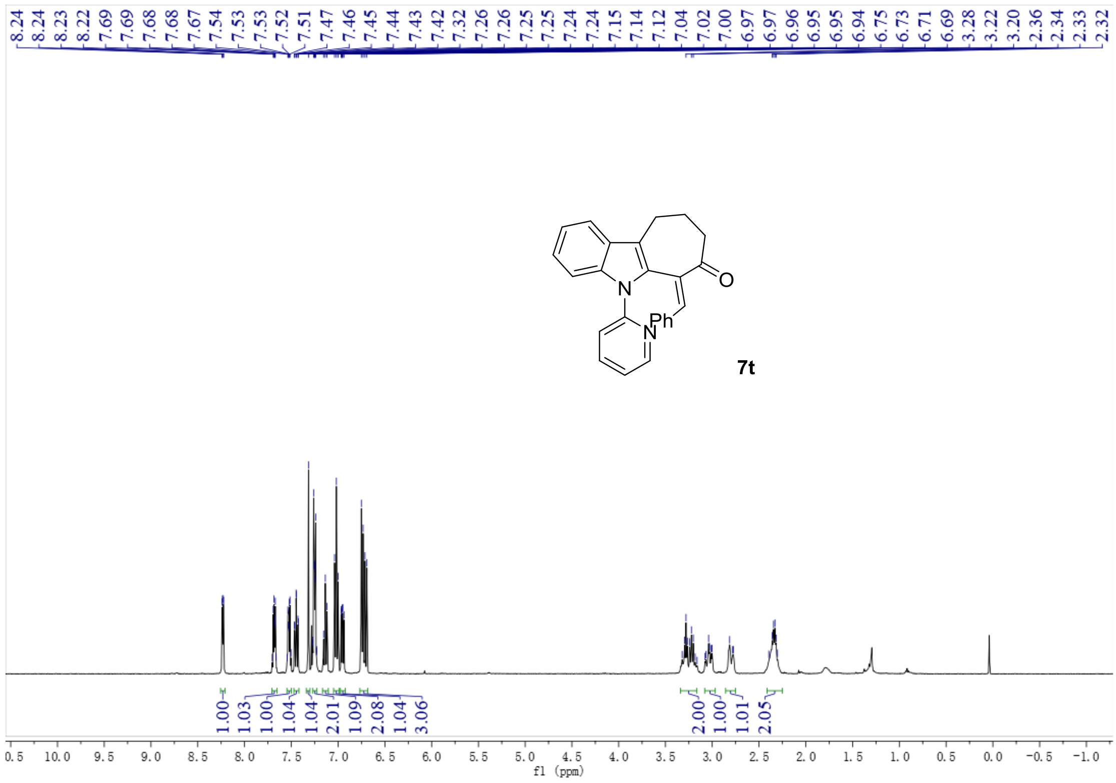

Supplementary Fig. 270 | ^{1}H NMR (400 MHz) of compound 7t (using $CDCl_3$ as solvent)

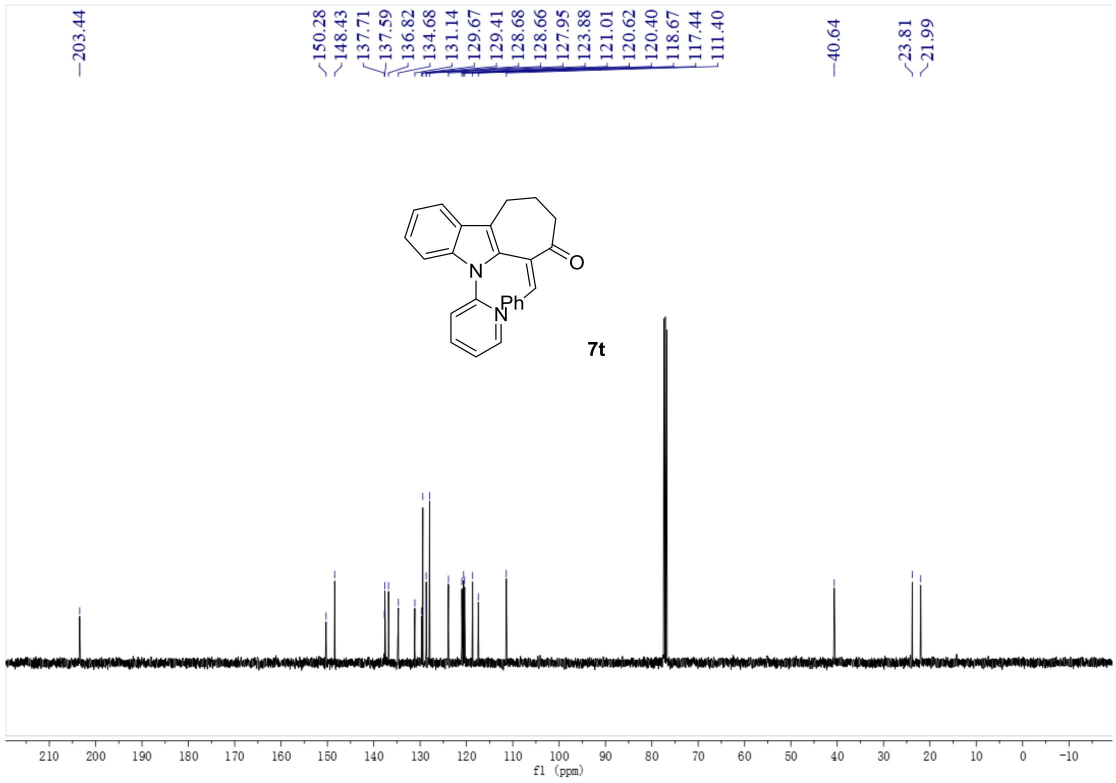

Supplementary Fig. 271 | ^{13}C NMR (101 MHz) of compound 7t (using $CDCl_3$ as solvent)

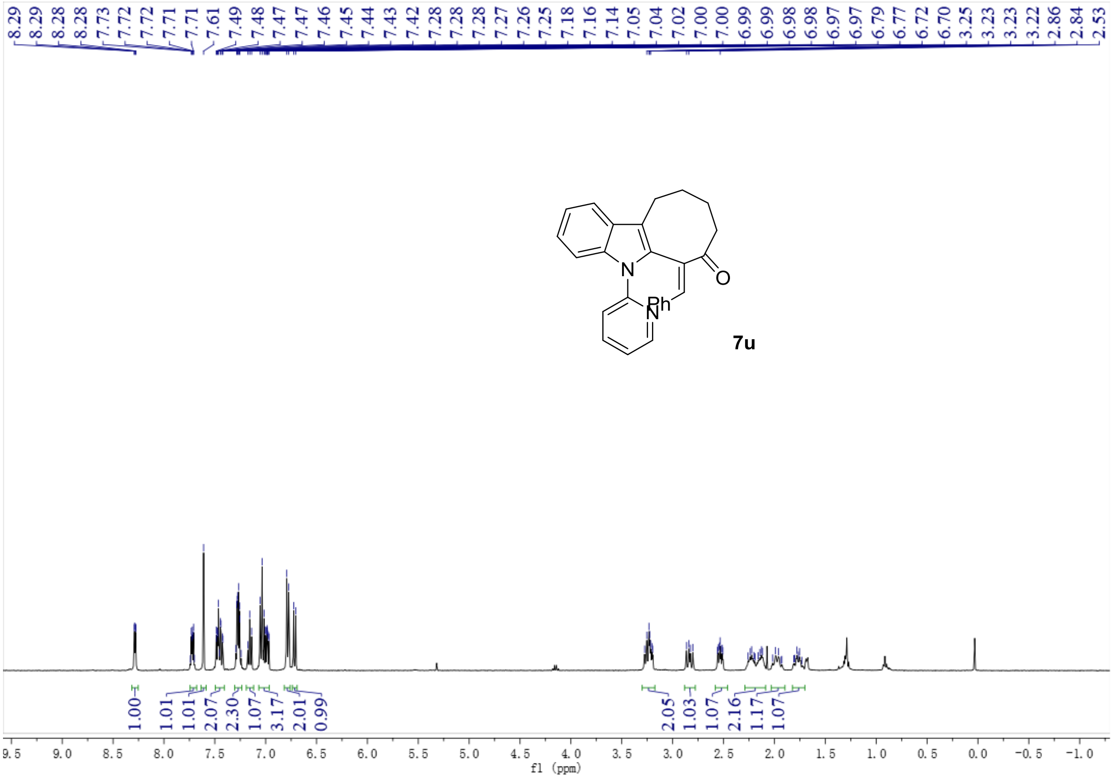

Supplementary Fig. 272 | ^{1}H NMR (400 MHz) of compound 7u (using $CDCl_3$ as solvent)

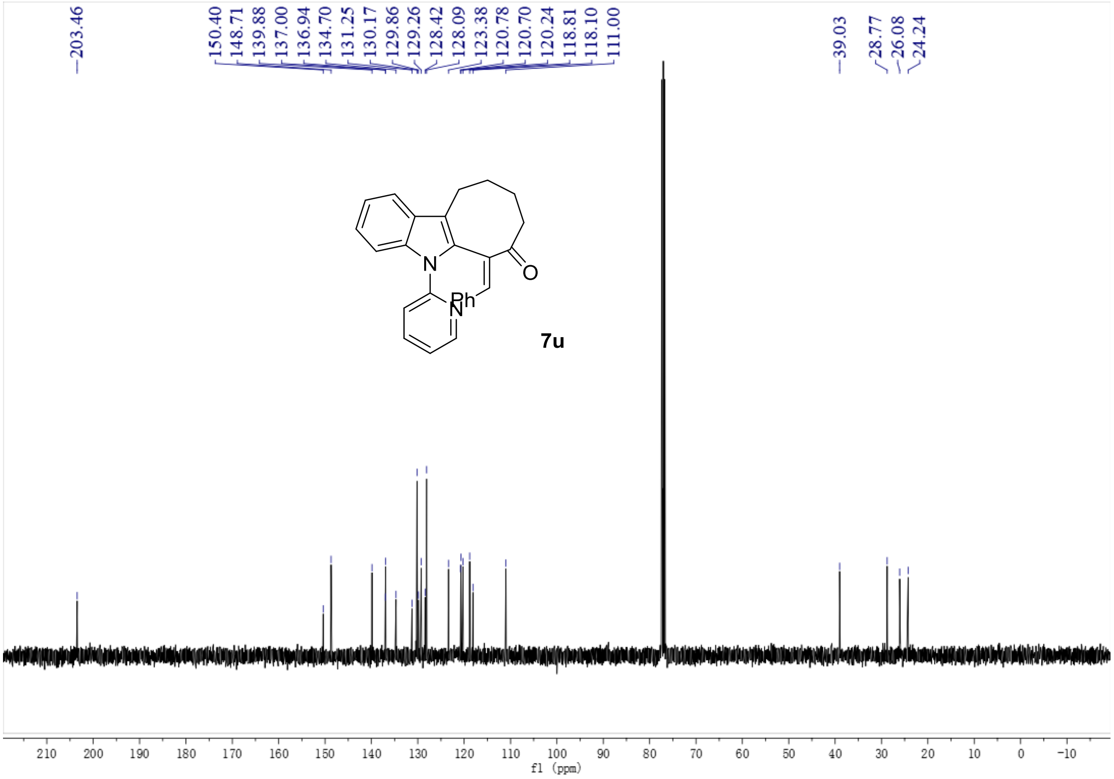

Supplementary Fig. 273 | ^{13}C NMR (101 MHz) of compound 7u (using $CDCl_3$ as solvent)

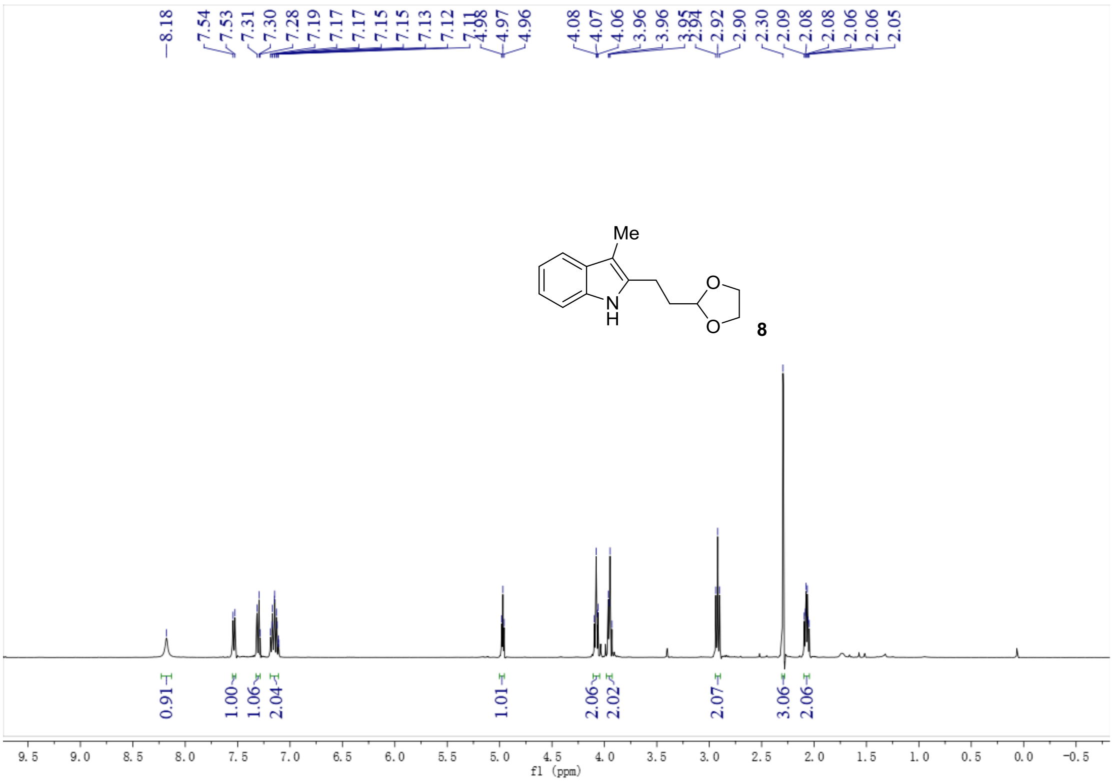

Supplementary Fig. 274 | ^{1}H NMR (400 MHz) of compound 8 (using $CDCl_3$ as solvent)

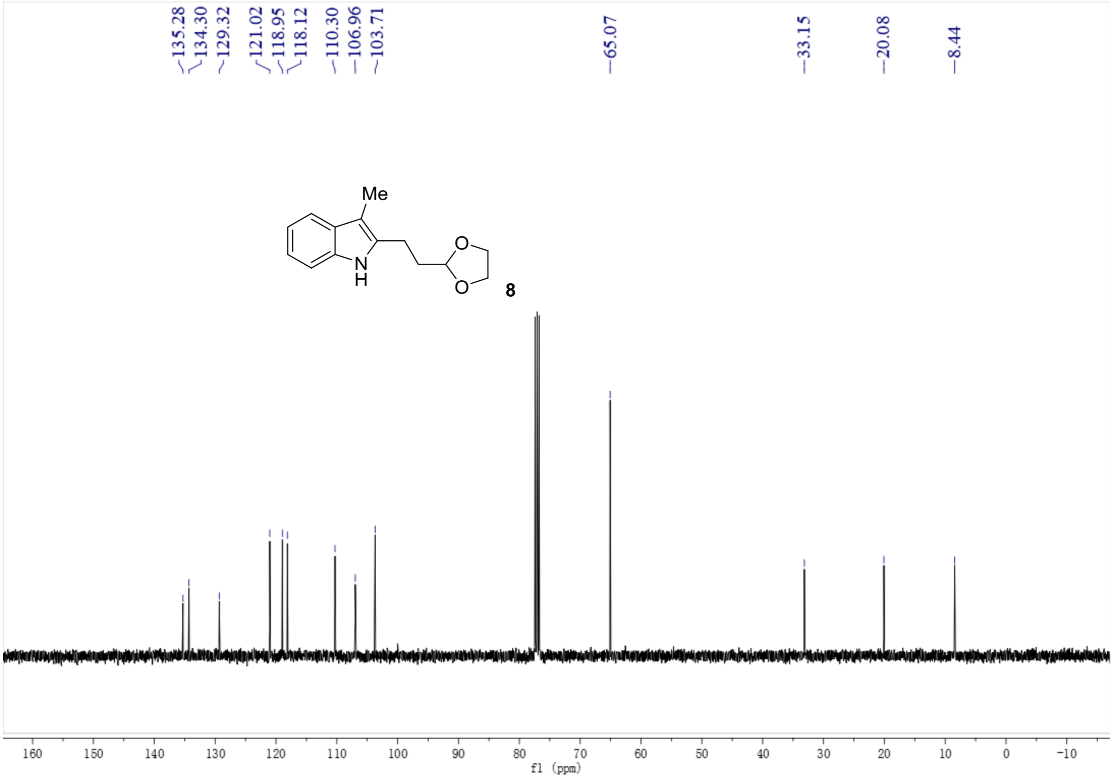

Supplementary Fig. 275 | ^{13}C NMR (101 MHz) of compound 8 (using $CDCl_3$ as solvent)

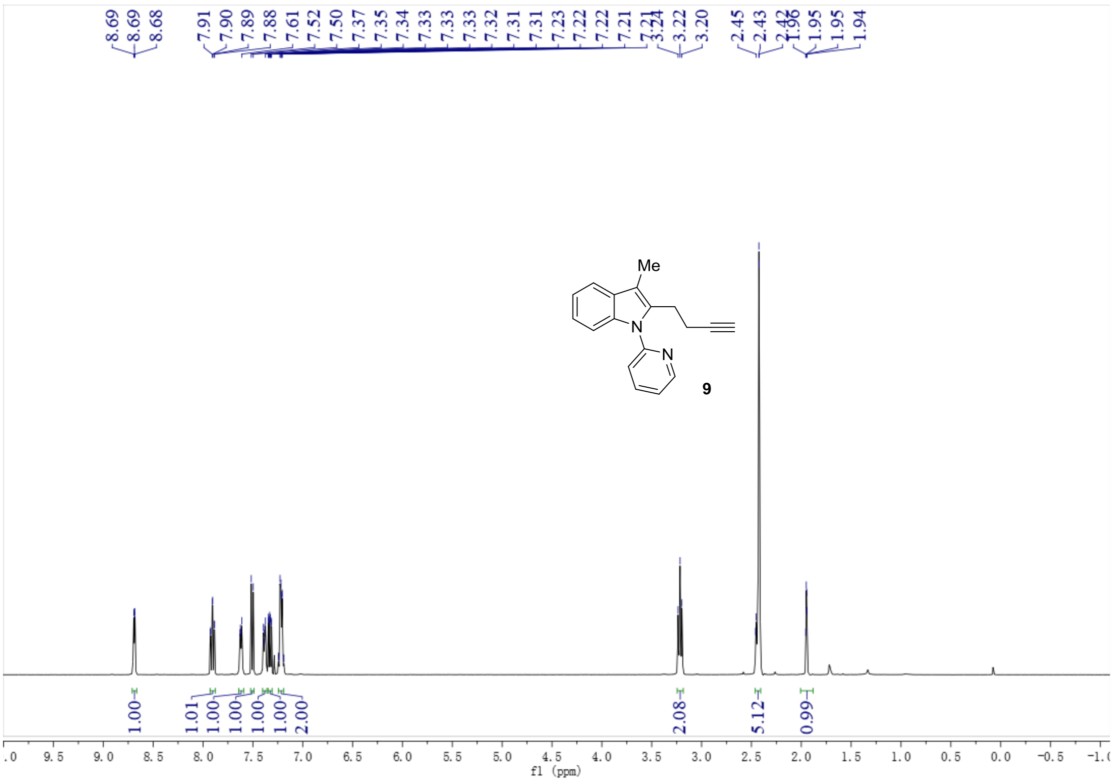

Supplementary Fig. 276 | ^{1}H NMR (400 MHz) of compound 9 (using $CDCl_3$ as solvent)

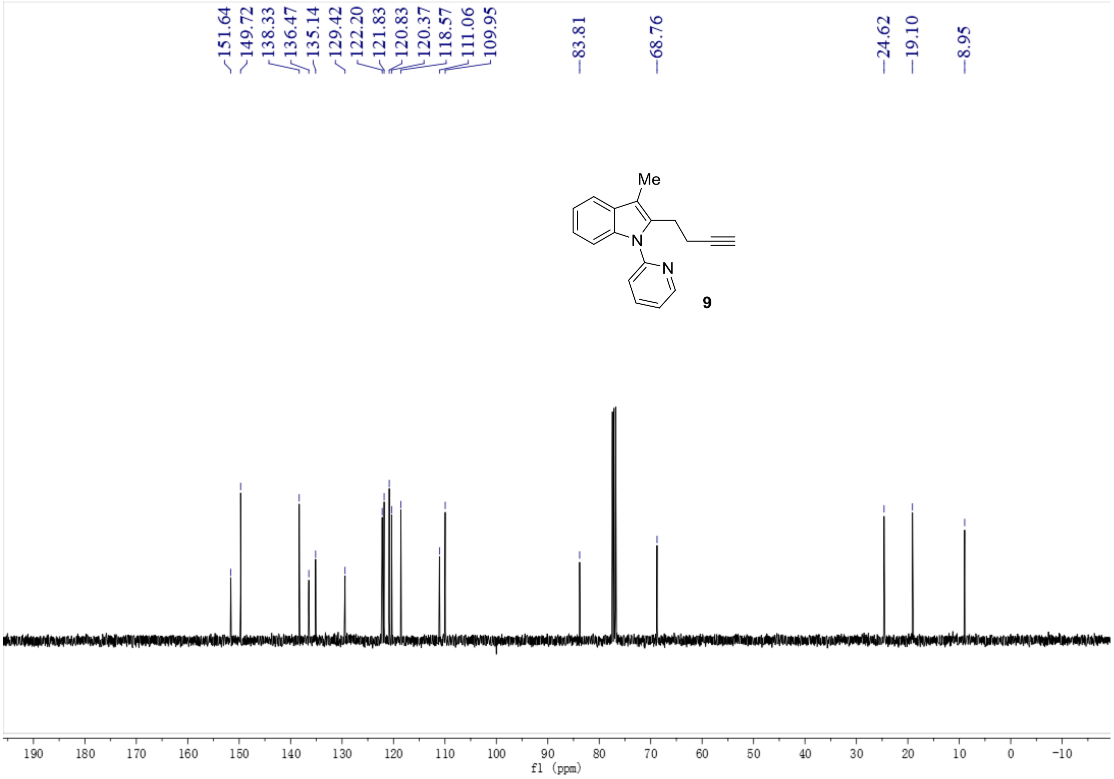

Supplementary Fig. 277 | ^{13}C NMR (101 MHz) of compound 9 (using $CDCl_3$ as solvent)

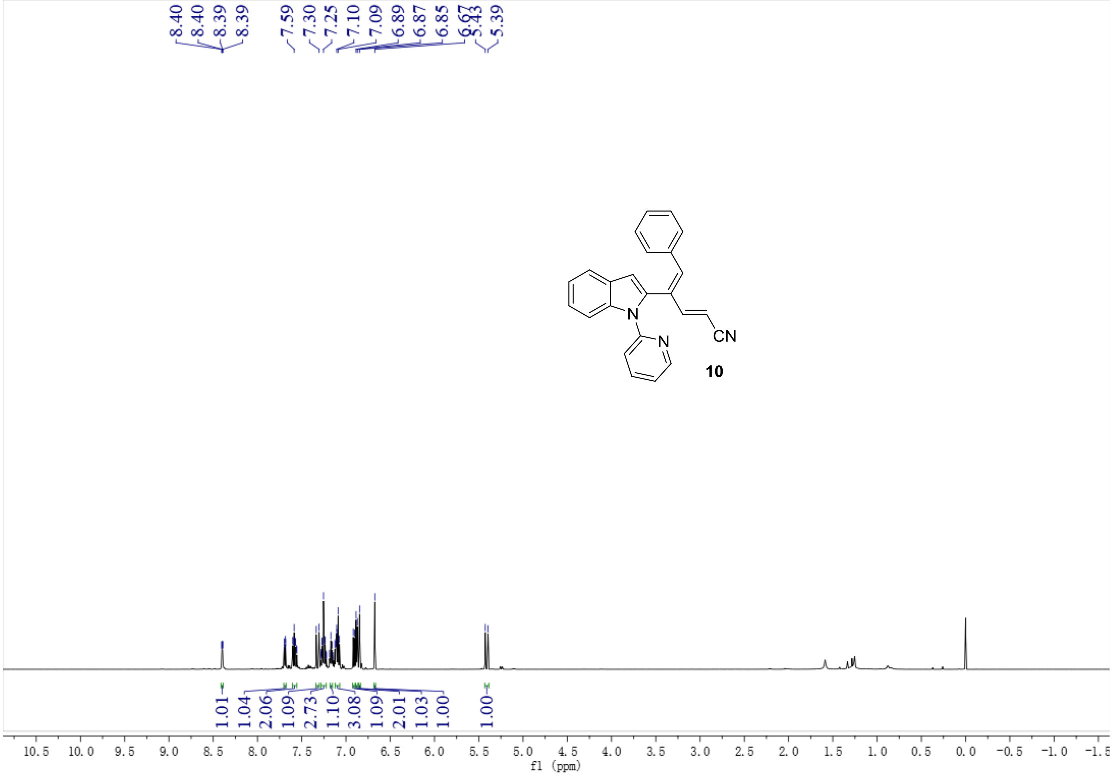

Supplementary Fig. 278 | ^{1}H NMR (500 MHz) of compound 10 (using $CDCl_3$ as solvent)

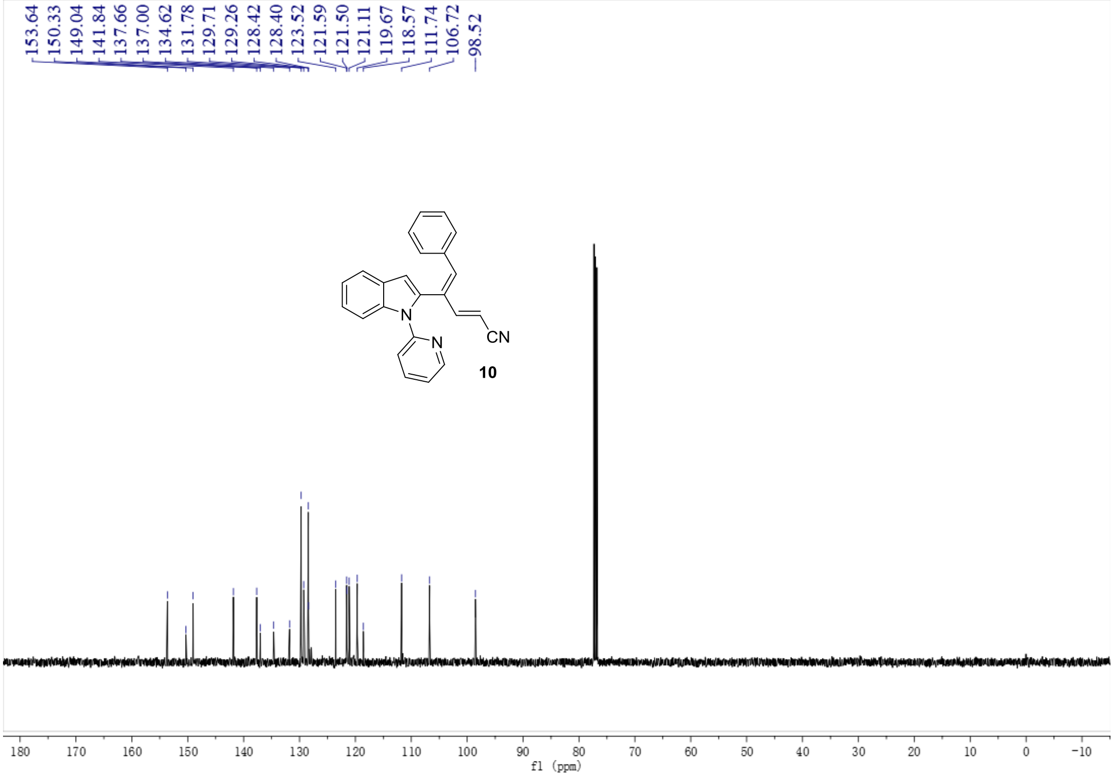

Supplementary Fig. 279 | ^{13}C NMR (126 MHz) of compound 10 (using $CDCl_3$ as solvent)

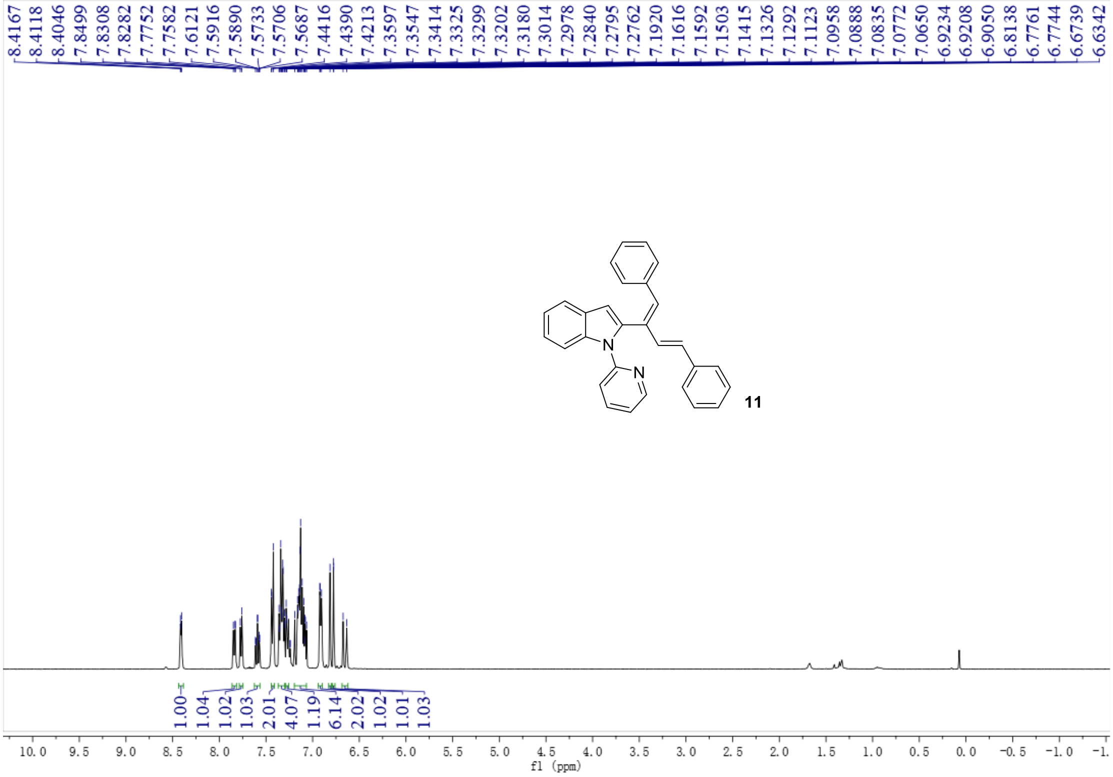

Supplementary Fig. 280 | ^{1}H NMR (400 MHz) of compound 11 (using $CDCl_3$ as solvent)

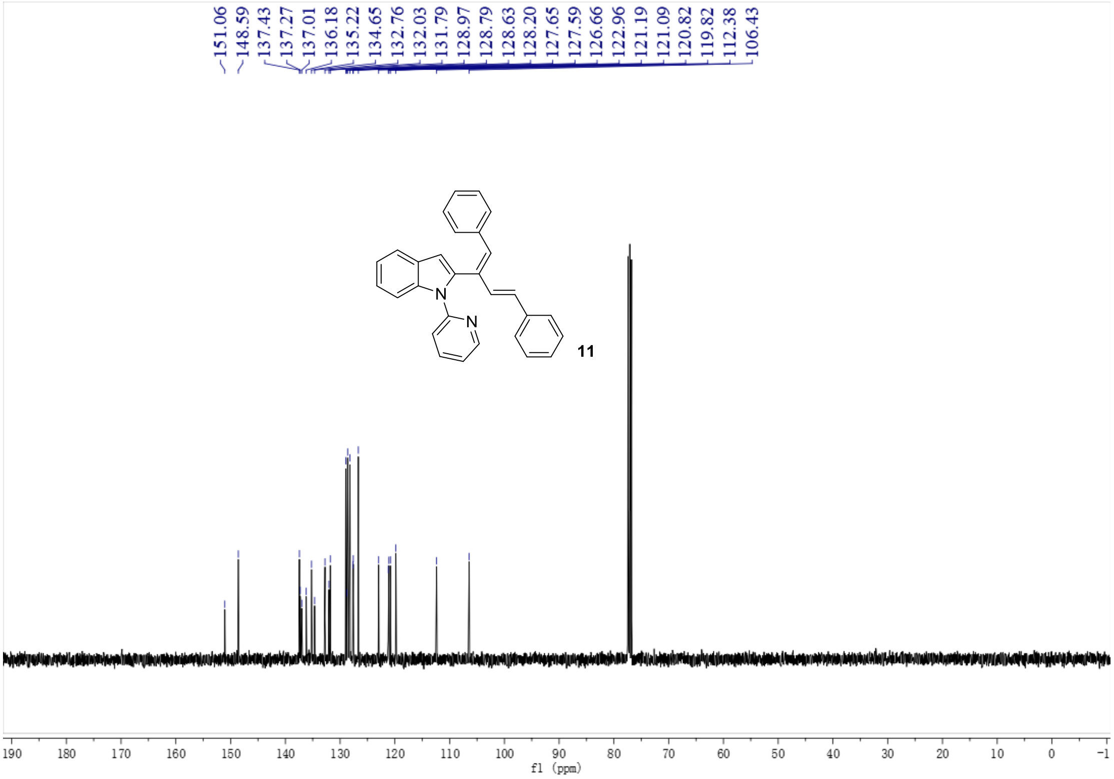

Supplementary Fig. 281 | ^{13}C NMR (101 MHz) of compound 11 (using $CDCl_3$ as solvent)

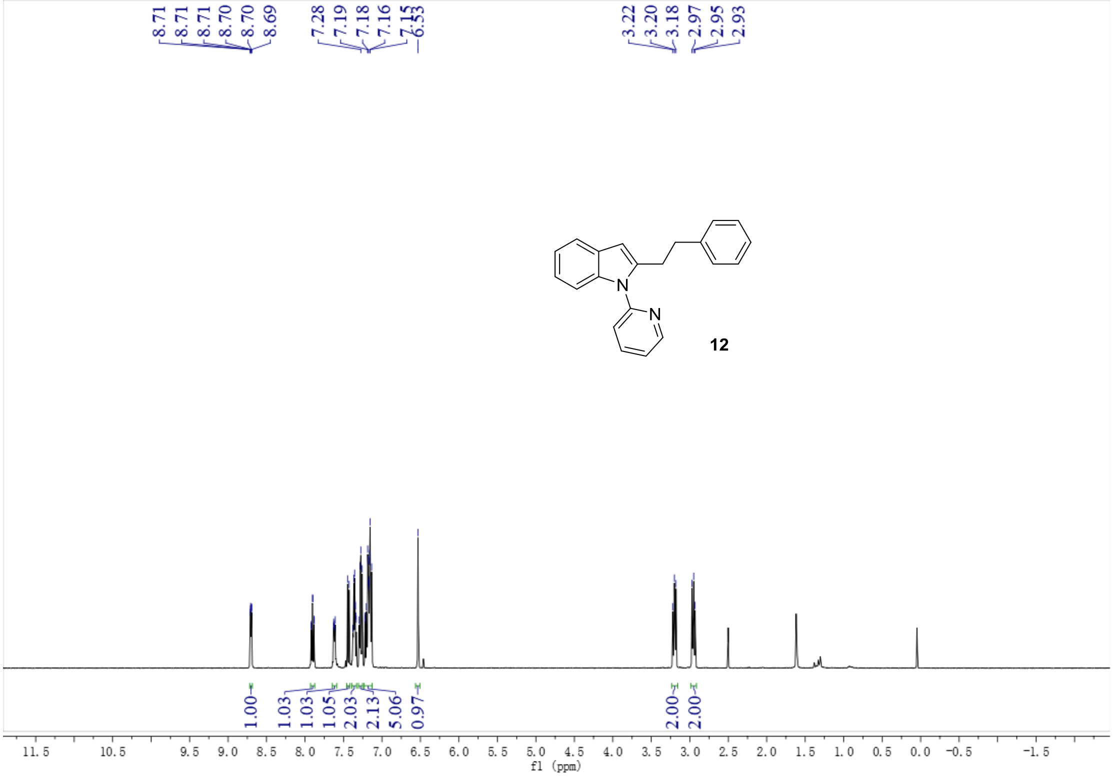

Supplementary Fig. 282 | ^{1}H NMR (400 MHz) of compound 12 (using $CDCl_3$ as solvent)

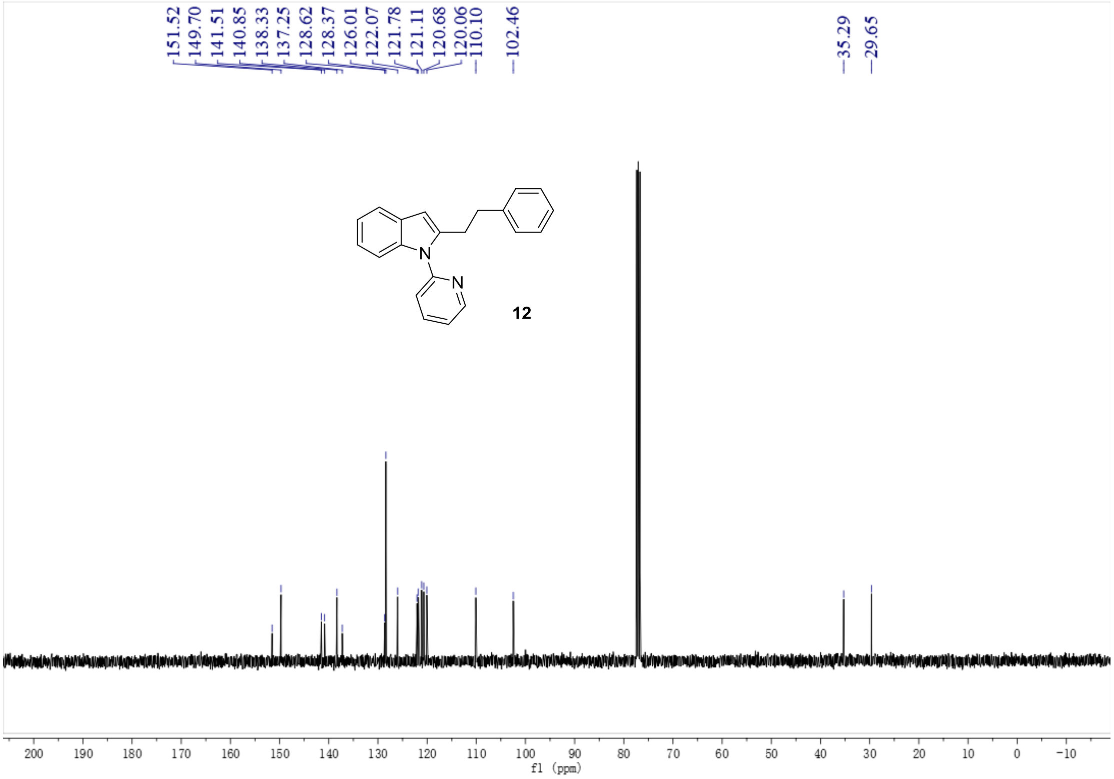

Supplementary Fig. 283 | ^{13}C NMR (101 MHz) of compound 12 (using $CDCl_3$ as solvent)

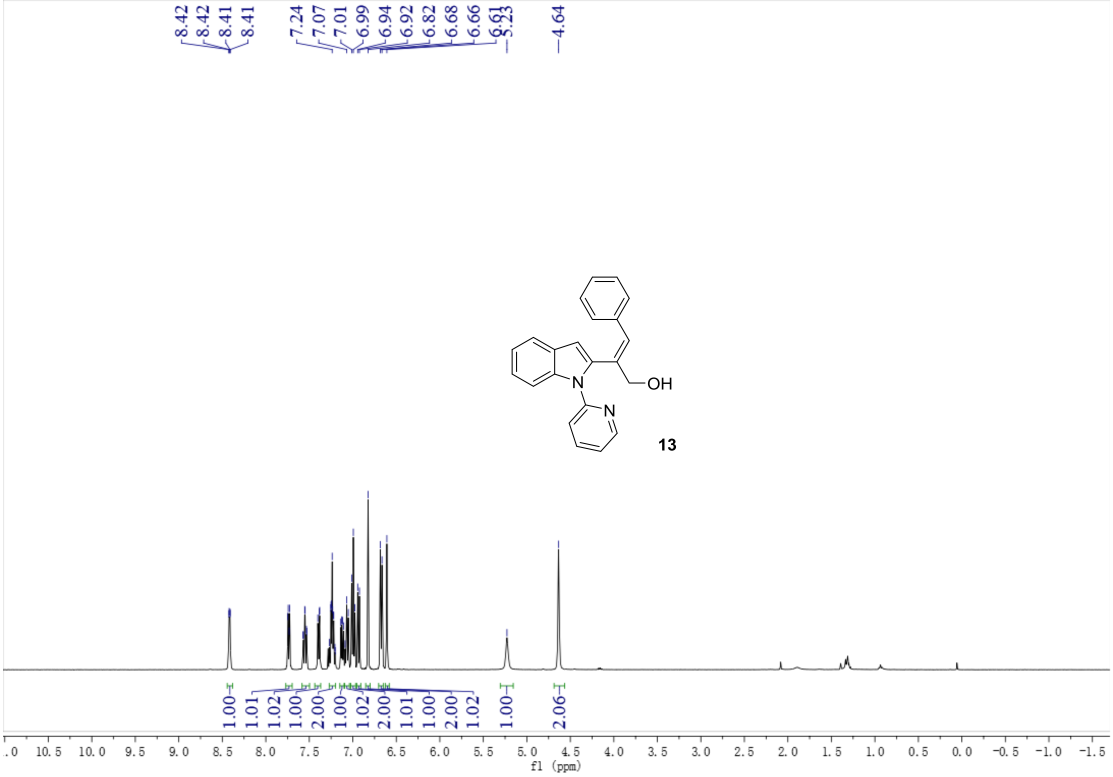

Supplementary Fig. 284 | ^{1}H NMR (400 MHz) of compound 13 (using $CDCl_3$ as solvent)

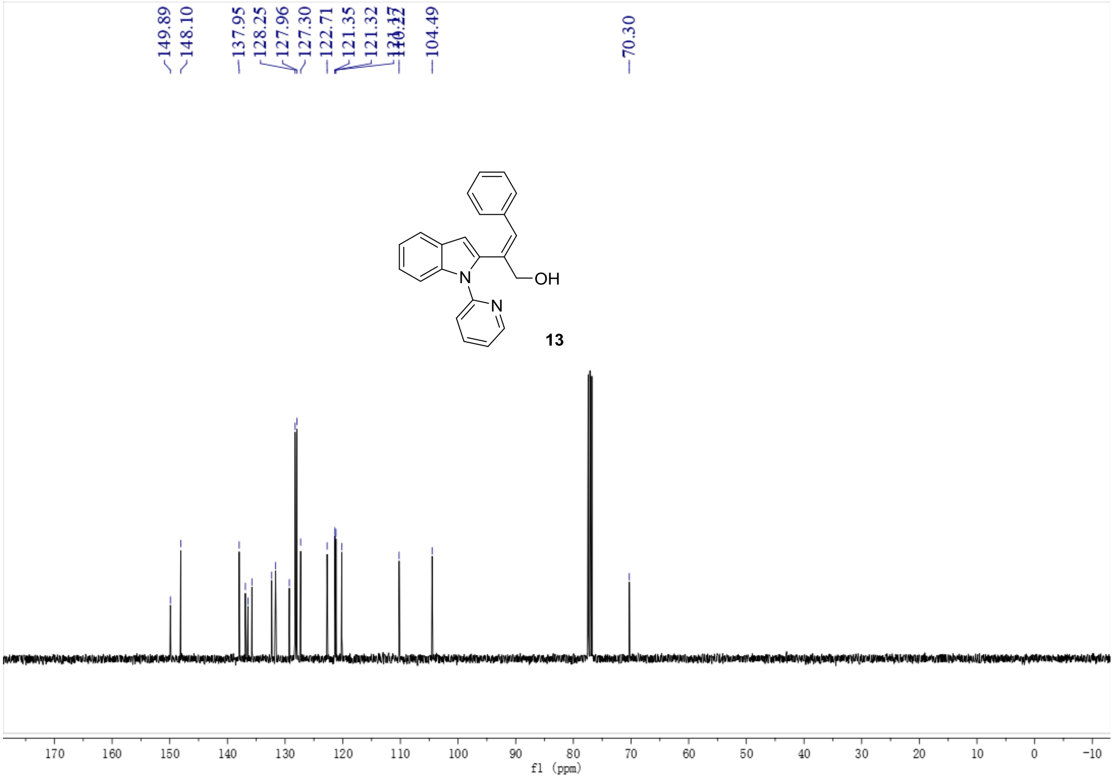

Supplementary Fig. 285 | ^{13}C NMR (101 MHz) of compound 13 (using $CDCl_3$ as solvent)

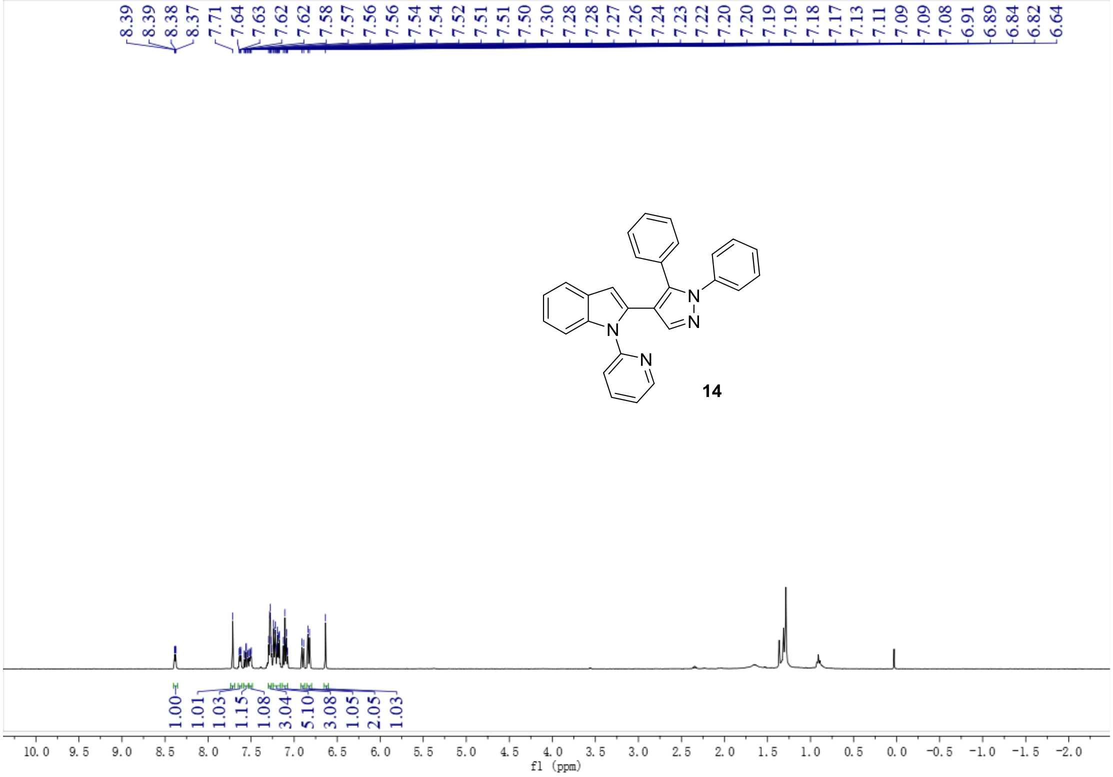

Supplementary Fig. 286 | ^{1}H NMR (400 MHz) of compound 14 (using $CDCl_3$ as solvent)

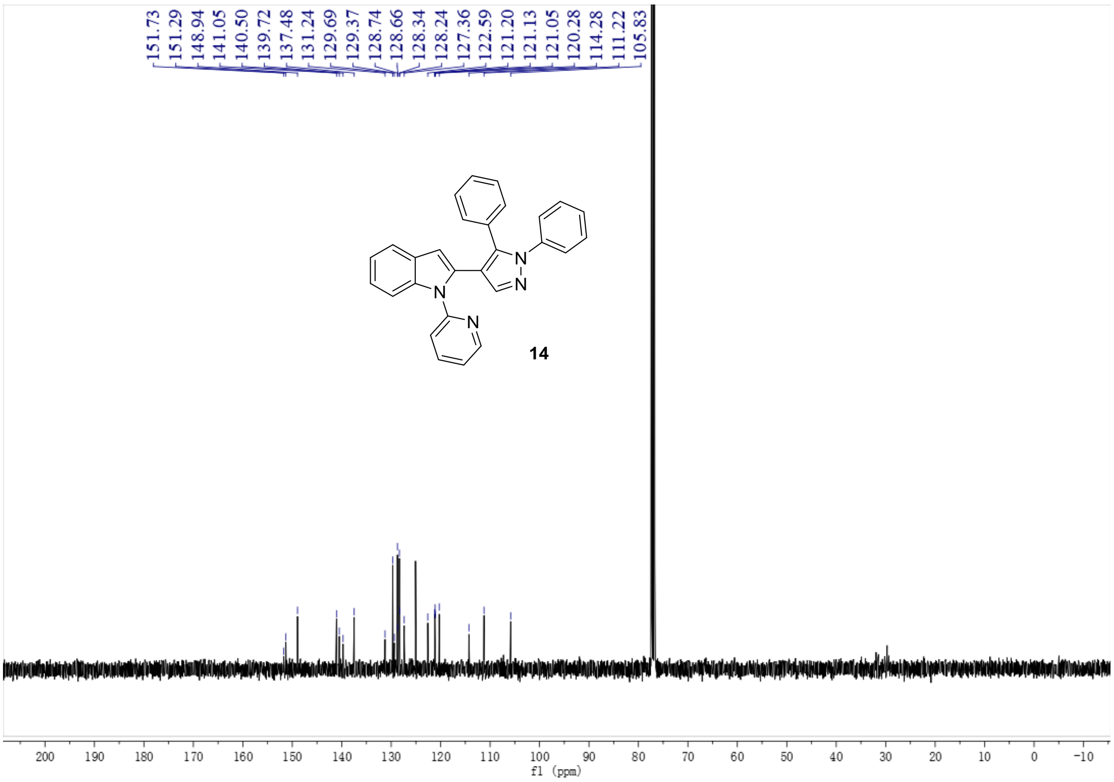

Supplementary Fig. 287 | ^{13}C NMR (101 MHz) of compound 14 (using $CDCl_3$ as solvent)

III. HR-MS Spectrum of the New Products

HR-MS spectrum of S1-1

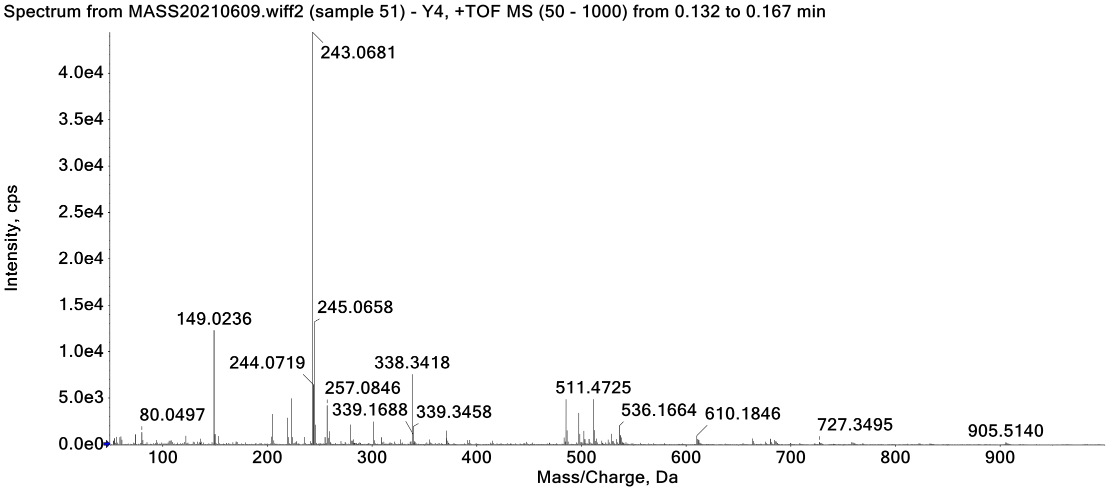

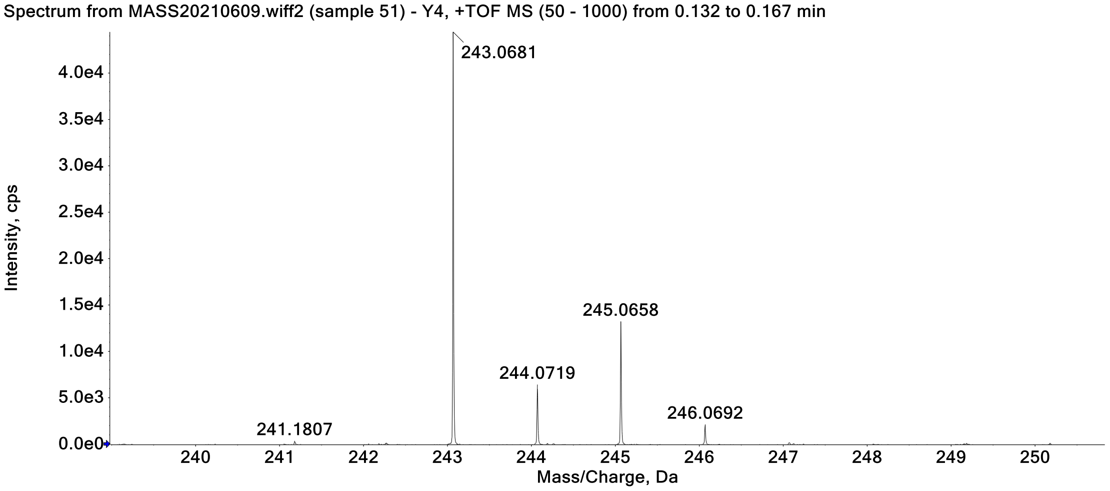

Hit	Formula	m/z	RDB	ppm	MS Rank	MSMS ppm	MSMS Rank	Found
1	C14H11ClN2	243.0684	10.0	-1.0	1			NA/NA

HR-MS spectrum of S1-2

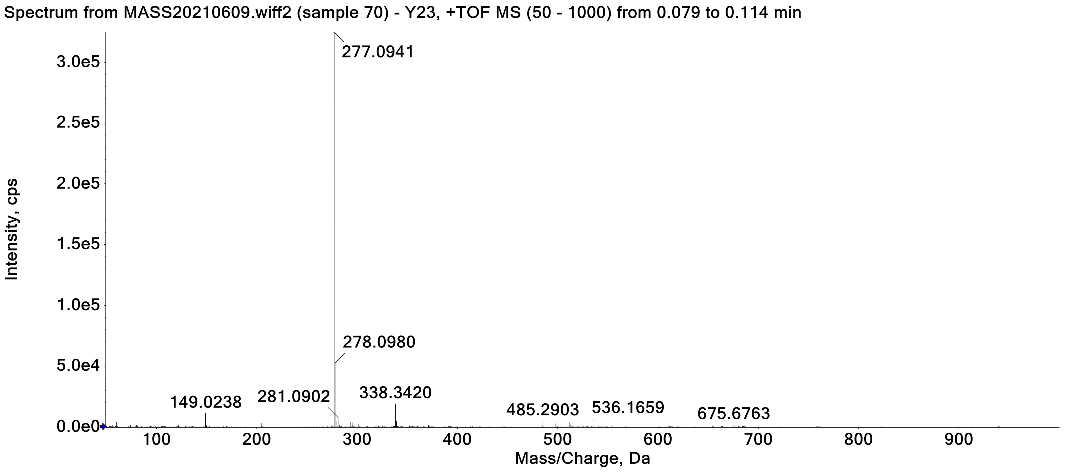

Spectrum from MASS20210609.wiff2 (sample 70) - Y23, +TOF MS (50 - 1000) from 0.079 to 0.114 min

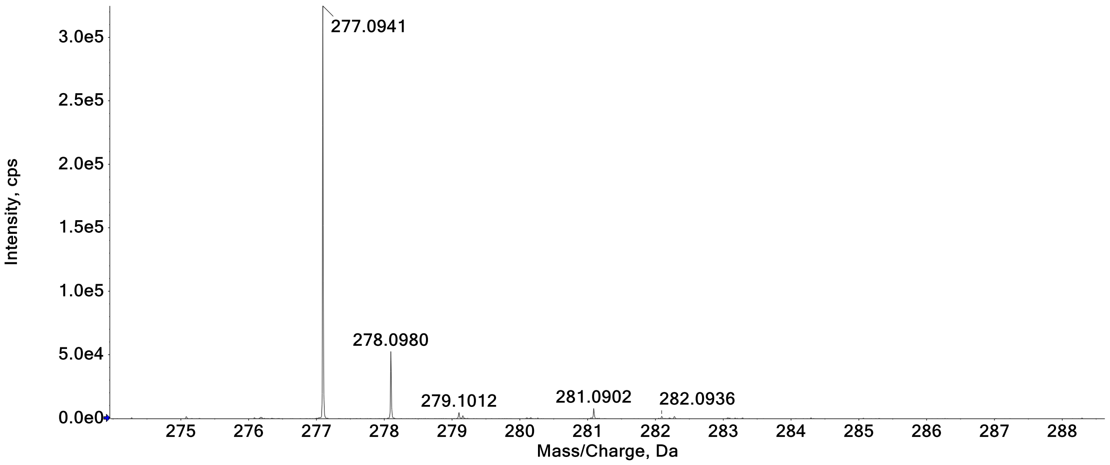

Spectrum from MASS20210609.wiff2 (sample 70) - Y23, +TOF MS (50 - 1000) from 0.079 to 0.114 min

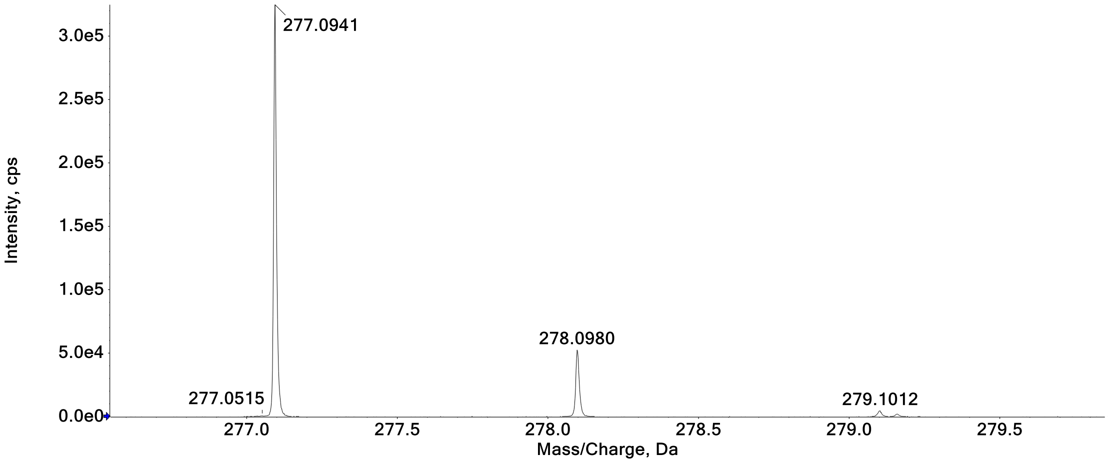

Hit	Formula	m/z	RDB	ppm	MS Rank	MSMS ppm	MSMS Rank	Found
1	C15H11F3N2	277.0947	10.0	-2.2	1			NA/NA

HR-MS spectrum of S1-3

Spectrum from MASS20211013.wiff2 (sample 38) - Y14, +TOF MS (50 - 1000) from 0.114 to 0.184 min

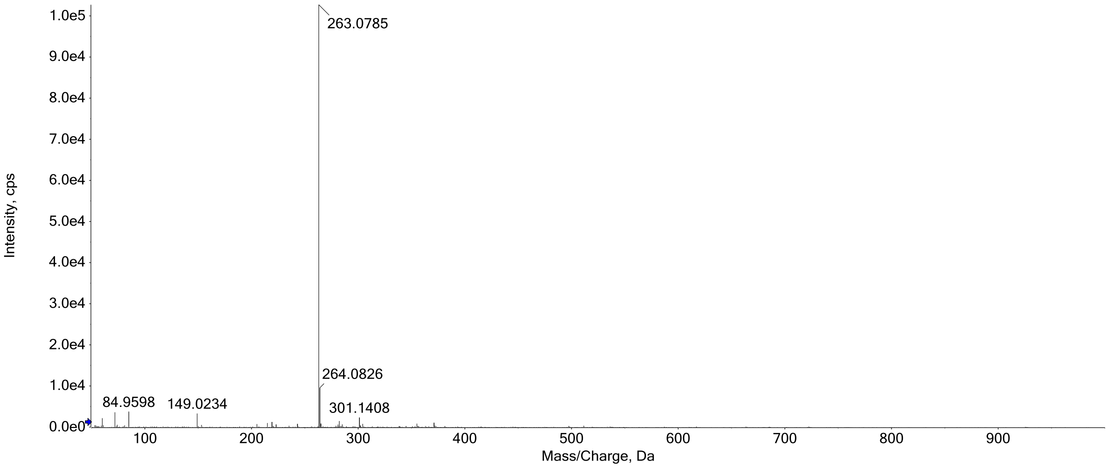

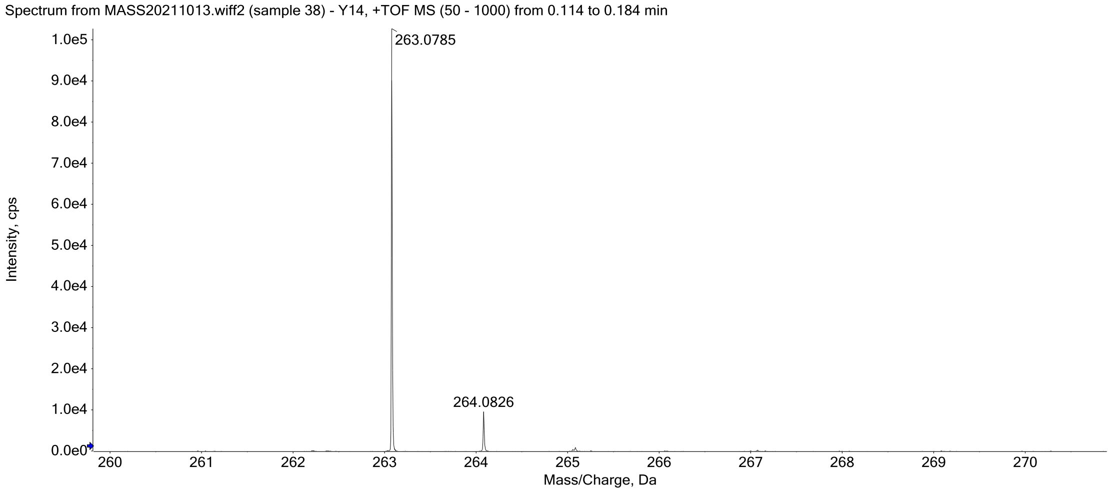

Hit	Formula	m/z	RDB	ppm	MS Rank	MSMS ppm	MSMS Rank	Found
1	C14H9F3N2	263.0791	10.0	-2.1	1			NA/NA

HR-MS spectrum of S2-1

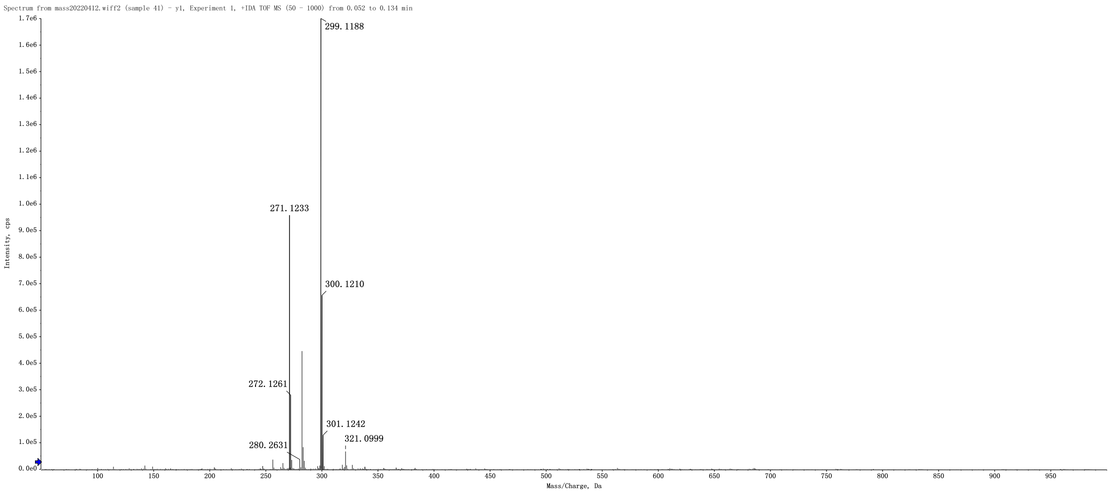

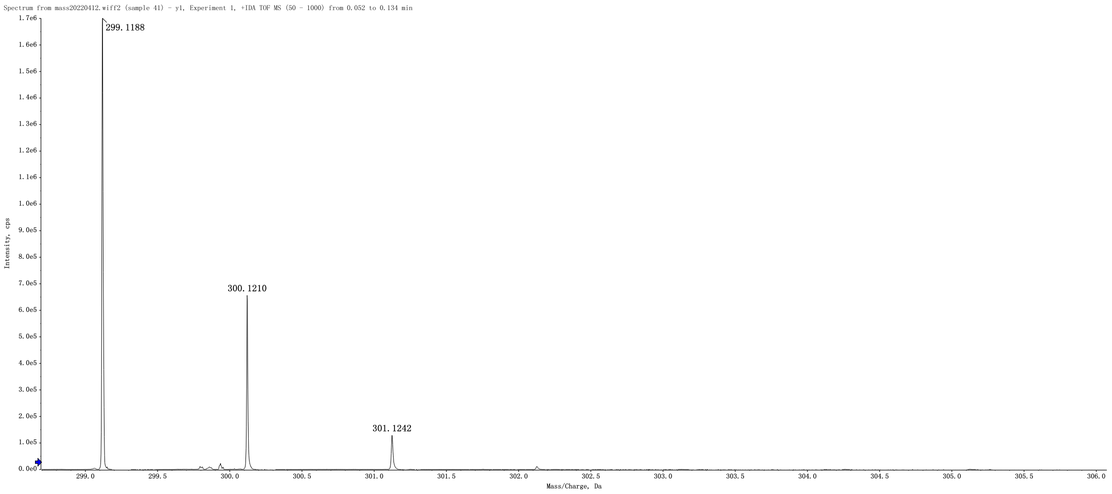

Hit	Formula	m/z	RDB	ppm	MS Rank	MSMS ppm	MSMS Rank	Found
1	C20H14N2O	299.1179	15.0	3.0	1			NA/NA

HR-MS spectrum of S2-2

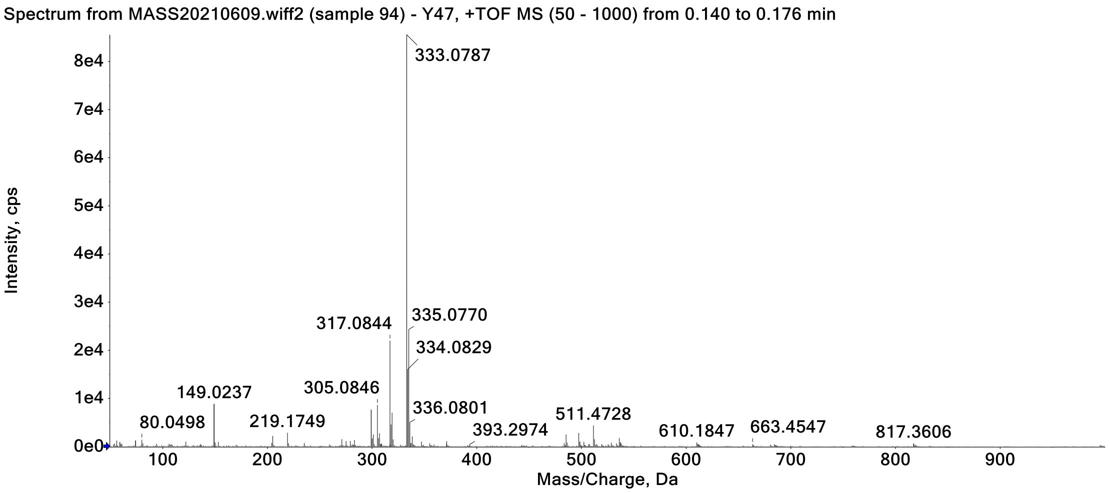

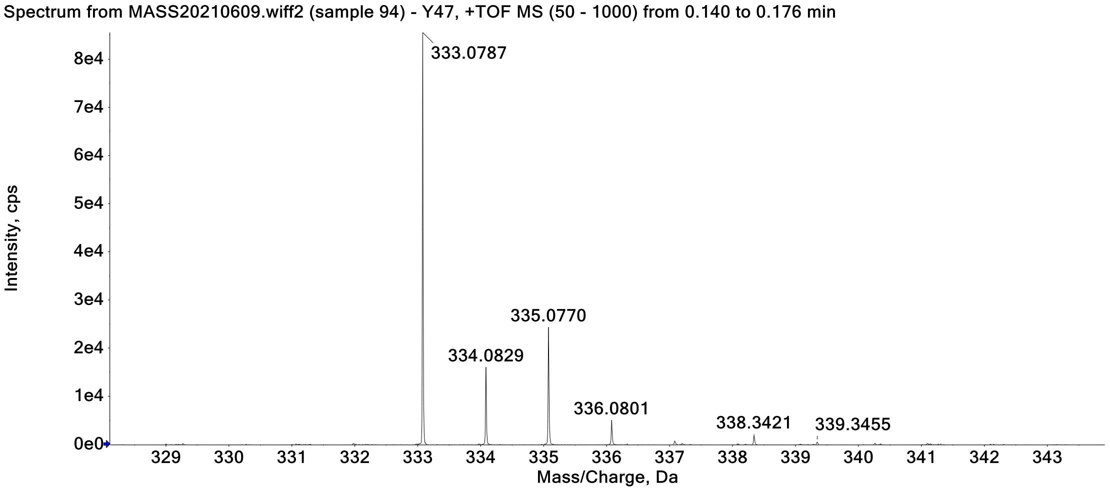

Hit	Formula	m/z	RDB	ppm	MS Rank	MSMS ppm	MSMS Rank	Found
1	C20H13ClN2O	333.0789	15.0	-0.7	1			NA/NA

HR-MS spectrum of S2-3

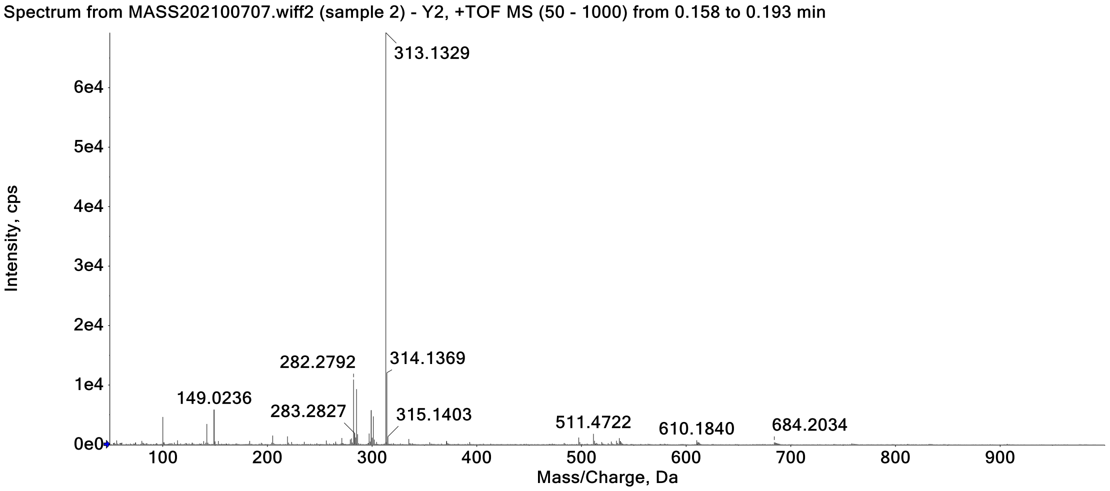

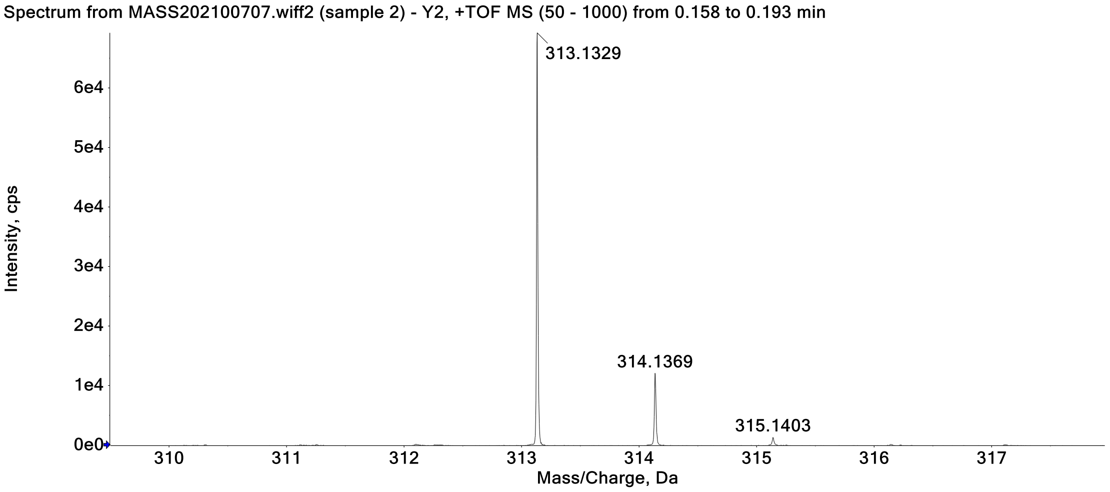

Hit	Formula	m/z	RDB	ppm	MS Rank	MSMS ppm	MSMS Rank	Found
1	C21H16N2O	313.1335	15.0	-2.0	1			NA/NA

HR-MS spectrum of S2-4

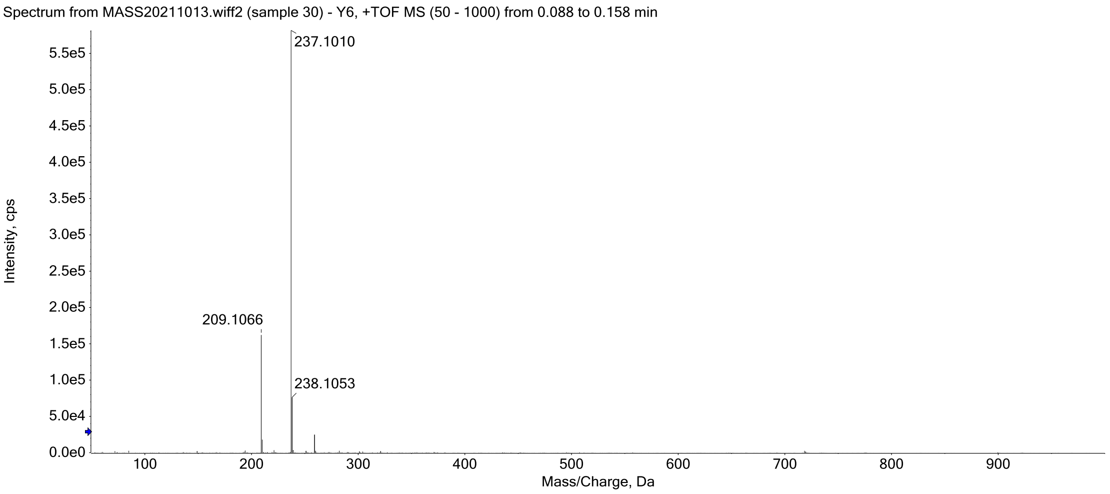

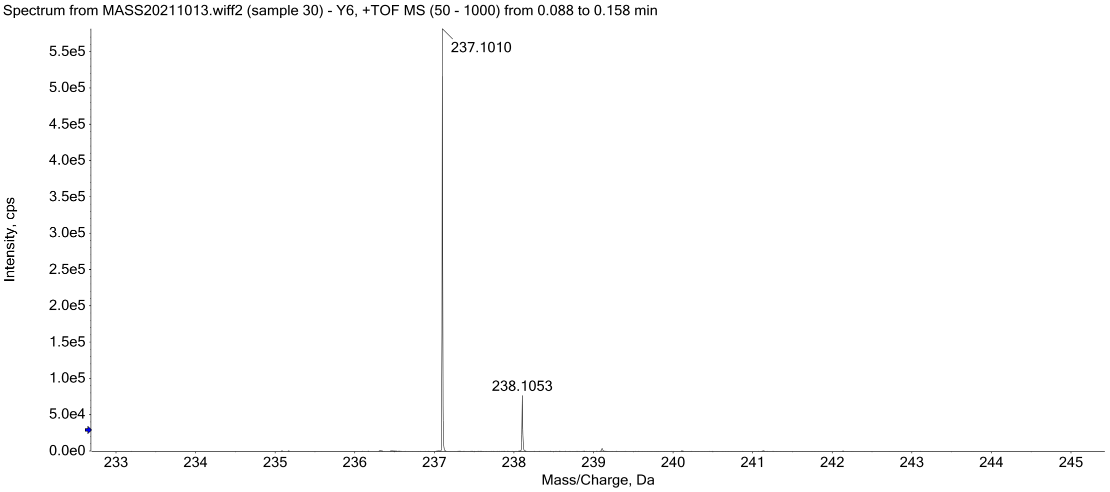

Hit	Formula	m/z	RDB	ppm	MS Rank	MSMS ppm	MSMS Rank	Found
1	C15H12N2O	237.1022	11.0	-3.1	1			NA/NA

HR-MS spectrum of S2-5

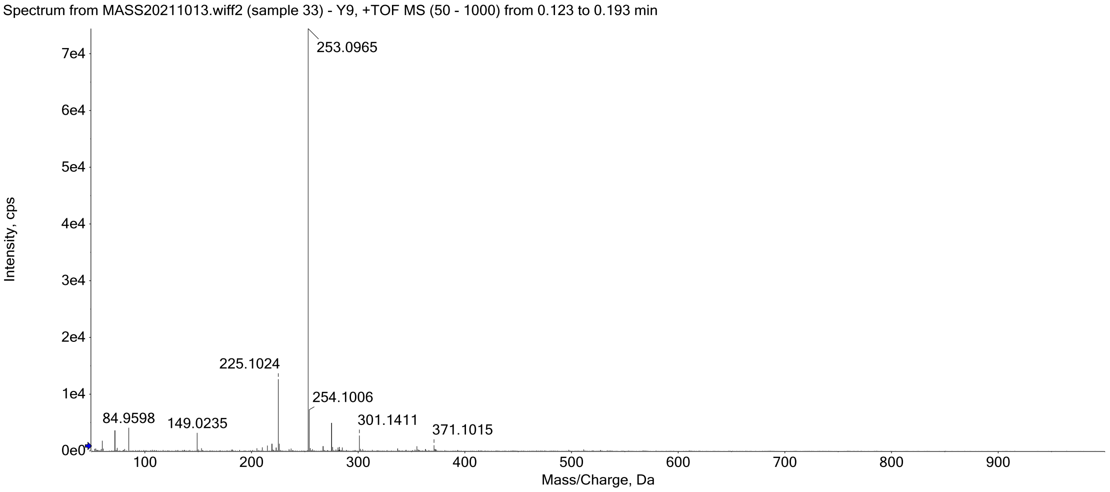

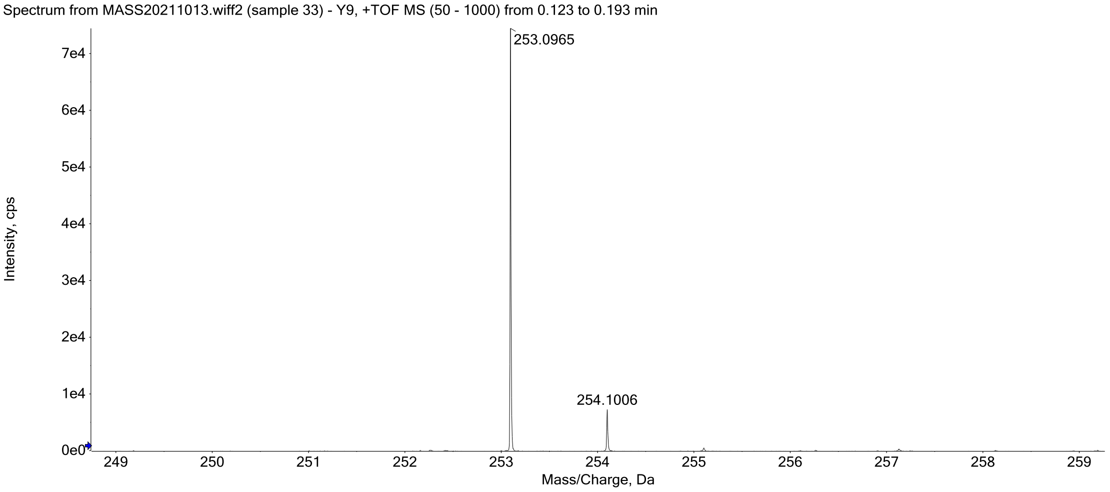

Hit	Formula	m/z	RDB	ppm	MS Rank	MSMS ppm	MSMS Rank	Found
1	C15H12N2O2	253.0972	11.0	-2.6	1			NA/NA

HR-MS spectrum of S2-6

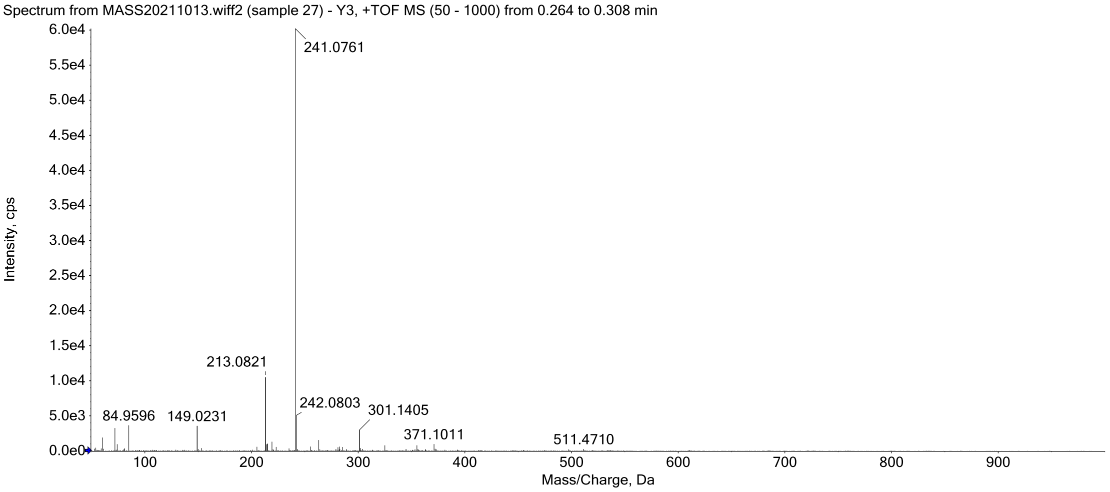

Spectrum from MASS20211013.wiff2 (sample 27) - Y3, +TOF MS (50 - 1000) from 0.264 to 0.308 min

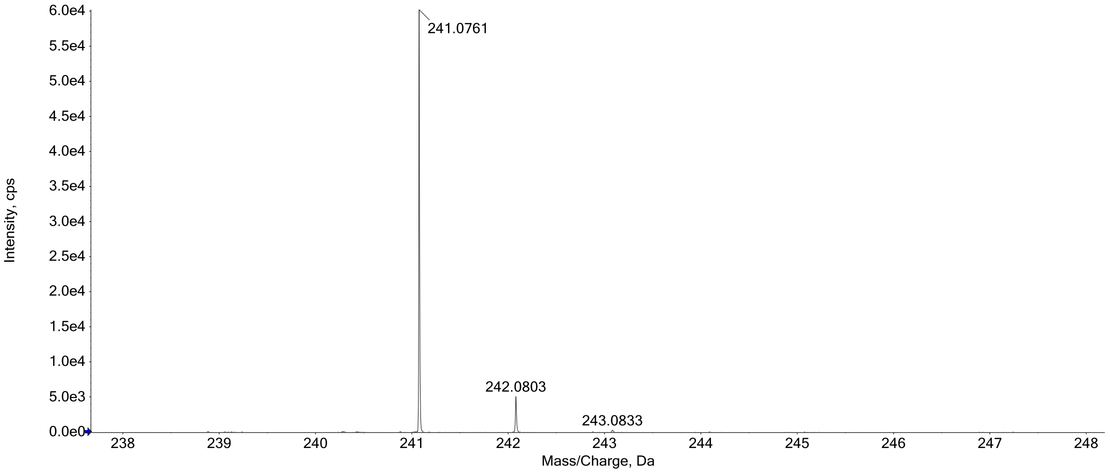

Spectrum from MASS20211013.wiff2 (sample 27) - Y3, +TOF MS (50 - 1000) from 0.0...S20211013.wiff2 (sample 27) - Y3, +TOF MS (50 - 1000) from 0.184 to 0.211 min]

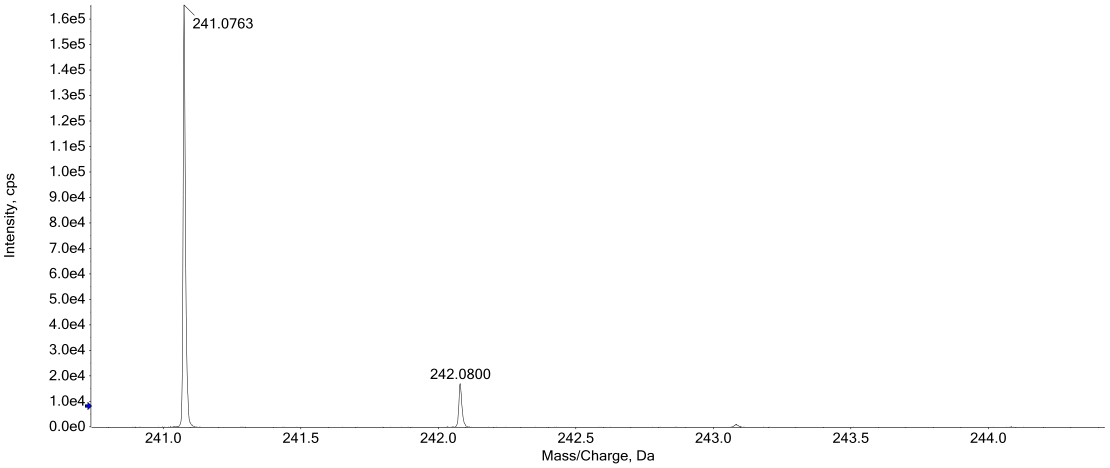

Hit	Formula	m/z	RDB	ppm	MS Rank	MSMS ppm	MSMS Rank	Found
1	C14H9FN2O	241.0772	11.0	-4.4	1			NA/NA

HR-MS spectrum of S2-7

Spectrum from MASS20211013.wiff2 (sample 39) - Y15, +TOF MS (50 - 1000) from 0.114 to 0.184 min

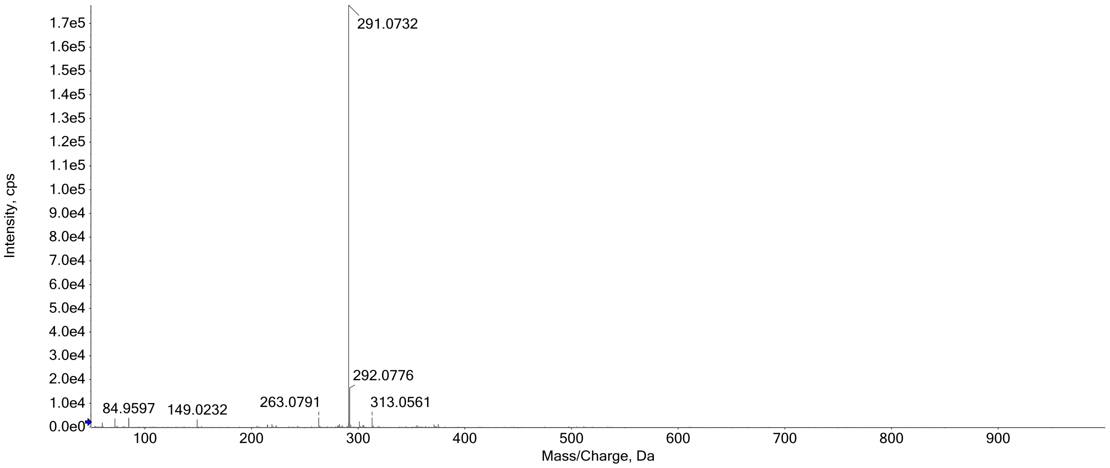

Spectrum from MASS20211013.wiff2 (sample 39) - Y15, +TOF MS (50 - 1000) from 0.114 to 0.184 min

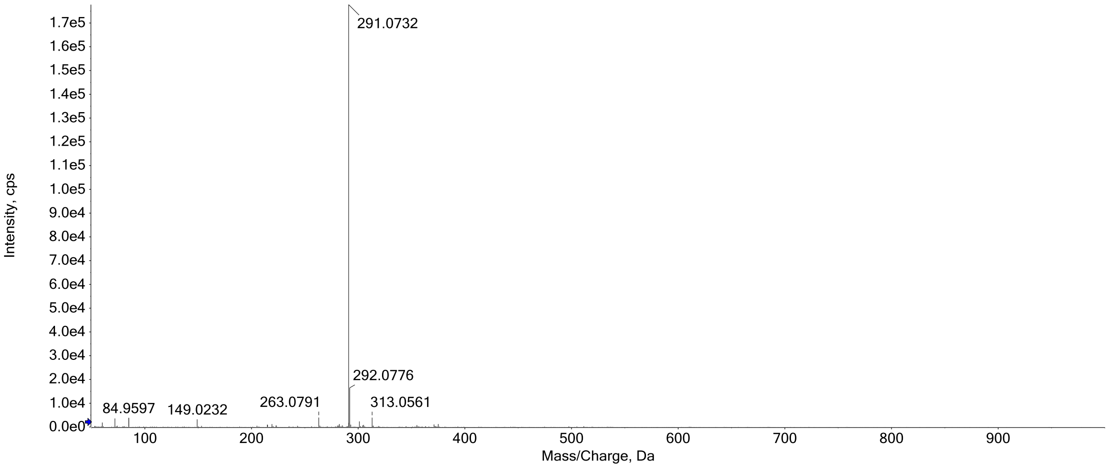

Spectrum from MASS20211013.wiff2 (sample 39) - Y15, +TOF MS (50 - 1000) from 0.114 to 0.184 min

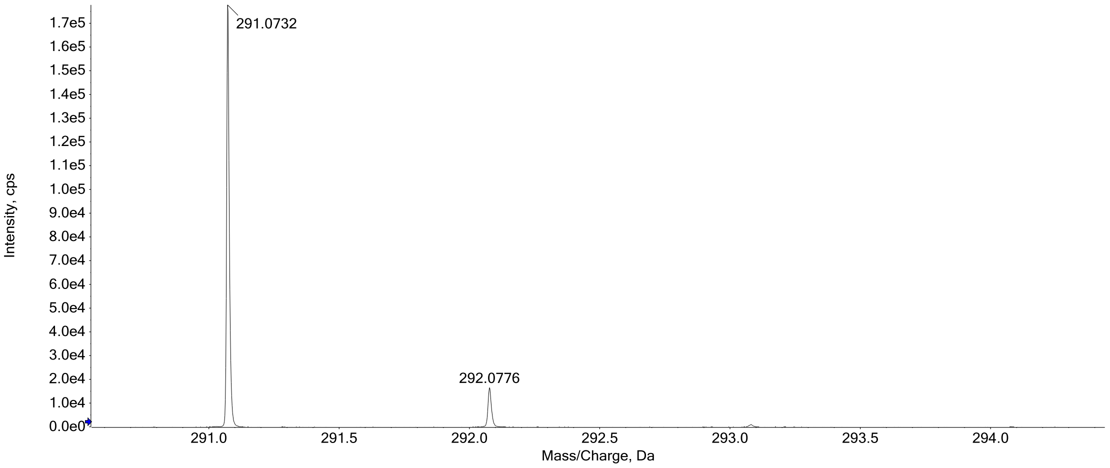

Hit	Formula	m/z	RDB	ppm	MS Rank	MSMS ppm	MSMS Rank	Found
1	C15H9F3N2O	291.0740	11.0	-2.7	1			NA/NA

HR-MS spectrum of S2-8

Spectrum from MASS20210609.wiff2 (sample 56) - Y9, +TOF MS (50 - 1000) from 0.132 to 0.167 min

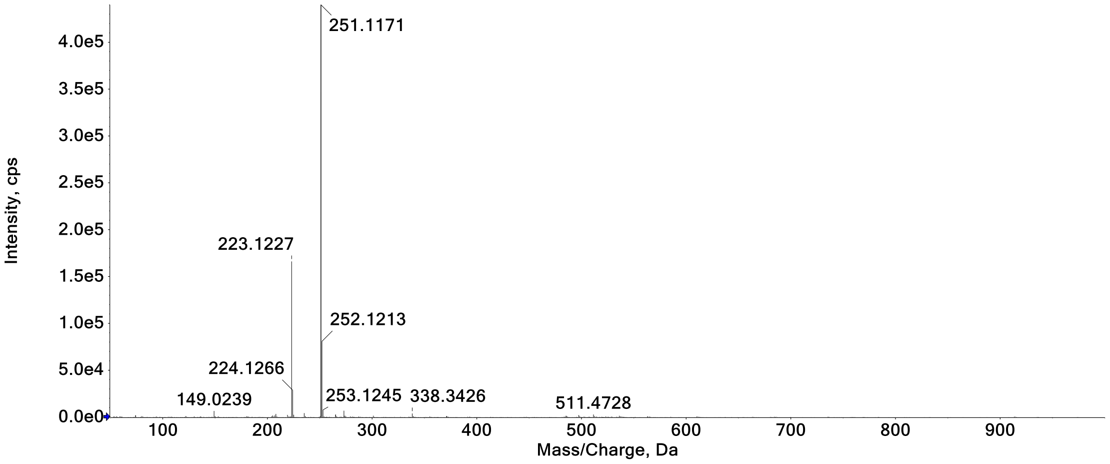

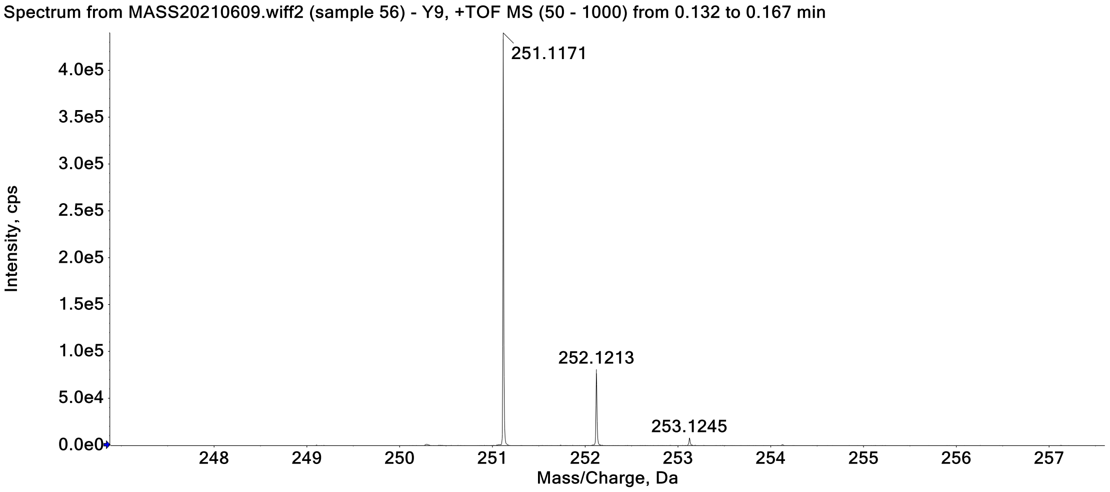

Hit	Formula	m/z	RDB	ppm	MS Rank	MSMS ppm	MSMS Rank	Found
1	C16H14N2O	251.1179	11.0	-3.1	1			NA/NA

HR-MS spectrum of S2-9

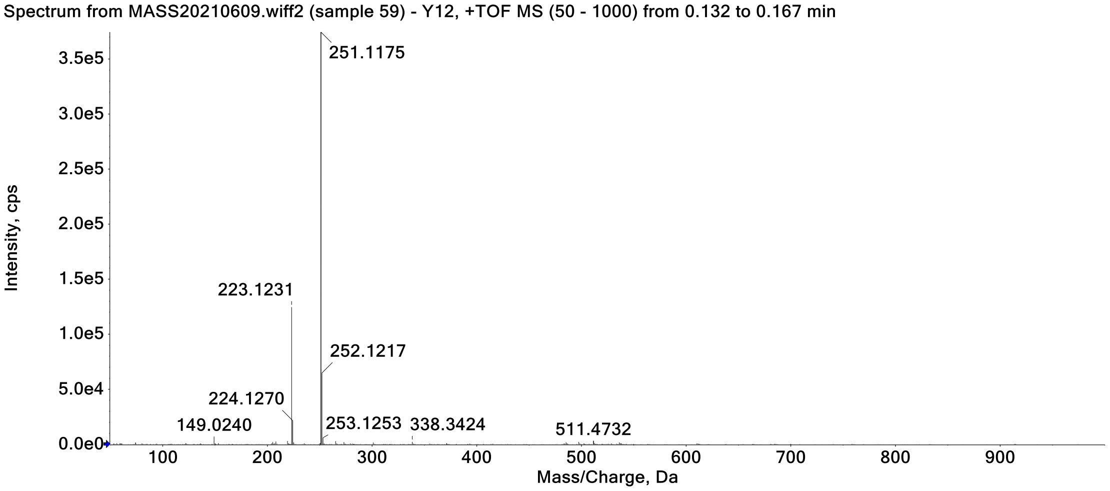

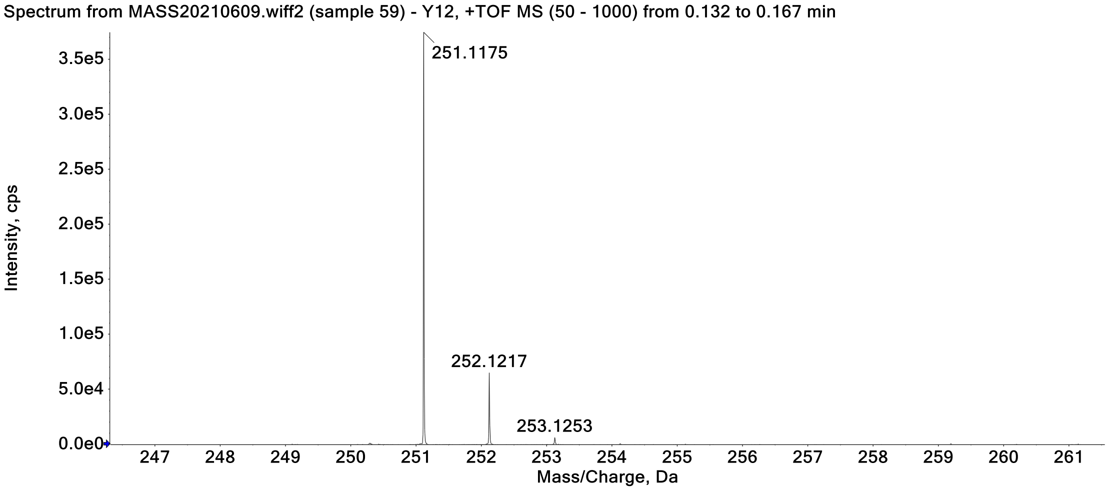

Hit	Formula	m/z	RDB	ppm	MS Rank	MSMS ppm	MSMS Rank	Found
1	C16H14N2O	251.1179	11.0	-1.6	1			NA/NA

HR-MS spectrum of S2-10

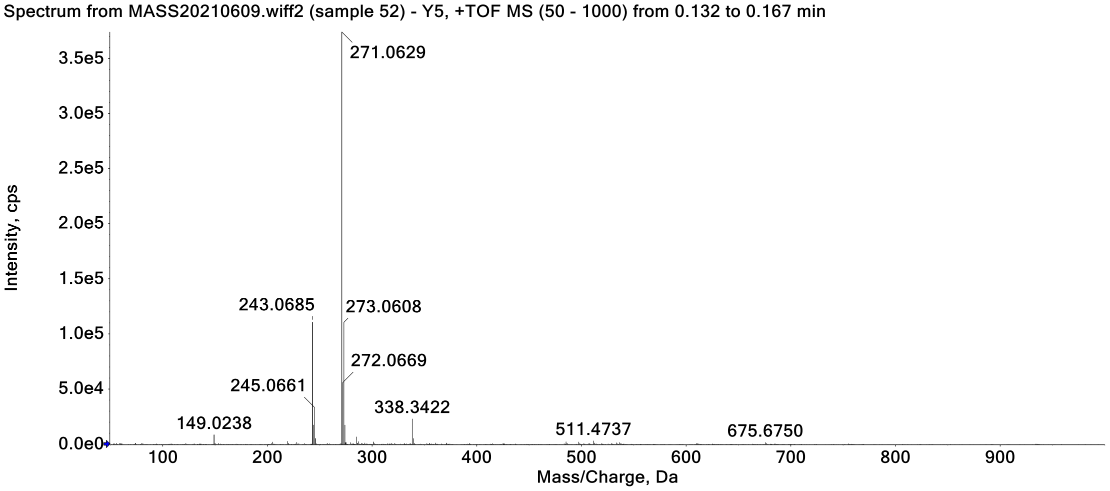

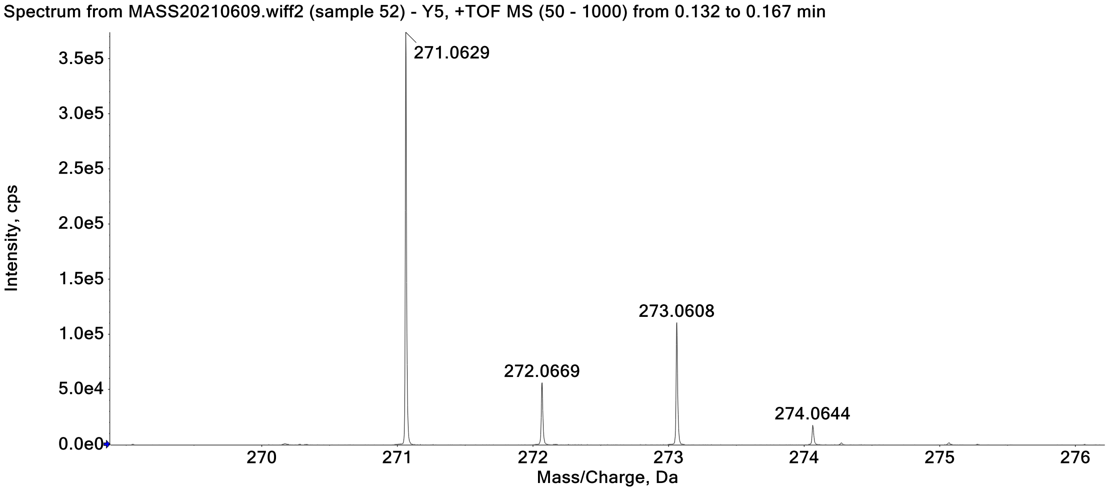

Hit	Formula	m/z	RDB	ppm	MS Rank	MSMS ppm	MSMS Rank	Found
1	C15H11ClN2O	271.0633	11.0	-1.4	1			NA/NA

HR-MS spectrum of S2-11

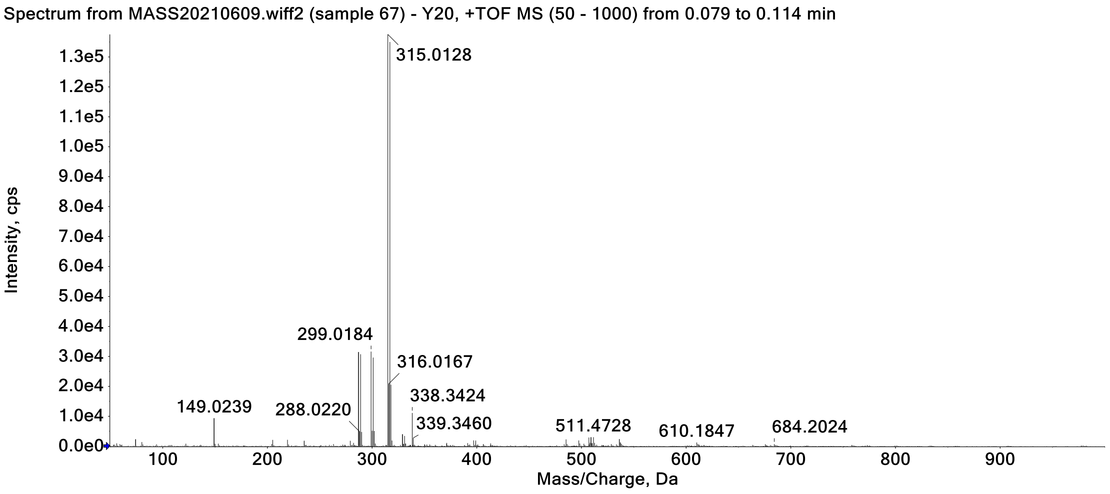

Spectrum from MASS20210609.wiff2 (sample 67) - Y20, +TOF MS (50 - 1000) from 0.079 to 0.114 min

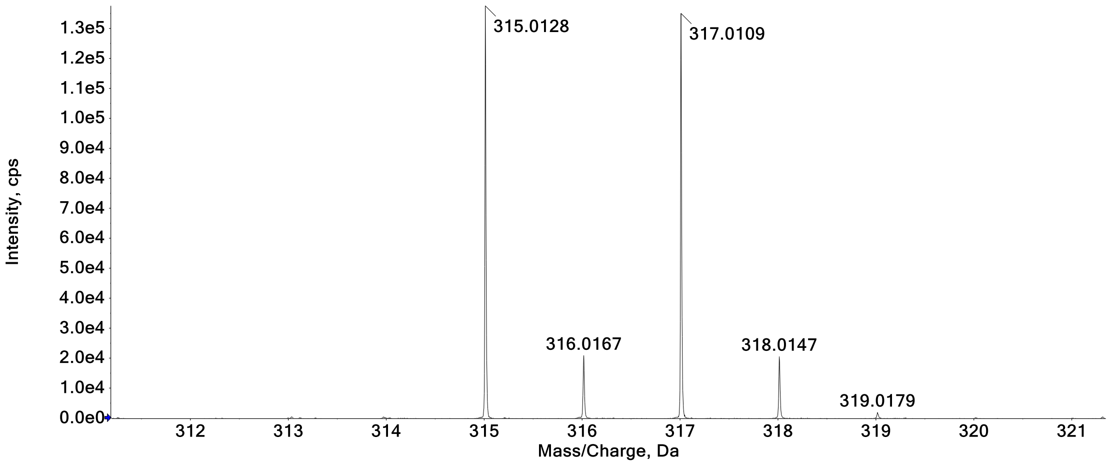

Hit	Formula	m/z	RDB	ppm	MS Rank	MSMS ppm	MSMS Rank	Found
1	C15H11BrN2O	315.0128	11.0	0.2	1			NA/NA

HR-MS spectrum of S2-12

Spectrum from MASS20210609.wiff2 (sample 71) - Y24, +TOF MS (50 - 1000) from 0.079 to 0.114 min

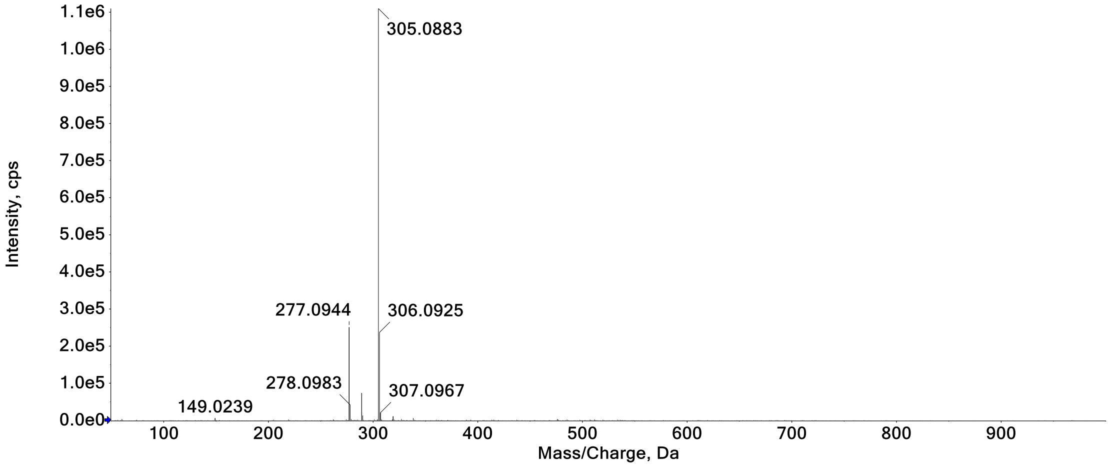

Spectrum from MASS20210609.wiff2 (sample 71) - Y24, +TOF MS (50 - 1000) from 0.079 to 0.114 min

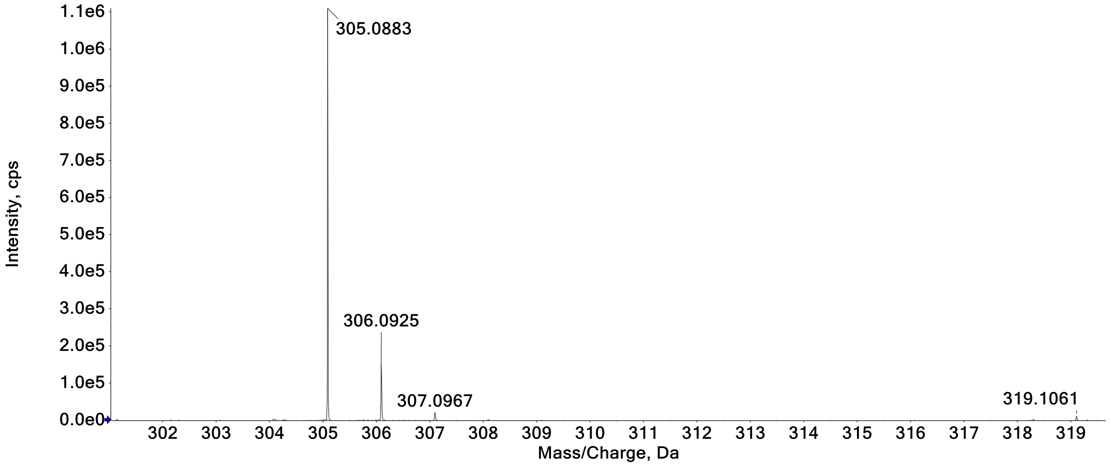

Hit	Formula	m/z	RDB	ppm	MS Rank	MSMS ppm	MSMS Rank	Found
1	C16H11F3N2O	305.0896	11.0	-4.3	1			NA/NA

HR-MS spectrum of S2-13

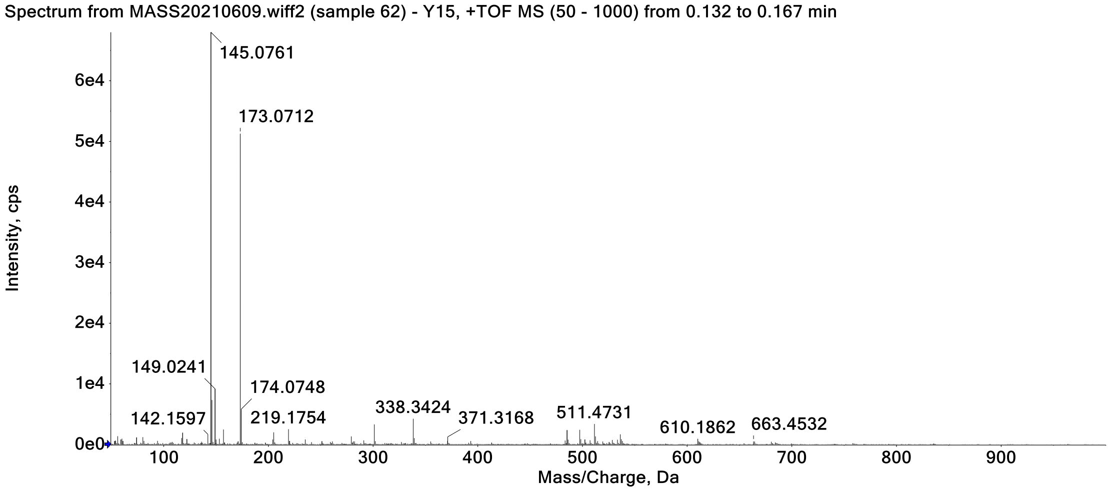

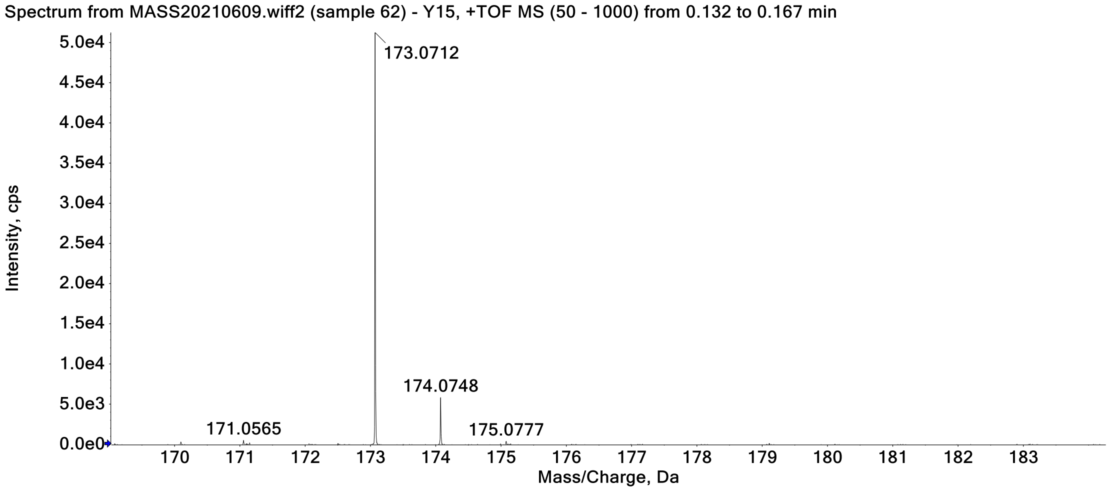

Hit	Formula	m/z	RDB	ppm	MS Rank	MSMS ppm	MSMS Rank	Found
1	C10H8N2O	173.0709	8.0	1.5	1			NA/NA

HR-MS spectrum of S3-1

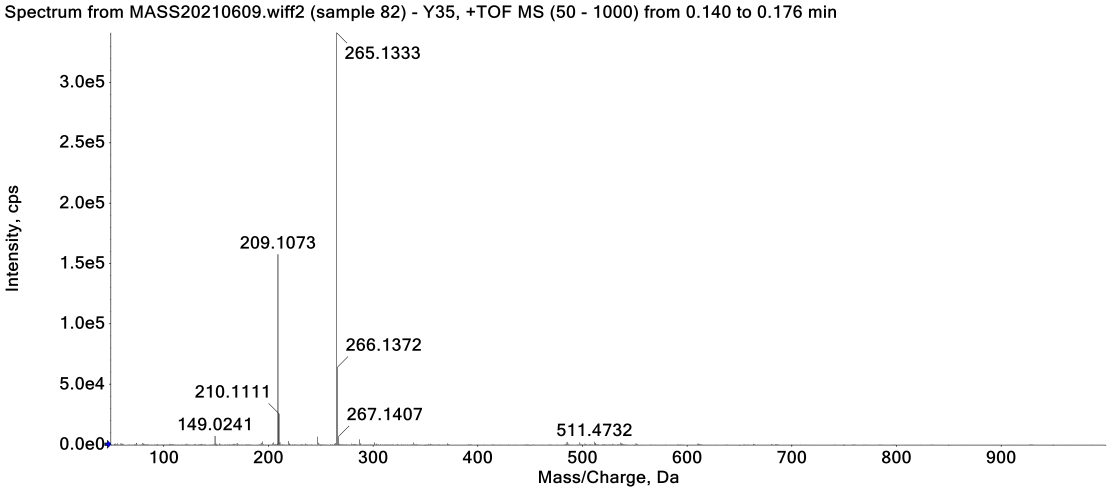

Spectrum from MASS20210609.wiff2 (sample 82) - Y35, +TOF MS (50 - 1000) from 0.140 to 0.176 min

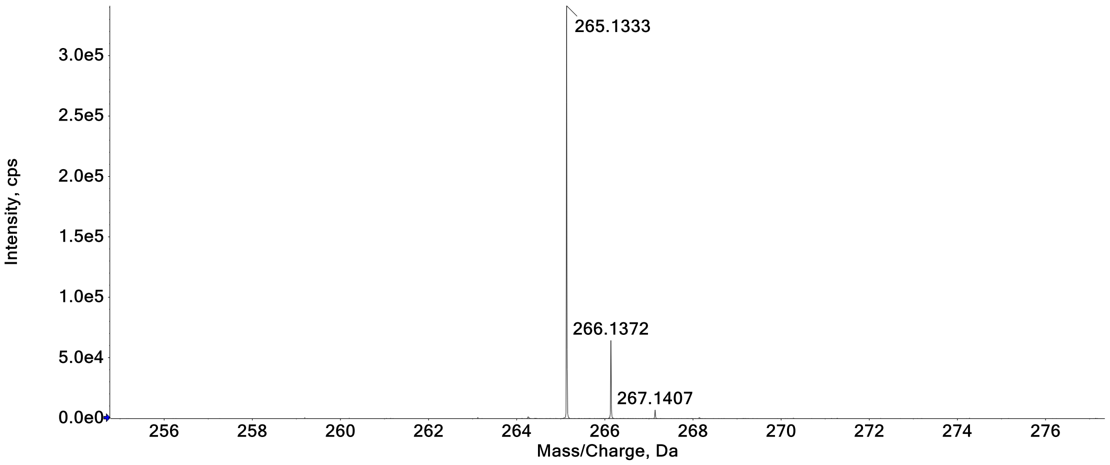

Hit	Formula	m/z	RDB	ppm	MS Rank	MSMS ppm	MSMS Rank	Found
1	C17H16N2O	265.1335	11.0	-0.9	1			NA/NA

HR-MS spectrum of S3-2

Spectrum from MASS20210609.wiff2 (sample 85) - Y38, +TOF MS (50 - 1000) from 0.140 to 0.176 min

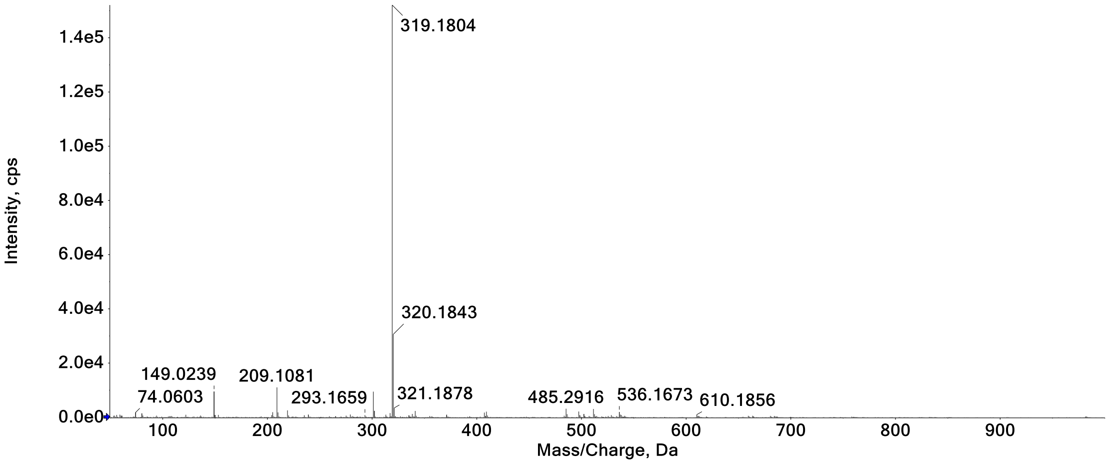

Spectrum from MASS20210609.wiff2 (sample 85) - Y38, +TOF MS (50 - 1000) from 0.140 to 0.176 min

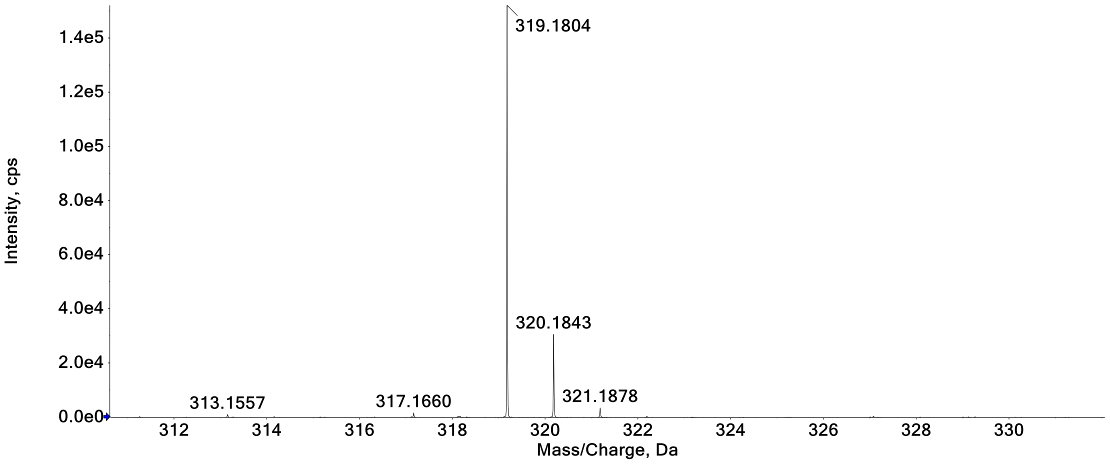

Hit	Formula	m/z	RDB	ppm	MS Rank	MSMS ppm	MSMS Rank	Found
1	C21H22N2O	319.1805	12.0	-0.3	1			NA/NA

HR-MS spectrum of S3-3

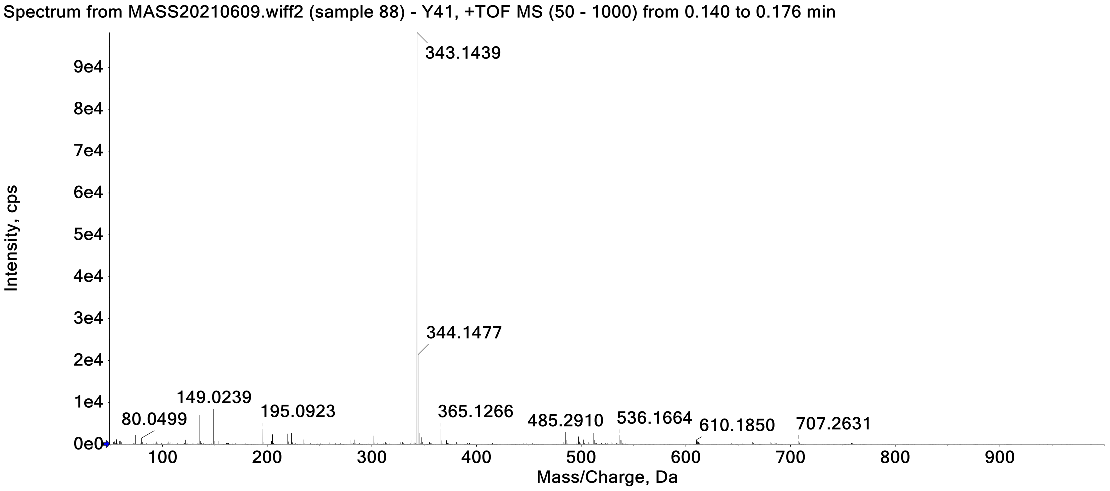

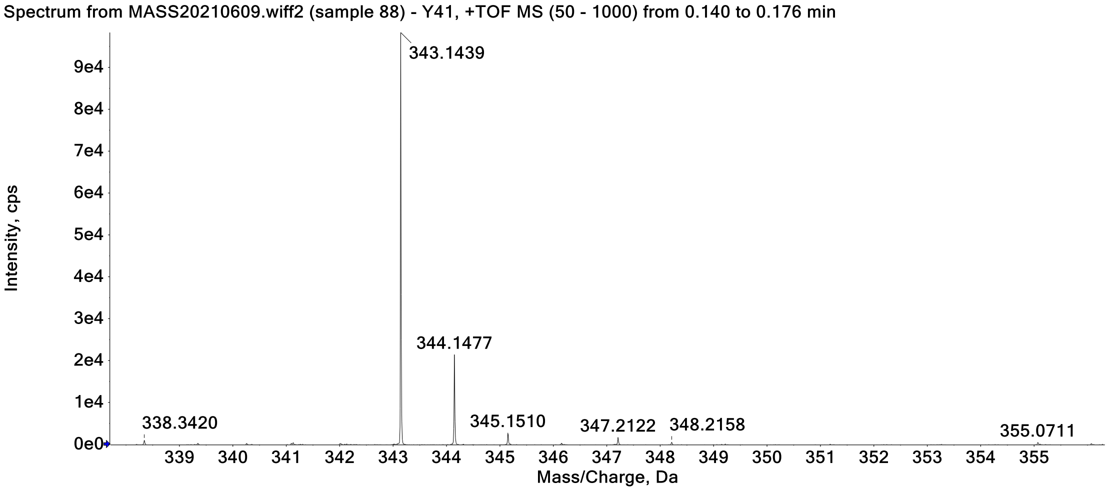

Hit	Formula	m/z	RDB	ppm	MS Rank	MSMS ppm	MSMS Rank	Found
1	C22H18N2O2	343.1441	15.0	-0.6	1			NA/NA

HR-MS spectrum of S3-4

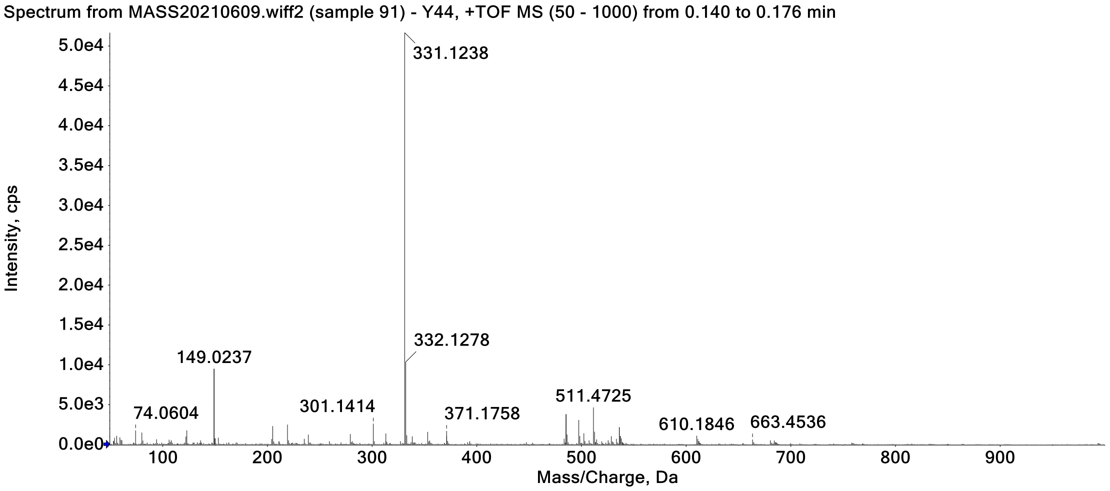

Spectrum from MASS20210609.wiff2 (sample 91) - Y44, +TOF MS (50 - 1000) from 0.140 to 0.176 min

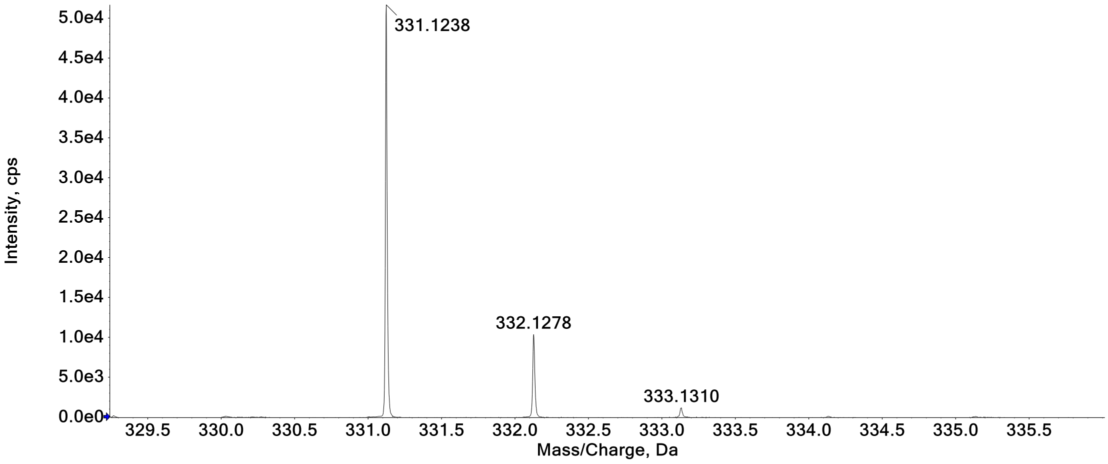

Hit	Formula	m/z	RDB	ppm	MS Rank	MSMS ppm	MSMS Rank	Found
1	C21H15FN2O	331.1241	15.0	-1.0	1			NA/NA

HR-MS spectrum of S3-5

Spectrum from MASS20220928.wiff2 (sample 2) - Y1, Experiment 1, +IDA T...) - Y1, Experiment 1, +IDA TOF MS (50 - 1000) from 0.138 to 0.230 min]

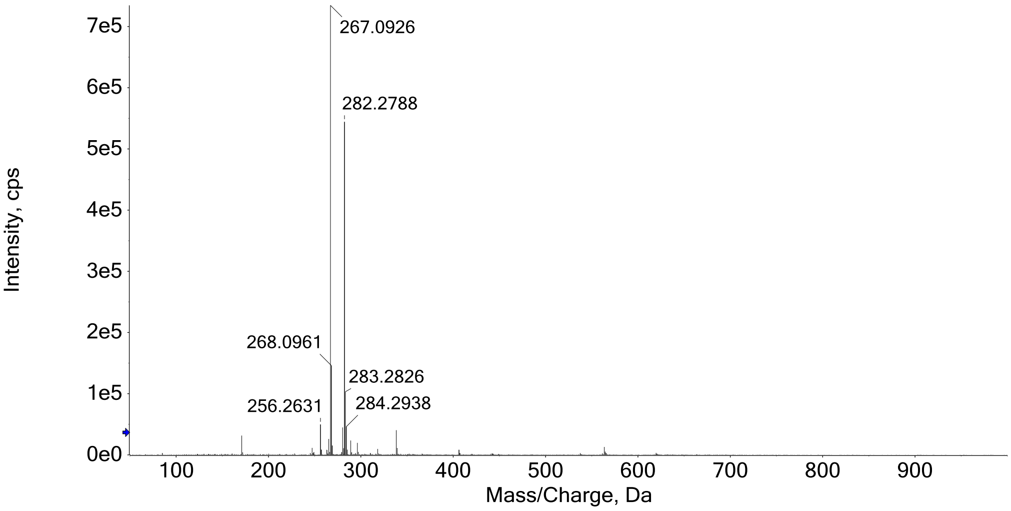

Spectrum from MASS20220928.wiff2 (sample 2) - Y1, Experiment 1, +IDA T...) - Y1, Experiment 1, +IDA TOF MS (50 - 1000) from 0.138 to 0.230 min]

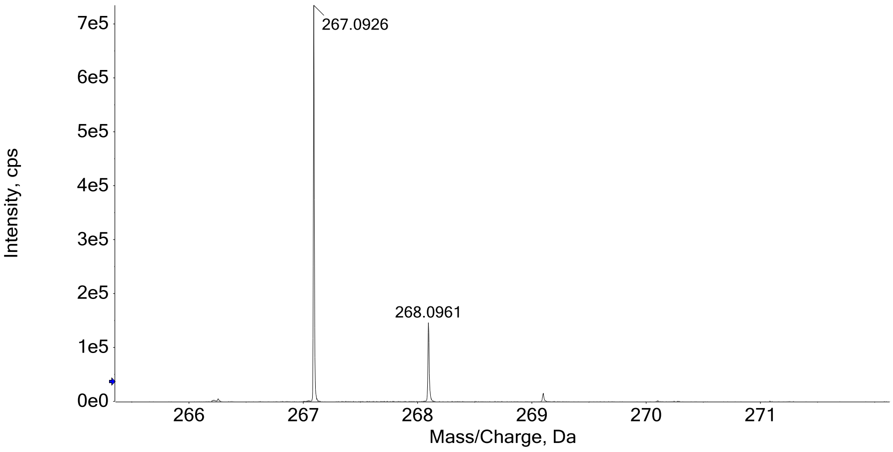

Hit	Formula	m/z	RDB	ppm	MS Rank	MSMS ppm	MSMS Rank	Found
1	C16H11FN2O	267.0928	12.0	-0.8	1			NA/NA

HR-MS spectrum of S4-2

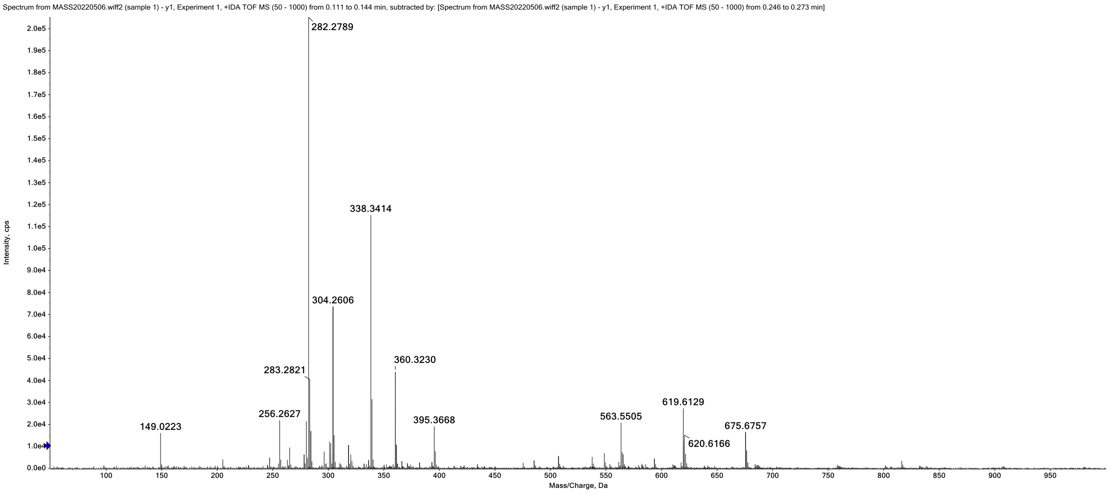

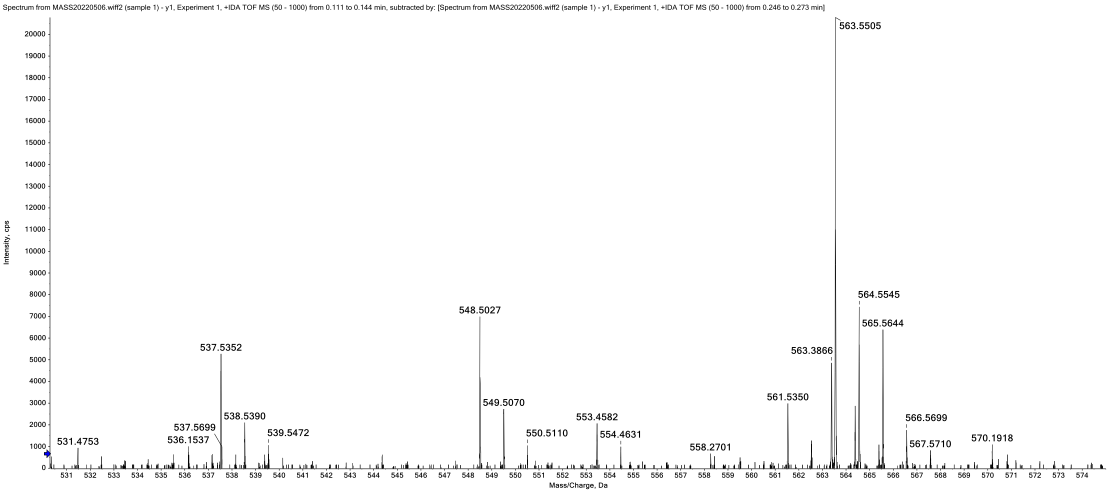

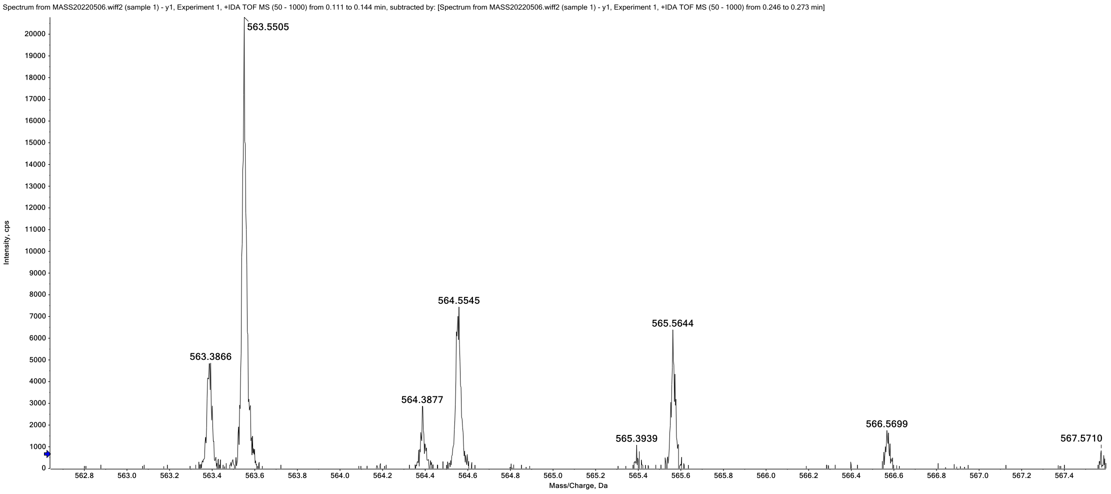

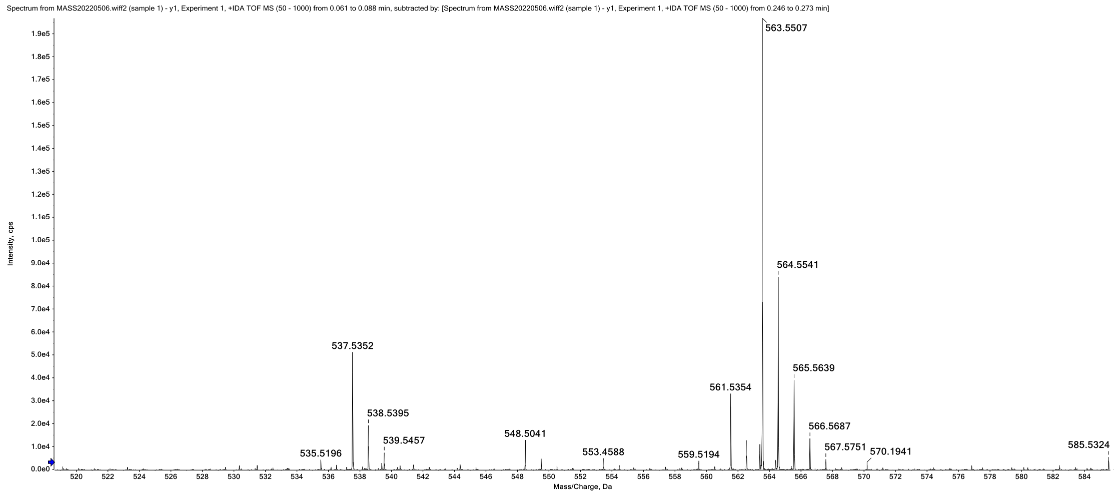

Hit	Formula	m/z	RDB	ppm	MS Rank	MSMS ppm	MSMS Rank	Found
1	C38H52O2	563.3860	13.0	2.2	1			NA/NA

HR-MS spectrum of 1a

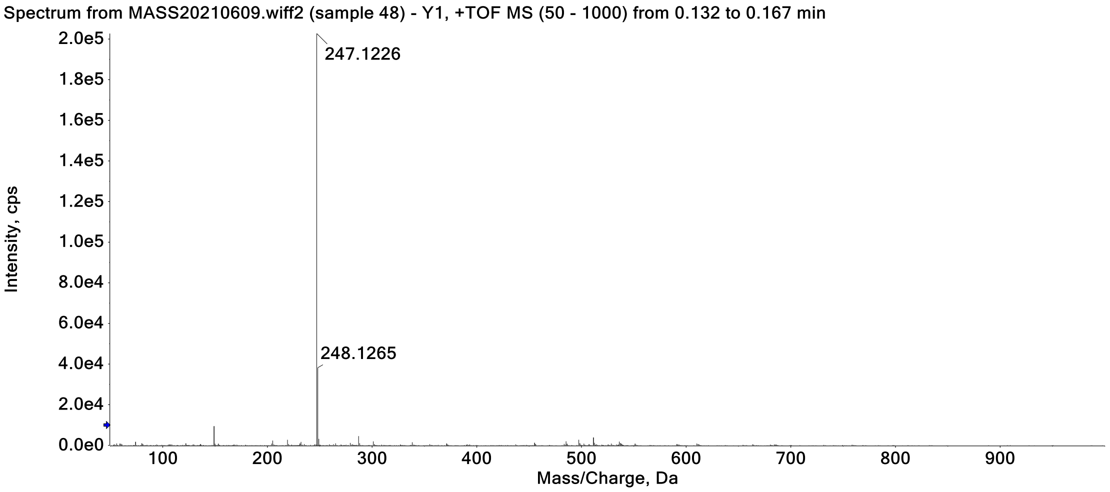

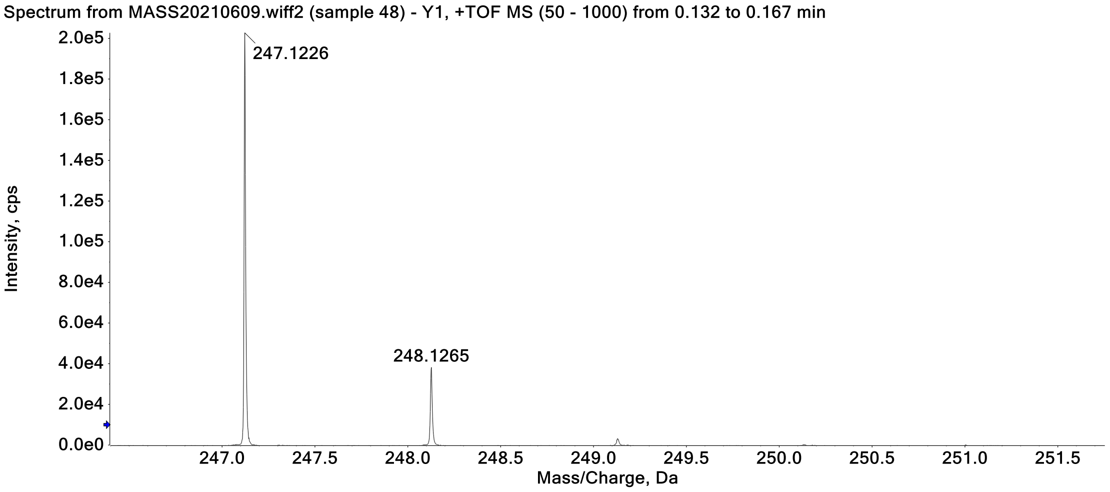

Hit	Formula	m/z	RDB	ppm	MS Rank	MSMS ppm	MSMS Rank	Found
1	C17H14N2	247.1230	12.0	-1.5	1			NA/NA

HR-MS spectrum of 1b

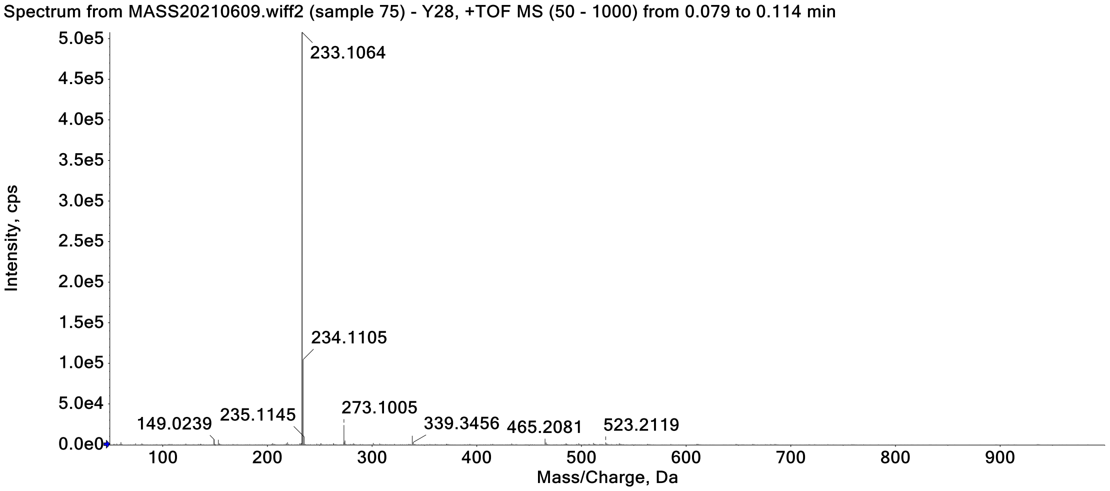

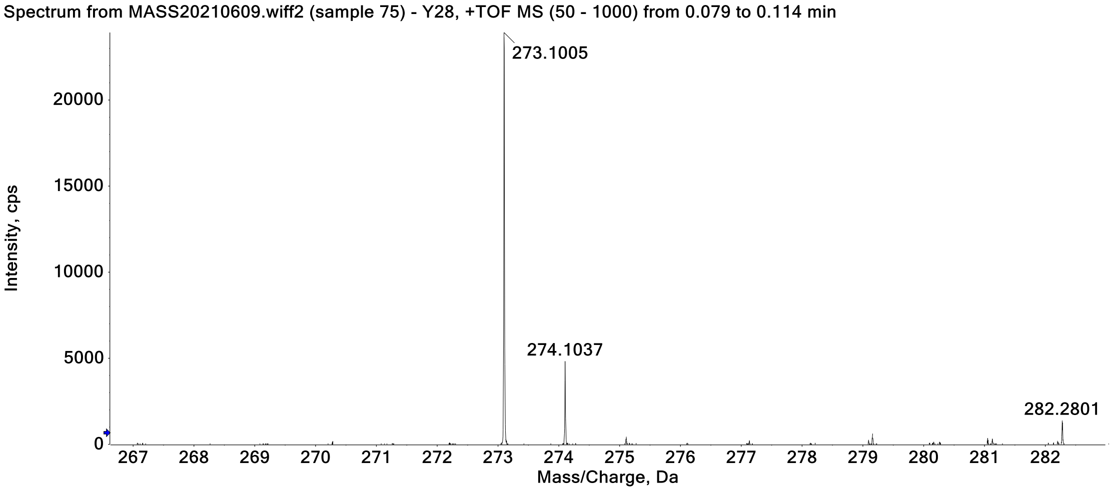

Hit	Formula	m/z	RDB	ppm	MS Rank	MSMS ppm	MSMS Rank	Found
1	C16H14N2O	273.0998	11.0	2.4	1			NA/NA

HR-MS spectrum of 1c

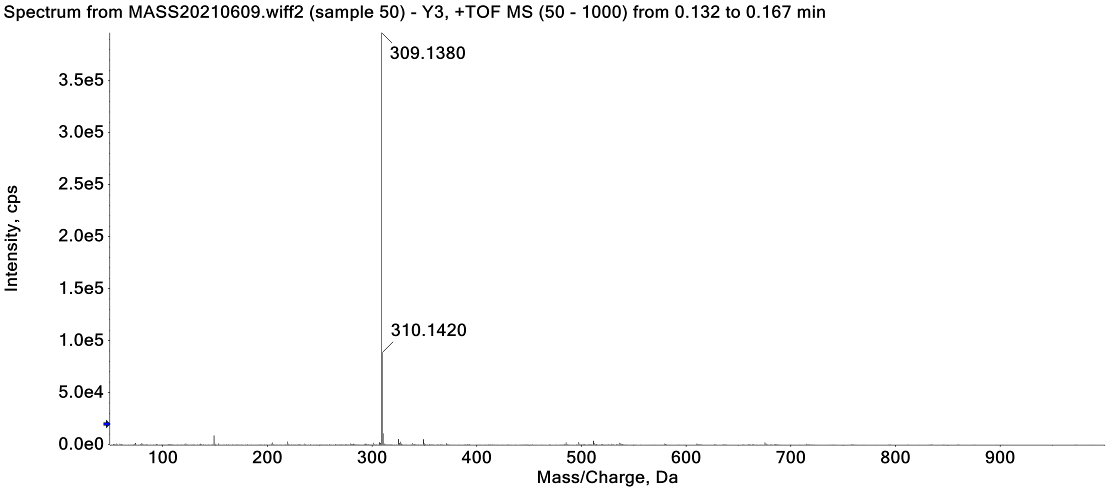

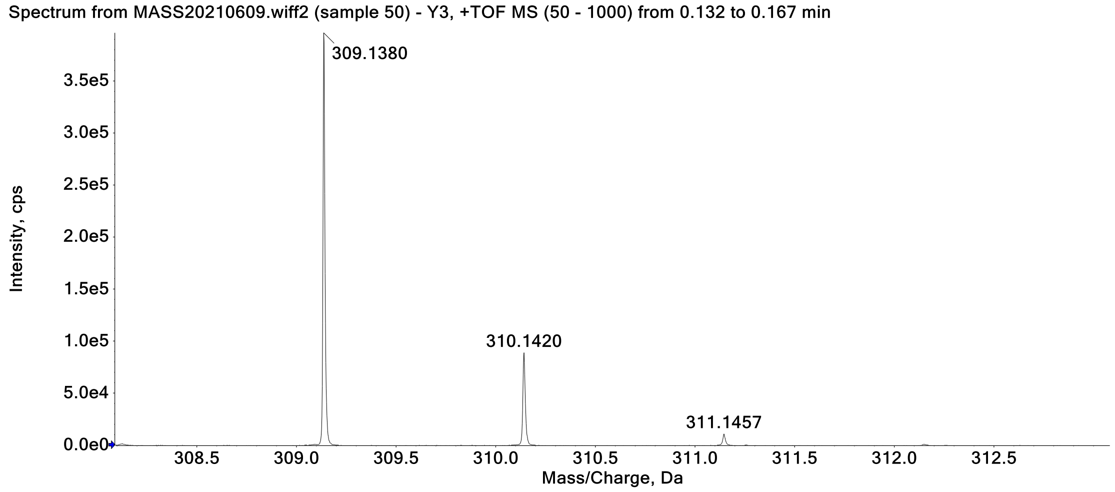

Hit	Formula	m/z	RDB	ppm	MS Rank	MSMS ppm	MSMS Rank	Found
1	C22H16N2	309.1386	16.0	-2.0	1			NA/NA

HR-MS spectrum of 1d

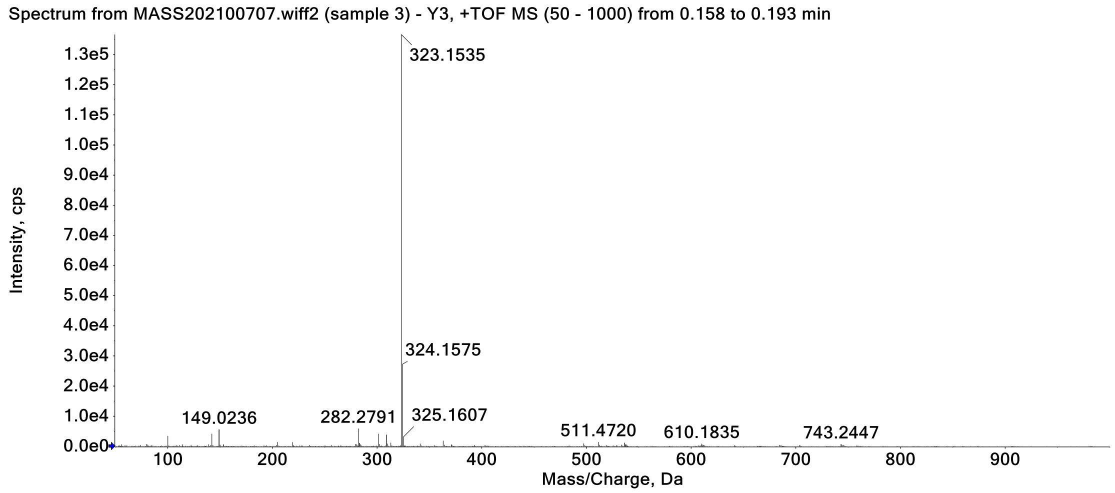

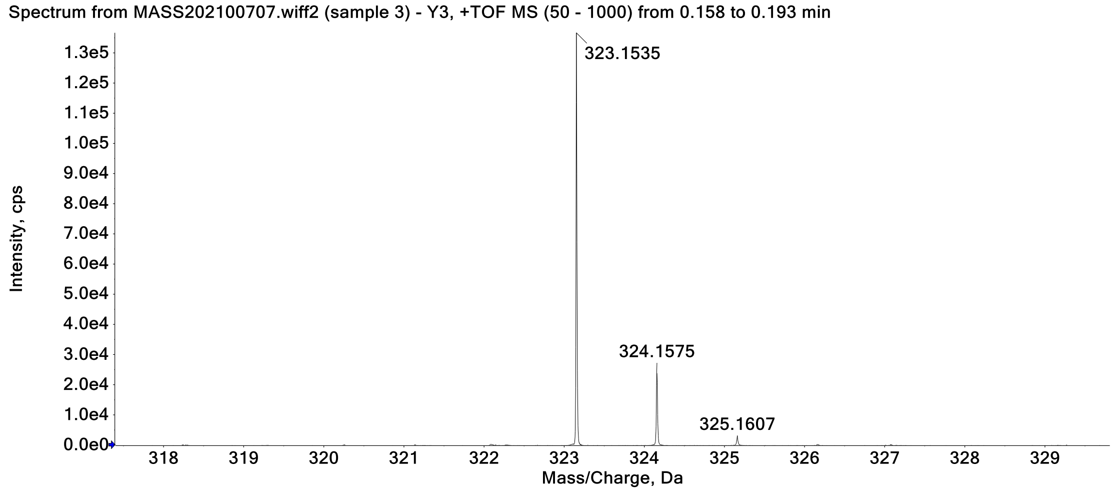

Hit	Formula	m/z	RDB	ppm	MS Rank	MSMS ppm	MSMS Rank	Found
1	C23H18N2	323.1543	16.0	-2.4	1			NA/NA

HR-MS spectrum of 1e

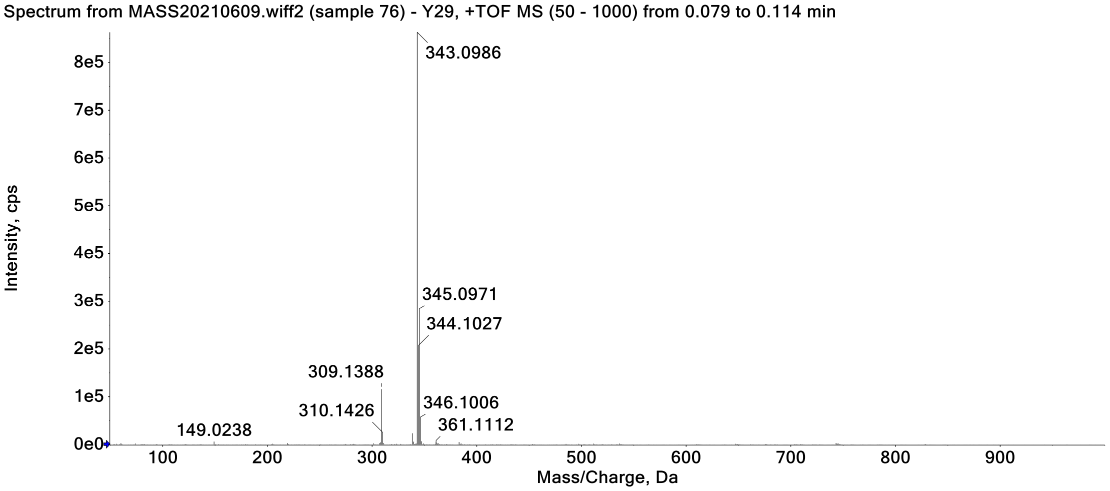

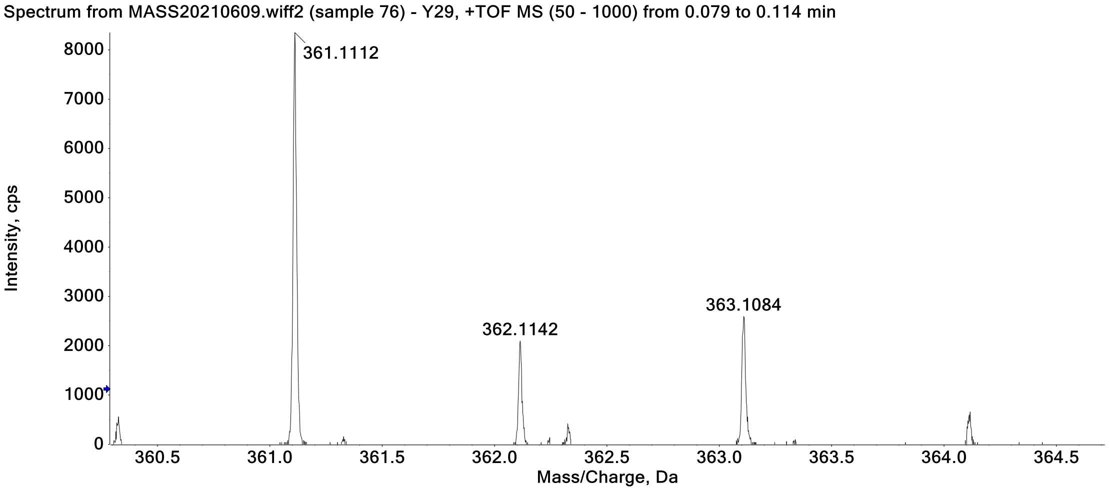

Hit	Formula	m/z	RDB	ppm	MS Rank	MSMS ppm	MSMS Rank	Found
1	C22H17ClN2O	361.1102	15.0	2.7	1			NA/NA

HR-MS spectrum of 1f

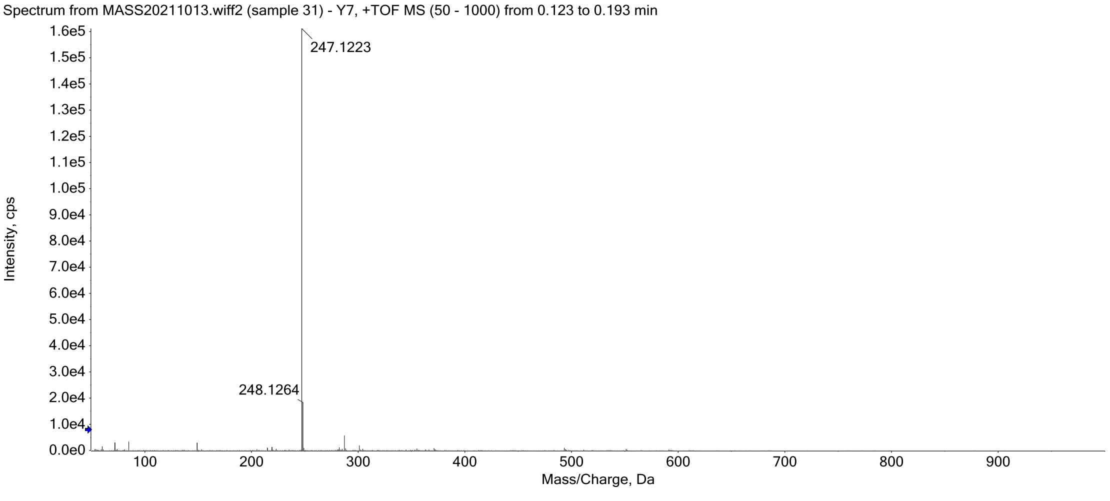

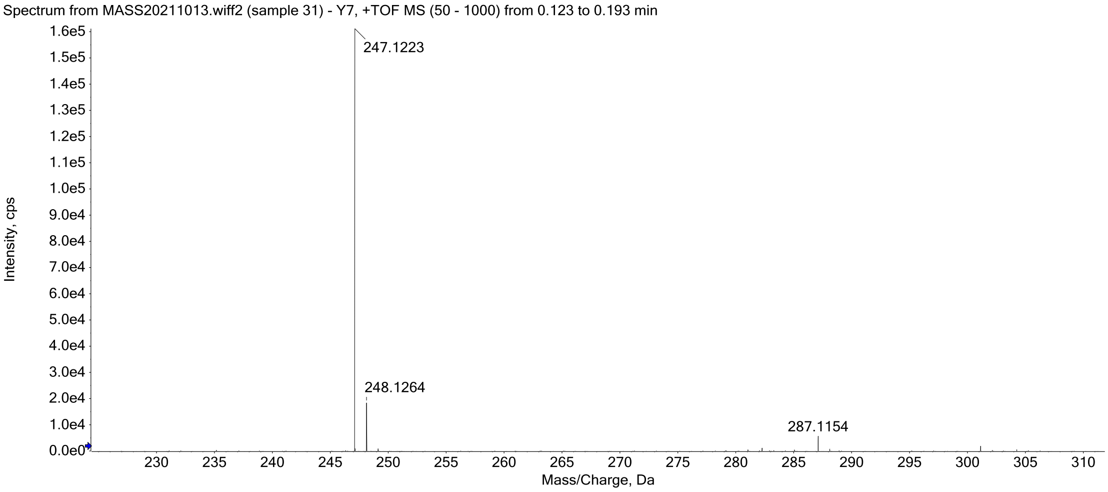

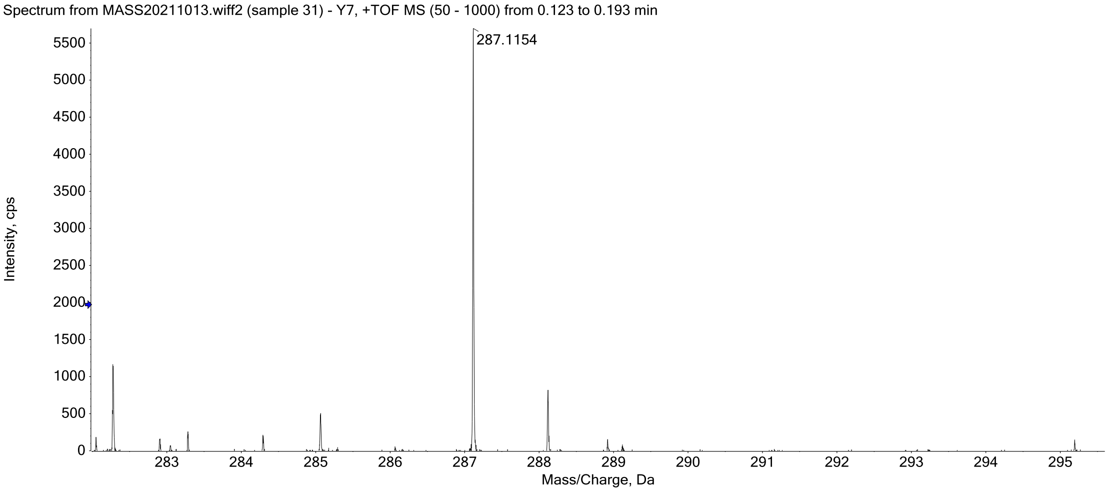

Hit	Formula	m/z	RDB	ppm	MS Rank	MSMS ppm	MSMS Rank	Found
1	C17H16N2O	287.1155	11.0	-0.3	1			NA/NA

HR-MS spectrum of 1g

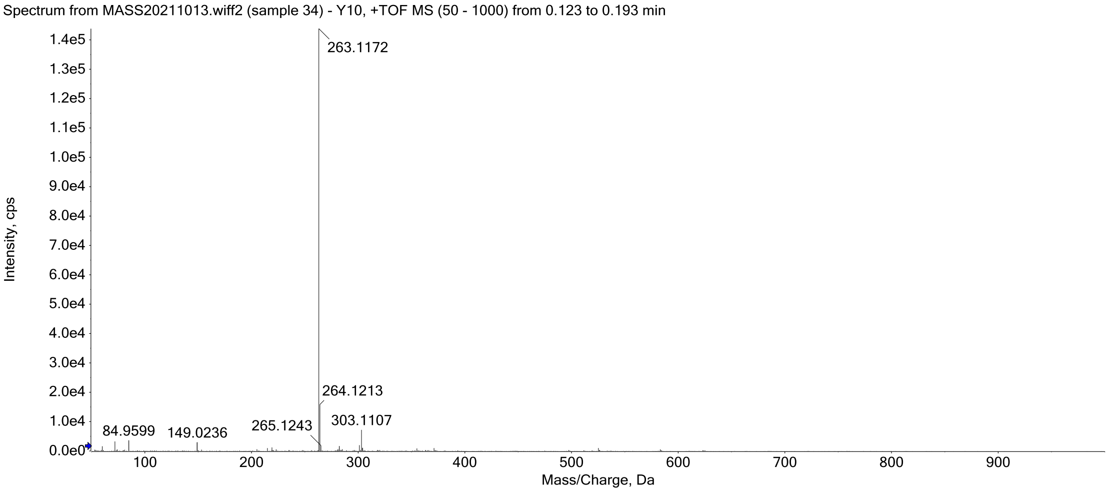

Spectrum from MASS20211013.wiff2 (sample 34) - Y10, +TOF MS (50 - 1000) from 0.123 to 0.193 min

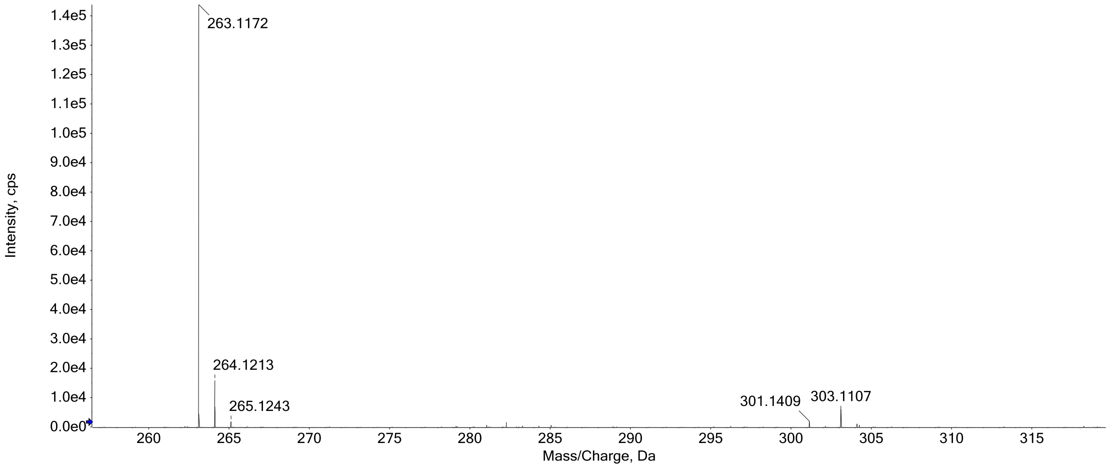

Spectrum from MASS20211013.wiff2 (sample 34) - Y10, +TOF MS (50 - 1000) from 0.123 to 0.193 min

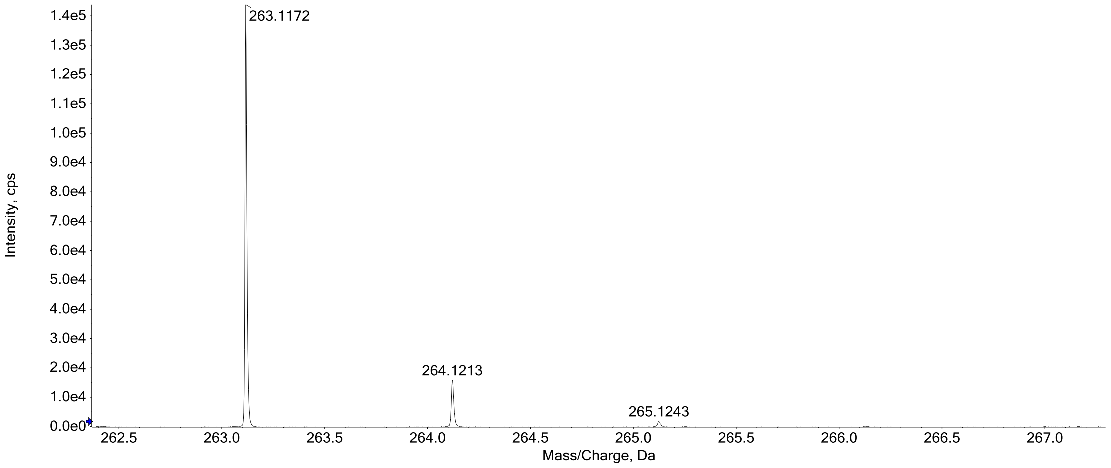

Hit	Formula	m/z	RDB	ppm	MS Rank	MSMS ppm	MSMS Rank	Found
1	C17H14N2O	263.1179	12.0	-2.6	1			NA/NA

HR-MS spectrum of 1h

Spectrum from MASS20211013.wiff2 (sample 28) - Y4, +TOF MS (50 - 1000) from 0.088 to 0.158 min

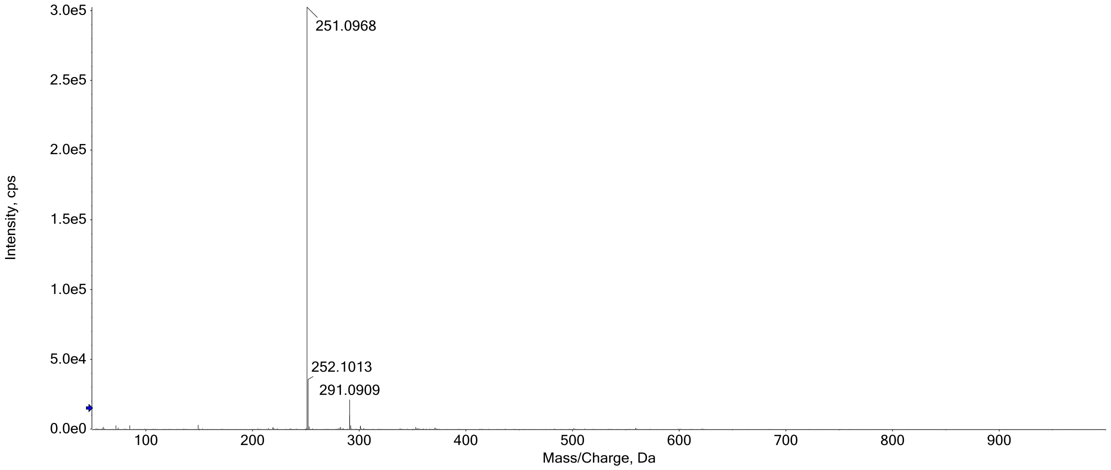

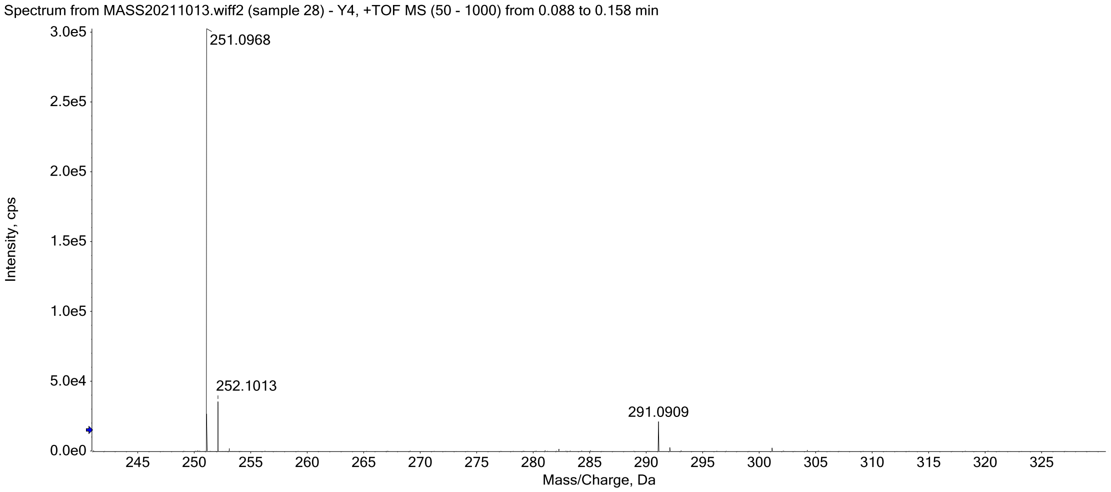

Hit	Formula	m/z	RDB	ppm	MS Rank	MSMS ppm	MSMS Rank	Found
1	C16H13FN2O	291.0904	11.0	1.7	1			NA/NA

HR-MS spectrum of 1i

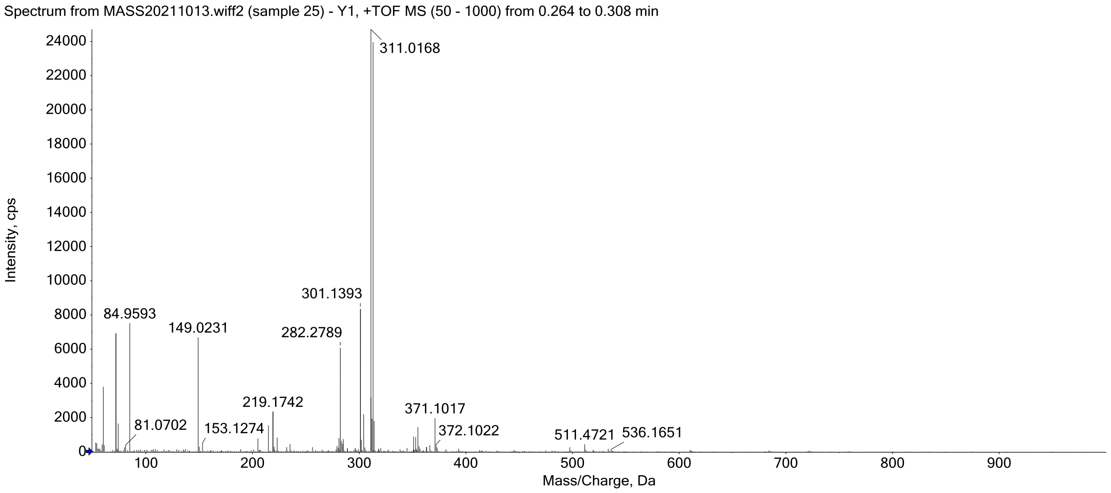

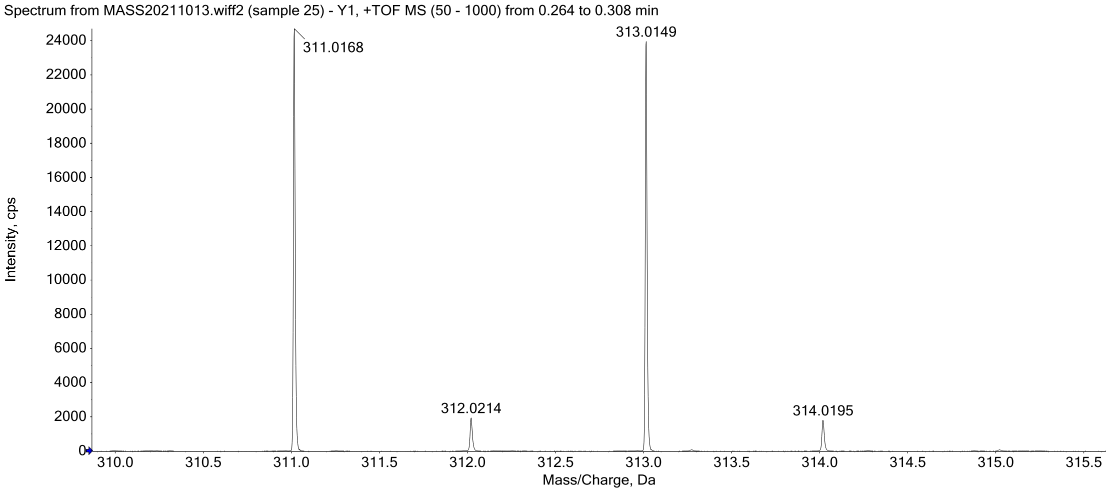

Hit	Formula	m/z	RDB	ppm	MS Rank	MSMS ppm	MSMS Rank	Found
1	C16H11BrN2	311.0178	12.0	-3.3	1			NA/NA

HR-MS spectrum of 1j

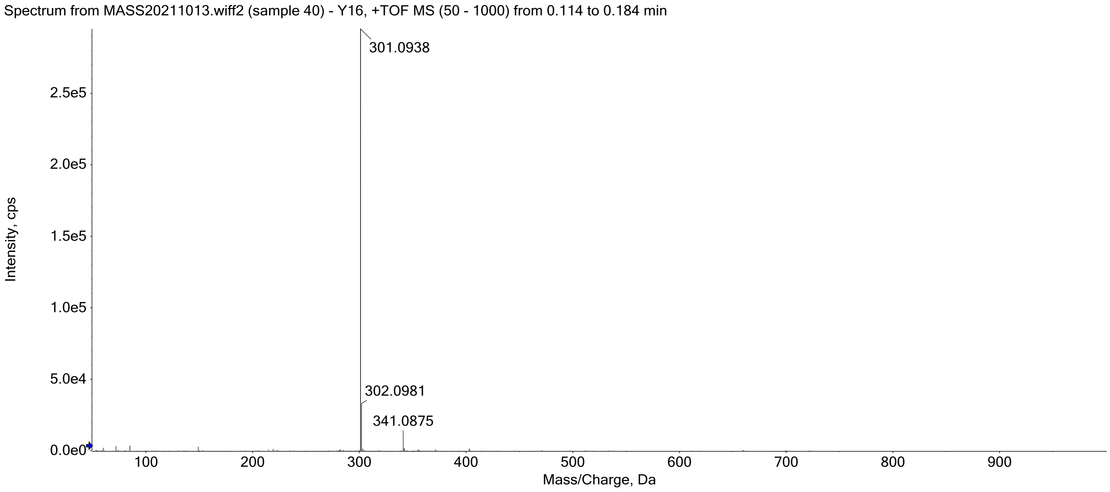

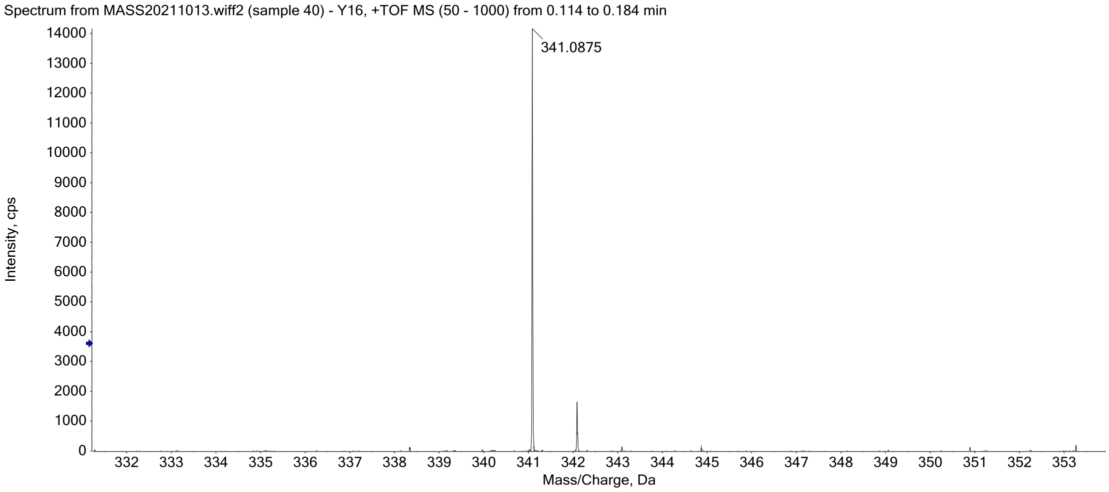

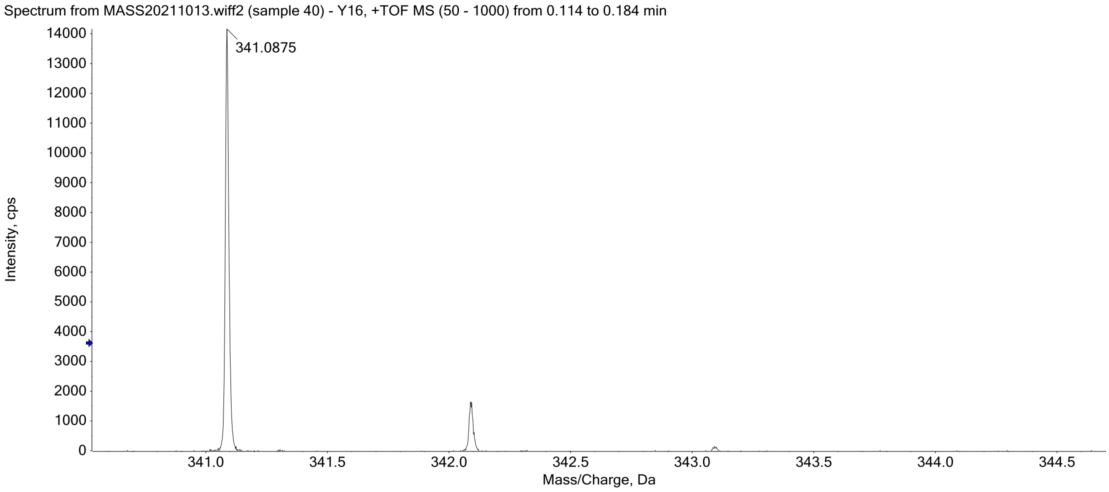

Hit	Formula	m/z	RDB	ppm	MS Rank	MSMS ppm	MSMS Rank	Found
1	C17H13F3N2O	341.0872	11.0	0.8	1			NA/NA

HR-MS spectrum of 1k

Spectrum from MASS20210609.wiff2 (sample 57) - Y10, +TOF MS (50 - 1000) from 0.132 to 0.167 min

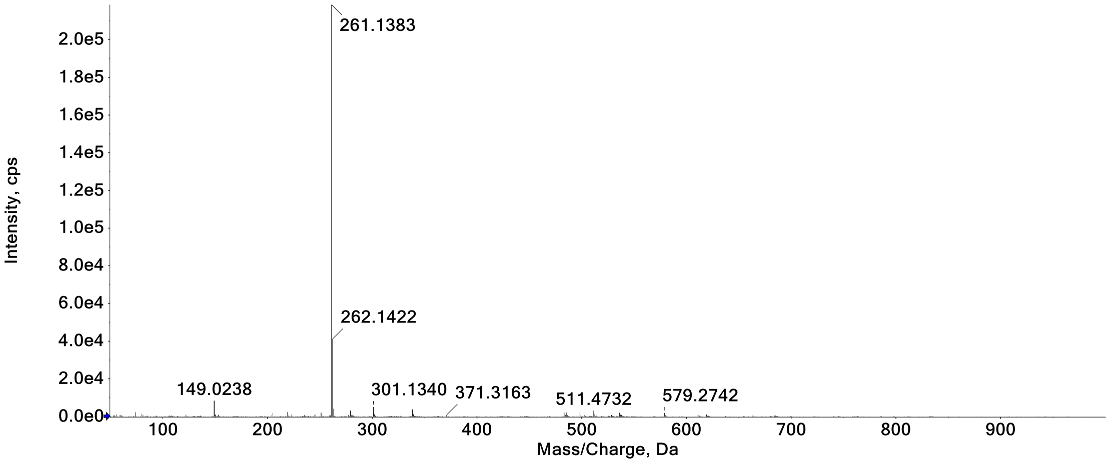

Spectrum from MASS20210609.wiff2 (sample 57) - Y10, +TOF MS (50 - 1000) from 0.132 to 0.167 min

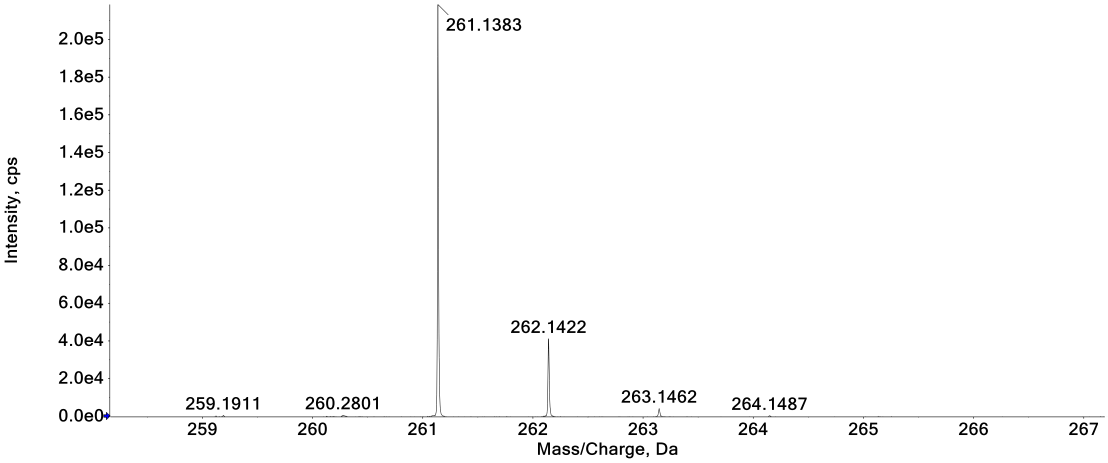

Hit	Formula	m/z	RDB	ppm	MS Rank	MSMS ppm	MSMS Rank	Found
1	C18H16N2	261.1386	12.0	-1.2	1			NA/NA

HR-MS spectrum of 1l

Spectrum from MASS20210609.wiff2 (sample 60) - Y13, +TOF MS (50 - 1000) from 0.132 to 0.167 min

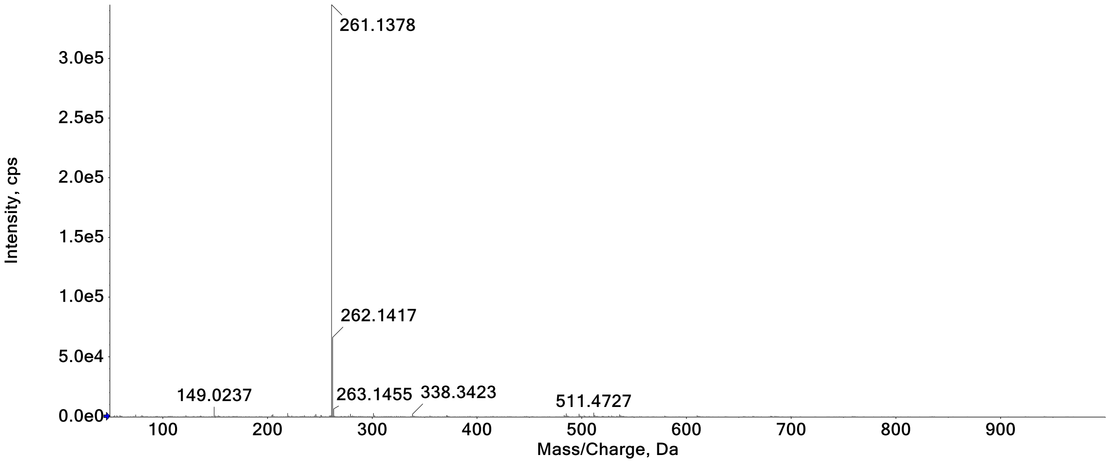

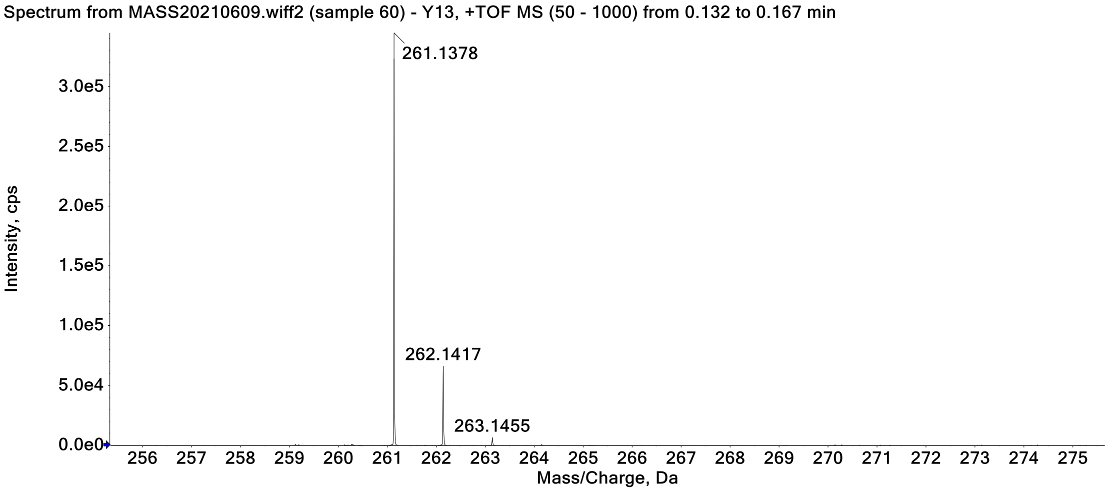

Hit	Formula	m/z	RDB	ppm	MS Rank	MSMS ppm	MSMS Rank	Found
1	C18H16N2	261.1386	12.0	-3.2	1			NA/NA

HR-MS spectrum of 1m

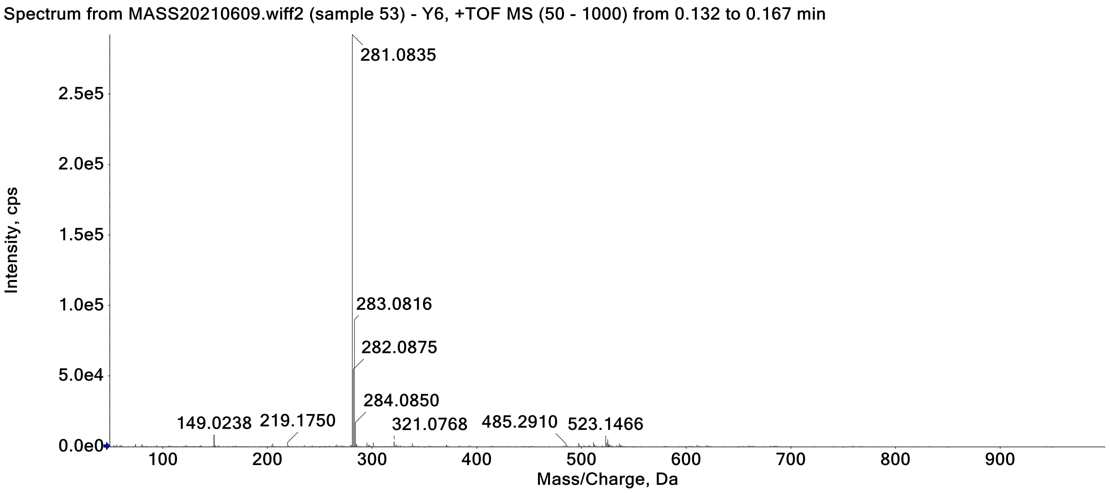

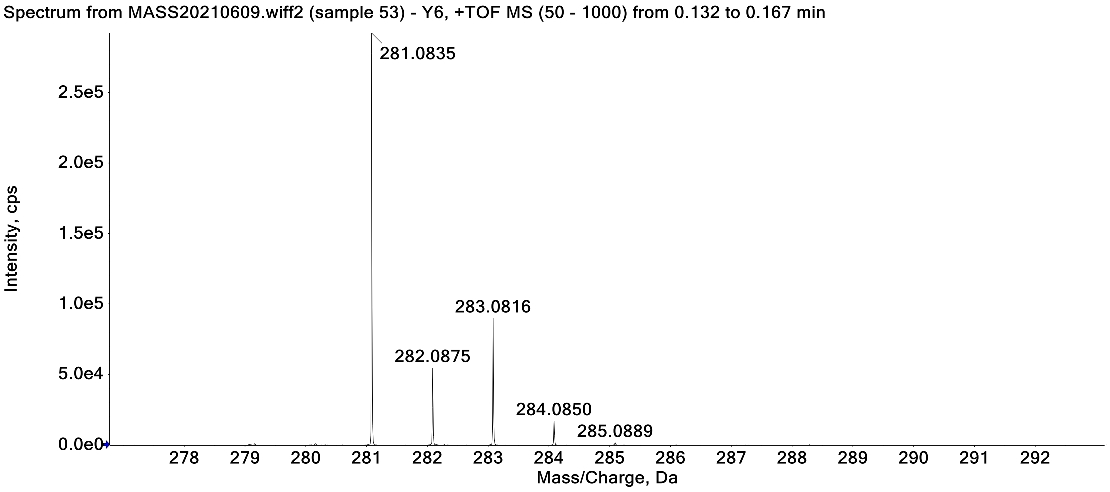

Hit	Formula	m/z	RDB	ppm	MS Rank	MSMS ppm	MSMS Rank	Found
1	C17H13ClN2	281.0840	12.0	-1.8	1			NA/NA

HR-MS spectrum of 1n

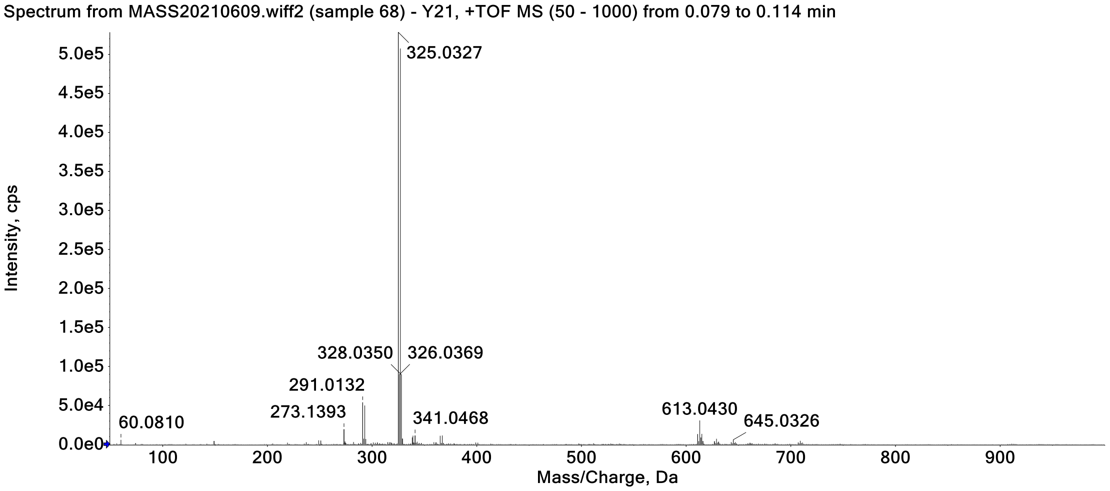

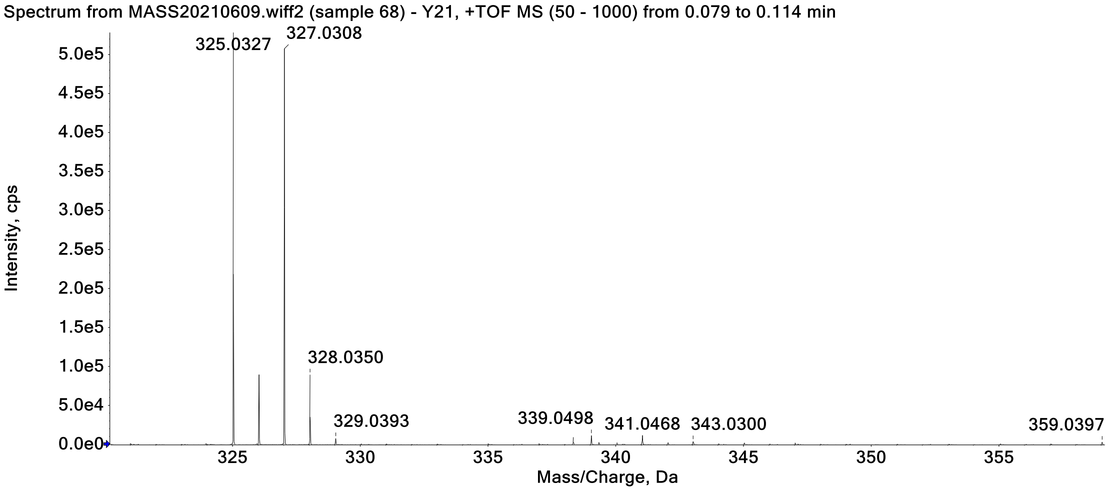

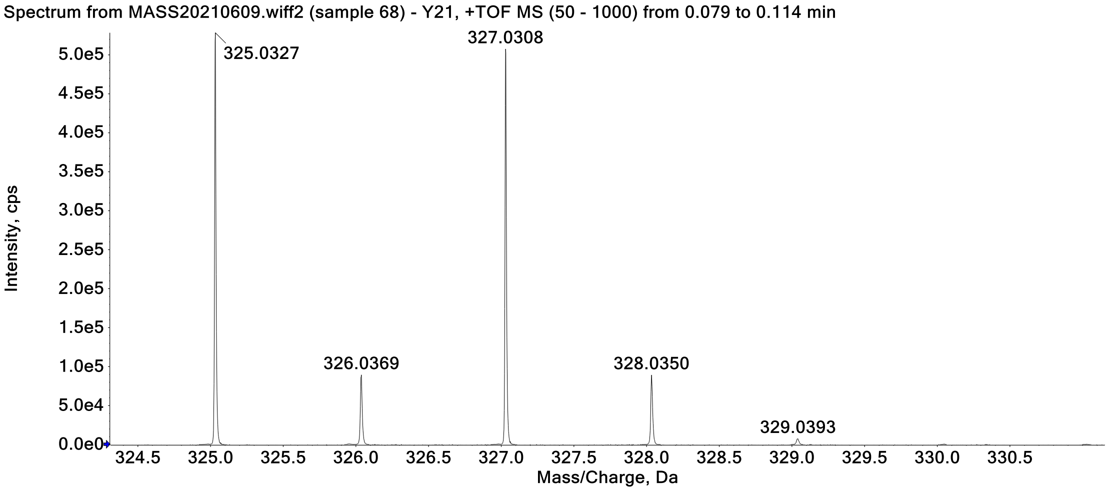

Hit	Formula	m/z	RDB	ppm	MS Rank	MSMS ppm	MSMS Rank	Found
1	C17H13BrN2	325.0335	12.0	-2.4	1			NA/NA

HR-MS spectrum of 1o

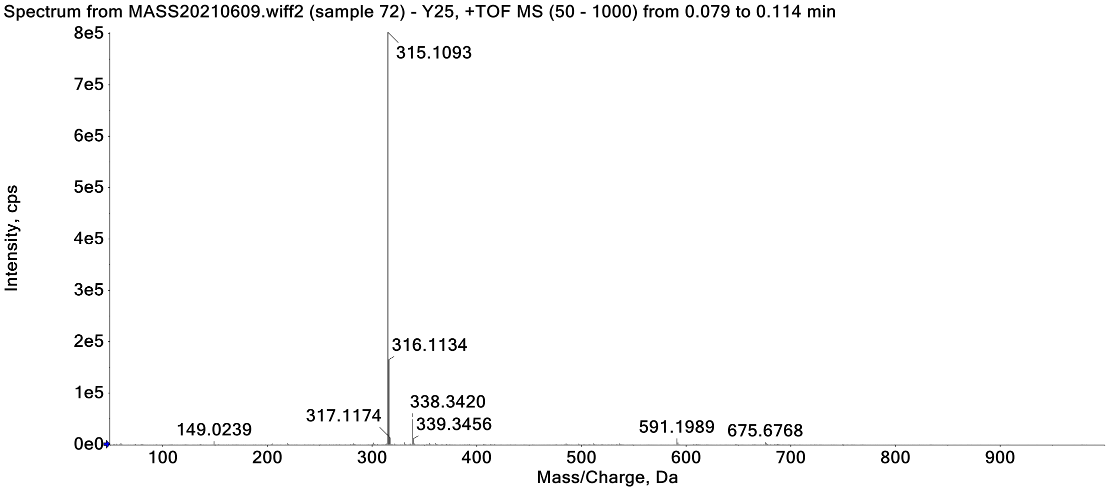

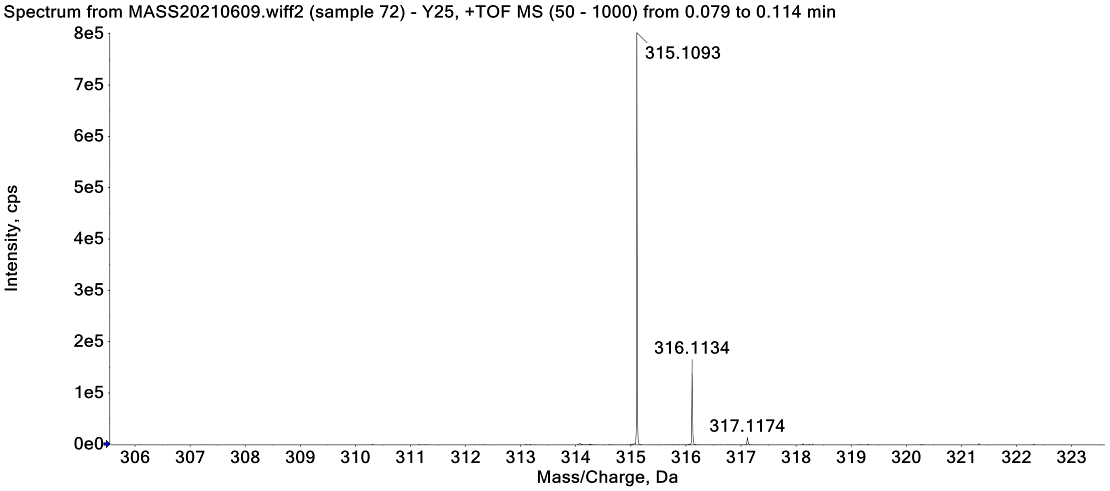

Hit	Formula	m/z	RDB	ppm	MS Rank	MSMS ppm	MSMS Rank	Found
1	C18H13F3N2	315.1104	12.0	-3.4	1			NA/NA

HR-MS spectrum of 1p

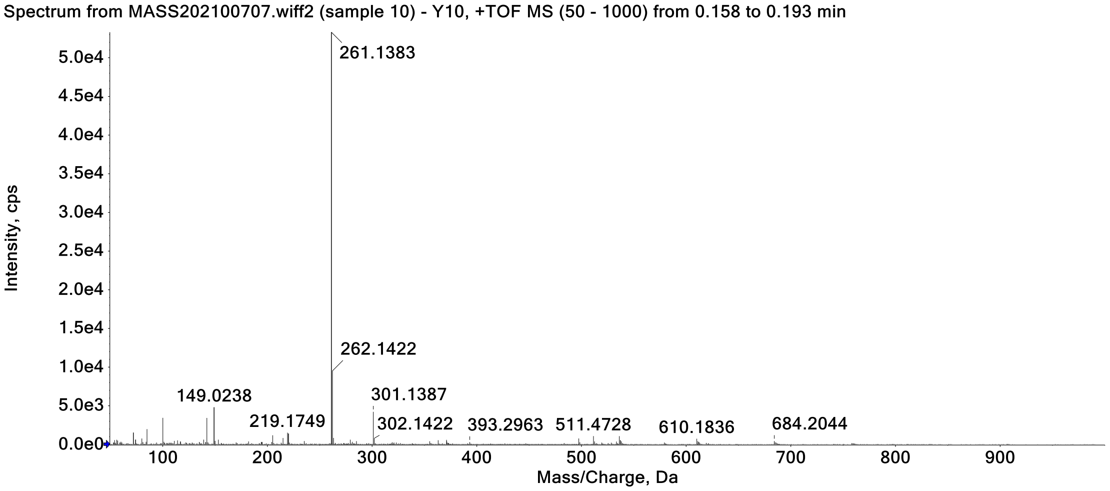

Spectrum from MASS202100707.wiff2 (sample 10) - Y10, +TOF MS (50 - 1000) from 0.158 to 0.193 min

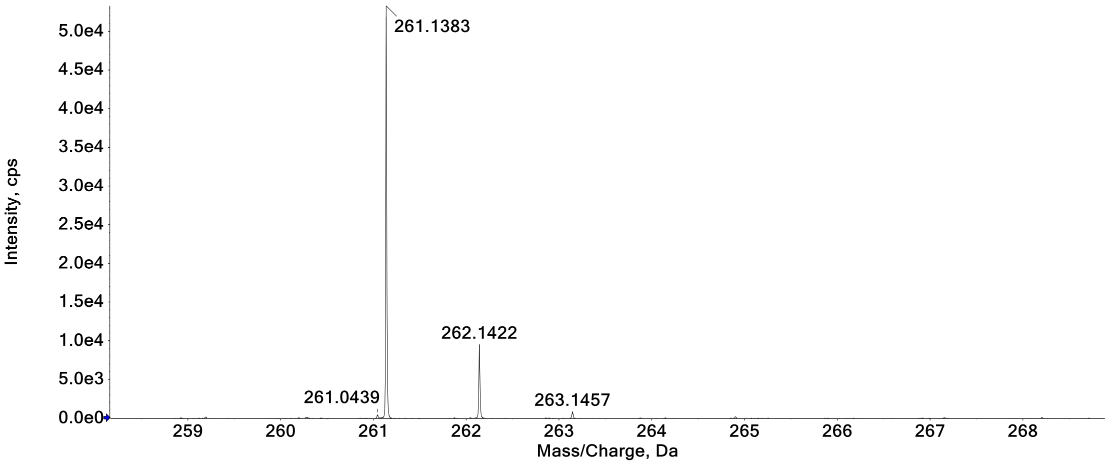

Hit	Formula	m/z	RDB	ppm	MS Rank	MSMS ppm	MSMS Rank	Found
1	C18H16N2	261.1386	12.0	-1.2	1			NA/NA

HR-MS spectrum of 1q

Spectrum from MASS20210609.wiff2 (sample 74) - Y27, +TOF MS (50 - 1000) from 0.079 to 0.114 min

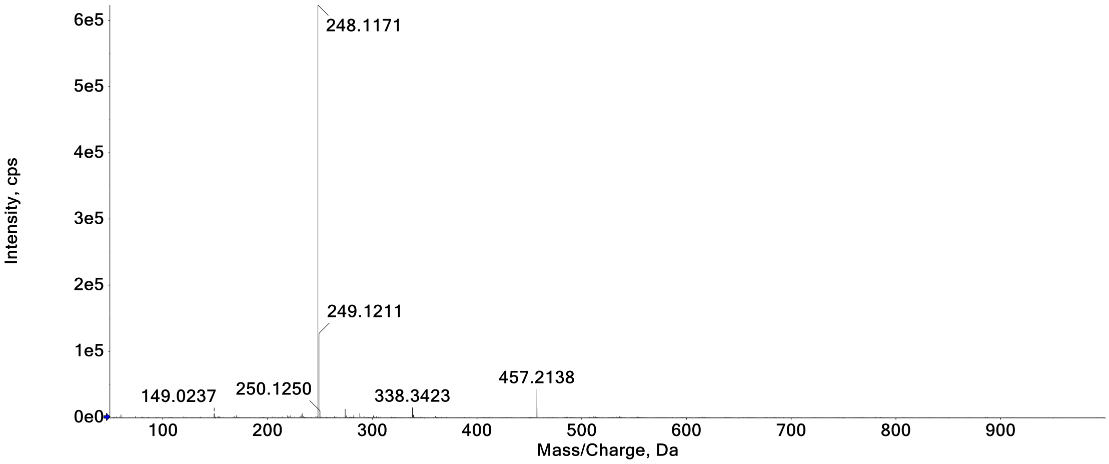

Spectrum from MASS20210609.wiff2 (sample 74) - Y27, +TOF MS (50 - 1000) from 0.079 to 0.114 min

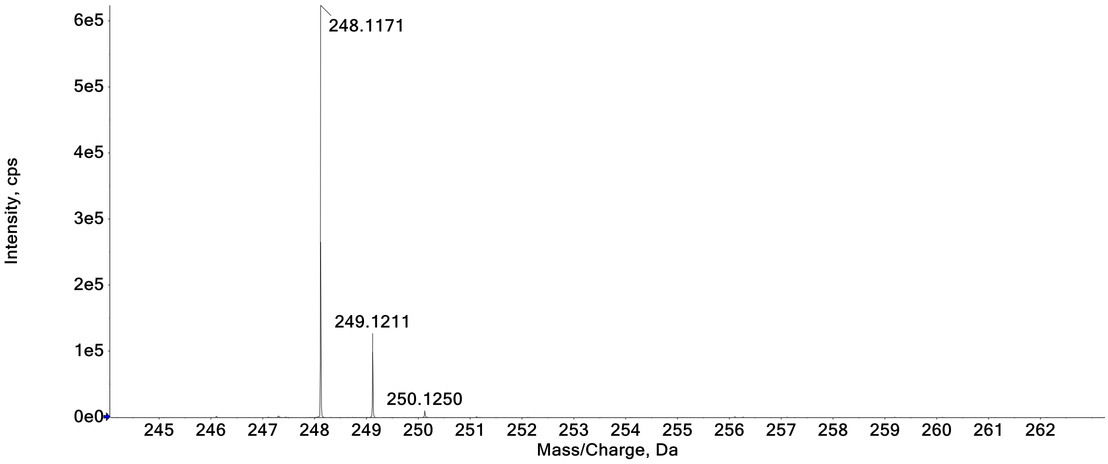

Hit	Formula	m/z	RDB	ppm	MS Rank	MSMS ppm	MSMS Rank	Found
1	C16H13N3	248.1182	12.0	-4.5	1			NA/NA

HR-MS spectrum of 1r

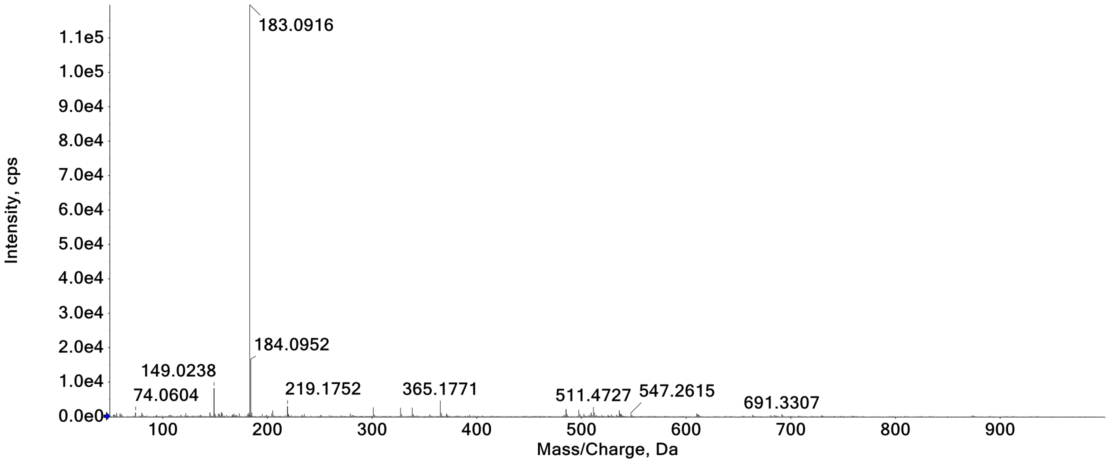

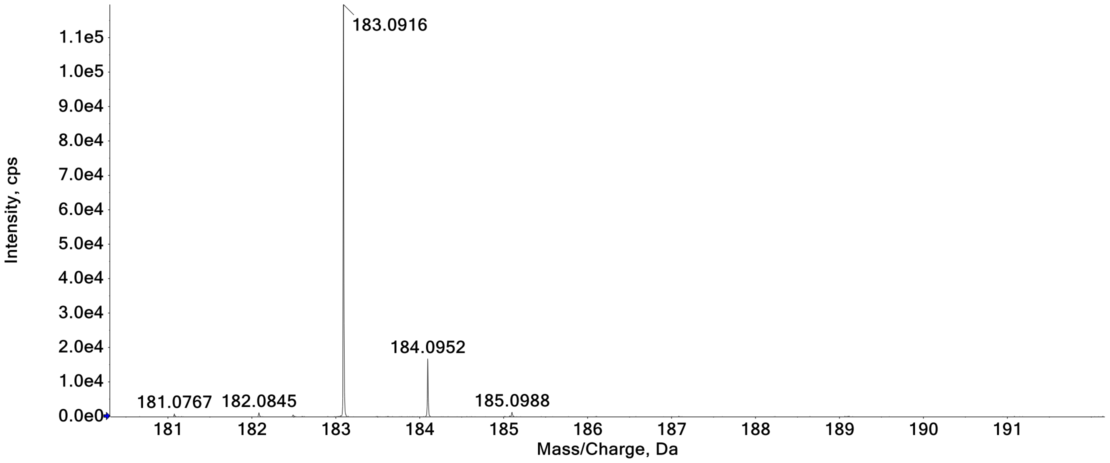

Hit	Formula	m/z	RDB	ppm	MS Rank	MSMS ppm	MSMS Rank	Found
1	C12H10N2	183.0917	9.0	-0.4	1			NA/NA

HR-MS spectrum of 1u

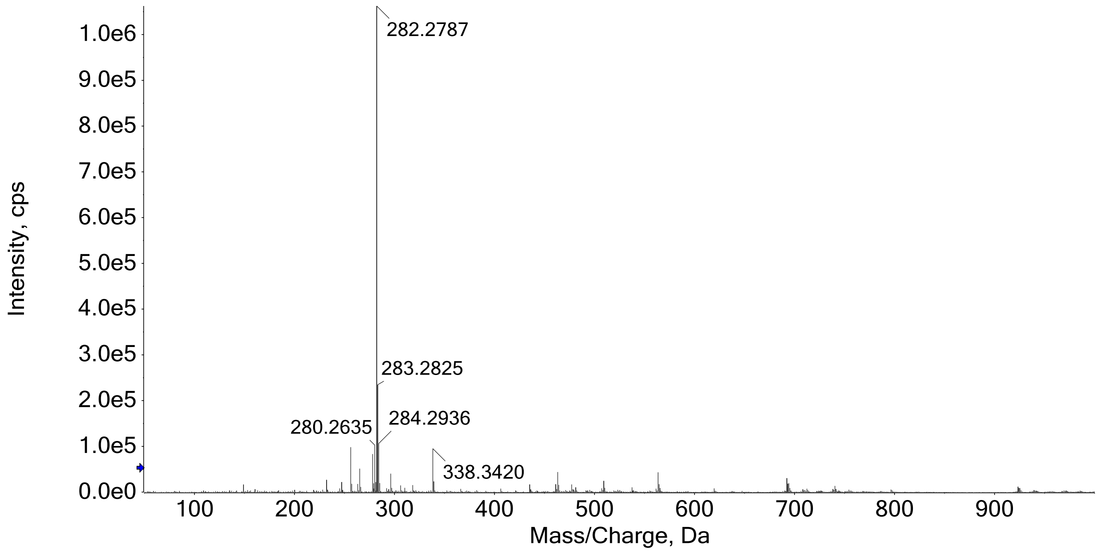

Spectrum from MASS20220928.wiff2 (sample 3) - Y2, Experiment 1, +IDA T...) - Y2, Experiment 1, +IDA TOF MS (50 - 1000) from 0.135 to 0.222 min]

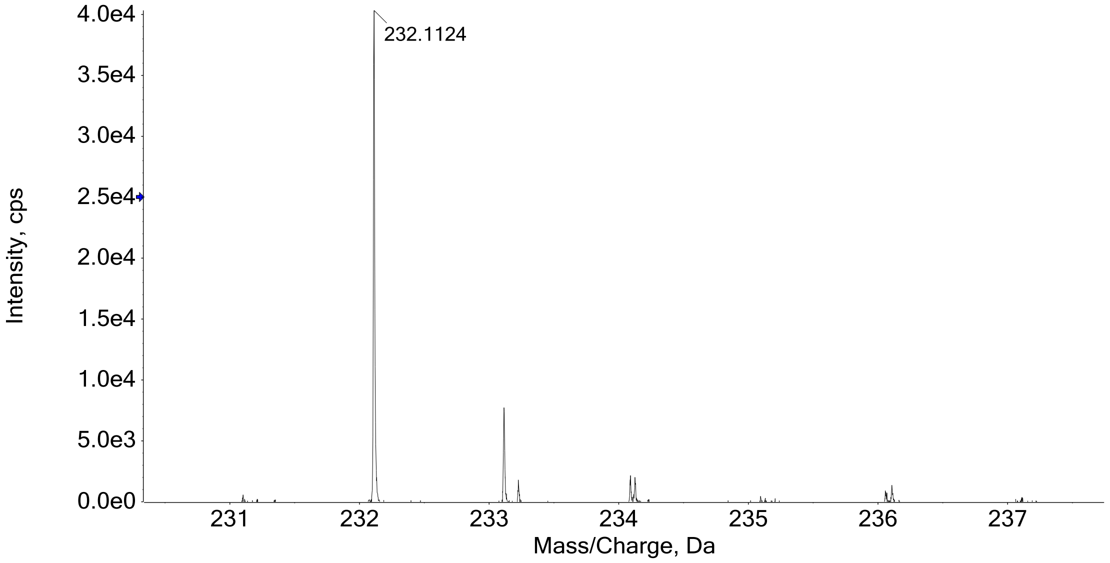

Hit	Formula	m/z	RDB	ppm	MS Rank	MSMS ppm	MSMS Rank	Found
1	C17H13N	232.1121	12.0	1.4	1			NA/NA

HR-MS spectrum of 3a

Spectrum from MASS20210609.wiff2 (sample 49) - Y2, +TOF MS (50 - 1000) from 0.132 to 0.167 min

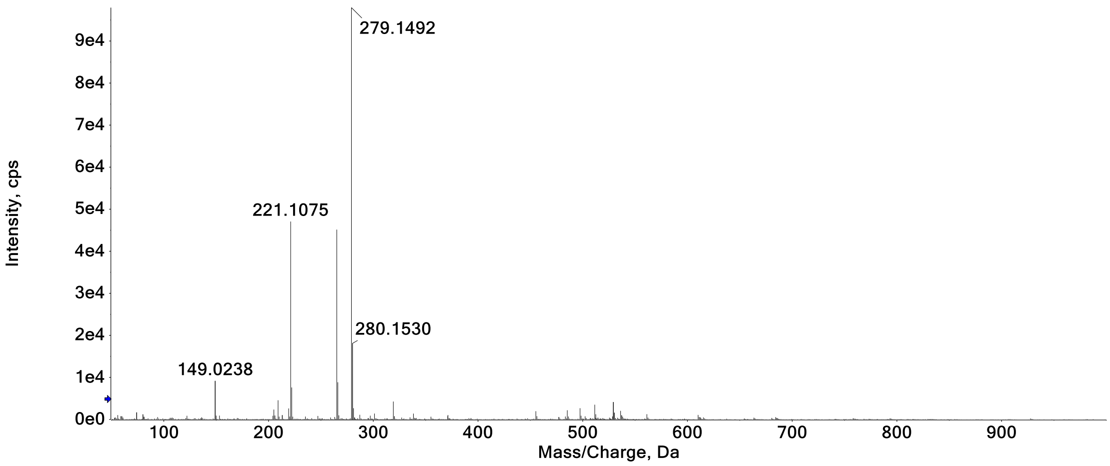

Spectrum from MASS20210609.wiff2 (sample 49) - Y2, +TOF MS (50 - 1000) from 0.132 to 0.167 min

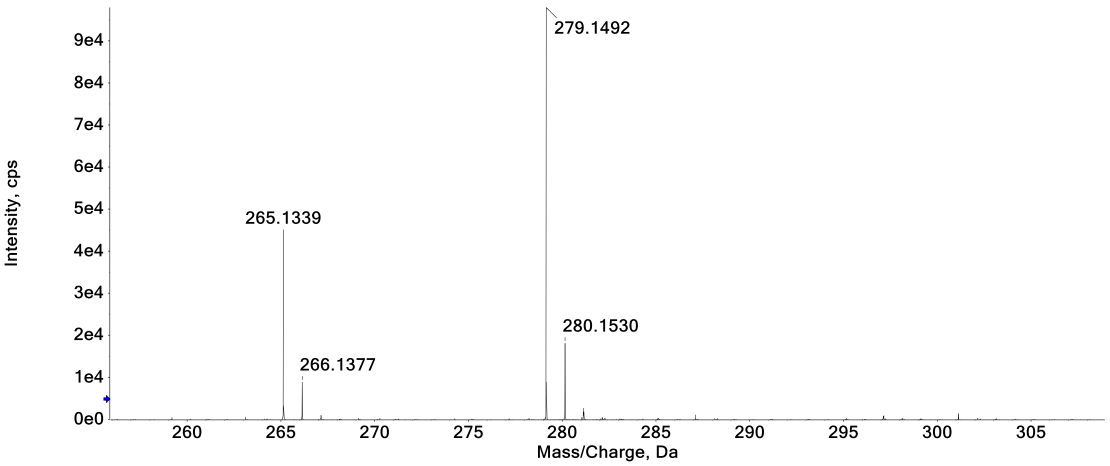

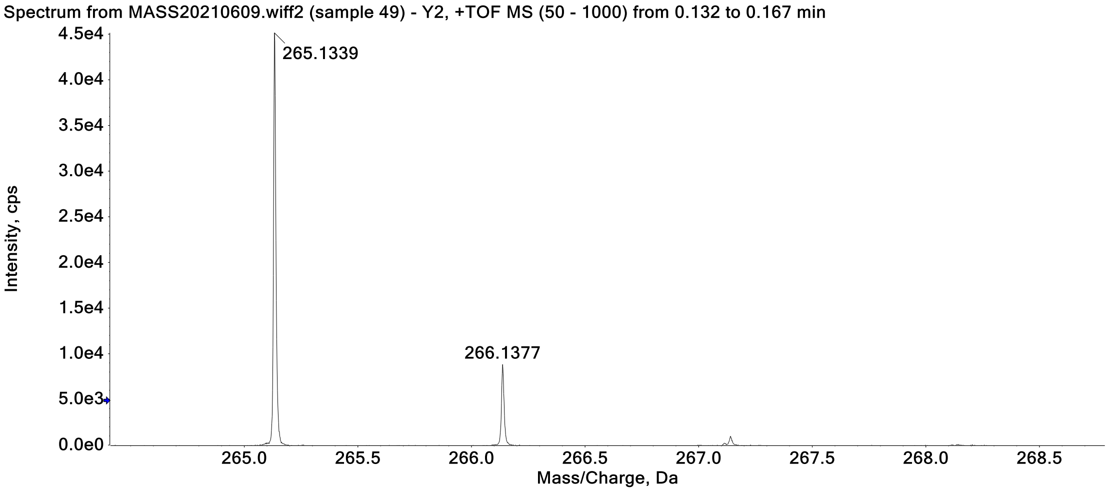

Hit	Formula	m/z	RDB	ppm	MS Rank	MSMS ppm	MSMS Rank	Found
1	C17H16N20	265.1335	11.0	1.4	1			NA/NA

HR-MS spectrum of 3c

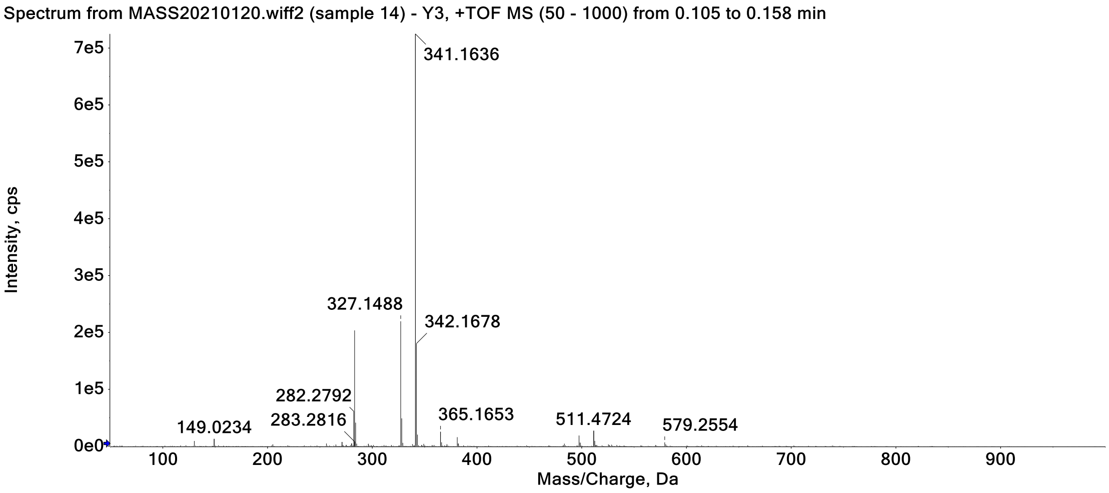

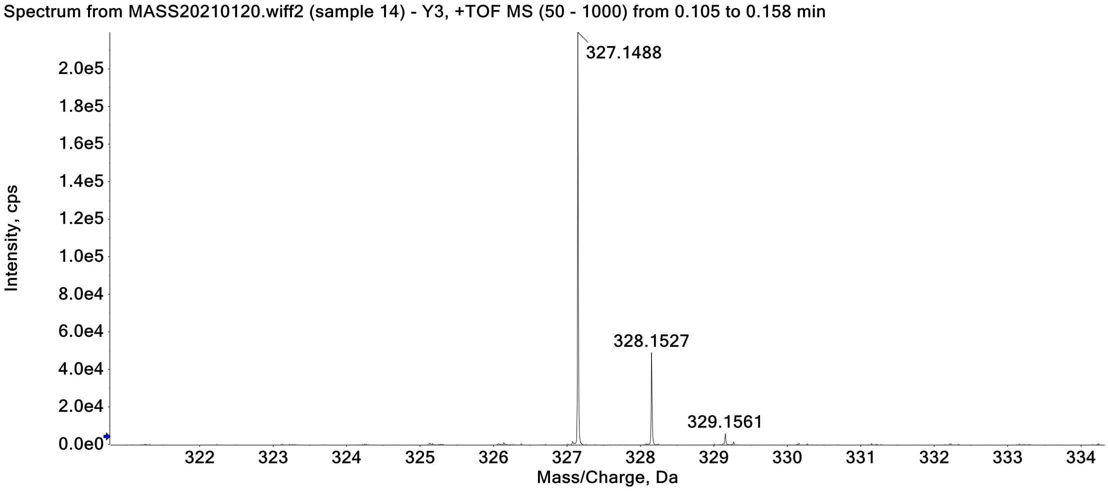

Hit	Formula	m/z	RDB	ppm	MS Rank	MSMS ppm	MSMS Rank	Found
1	C22H18N20	327.1492	15.0	-1.2	1			NA/NA

HR-MS spectrum of 3d

Spectrum from MASS202100707.wiff2 (sample 4) - Y4, +TOF MS (50 - 1000) from 0.158 to 0.193 min

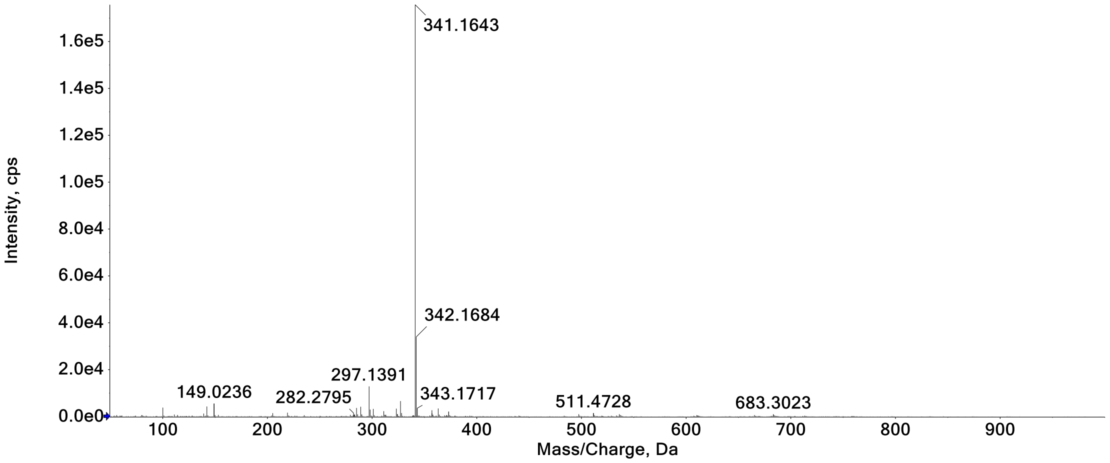

Spectrum from MASS202100707.wiff2 (sample 4) - Y4, +TOF MS (50 - 1000) from 0.158 to 0.193 min

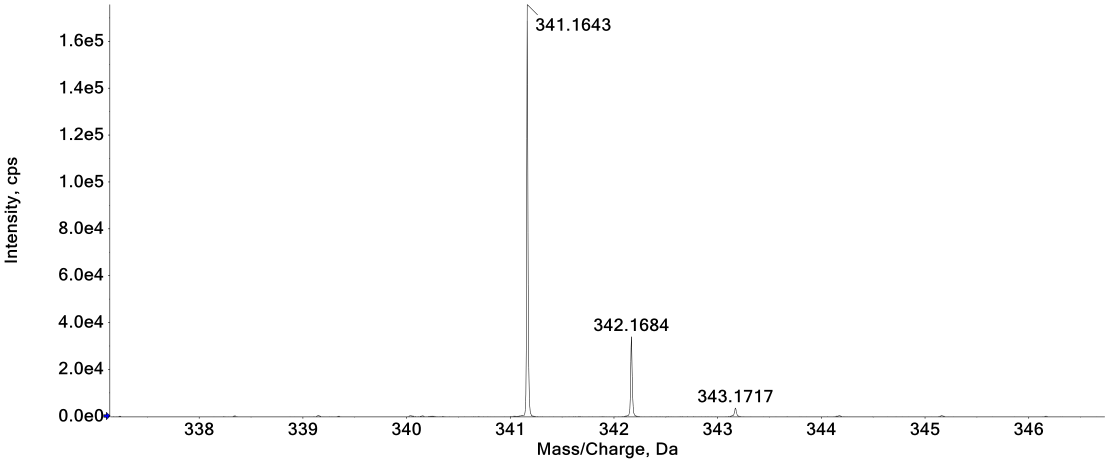

Hit	Formula	m/z	RDB	ppm	MS Rank	MSMS ppm	MSMS Rank	Found
1	C23H20N2O	341.1648	15.0	-1.6	1			NA/NA

HR-MS spectrum of 3e

Spectrum from MASS20210609.wiff2 (sample 77) - Y30, +TOF MS (50 - 1000) from 0.079 to 0.114 min

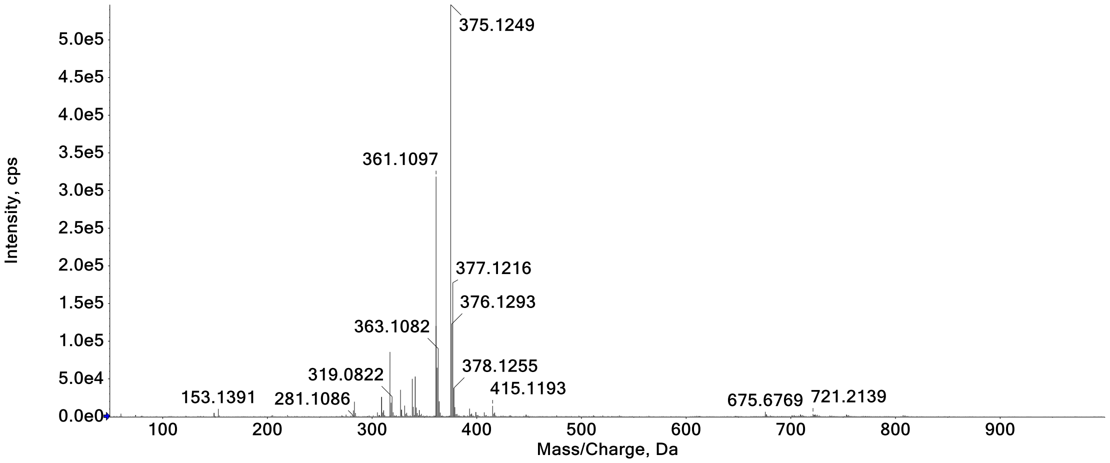

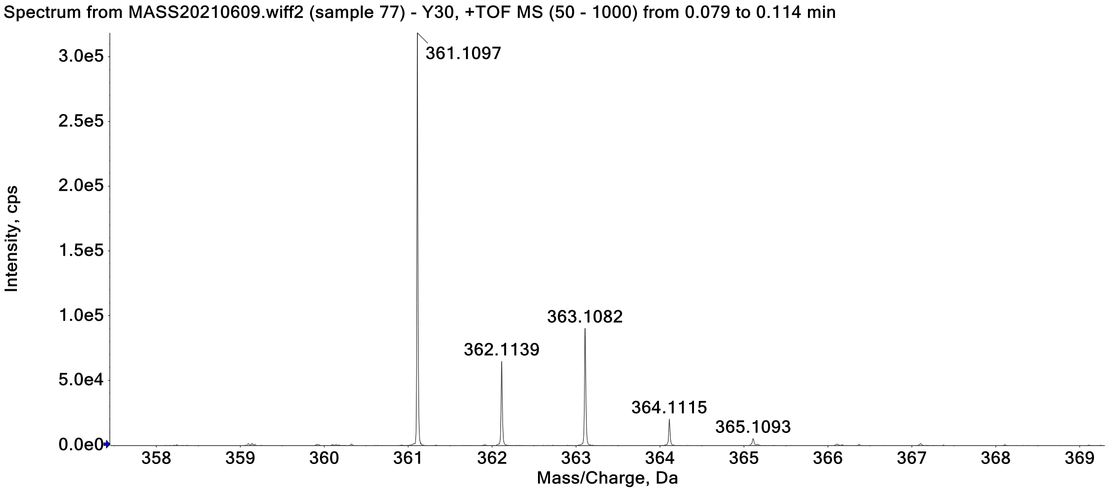

Hit	Formula	m/z	RDB	ppm	MS Rank	MSMS ppm	MSMS Rank	Found
1	C22H17ClN2O	361.1102	15.0	-1.4	1			NA/NA

HR-MS spectrum of 3f

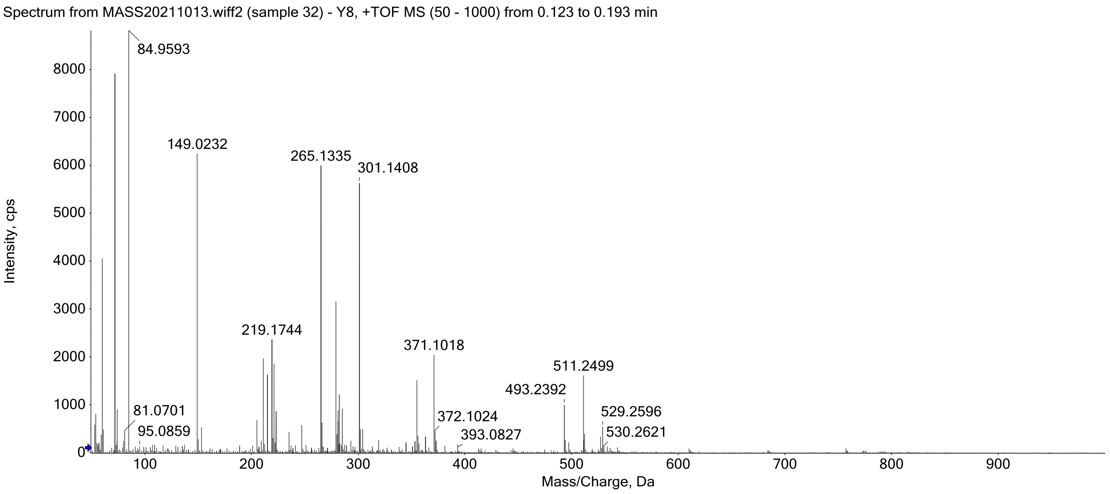

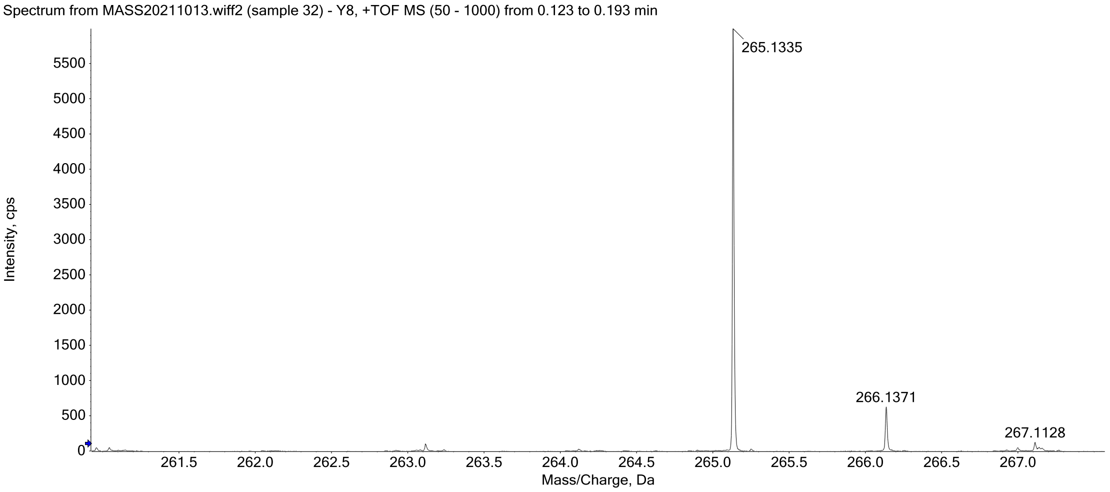

Hit	Formula	m/z	RDB	ppm	MS Rank	MSMS ppm	MSMS Rank	Found
1	C17H16N2O	265.1335	11.0	-0.1	1			NA/NA

HR-MS spectrum of 3g

Spectrum from MASS20211013.wiff2 (sample 35) - Y11, +TOF MS (50 - 1000) from 0.123 to 0.193 min

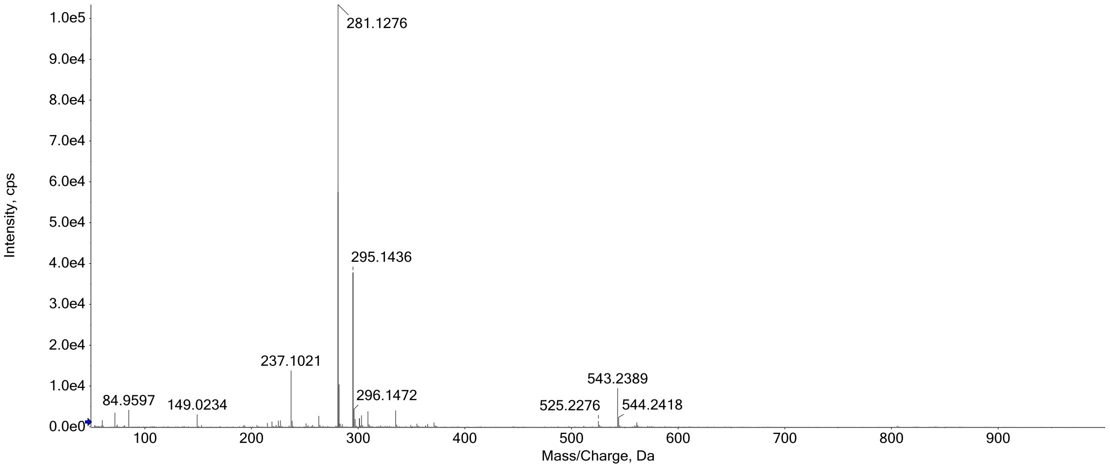

Spectrum from MASS20211013.wiff2 (sample 35) - Y11, +TOF MS (50 - 1000) from 0.123 to 0.193 min

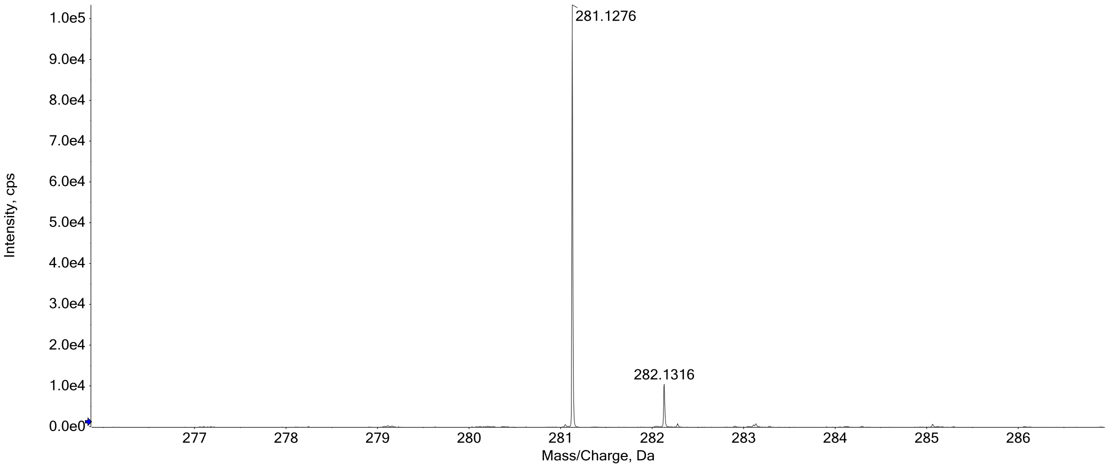

Hit	Formula	m/z	RDB	ppm	MS Rank	MSMS ppm	MSMS Rank	Found
1	C17H16N2O2	281.1285	11.0	-3.0	1			NA/NA

HR-MS spectrum of 3h

Spectrum from MASS20211013.wiff2 (sample 29) - Y5, +TOF MS (50 - 1000) from 0.088 to 0.158 min

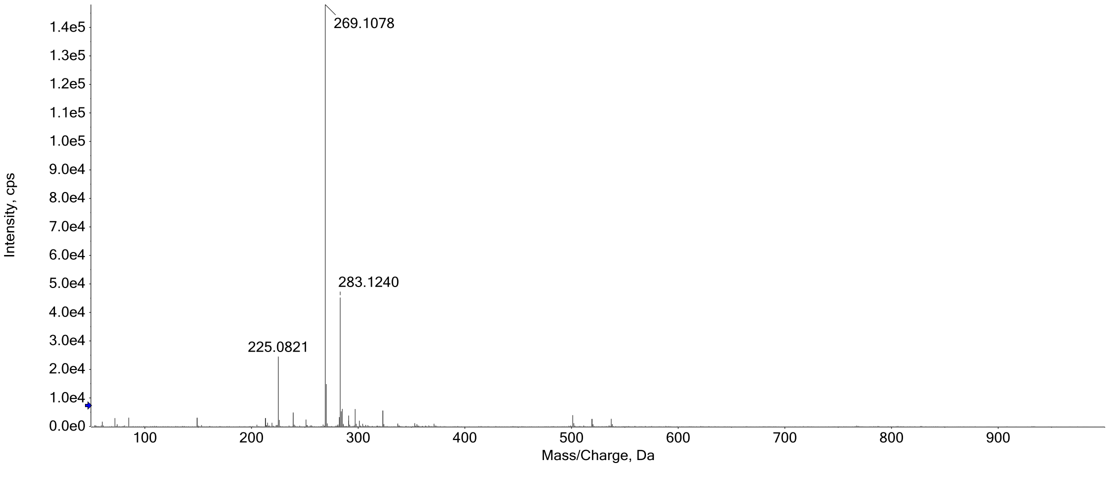

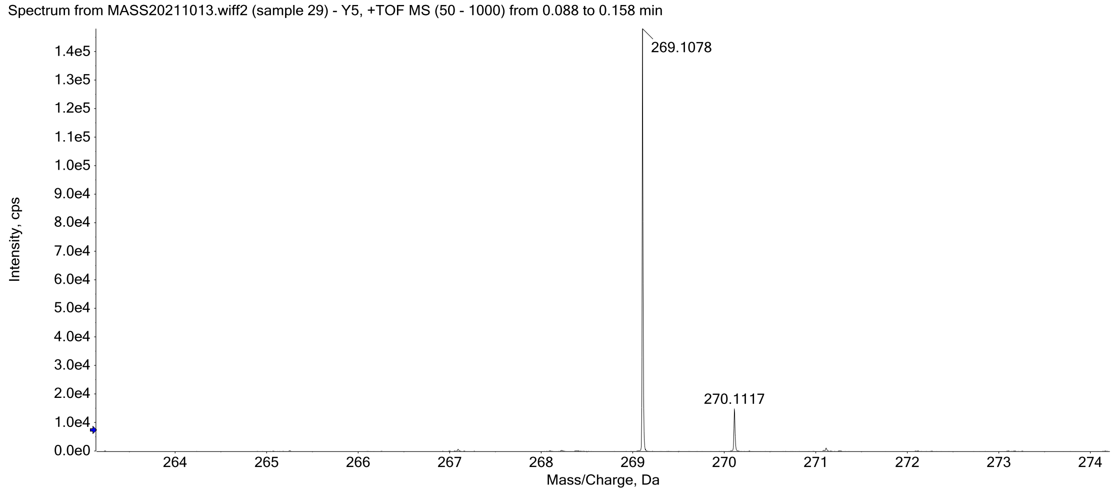

Hit	Formula	m/z	RDB	ppm	MS Rank	MSMS ppm	MSMS Rank	Found
1	C16H13FN2O	269.1085	11.0	-2.5	1			NA/NA

HR-MS spectrum of 3i

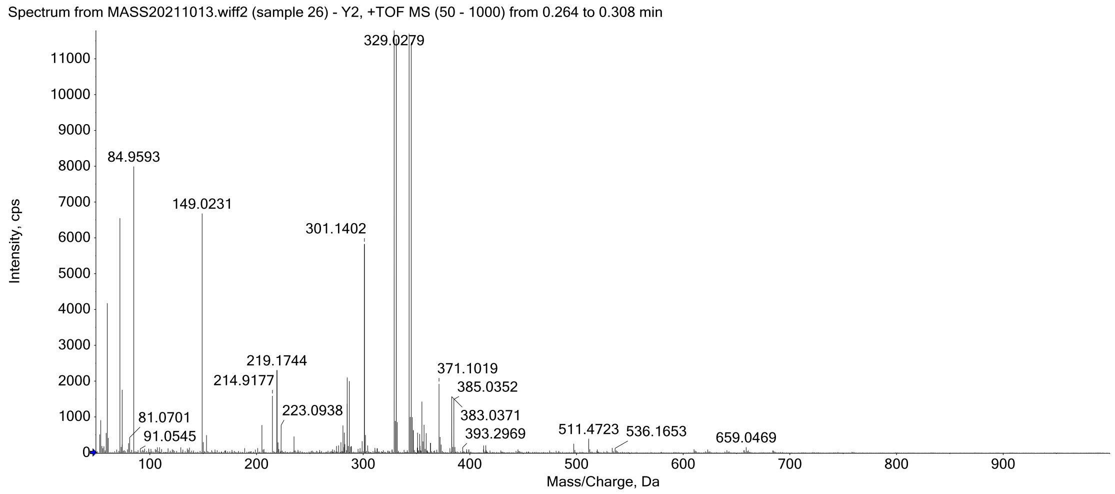

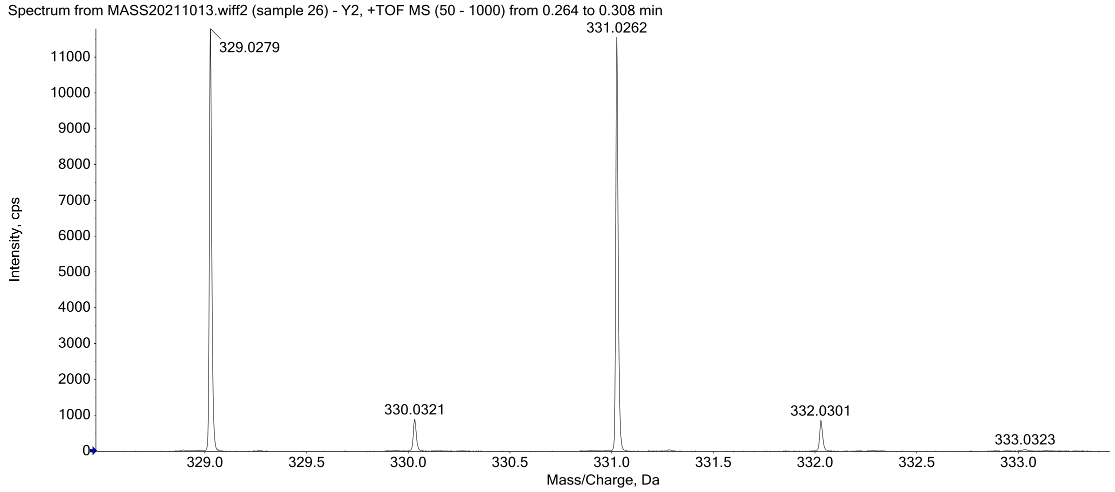

Hit	Formula	m/z	RDB	ppm	MS Rank	MSMS ppm	MSMS Rank	Found
1	C16H13BrN2O	329.0284	11.0	-1.5	1			NA/NA

HR-MS spectrum of 3j

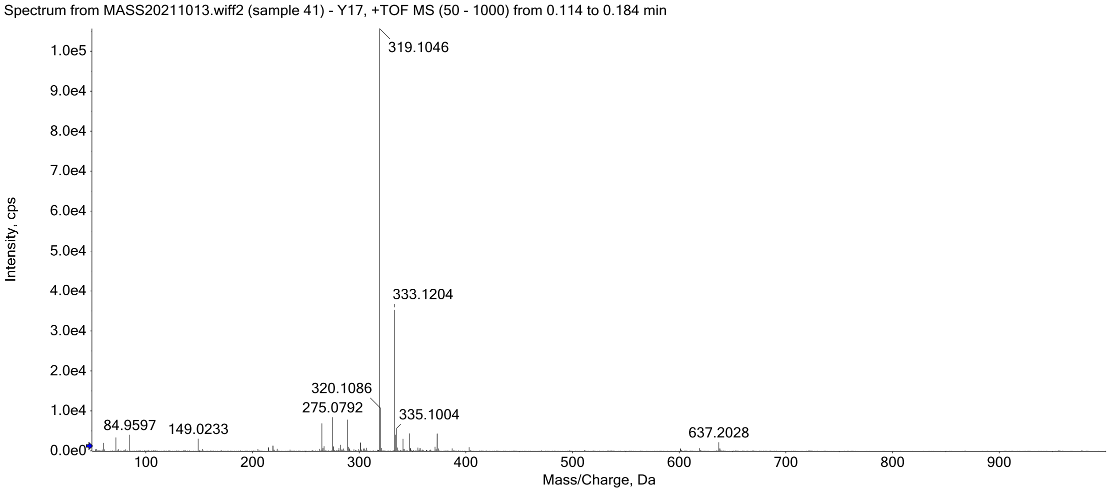

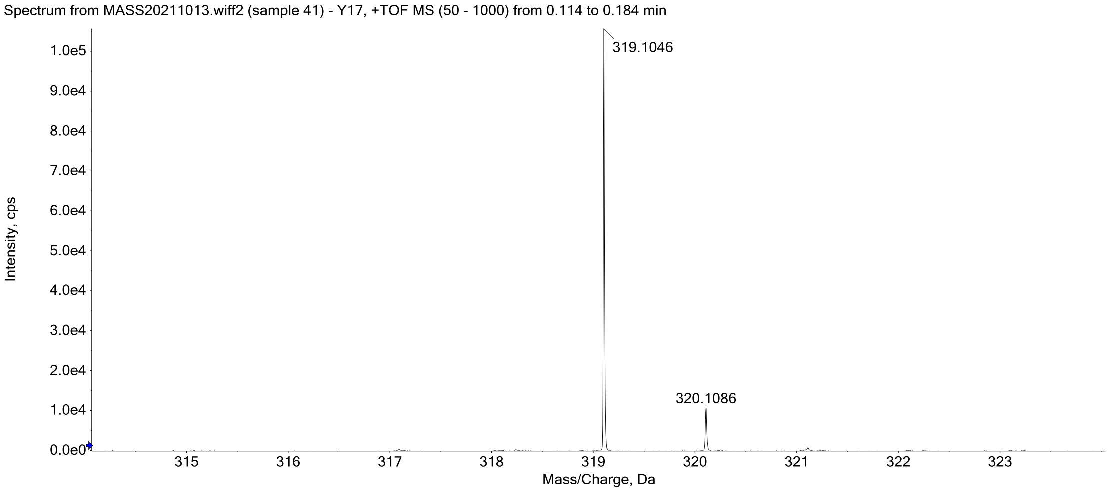

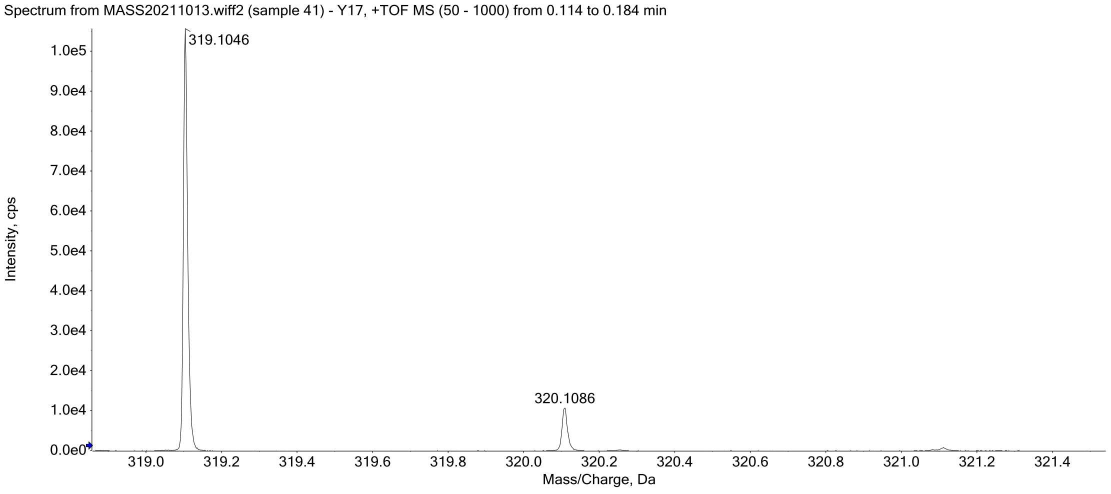

HR-MS spectrum of 3k

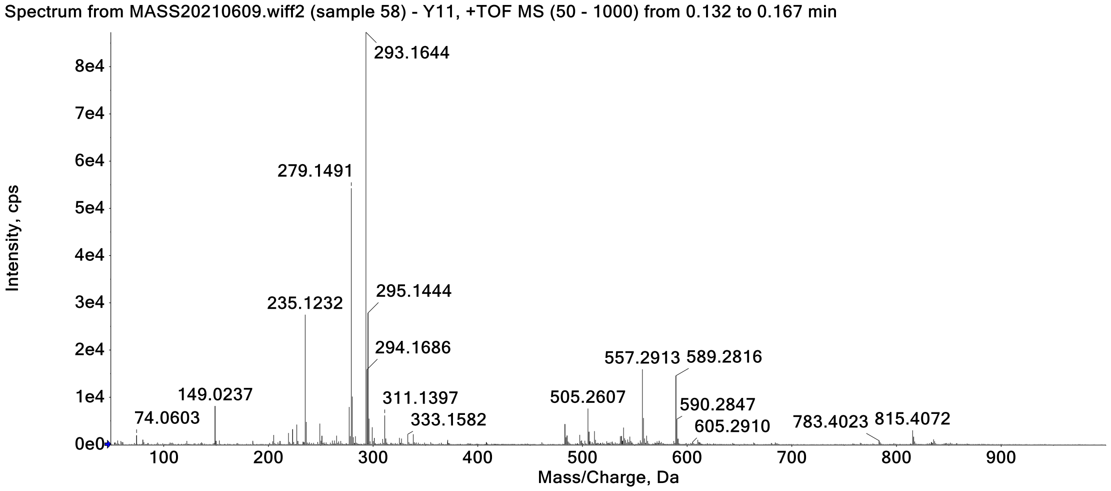

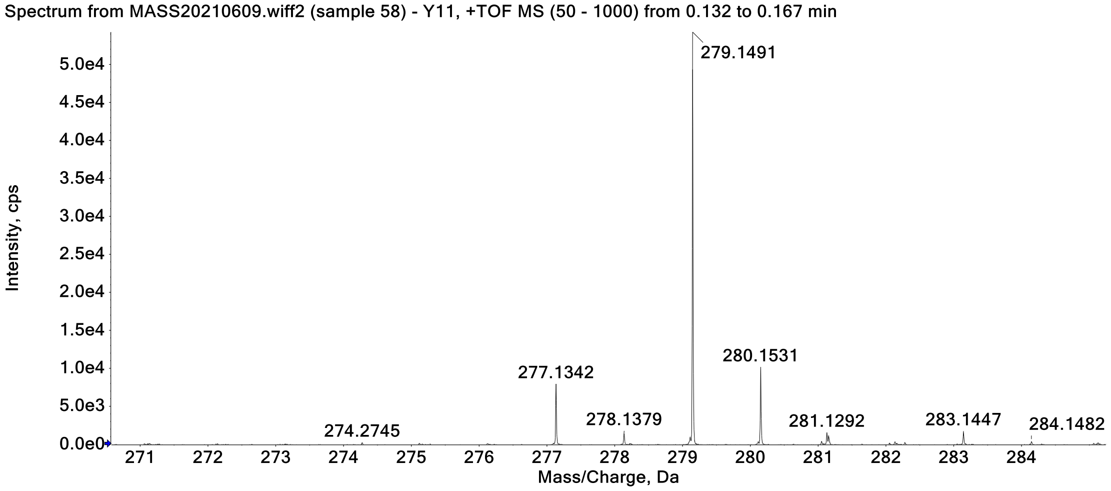

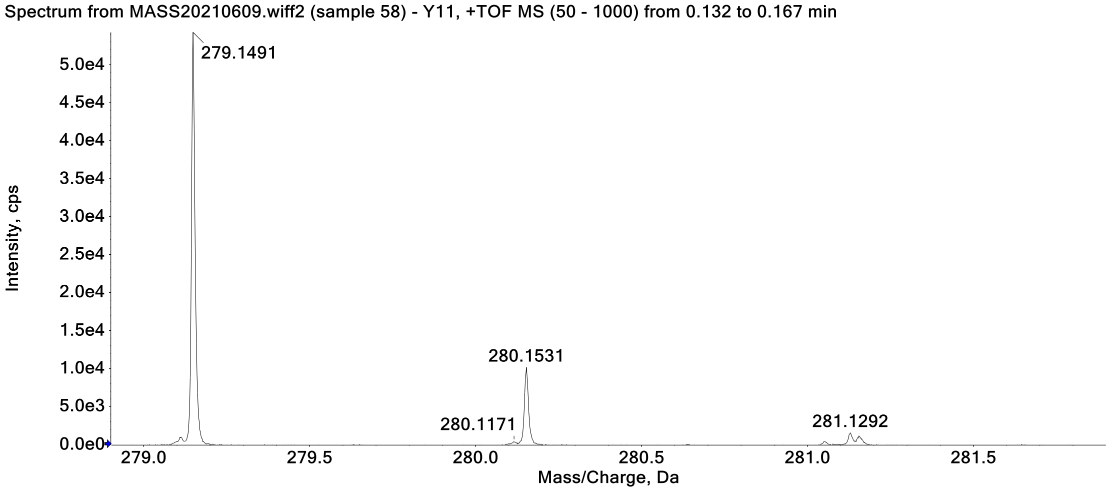

Hit	Formula	m/z	RDB	ppm	MS Rank	MSMS ppm	MSMS Rank	Found
1	C18H18N2O	279.1492	11.0	-0.3	1			NA/NA

HR-MS spectrum of 3l

Spectrum from MASS20210609.wiff2 (sample 61) - Y14, +TOF MS (50 - 1000) from 0.132 to 0.167 min

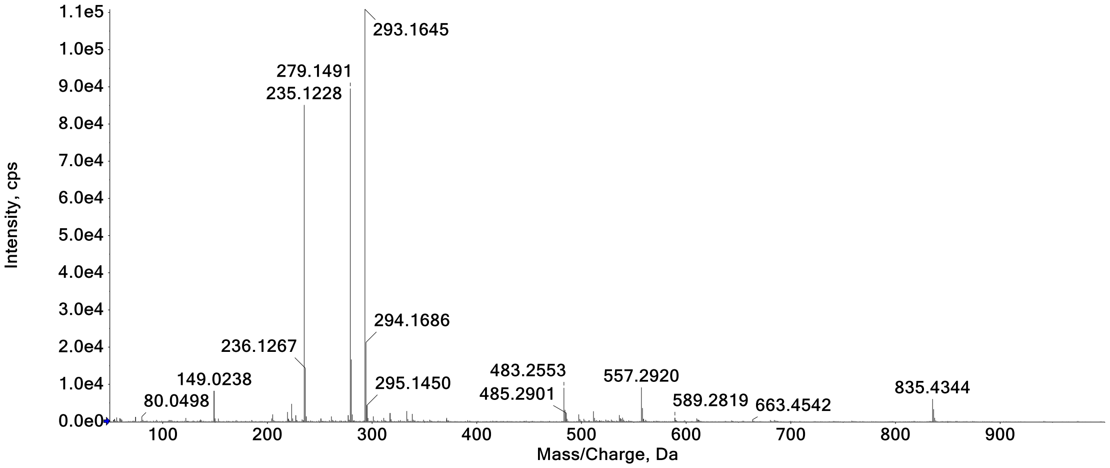

Spectrum from MASS20210609.wiff2 (sample 61) - Y14, +TOF MS (50 - 1000) from 0.132 to 0.167 min

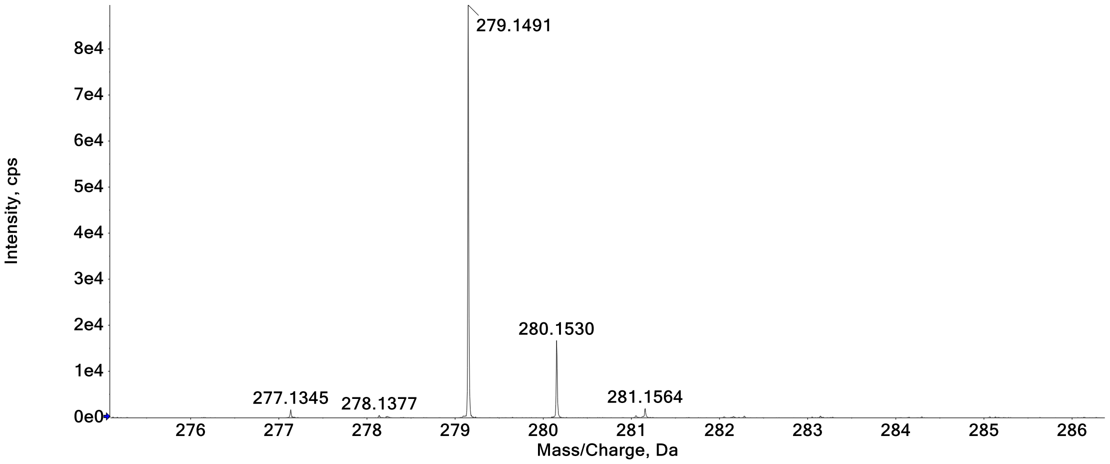

Hit	Formula	m/z	RDB	ppm	MS Rank	MSMS ppm	MSMS Rank	Found
1	C18H18N2O	279.1492	11.0	-0.3	1			NA/NA

HR-MS spectrum of 3m

Spectrum from MASS20210609.wiff2 (sample 54) - Y7, +TOF MS (50 - 1000) from 0.132 to 0.167 min

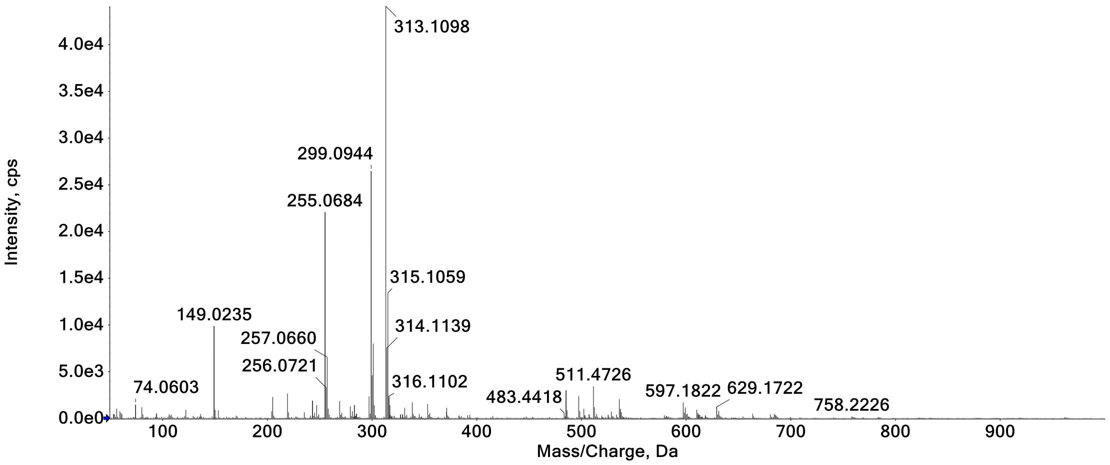

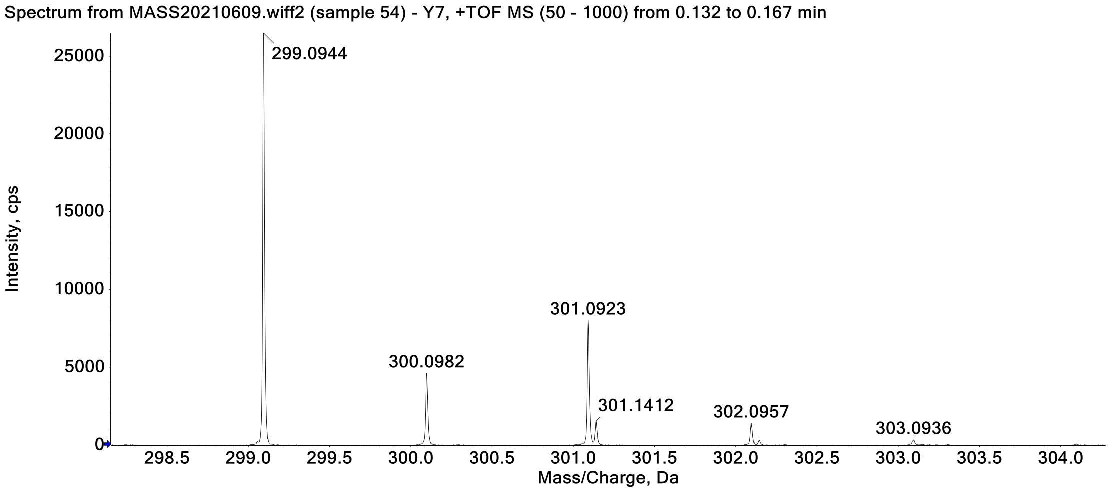

Hit	Formula	m/z	RDB	ppm	MS Rank	MSMS ppm	MSMS Rank	Found
1	C17H15ClN2O	299.0946	11.0	-0.6	1			NA/NA

HR-MS spectrum of 3n

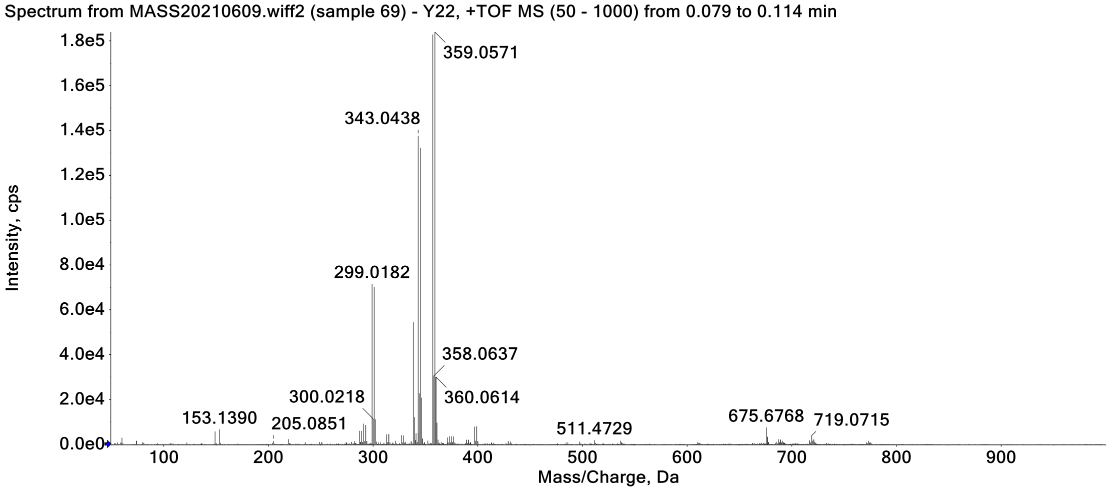

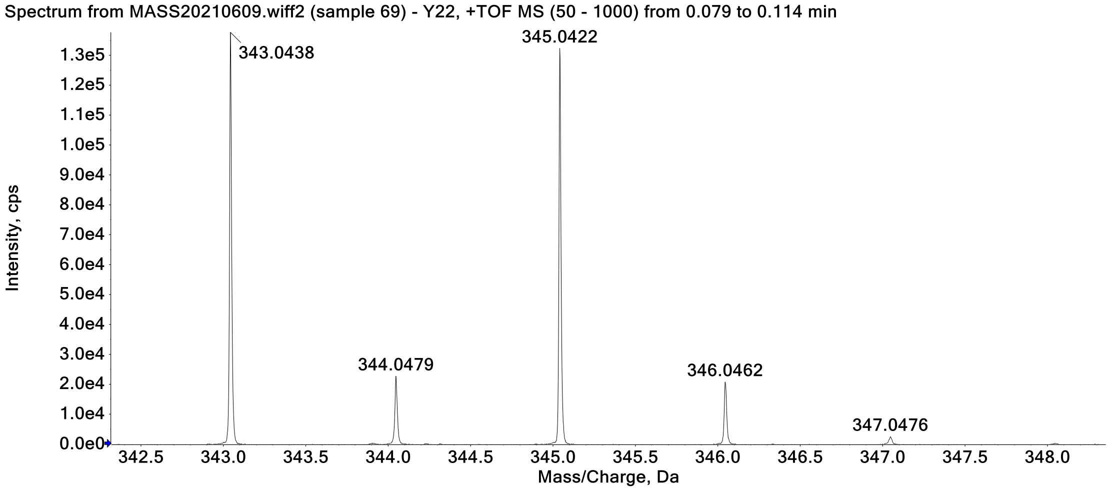

Hit	Formula	m/z	RDB	ppm	MS Rank	MSMS ppm	MSMS Rank	Found
1	C17H15BrN2O	343.0441	11.0	-0.7	1			NA/NA

HR-MS spectrum of 3o

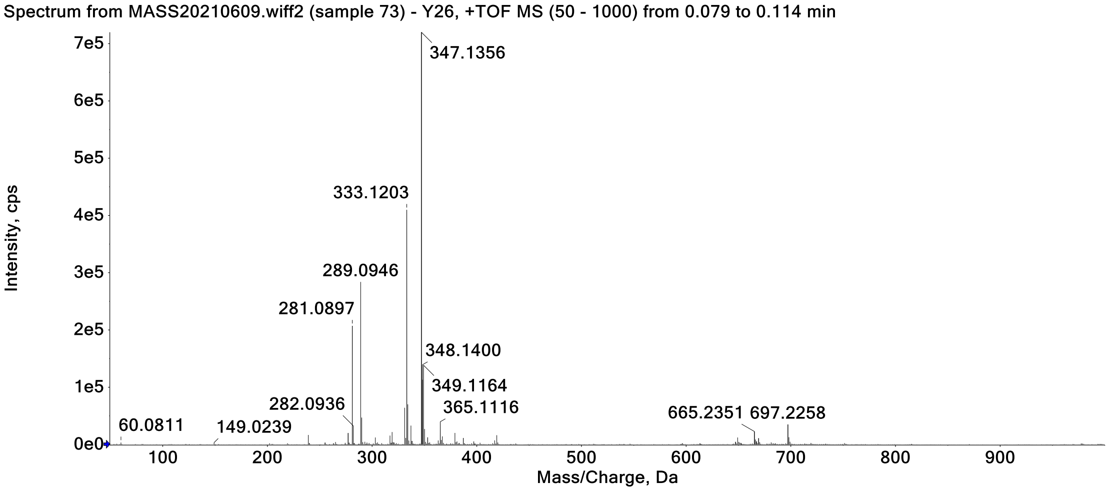

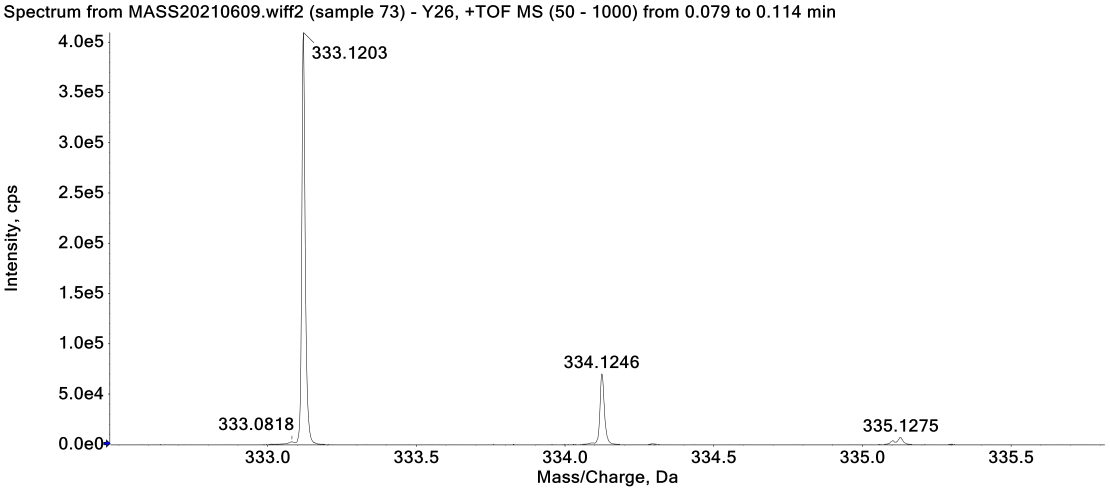

Hit	Formula	m/z	RDB	ppm	MS Rank	MSMS ppm	MSMS Rank	Found
1	C18H15F3N2O	333.1209	11.0	-1.9	1			NA/NA

HR-MS spectrum of 3p

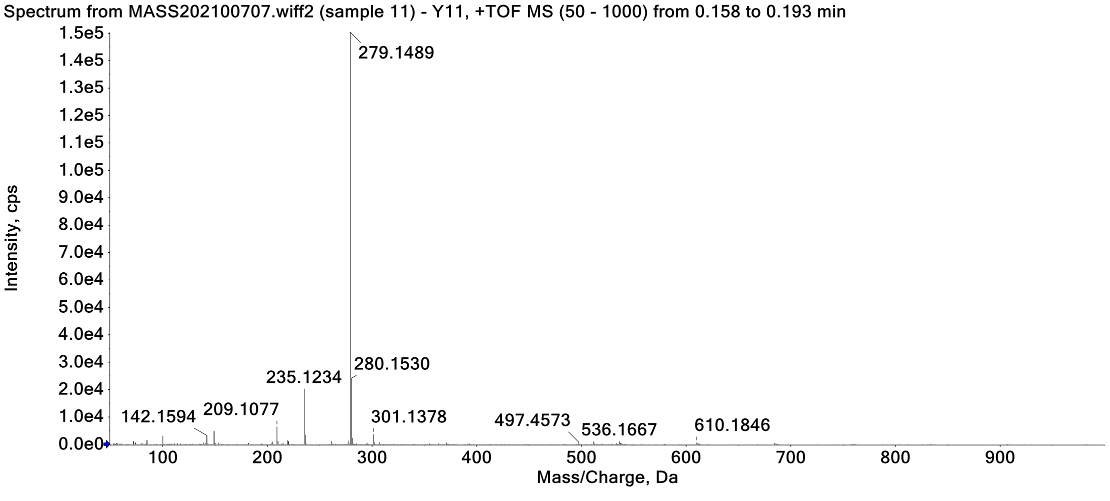

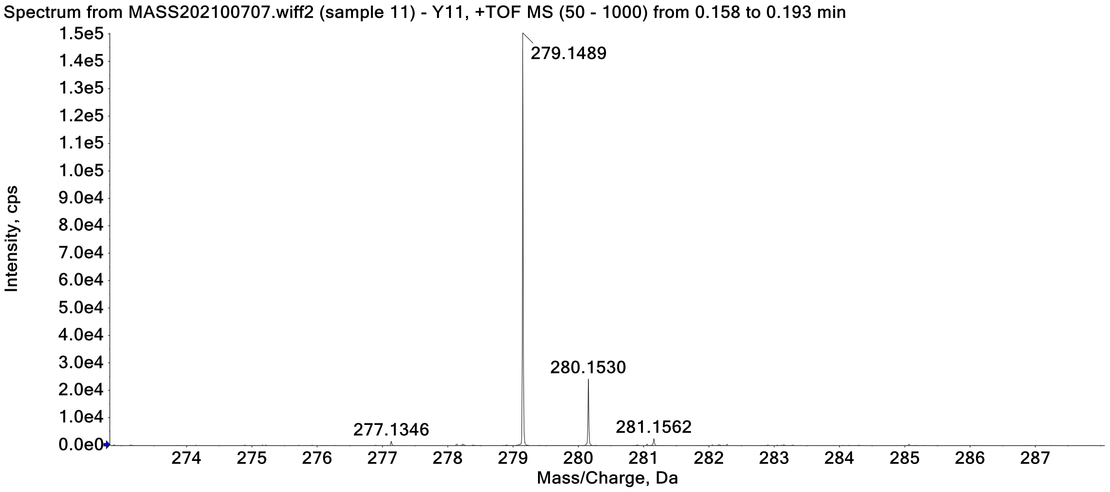

Hit	Formula	m/z	RDB	ppm	MS Rank	MSMS ppm	MSMS Rank	Found
1	C18H18N2O	279.1492	11.0	-1.0	1			NA/NA

HR-MS spectrum of 3r

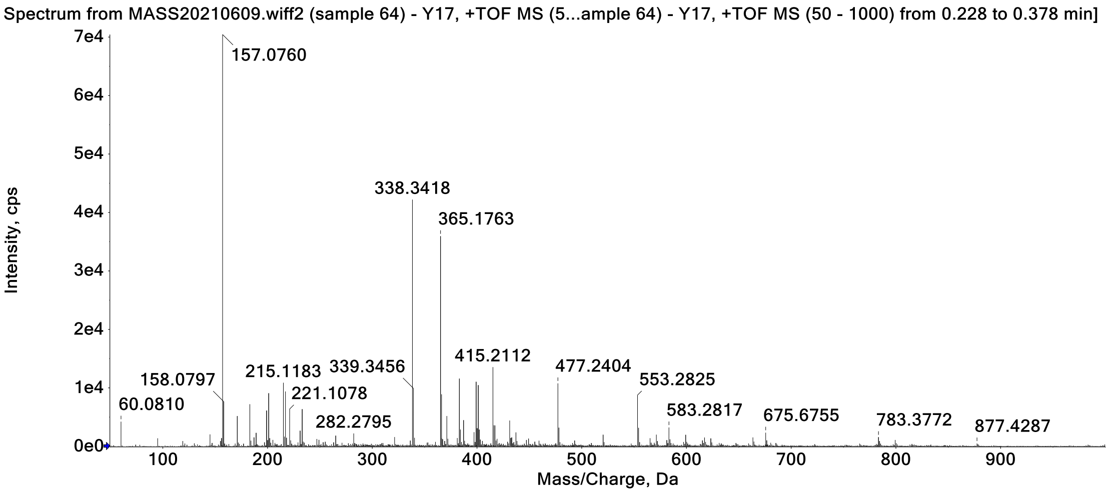

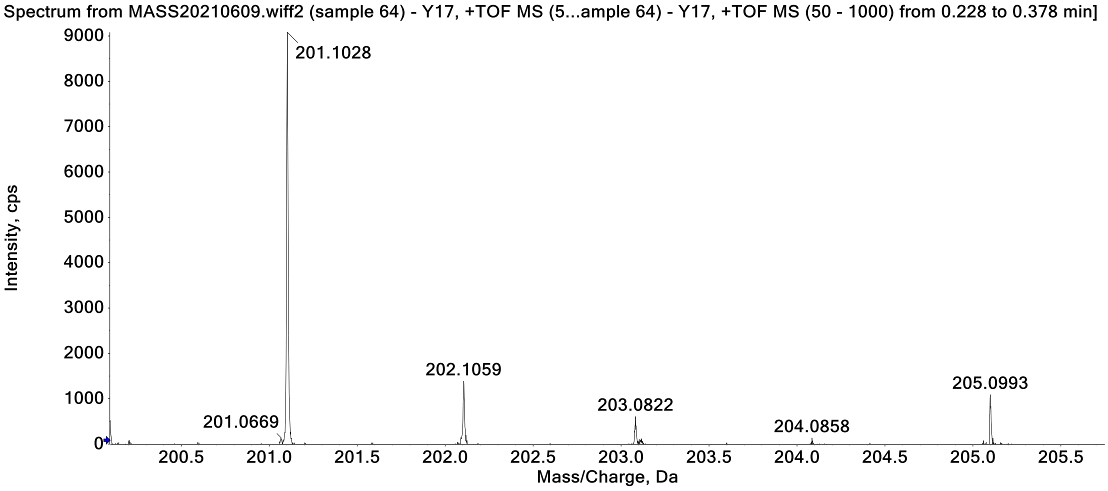

Hit	Formula	m/z	RDB	ppm	MS Rank	MSMS ppm	MSMS Rank	Found
1	C12H12N2O	201.1022	8.0	2.8	1			NA/NA

HR-MS spectrum of 3u´

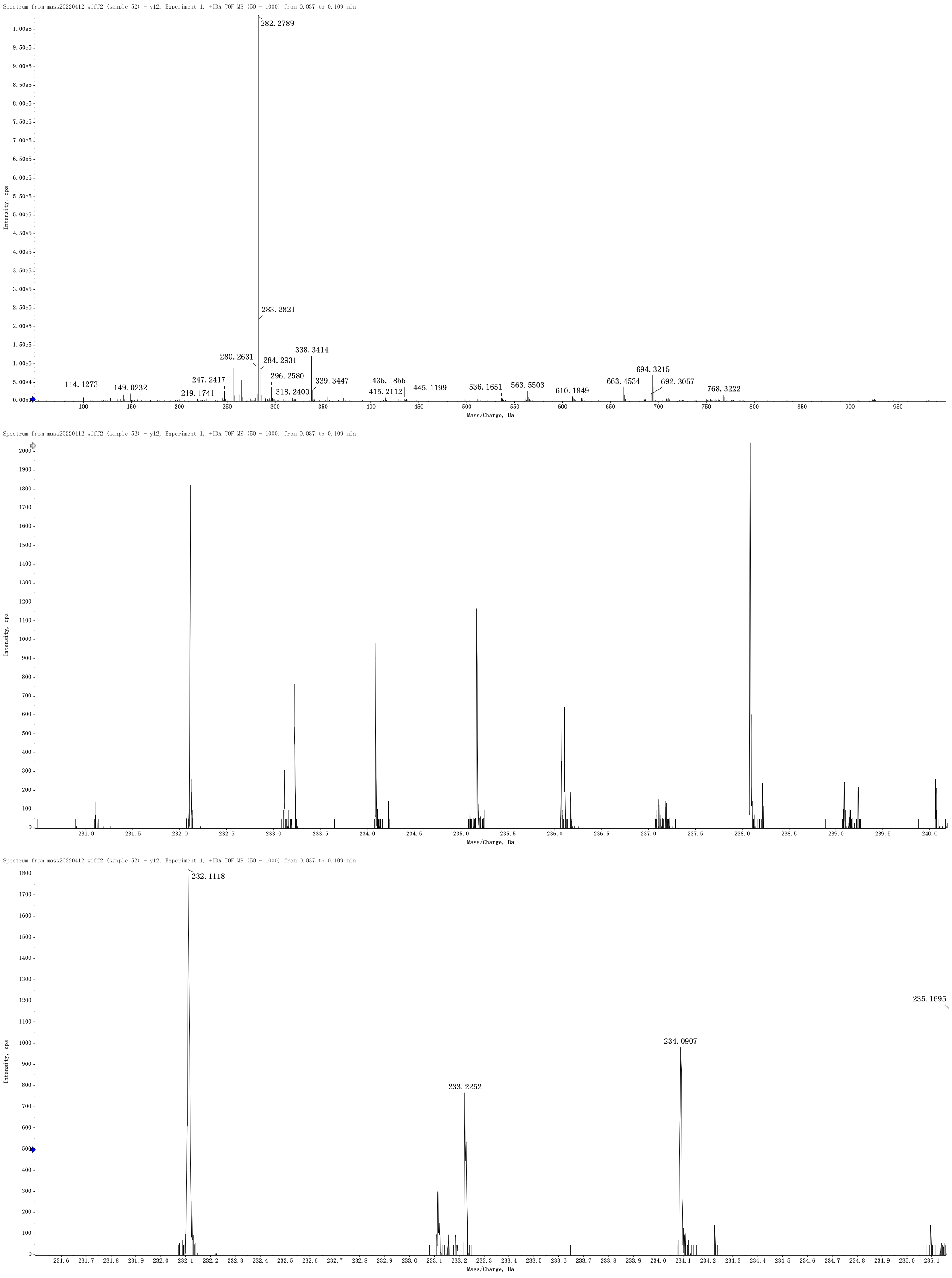

Hit	Formula	m/z	RDB	ppm	MS Rank	MSMS ppm	MSMS Rank	Found
1	C17H13N	232.1121	12.0	-1.2	1			NA/NA

HR-MS spectrum of 4a

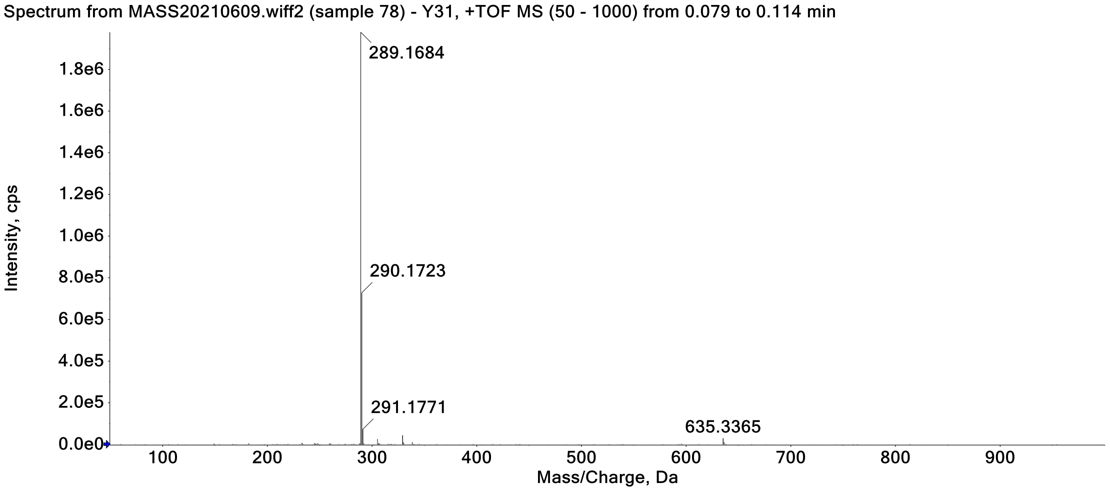

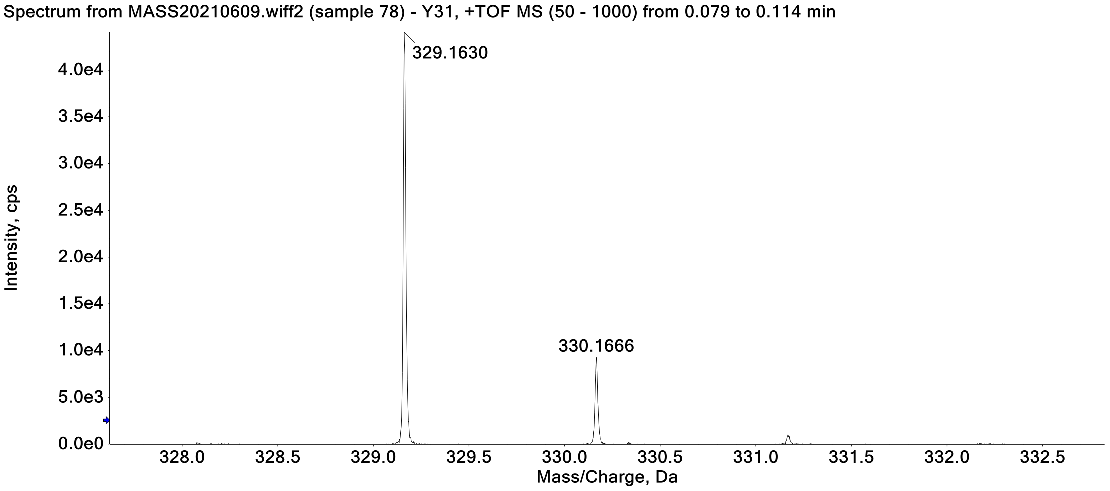

Hit	Formula	m/z	RDB	ppm	MS Rank	MSMS ppm	MSMS Rank	Found
1	C20H22N2O	329.1624	11.0	1.7	1			NA/NA

HR-MS spectrum of 4b

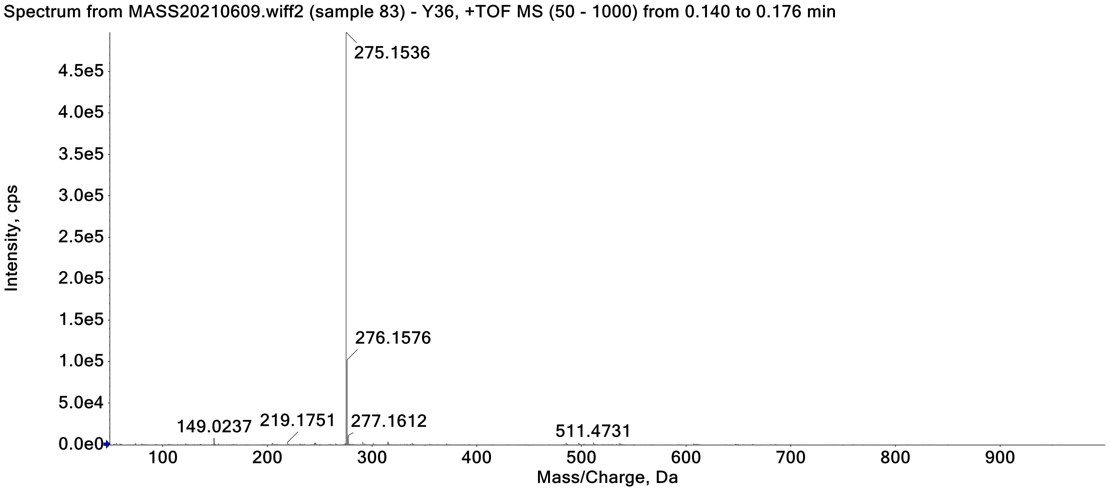

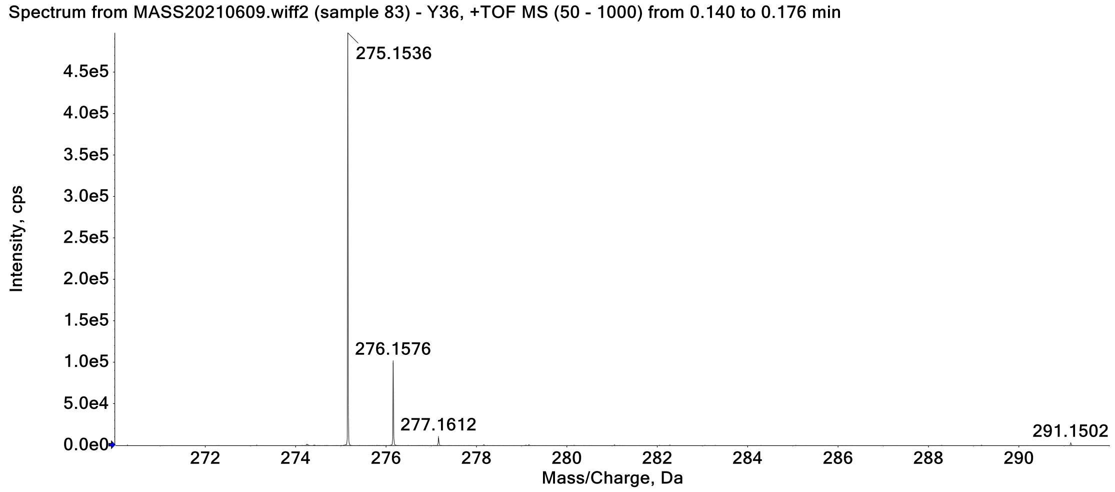

Hit	Formula	m/z	RDB	ppm	MS Rank	MSMS ppm	MSMS Rank	Found
1	C19H18N2	275.1543	12.0	-2.5	1			NA/NA

HR-MS spectrum of 4c

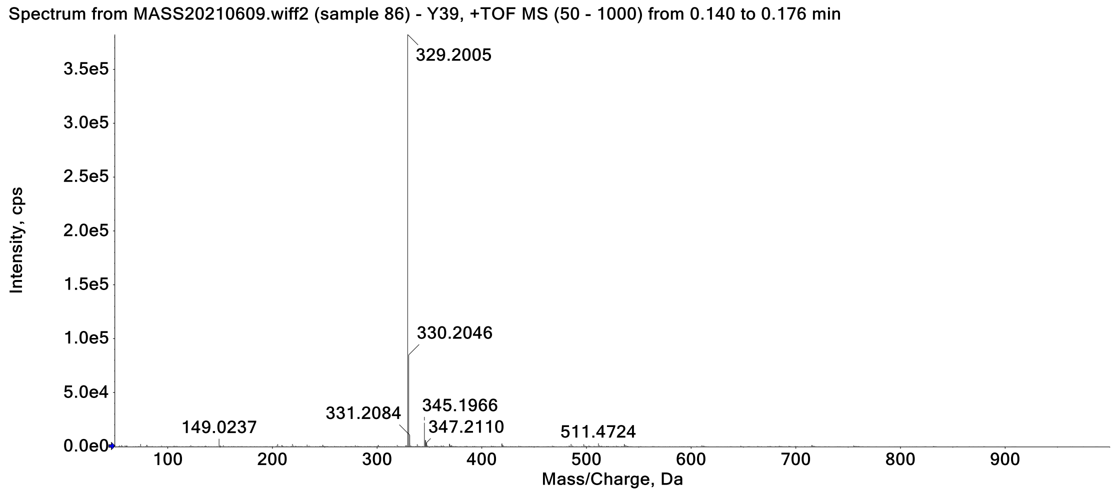

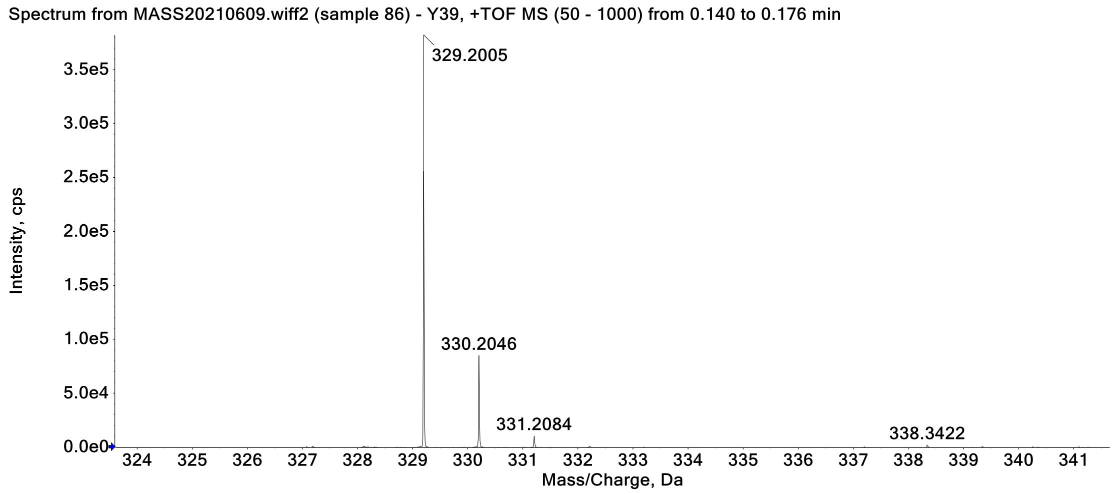

Hit	Formula	m/z	RDB	ppm	MS Rank	MSMS ppm	MSMS Rank	Found
1	C23H24N2	329.2012	13.0	-2.2	1			NA/NA

HR-MS spectrum of 4d

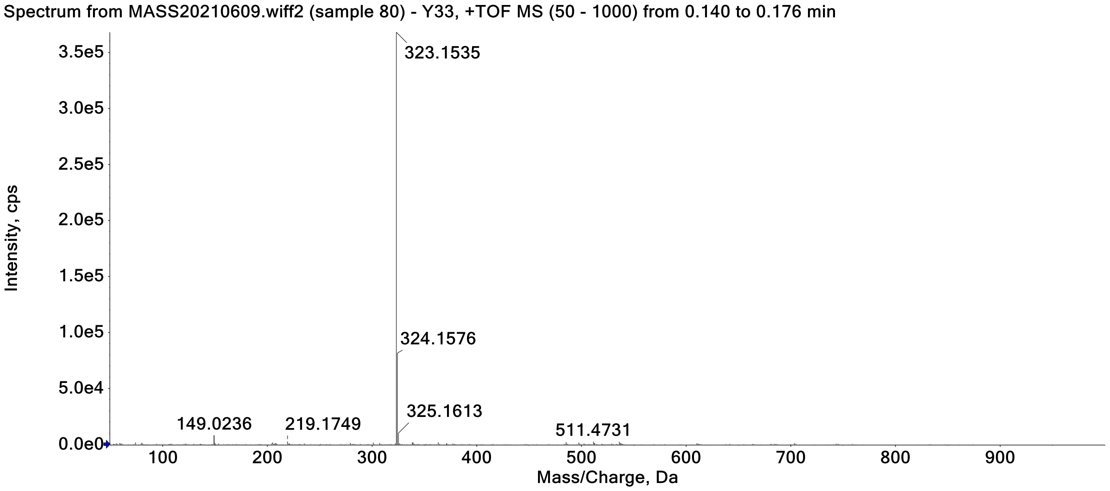

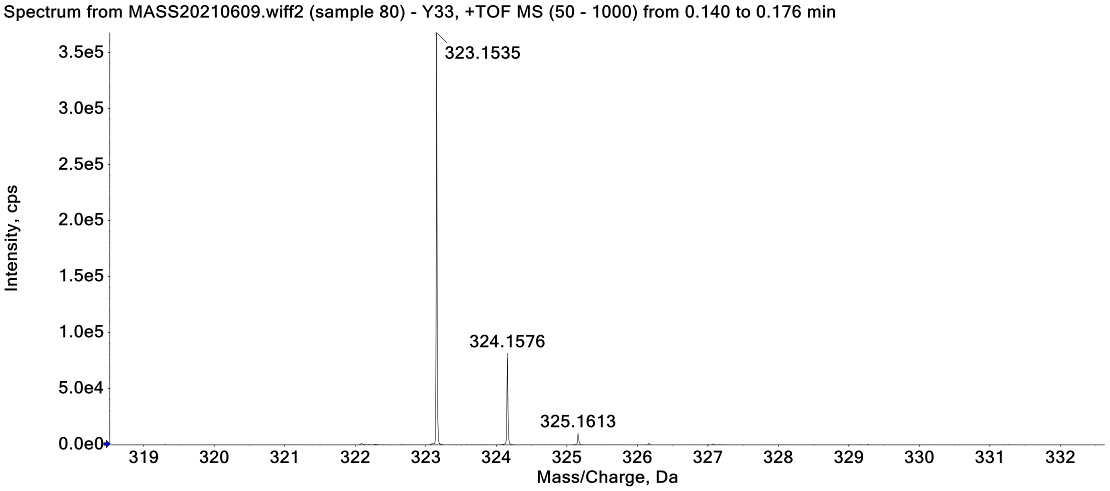

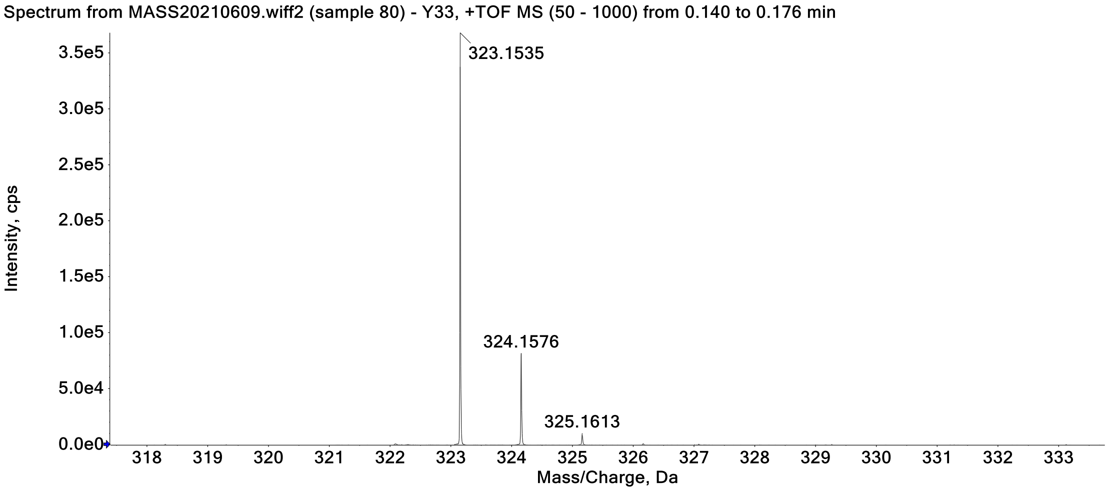

Hit	Formula	m/z	RDB	ppm	MS Rank	MSMS ppm	MSMS Rank	Found
1	C23H18N2	323.1543	16.0	-2.4	1			NA/NA

HR-MS spectrum of 4e

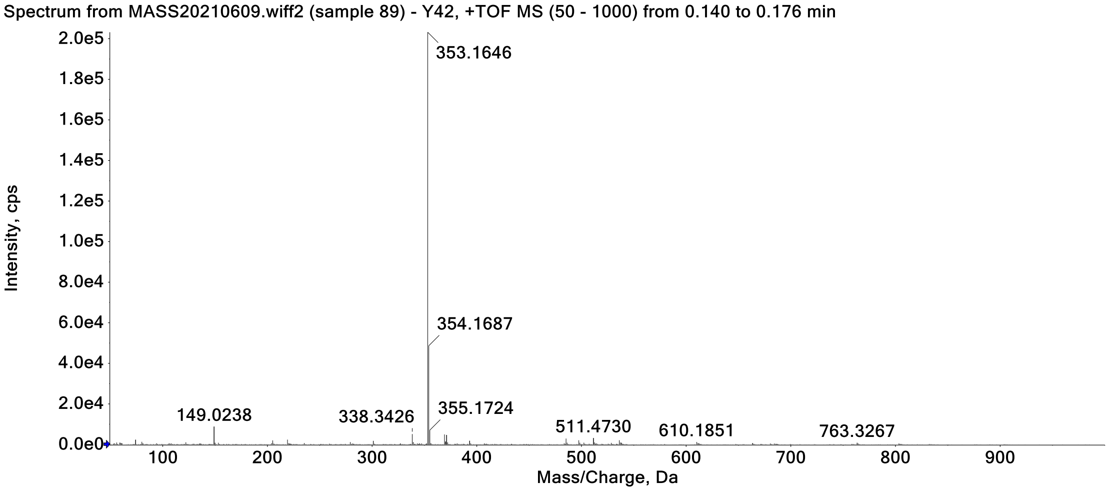

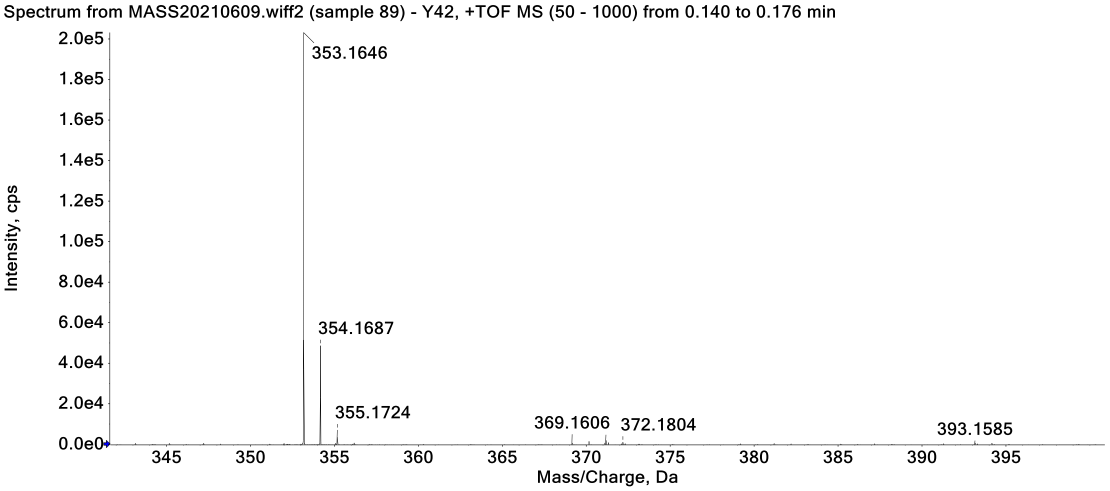

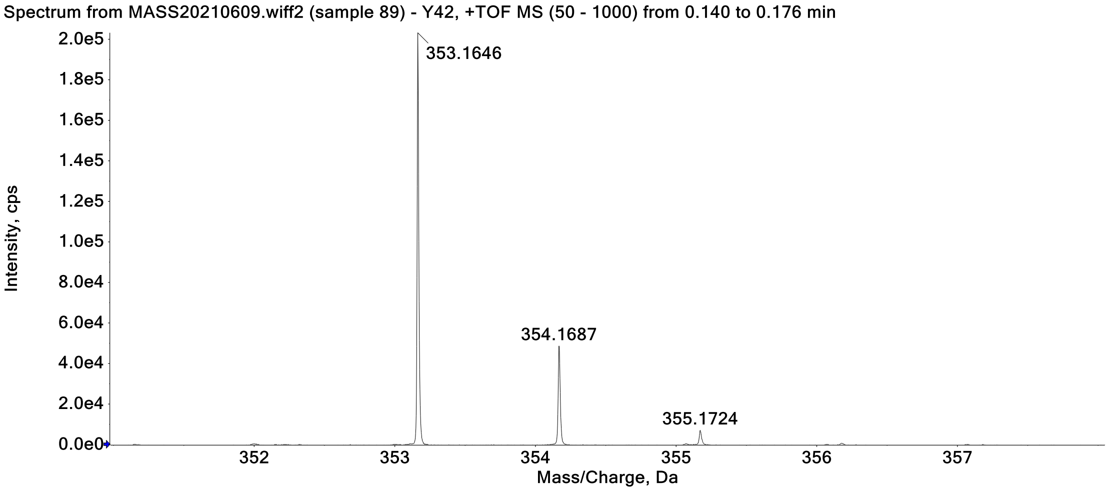

Hit	Formula	m/z	RDB	ppm	MS Rank	MSMS ppm	MSMS Rank	Found
1	C24H20N2O	353.1648	16.0	-0.7	1			NA/NA

HR-MS spectrum of 4f

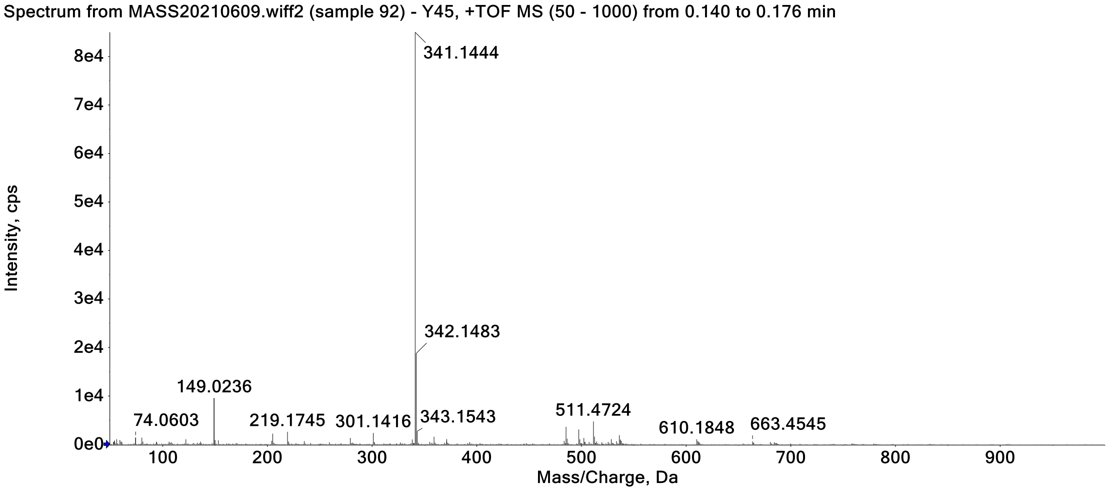

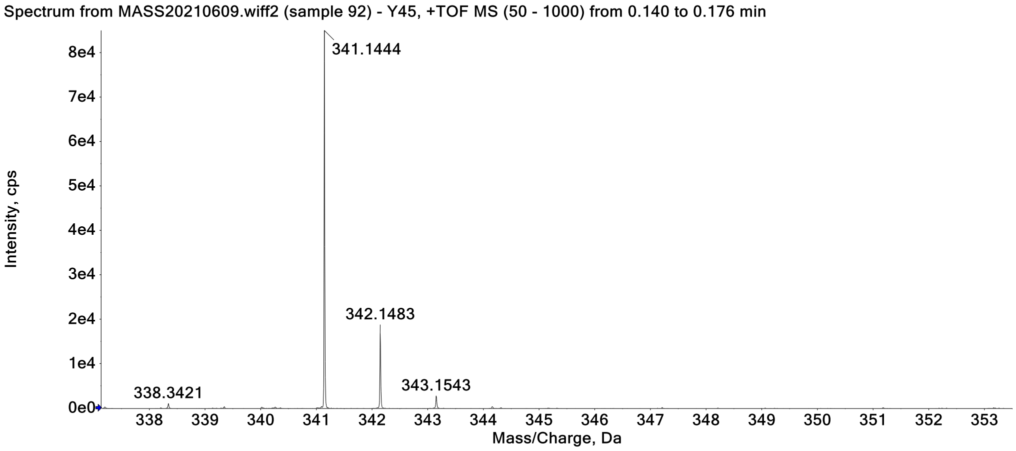

Hit	Formula	m/z	RDB	ppm	MS Rank	MSMS ppm	MSMS Rank	Found
1	C23H17FN2	341.1449	16.0	-1.3	1			NA/NA

HR-MS spectrum of 4g

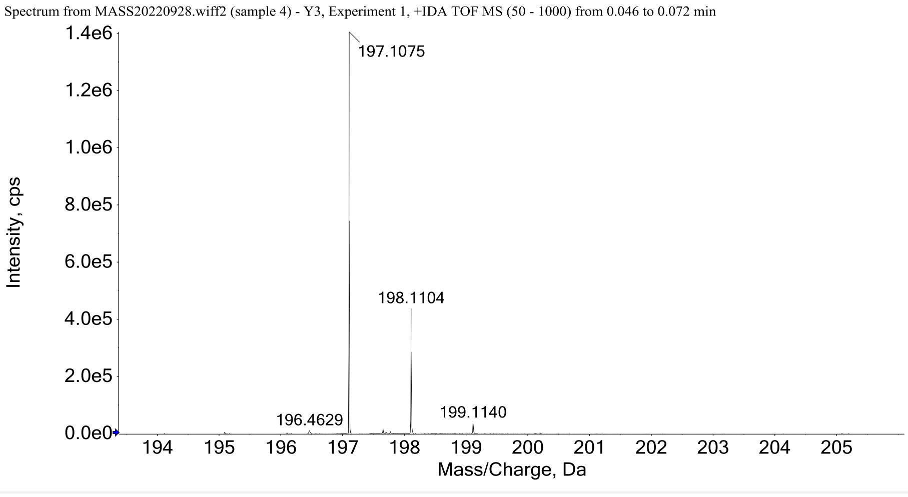

Hit	Formula	m/z	RDB	ppm	MS Rank	MSMS ppm	MSMS Rank	Found
1	C13H12N2	197.1073	9.0	0.9	1			NA/NA

HR-MS spectrum of 4h

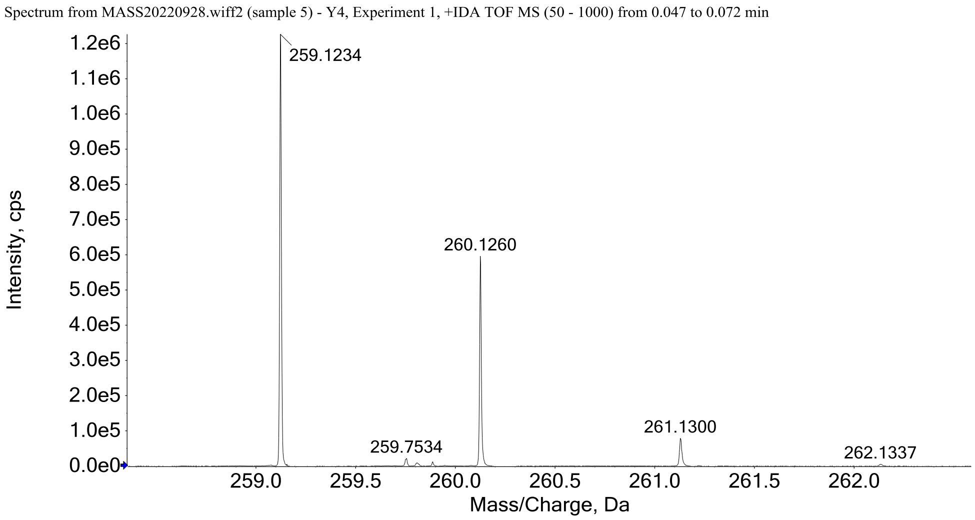

Hit	Formula	m/z	RDB	ppm	MS Rank	MSMS ppm	MSMS Rank	Found
1	C18H14N2	259.1230	13.0	1.6	1			NA/NA

HR-MS spectrum of 4i

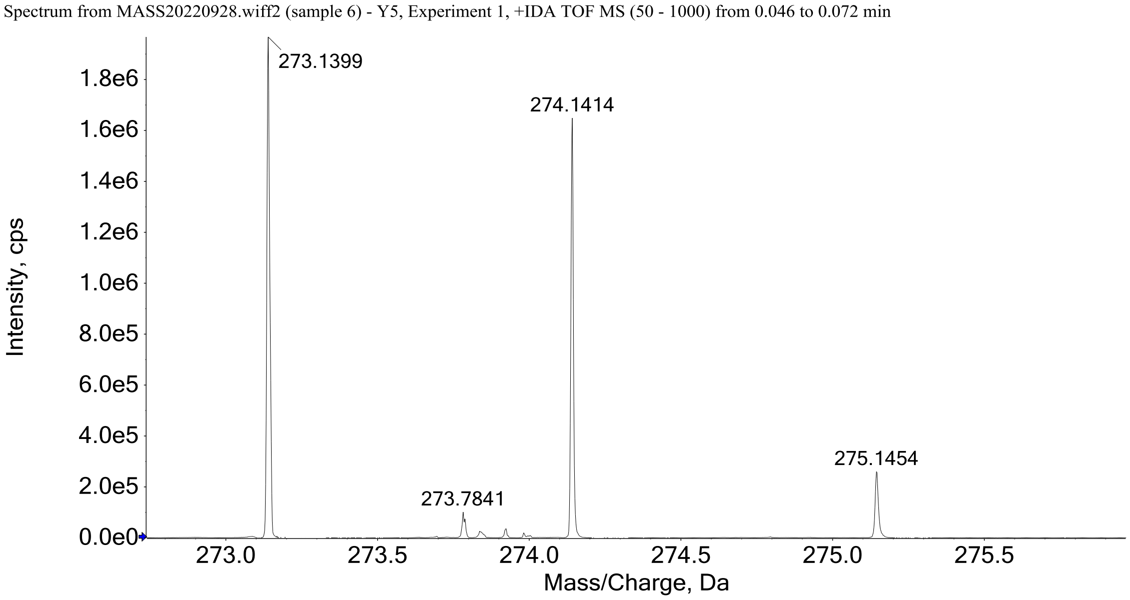

Hit	Formula	m/z	RDB	ppm	MS Rank	MSMS ppm	MSMS Rank	Found
1	C19H16N2	273.1386	13.0	4.7	1			NA/NA

HR-MS spectrum of 4j

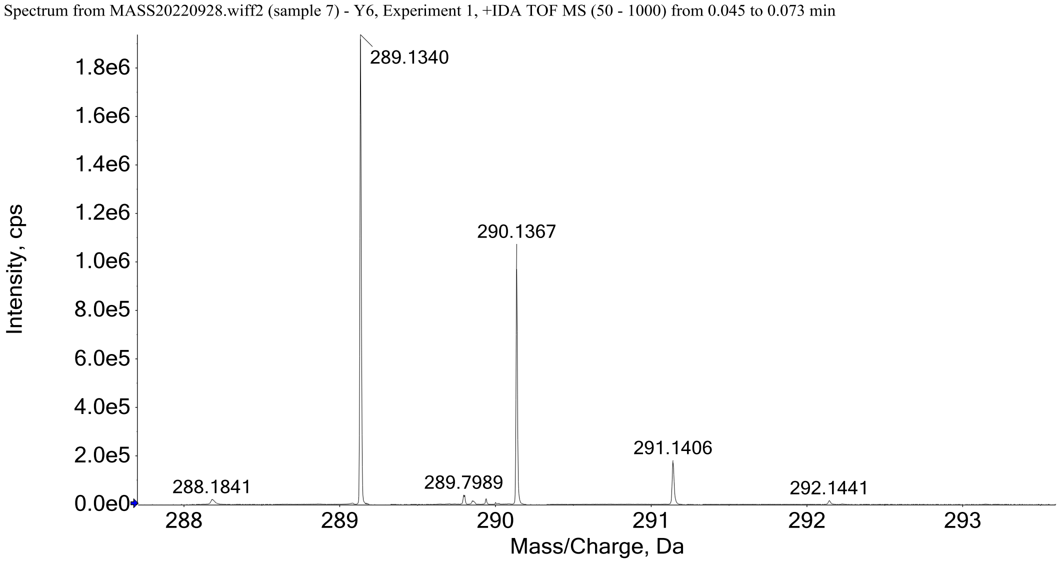

Hit	Formula	m/z	RDB	ppm	MS Rank	MSMS ppm	MSMS Rank	Found
1	C19H16N2O	289.1335	13.0	1.6	1			NA/NA

HR-MS spectrum of 4k

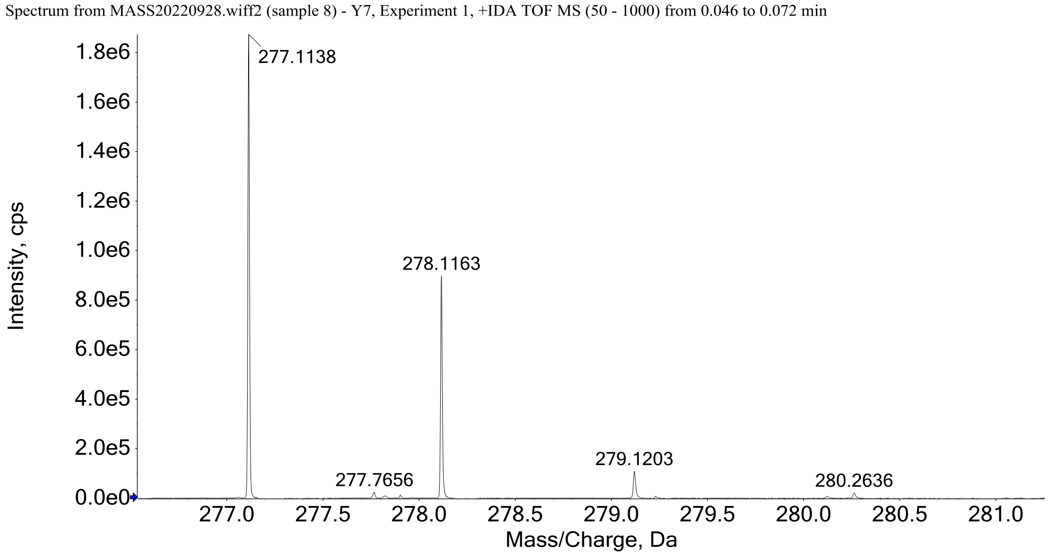

Hit	Formula	m/z	RDB	ppm	MS Rank	MSMS ppm	MSMS Rank	Found
1	C18H13FN2	277.1136	13.0	0.9	1			NA/NA

HR-MS spectrum of 4l

Spectrum from MASS202100707.wiff2 (sample 5) - Y5, +TOF MS (50 - 1000) from 0.158 to 0.193 min

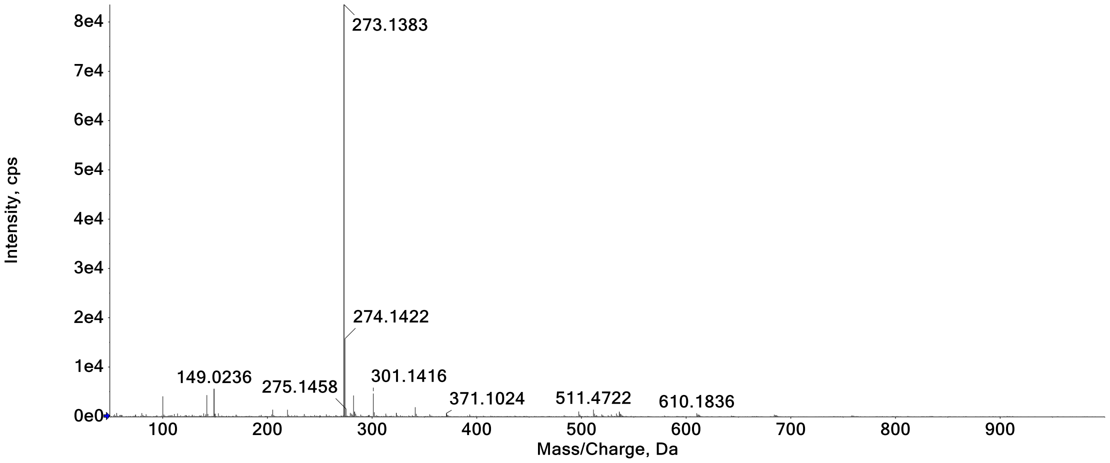

Spectrum from MASS202100707.wiff2 (sample 5) - Y5, +TOF MS (50 - 1000) from 0.158 to 0.193 min

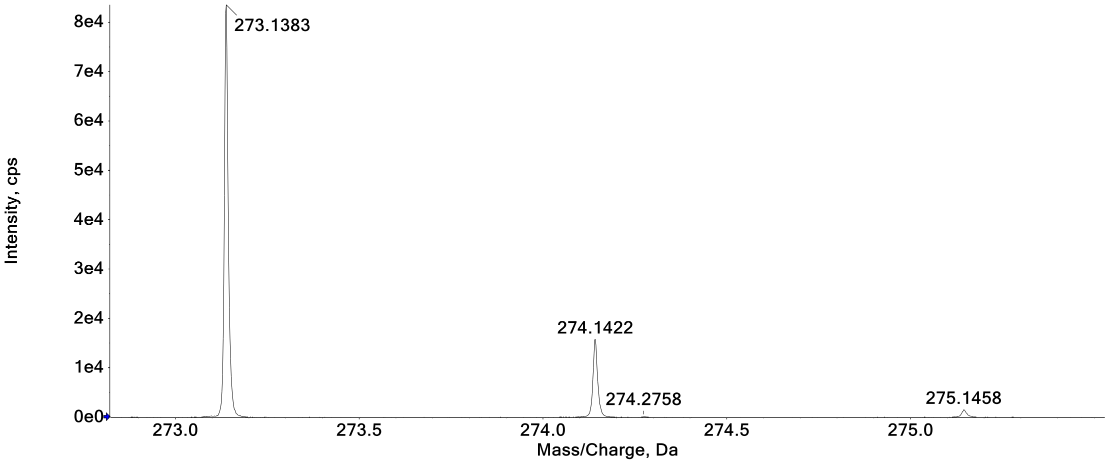

Hit	Formula	m/z	RDB	ppm	MS Rank	MSMS ppm	MSMS Rank	Found
1	C19H16N2	273.1386	13.0	-1.2	1			NA/NA

HR-MS spectrum of 4m

Spectrum from MASS20210609.wiff2 (sample 55) - Y8, +TOF MS (50 - 1000) from 0.132 to 0.167 min

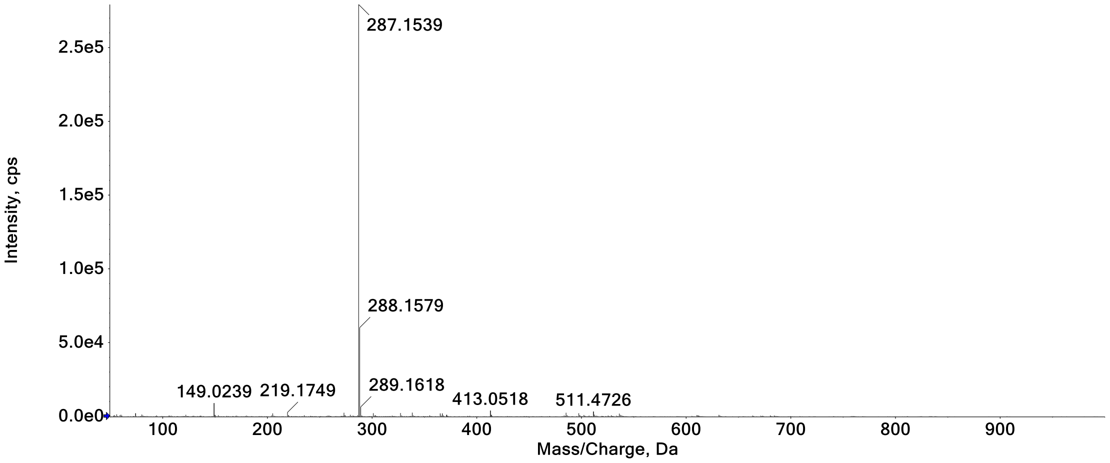

Spectrum from MASS20210609.wiff2 (sample 55) - Y8, +TOF MS (50 - 1000) from 0.132 to 0.167 min

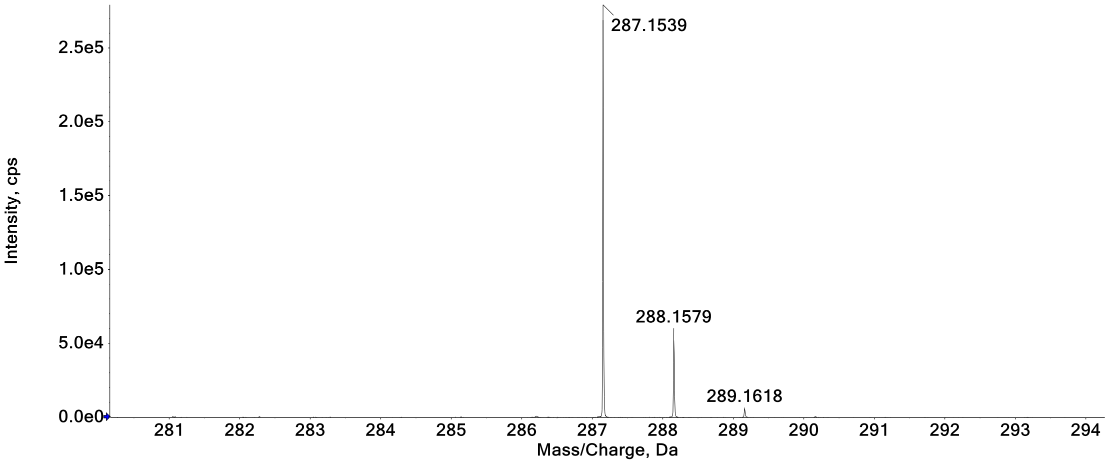

Spectrum from MASS20210609.wiff2 (sample 55) - Y8, +TOF MS (50 - 1000) from 0.132 to 0.167 min

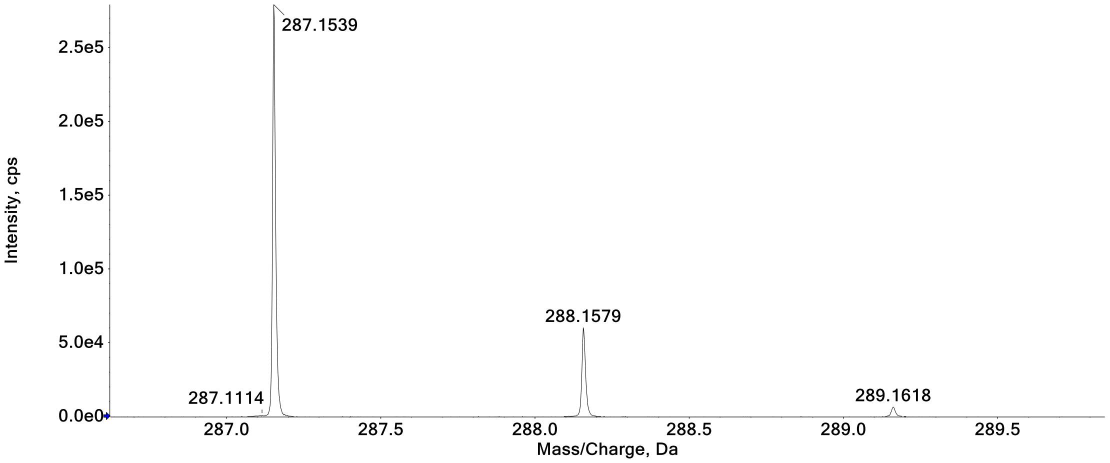

Hit	Formula	m/z	RDB	ppm	MS Rank	MSMS ppm	MSMS Rank	Found
1	C20H18N2	287.1543	13.0	-1.3	1			NA/NA

HR-MS spectrum of 5a

Spectrum from MASS20210609.wiff2 (sample 79) - Y32, +TOF MS (50 - 1000) from 0.079 to 0.114 min

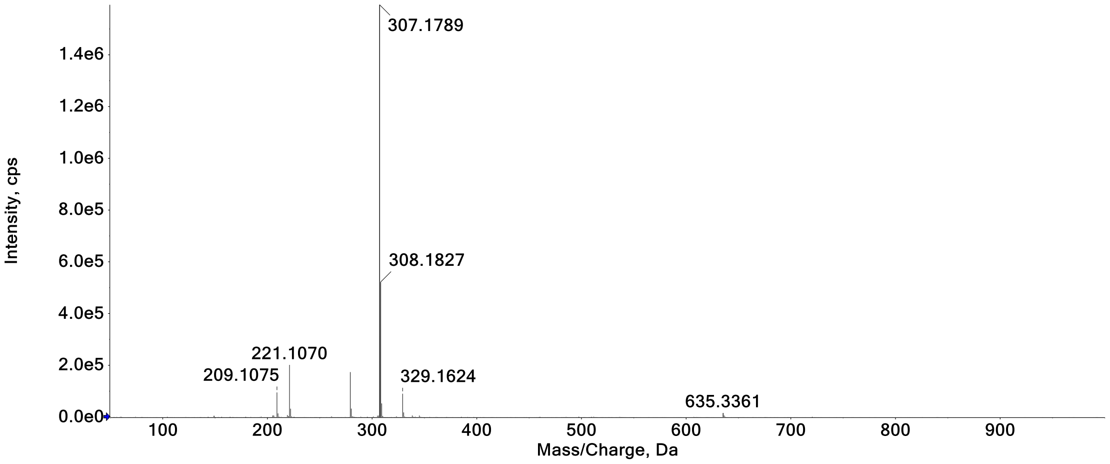

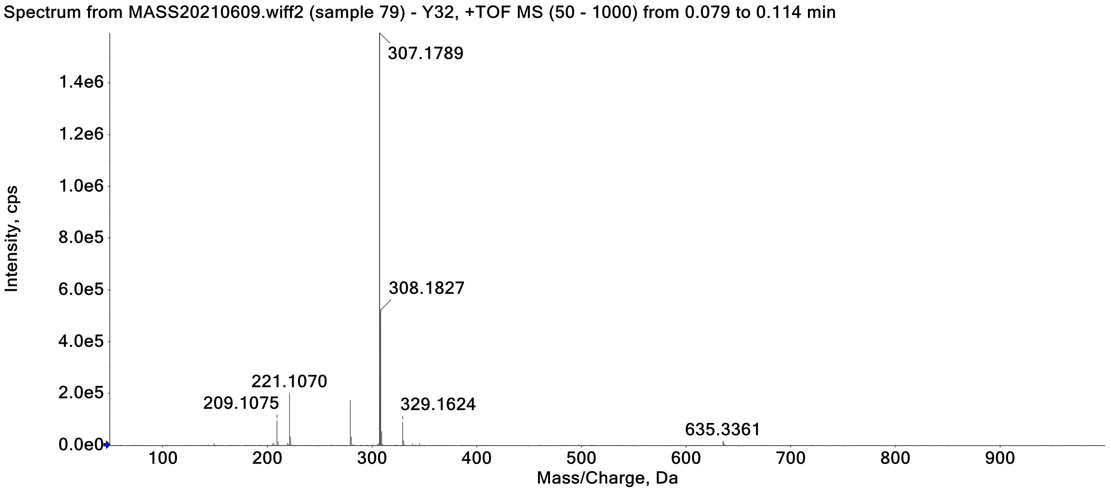

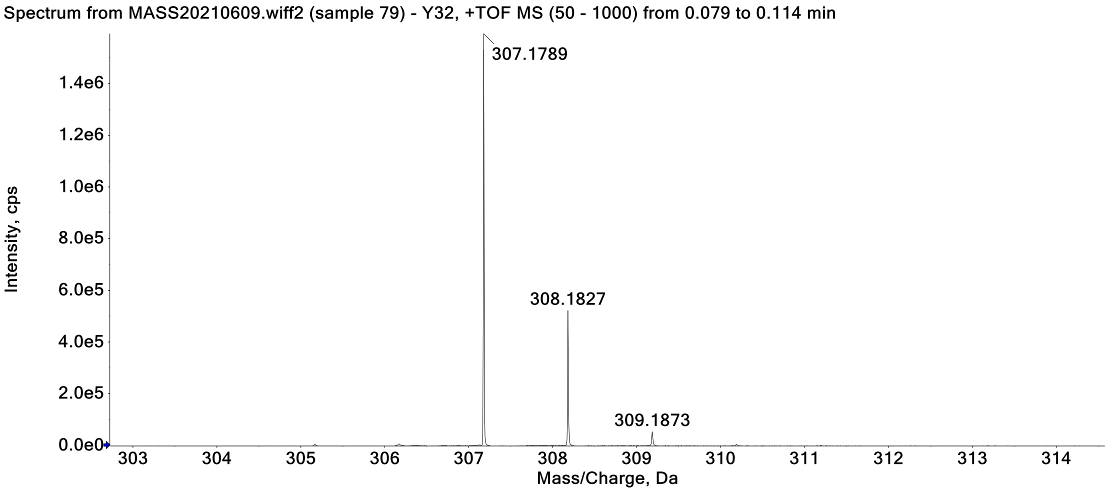

Hit	Formula	m/z	RDB	ppm	MS Rank	MSMS ppm	MSMS Rank	Found
1	C20H22N2O	307.1805	11.0	-0.9	1			NA/NA

HR-MS spectrum of 5b

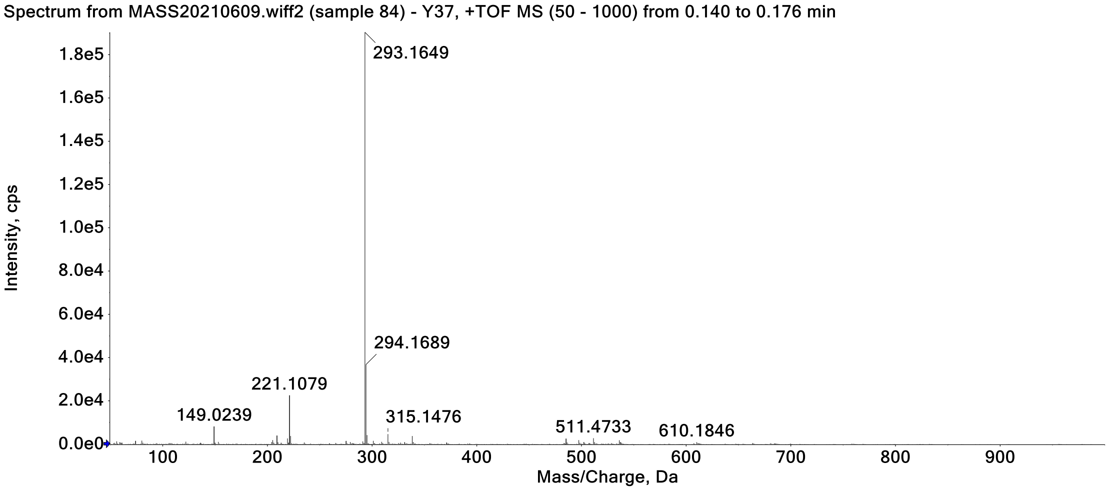

Spectrum from MASS20210609.wiff2 (sample 84) - Y37, +TOF MS (50 - 1000) from 0.140 to 0.176 min

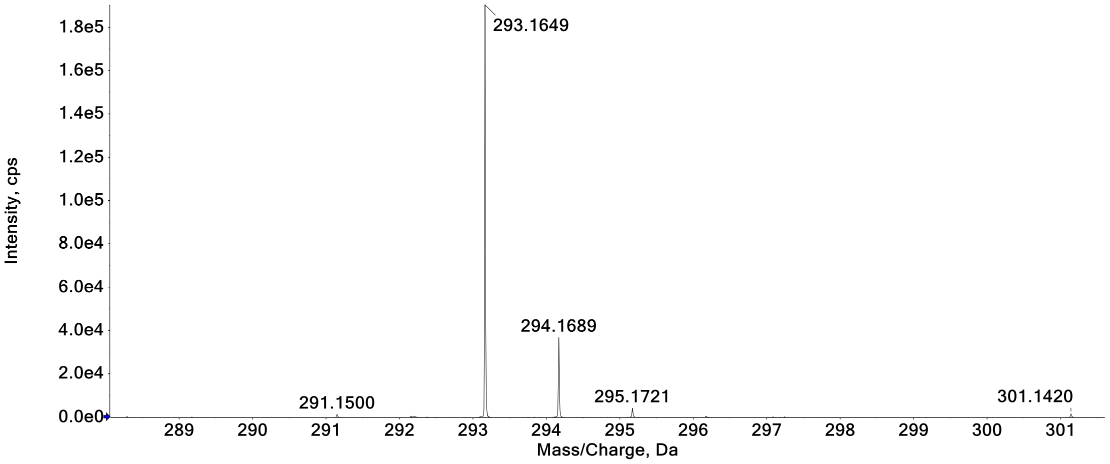

Hit	Formula	m/z	RDB	ppm	MS Rank	MSMS ppm	MSMS Rank	Found
1	C19H20N2O	293.1648	11.0	0.2	1			NA/NA

HR-MS spectrum of 5c

Spectrum from MASS20210609.wiff2 (sample 87) - Y40, +TOF MS (50 - 1000) from 0.140 to 0.176 min

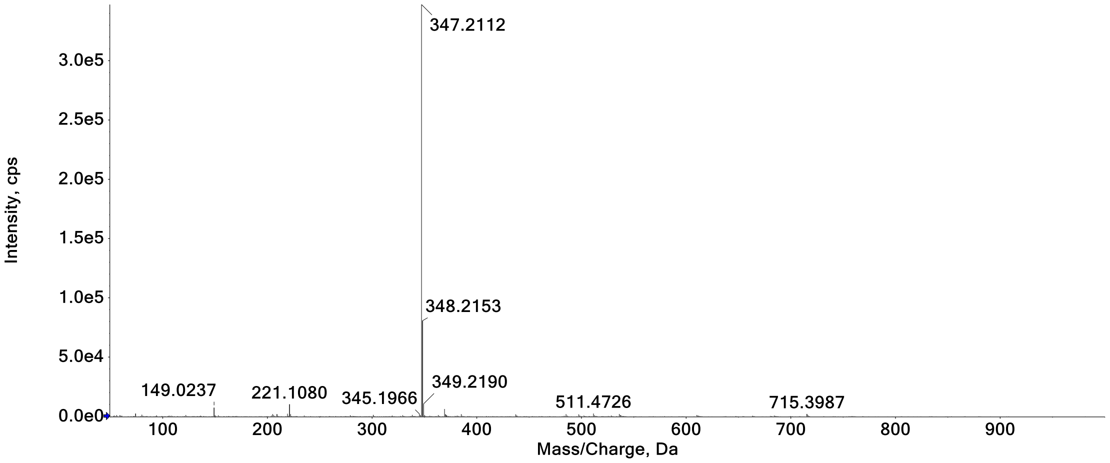

Spectrum from MASS20210609.wiff2 (sample 87) - Y40, +TOF MS (50 - 1000) from 0.140 to 0.176 min

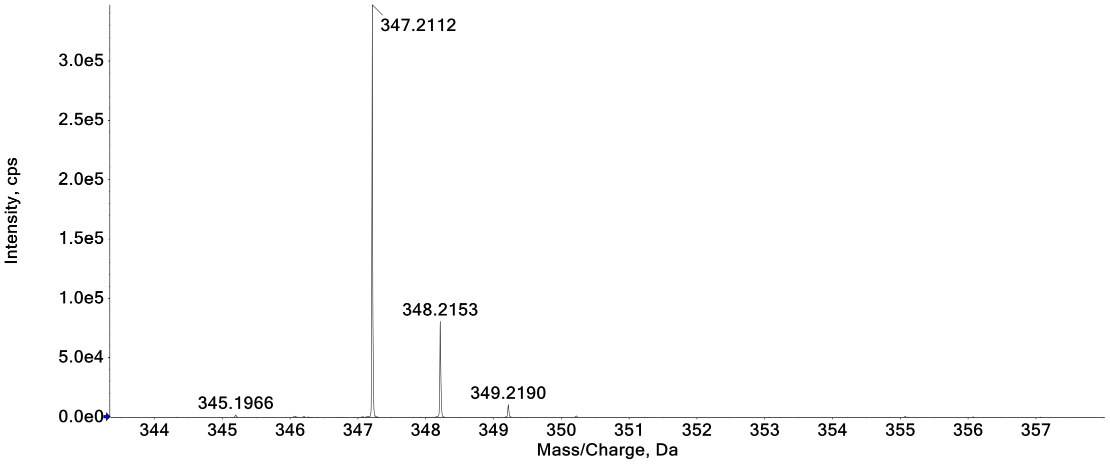

Hit	Formula	m/z	RDB	ppm	MS Rank	MSMS ppm	MSMS Rank	Found
1	C23H26N2O	347.2118	12.0	-1.7	1			NA/NA

HR-MS spectrum of 5d

Spectrum from MASS20210609.wiff2 (sample 81) - Y34, +TOF MS (50 - 1000) from 0.140 to 0.176 min

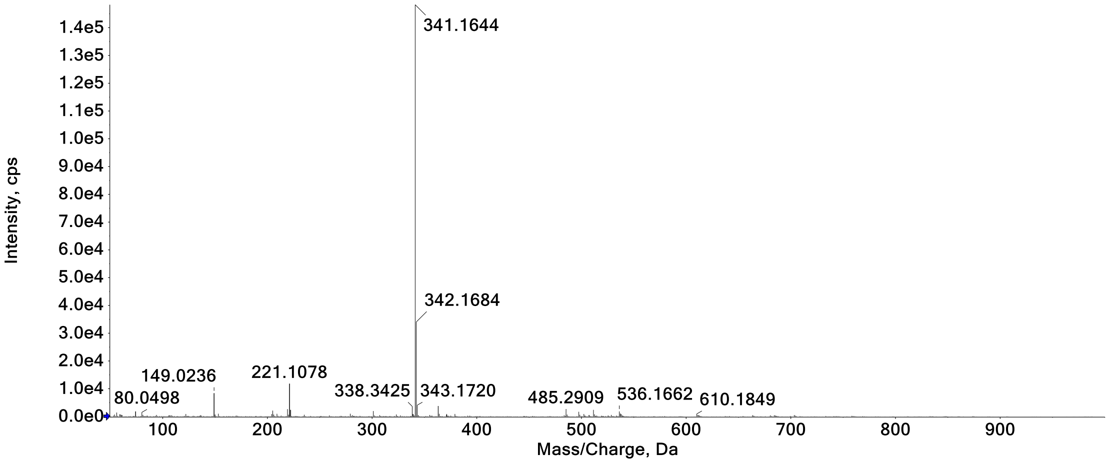

Spectrum from MASS20210609.wiff2 (sample 81) - Y34, +TOF MS (50 - 1000) from 0.140 to 0.176 min

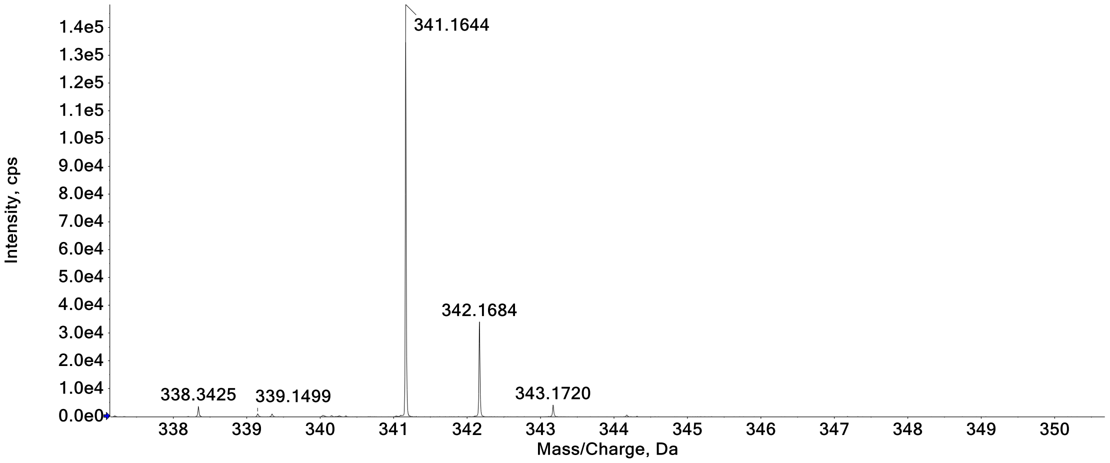

Hit	Formula	m/z	RDB	ppm	MS Rank	MSMS ppm	MSMS Rank	Found
1	C23H20N2O	341.1648	15.0	-1.3	1			NA/NA

HR-MS spectrum of 5e

Spectrum from MASS20210609.wiff2 (sample 90) - Y43, +TOF MS (50 - 1000) from 0.140 to 0.176 min

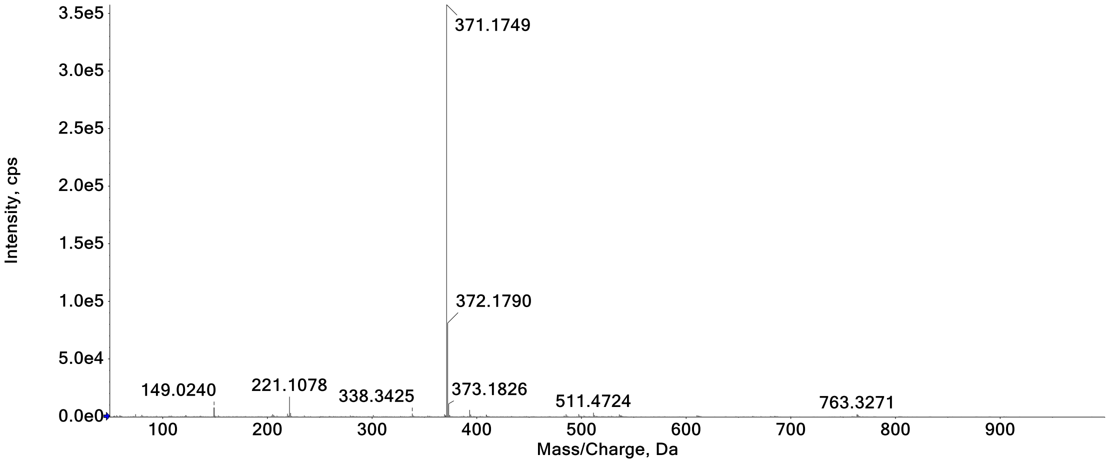

Spectrum from MASS20210609.wiff2 (sample 90) - Y43, +TOF MS (50 - 1000) from 0.140 to 0.176 min

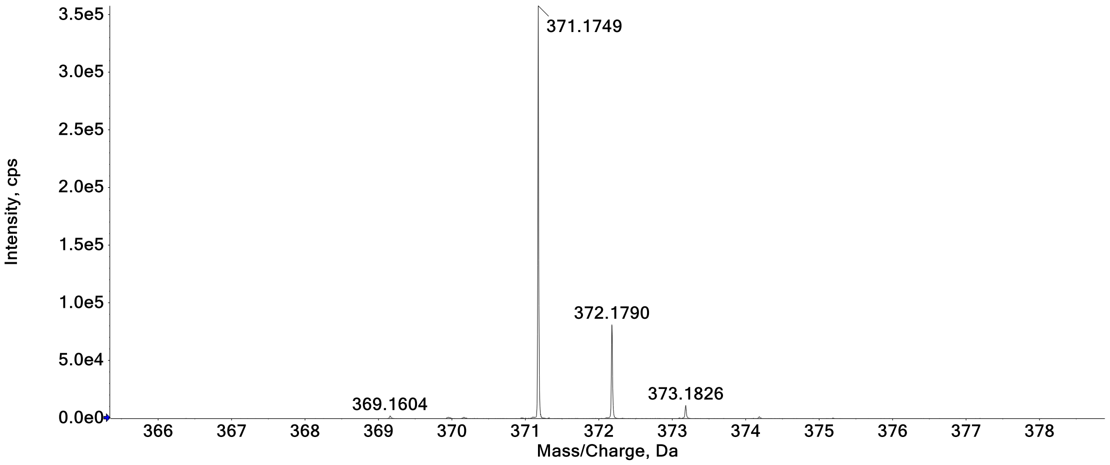

Hit	Formula	m/z	RDB	ppm	MS Rank	MSMS ppm	MSMS Rank	Found
1	C24H22N2O2	371.1754	15.0	-1.4	1			NA/NA

HR-MS spectrum of 5f

Spectrum from MASS20210609.wiff2 (sample 93) - Y46, +TOF MS (50 - 1000) from 0.140 to 0.176 min

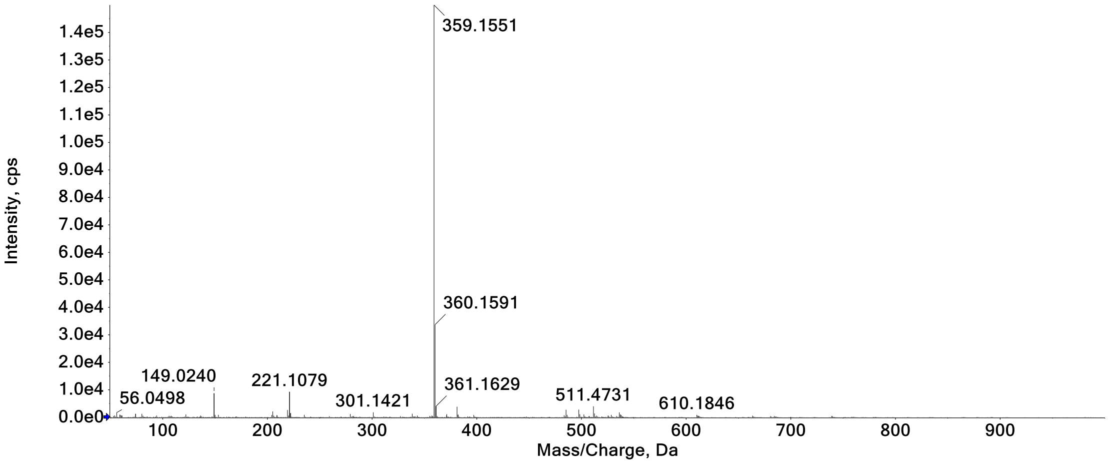

Spectrum from MASS20210609.wiff2 (sample 93) - Y46, +TOF MS (50 - 1000) from 0.140 to 0.176 min

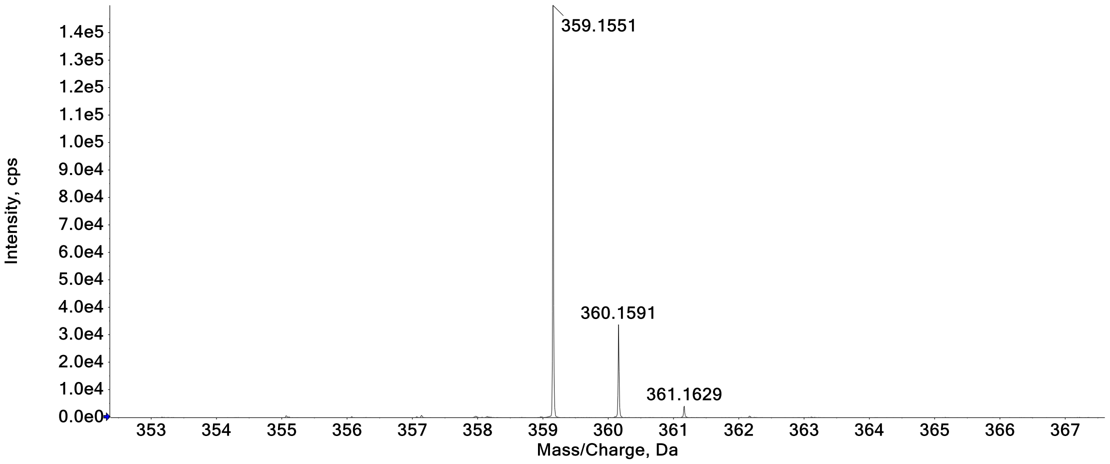

Hit	Formula	m/z	RDB	ppm	MS Rank	MSMS ppm	MSMS Rank	Found
1	C23H19FN2O	359.1554	15.0	-0.9	1			NA/NA

HR-MS spectrum of 5i

Spectrum from MASS20220928.wiff2 (sample 10) - Y9, Experiment 1, +IDA TOF MS (50 - 1000) from 0.046 to 0.071 min

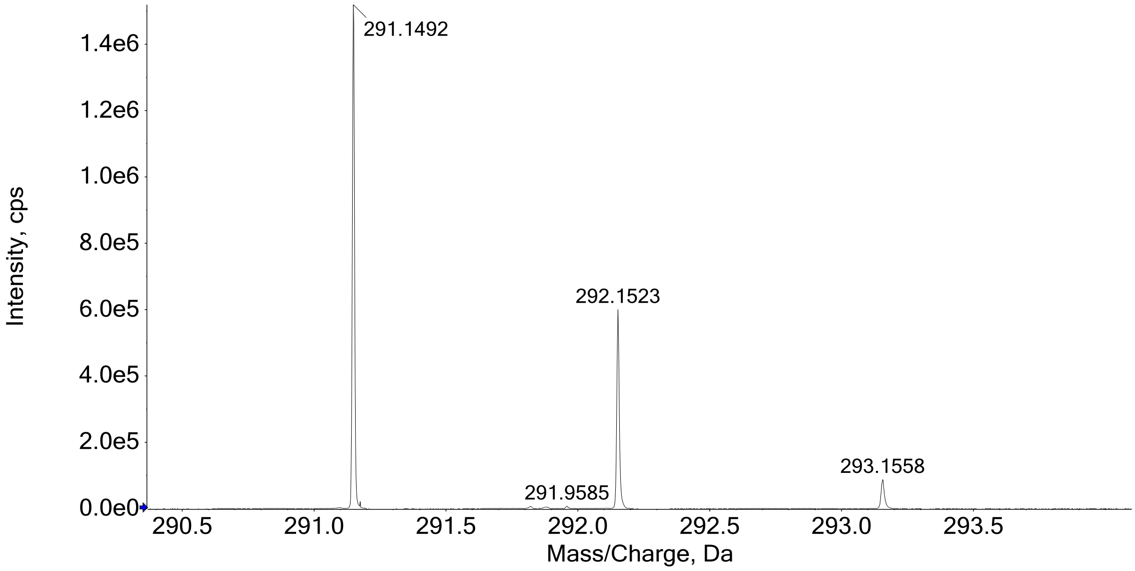

Hit	Formula	m/z	RDB	ppm	MS Rank	MSMS ppm	MSMS Rank	Found
1	C19H18N2O	291.1492	12.0	0.0	1			NA/NA

HR-MS spectrum of 5j

Spectrum from MASS20220928.wiff2 (sample 11) - Y10, Experiment 1, +IDA TOF MS (50 - 1000) from 0.046 to 0.069 min

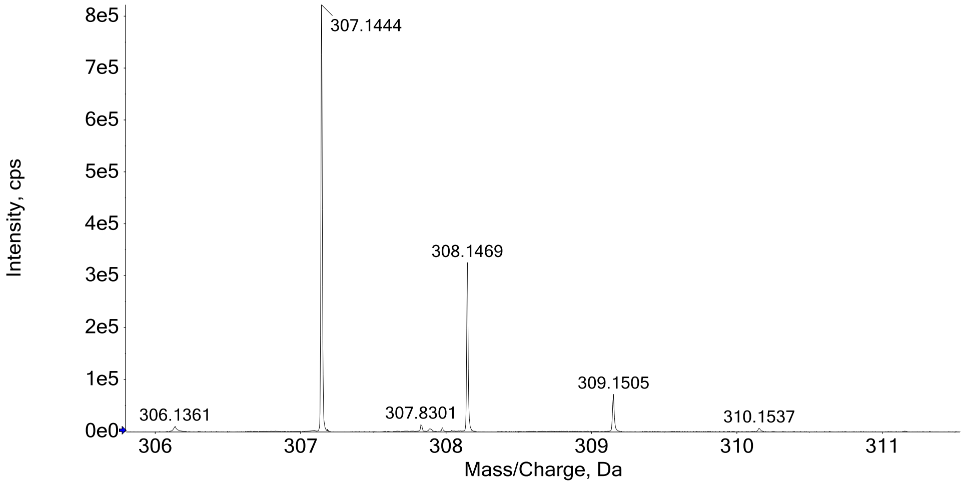

Hit	Formula	m/z	RDB	ppm	MS Rank	MSMS ppm	MSMS Rank	Found
1	C19H18N2O2	307.1441	12.0	1.0	1			NA/NA

HR-MS spectrum of 5k

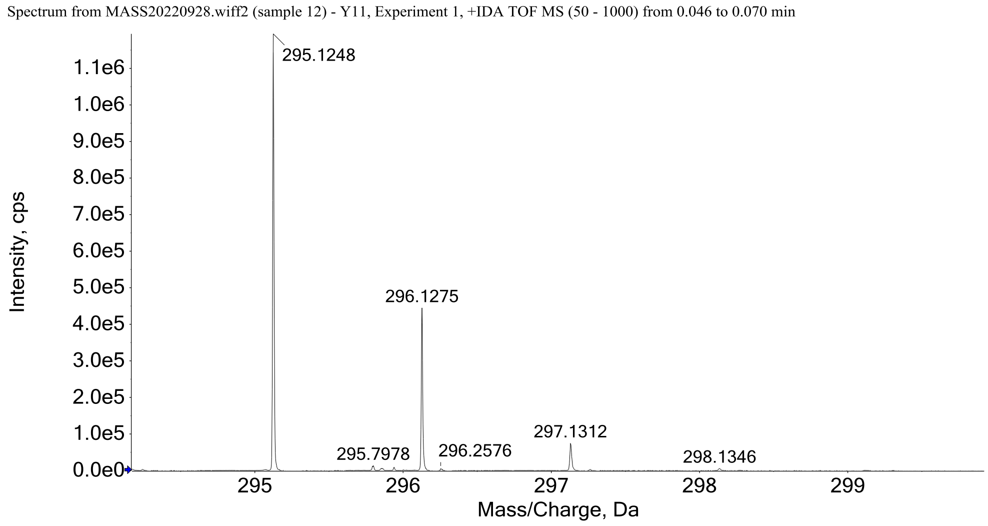

Hit	Formula	m/z	RDB	ppm	MS Rank	MSMS ppm	MSMS Rank	Found
1	C18H15FN2O	295.1241	12.0	2.3	1			NA/NA

HR-MS spectrum of 5l

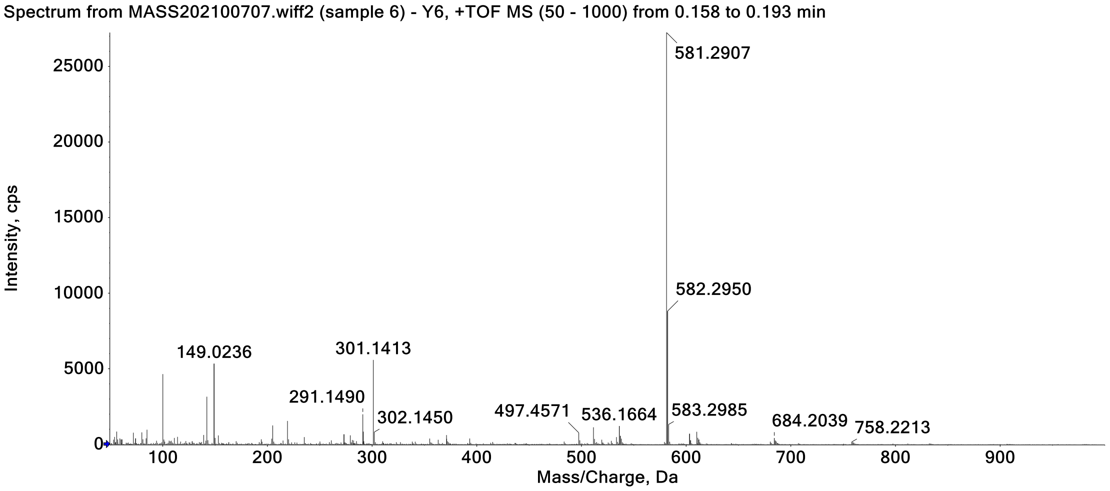

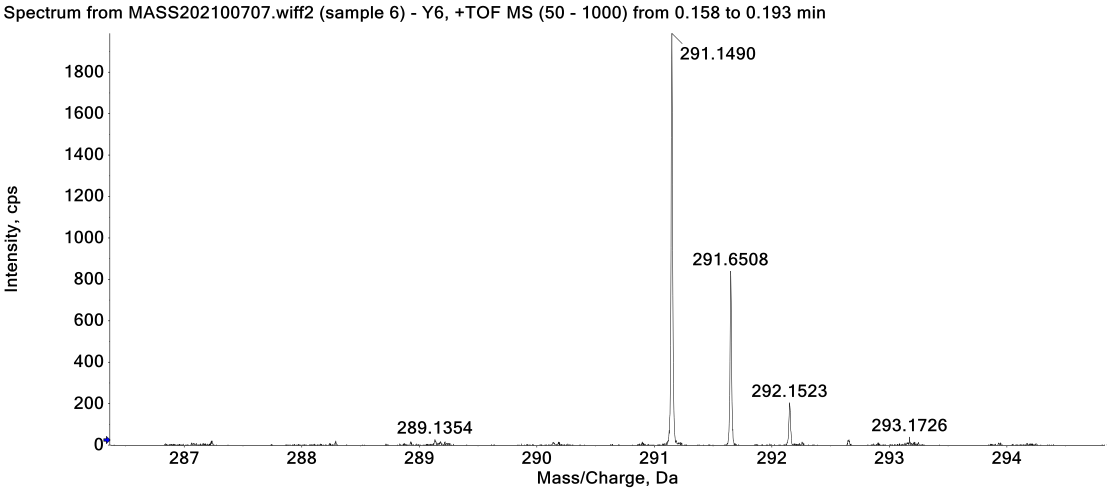

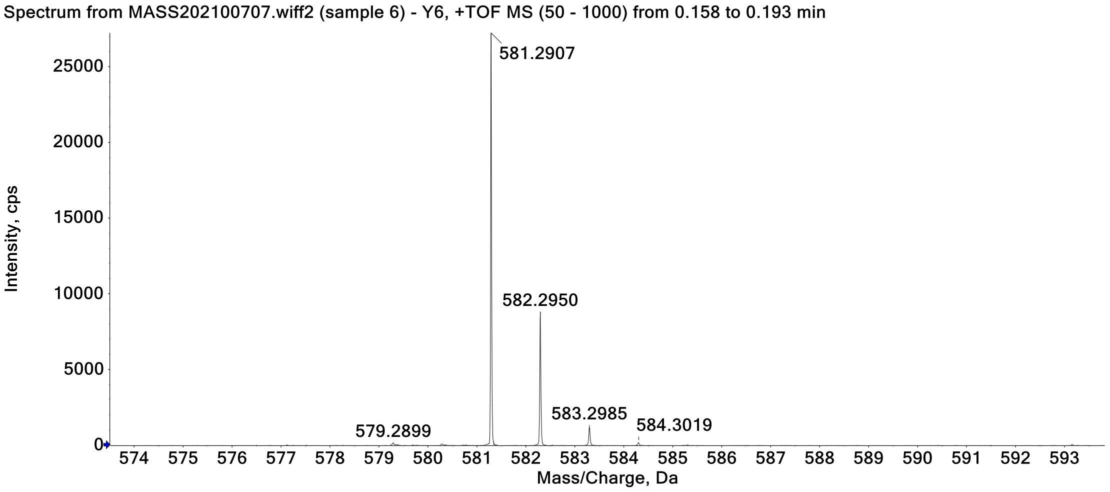

Hit	Formula	m/z	RDB	ppm	MS Rank	MSMS ppm	MSMS Rank	Found
1	C38H36N4O2	581. 2911	23. 0	-0. 7	1			NA/NA

HR-MS spectrum of 5m

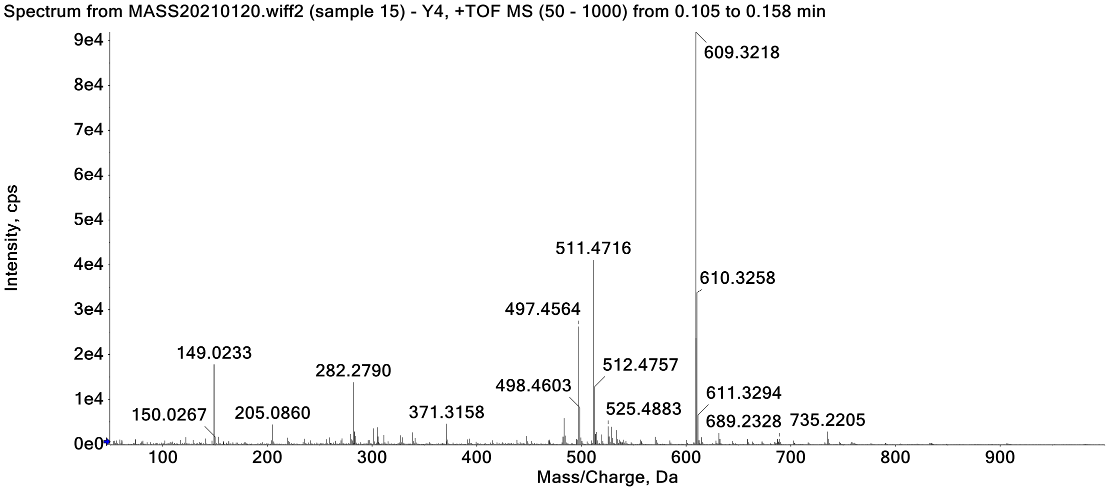

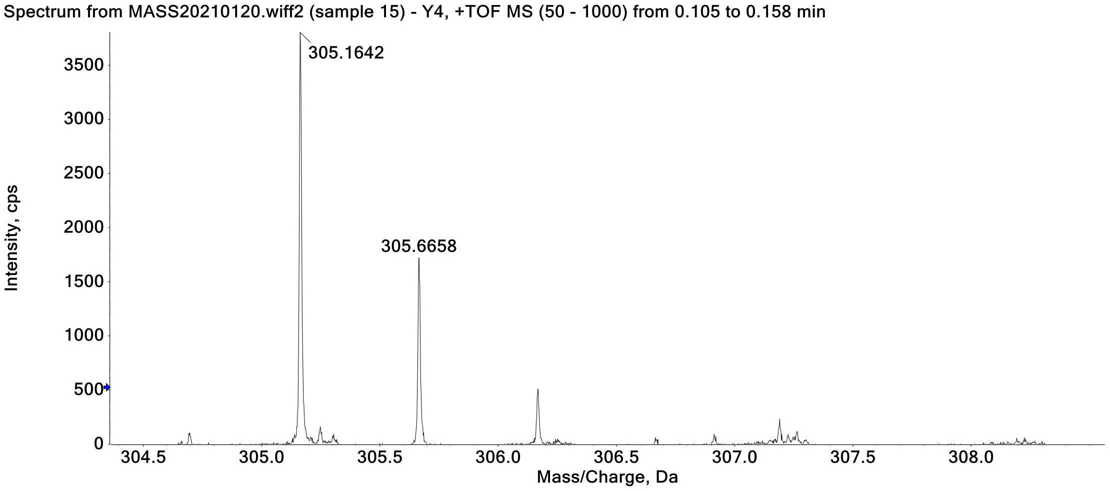

Hit	Formula	m/z	RDB	ppm	MS Rank	MSMS ppm	MSMS Rank	Found
1	C20H20N2O	305. 1648	12. 0	-2. 1	1			NA/NA

HR-MS spectrum of 5n

Spectrum from MASS20211013.wiff2 (sample 54) - Y30, +TOF MS (50 - 1000) from 0.149 to 0.220 min

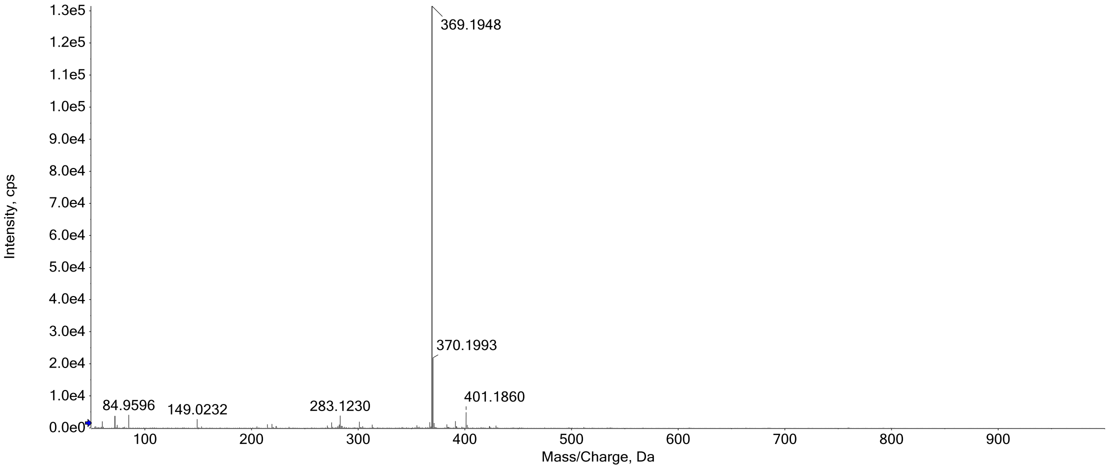

Spectrum from MASS20211013.wiff2 (sample 54) - Y30, +TOF MS (50 - 1000) from 0.149 to 0.220 min

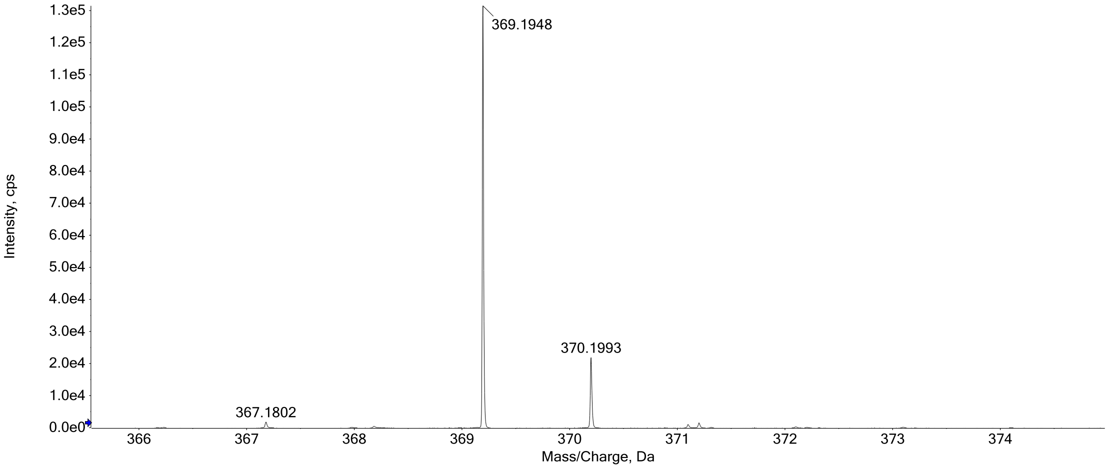

Hit	Formula	m/z	RDB	ppm	MS Rank	MSMS ppm	MSMS Rank	Found
1	C25H24N2O	369.1961	15.0	-2.3	1			NA/NA

HR-MS spectrum of 6a

Spectrum from MASS20211013.wiff2 (sample 36) - Y12, +TOF MS (50 - 1000) from 0.123 to 0.193 min

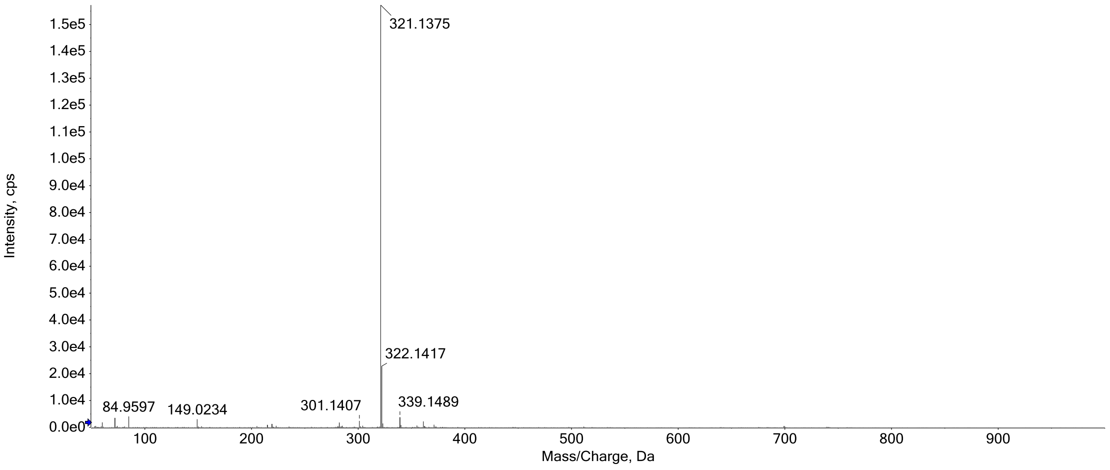

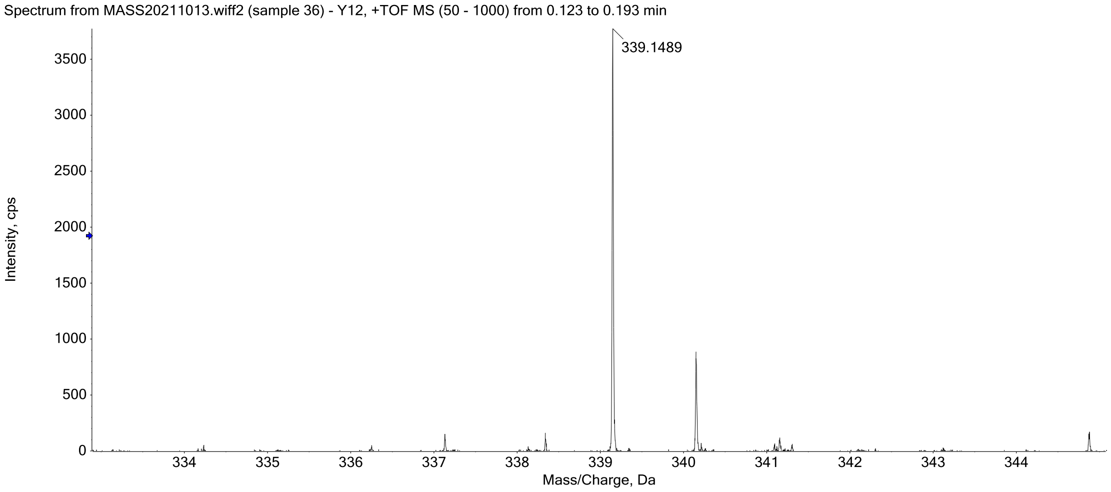

Hit	Formula	m/z	RDB	ppm	MS Rank	MSMS ppm	MSMS Rank	Found
1	C23H18N2O	339.1492	16.0	-0.9	1			NA/NA

HR-MS spectrum of 6b

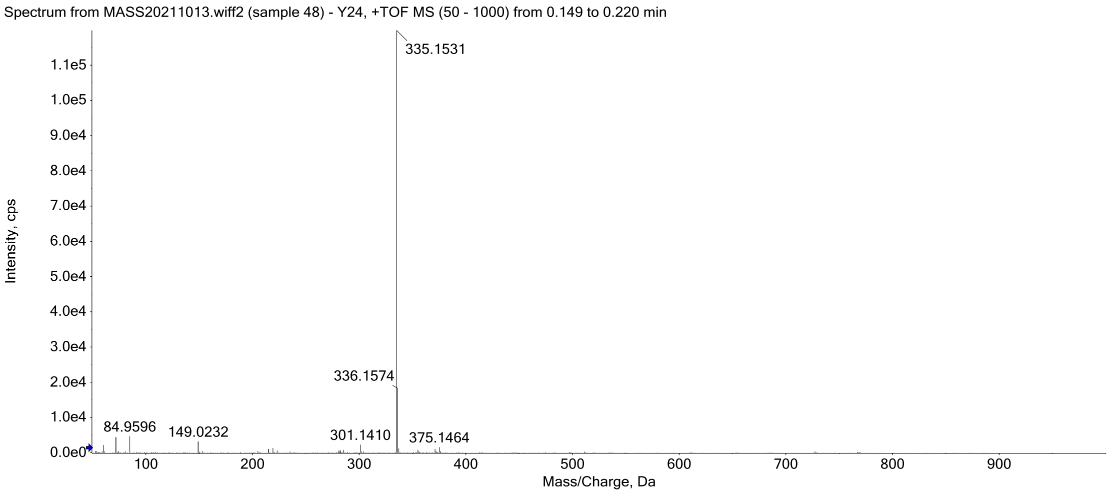

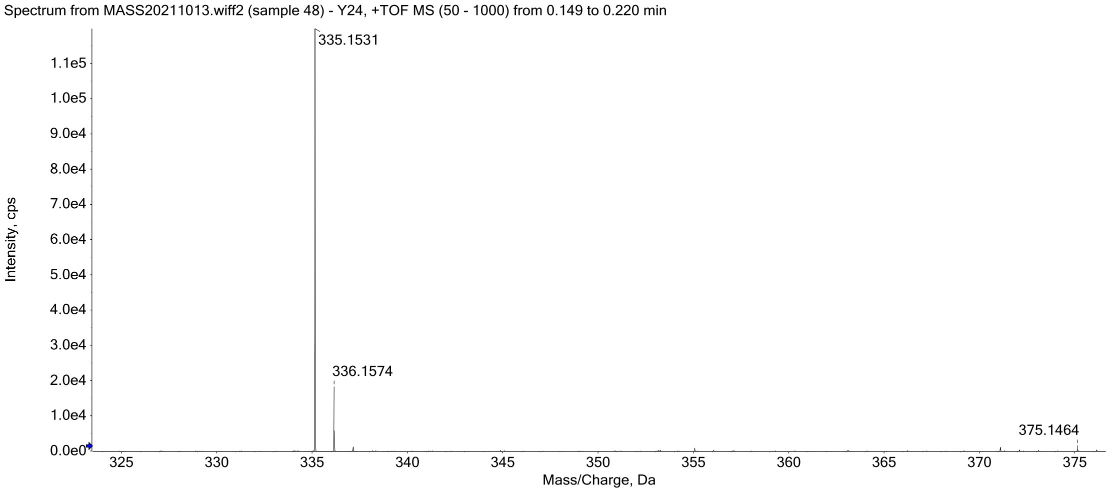

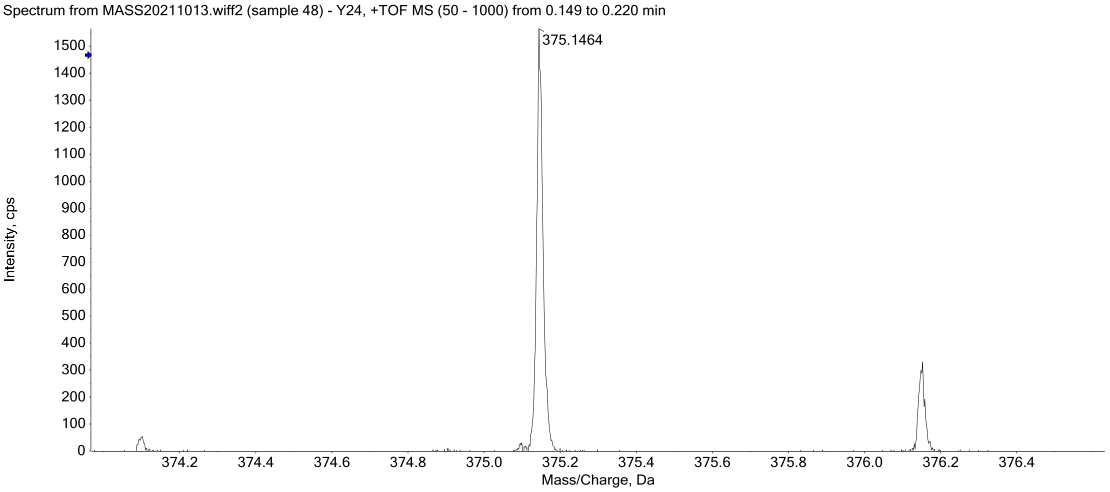

Hit	Formula	m/z	RDB	ppm	MS Rank	MSMS ppm	MSMS Rank	Found
1	C24H20N2O	375.1468	16.0	-1.0	1			NA/NA

HR-MS spectrum of 6c

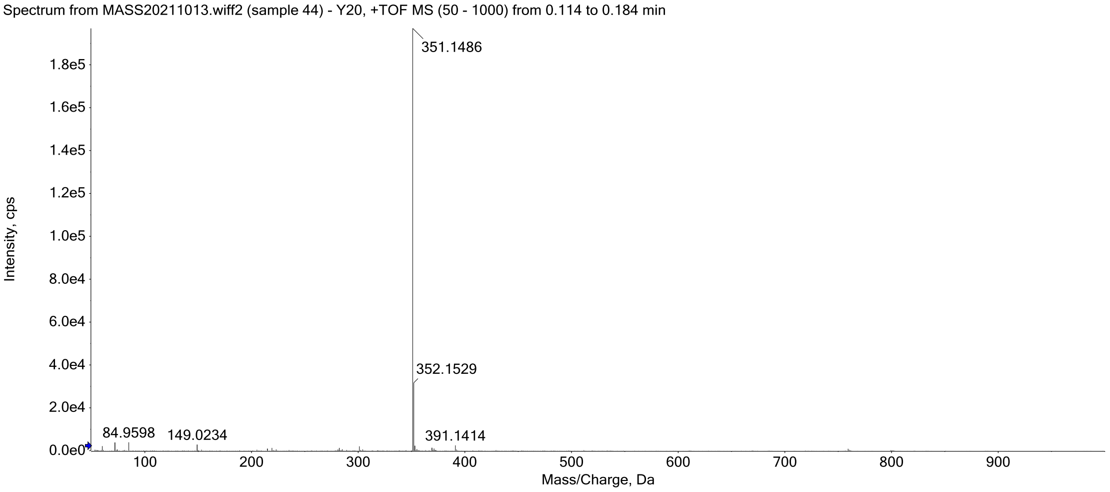

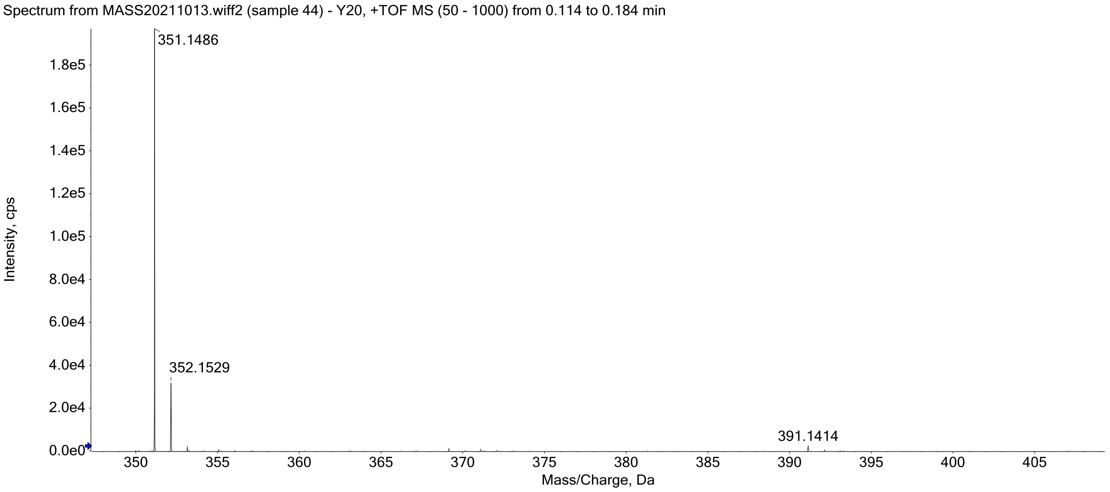

Hit	Formula	m/z	RDB	ppm	MS Rank	MSMS ppm	MSMS Rank	Found
1	C24H18N2O	351.1492	17.0	-1.7	1			NA/NA

HR-MS spectrum of 6d

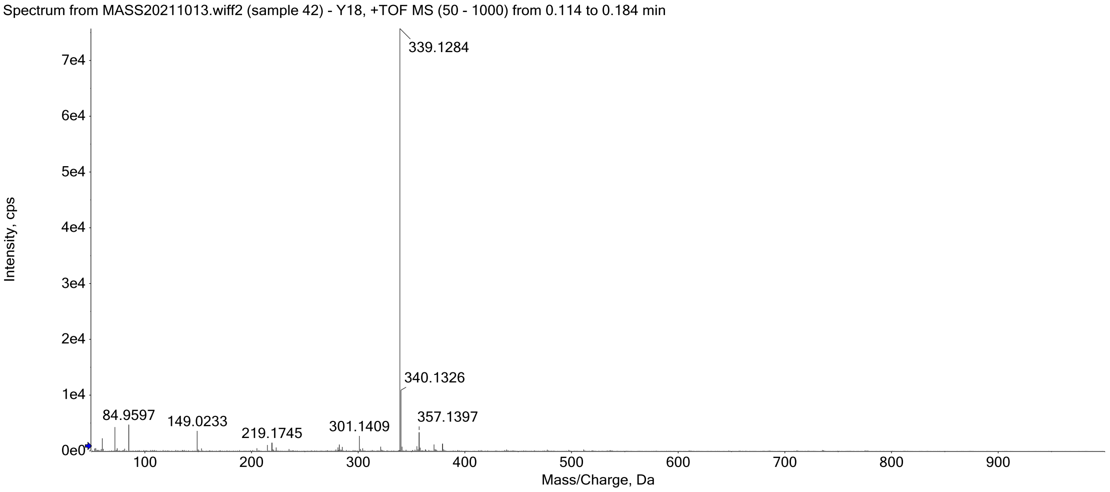

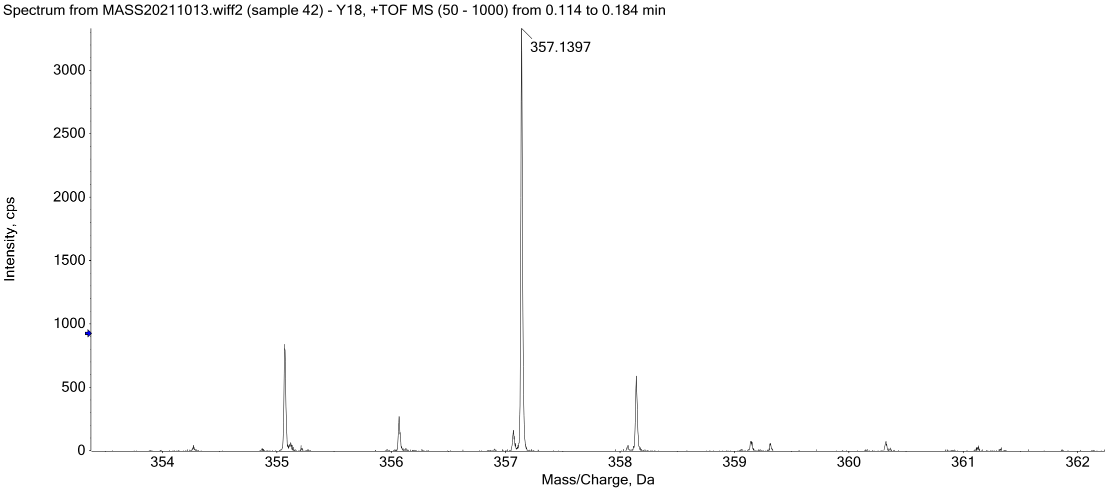

Hit	Formula	m/z	RDB	ppm	MS Rank	MSMS ppm	MSMS Rank	Found
1	C23H17FN2O	357.1398	16.0	-0.2	1			NA/NA

HR-MS spectrum of 6e

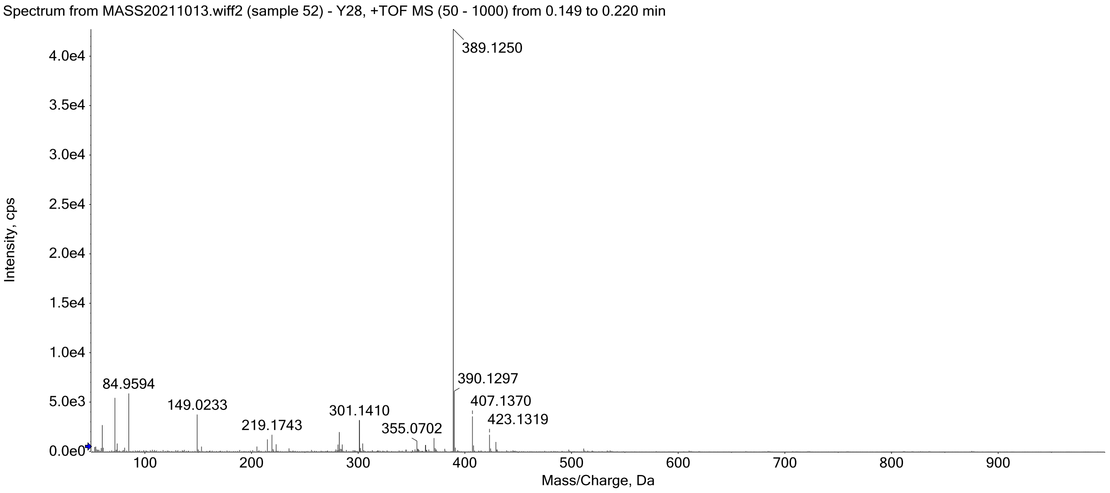

Spectrum from MASS20211013.wiff2 (sample 52) - Y28, +TOF MS (50 - 1000) from 0.149 to 0.220 min

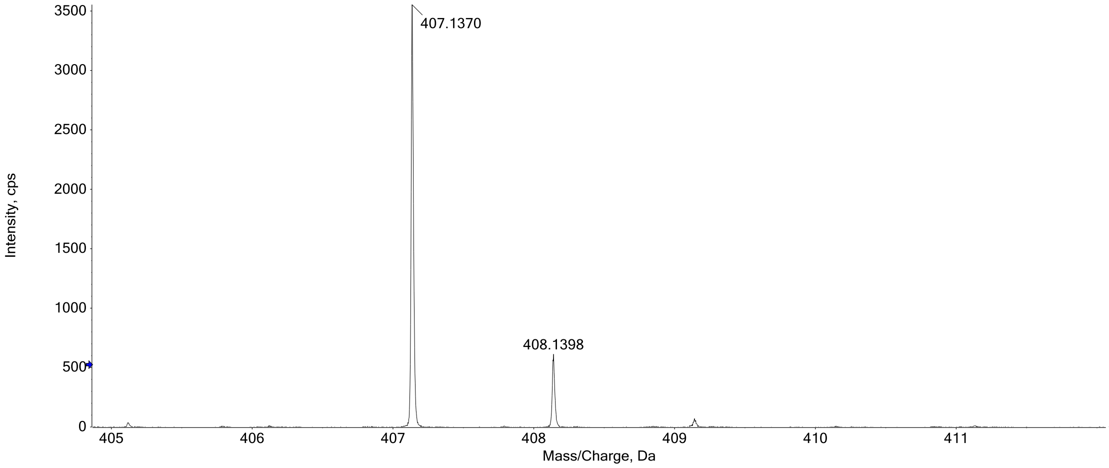

Hit	Formula	m/z	RDB	ppm	MS Rank	MSMS ppm	MSMS Rank	Found
1	C24H17F3N2O	407.1366	16.0	1.0	1			NA/NA

HR-MS spectrum of 6f

Spectrum from MASS20211013.wiff2 (sample 46) - Y22, +TOF MS (50 - 1000) from 0.123 to 0.193 min

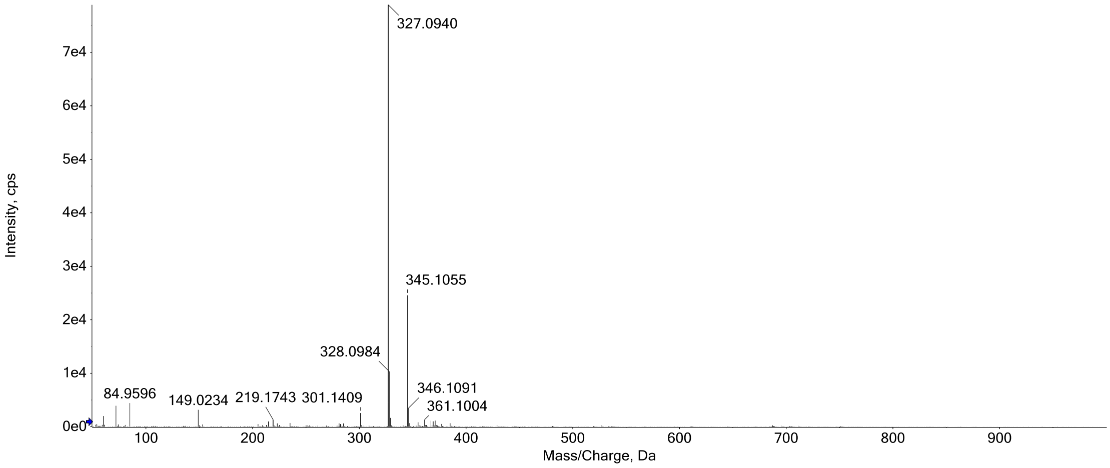

Spectrum from MASS20211013.wiff2 (sample 46) - Y22, +TOF MS (50 - 1000) from 0.123 to 0.193 min

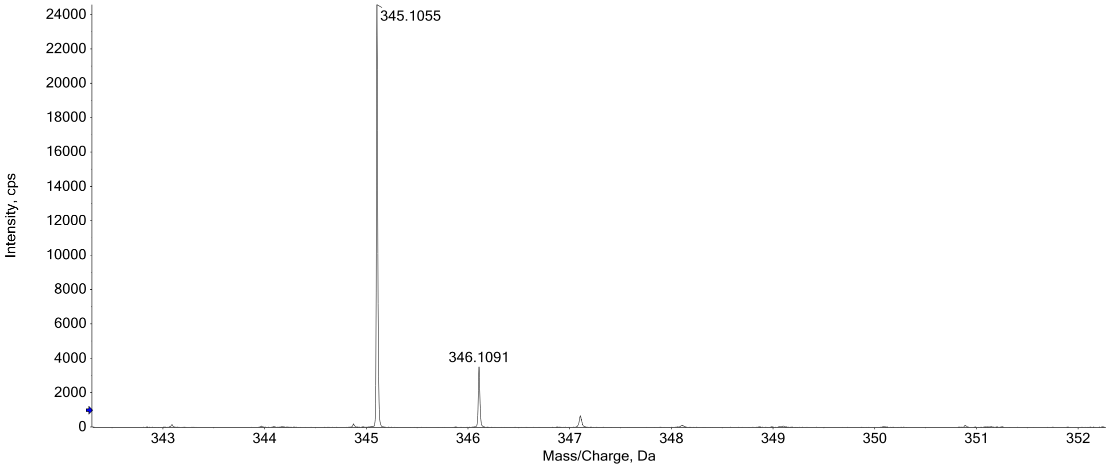

Hit	Formula	m/z	RDB	ppm	MS Rank	MSMS ppm	MSMS Rank	Found
1	C21H16N2OS	345.1056	15.0	-0.3	1			NA/NA

HR-MS spectrum of 6g

Spectrum from MASS20210609.wiff2 (sample 65) - Y18, +TOF MS (50 - 1000) from 0.079 to 0.114 min

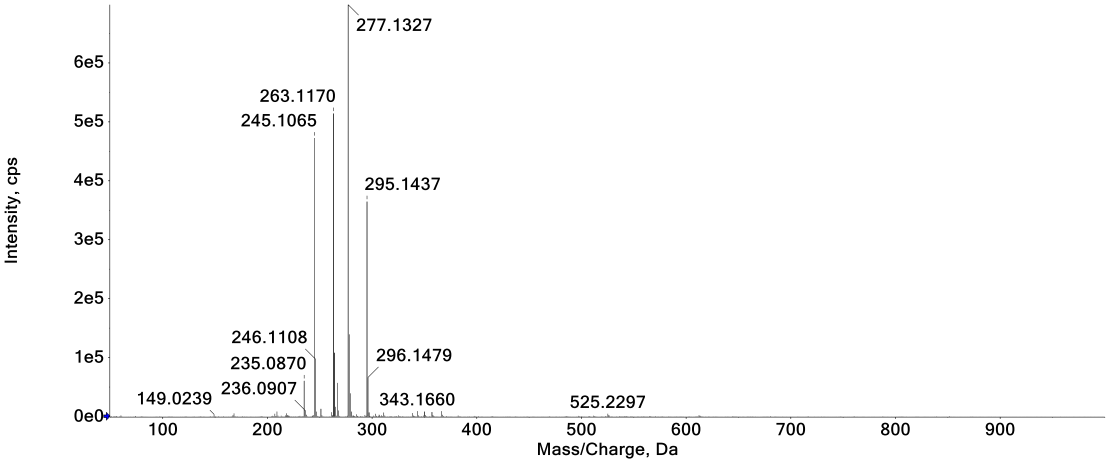

Spectrum from MASS20210609.wiff2 (sample 65) - Y18, +TOF MS (50 - 1000) from 0.079 to 0.114 min

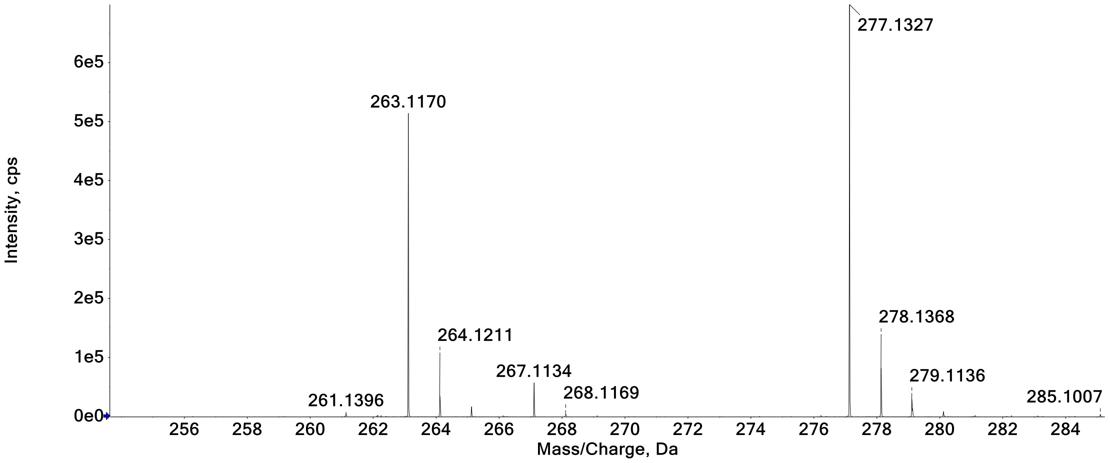

Spectrum from MASS20210609.wiff2 (sample 65) - Y18, +TOF MS (50 - 1000) from 0.079 to 0.114 min

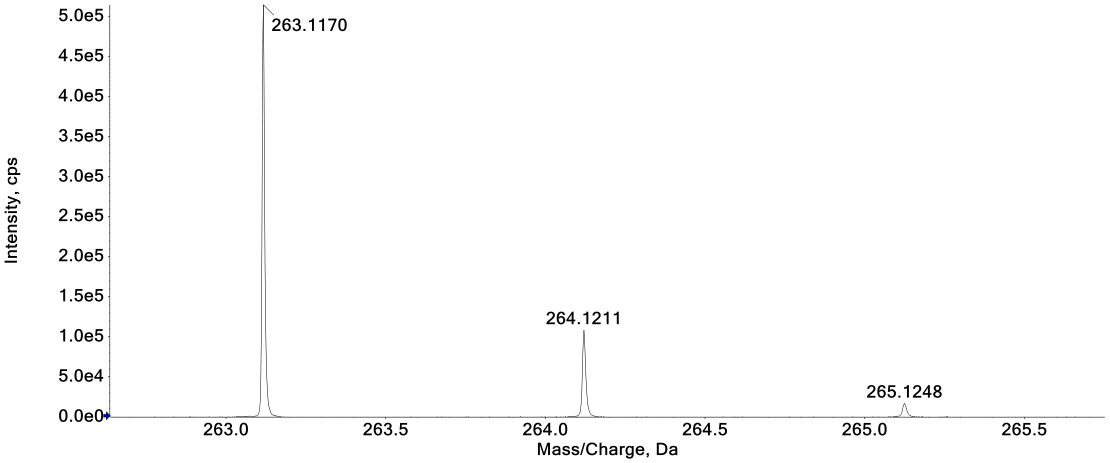

Hit	Formula	m/z	RDB	ppm	MS Rank	MSMS ppm	MSMS Rank	Found
1	C17H14N2O	263.1179	12.0	-3.4	1			NA/NA

HR-MS spectrum of 6h

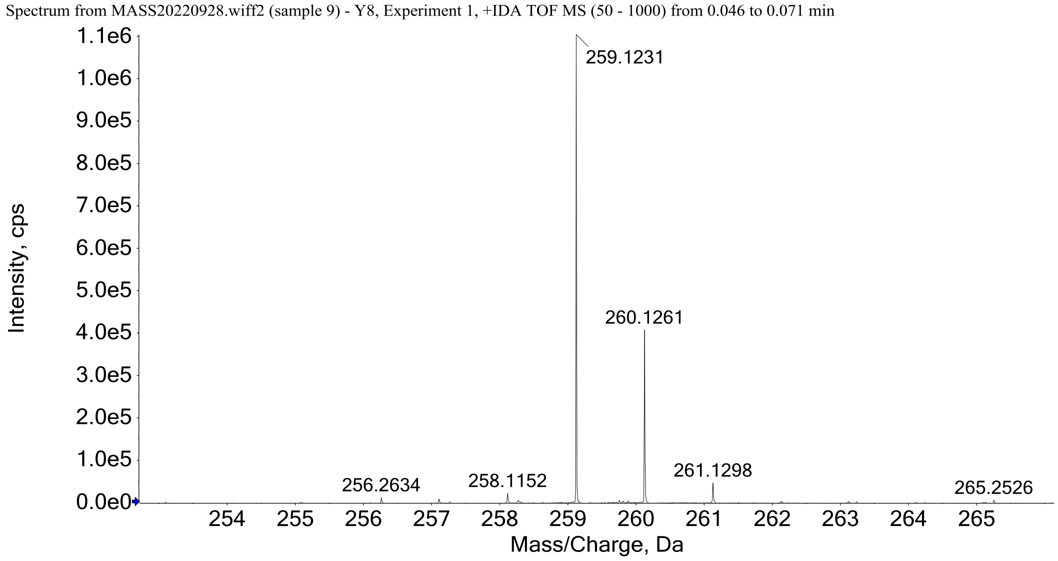

Hit	Formula	m/z	RDB	ppm	MS Rank	MSMS ppm	MSMS Rank	Found
1	C18H14N2	259.1230	13.0	0.5	1			NA/NA

HR-MS spectrum of 6i

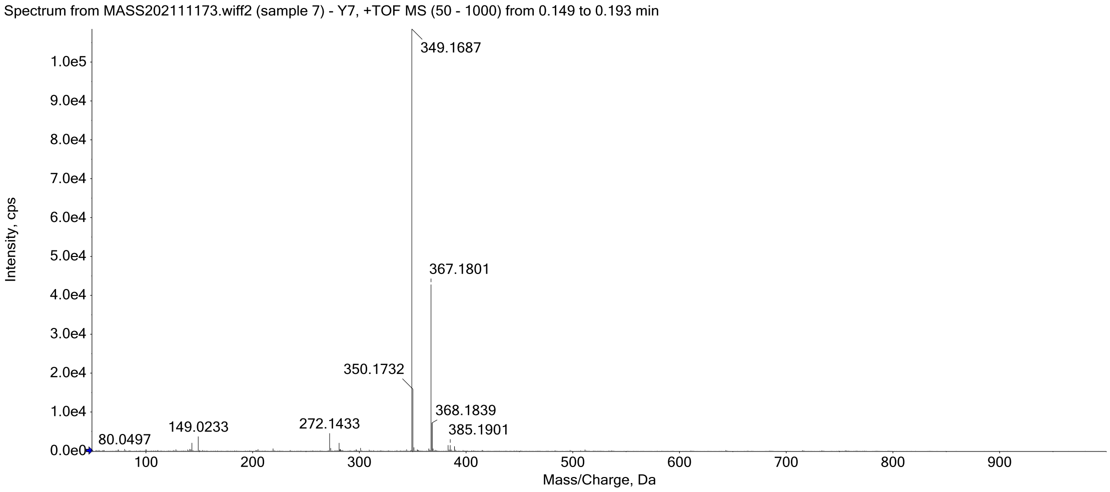

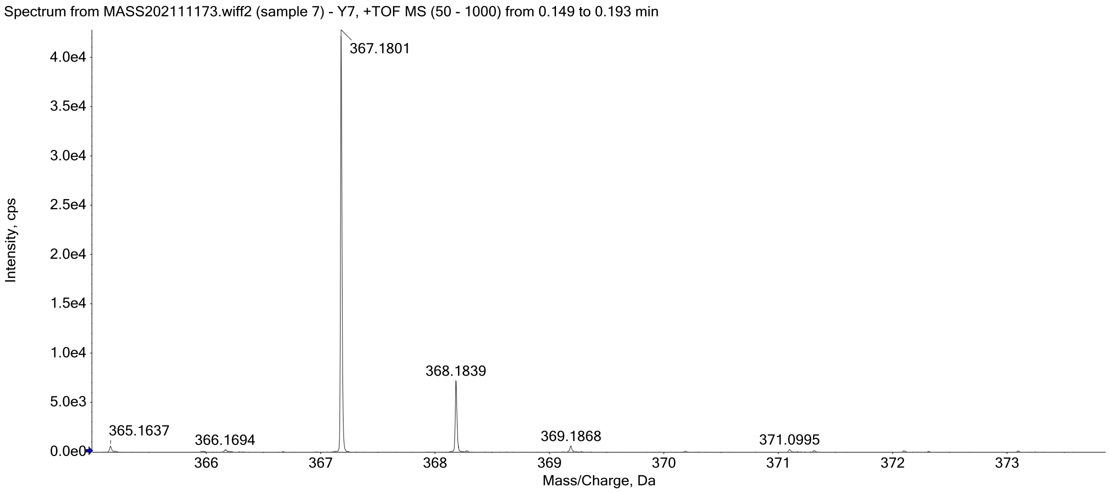

Hit	Formula	m/z	RDB	ppm	MS Rank	MSMS ppm	MSMS Rank	Found
1	C25H22N2O	367.1805	16.0	-1.1	1			NA/NA

HR-MS spectrum of 6j

Spectrum from MASS20211013.wiff2 (sample 50) - Y26, +TOF MS (50 - 1000) from 0.149 to 0.220 min

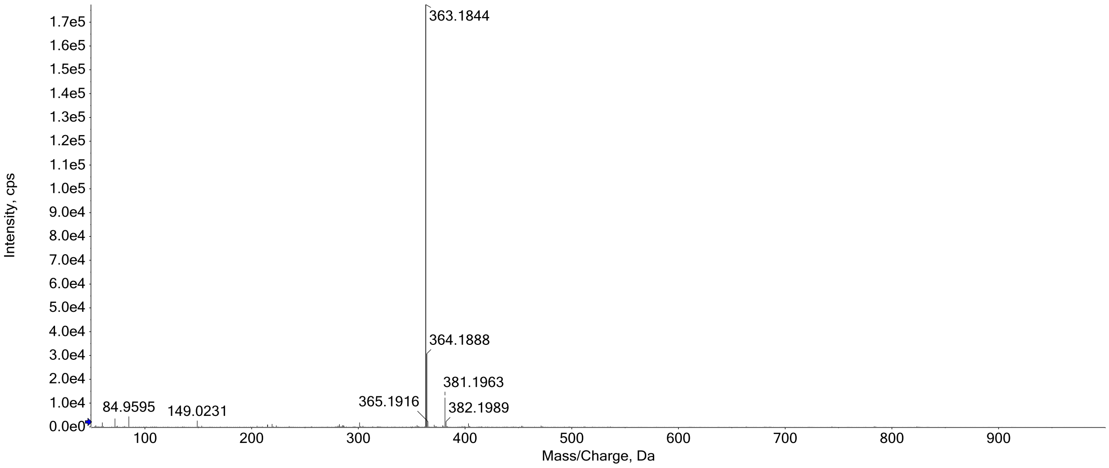

Spectrum from MASS20211013.wiff2 (sample 50) - Y26, +TOF MS (50 - 1000) from 0.149 to 0.220 min

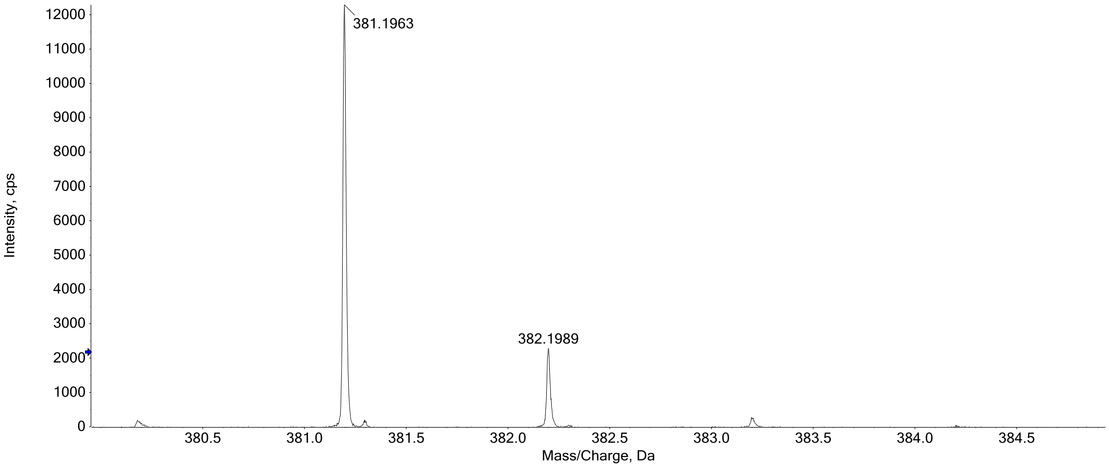

Hit	Formula	m/z	RDB	ppm	MS Rank	MSMS ppm	MSMS Rank	Found
1	C26H24N2O	381.1961	16.0	0.4	1			NA/NA

HR-MS spectrum of 6k

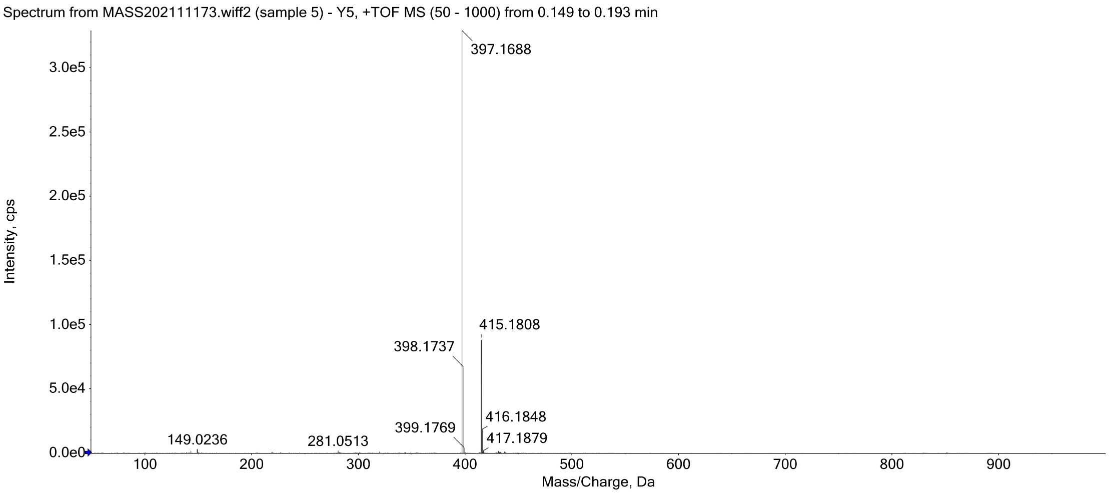

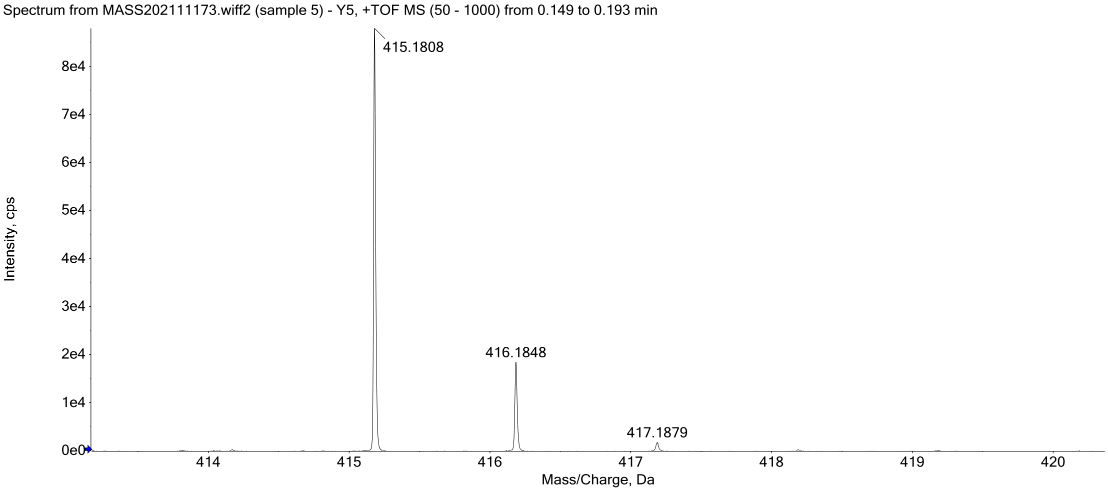

Hit	Formula	m/z	RDB	ppm	MS Rank	MSMS ppm	MSMS Rank	Found
1	C29H22N2O	415.1805	20.0	0.7	1			NA/NA

HR-MS spectrum of 6l

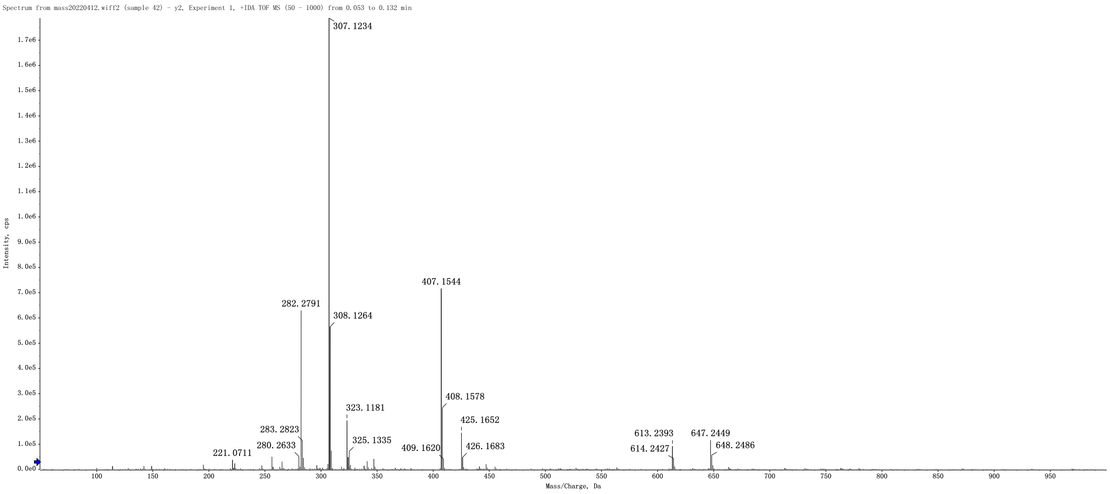

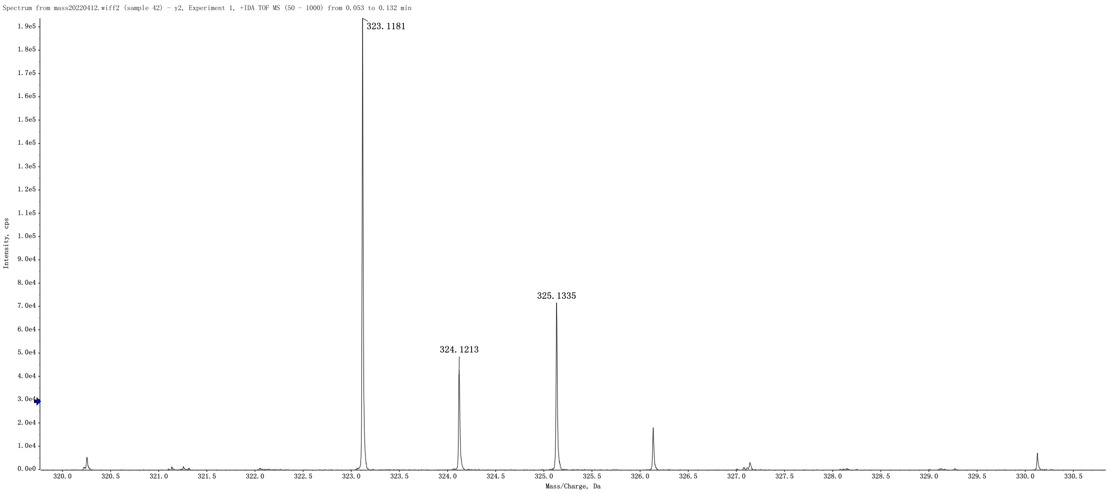

Hit	Formula	m/z	RDB	ppm	MS Rank	MSMS ppm	MSMS Rank	Found
1	C22H14N2O	323.1179	17.0	0.7	1			NA/NA

HR-MS spectrum of 6m

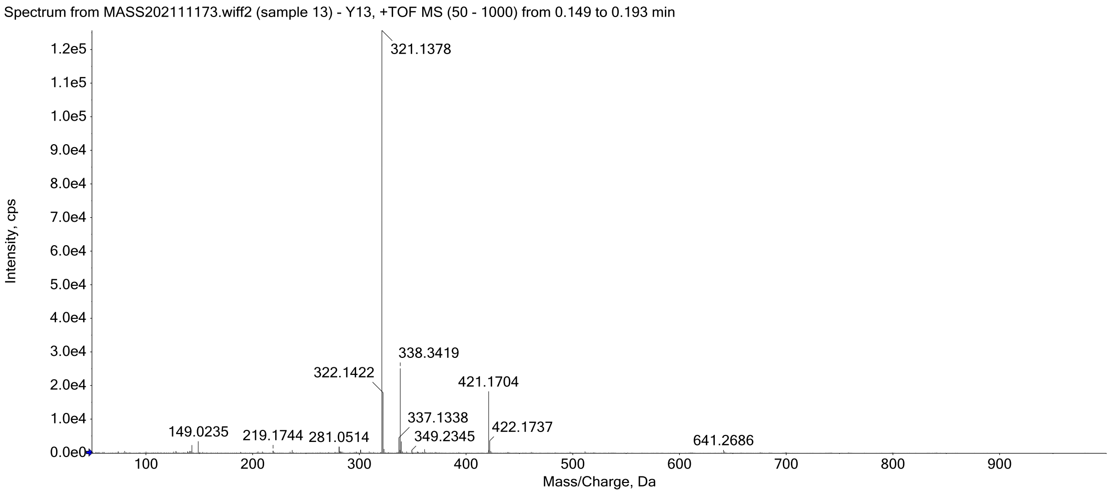

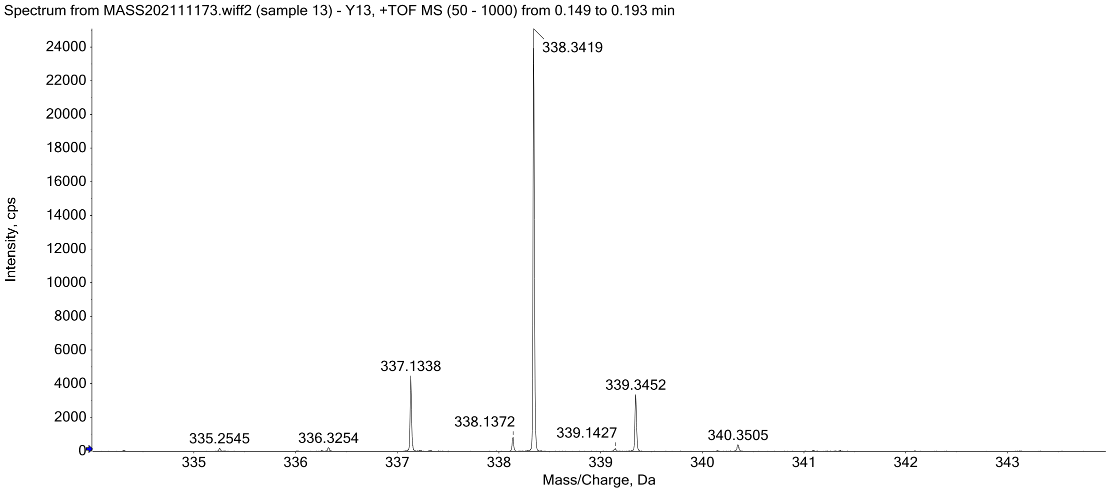

Spectrum from MASS202111173.wiff2 (sample 13) - Y13, +TOF MS (50 - 1000) from 0...02111173.wiff2 (sample 13) - Y13, +TOF MS (50 - 1000) from 0.237 to 0.299 min]

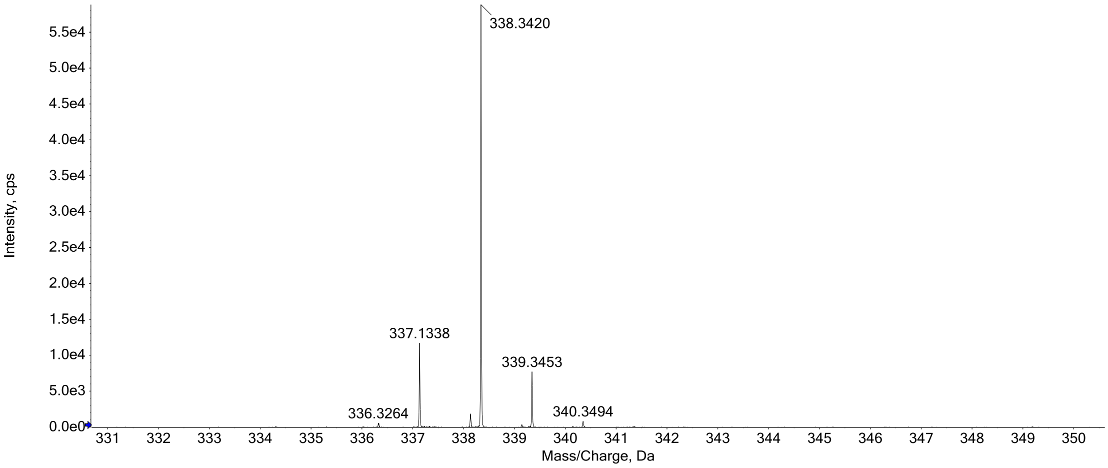

Spectrum from MASS202111173.wiff2 (sample 13) - Y13, +TOF MS (50 - 1000) from 0...02111173.wiff2 (sample 13) - Y13, +TOF MS (50 - 1000) from 0.237 to 0.299 min]

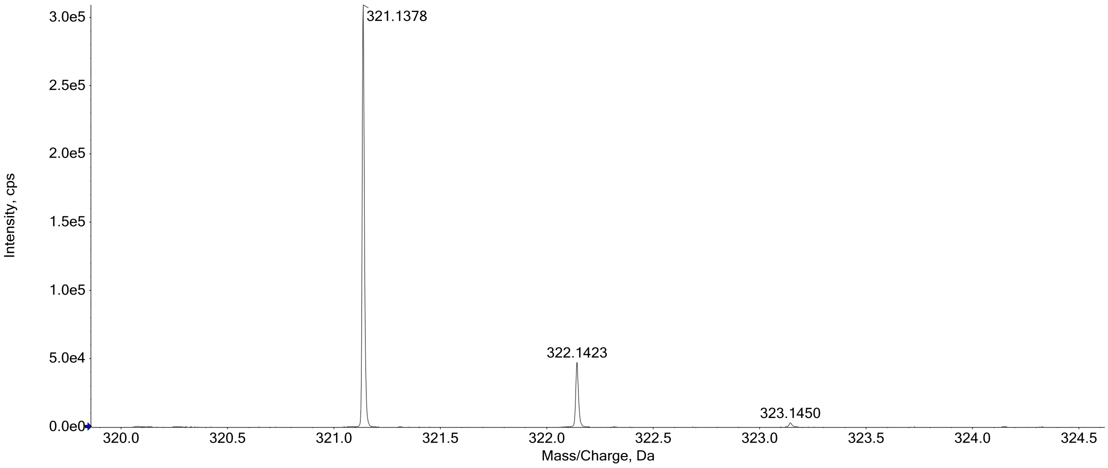

Hit	Formula	m/z	RDB	ppm	MS Rank	MSMS ppm	MSMS Rank	Found
1	C23H16N2	321.1386	17.0	-2.6	1			NA/NA

HR-MS spectrum of 6n

Spectrum from MASS202111173.wiff2 (sample 1) - Y1, +TOF MS (50 - 1000) from 0.1...S202111173.wiff2 (sample 1) - Y1, +TOF MS (50 - 1000) from 0.237 to 0.299 min]

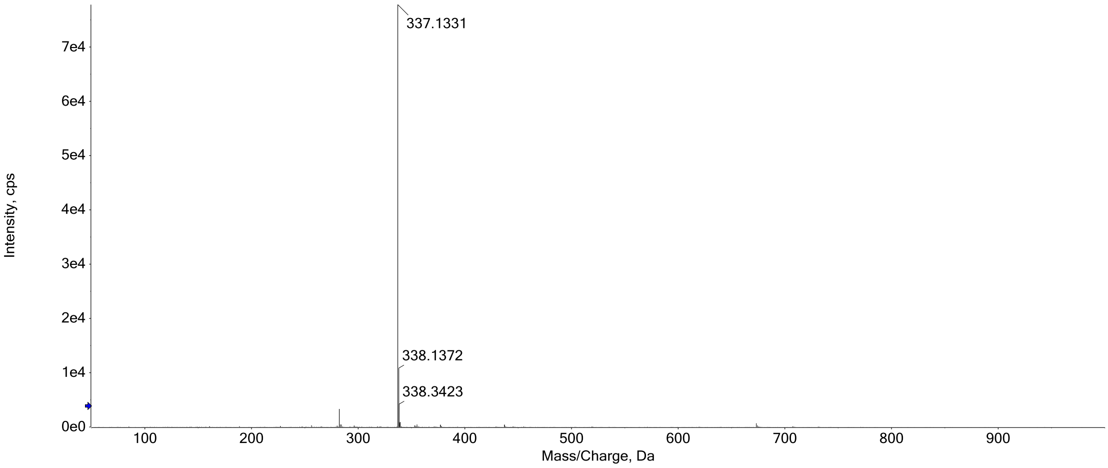

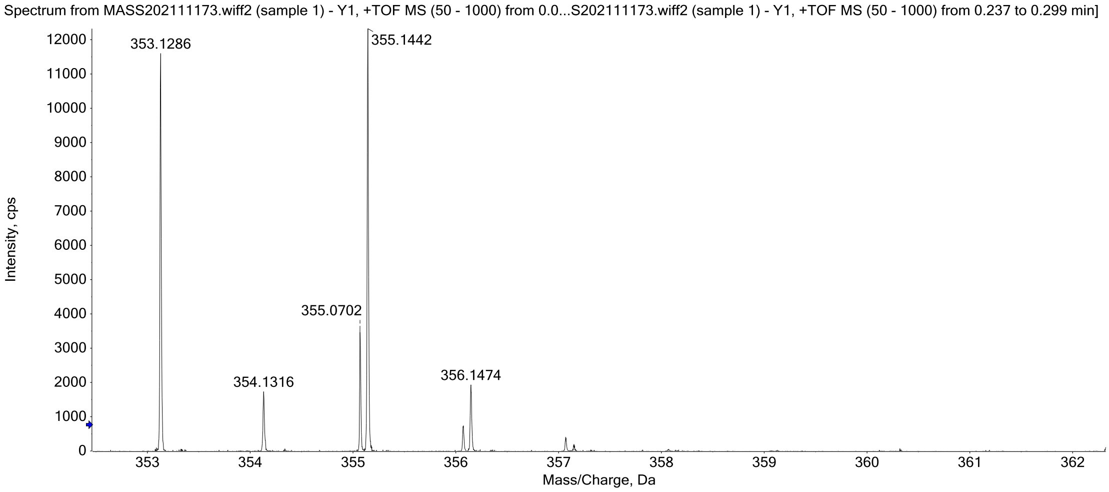

Hit	Formula	m/z	RDB	ppm	MS Rank	MSMS ppm	MSMS Rank	Found
1	C23H18N2O2	355.1441	16.0	0.3	1			NA/NA

HR-MS spectrum of 6o

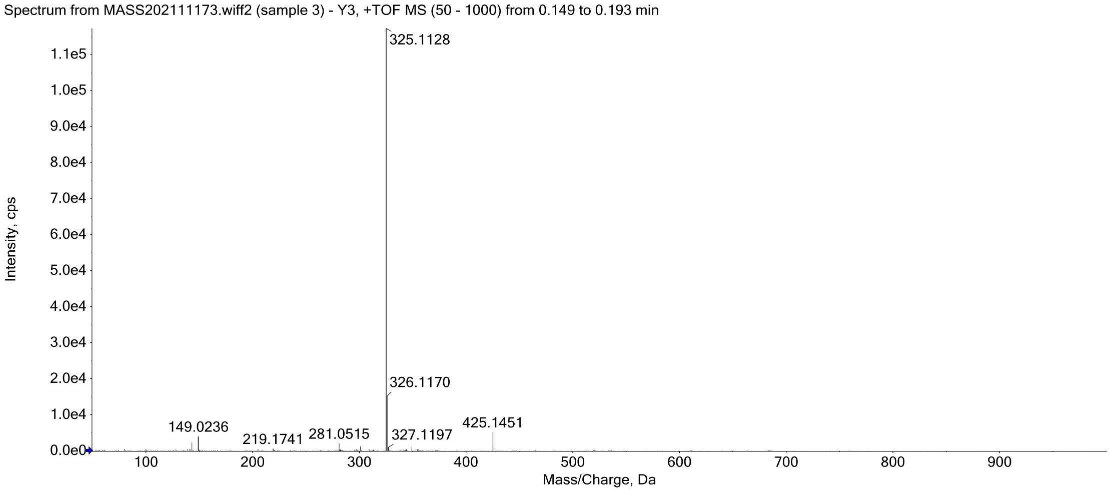

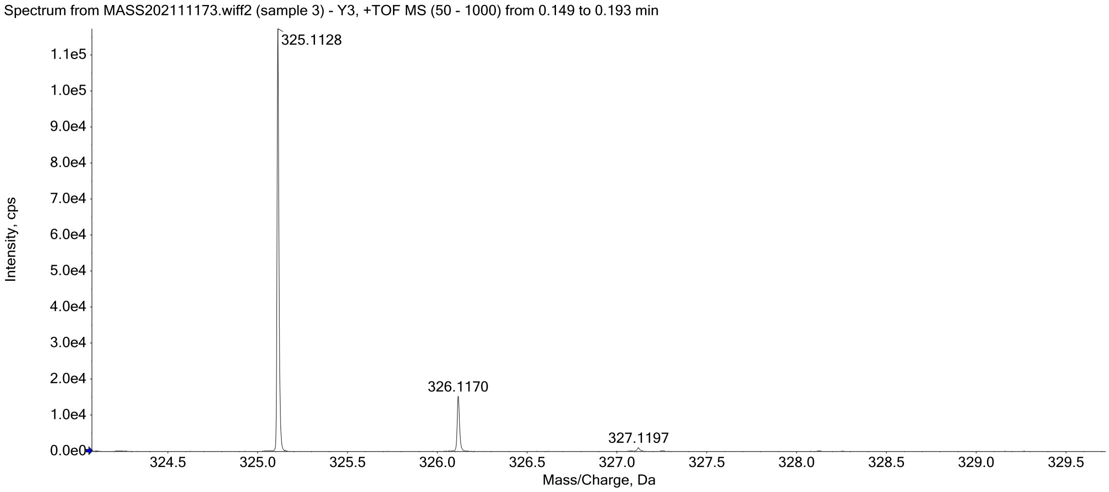

Hit	Formula	m/z	RDB	ppm	MS Rank	MSMS ppm	MSMS Rank	Found
1	C22H13FN2	325.1136	17.0	-2.3	1			NA/NA

HR-MS spectrum of 6p

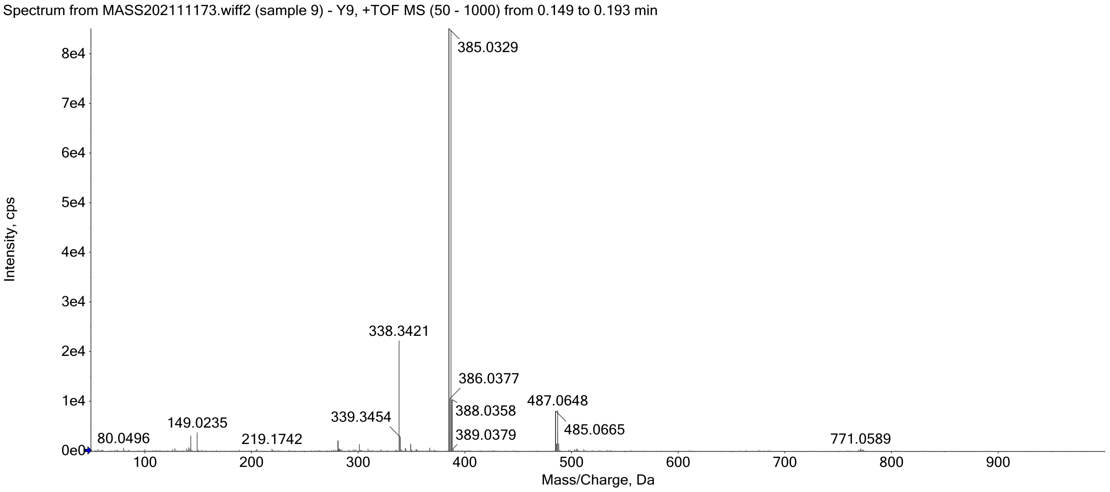

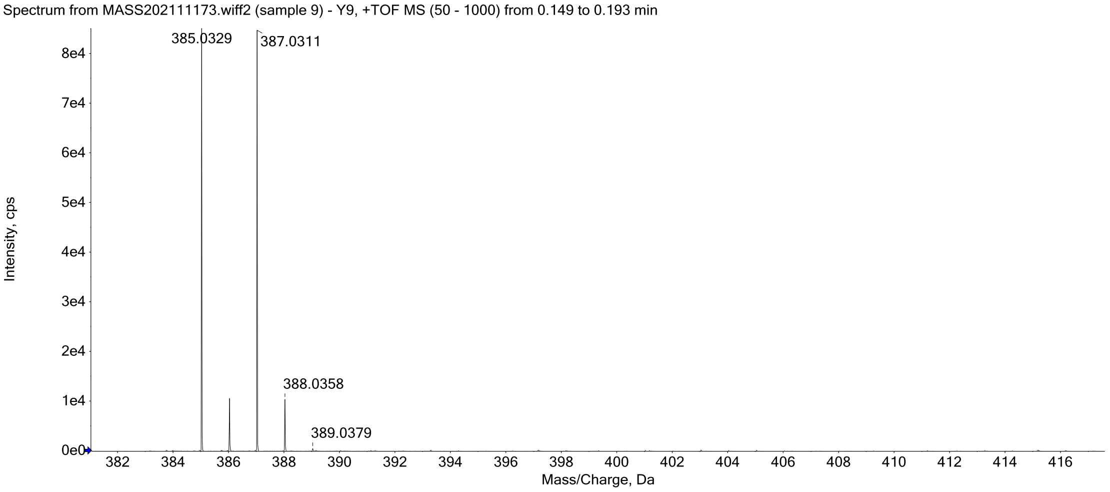

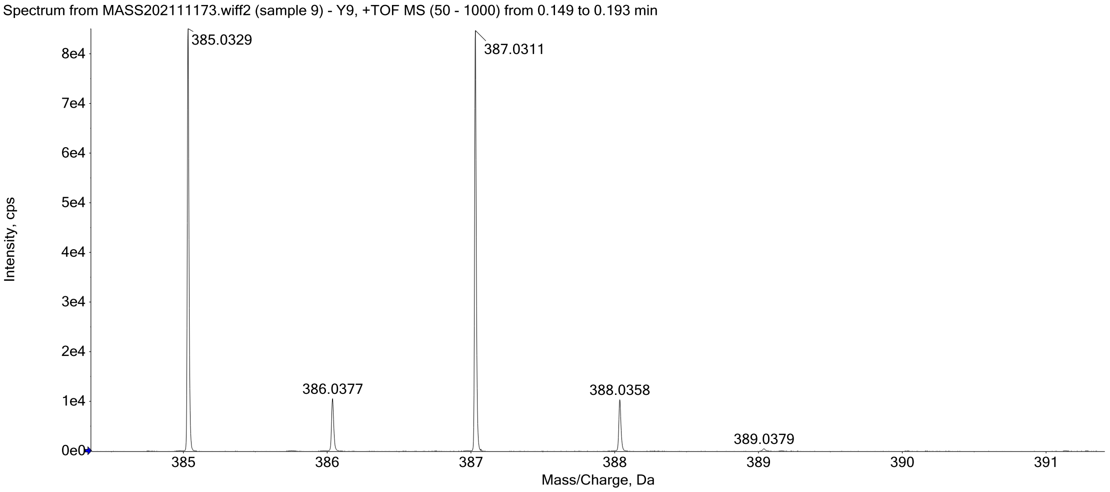

Hit	Formula	m/z	RDB	ppm	MS Rank	MSMS ppm	MSMS Rank	Found
1	C22H13BrN2	385.0335	17.0	-1.5	1			NA/NA

HR-MS spectrum of 6q

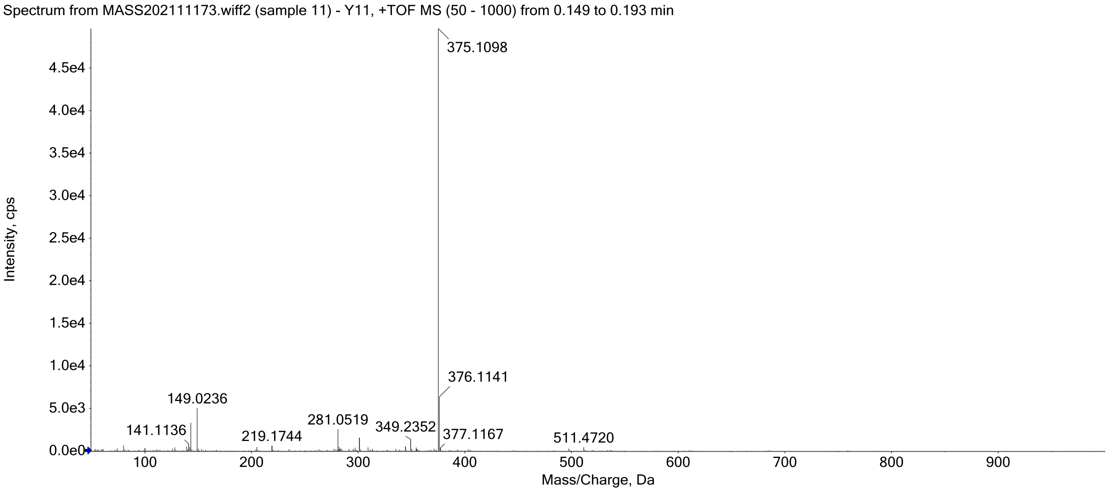

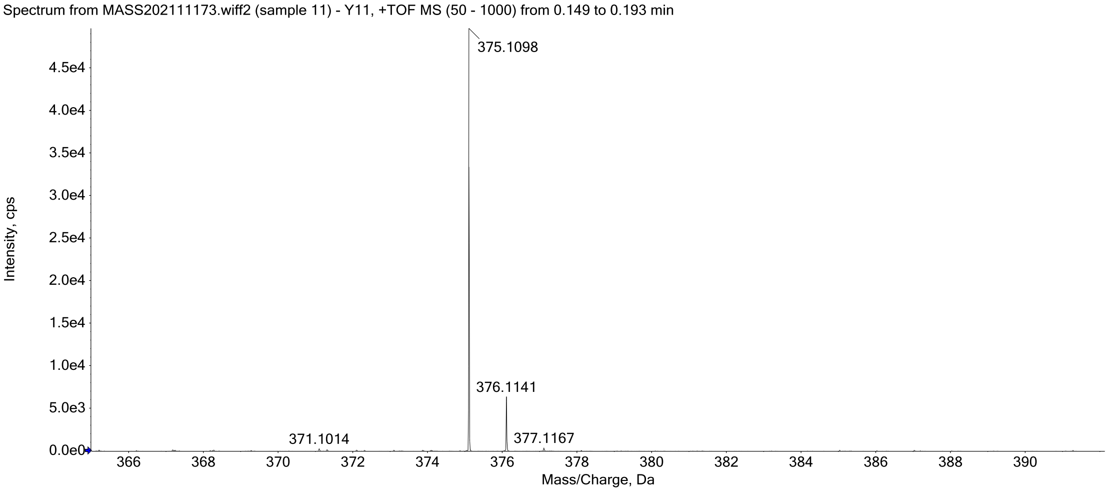

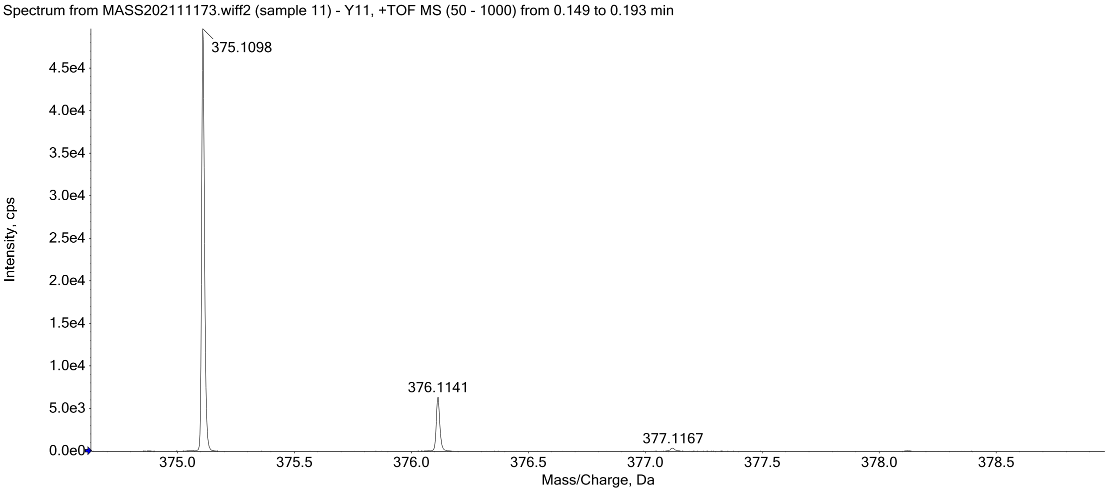

Hit	Formula	m/z	RDB	ppm	MS Rank	MSMS ppm	MSMS Rank	Found
1	C23H13F3N2	375.1104	17.0	-1.5	1			NA/NA

HR-MS spectrum of 6r

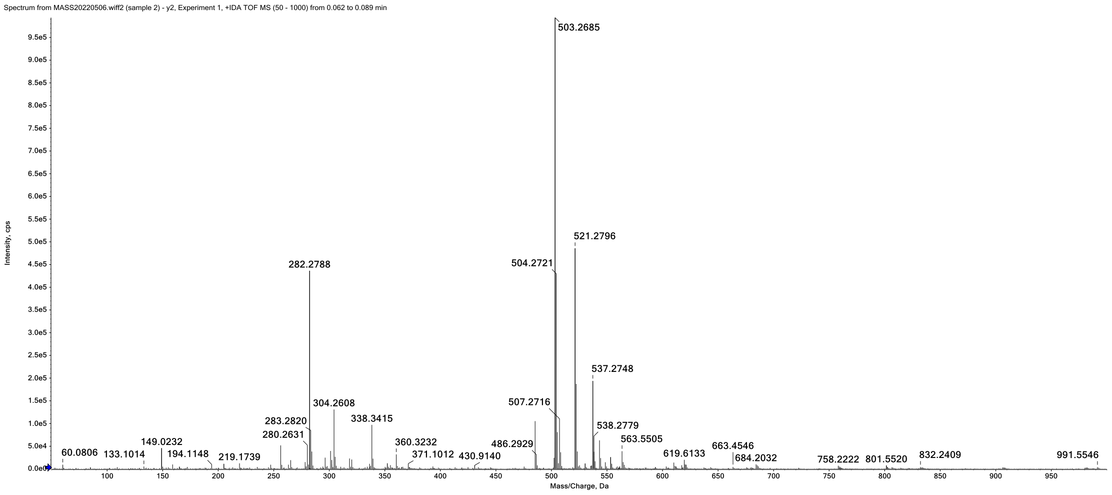

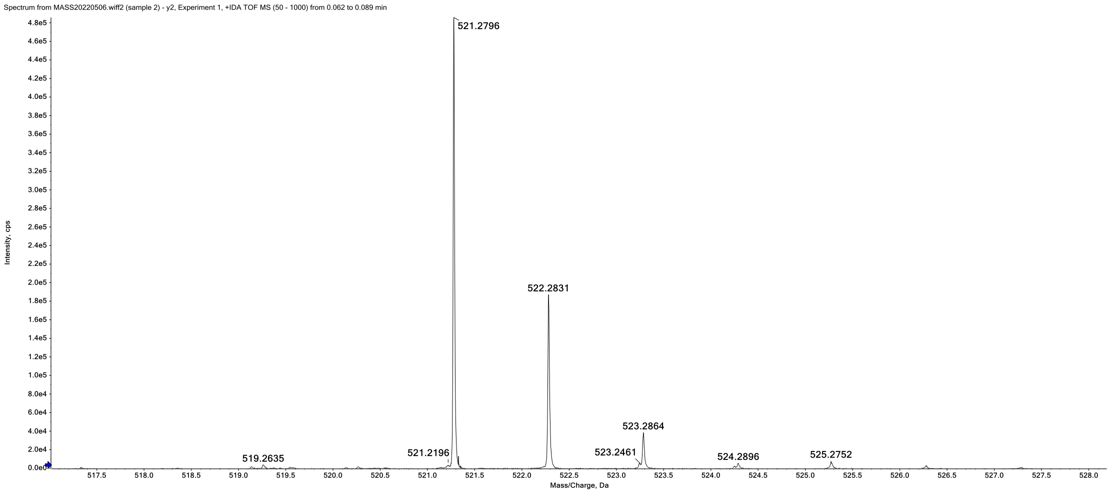

Hit	Formula	m/z	RDB	ppm	MS Rank	MSMS ppm	MSMS Rank	Found
1	C34H36N2O3	521.2799	18.0	-0.5	1			NA/NA

HR-MS spectrum of 6s

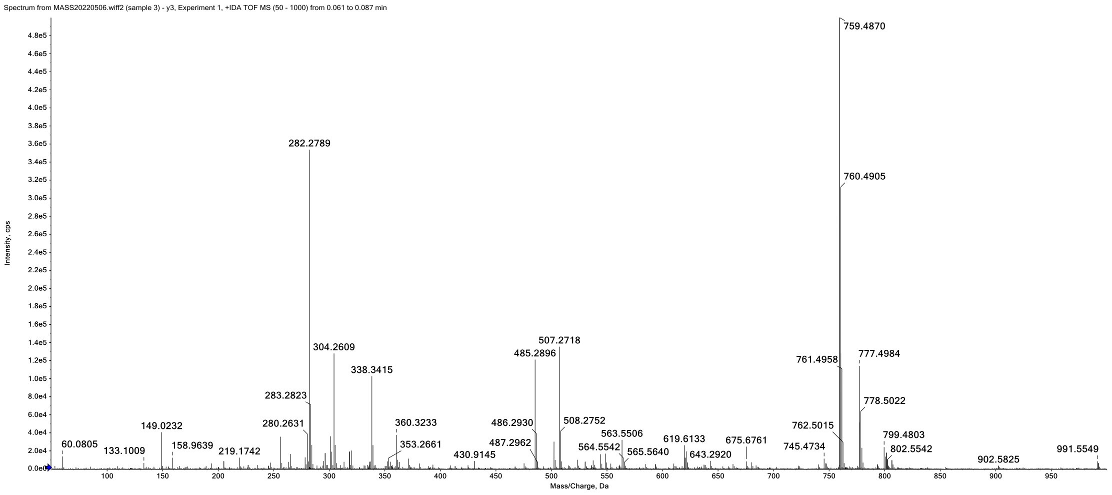

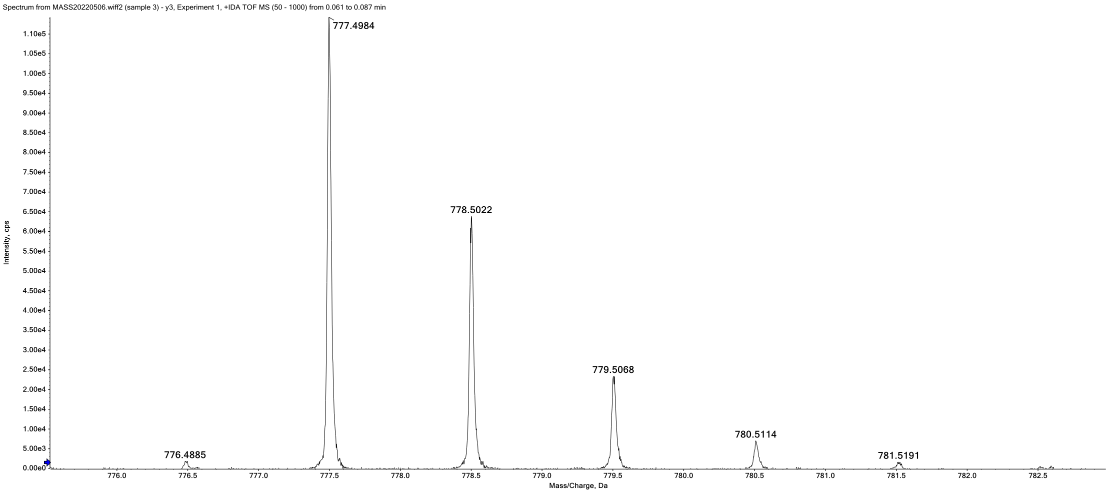

Hit	Formula	m/z	RDB	ppm	MS Rank	MSMS ppm	MSMS Rank	Found
1	C53H64N2O3	777.4990	23.0	-0.7	1			NA/NA

HR-MS spectrum of 6t

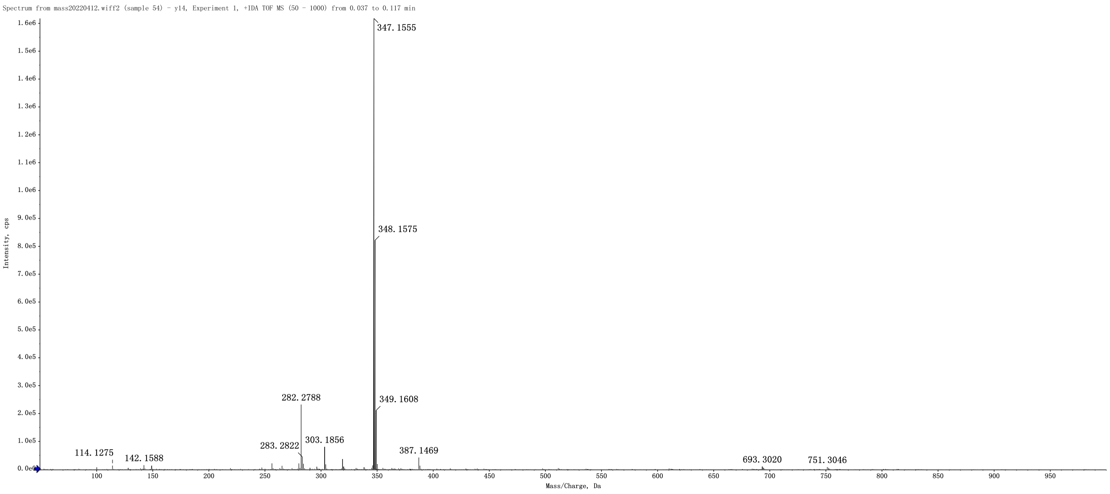

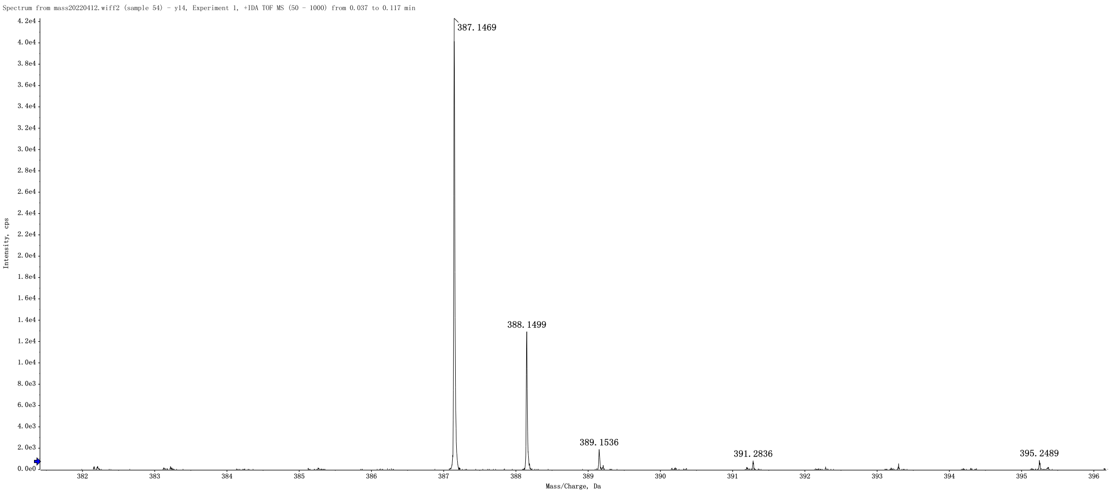

HR-MS spectrum of 6u

Spectrum from MASS202111173.wiff2 (sample 15) - Y15, +TOF MS (50 - 1000) from 0.149 to 0.193 min

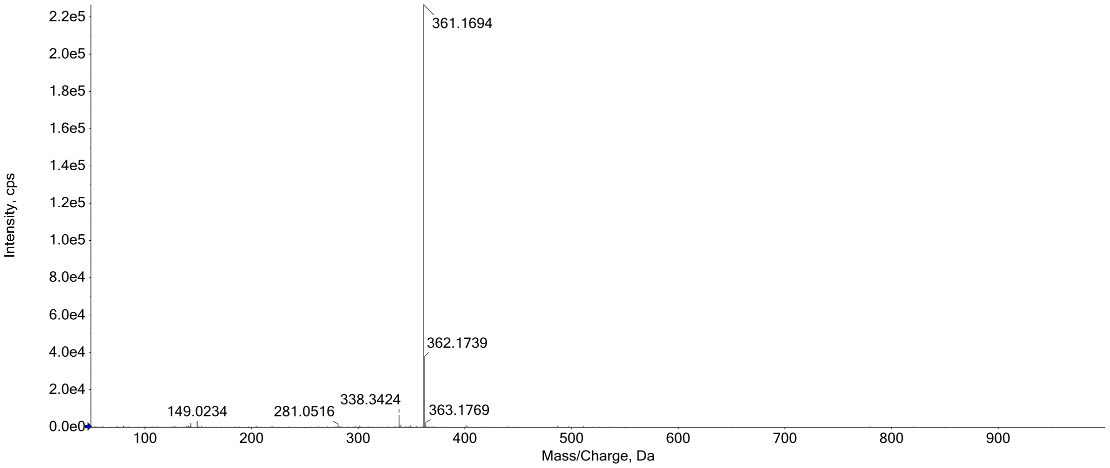

Spectrum from MASS202111173.wiff2 (sample 15) - Y15, +TOF MS (50 - 1000) from 0.149 to 0.193 min

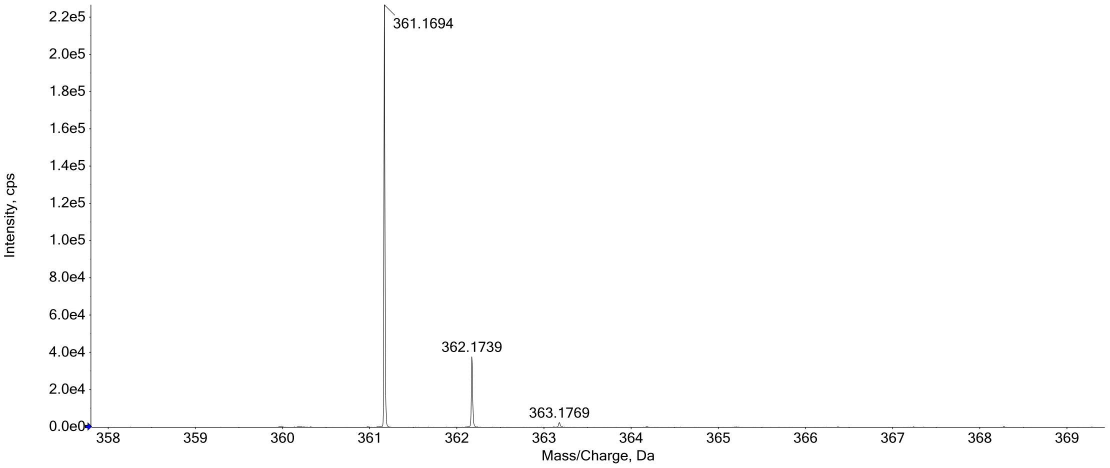

Hit	Formula	m/z	RDB	ppm	MS Rank	MSMS ppm	MSMS Rank	Found
1	C26H20N2	361.1699	18.0	-1.5	1			NA/NA

HR-MS spectrum of 7a

Spectrum from MASS20211013.wiff2 (sample 37) - Y13, +TOF MS (50 - 1000) from 0.044 to 0.114 min

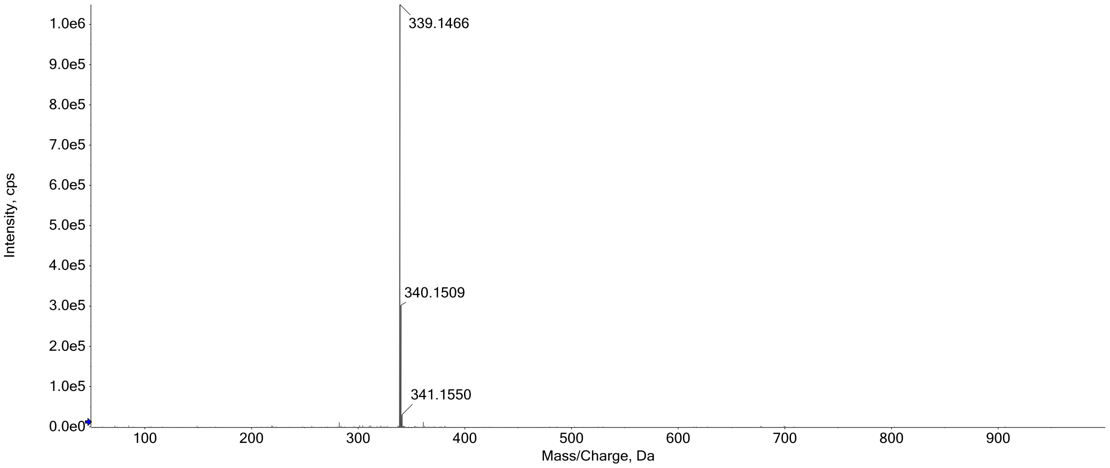

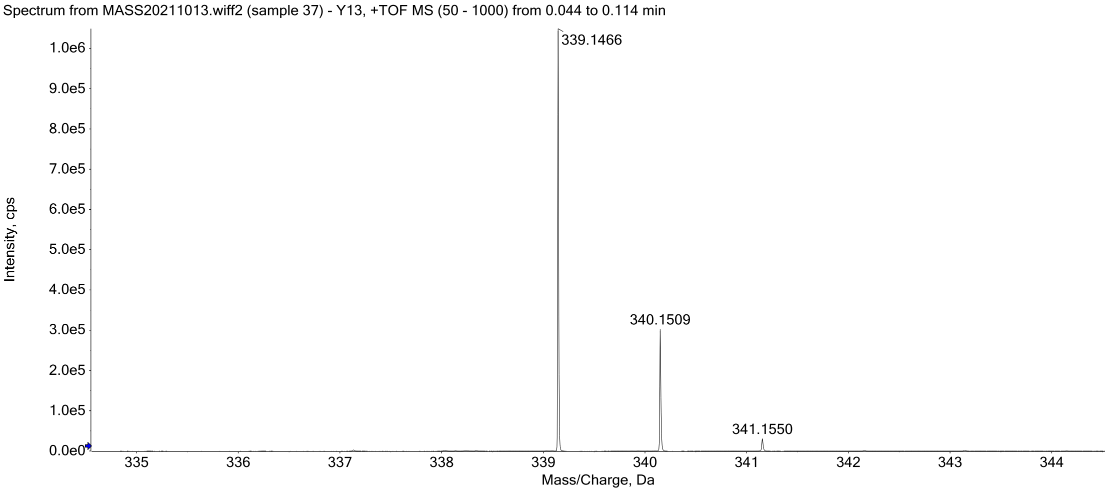

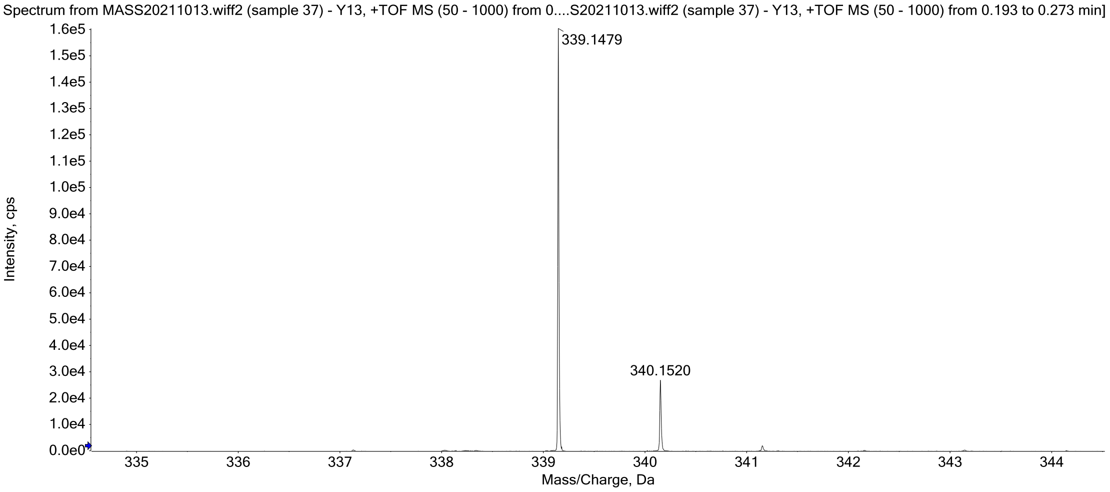

Hit	Formula	m/z	RDB	ppm	MS Rank	MSMS ppm	MSMS Rank	Found
1	C23H18N2O	339.1492	16.0	-3.8	1			NA/NA

HR-MS spectrum of 7b

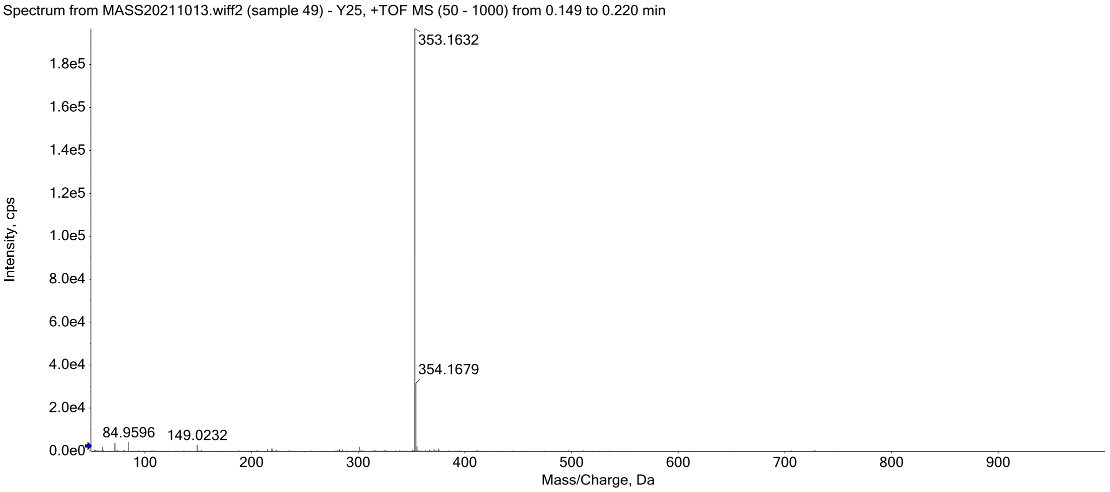

Spectrum from MASS20211013.wiff2 (sample 49) - Y25, +TOF MS (50 - 1000) from 0.149 to 0.220 min

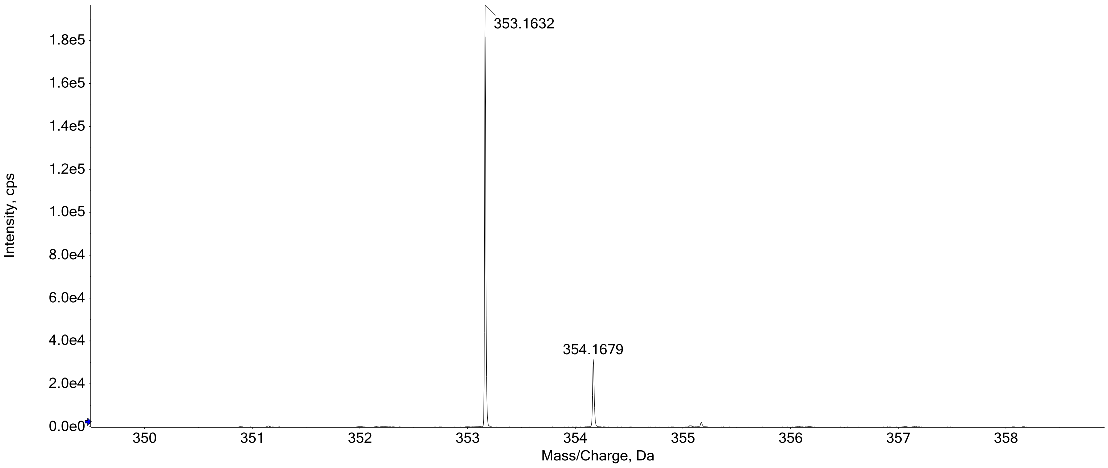

Hit	Formula	m/z	RDB	ppm	MS Rank	MSMS ppm	MSMS Rank	Found
1	C24H20N2O	353.1648	16.0	-4.6	1			NA/NA

HR-MS spectrum of 7c

Spectrum from MASS20211013.wiff2 (sample 45) - Y21, +TOF MS (50 - 1000) from 0.114 to 0.184 min

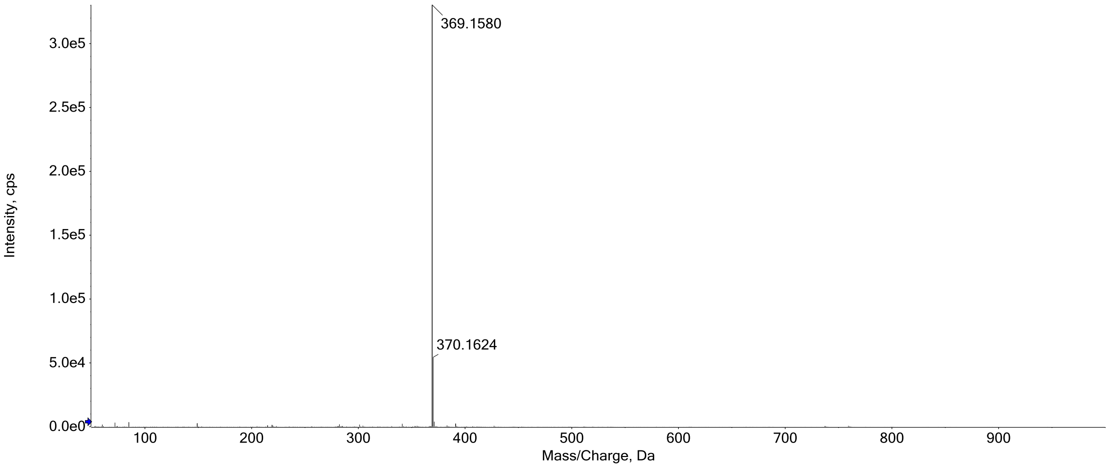

Spectrum from MASS20211013.wiff2 (sample 45) - Y21, +TOF MS (50 - 1000) from 0.114 to 0.184 min

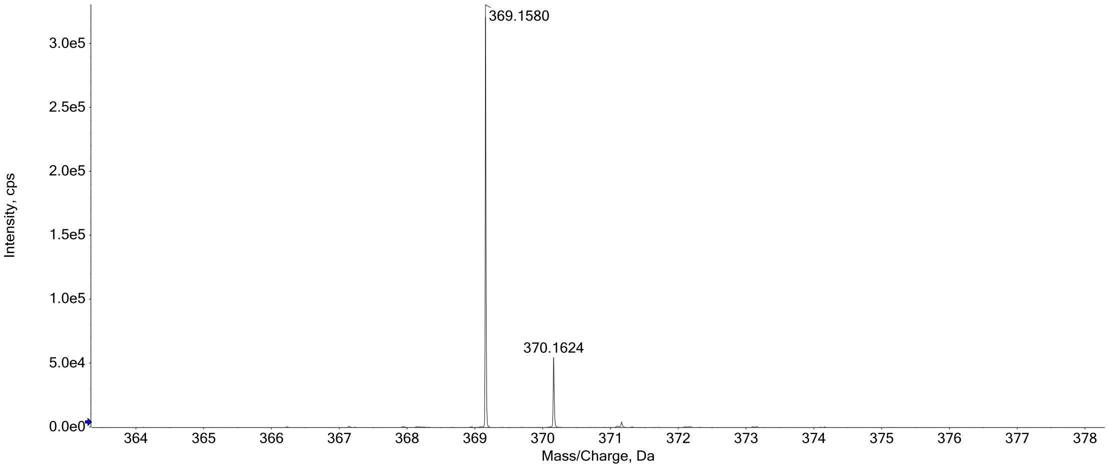

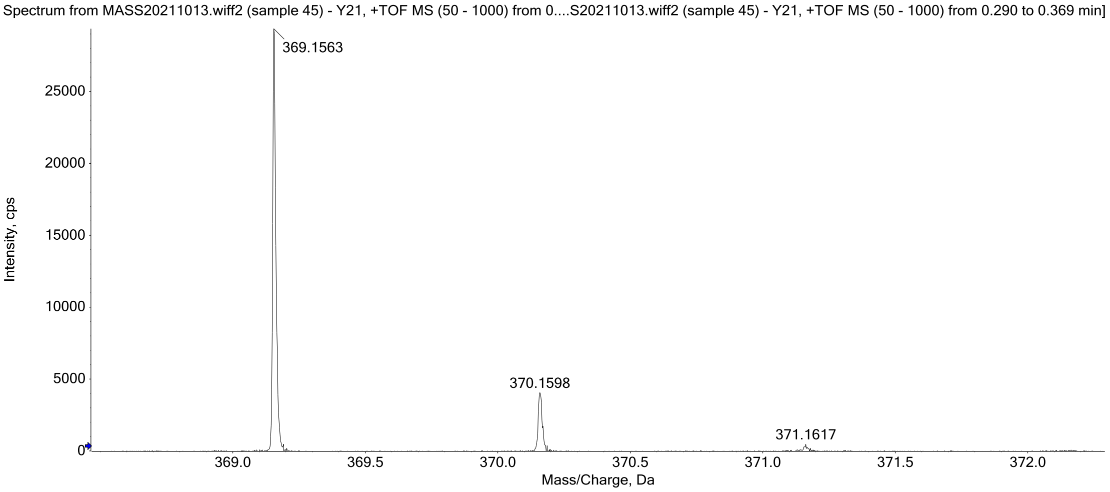

Hit	Formula	m/z	RDB	ppm	MS Rank	MSMS ppm	MSMS Rank	Found
1	C24H20N2O2	369.1598	16.0	-4.2	1			NA/NA

HR-MS spectrum of 7d

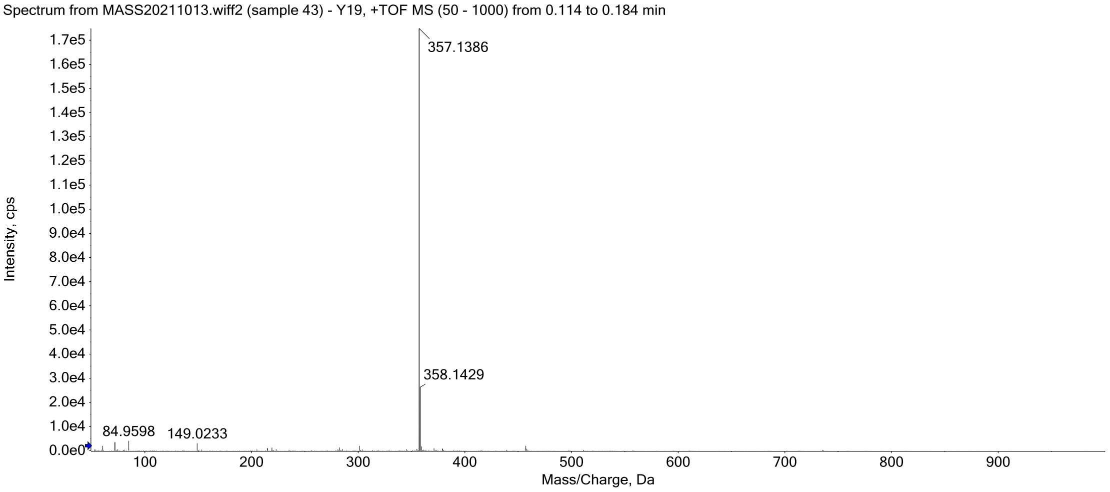

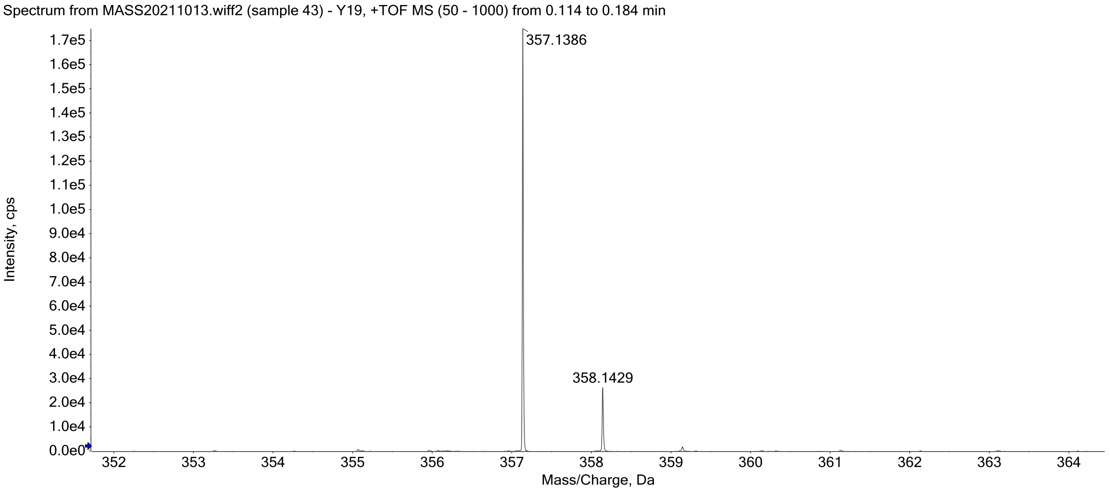

Hit	Formula	m/z	RDB	ppm	MS Rank	MSMS ppm	MSMS Rank	Found
1	C23H17FN2O	357.1398	16.0	-3.3	1			NA/NA

HR-MS spectrum of 7e

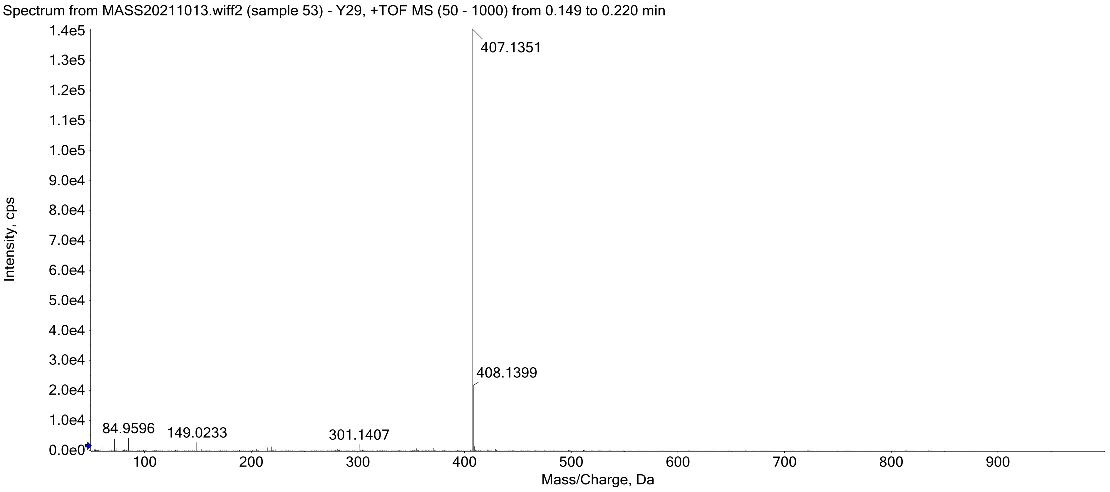

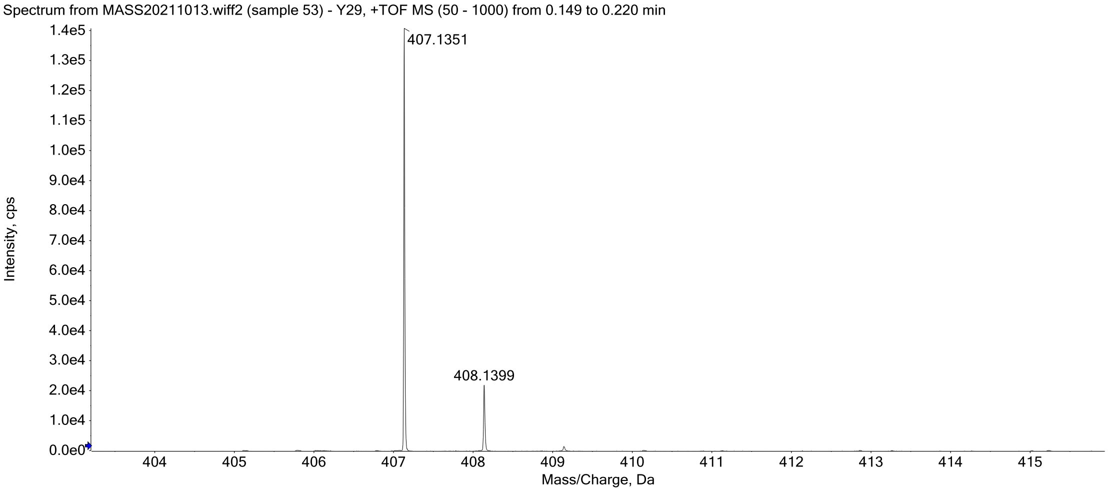

Hit	Formula	m/z	RDB	ppm	MS Rank	MSMS ppm	MSMS Rank	Found
1	C24H17F3N2O	407.1366	16.0	-3.6	1			NA/NA

HR-MS spectrum of 7f

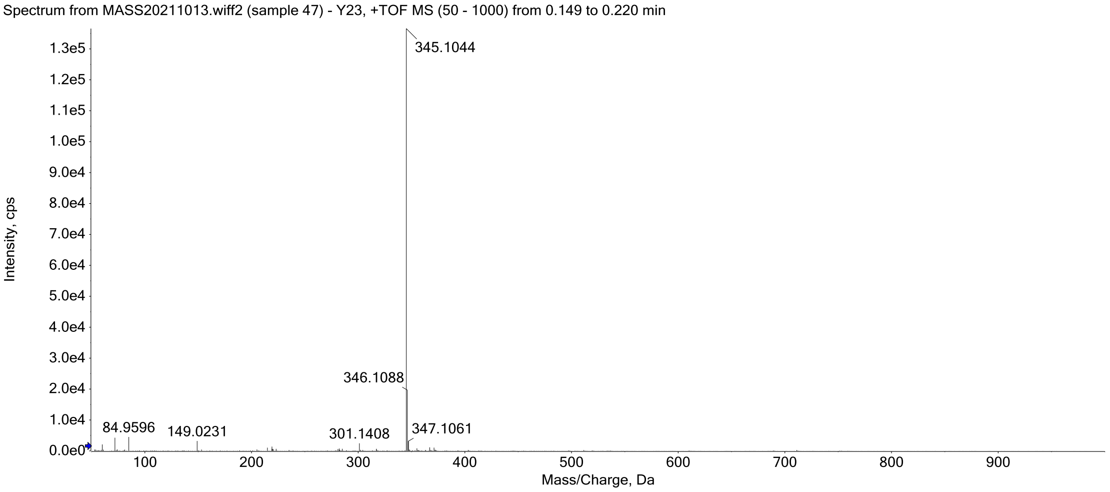

Spectrum from MASS20211013.wiff2 (sample 47) - Y23, +TOF MS (50 - 1000) from 0.149 to 0.220 min

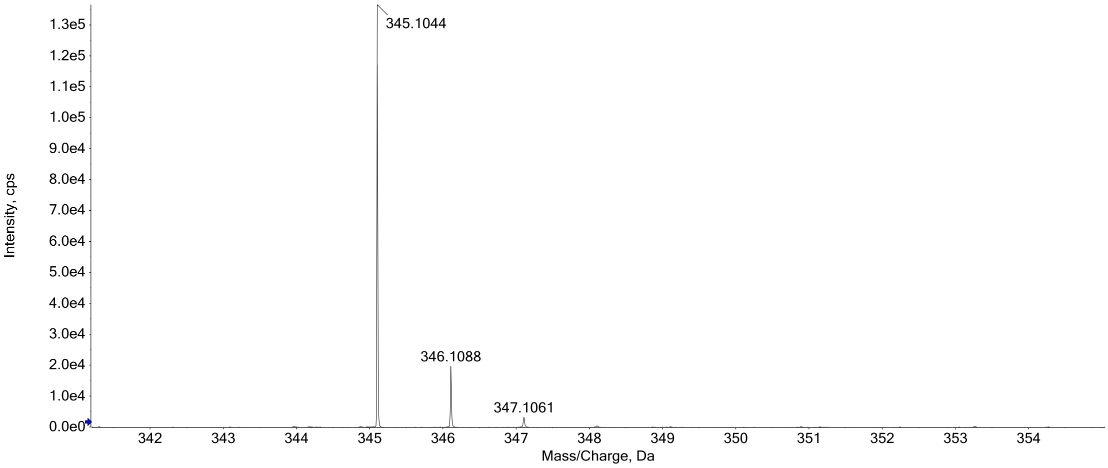

Hit	Formula	m/z	RDB	ppm	MS Rank	MSMS ppm	MSMS Rank	Found
1	C21H16N2OS	345.1056	15.0	-3.5	1			NA/NA

HR-MS spectrum of 7g

Spectrum from MASS20210609.wiff2 (sample 66) - Y19, +TOF MS (50 - 1000) from 0.079 to 0.114 min

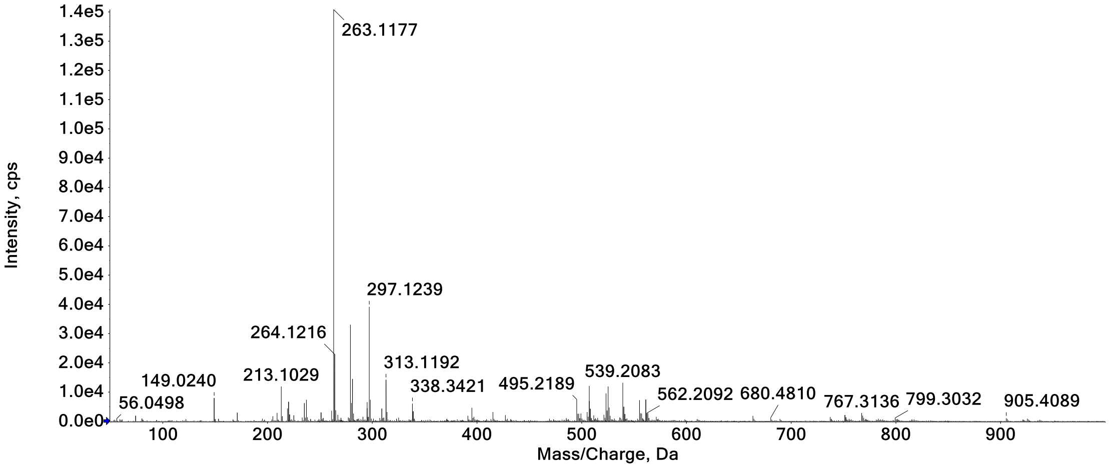

Spectrum from MASS20210609.wiff2 (sample 66) - Y19, +TOF MS (50 - 1000) from 0.079 to 0.114 min

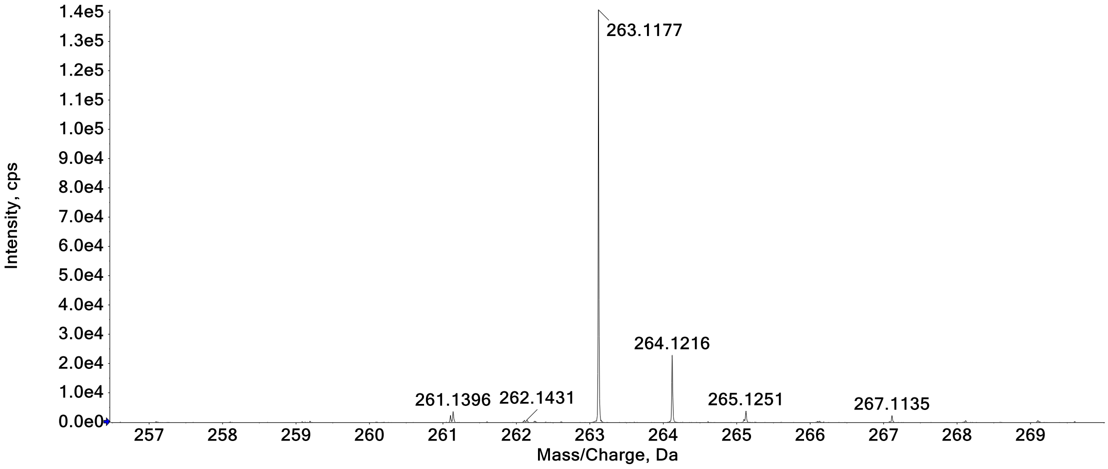

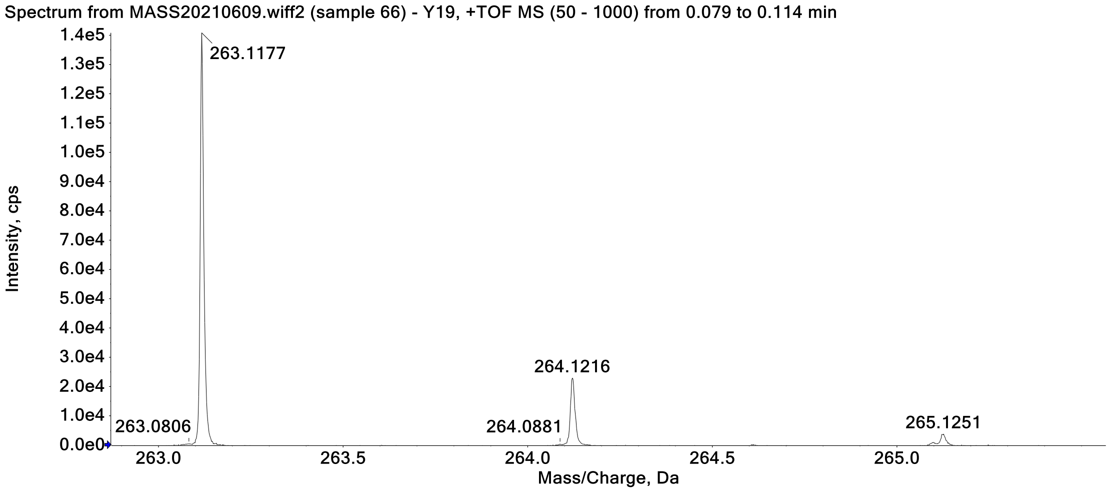

Hit	Formula	m/z	RDB	ppm	MS Rank	MSMS ppm	MSMS Rank	Found
1	C17H14N2O	263.1179	12.0	-0.7	1			NA/NA

HR-MS spectrum of 7h

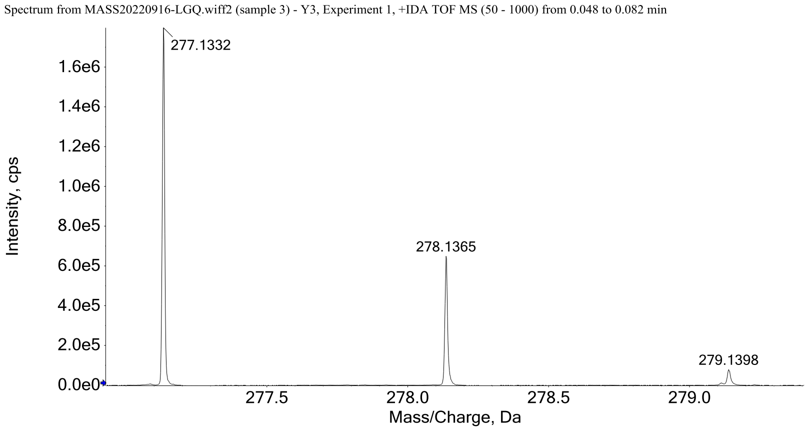

Hit	Formula	m/z	RDB	ppm	MS Rank	MSMS ppm	MSMS Rank	Found
1	C18H16N2O	277.1335	12.0	-1.2	1			NA/NA

HR-MS spectrum of 7h′

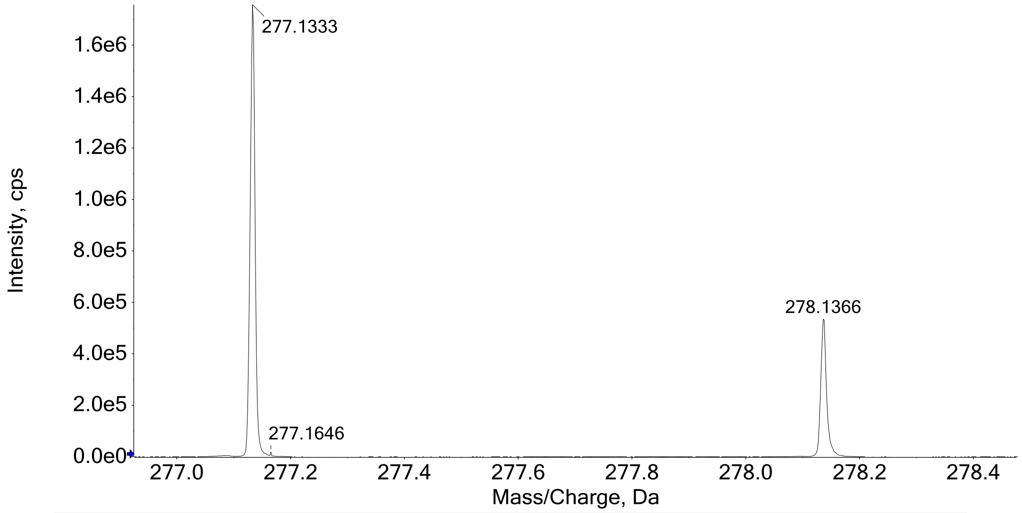

Hit	Formula	m/z	RDB	ppm	MS Rank	MSMS ppm	MSMS Rank	Found
1	C18H16N2O	277.1335	12.0	-0.9	1			NA/NA

HR-MS spectrum of 7i

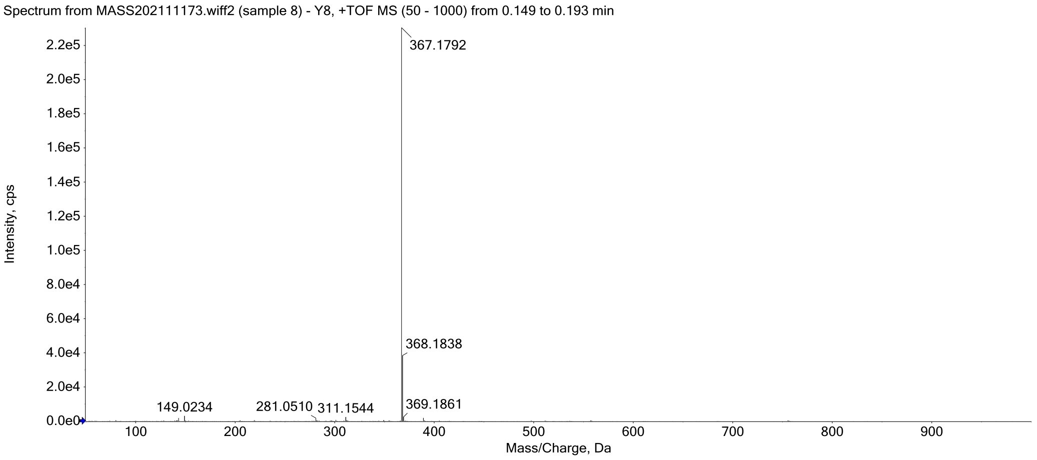

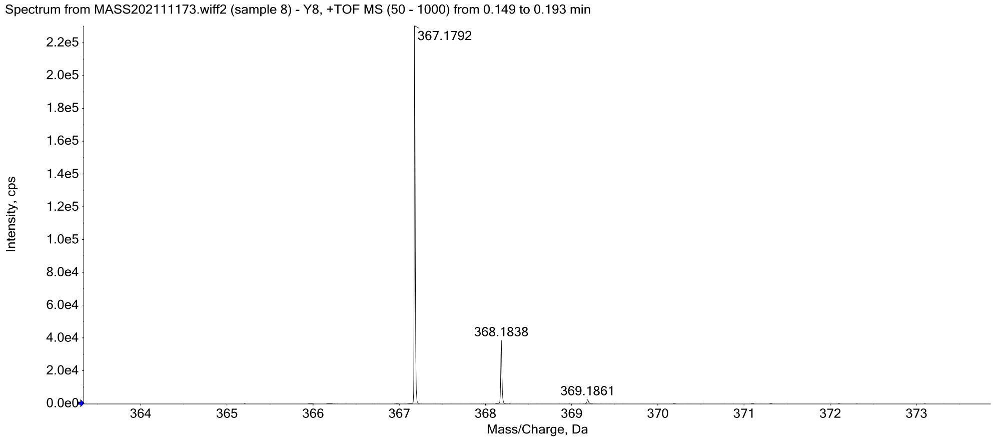

Hit	Formula	m/z	RDB	ppm	MS Rank	MSMS ppm	MSMS Rank	Found
1	C25H22N2O	367.1805	16.0	-3.5	1			NA/NA

HR-MS spectrum of 7j

Spectrum from MASS20211013.wiff2 (sample 51) - Y27, +TOF MS (50 - 1000) from 0.149 to 0.220 min

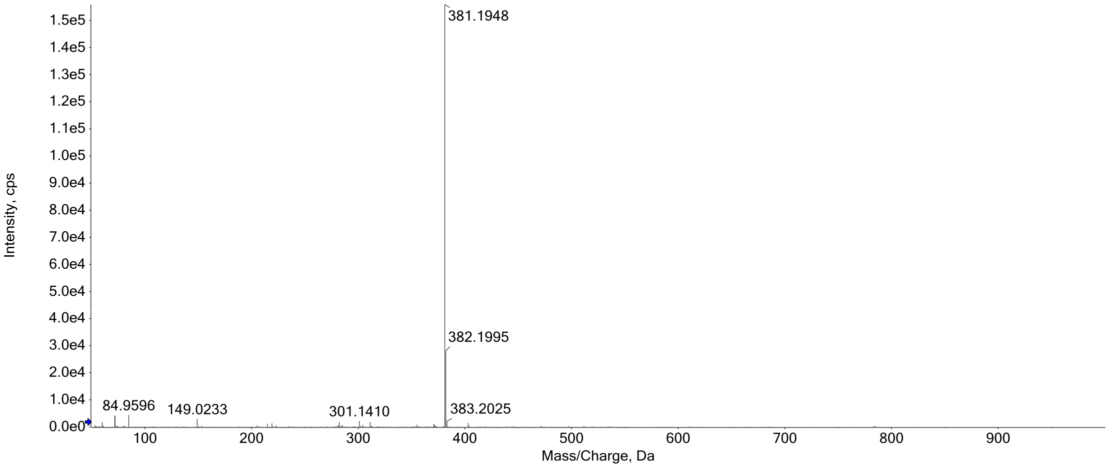

Spectrum from MASS20211013.wiff2 (sample 51) - Y27, +TOF MS (50 - 1000) from 0.149 to 0.220 min

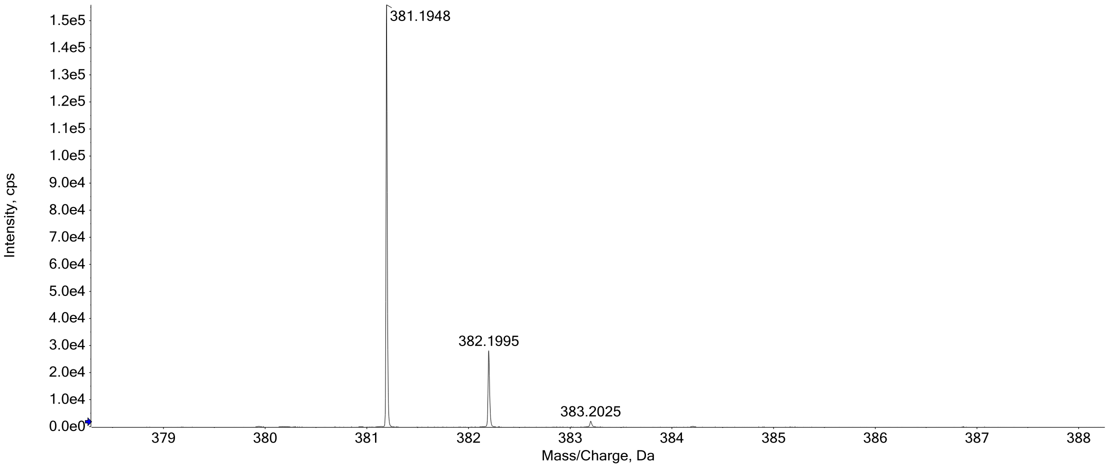

Hit	Formula	m/z	RDB	ppm	MS Rank	MSMS ppm	MSMS Rank	Found
1	C26H24N2O	381.1961	16.0	-3.5	1			NA/NA

HR-MS spectrum of 7k

Spectrum from MASS202111173.wiff2 (sample 6) - Y6, +TOF MS (50 - 1000) from 0.149 to 0.193 min

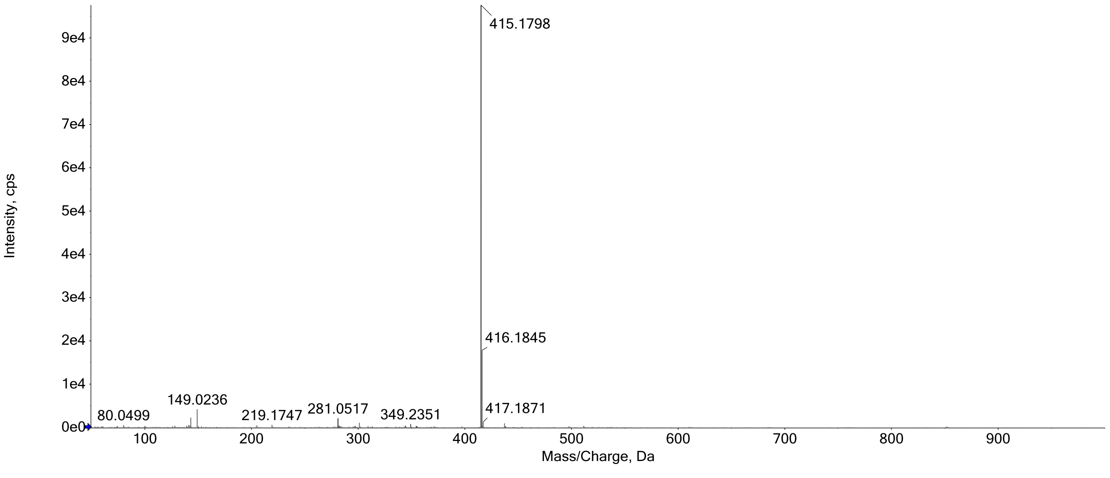

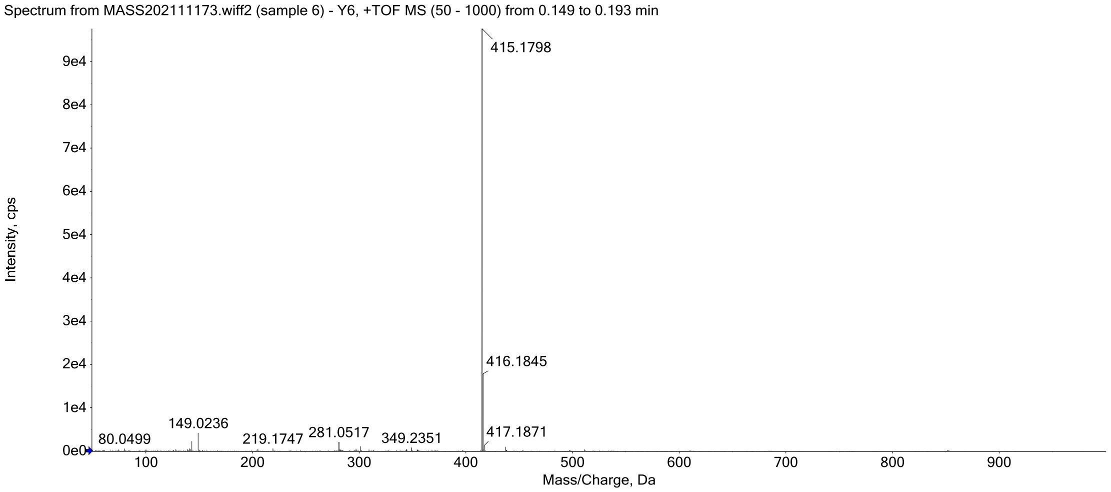

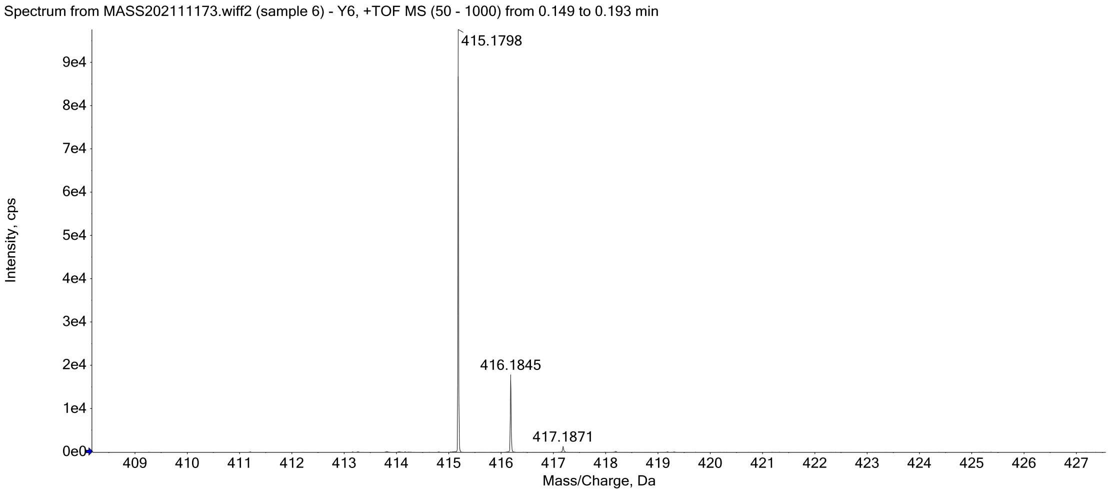

Hit	Formula	m/z	RDB	ppm	MS Rank	MSMS ppm	MSMS Rank	Found
1	C29H22N2O	415.1805	20.0	-1.7	1			NA/NA

HR-MS spectrum of 7l

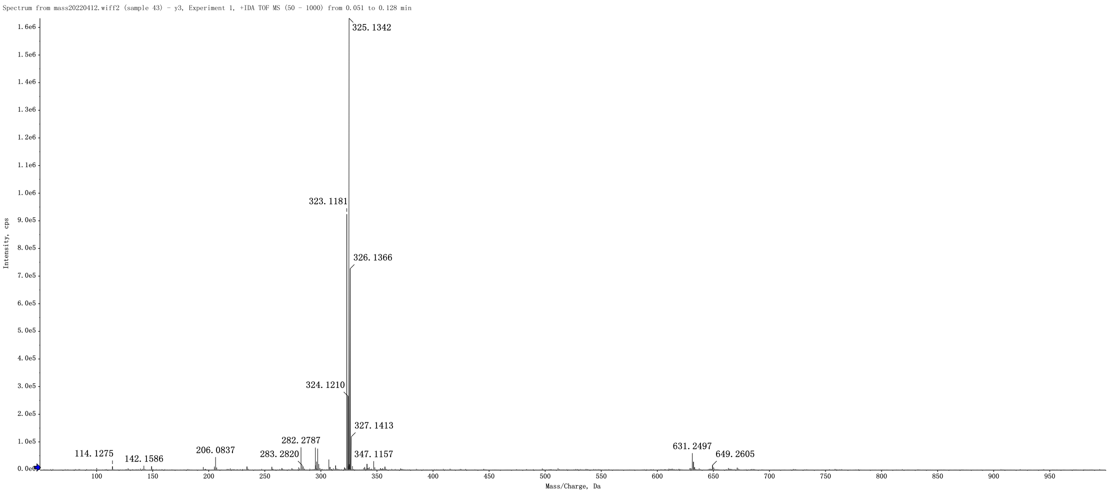

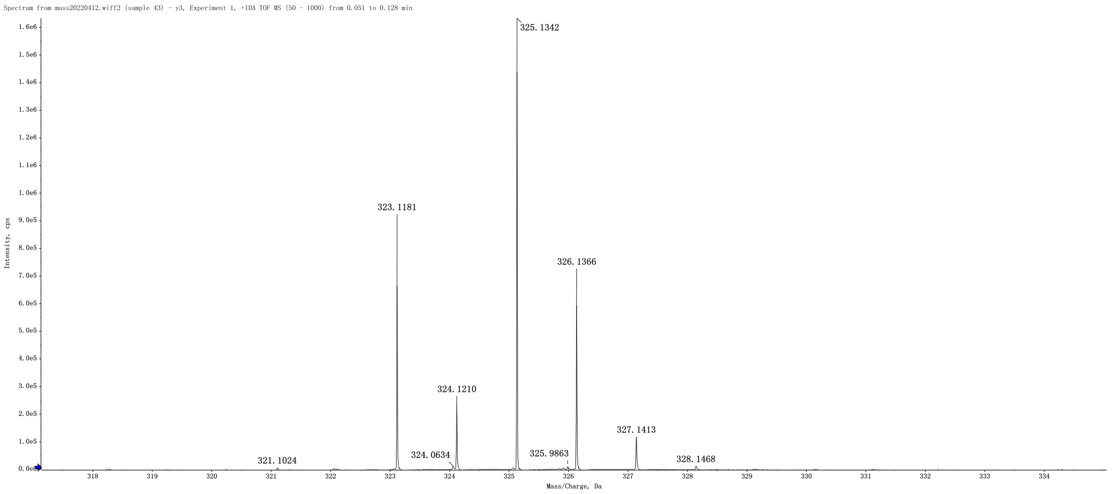

Hit	Formula	m/z	RDB	ppm	MS Rank	MSMS ppm	MSMS Rank	Found
1	C22H16N2O	325.1335	16.0	2.0	1			NA/NA

HR-MS spectrum of 7m

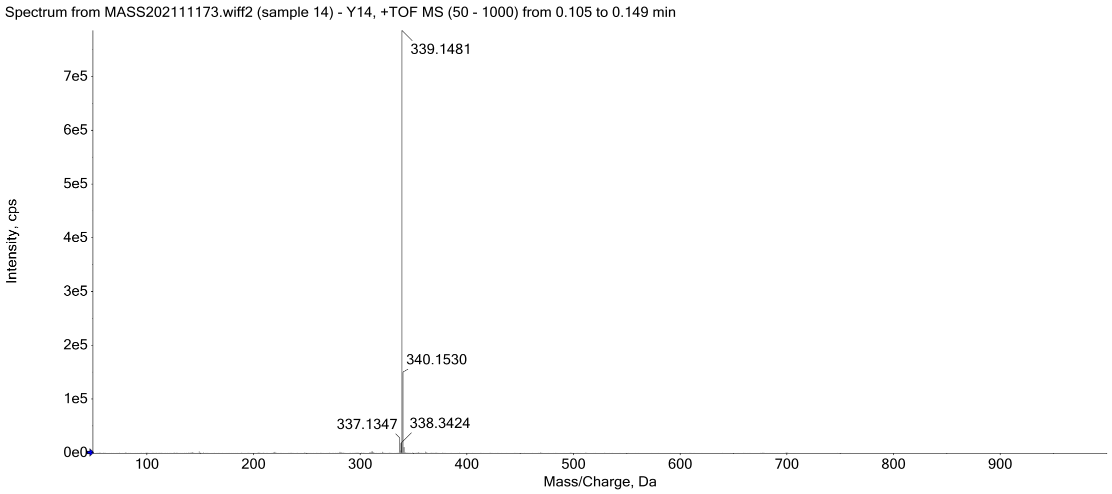

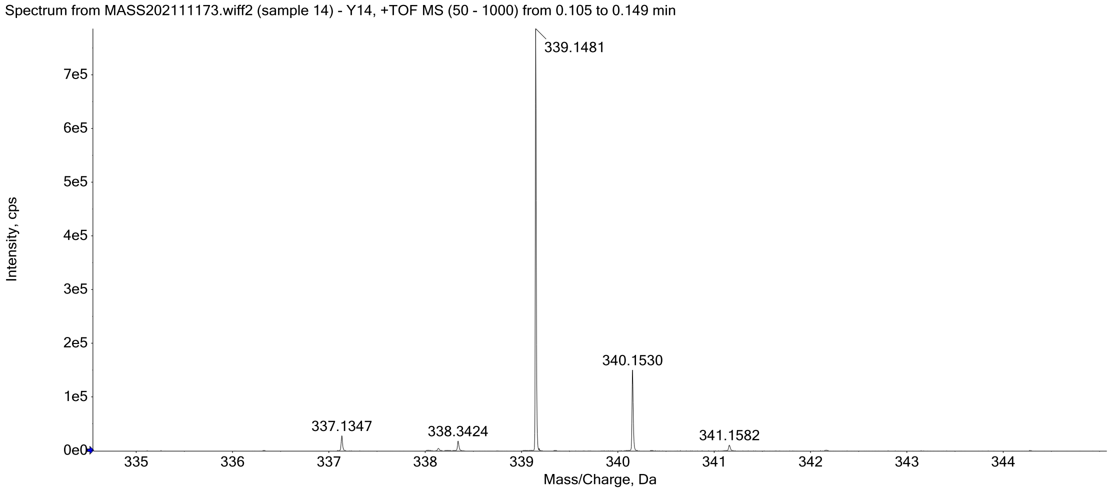

Spectrum from MASS202111173.wiff2 (sample 14) - Y14, +TOF MS (50 - 1000) from 0.105 to 0.149 min

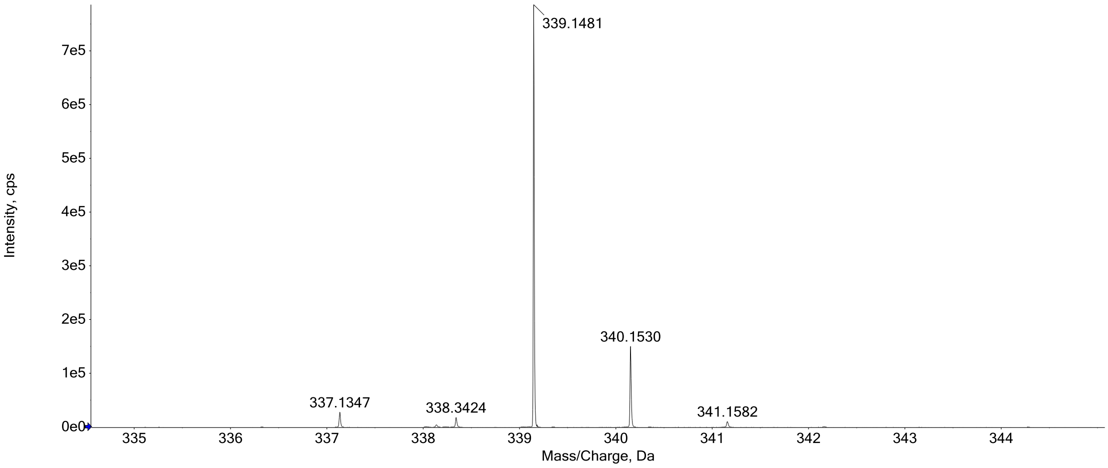

Spectrum from MASS202111173.wiff2 (sample 14) - Y14, +TOF MS (50 - 1000) from 0...02111173.wiff2 (sample 14) - Y14, +TOF MS (50 - 1000) from 0.237 to 0.299 min]

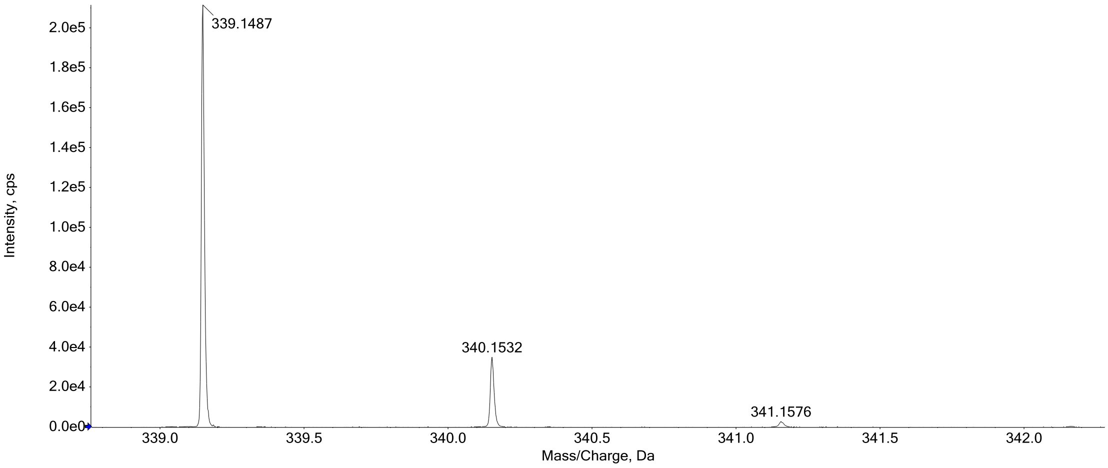

Hit	Formula	m/z	RDB	ppm	MS Rank	MSMS ppm	MSMS Rank	Found
1	C23H18N2O	339.1492	16.0	-1.4	1			NA/NA

HR-MS spectrum of 7n

Spectrum from MASS202111173.wiff2 (sample 2) - Y2, +TOF MS (50 - 1000) from 0.096 to 0.140 min

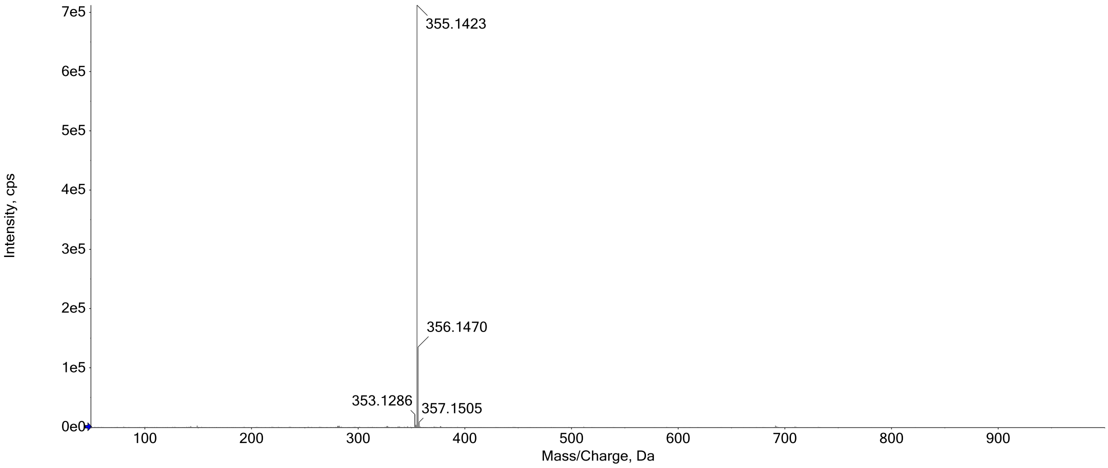

Spectrum from MASS202111173.wiff2 (sample 2) - Y2, +TOF MS (50 - 1000) from 0.096 to 0.140 min

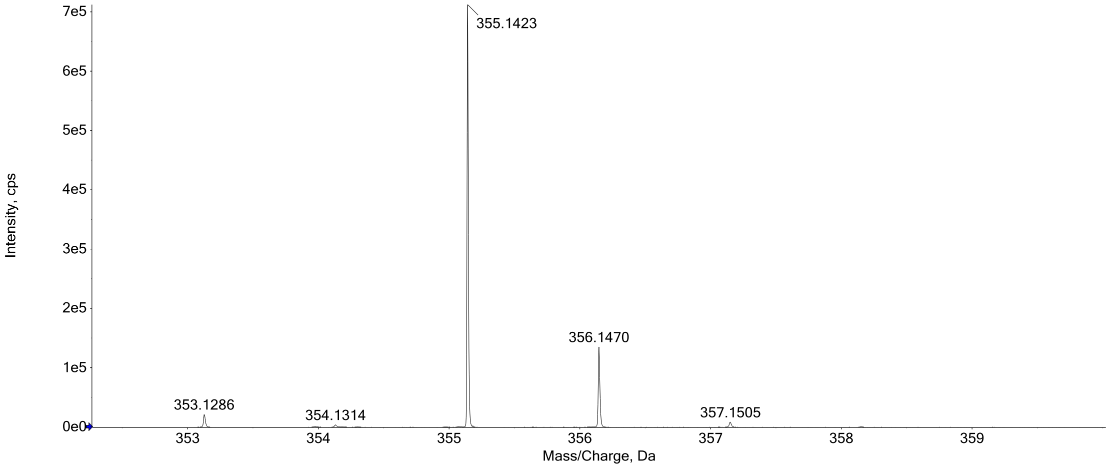

Spectrum from MASS202111173.wiff2 (sample 2) - Y2, +TOF MS (50 - 1000) from 0.1...S202111173.wiff2 (sample 2) - Y2, +TOF MS (50 - 1000) from 0.237 to 0.299 min]

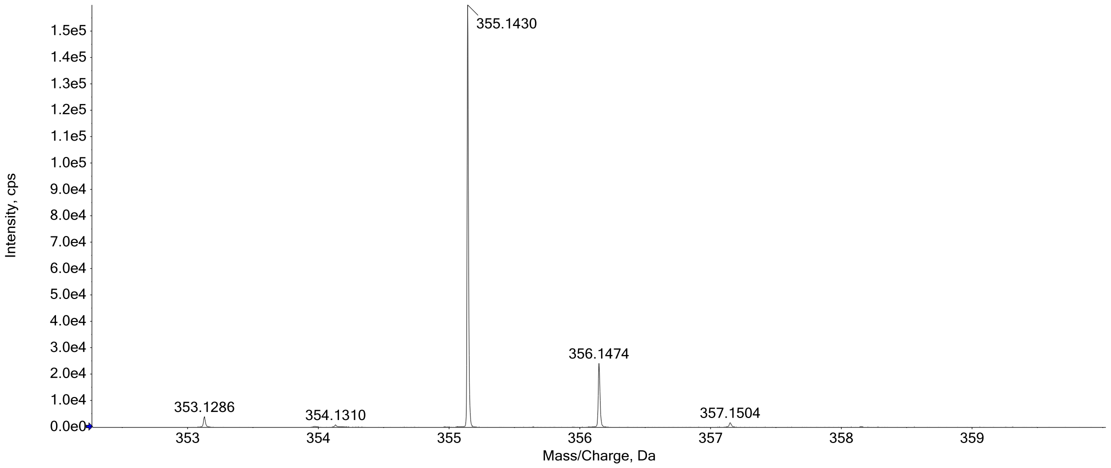

Hit	Formula	m/z	RDB	ppm	MS Rank	MSMS ppm	MSMS Rank	Found
1	C23H18N2O2	355.1441	16.0	-3.1	1			NA/NA

HR-MS spectrum of 7o

Spectrum from MASS202111173.wiff2 (sample 4) - Y4, +TOF MS (50 - 1000) from 0.149 to 0.193 min

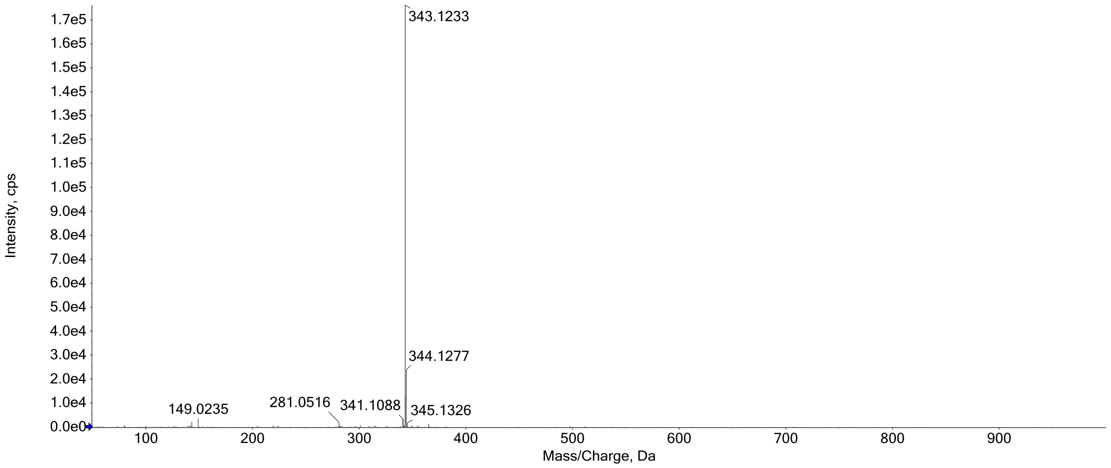

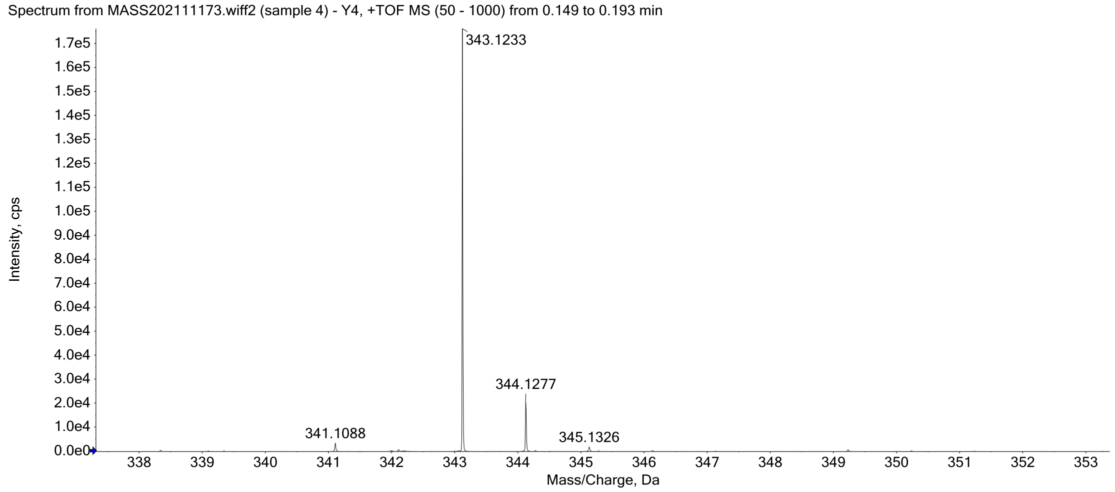

Hit	Formula	m/z	RDB	ppm	MS Rank	MSMS ppm	MSMS Rank	Found
1	C22H15FN2O	343.1241	16.0	-2.4	1			NA/NA

HR-MS spectrum of 7p

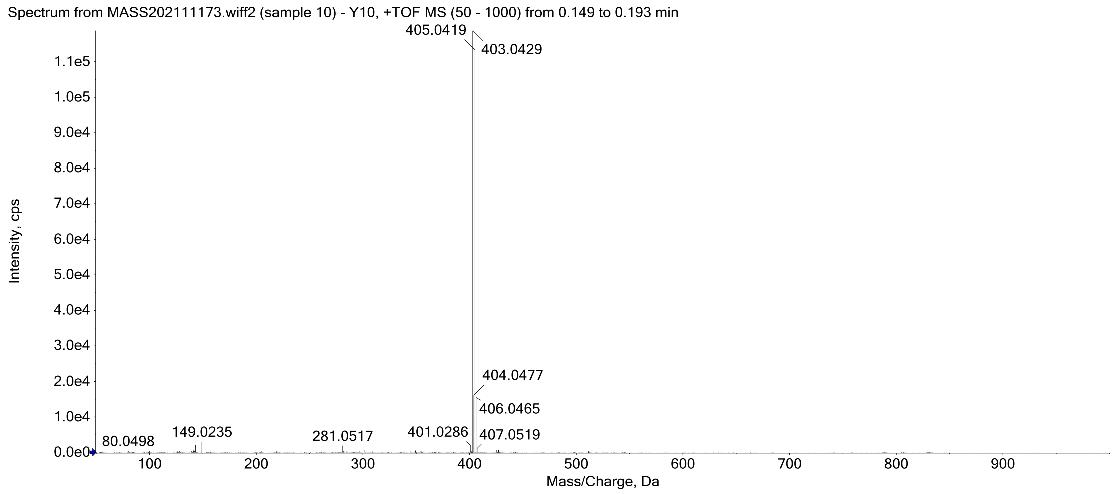

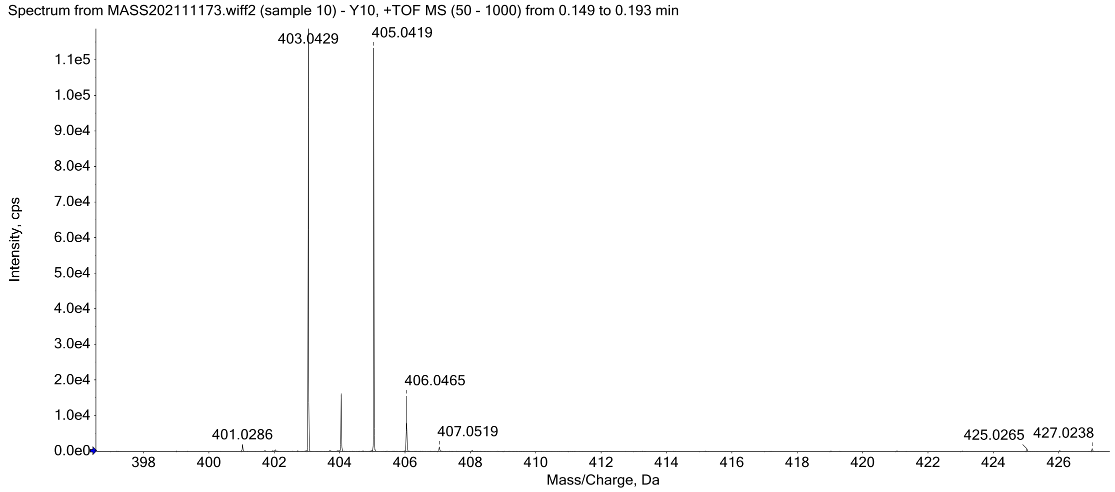

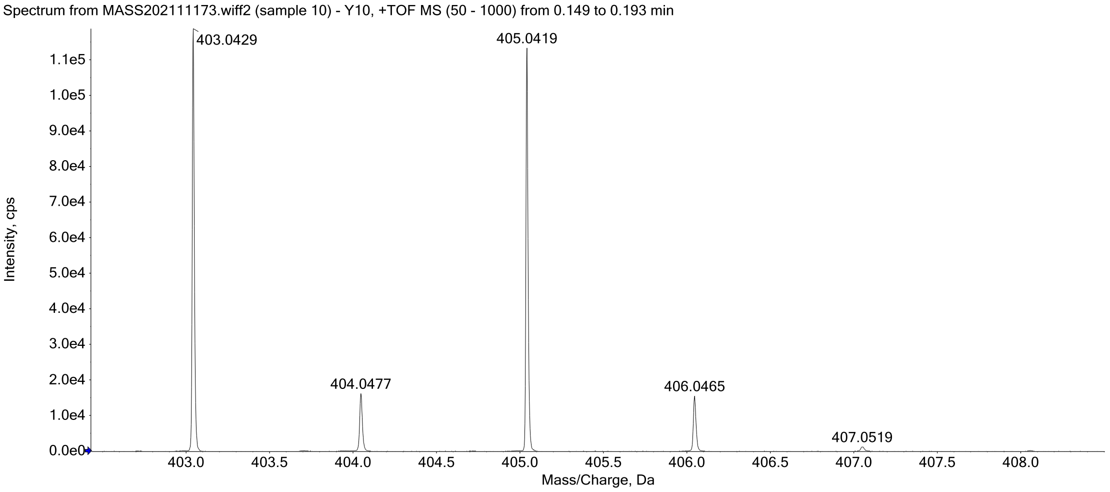

Hit	Formula	m/z	RDB	ppm	MS Rank	MSMS ppm	MSMS Rank	Found
1	C22H15BrN2O	403.0441	16.0	-2.9	1			NA/NA

HR-MS spectrum of 7q

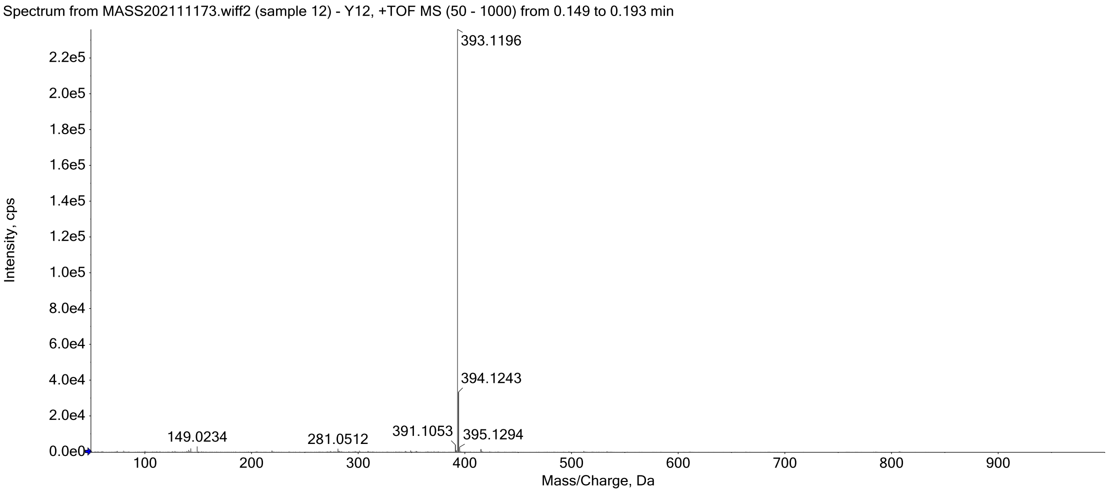

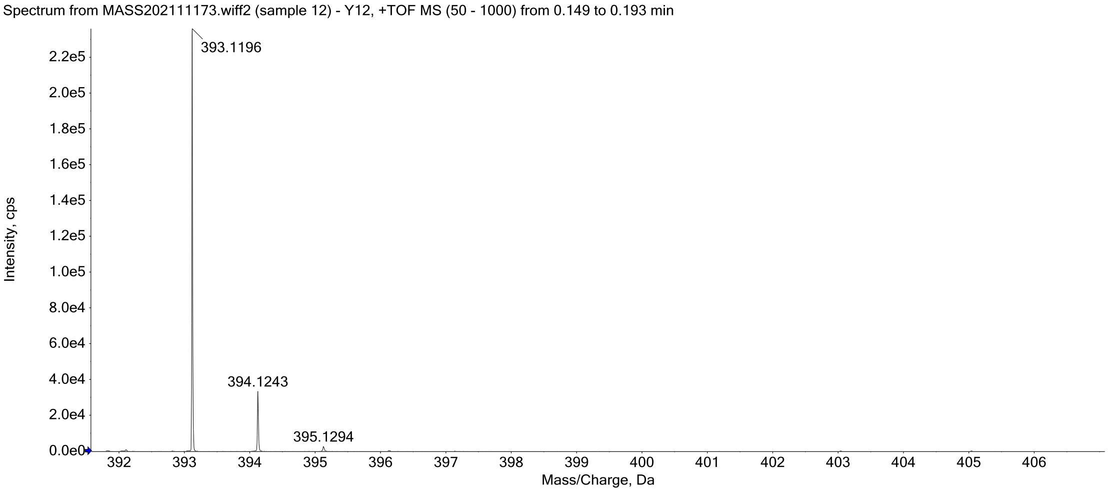

Hit	Formula	m/z	RDB	ppm	MS Rank	MSMS ppm	MSMS Rank	Found
1	C23H15F3N2O	393.1209	16.0	-3.4	1			NA/NA

HR-MS spectrum of 7r

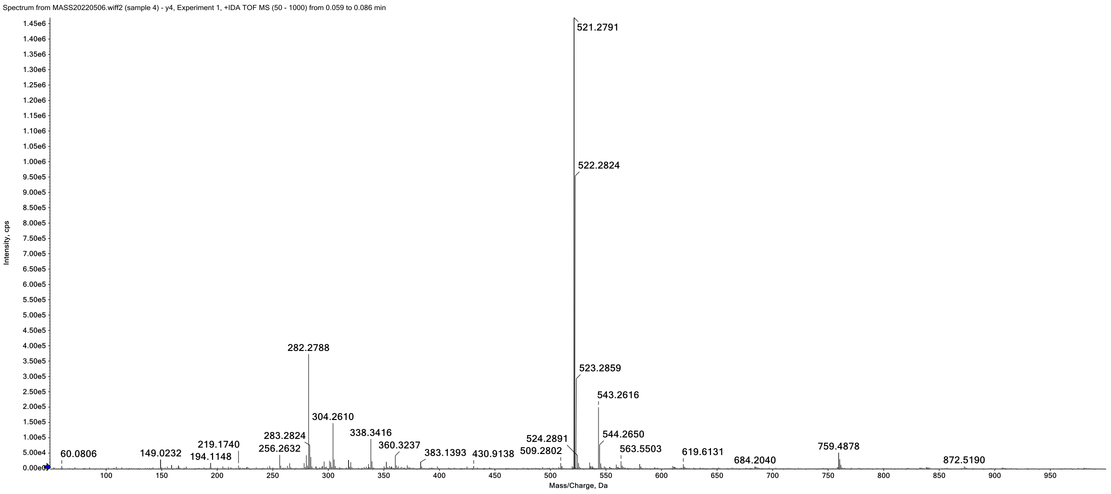

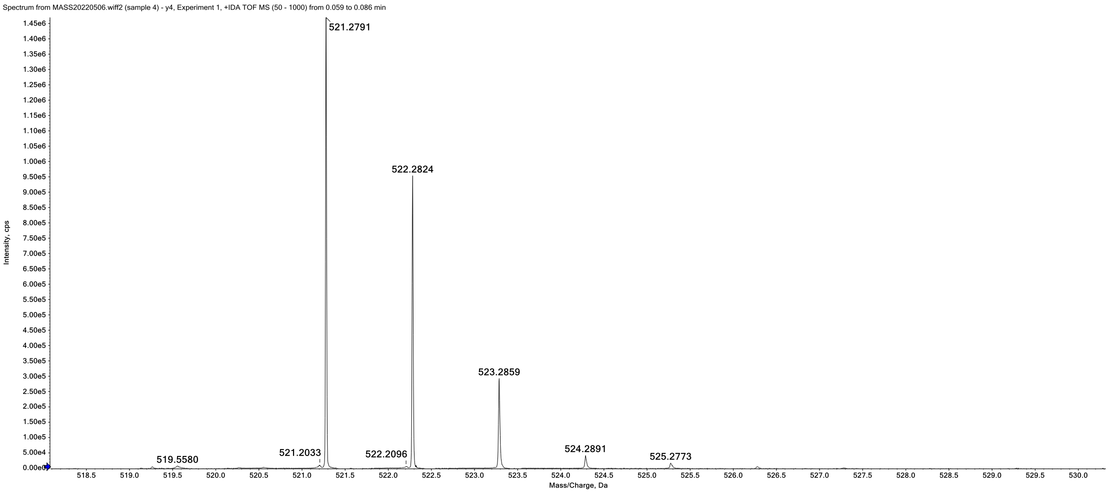

Hit	Formula	m/z	RDB	ppm	MS Rank	MSMS ppm	MSMS Rank	Found
1	C34H36N2O3	521.2799	18.0	-1.5	1			NA/NA

HR-MS spectrum of 7s

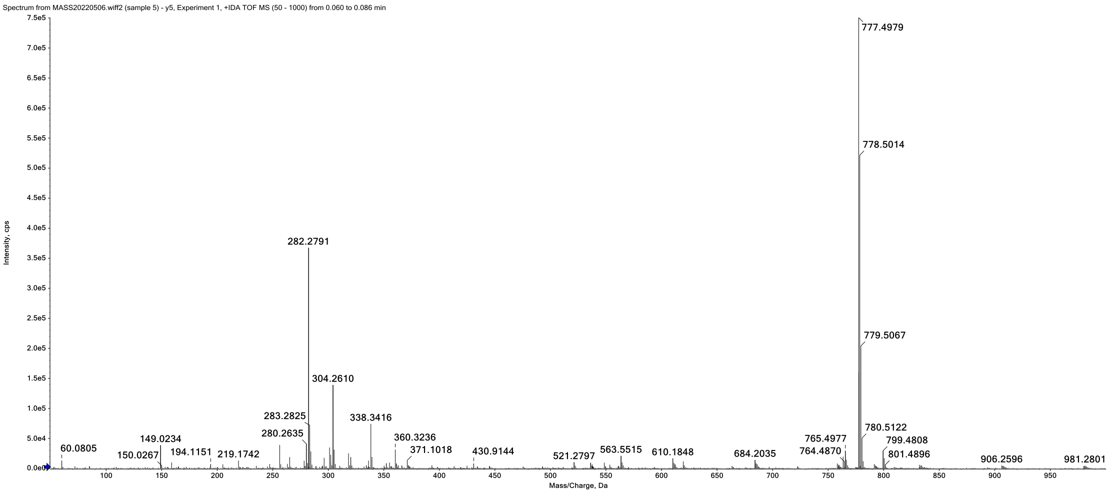

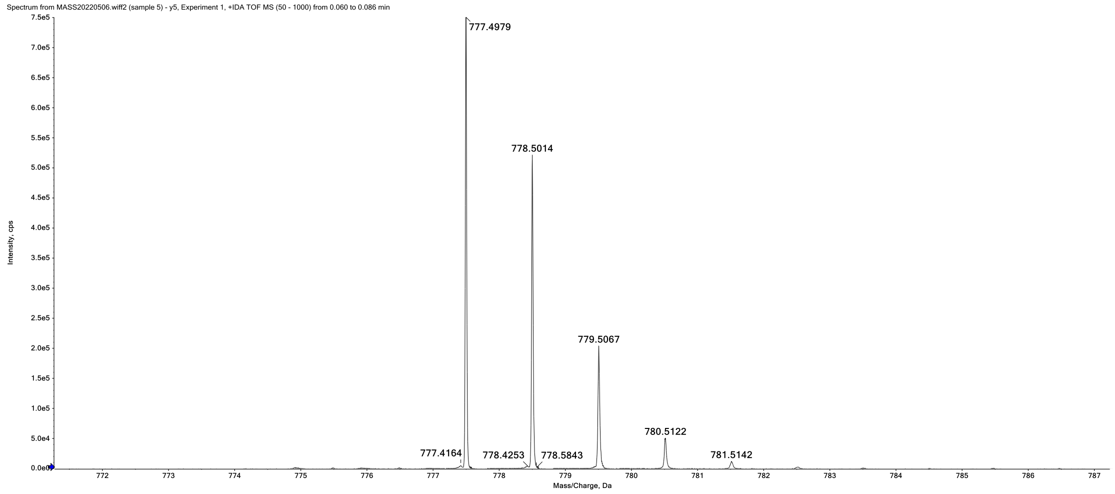

Hit	Formula	m/z	RDB	ppm	MS Rank	MSMS ppm	MSMS Rank	Found
1	C53H64N2O3	777.4990	23.0	-1.4	1			NA/NA

HR-MS spectrum of 7t

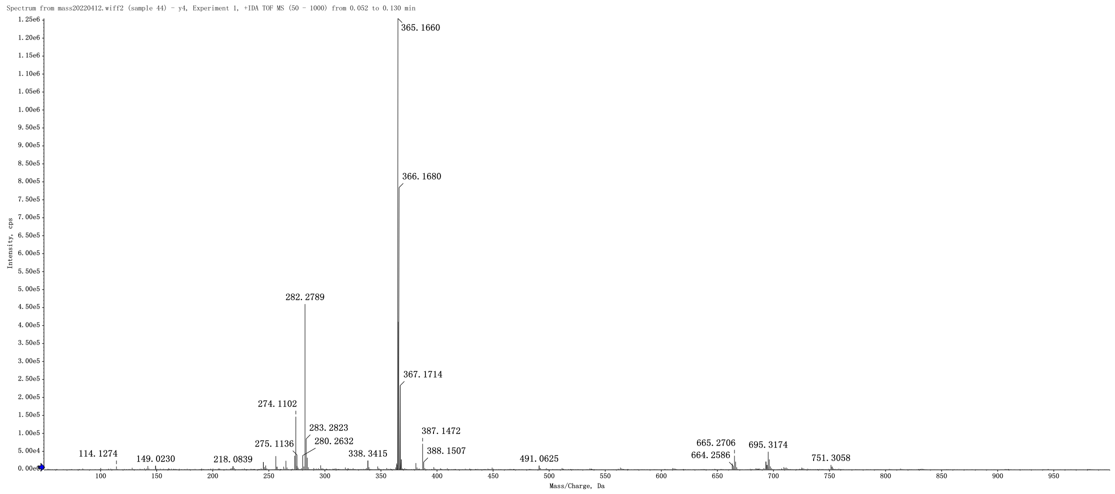

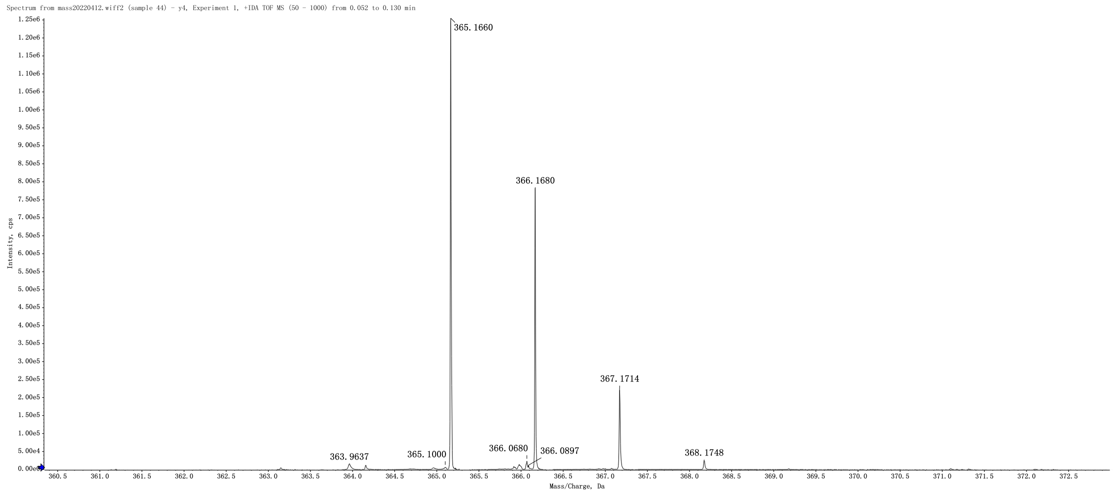

Hit	Formula	m/z	RDB	ppm	MS Rank	MSMS ppm	MSMS Rank	Found
1	C25H20N2O	365.1648	17.0	3.2	1			NA/NA

HR-MS spectrum of 7u

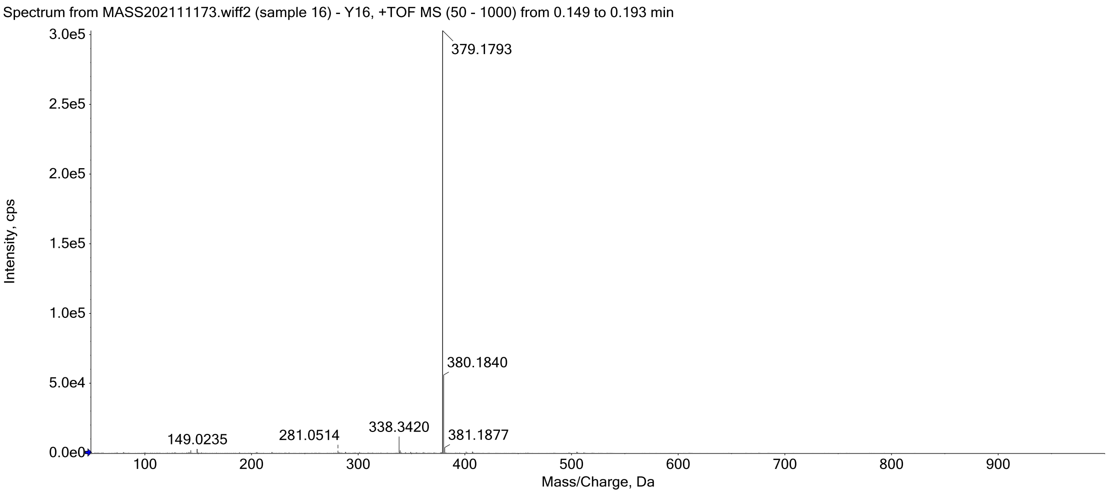

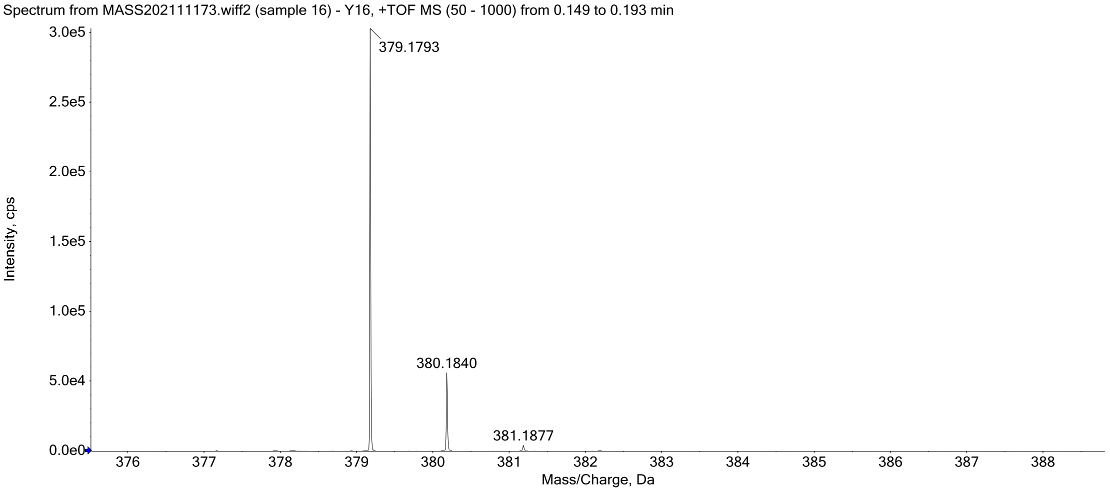

Hit	Formula	m/z	RDB	ppm	MS Rank	MSMS ppm	MSMS Rank	Found
1	C26H22N2O	379.1805	17.0	-3.1	1			NA/NA

HR-MS spectrum of 8

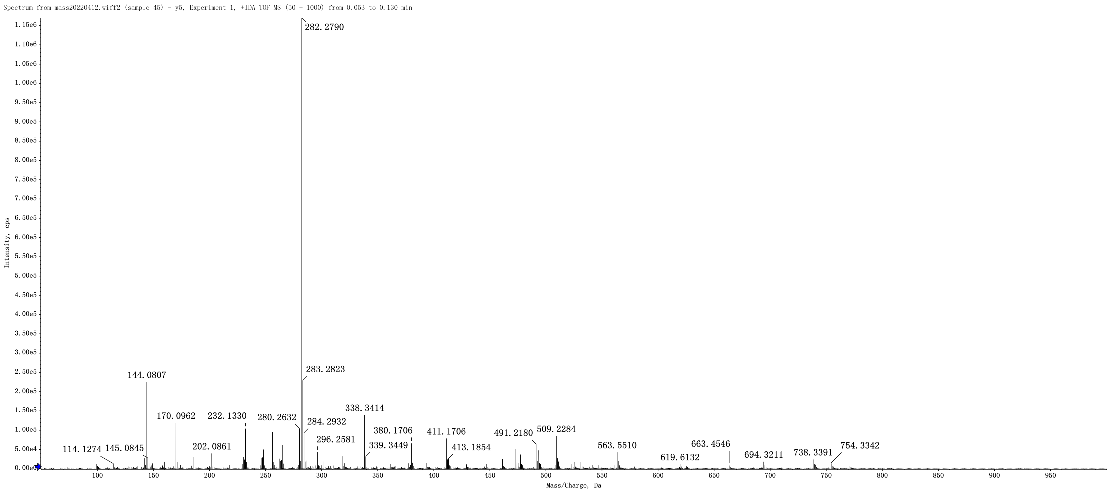

Spectrum from mass20220412.wiff2 (sample 45) - y5, Experiment 1, +IDA TOF MS (50 - 1000) from 0.037 to 0.112 min, subtracted by: [Spectrum from mass20220412.wiff2 (sample 45) - y5, Experiment 1, +IDA TOF MS (50 - 1000) from 0.309 to 0.386 min]

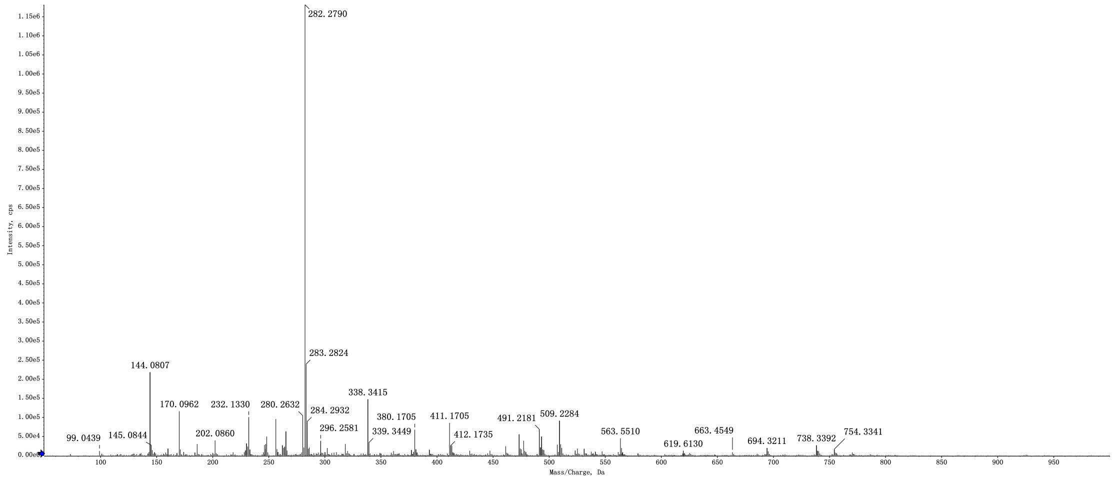

Spectrum from mass20220412.wiff2 (sample 45) - y5, Experiment 1, +IDA TOF MS (50 - 1000) from 0.037 to 0.112 min, subtracted by: [Spectrum from mass20220412.wiff2 (sample 45) - y5, Experiment 1, +IDA TOF MS (50 - 1000) from 0.309 to 0.386 min]

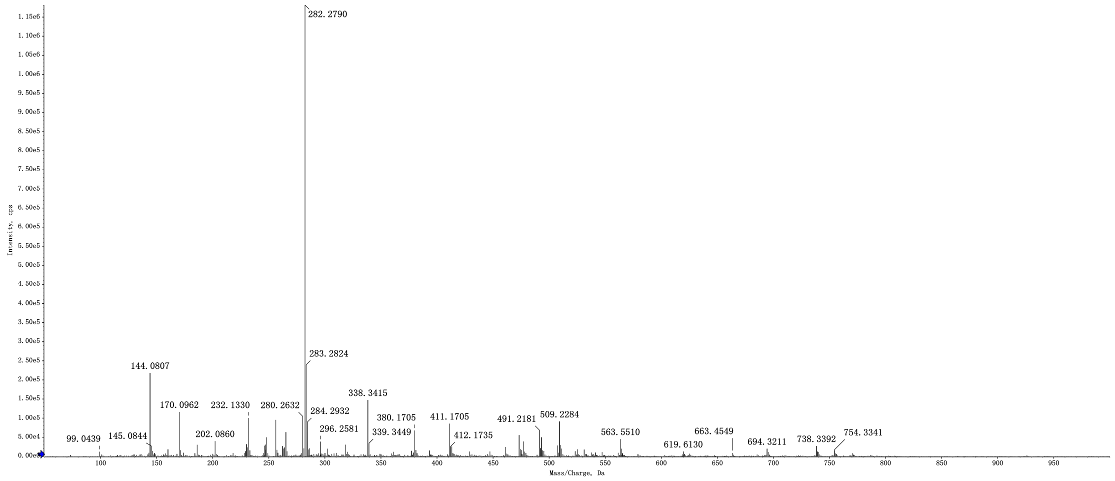

Spectrum from mass20220412.wiff2 (sample 45) - y5, Experiment 1, +IDA TOF MS (50 - 1000) from 0.037 to 0.112 min, subtracted by: [Spectrum from mass20220412.wiff2 (sample 45) - y5, Experiment 1, +IDA TOF MS (50 - 1000) from 0.309 to 0.386 min]

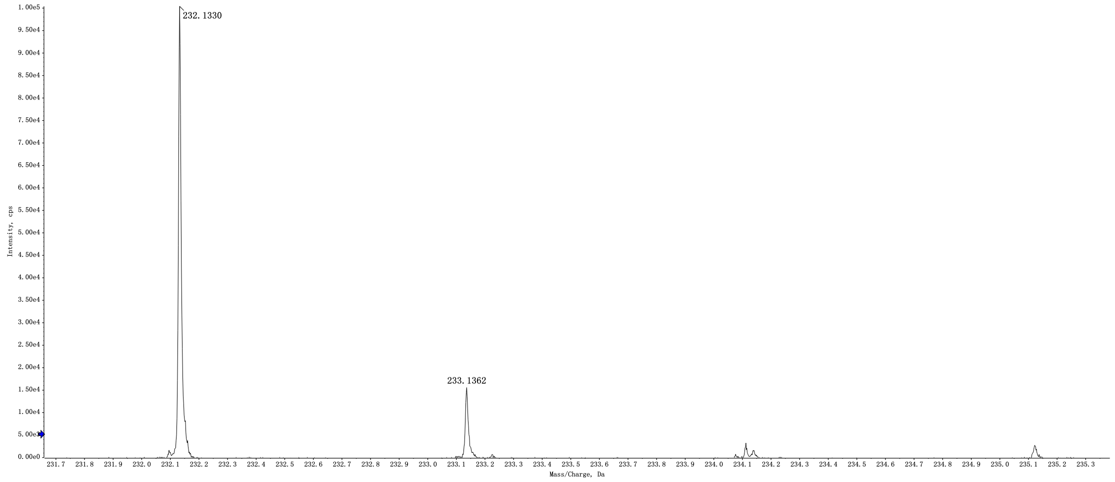

Hit	Formula	m/z	RDB	ppm	MS Rank	MSMS ppm	MSMS Rank	Found
1	C14H17NO2	232.1332	7.0	-0.9	1			NA/NA

HR-MS spectrum of 9

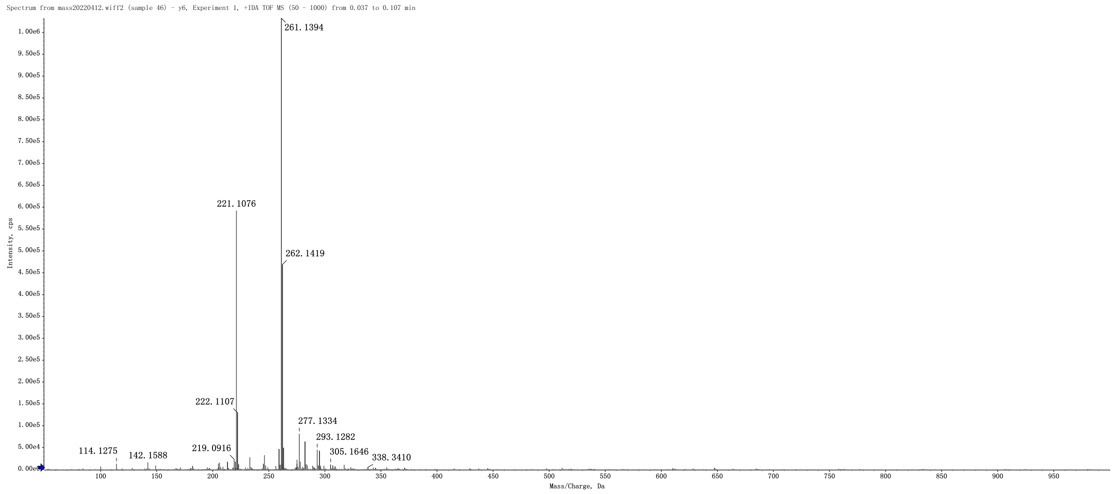

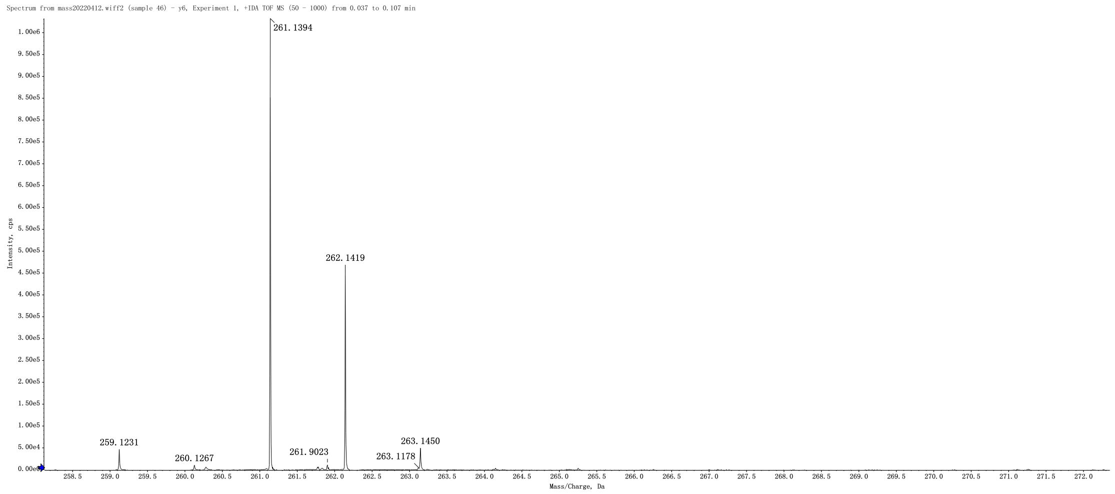

Hit	Formula	m/z	RDB	ppm	MS Rank	MSMS ppm	MSMS Rank	Found
1	C18H16N2	261.1386	12.0	3.0	1			NA/NA

HR-MS spectrum of 10

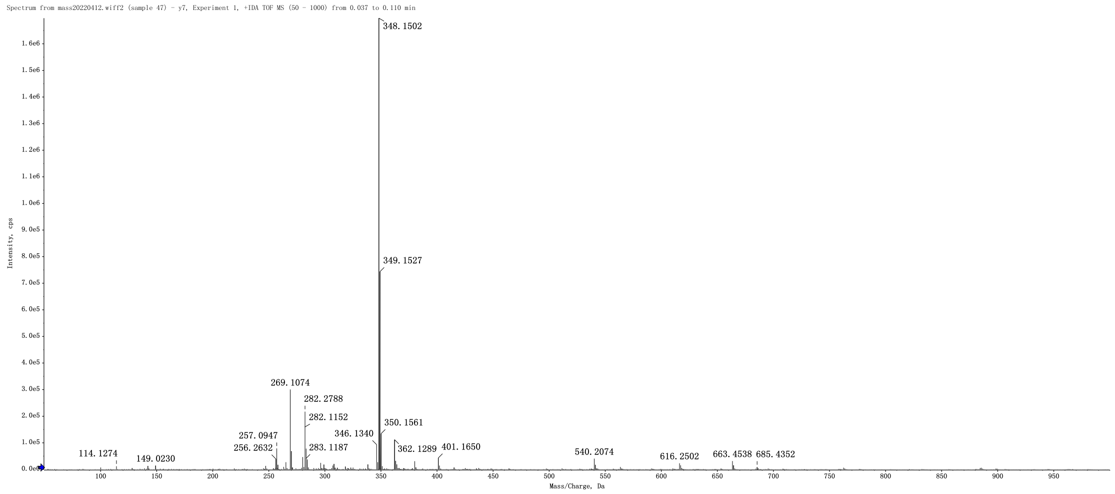

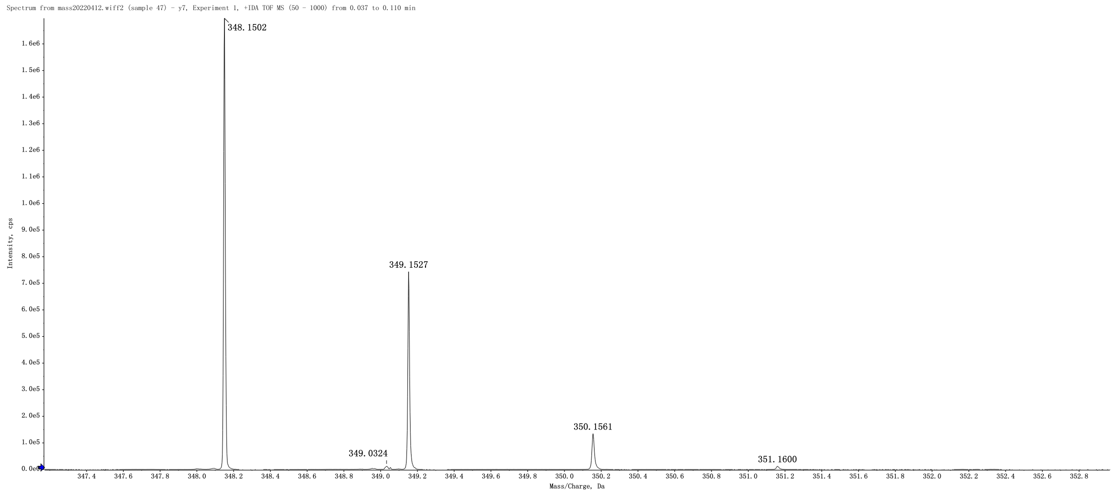

Hit	Formula	m/z	RDB	ppm	MS Rank	MSMS ppm	MSMS Rank	Found
1	C24H17N3	348.1495	18.0	1.9	1			NA/NA

HR-MS spectrum of 11

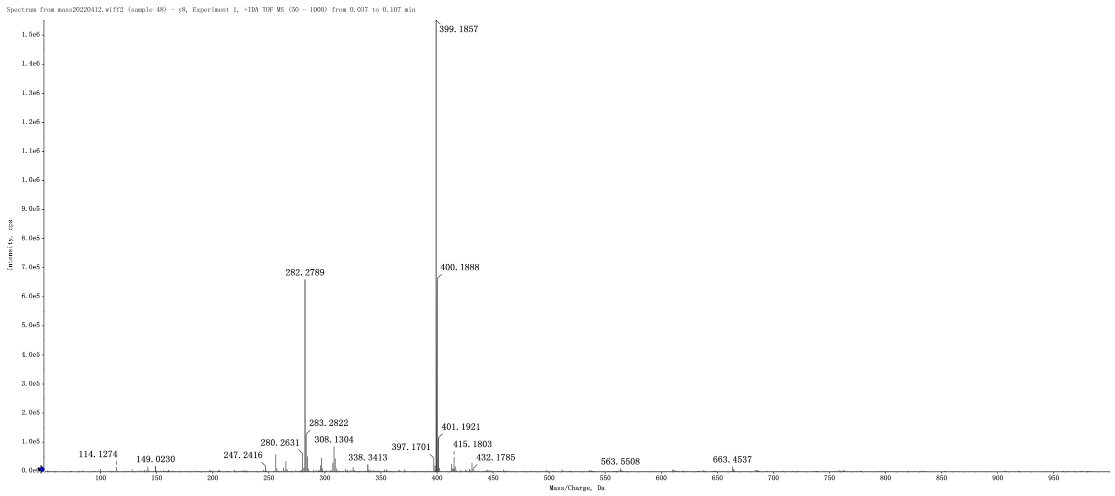

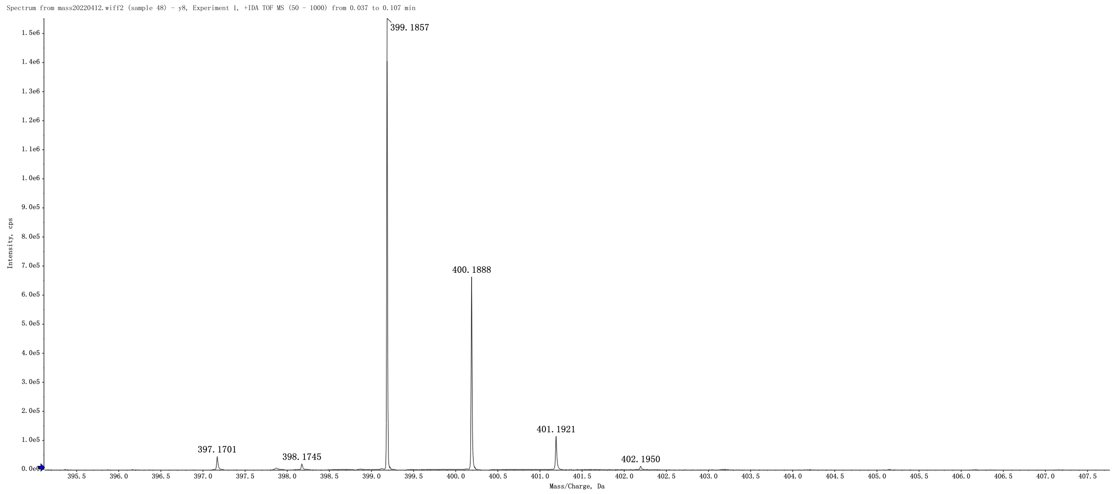

Hit	Formula	m/z	RDB	ppm	MS Rank	MSMS ppm	MSMS Rank	Found
1	C29H22N2	399.1856	20.0	0.3	1			NA/NA

HR-MS spectrum of 12

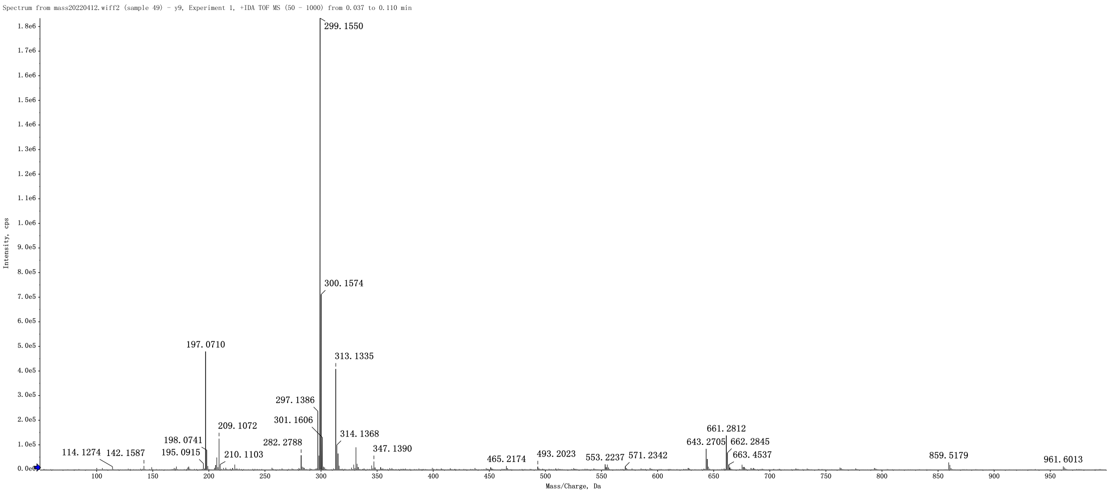

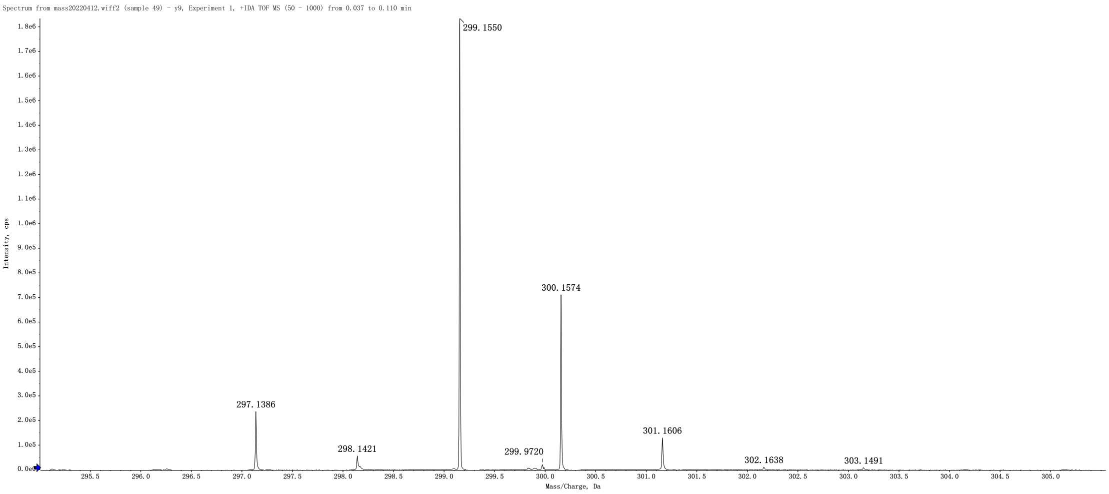

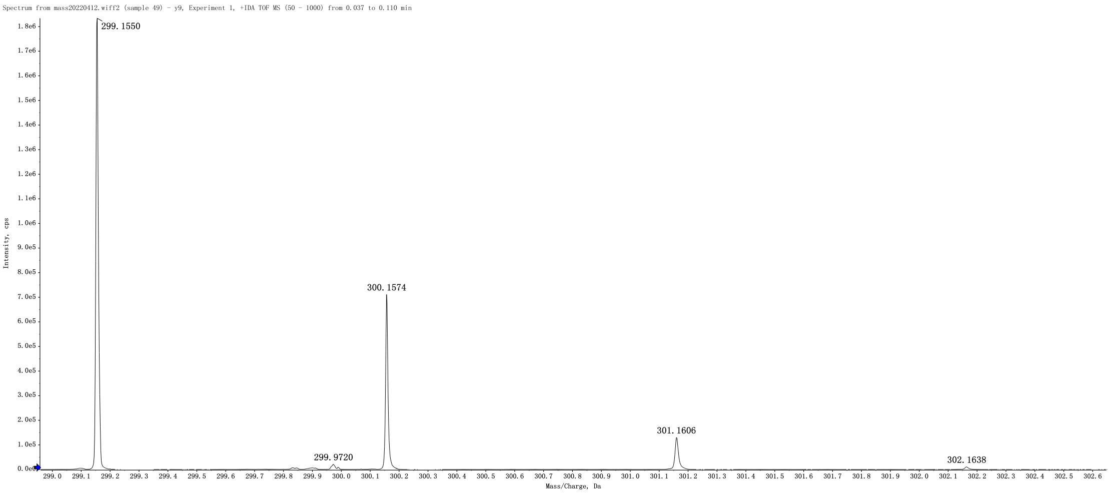

Hit	Formula	m/z	RDB	ppm	MS Rank	MSMS ppm	MSMS Rank	Found
1	C21H18N2	299.1543	14.0	2.4	1			NA/NA

HR-MS spectrum of 13

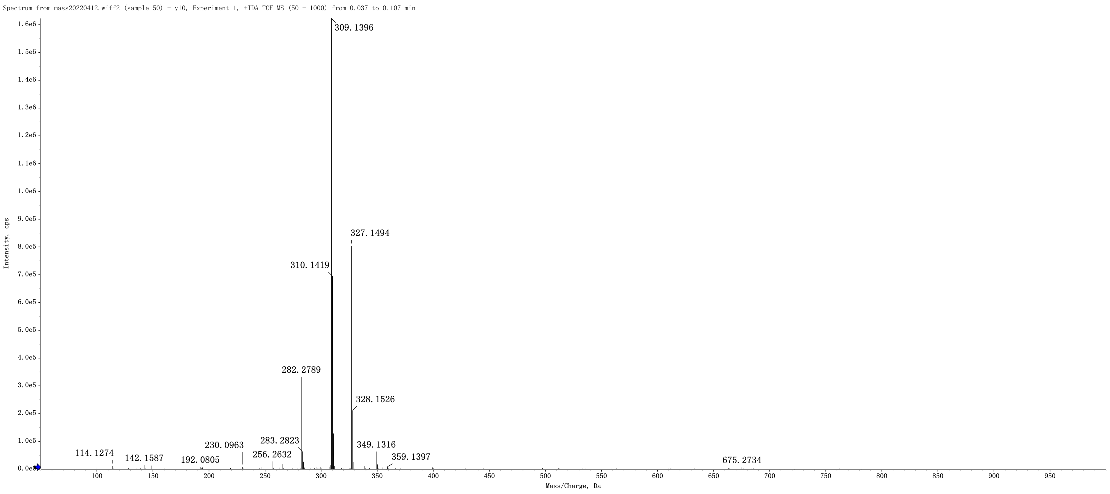

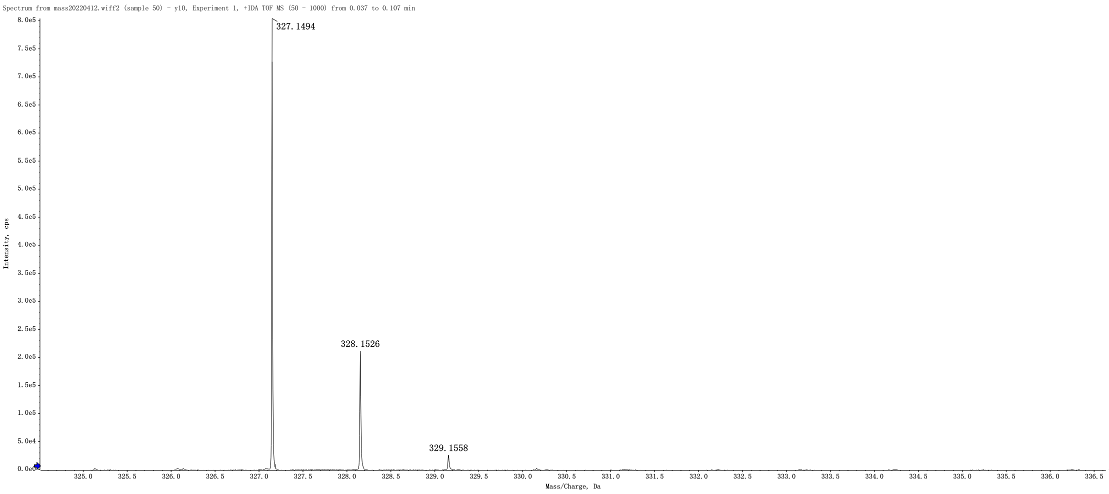

Hit	Formula	m/z	RDB	ppm	MS Rank	MSMS ppm	MSMS Rank	Found
1	C22H18N2O	327.1492	15.0	0.6	1			NA/NA

HR-MS spectrum of 14

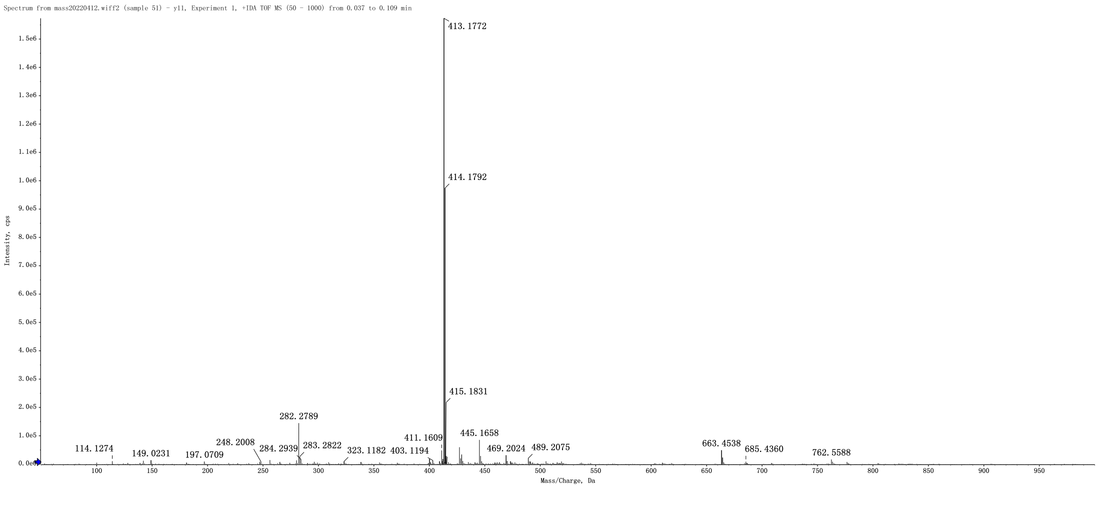

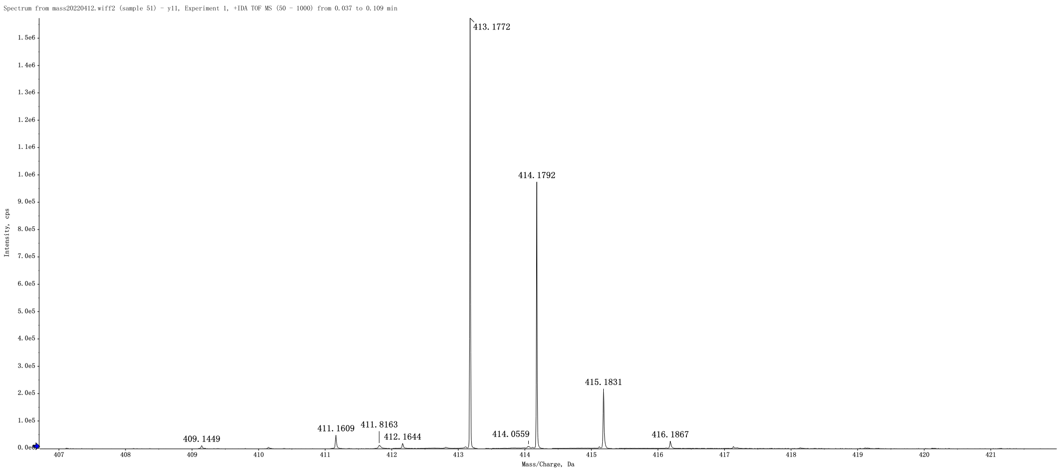

Hit	Formula	m/z	RDB	ppm	MS Rank	MSMS ppm	MSMS Rank	Found
1	C28H20N4	413.1761	21.0	2.7	1			NA/NA

IV. Supplementary References

1. (a) Hu, X., Shao, Y., Xie, H., Chen, X., Chen, F., Ke, Z., Jiang, H. & Zeng, W. Direct Carbon–Carbon σ Bond Amination of Unstrained Arylalkylketones. *ACS Catal*. **10**, 8402 – 8408 (2020); (b) Yang, P. & Bao, Y.-S. Palladium nanoparticles supported on organofunctionalized kaolin as an effcient heterogeneous catalyst for directed C – H functionalization of arylpyrazoles. *RSC Adv.* **7**, 53878–53886 (2017).

2. Zhu, C., Pinkert, T., Greßies, S. & Glorius, F. One-Pot C–H Formylation Enabled by Relay Catalysis of Manganese(I) and Iron(III). *ACS Catal*. **8**, 10036 – 10042 (2018).

3. Estopiñá-Durán, S., Mclean, E., Donnelly, L., Hockin, B. & Taylor, J. Arylboronic Acid Catalyzed C – Alkylation and Allylation Reactions Using Benzylic Alcohols. *Org. Lett.* **22**, 7547 – 7551 (2020).

4. Bai, J.-F., Yasumoto, K., Kano, T. & Maruoka, K. Asymmetric Synthesis of Chiral 1,4-Enynes through Organocatalytic Alkenylation of Propargyl Alcohols with Trialkenylboroxines. *Angew. Chem., Int. Ed*. **58**, 8898 – 8901 (2019).

5. Yang, B., Li, S.-J., Wang, Y., Lan, Y. & Zhu, S. Hydrogen radical-shuttle (HRS)-enabled photoredox synthesis of indanones via decarboxylative annulation. *Nat. Commun.* **12**, 5257 – 5268 (2021).

6. Tiwari, V., Kamal, N. & Kapur, M. Ruthenium-Catalyzed Heteroatom-Directed Regioselective C–H Arylation of Indoles Using a Removable Tether. *Org. Lett.* **17**, 1766 – 1769 (2015).

7. Li, J., Zhang, Z., Ma, W., Tang, M., Wang, D. & Zou, L.-H. Mild Cobalt (III)-Catalyzed C–H Hydroarylation of Conjugated C=C/C=O Bonds. *Adv. Synth. Catal.* **359**, 1717 – 1724 (2017).

8. Yang, C., Liu, Z., Hu, X., Xie, H., Jiang, H. & Zeng, W. Rh(III)-Catalyzed Csp^2 – Csp^3 bond alkoxylation of α-indolyl alcohols via C – C σ bond cleavage. *Org. Chem. Front*., **8**, 2949 – 2954 (2021).

9. Liu, Y., Xu, W. & Wang, X. Gold(I)-Catalyzed Tandem Cyclization Approach to Tetracyclic Indolines. *Org. Lett.* **12**, 1448 – 1451 (2010).

10. Lee, H., Kim, S. & Kim, J. Facile One-Pot Synthesis of Cinnamamides from Aromatic Aldehydes and Acetonitrile with Me_3SiOK. *Bull. Kor. Chem. Soc*. **32**, 1748 – 1750 (2011).

11. Wen, X.-M., Tao, L., Xiang, Y.-Z., Fang, Y.-G. & Yu, X.-Q. A facile synthesis of trans-alkenes in micellar media. *ARKIVOC* **13**, 169 – 174 (2005).

12. Kim, Y., Park, S. & Kim, J. Cobalt-Catalyzed Direct $C(sp^2)$–H Alkylation with Unactivated Alkenes. *Eur. J. Org. Chem*. **2020**, 4026 – 4030 (2020).

13. Shintani, R., Fujie, R., Takeda, M. & Nozaki, K. Silylative Cyclopropanation of Allyl Phosphates with Silylboronat. *Angew. Chem., Int. Ed*. **126**, 6664 – 6667 (2014).

14. Annes, S., Vairaprakash, P. & Ramesh, S. TfOH mediated intermolecular electrocyclization for the synthesis of pyrazolines and its application in alkaloid synthesis. *RSC Adv.* **8**, 30071 – 30075 (2018).

15. Gaussian 09, Revision D.01; Frisch, M. J.; Trucks, G. W.; Schlegel, H. B.; Scuseria, G. E.; Robb, M. A.; Cheeseman, J. R.; Scalmani, G.; Barone, V.; Mennucci, B.; Petersson, G. A.; Nakatsuji, H.; Caricato, M.; Li, X.; Hratchian, H. P.; Izmaylov, A. F.; Bloino, J.; Zheng, G.; Sonnenberg, J. L.; Hada, M.; Ehara, M.; Toyota, K.; Fukuda, R.; Hasegawa, J.; Ishida, M.; Nakajima, T.; Honda, Y.; Kitao, O.; Nakai, H.; Vreven, T.; Montgomery, J. A., Jr.; Peralta, J. E.; Ogliaro, F.; Bearpark, M.; Heyd, J. J.; Brothers, E.; Kudin, K. N.; Staroverov, V. N.; Kobayashi, R.; Normand, J.; Raghavachari, K.; Rendell, A.; Burant, J. C.; Iyengar, S. S.; Tomasi, J.; Cossi, M.; Rega, N.; Millam, M. J.; Klene, M.; Knox, J. E.; Cross, J. B.; Bakken, V.; Adamo, C.; Jaramillo, J.; Gomperts, R.; Stratmann, R. E.; Yazyev, O.; Austin, A. J.; Cammi, R.; Pomelli, C.; Ochterski, J. W.; Martin, R. L.; Morokuma, K.; Zakrzewski, V. G.; Voth, G. A.; Salvador, P.; Dannenberg, J. J.; Dapprich, S.; Daniels, A. D.; Farkas, Ö .; Foresman, J. B.; Ortiz, J. V.; Cioslowski, J.; Fox, D. J.: Gaussian,Inc., Wallingford CT, **2009**.

16. Zhao, Y. & Truhlar, D. G. Density functionals with broad applicability in chemistry. *Acc. Chem. Res.* 41, 157 – 167 (2008).

17. Zhao, Y. & Truhlar, D. G. The M06 suite of density functionals for main group thermochemistry, thermochemical kinetics, noncovalent interactions, excited states, and transition elements: two new functionals and systematic testing of four M06-class functionals and 12 other functionals. *Theor. Chem. Acc.* **120**, 215 – 241 (2008).

18. Wang, C., Wang, J., Cai, Q., Li, Z., Zhao, H.-K. & Luo, R.,Exploring accurate Poisson–Boltzmann methods for biomolecular simulations. *Comput. Theor. Chem.* **1024**, 34 – 44 (2013).

19. Weigend, F. & Ahlrichs, R. Balanced basis sets of split valence, triple zeta valence and quadruple zeta valence quality for H to Rn: Design and assessment of accuracy. *Phys. Chem. Chem. Phys.* **7**, 3297 – 3305 (2005).

20. Fukui, K. The path of chemical reactions-the IRC approach. *Acc. Chem. Res.* **14**, 363 – 368 (1981).

21. Weigend, F. Accurate Coulomb-fitting basis sets for H to Rn. *Phys. Chem. Chem. Phys.* **8**, 1057 – 1065 (2006).

22. Marenich, A. V., Cramer, C. J. & Truhlar, D. G. Universal Solvation Model Based on Solute Electron Density and on a Continuum Model of the Solvent Defined by the Bulk Dielectric Constant and Atomic Surface Tensions. *J. Phys. Chem. B*. **113**, 6378 – 6396 (2009).

23. Besora, M., Vidossich, P., Lledós, A., Ujaque, G. & Maseras, F. Calculation of Reaction Free Energies in Solution: A Comparison of Current Approaches. *J. Phys. Chem. A.* **122**, 1392 – 1399 (2018).

24. Lu, T. & Chen, F. Multiwfn: a multifunctional wavefunction analyzer. *J. Comput. Chem.* **33**, 580 – 592 (2012).
