# Supplementary material for: Mn(I)-catalyzed sigmatropic rearrangement of β, γ-unsaturated alcohols
Source: Nat Commun. 2023 Apr 3;14:1862. doi: 10.1038/s41467-023-37299-x (PMC10070501; doi:10.1038/s41467-023-37299-x)
Supplement: Supplementary file 3 — Description of Additional Supplementary Files [file 41467_2023_37299_MOESM3_ESM.pdf]

Supplementary Data Legends

The Supplementary Data include the following materials

Data-I: All the original ^{1}H NMR and ^{13}C NMR spectral data from this manuscript.

Data-II: All the original HR-MS spectral data from this manuscript.

Data-III: All the original IR spectral data from this manuscript.

Data-IV: Cartesian coordinates about the DFT calculations from this manuscript.

Data-V: The original single crystal data about compounds 5 m, 7f and 7t.
